# Extensive microbial diversity within the chicken gut microbiome revealed by metagenomics and culture

**DOI:** 10.7717/peerj.10941

**Published:** 2021-04-06

**Authors:** Rachel Gilroy, Anuradha Ravi, Maria Getino, Isabella Pursley, Daniel L. Horton, Nabil-Fareed Alikhan, Dave Baker, Karim Gharbi, Neil Hall, Mick Watson, Evelien M. Adriaenssens, Ebenezer Foster-Nyarko, Sheikh Jarju, Arss Secka, Martin Antonio, Aharon Oren, Roy R. Chaudhuri, Roberto La Ragione, Falk Hildebrand, Mark J. Pallen

**Affiliations:** 1 Quadram Institute Bioscience, Norwich, UK; 2 School of Veterinary Medicine, University of Surrey, Guildford, UK; 3 Earlham Institute, Norwich Research Park, Norwich, UK; 4 University of East Anglia, Norwich, UK; 5 Roslin Institute, University of Edinburgh, Edinburgh, UK; 6 Medical Research Council Unit The Gambia at the London School of Hygiene and Tropical Medicine, Atlantic Boulevard, Banjul, The Gambia; 7 West Africa Livestock Innovation Centre, Banjul, The Gambia; 8 Department of Plant and Environmental Sciences, The Alexander Silberman Institute of Life Sciences, Edmond J. Safra Campus, Hebrew University of Jerusalem, Jerusalem, Israel; 9 Department of Molecular Biology and Biotechnology, University of Sheffield, Sheffield, UK

**Keywords:** Chickens, Gut microbiome, Biodiversity, Metagenomics, Metagenome-assembled genome, Bacterial nomenclature, Uncultured bacteria, Candidatus

## Abstract

**Background:**

The chicken is the most abundant food animal in the world. However, despite its importance, the chicken gut microbiome remains largely undefined. Here, we exploit culture-independent and culture-dependent approaches to reveal extensive taxonomic diversity within this complex microbial community.

**Results:**

We performed metagenomic sequencing of fifty chicken faecal samples from two breeds and analysed these, alongside all (*n* = 582) relevant publicly available chicken metagenomes, to cluster over 20 million non-redundant genes and to construct over 5,500 metagenome-assembled bacterial genomes. In addition, we recovered nearly 600 bacteriophage genomes. This represents the most comprehensive view of taxonomic diversity within the chicken gut microbiome to date, encompassing hundreds of novel candidate bacterial genera and species. To provide a stable, clear and memorable nomenclature for novel species, we devised a scalable combinatorial system for the creation of hundreds of well-formed Latin binomials. We cultured and genome-sequenced bacterial isolates from chicken faeces, documenting over forty novel species, together with three species from the genus *Escherichia*, including the newly named species *Escherichia whittamii*.

**Conclusions:**

Our metagenomic and culture-based analyses provide new insights into the bacterial, archaeal and bacteriophage components of the chicken gut microbiome. The resulting datasets expand the known diversity of the chicken gut microbiome and provide a key resource for future high-resolution taxonomic and functional studies on the chicken gut microbiome.

## Introduction

The domestic chicken is the most abundant bird and most abundant food animal on Earth, accounting for a larger fraction of the planet’s biomass than all species of wild birds combined (Bennett et al., 2018). Consumption of chicken meat is growing faster than any other type of meat and is seen as a cheaper, healthier, low-carbon alternative to meat from mammalian livestock (Eshel et al., 2014; Willett et al., 2019). Chicken eggs remain a nutritious, affordable food across the globe (Réhault-Godbert, Guyot & Nys, 2019).

The chicken gastrointestinal tract is home to a complex community of microbes and their genes—the chicken gut microbiome—that underpins links between diet, health and productivity in poultry, as evidenced by the ability of antibiotics to promote growth in chicks (Bedford, 2000). This microbial community also acts as a source of pathogens associated with disease in birds or in humans—including *Campylobacter*, *Salmonella*, and *Escherichia coli*—as well as providing a reservoir of antimicrobial resistance genes (Florez-Cuadrado et al., 2018; Jørgensen et al., 2019; Hermans et al., 2012).

Previous studies of this community have documented a rich variety of microorganisms (dominated by bacteria, but including viruses, archaea and microbial eukaryotes) and have shown that the taxonomic composition of this community varies with age, breed and disease status (Shang et al., 2018; Rychlik, 2020). However, these earlier efforts have largely relied on analyses of molecular barcodes (in particular short 16S rRNA gene sequences), which fail to provide species-level resolution, are unable to detect viruses and reveal nothing about the genome sequences, population structures or functional repertoires of microbial species (Hillmann et al., 2018).

Two strategies have proven productive for exploring taxonomic and functional diversity in complex microbial communities (Almeida et al., 2019; Forster et al., 2019). Culture-independent approaches rely on shotgun metagenomic sequencing of DNA extracted from relevant samples, followed by bioinformatics-based community profiling and analysis (Glendinning et al., 2020; Sergeant et al., 2014). Culture-dependent approaches combine large-scale isolation of microorganisms in pure culture with whole-genome sequencing and phylogenomic analysis (Medvecky et al., 2018). To explore taxonomic novelty in the chicken gut microbiome, we generated phylogenetic profiles to document known and unknown diversity and then exploited culture-dependent and culture-independent approaches to create an unprecedented high-quality reference collection of microbial genes and genomes from the chicken gut, revealing and naming hundreds of new candidate species from this commonplace but important ecological setting.

## Materials and Methods

### Sample collection and storage

Faecal samples were collected in South-East England from adult Lohmann Brown laying hens and adult Silkie hens in 2018. Birds were housed in a large outdoor run with a substrate of stone chippings and small turf enrichment beds during the day and kept in a coop overnight. They were fed a commercial layer feed, Farmgate Layer pellets and mash (ForFarmers UK Limited, Rougham, Bury St Edmunds), according to the manufacturer’s instructions and no antibiotics were used. Faecal sampling was approved by the University of Surrey’s NASPA ethics committee.

Sixty faecal samples were collected from the Lohmann Brown laying hens and thirty samples from the Silkie hens (six and three samples per day, respectively, for 10 days). Freshly evacuated faeces from individual birds were collected in sterile containers and immediately stored at −20 °C. Samples were then transferred to the laboratory for culture and/or DNA extraction. DNA was extracted using DNeasy PowerSoil kit (Qiagen, Hilden, Germany), following manufacturer’s instructions and then stored at −20 °C.

### Sequencing and subsequent workflow

**Figure 1:**
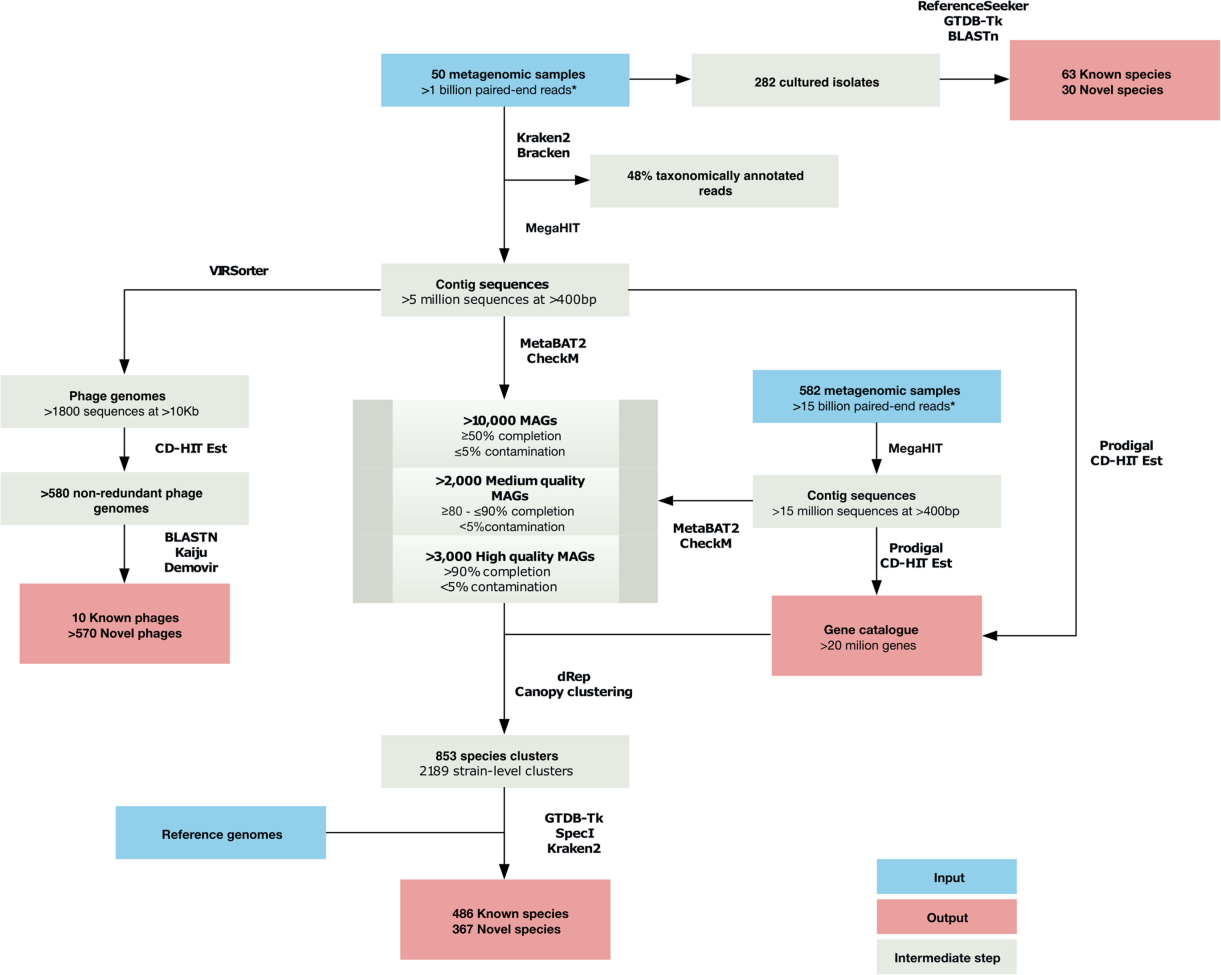
Analytical Workflow. An asterisk (*) indicates read numbers are detailed post-filtering of diet and host associated reads.

Workflow from this point forward is summarised in Fig. 1.The fifty samples yielding >20 ng DNA were processed according to the Low Input, Transpose Enabled (LITE) library construction pipeline (Perez-Sepulveda et al., 2020) before being subjected to paired-end (2 × 150 bp) metagenomic sequencing on the Illumina Novaseq 6000 platform. Bioinformatics analyses were performed on the Earlham Institute’s High Performance Computing cluster and on the Cloud Infrastructure for Microbial Bioinformatics (Connor et al., 2016). Sequences were assessed for quality using FastQC Version 0.11.8 and trimmed using Trimmomatic Version 0.36, configured to a minimum read length of 40, ‘leading’ and ‘trailing’ settings of 3 (SLIDINGWINDOW:4:20) (http://www.bioinformatics.babraham.ac.uk/projects/fastqc; Bolger, Lohse & Usadel, 2014). Metagenomic sequences for all samples have been uploaded to the Sequence Read Archive under Bioproject ID PRJNA543206.

### Reference-based metagenomic analysis

An initial analysis of our chicken faecal sequences using the Kraken 2 taxonomic classifier (Wood, Lu & Langmead, 2019) was performed on custom databases representing the domestic chicken genome (GenBank assembly accession GCF_000002315.6) and the food plants *Triticum aestivum* (wheat), *Aegilops tauschii* (diploid progenitor of the D genome of hexaploid wheat) and *Glycine max* (soy bean): GenBank assembly accessions GCF_001957025.1, GCA_900519105.1, GCF_000004515.5. Kraken 2 revealed that 8% of reads originated from the chicken and at least 16% originated from the diet. These sequences were filtered from our dataset and excluded from subsequent analyses by keeping only reads ‘Unclassified’ by Kraken 2 after comparison with each database in turn.

The remaining dataset underwent taxonomic profiling using Kraken 2 against a microbial database built from all complete/representative archaeal, bacterial, fungal, protozoan, viral and UniVec_Core sequences in RefSeq (O’Leary et al., 2016) in January 2020. Bracken (Lu et al., 2017) was used to estimate taxon abundance from the Kraken 2 profiles, accepting only those taxa with ≥1,000 assigned reads. Bracken-database files were generated using ‘bracken-build’ on our microbial database and visualised using Pavian (Breitwieser & Salzberg, 2016).

### Metagenomic assembly

We searched the NCBI BioProjects database (https://www.ncbi.nlm.nih.gov/bioproject/) in November 2019 with the term ‘chicken gut microbiome’ and then selected nine publicly available projects that contained at least one metagenomic sequence dataset >1 GByte in size: PRJEB33338, PRJNA193217, PRJNA291299, PRJNA375762, PRJNA415593, PRJNA417359, PRJEB22062, PRJNA543206, PRJNA417359, PRJNA385038, PRJNA616250. Only four of these studies were linked to research publications at the time of publication (Glendinning et al., 2020; Sergeant et al., 2014; Foster-Nyarko et al., 2020; Luiken et al., 2020)

All shotgun metagenomic reads were quality-filtered by removing reads shorter than 70% of the maximum expected read length (100 bp, 250 bp for MiSeq data), an estimated accumulated error >2.5 with a probability of ≥0.01 (Puente-Sánchez, Aguirre & Parro, 2016) or with an observed accumulated error >2, or >1 ambiguous position to assist assembly. If base quality dropped below 20 in a window of 15 bases at the 3′ end, or if the accumulated error exceeded 2, reads were trimmed. All these filter steps are integrated in sdm (Hildebrand et al., 2014). Reads mapping to the chicken genome and diet were removed from the metagenomic data as described previously, classifying reads with Kraken 2 against custom databases built on the aforementioned genomes.

Sequence datasets from our fifty samples—together with 582 samples from the selected BioProjects—were assembled using MegaHIT (Li et al., 2016) under the option ‘--k-list 25,43,67,87,101,127’. To avoid artefacts that sometimes result from co-assembly of sequences from different samples and different sources, we performed individual assemblies on each sample, with the exception of BioProject PRJNA417359. For that BioProject, as multiple metagenomic samples had been sourced from different tissues of the same individual bird, we co-assembled reads from the 120 BioSamples from that project.

### Bacteriophage identification and characterisation

Contig sequences from the MegaHIT assemblies of our 50 samples that were ≥10 kb were analysed with VirSorter v1.0.5 with the ‘-db 2’ option to identify viral genomes (Roux et al., 2015). VirSorter Category 1 and 2 contig sequences were collapsed at 95% nucleotide identity over 70% of the sequence length using CD-Hit Est v4.6.1 (Fu et al., 2012). Classification of bacteriophage sequences relied on nucleotide searches using BLASTN against the NCBI NT database (Completed April 2020) and protein searches using Kaiju Version 1.7.3 against the RefSeq database (Completed April 2020) (Menzel, Ng & Krogh, 2016). Only bacteriophage genomes with BLASTN hit *E*-Value < 0.05, percentage identity >70% and query covering >50% were selected as reliable hits.

A taxonomic assignment was drawn from the highest scoring BLASTN (or in rare cases BLASTP) hit ranked by query cover and percentage ID. Synteny between predicted coliphages and their respective reference genomes were visualised using EasyFig (Sullivan, Petty & Beatson, 2011). *Escherichia* bacteriophage coverage per sample was determined using Anvi’o v6.1 (Eren et al., 2015) using default parameters and visualised in R using the Pheatmap package (https://www.rdocumentation.org/packages/pheatmap). Remaining viral genomes were filtered for completeness, retaining those that were circular and encoded a complete terminase gene (as predicted by VirSorter). Taxonomic assignments to the level of family were performed on viral genomes using Demovir (https://github.com/feargalr/Demovir).

### Gene catalogue

Complete genes identified by Prodigal v2.6.1 (Hyatt et al., 2010) were clustered at 95% nucleotide identity using CD-HIT-Est v4.6.1 (Fu et al., 2012). Incomplete genes were then mapped to this complete gene list using Bowtie2 v 2.3.4.1 (Langmead & Salzberg, 2012) and any mapping at 95% nucleotide identity were incorporated into the relevant gene clusters. Finally, genes representing the 40 conserved marker genes defined by Mende et al. (2013) were clustered separately and then merged with the existing set of gene clusters. We thus obtained a gene catalogue of >20 million genes, defined as non-redundant at 95% average nucleotide identity (ANI). The final gene catalogue was uploaded to FigShare (https://doi.org/10.6084/m9.figshare.13116809.v4)

### Abundance estimates of contigs and genes

Prodigal (Hyatt et al., 2010) was applied in metagenome-mode to all contigs from the MegaHIT assemblies. Unfiltered reads from each sample were mapped against their respective assembly to provide an estimate of contig and gene abundance using Bowtie2 (Langmead & Salzberg, 2012) with the options ‘--no-unal--end-to-end -score-min L, −0.6,−0.6’. Samtools 1.3.1 was used to sort and index all resulting Bam files (Li et al., 2009). Only reads with mapping quality >20, >95% nucleotide identity and >75% overall alignment length were retained. BEDTools v2.21.0 (Quinlan, 2014) was used to create depth profiles from the Bam files. These depth profiles were then translated with rdCover (https://github.com/hildebra/rdCover) into average coverage (in a 50 bp window) per contig or per gene predicted from each contig. Bam files were translated to abundances using the ‘jgi_summarize_bam_contig_depths’ script from the MetaBAT 2 package (Kang et al., 2019).

Gene abundances were linked to their respective gene clusters and originating samples. Redundant genes representing the same orthologue were removed.

### Binning

We identified metagenomic species (MGSs) using the combinatorial approach described by Hildebrand et al. (2019), incorporating single-assembly binning in the creation of metagenome-assembled genomes (MAGs), gene catalogue binning in the creation of canopy clusters (Nielsen et al., 2014) and hierarchical clustering of candidate genes using the R function hclust, method = complete. To start with, we used MetaBAT 2 v2.15 (Kang et al., 2019) to bin contigs ≥400 bp. These were quality filtered using CheckM v1.0.11 (Parks et al., 2015) to obtain 5,595 bins at >80% completeness and <5% contamination.

Species-level clusters were formed using a combination of two distinct approaches. One approach removed redundancy between samples by pre-clustering bins if ≥30% of their genes overlapped with a higher-quality bin to create a set of pre-MGS bins. Lower-quality bins (>60% completeness and <10% contamination) were also included in the analysis but were not used to form new species clusters. To recover prokaryotic species usually obscured using single-sample assemblies and conventional binning techniques, we refined all species bins into ‘hcl-clusters’ using gene correlations and hierarchical clustering, as described by Hildebrand et al. (2019). We chose genes occurring in ≥10% of all associated MAGs as representatives for each pre-MGS bin and used these to fish for additional co-occurring genes from the gene catalogue, using a threshold of >0.75 Pearson correlation and >0.85 spearman rho to identify gene co-occurrences within this core gene set. We then merged MetaBAT 2 bins, canopy bins and co-occurring genes into our species bins. We used the presence of 40 known single-copy marker genes, without duplicates, as a quality criterion in selection of sub-clusters, before extracting the final set of MGS gene representatives using MATAFILER (https://github.com/hildebra/MATAFILER). The final collection of MGS bins (canopy clusters + hcl-clusters) was re-assessed for contamination and completeness using CheckM (Parks et al., 2015), so that we could be confident that each bin represents a single species.

A second approach de-replicated all MAGs at 95% ANI (species-level) and 99% ANI (strain-level) using dRep Version 2.0 (Olm et al., 2017) and only species not identified in approach one were added to the resulting non-redundant species catalogue. The minimum aligned fraction used during ANI genome alignment was 60%. A single representative MAG for each novel species cluster was uploaded to NCBI SRA under BioProject PRJNA543206 and all MAGs generated were uploaded to FigShare (https://doi.org/10.6084/m9.figshare.13116809.v4.). CompareM Version0.1.1 (https://github.com/dparks1134/CompareM) was used to calculate average amino acid identity (AAI) when identifying novel genera, using a cut-off of 60% for the percentage identity and 70% for the minimum alignment length used to delineate genus boundaries.

### Taxonomy of metagenomic species

We used the Genome Taxonomy Database Toolkit (GTDB-Tk Release 95) to perform taxonomic assignments on strain-level dereplicated MAGs (Chaumeil et al., 2019). In addition, genes from each MGS were analysed through GTDB-Tk (Release 95), proGenomes resource (Mende et al., 2017) and underwent k-mer-based taxonomic profiling using Kraken 2. In assigning taxonomy, we allowed GTDB assignments to take precedence—only when no GTDB taxonomy was available would we adopt taxonomies assigned by ProGenomes and Kraken 2 and, then, only where genus and family assignments from these sources matched. When exploiting the taxonomy assigned according genes from metagenomic species, we applied a least-common-ancestor approach to unplaced taxa at higher taxonomic levels. Species distribution analyses were conducted using the Vegan package in R (R-Core-Team, 2018), before visualisation using ggplot2 (Wickham, 2016) and Pheatmap R packages (https://www.rdocumentation.org/packages/pheatmap). Pan-genome analysis was conducted using Roary v3.11.2 and visualised using the roary2svg.pl script (Page et al., 2015). Comparison of our derived metagenomes with those of Glendinning et al. (2020) was performed at 95% ANI using dRep and visualised using web-tool BioVenn (Hulsen, De Vlieg & Alkema, 2008).

### Bacterial culture

To estimate species richness and diversity, the Phyloseq package of R (R-Core-Team, 2018) was applied to the output from Bracken (Lu et al., 2017) on all of our chicken faecal metagenomic datasets. The six faecal samples that showed highest species richness and taxonomic diversity were selected for culture-based studies. Frozen faecal samples were thawed, vortexed and two 0.5 g aliquots (once processed aerobically, the other anaerobically) from each sample were suspended in 5 ml PBS. Each aliquot was vortexed until homogenised, before performing serial dilutions in duplicate down to 1 × 10^−5^. Processing of samples for aerobic and anaerobic culture was identical, except that, for anaerobic culture, all culture media, diluent and consumables were pre-reduced to anaerobic conditions for at least 24 h before faecal samples were processed in a Whitley A95TG workstation.

For dilutions 10^−3^–10^5^, 200 µl was plated directly on to a set of three agar plates for each culture medium (Brain Heart Infusion, Colombia Blood Agar, Yeast extract, casitone and fatty acid) with or without vancomycin supplementation at a concentration of 6 µg/ml (Table S1). Cultures were incubated at 37 °C for 72 h in their respective conditions before assessment of colony growth. Well-isolated colonies were picked according to colonial morphotype distinctive in colour, shape and size, before being re-streaked on to the growth medium from which they were sourced to confirm purity. Individual colonies were subsequently used to inoculate 2 ml of broth based on the source culture medium, incubated at 37 °C for a further 24 h before bacterial DNA extraction. All isolates were archived at −80 °C in glycerol at 20% concentration.

### Genome sequencing and analysis

Genomic DNA was extracted using a DNeasy UltraClean DNA isolation kit according to the manufacturer’s instructions (Qiagen, Hilden, Germany). DNA was quantified using a Qubit^®^ fluorometer (Invitrogen, Carlsbad, CA, USA) high-sensitivity assay, before dilution to the required concentration in RNase-free water and purification on AMPure XP beads (Beckman Coulter, Brea, CA, USA). Sequencing libraries were prepared from 0.5 ng/µl of RNA free genomic DNA. A total of 282 isolates were included for genomic sequencing using the Nextera-XT DNA sample preparation kit (Illumina, San Diego, CA, USA) and whole-genome sequencing performed using the Illumina NextSeq sequencing platform, generating paired-end reads (2 × 150 bp).

Paired-end reads were quality-assessed and trimmed using FastQC and Trimmomatic as described above. Trimmed reads were assembled into contigs using SPAdes version 3.13.1 (Bankevich et al., 2012). Contigs shorter than 500 bp were discarded from analysis. Genome contamination and completeness was assessed using CheckM version 1.0.13. To confirm assembly quality, only genomes conforming to all the following criteria were included in further analysis: (i) contig N50 of >20 kbp (ii) 90% of assembled bases at >5× read coverage (iii) completeness of >95% (iv) contamination of <5% (v) complete 16S rRNA gene sequence.

### Genome sequence taxonomic assignment

Barrnap Version 0.9 (https://github.com/tseemann/barrnap) was applied to all genomes that passed the quality filters to extract full-length 16S rRNA gene sequences. These were then compared to NCBI 16S rRNA gene sequences from RefSeq genomes using the NCBI’s web-based BLASTN facility (Altschul et al., 1990). 16S rRNA gene sequences that showed an identity of <98.7% to known sequences were assigned to novel species, using the conservative approach in proposed minimal standards (Chun et al., 2018). We used ReferenceSeeker Version 1.6.2 (Schwengers et al., 2019) to determine ANI and conserved DNA values compared to RefSeq bacterial genomes (Completed March 2020) (O’Leary et al., 2016). Genomes that showed ANI ≤95% and conserved DNA ≤69% to the closest relative were designated novel species. The Genome Taxonomy Database Toolkit (GTDB-Tk Release 95) was used to perform taxonomic assignments on isolate genomes (Chaumeil et al., 2019). Genomes were clustered at 95% and 99% ANI before selection of a single representative isolate per species using dRep (Olm et al., 2017). Where a genome previously designated as novel clustered with a genome of assigned taxonomy, this taxonomy was then applied to the previously designated ‘novel’ genome. Final taxonomic assignments were based on genome-based ANI values derived from RefSeq and GTDB—with GTDB assignments taking precedence. A single representative genome for each novel or renamed species cluster was uploaded to NCBI SRA under BioProject PRJNA543206 and all genomes alongside respective 16S rRNA gene sequences generated were uploaded to FigShare (https://doi.org/10.6084/m9.figshare.13234556).

### Phylogenetic analysis

For phylogenetic analysis of all MGS and genome sequenced isolates we used PhyloPhlAn v3.0.58 (Asnicar et al., 2020) with the ‘diversity high’ and a proteome input predicted from all genome sequences using Prodigal v2.6.1 (Hyatt et al., 2010). Diamond v0.9.34 (Buchfink, Xie & Huson, 2015) was used to perform a search against 400 universal PhyloPhlAn markers. MAFFT v.7.271 (Katoh et al., 2002) was used to perform multiple sequence alignment before refinement with trimAl v.1.4 (Capella-Gutiérrez, Silla-Martínez & Gabaldón, 2009) and reconstruction into trees using FastTree v2.1 and RAxML v. 8.2.12 (Price, Dehal & Arkin, 2010; Stamatakis, 2014). All trees were visualised and annotated manually using the online iTOLv5.7 platform (Letunic & Bork, 2016). Trees were scrutinised to confirm that species and genera were monophyletic. Phylogeny for all cultured genomes unassigned at species level was confirmed as previously described against all available reference proteomes of that respective genus downloaded from NCBI.

To investigate the phylogenetic placement of cultured isolates designated as *Escherichia marmotae* and *Escherichia* sp001660175 by GTDB, we constructed a core genome phylogenetic tree. The genomes from cultured isolates were compared to genomes representing the full diversity of the genus *Escherichia*. Three *Salmonella* genomes were included as an outgroup. The genome sequences were aligned using Mugsy (Angiuoli & Salzberg, 2011), and alignment blocks conserved across all genomes were concatenated to produce a core genome alignment. A phylogenetic tree was constructed by maximum likelihood with 100 rapid bootstrap replicates, using the general time reversible model of nucleotide substitution with gamma correction for rate heterogeneity, as implemented in RAxML version 8.2.12 (Stamatakis, 2014).

## Results

### Reference-based profiling documents novel diversity

We collected faecal samples from 90 chickens reared in the UK belonging to two breeds: Lohman Browns (*n* = 60) and Silkies (*n* = 30). Short-read sequencing of 50 of these faecal samples generated a metagenomic dataset in excess of a billion paired-end reads or three hundred billion base pairs (Table S2).

We initially analysed the faecal samples using the k-mer-based programme Kraken 2, followed by refined phylogenetic analysis using the allied programme Bracken (Lu et al., 2017) (Table S3). Unsurprisingly, these programmes assigned sequence reads from the faecal samples to all three domains of life, as well as to viruses (Table S4), although relative abundance assignments show that bacteria predominate in this environment. Sequences were assigned to a wide range of bacterial phyla, including the three expected as predominant in the vertebrate gut (Bacteroidetes, Firmicutes, Proteobacteria), but also including over twenty additional phyla. Searches of the PubMed database with each phylum name and the term ‘chicken’ reveal that round half of these have been previously documented in the chicken gut. However, at least a dozen appear to be novel in this setting, including the *Aquificae, Balneolaeota, Calditrichaeota, Chlorobi, Dictyoglomi, Fibrobacteres, Gemmatimonadetes, Ignavibacteriae, Kiritimatiellaeota, Lentisphaerae, Nitrospirae*, and the *Thermodesulfobacteria*.

When we rank-ordered the species identified by Bracken according to maximum abundance in any one sample, we found, as expected, that species from the family *Lactobacillaceae* dominated among the top 20 most abundant organisms. However, we found that two species of *Escherichia— Escherichia coli* and *Escherichia marmotae—*accounted for ≥5% of reads in nearly half of the samples (22/50) and in two samples, accounted for more than 50%. Such monodominance of the gut microbiome by bacterial species has been described in diseased humans (Hildebrand et al., 2019; Ravi et al., 2019), but is surprising in the context of poultry reported as apparently healthy by their handlers. We also noted a high relative abundance of the recently described chicken pathogen *Gallibacterium anatis* (Narasinakuppe Krishnegowda et al., 2020) in most birds (with four birds showing >5% reads assigned to this organism), despite their healthy status. Similarly, *Fusobacterium mortiferum*—an opportunistic pathogen of humans (Almohaya et al., 2020)—accounted for >10% of sequences in 11 birds, corroborating a recent report of high abundance of 16S rRNA gene sequences from this organism obtained from the chicken caecum (Kollarcikova et al., 2019).

Bracken assigned sequences to over a hundred bacteriophage genomes, predominately phages infecting members of the *Enterobacteriaceae* assigned to the families *Myoviridae* and *Podoviridae*. Particularly noteworthy was the high abundance of reads in some samples from two distinct bacteriophages that prey on *E. coli*: phiEcoM-GJ1—a lytic bacteriophage isolated in Canada from pig sewage (Jamalludeen et al., 2008)—which accounted for 6.5% reads in a single sample and phAPEC8—a lytic bacteriophage with a large 147 kb genome, isolated from a Belgian poultry farm—which accounted for 10% of reads in a single sample and for >1% of reads in three others (Tsonos et al., 2012).

Although these k-mer-based analyses can provide interesting insights into taxonomic diversity within the chicken gut, we quickly realised that they provide an incomplete and misleading picture of this important microbiome for several reasons: (1) they often report the presence of highly implausible organisms—for example, Kraken 2 reported the presence of human pathogens such as *Shigella flexneri* and *Plasmodium falciparum* that are simply not credible in this context on clinical grounds; (2) as with studies on 16S rRNA gene sequences, they fail to provide genomic data or insights into the functional diversity or population structure of the microbial species that they identify and; (3) they rely on a reference database and so can only report previously known organisms and can never uncover ‘unknown unknowns’.

The scale of the problem of unknown diversity is clear from the observation that nearly three quarters (73%) of sequence reads from our chicken samples cannot be confidently classified by Kraken 2 to species level and more than half of the reads (52%) cannot be classified at all and are simply designated as ‘Unassigned’. We therefore sought to extend our understanding of this community through two powerful reference-free approaches: assembly-based metagenome analyses and high-throughput culture.

### Metagenomic assembly uncovers a wealth of viral diversity

Assembly of metagenomic sequences is a reference-free approach that involves aligning and merging short sequence reads into long contiguous sequences (contigs) contigs.

To confirm the presence of bacteriophages inferred through the reference-based analysis and to identify novel viral genomes, we assembled sequence reads from our fifty chicken faecal samples into contigs. Contigs ≥10 kb were analysed with VirSorter—a programme designed to detect viral signals in microbial sequence data to find novel viruses (Roux et al., 2015).

**Figure 2:**
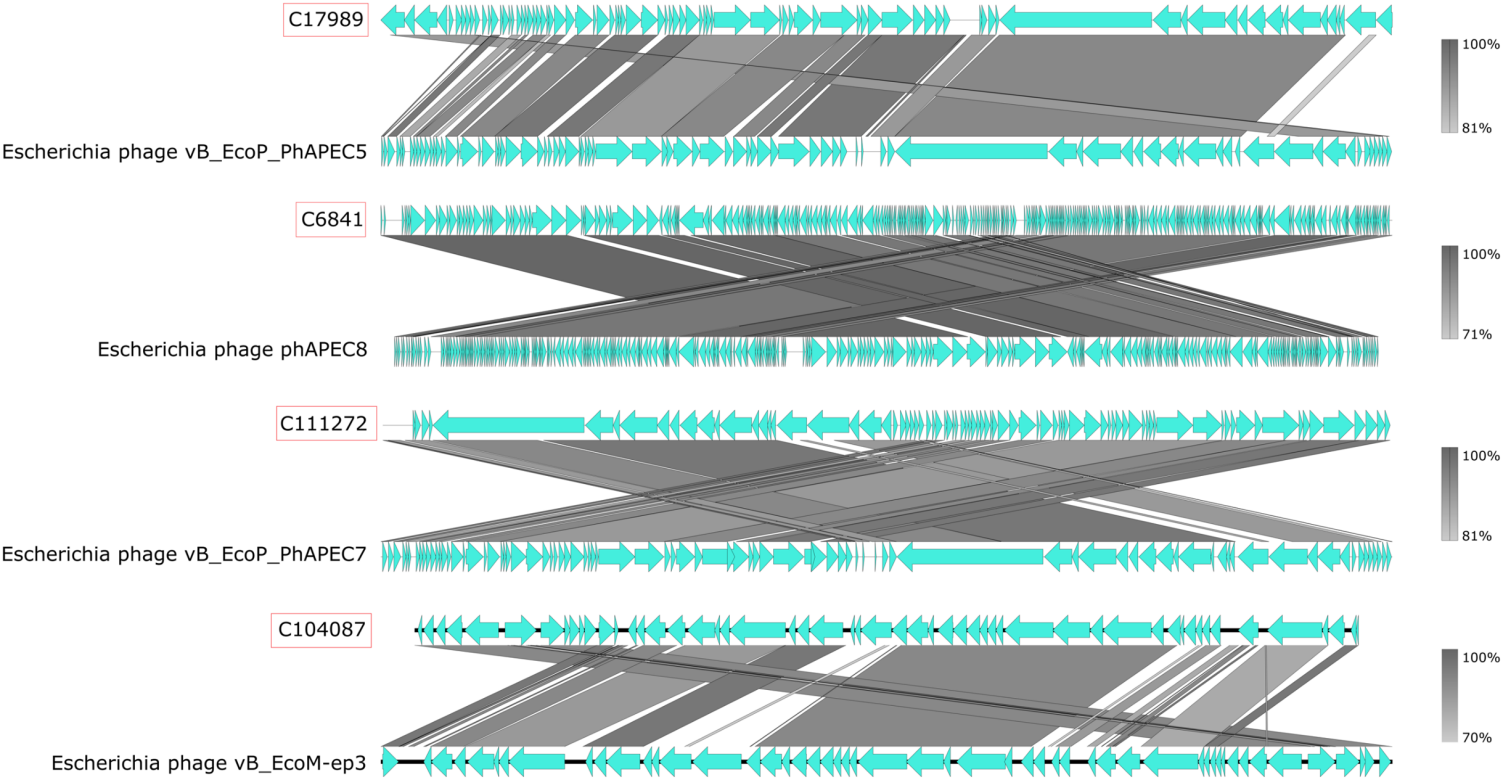
Genome synteny of recovered phage genomes. Synteny plots comparing four novel coliphage genomes recovered from chicken faecal metagenomes (in red) to closest reference genomes. The figure was generated using EasyFig.

**Figure 3:**
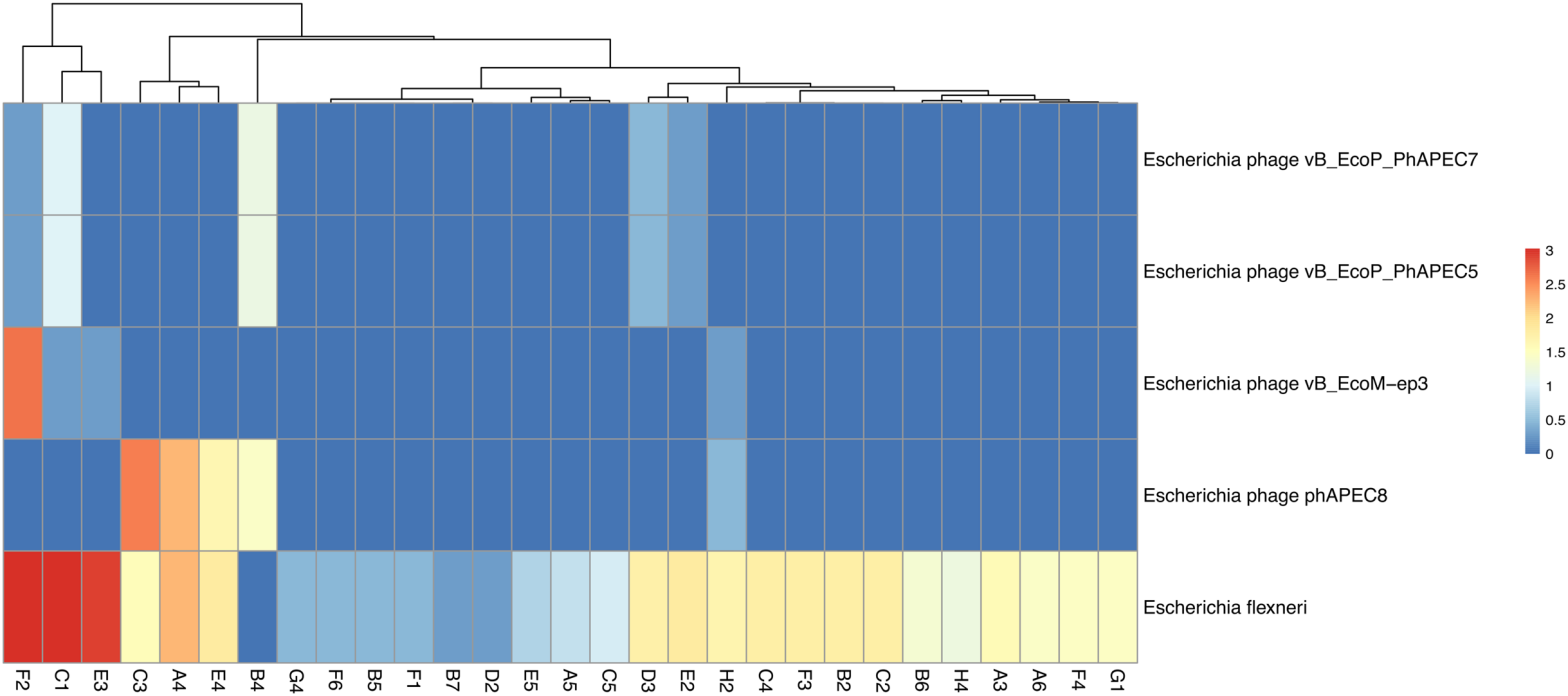
Coliphage abundance within chicken faecal samples. Coverage of four coliphages and of putative host bacterial species. Only samples in which at least one genome had ≥1× coverage are shown (*n* = 29). All coverage values have been Log_10_ transformed with blue depicting low abundance and red high abundance.

VirSorter identified 184 of our chicken faecal contigs as Category 1 (‘most confident’) bacteriophage sequences and identified an additional 1,840 contigs as Category 2 (‘likely’) bacteriophage sequences. This was de-replicated to 1,455 genomes using similarity thresholds of 95% ANI over 70% of the genome (Table S5). BLASTN analysis revealed only 10 of these bacteriophage genomes showed high similarity (percentage identity > 70%; query covering > 50%) to known phages at the nucleotide level (Table S6). These included close relatives of the two phages (phiEcoM-GJ1 and phAPEC8) found highly abundant in the Bracken analyses (Fig. 2). Interestingly, more than one genus of coliphage (e.g. *Jilinvirus, Phapecoctavirus*, or *Gamaleyavirus*) was often detected in the same sample, along with an abundance of reads from their predicted prey (*Escherichia*) suggesting interesting dynamics in phage-host and phage-phage interactions (Fig. 3; Table S7).

Of the remaining 1,445 unclassified bacteriophage genomes, nearly 600 encoded either an obvious terminase region or were circular and as such were suggested as being near-complete. Classification of these genomes revealed all genomes were predicted to belong to the order *Caudovirales* of tailed phages, with the majority belonging to the family *Siphoviridae* (*n* = 429), but we also found representatives from the *Myoviridae* (*n* = 87) and *Podoviridae* (*n* = 27), plus some bacteriophages unclassified at family level (*n* = 28) (Table S8).

### Remarkable microbial genome diversity in the chicken gut

Next, we subjected our samples to computational binning—a process of grouping contigs on the basis of sequence composition and depth of coverage into discrete population bins representing metagenome-assembled genomes (MAGs). However, to carry out a definitive survey of bacterial and archaeal diversity in the chicken gut microbiome—in addition to analysing the fifty faecal samples mentioned and before we started the binning—we retrieved all publicly available chicken gut metagenomic datasets, to create an expansive dataset representing >630 samples, drawn from ten studies and twelve countries (Belgium, China, France, Germany, Italy, Malaysia, Netherlands, Poland, Spain, The Gambia, UK, USA) (Figs. S1A and S1B; Table S9).

Sequence assembly and binning on all these samples generated 5,595 MAGs that passed our quality threshold of ≥80% completion and ≤5% contamination (Fig. S1C). Of these 3,131 could be considered high-quality draft genomes, with >90% completion and <5% contamination, as judged by recently published criteria (Table S10) (Bowers et al., 2017). Genome sizes of the MAGs ranged from ~0.4 to 6.8 Mbp, while GC content ranged from 24% to 73%.

Then, we grouped the MAGs into metagenomic species (MGSs). Initially, this involved de-replicating MAGs at the widely accepted 95% average nucleotide identity (ANI) for defining bacterial and archaeal species and 99% ANI for defining bacterial and archaeal strains (Jain et al., 2018; Luo, Rodriguez-R & Konstantinidis, 2014). De-replication of MAGs at 95% ANI resulted in 846 clusters representing bacterial and archaeal species, while de-replication at 99% ANI resulted in 2182 clusters, representing strains. However, to improve recovery of MAGs, MGSs and associated gene sets, we used gene correlations to identify species-representative genes and then applied hierarchical clustering to co-occurring genes across the samples. This allowed us to identify additional genes from the core genome of a species, even when they show divergent nucleotide compositions (such as genes from genomic islands and plasmids) (Hildebrand et al., 2019). Similarly, using canopy clustering (Nielsen et al., 2014), we could identify commonly occurring species of low abundance. Using these approaches, we were able to identify an additional seven MGSs (Table S11). These MGS were prevalent at >1× coverage in 53% of all analysed samples spanning at least 4 different BioProjects.

**Figure 4:**
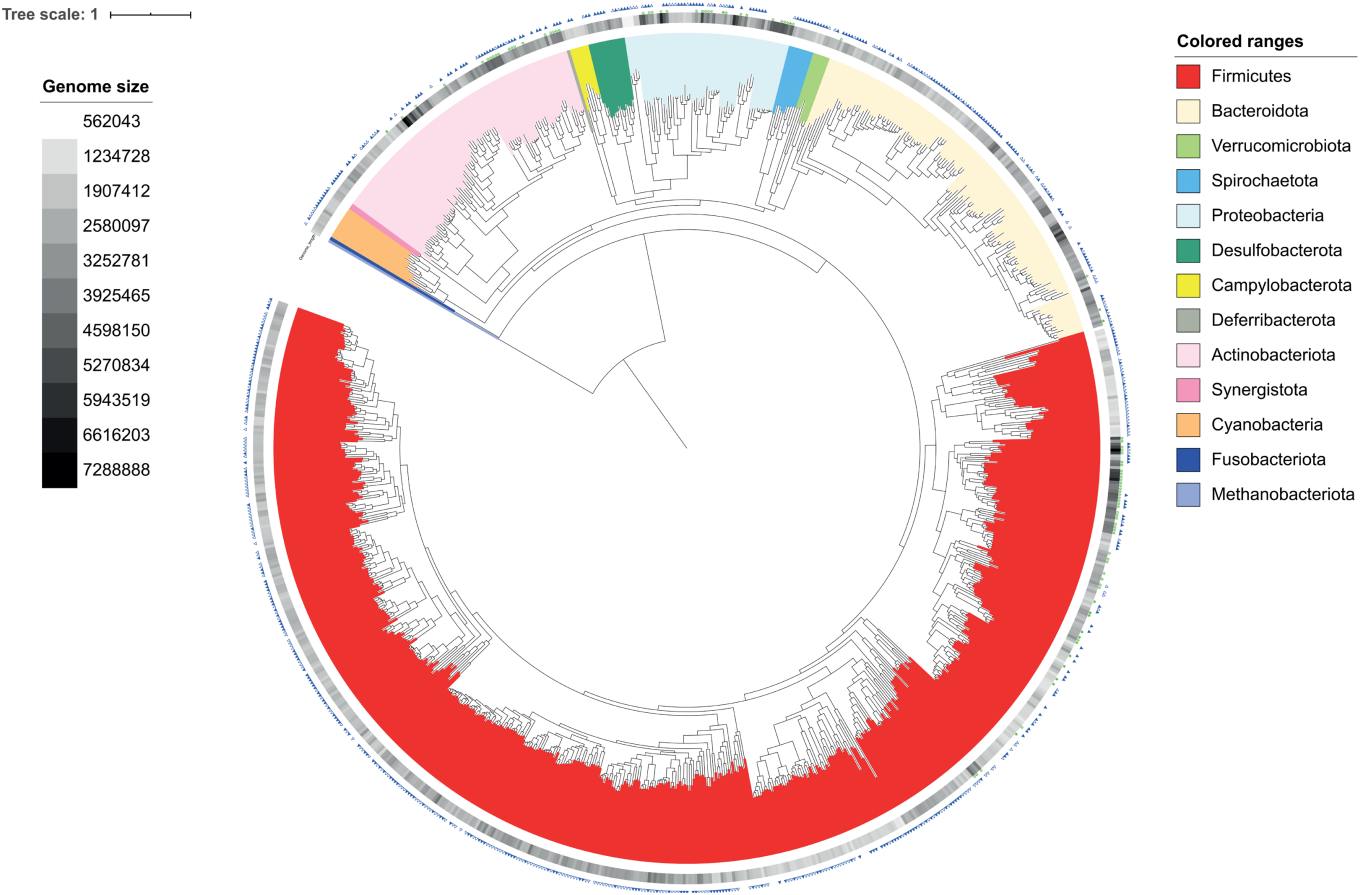
Phylogenetic tree of draft MGS genomes derived from 820 metagenomic samples of the chicken gut and draft genomes of 93 species cultured from chicken faecal samples. Phylum, generally as assigned by GTDB, is indicated by colour range. Data symbols in the outer layers have been used to describe further characteristics for each draft genomes. Triangles indicate sequence novelty and status of binomial designation within publicly available databases or published research with filled symbols indicating novel species assigned a binomial as part of this research, hollow symbol indicated a known species assigned a binomial as part of this research and no symbol indicated a known species with a well-formed binomial already assigned. Stars are used to indicate isolation source, with filled symbols indicating isolation of species in both culture and metagenomic assembly and hollow symbols indicating isolation in culture alone. Tree branches have been collapsed where duplicate species have been identified by different methodologies. The tree was reconstructed using PhyloPhlAn 3.0.58 against 400 marker genes before reconstruction using FastTree and RAxML of a MAFFT sequence alignment and visualised using the online iTOLv5.7 tool including provision of a heat map according to individual genome length.

Analysis of bacterial metagenomic species, primarily using the Genome Taxonomy Database (GTDB) taxonomy (Parks et al., 2020), confirmed and extended the taxonomic novelty uncovered by reference-based community profiling (Fig. 4), recovering species spanning nineteen of the bacterial phyla defined by GTDB (Table S12). These include *Cyanobacteria* (12 species; 32 strains); *Deferribacterota* (1 species; 1 strain) *Synergistota* (2 species; 5 strains) and the *Verrucomicrobiota* (7 species; 8 strains).

Of the 853 de-replicated bacterial metagenomic species, 321 represented previously delineated species catalogued in publicly available databases (Table S13). Following direct comparison, a further 165 metagenomic species had been previously identified by Glendinning et al. (2020), with these sequences not currently available in public archives. However, only 158 of our metagenomic species possess validly published names based on Latin binomials.

We performed a search of PubMed with the species name and ‘chicken’, leaving aside the 33 species named by Glendinning et al. (2020). This suggested that our study provides the first-evidence-in-chickens for the majority (81/125) of these species (Table S14). Examples include: *Jeotgalicoccus halophilus*, first isolated from the traditional fermented seafood, Jeotgal (Yoon et al., 2003) and present in 197 chicken samples; *Aliicoccus persicus*, first isolated from a hypersaline lake (Amoozegar et al., 2014) and present in 241 chicken samples; and *Bacteroides reticulotermitis*, first isolated from the gut of a termite (Sakamoto & Ohkuma, 2013) and present in 39 chicken samples.

We found that 309 of our metagenomic species could be assigned a taxonomy only at the level of genus and so represent novel candidate species. A further 56 species could be assigned a taxonomy only at the level of family and, after AAI clustering at 60%, were assigned to 36 novel candidate genera. One candidate bacterial species could be assigned a taxonomy only at the level of order (*Oscillospirales*) and so represent a new family.

Three MAGs were assigned to the domain Archaea. One represents the species *Methanobrevibacter woesei*—which is already known to inhabit the chicken gut (Saengkerdsub et al., 2007)—while the other two represent novel species within the genera *Methanocorpusculum* and UBA71, which we have renamed *Candidatus* Methanospyradousia.

### Linnaean binomials for hundreds of new candidate species

Linnaeus first proposed the assignment of Latin binomials to provide a universal nomenclature for biological species (Linnaeus, 1759). The International Code of Nomenclature of Prokaryotes (ICNP) sets the rules for naming prokaryotic species (Parker, Tindall & Garrity, 2019), but currently precludes the valid publication of names of uncultivated organisms, represented by MAGs or other sequences. Furthermore, high-throughput generation of MAGs and of sequence-based taxonomies for bacteria, such as the GTDB (Parks et al., 2020) is often assumed to preclude the detailed attention usually given to one-by-one construction of Linnaean binomials. As a result, most uncultured taxa, as well as many taxa defined on sequence-based criteria, have been assigned unstable, confusing and hard to-remember alphanumerical identifiers.

To provide a stable, clear and memorable nomenclature for novel and/or previously unnamed bacterial and archaeal species from the chicken gut, we exploited the provision within the ICNP for naming uncultivated taxa via *Candidatus* assignments, which, although provisional, provide the scientific community with well-formed Latin binomials (Oren, 2017; Oren et al., 2020). However, this prompted us into an unprecedented effort to create hundreds of new names for the purpose of this single research study—an effort that required us to devise a scalable combinatorial system for the creation of binomials. Here, we made extensive combinatorial use of several dozen Latin and Greek roots pertaining to poultry (*avi-, galli-, pulli-, alektryo, ptero, kotto-, ornitho-*), intestines (*intestini- entero-*), faeces (*faec-, kakke, merd-, kopro-, excrement-*) or microbial life *(-monas, -bacterium, -microbium, -coccus, -bacillus, -bium, -cola*)—twinned with addition of these roots (singly or in tandem) and/or prefixes (*allo, hetero, meta-, para-, crypto-*) to existing genus names—to create over 150 *Candidatus* genus names. For genera with alphanumeric designations in GTDB Release 05-RS95 (Parks et al., 2020) known to occur also in gut microbiomes of other animals, we adopted a similar combinatorial approach, but avoided roots pertaining to poultry and stuck instead with combinations that simply meant ‘gut or faecal microbe’, for example *Fimicola, Caccocola*. An additional source of diversity stemmed from repetitive use of around forty *Candidatus* species epithets built from similar roots, which when combined with genus names gave us a total of over 650 distinctive binomials for new *Candidatus* species (Table 1; Table S15).

**Table 1:** Protologues for new *Candidatus* taxa identified from metagenomic analysis of chicken gut samples.

**Description of *Candidatus* Acetatifactor stercoripullorum sp. nov.**
*Candidatus* Acetatifactor stercoripullorum (ster.co.ri.pul.lo’rum. L. neut. n. *stercus* dung; L. masc. n. *pullus* a young chicken; N.L. gen. n. *stercoripullorum* of the faceces of young chickens)
A bacterial species identified by metagenomic analyses. This species includes all bacteria with genomes that show ≥95% average nucleotide identity (ANI) to the type genome, which has been assigned the MAG ID CHK195-6426 and which is available via NCBI BioSample SAMN15816622. The GC content of the type genome is 48.46% and the genome length is 3.1 Mbp.
**Description of *Candidatus* Acinetobacter avistercoris sp. nov.**
*Candidatus* Acinetobacter avistercoris (a.vi.ster’co.ris. L. fem. n. *avis* bird; L. neut. n. *stercus* dung; N.L. gen. n. *avistercoris* of bird faeces)
A bacterial species identified by metagenomic analyses. This species includes all bacteria with genomes that show ≥95% average nucleotide identity (ANI) to the type genome, which has been assigned the MAG ID 5402 and which is available via NCBI BioSample SAMN15816735. The GC content of the type genome is 38.29% and the genome length is 3.9 Mbp.
**Description of *Candidatus* Acutalibacter ornithocaccae sp. nov.**
*Candidatus* Acutalibacter ornithocaccae (or.ni.tho.cac’cae. Gr. masc. or fem. n. *ornis, ornithos* bird; Gr. fem. n. *kakke* faeces; N.L. gen. n. *ornithocaccae* of bird faeces)
A bacterial species identified by metagenomic analyses. This species includes all bacteria with genomes that show ≥95% average nucleotide identity (ANI) to the type genome, which has been assigned the MAG ID ChiBcolR8-3208 and which is available via NCBI BioSample SAMN15816822. This is a new name for the alphanumeric GTDB species sp000435395. The GC content of the type genome is 62.02% and the genome length is 2.1 Mbp.
**Description of *Candidatus* Acutalibacter pullicola sp. nov.**
*Candidatus* Acutalibacter pullicola (pul.li’co.la. L. masc. n. *pullus* a young chicken; L. suff. *-cola* inhabitant of; N.L. n. *pullicola* an inhabitant of young chickens)
A bacterial species identified by metagenomic analyses. This species includes all bacteria with genomes that show ≥95% average nucleotide identity (ANI) to the type genome, which has been assigned the MAG ID CHK185-1770 and which is available via NCBI BioSample SAMN15816590. The GC content of the type genome is 58.43% and the genome length is 2.1 Mbp.
**Description of *Candidatus* Acutalibacter pullistercoris sp. nov.**
*Candidatus* Acutalibacter pullistercoris (pul.li.ster’co.ris. L. masc. n. *pullus* a young chicken; L. neut. n. *stercus* dung; N.L. gen. n. *pullistercoris* of young chicken faeces)
A bacterial species identified by metagenomic analyses. This species includes all bacteria with genomes that show ≥95% average nucleotide identity (ANI) to the type genome, which has been assigned the MAG ID 1282 and which is available via NCBI BioSample SAMN15816718. The GC content of the type genome is 63.65% and the genome length is 2.0 Mbp.
**Description of *Candidatus* Acutalibacter stercoravium sp. nov.**
*Candidatus* Acutalibacter stercoravium (ster.cor.a’vi.um. L. neut. n. *stercus* dung; L. fem. n. *avis* bird; N.L. gen. n. *stercoravium* of bird faeces)
A bacterial species identified by metagenomic analyses. This species includes all bacteria with genomes that show ≥95% average nucleotide identity (ANI) to the type genome, which has been assigned the MAG ID ChiBcolR1-495 and which is available via NCBI BioSample SAMN15816868. This is a new name for the alphanumeric GTDB species sp900543555. The GC content of the type genome is 60.31% and the genome length is 2.0 Mbp.
**Description of *Candidatus* Acutalibacter stercorigallinarum sp. nov.**
*Candidatus* Acutalibacter stercorigallinarum (ster.co.ri.gal.li.na’rum. L. neut. n. *stercus* dung; L. fem. n. *gallina* hen; N.L. gen. n. *stercorigallinarum* of hen faeces)
A bacterial species identified by metagenomic analyses. This species includes all bacteria with genomes that show ≥95% average nucleotide identity (ANI) to the type genome, which has been assigned the MAG ID ChiGjej2B2-2649 and which is available via NCBI BioSample SAMN15816629. The GC content of the type genome is 63.77% and the genome length is 2.1 Mbp.
**Description of *Candidatus* Agathobaculum intestinigallinarum sp. nov.**
*Candidatus* Agathobaculum intestinigallinarum (in.tes.ti.ni.gal.li.na’rum. L. neut. n. *intestinum* gut; L. fem. n. *gallina* hen; N.L. gen. n. *intestinigallinarum* of the gut of the hens)
A bacterial species identified by metagenomic analyses. This species includes all bacteria with genomes that show ≥95% average nucleotide identity (ANI) to the type genome, which has been assigned the MAG ID ChiGjej6B6-20540 and which is available via NCBI BioSample SAMN15816816. This is a new name for the alphanumeric GTDB species sp900555465. The GC content of the type genome is 60.79% and the genome length is 2.0 Mbp.

**Table:** 

**Description of *Candidatus* Agathobaculum intestinipullorum sp. nov.**
*Candidatus* Agathobaculum intestinipullorum (in.tes.ti.ni.pul.lo’rum. L. neut. n. *intestinum* gut; L. masc. n. *pullus* a young chicken; N.L. gen. n. *intestinipullorum* of the gut of young chickens)
A bacterial species identified by metagenomic analyses. This species includes all bacteria with genomes that show ≥95% average nucleotide identity (ANI) to the type genome, which has been assigned the MAG ID ChiBcec16-9926 and which is available via NCBI BioSample SAMN15816670. The GC content of the type genome is 57.76% and the genome length is 1.9 Mbp.
**Description of *Candidatus* Agathobaculum merdavium sp. nov.**
*Candidatus* Agathobaculum merdavium (merd.a’vi.um. L. fem. n. *merda* faeces; L. fem. n. *avis* bird; N.L. gen. n. *merdavium* of bird faeces)
A bacterial species identified by metagenomic analyses. This species includes all bacteria with genomes that show ≥95% average nucleotide identity (ANI) to the type genome, which has been assigned the MAG ID ChiBcec15-6302 and which is available via NCBI BioSample SAMN15816712. The GC content of the type genome is 57.98% and the genome length is 2.0 Mbp.
**Description of *Candidatus* Agathobaculum merdigallinarum sp. nov.**
*Candidatus* Agathobaculum merdigallinarum (mer.di.gal.li.na’rum. L. fem. n. *merda* faeces; L. fem. n. *gallina* hen; N.L. gen. n. *merdigallinarum* of hen faeces)
A bacterial species identified by metagenomic analyses. This species includes all bacteria with genomes that show ≥95% average nucleotide identity (ANI) to the type genome, which has been assigned the MAG ID ChiSjej1B19-3834 and which is available via NCBI BioSample SAMN15816715. The GC content of the type genome is 57.98% and the genome length is 2.0 Mbp.
**Description of *Candidatus* Agathobaculum merdipullorum sp. nov.**
*Candidatus* Agathobaculum merdipullorum (mer.di.pul.lo’rum. L. fem. n. *merda* faeces; L. masc. n. *pullus* a young chicken; N.L. gen. n. *merdipullorum* of the faeces of young chickens)
A bacterial species identified by metagenomic analyses. This species includes all bacteria with genomes that show ≥95% average nucleotide identity (ANI) to the type genome, which has been assigned the MAG ID CHK149-1869 and which is available via NCBI BioSample SAMN15816722. The GC content of the type genome is 56.28% and the genome length is 1.7 Mbp.
**Description of *Candidatus* Agathobaculum pullicola sp. nov.**
*Candidatus* Agathobaculum pullicola (pul.li’co.la. L. masc. n. *pullus* a young chicken; L. suff. *-cola* inhabitant of; N.L. n. *pullicola* an inhabitant of young chickens)
A bacterial species identified by metagenomic analyses. This species includes all bacteria with genomes that show ≥95% average nucleotide identity (ANI) to the type genome, which has been assigned the MAG ID 2940 and which is available via NCBI BioSample SAMN15816725. The GC content of the type genome is 54.80% and the genome length is 2.0 Mbp.
**Description of *Candidatus* Agathobaculum pullistercoris sp. nov.**
*Candidatus* Agathobaculum pullistercoris (pul.li.ster’co.ris. L. masc. n. *pullus* a young chicken; L. neut. n. *stercus* dung; N.L. gen. n. *pullistercoris* of young chicken faeces)
A bacterial species identified by metagenomic analyses. This species includes all bacteria with genomes that show ≥95% average nucleotide identity (ANI) to the type genome, which has been assigned the MAG ID CHK180-9785 and which is available via NCBI BioSample SAMN15816619. The GC content of the type genome is 58.01% and the genome length is 2.3 Mbp.
**Description of *Candidatus* Agathobaculum stercoravium sp. nov.**
*Candidatus* Agathobaculum stercoravium (ster.cor.a’vi.um. L. neut. n. *stercus* dung; L. fem. n. *avis* bird; N.L. gen. n. *stercoravium* of bird faeces)
A bacterial species identified by metagenomic analyses. This species includes all bacteria with genomes that show ≥95% average nucleotide identity (ANI) to the type genome, which has been assigned the MAG ID ChiW21-6059 and which is available via NCBI BioSample SAMN15816625. The GC content of the type genome is 59.83% and the genome length is 2.3 Mbp.
**Description of *Candidatus* Agrococcus pullicola sp. nov.**
*Candidatus* Agrococcus pullicola (pul.li’co.la. L. masc. n. *pullus* a young chicken; L. suff. *-cola* inhabitant of; N.L. n. *pullicola* an inhabitant of young chickens)
A bacterial species identified by metagenomic analyses. This species includes all bacteria with genomes that show ≥95% average nucleotide identity (ANI) to the type genome, which has been assigned the MAG ID ChiGjej1B1-98 and which is available via NCBI BioSample SAMN15816710. The GC content of the type genome is 63.86% and the genome length is 3.0 Mbp.

**Table:** 

**Description of *Candidatus* Akkermansia intestinavium sp. nov.**
*Candidatus* Akkermansia intestinavium (in.tes.tin.a’vi.um. L. neut. n. *intestinum* gut; L. fem. n. *avis* bird; N.L. gen. n. *intestinavium* of the gut of birds)
A bacterial species identified by metagenomic analyses. This species includes all bacteria with genomes that show ≥95% average nucleotide identity (ANI) to the type genome, which has been assigned the MAG ID ChiGjej6B6-8097 and which is available via NCBI BioSample SAMN15816856. This is a new name for the alphanumeric GTDB species sp900548895. The GC content of the type genome is 65.09% and the genome length is 2.2 Mbp.
**Description of *Candidatus* Akkermansia intestinigallinarum sp. nov.**
*Candidatus* Akkermansia intestinigallinarum (in.tes.ti.ni.gal.li.na’rum. L. neut. n. *intestinum* gut; L. fem. n. *gallina* hen; N.L. gen. n. *intestinigallinarum* of the gut of the hens)
A bacterial species identified by metagenomic analyses. This species includes all bacteria with genomes that show ≥95% average nucleotide identity (ANI) to the type genome, which has been assigned the MAG ID 14975 and which is available via NCBI BioSample SAMN15816742. The GC content of the type genome is 63.40% and the genome length is 2.1 Mbp.
**Description of *Candidatus* Alectryobacillus gen. nov.**
*Candidatus* Alectryobacillus (A.lec.try.o.ba.cil’lus. Gr. neut. n. *alektryon* chicken; L. masc. n. *bacillus* a rod; N.L. masc. n. *Alectryobacillus* a bacillus found in poultry)
A bacterial genus identified by metagenomic analyses. The genus includes all bacteria with genomes that show ≥60% average amino acid identity (AAI) to the type genome from the type species *Candidatus* Alectryobacillus merdavium. This genus has been assigned by GTDB-Tk v1.3.0 working on GTDB Release 05-RS95 (Chaumeil et al., 2019; Parks et al., 2020) to the order *RFN20* and to the family *CAG-826*.
**Description of *Candidatus* Alectryobacillus merdavium sp. nov.**
*Candidatus* Alectryobacillus merdavium (merd.a’vi.um. L. fem. n. *merda* faeces; L. fem. n. *avis* bird; N.L. gen. n. *merdavium* of bird faeces)
A bacterial species identified by metagenomic analyses. This species includes all bacteria with genomes that show ≥95% average nucleotide identity (ANI) to the type genome, which has been assigned the MAG ID 13038 and which is available via NCBI BioSample SAMN15816966. The GC content of the type genome is 27.10% and the genome length is 1.2 Mbp.
**Description of *Candidatus* Alectryocaccobium gen. nov.**
*Candidatus* Alectryocaccobium (A.lec.try.o.cac.co’bi.um. Gr. neut. n. *alektryon* chicken; Gr. fem. n. *kakke* faeces; Gr. masc. n. *bios* life; N.L. neut. n. *Alectryocaccobium* A life form found in chicken faceces)
A bacterial genus identified by metagenomic analyses. The genus includes all bacteria with genomes that show ≥60% average amino acid identity (AAI) to the type genome from the type species *Candidatus* Alectryocaccobium stercorigallinarum. This genus has been assigned by GTDB-Tk v1.3.0 working on GTDB Release 05-RS95 (Chaumeil et al., 2019; Parks et al., 2020) to the order *Lachnospirales* and to the family *Lachnospiraceae*.
**Description of *Candidatus* Alectryocaccobium stercorigallinarum sp. nov.**
*Candidatus* Alectryocaccobium stercorigallinarum (ster.co.ri.gal.li.na’rum. L. neut. n. *stercus* dung; L. fem. n. *gallina* hen; N.L. gen. n. *stercorigallinarum* of hen faeces)
A bacterial species identified by metagenomic analyses. This species includes all bacteria with genomes that show ≥95% average nucleotide identity (ANI) to the type genome, which has been assigned the MAG ID ChiGjej2B2-785 and which is available via NCBI BioSample SAMN15816998. The GC content of the type genome is 46.32% and the genome length is 1.5 Mbp.
**Description of *Candidatus* Alectryocaccomicrobium gen. nov.**
*Candidatus* Alectryocaccomicrobium (A.lec.try.o.cac.co.mi.cro’bi.um. Gr. neut. n. *alektryon* chicken; Gr. fem. n. *kakke* faeces; N.L. neut. n. *microbium* a microbe; N.L. neut. n. *Alectryocaccomicrobium* A microbe found in chicken faceces)
A bacterial genus identified by metagenomic analyses. The genus includes all bacteria with genomes that show ≥60% average amino acid identity (AAI) to the type genome from the type species *Candidatus* Alectryocaccomicrobium excrementavium. This genus has been assigned by GTDB-Tk v1.3.0 working on GTDB Release 05-RS95 (Chaumeil et al., 2019; Parks et al., 2020) to the order *Christensenellales* and to the family *CAG-74*.
**Description of *Candidatus* Alectryocaccomicrobium excrementavium sp. nov.**
*Candidatus* Alectryocaccomicrobium excrementavium (ex.cre.ment.a’vi.um. L. neut. n. *excrementum* excrement; L. fem. n. *avis* bird; N.L. gen. n. *excrementavium* of bird excrement)
A bacterial species identified by metagenomic analyses. This species includes all bacteria with genomes that show ≥95% average nucleotide identity (ANI) to the type genome, which has been assigned the MAG ID 13766 and which is available via NCBI BioSample SAMN15816965. The GC content of the type genome is 59.90% and the genome length is 3.0 Mbp.

**Table:** 

**Description of *Candidatus* Alistipes avicola sp. nov.**
*Candidatus* Alistipes avicola (a.vi’co.la. L. fem. n. *avis* bird; L. suff. *-cola* inhabitant of; N.L. n. *avicola* inhabitant of birds)
A bacterial species identified by metagenomic analyses. This species includes all bacteria with genomes that show ≥95% average nucleotide identity (ANI) to the type genome, which has been assigned the MAG ID CHK169-11906 and which is available via NCBI BioSample SAMN15816659. The GC content of the type genome is 53.79% and the genome length is 1.6 Mbp.
**Description of *Candidatus* Alistipes avistercoris sp. nov.**
*Candidatus* Alistipes avistercoris (a.vi.ster’co.ris. L. fem. n. *avis* bird; L. neut. n. *stercus* dung; N.L. gen. n. *avistercoris* of bird faeces)
A bacterial species identified by metagenomic analyses. This species includes all bacteria with genomes that show ≥95% average nucleotide identity (ANI) to the type genome, which has been assigned the MAG ID 653 and which is available via NCBI BioSample SAMN15816855. This is a new name for the alphanumeric GTDB species sp000434235. The GC content of the type genome is 62.33% and the genome length is 2.4 Mbp.
**Description of *Candidatus* Alistipes cottocaccae sp. nov.**
*Candidatus* Alistipes cottocaccae (cot.to.cac’cae. Gr. masc. n. *kottos* chicken Gr. fem. n. *kakke* faeces; N.L. gen. n. *cottocaccae* of chicken faeces)
A bacterial species identified by metagenomic analyses. This species includes all bacteria with genomes that show ≥95% average nucleotide identity (ANI) to the type genome, which has been assigned the MAG ID ChiBcec16-1783 and which is available via NCBI BioSample SAMN15816853. This is a new name for the alphanumeric GTDB species sp002161445. The GC content of the type genome is 60.94% and the genome length is 2.4 Mbp.
**Description of *Candidatus* Alistipes excrementavium sp. nov.**
*Candidatus* Alistipes excrementavium (ex.cre.ment.a’vi.um. L. neut. n. *excrementum* excrement; L. fem. n. *avis* bird; N.L. gen. n. *excrementavium* of bird excrement)
A bacterial species identified by metagenomic analyses. This species includes all bacteria with genomes that show ≥95% average nucleotide identity (ANI) to the type genome, which has been assigned the MAG ID CHK15-232 and which is available via NCBI BioSample SAMN15816809. This is a new name for the alphanumeric GTDB species sp900021155. The GC content of the type genome is 61.18% and the genome length is 2.2 Mbp.
**Description of *Candidatus* Alistipes excrementigallinarum sp. nov.**
*Candidatus* Alistipes excrementigallinarum (ex.cre.men.ti.gal.li.na’rum. L. neut. n. *excrementum* excrement; L. fem. n. *gallina* hen; N.L. gen. n. *excrementigallinarum* of hen excrement)
A bacterial species identified by metagenomic analyses. This species includes all bacteria with genomes that show ≥95% average nucleotide identity (ANI) to the type genome, which has been assigned the MAG ID CHK106-249 and which is available via NCBI BioSample SAMN15816875. The GC content of the type genome is 63.33% and the genome length is 2.3 Mbp.
**Description of *Candidatus* Alistipes excrementipullorum sp. nov.**
*Candidatus* Alistipes excrementipullorum (ex.cre.men.ti.pul.lo’rum. L. neut. n. *excrementum* excrement; L. masc. n. *pullus* a young chicken; N.L. gen. n. *excrementipullorum* of young chicken excrement)
A bacterial species identified by metagenomic analyses. This species includes all bacteria with genomes that show ≥95% average nucleotide identity (ANI) to the type genome, which has been assigned the MAG ID ChiHjej8B7-9065 and which is available via NCBI BioSample SAMN15816799. This is a new name for the alphanumeric GTDB species. The GC content of the type genome is 56.25% and the genome length is 1.7 Mbp.
**Description of *Candidatus* Alistipes faecavium sp. nov.**
*Candidatus* Alistipes faecavium (faec.a’vi.um. L. fem. n. *faex, faecis* excrement; L. fem. n. *avis* bird; N.L. gen. n. *faecavium* of bird faeces)
A bacterial species identified by metagenomic analyses. This species includes all bacteria with genomes that show ≥95% average nucleotide identity (ANI) to the type genome, which has been assigned the MAG ID ChiGjej2B2-19477 and which is available via NCBI BioSample SAMN15816800. The GC content of the type genome is 62.24% and the genome length is 2.3 Mbp.
**Description of *Candidatus* Alistipes faecigallinarum sp. nov.**
*Candidatus* Alistipes faecigallinarum (fae.ci.gal.li.na’rum. L. fem. n. *faex, faecis* excrement; L. fem. n. *gallina* hen; N.L. gen. n. *faecigallinarum* of chicken faeces)
A bacterial species identified by metagenomic analyses. This species includes all bacteria with genomes that show ≥95% average nucleotide identity (ANI) to the type genome, which has been assigned the MAG ID 6451 and which is available via NCBI BioSample SAMN15816915. The GC content of the type genome is 61.37% and the genome length is 2.2 Mbp.
**Description of *Candidatus* Alistipes intestinigallinarum sp. nov.**
*Candidatus* Alistipes intestinigallinarum (in.tes.ti.ni.gal.li.na’rum. L. neut. n. *intestinum* gut; L. fem. n. *gallina* hen; N.L. gen. n. *intestinigallinarum* of the gut of the hens)
A bacterial species identified by metagenomic analyses. This species includes all bacteria with genomes that show ≥95% average nucleotide identity (ANI) to the type genome, which has been assigned the MAG ID 5134 and which is available via NCBI BioSample SAMN15816708. The GC content of the type genome is 59.58% and the genome length is 2.7 Mbp.

**Table:** 

**Description of *Candidatus* Alistipes intestinipullorum sp. nov.**
*Candidatus* Alistipes intestinipullorum (in.tes.ti.ni.pul.lo’rum. L. neut. n. *intestinum* gut; L. masc. n. *pullus* a young chicken; N.L. gen. n. *intestinipullorum* of the gut of young chickens)
A bacterial species identified by metagenomic analyses. This species includes all bacteria with genomes that show ≥95% average nucleotide identity (ANI) to the type genome, which has been assigned the MAG ID ChiGjej2B2-5998 and which is available via NCBI BioSample SAMN15816759. The GC content of the type genome is 59.58% and the genome length is 2.3 Mbp.
**Description of *Candidatus* Alistipes merdavium sp. nov.**
*Candidatus* Alistipes merdavium (merd.a’vi.um. L. fem. n. *merda* faeces; L. fem. n. *avis* bird; N.L. gen. n. *merdavium* of bird faeces)
A bacterial species identified by metagenomic analyses. This species includes all bacteria with genomes that show ≥95% average nucleotide identity (ANI) to the type genome, which has been assigned the MAG ID ChiBcolR5-1230 and which is available via NCBI BioSample SAMN15816813. This is a new name for the alphanumeric GTDB species sp900544265. The GC content of the type genome is 63.44% and the genome length is 2.2 Mbp.
**Description of *Candidatus* Alistipes merdigallinarum sp. nov.**
*Candidatus* Alistipes merdigallinarum (mer.di.gal.li.na’rum. L. fem. n. *merda* faeces; L. fem. n. *gallina* hen; N.L. gen. n. *merdigallinarum* of hen faeces)
A bacterial species identified by metagenomic analyses. This species includes all bacteria with genomes that show ≥95% average nucleotide identity (ANI) to the type genome, which has been assigned the MAG ID 2432 and which is available via NCBI BioSample SAMN15816893. Although GTDB has assigned this species to the genus it calls Alistipes_A, this genus designation cannot be incorporated into a well-formed binomial, so in naming this species, we have used the current validly published name for the genus. The GC content of the type genome is 49.96% and the genome length is 2.2 Mbp.
**Description of *Candidatus* Alistipes merdipullorum sp. nov.**
*Candidatus* Alistipes merdipullorum (mer.di.pul.lo’rum. L. fem. n. *merda* faeces; L. masc. n. *pullus* a young chicken; N.L. gen. n. *merdipullorum* of the faeces of young chickens)
A bacterial species identified by metagenomic analyses. This species includes all bacteria with genomes that show ≥95% average nucleotide identity (ANI) to the type genome, which has been assigned the MAG ID ChiHjej9B8-3741 and which is available via NCBI BioSample SAMN15816807. This is a new name for the alphanumeric GTDB species sp900546065. The GC content of the type genome is 57.66% and the genome length is 2.3 Mbp.
**Description of *Candidatus* Alistipes pullicola sp. nov.**
*Candidatus* Alistipes pullicola (pul.li’co.la. L. masc. n. *pullus* a young chicken; L. suff. *-cola* inhabitant of; N.L. n. *pullicola* an inhabitant of young chickens)
A bacterial species identified by metagenomic analyses. This species includes all bacteria with genomes that show ≥95% average nucleotide identity (ANI) to the type genome, which has been assigned the MAG ID ChiHjej10B9-11434 and which is available via NCBI BioSample SAMN15816929. This is a new name for the alphanumeric GTDB species sp900546005. Although GTDB has assigned this species to the genus it calls Alistipes_A, this genus designation cannot be incorporated into a well-formed binomial, so in naming this species, we have used the current validly published name for the genus. The GC content of the type genome is 52.02% and the genome length is 1.9 Mbp.
**Description of *Candidatus* Alistipes pullistercoris sp. nov.**
*Candidatus* Alistipes pullistercoris (pul.li.ster’co.ris. L. masc. n. *pullus* a young chicken; L. neut. n. *stercus* dung; N.L. gen. n. *pullistercoris* of young chicken faeces)
A bacterial species identified by metagenomic analyses. This species includes all bacteria with genomes that show ≥95% average nucleotide identity (ANI) to the type genome, which has been assigned the MAG ID 3244 and which is available via NCBI BioSample SAMN15816930. This is a new name for the alphanumeric GTDB species sp900240235. Although GTDB has assigned this species to the genus it calls Alistipes_A, this genus designation cannot be incorporated into a well-formed binomial, so in naming this species, we have used the current validly published name for the genus. The GC content of the type genome is 56.88% and the genome length is 2.0 Mbp.
**Description of *Candidatus* Alistipes stercoravium sp. nov.**
*Candidatus* Alistipes stercoravium (ster.cor.a’vi.um. L. neut. n. *stercus* dung; L. fem. n. *avis* bird; N.L. gen. n. *stercoravium* of bird faeces)
A bacterial species identified by metagenomic analyses. This species includes all bacteria with genomes that show ≥95% average nucleotide identity (ANI) to the type genome, which has been assigned the MAG ID ChiHjej8B7-9257 and which is available via NCBI BioSample SAMN15816640. The GC content of the type genome is 61.39% and the genome length is 2.0 Mbp.
**Description of *Candidatus* Alistipes stercorigallinarum sp. nov.**
*Candidatus* Alistipes stercorigallinarum (ster.co.ri.gal.li.na’rum. L. neut. n. *stercus* dung; L. fem. n. *gallina* hen; N.L. gen. n. *stercorigallinarum* of hen faeces)
A bacterial species identified by metagenomic analyses. This species includes all bacteria with genomes that show ≥95% average nucleotide identity (ANI) to the type genome, which has been assigned the MAG ID ChiHcolR4-13572 and which is available via NCBI BioSample SAMN15816817. This is a new name for the alphanumeric GTDB species sp900542505. The GC content of the type genome is 62.42% and the genome length is 2.2 Mbp.

**Table:** 

**Description of *Candidatus* Alistipes stercoripullorum sp. nov.**
*Candidatus* Alistipes stercoripullorum (ster.co.ri.pul.lo’rum. L. neut. n. *stercus* dung; L. masc. n. *pullus* a young chicken; N.L. gen. n. *stercoripullorum* of the faceces of young chickens)
A bacterial species identified by metagenomic analyses. This species includes all bacteria with genomes that show ≥95% average nucleotide identity (ANI) to the type genome, which has been assigned the MAG ID ChiBcec8-6454 and which is available via NCBI BioSample SAMN15816818. This is a new name for the alphanumeric GTDB species sp006542685. The GC content of the type genome is 62.87% and the genome length is 2.4 Mbp.
**Description of *Candidatus* Anaerobiospirillum merdipullorum sp. nov.**
*Candidatus* Anaerobiospirillum merdipullorum (mer.di.pul.lo’rum. L. fem. n. *merda* faeces; L. masc. n. *pullus* a young chicken; N.L. gen. n. *merdipullorum* of the faeces of young chickens)
A bacterial species identified by metagenomic analyses. This species includes all bacteria with genomes that show ≥95% average nucleotide identity (ANI) to the type genome, which has been assigned the MAG ID 687 and which is available via NCBI BioSample SAMN15816911. Although GTDB has assigned this species to the genus it calls Anaerobiospirillum_A, this genus designation cannot be incorporated into a well-formed binomial, so in naming this species, we have used the current validly published name for the genus. The GC content of the type genome is 49.84% and the genome length is 2.0 Mbp.
**Description of *Candidatus* Anaerobiospirillum pullicola sp. nov.**
*Candidatus* Anaerobiospirillum pullicola (pul.li’co.la. L. masc. n. *pullus* a young chicken; L. suff. *-cola* inhabitant of; N.L. n. *pullicola* an inhabitant of young chickens)
A bacterial species identified by metagenomic analyses. This species includes all bacteria with genomes that show ≥95% average nucleotide identity (ANI) to the type genome, which has been assigned the MAG ID 378 and which is available via NCBI BioSample SAMN15816727. The GC content of the type genome is 52.37% and the genome length is 3.9 Mbp.
**Description of *Candidatus* Anaerobiospirillum pullistercoris sp. nov.**
*Candidatus* Anaerobiospirillum pullistercoris (pul.li.ster’co.ris. L. masc. n. *pullus* a young chicken; L. neut. n. *stercus* dung; N.L. gen. n. *pullistercoris* of young chicken faeces)
A bacterial species identified by metagenomic analyses. This species includes all bacteria with genomes that show ≥95% average nucleotide identity (ANI) to the type genome, which has been assigned the MAG ID USASDec5-558 and which is available via NCBI BioSample SAMN15816730. The GC content of the type genome is 49.01% and the genome length is 3.3 Mbp.
**Description of *Candidatus* Anaerobiospirillum stercoravium sp. nov.**
*Candidatus* Anaerobiospirillum stercoravium (ster.cor.a’vi.um. L. neut. n. *stercus* dung; L. fem. n. *avis* bird; N.L. gen. n. *stercoravium* of bird faeces)
A bacterial species identified by metagenomic analyses. This species includes all bacteria with genomes that show ≥95% average nucleotide identity (ANI) to the type genome, which has been assigned the MAG ID USASDcec2-551 and which is available via NCBI BioSample SAMN15816778. The GC content of the type genome is 56.27% and the genome length is 2.9 Mbp.
**Description of *Candidatus* Anaerobutyricum avicola sp. nov.**
*Candidatus* Anaerobutyricum avicola (a.vi’co.la. L. fem. n. *avis* bird; L. suff. *-cola* inhabitant of; N.L. n. *avicola* inhabitant of birds)
A bacterial species identified by metagenomic analyses. This species includes all bacteria with genomes that show ≥95% average nucleotide identity (ANI) to the type genome, which has been assigned the MAG ID ChiSxjej6B18-9268 and which is available via NCBI BioSample SAMN15816760. The GC content of the type genome is 50.20% and the genome length is 2.5 Mbp.
**Description of *Candidatus* Anaerobutyricum faecale sp. nov.**
*Candidatus* Anaerobutyricum faecale (fae.ca’le. L. neut. adj. *faecale* of faeces)
A bacterial species identified by metagenomic analyses. This species includes all bacteria with genomes that show ≥95% average nucleotide identity (ANI) to the type genome, which has been assigned the MAG ID CHK182-24705 and which is available via NCBI BioSample SAMN15816814. This is a new name for the alphanumeric GTDB species sp002161065. The GC content of the type genome is 48.07% and the genome length is 2.8 Mbp.
**Description of *Candidatus* Anaerobutyricum stercoripullorum sp. nov.**
*Candidatus* Anaerobutyricum stercoripullorum (ster.co.ri.pul.lo’rum. L. neut. n. *stercus* dung; L. masc. n. *pullus* a young chicken; N.L. gen. n. *stercoripullorum* of the faceces of young chickens)
A bacterial species identified by metagenomic analyses. This species includes all bacteria with genomes that show ≥95% average nucleotide identity (ANI) to the type genome, which has been assigned the MAG ID ChiSxjej3B15-1167 and which is available via NCBI BioSample SAMN15816729. The GC content of the type genome is 52.36% and the genome length is 2.3 Mbp.
**Description of *Candidatus* Anaerobutyricum stercoris sp. nov.**
*Candidatus* Anaerobutyricum stercoris (ster’co.ris. L. gen. n. *stercoris* of dung, excrement)
A bacterial species identified by metagenomic analyses. This species includes all bacteria with genomes that show ≥95% average nucleotide identity (ANI) to the type genome, which has been assigned the MAG ID CHK179-28034 and which is available via NCBI BioSample SAMN15816848. This is a new name for the alphanumeric GTDB species sp900016875. The GC content of the type genome is 47.36% and the genome length is 3.0 Mbp.

**Table:** 

**Description of *Candidatus* Anaerofilum excrementigallinarum sp. nov.**
*Candidatus* Anaerofilum excrementigallinarum (ex.cre.men.ti.gal.li.na’rum. L. neut. n. *excrementum* excrement; L. fem. n. *gallina* hen; N.L. gen. n. *excrementigallinarum* of hen excrement)
A bacterial species identified by metagenomic analyses. This species includes all bacteria with genomes that show ≥95% average nucleotide identity (ANI) to the type genome, which has been assigned the MAG ID 3951 and which is available via NCBI BioSample SAMN15816720. The GC content of the type genome is 61.37% and the genome length is 2.5 Mbp.
**Description of *Candidatus* Anaerofilum faecale sp. nov.**
*Candidatus* Anaerofilum faecale (fae.ca’le. L. neut. adj. *faecale* of faeces)
A bacterial species identified by metagenomic analyses. This species includes all bacteria with genomes that show ≥95% average nucleotide identity (ANI) to the type genome, which has been assigned the MAG ID ChiGjej6B6-374 and which is available via NCBI BioSample SAMN15816865. This is a new name for the alphanumeric GTDB species sp002160015. The GC content of the type genome is 63.11% and the genome length is 2.3 Mbp.
**Description of *Candidatus* Anaeromassilibacillus stercoravium sp. nov.**
*Candidatus* Anaeromassilibacillus stercoravium (ster.cor.a’vi.um. L. neut. n. *stercus* dung; L. fem. n. *avis* bird; N.L. gen. n. *stercoravium* of bird faeces)
A bacterial species identified by metagenomic analyses. This species includes all bacteria with genomes that show ≥95% average nucleotide identity (ANI) to the type genome, which has been assigned the MAG ID ChiSjej5B23-4625 and which is available via NCBI BioSample SAMN15816824. This is a new name for the alphanumeric GTDB species sp002159845. The GC content of the type genome is 54.17% and the genome length is 2.2 Mbp.
**Description of *Candidatus* Anaerostipes avicola sp. nov.**
*Candidatus* Anaerostipes avicola (a.vi’co.la. L. fem. n. *avis* bird; L. suff. *-cola* inhabitant of; N.L. n. *avicola* inhabitant of birds)
A bacterial species identified by metagenomic analyses. This species includes all bacteria with genomes that show ≥95% average nucleotide identity (ANI) to the type genome, which has been assigned the MAG ID CHK189-27985 and which is available via NCBI BioSample SAMN15816576. The GC content of the type genome is 43.22% and the genome length is 2.5 Mbp.
**Description of *Candidatus* Anaerostipes avistercoris sp. nov.**
*Candidatus* Anaerostipes avistercoris (a.vi.ster’co.ris. L. fem. n. *avis* bird; L. neut. n. *stercus* dung; N.L. gen. n. *avistercoris* of bird faeces)
A bacterial species identified by metagenomic analyses. This species includes all bacteria with genomes that show ≥95% average nucleotide identity (ANI) to the type genome, which has been assigned the MAG ID ChiSjej3B21-8574 and which is available via NCBI BioSample SAMN15816634. The GC content of the type genome is 44.43% and the genome length is 2.6 Mbp.
**Description of *Candidatus* Anaerostipes excrementavium sp. nov.**
*Candidatus* Anaerostipes excrementavium (ex.cre.ment.a’vi.um. L. neut. n. *excrementum* excrement; L. fem. n. *avis* bird; N.L. gen. n. *excrementavium* of bird excrement)
A bacterial species identified by metagenomic analyses. This species includes all bacteria with genomes that show ≥95% average nucleotide identity (ANI) to the type genome, which has been assigned the MAG ID CHK191-13928 and which is available via NCBI BioSample SAMN15816615. The GC content of the type genome is 41.56% and the genome length is 2.7 Mbp.
**Description of *Candidatus* Anaerotignum merdipullorum sp. nov.**
*Candidatus* Anaerotignum merdipullorum (mer.di.pul.lo’rum. L. fem. n. *merda* faeces; L. masc. n. *pullus* a young chicken; N.L. gen. n. *merdipullorum* of the faeces of young chickens)
A bacterial species identified by metagenomic analyses. This species includes all bacteria with genomes that show ≥95% average nucleotide identity (ANI) to the type genome, which has been assigned the MAG ID CHK190-6203 and which is available via NCBI BioSample SAMN15816613. The GC content of the type genome is 44.75% and the genome length is 2.2 Mbp.
**Description of *Candidatus* Anaerotruncus excrementipullorum sp. nov.**
*Candidatus* Anaerotruncus excrementipullorum (ex.cre.men.ti.pul.lo’rum. L. neut. n. *excrementum* excrement; L. masc. n. *pullus* a young chicken; N.L. gen. n. *excrementipullorum* of young chicken excrement)
A bacterial species identified by metagenomic analyses. This species includes all bacteria with genomes that show ≥95% average nucleotide identity (ANI) to the type genome, which has been assigned the MAG ID CHK188-5543 and which is available via NCBI BioSample SAMN15816616. The GC content of the type genome is 64.05% and the genome length is 1.9 Mbp.
**Description of *Candidatus* Aphodenecus gen. nov.**
*Candidatus* Aphodenecus (Aph.od.en.e’cus. Gr. fem. n. *aphodos* dung; Gr. masc. *enoikos* inhabitant; N.L. masc. n. *Aphodenecus* a microbe associated with faeces)
A bacterial genus identified by metagenomic analyses. The genus includes all bacteria with genomes that show ≥60% average amino acid identity (AAI) to the type genome from the type species *Candidatus* Aphodenecus pullistercoris. This is a name for the alphanumeric GTDB genus Spiro-01. This genus has been assigned by GTDB-Tk v1.3.0 working on GTDB Release 05-RS95 (Chaumeil et al., 2019; Parks et al., 2020) to the order *Sphaerochaetales* and to the family *Sphaerochaetaceae*.

**Table:** 

**Description of *Candidatus* Aphodenecus pullistercoris sp. nov.**
*Candidatus* Aphodenecus pullistercoris (pul.li.ster’co.ris. L. masc. n. *pullus* a young chicken; L. neut. n. *stercus* dung; N.L. gen. n. *pullistercoris* of young chicken faeces)
A bacterial species identified by metagenomic analyses. This species includes all bacteria with genomes that show ≥95% average nucleotide identity (ANI) to the type genome, which has been assigned the MAG ID 11167 and which is available via NCBI BioSample SAMN15817123. The GC content of the type genome is 59.34% and the genome length is 2.0 Mbp.
**Description of *Candidatus* Aphodocola gen. nov.**
*Candidatus* Aphodocola (Aph.o.do’co.la. Gr. fem. n. *aphodos* dung; L. suff. *-cola* inhabitant of; N.L. fem. n. *Aphodocola* a microbe associated with faeces)
A bacterial genus identified by metagenomic analyses. The genus includes all bacteria with genomes that show ≥60% average amino acid identity (AAI) to the type genome from the type species *Candidatus* Aphodocola excrementigallinarum. This is a name for the alphanumeric GTDB genus CAG-594. This genus has been assigned by GTDB-Tk v1.3.0 working on GTDB Release 05-RS95 (Chaumeil et al., 2019; Parks et al., 2020) to the order *RF39* and to the family *CAG-433*.
**Description of *Candidatus* Aphodocola excrementigallinarum sp. nov.**
*Candidatus* Aphodocola excrementigallinarum (ex.cre.men.ti.gal.li.na’rum. L. neut. n. *excrementum* excrement; L. fem. n. *gallina* hen; N.L. gen. n. *excrementigallinarum* of hen excrement)
A bacterial species identified by metagenomic analyses. This species includes all bacteria with genomes that show ≥95% average nucleotide identity (ANI) to the type genome, which has been assigned the MAG ID CHK193-30670 and which is available via NCBI BioSample SAMN15817049. The GC content of the type genome is 27.74% and the genome length is 1.2 Mbp.
**Description of *Candidatus* Aphodomonas gen. nov.**
*Candidatus* Aphodomonas (Aph.o.do.mo’nas. Gr. fem. n. *aphodos* dung; L. fem. n. *monas* a monad; N.L. fem. n. *Aphodomonas* a microbe associated with faeces)
A bacterial genus identified by metagenomic analyses. The genus includes all bacteria with genomes that show ≥60% average amino acid identity (AAI) to the type genome from the type species *Candidatus* Aphodomonas merdavium. This is a name for the alphanumeric GTDB genus SFFS01. This genus has been assigned by GTDB-Tk v1.3.0 working on GTDB Release 05-RS95 (Chaumeil et al., 2019; Parks et al., 2020) to the order *Christensenellales* and to the family *CAG-74*.
**Description of *Candidatus* Aphodomonas merdavium sp. nov.**
*Candidatus* Aphodomonas merdavium (merd.a’vi.um. L. fem. n. *merda* faeces; L. fem. n. *avis* bird; N.L. gen. n. *merdavium* of bird faeces)
A bacterial species identified by metagenomic analyses. This species includes all bacteria with genomes that show ≥95% average nucleotide identity (ANI) to the type genome, which has been assigned the MAG ID ChiGjej2B2-35035 and which is available via NCBI BioSample SAMN15817117. The GC content of the type genome is 59.45% and the genome length is 2.1 Mbp.
**Description of *Candidatus* Aphodomorpha gen. nov.**
*Candidatus* Aphodomorpha (Aph.o.do.mor’pha. Gr. fem. n. *aphodos* dung; Gr. fem. n. *morphe* a form, shape; N.L. fem. n. *Aphodomorpha* a microbe associated with faeces)
A bacterial genus identified by metagenomic analyses. The genus includes all bacteria with genomes that show ≥60% average amino acid identity (AAI) to the type genome from the type species *Candidatus* Aphodomorpha intestinavium. This is a name for the alphanumeric GTDB genus UMGS1241. This genus has been assigned by GTDB-Tk v1.3.0 working on GTDB Release 05-RS95 (Chaumeil et al., 2019; Parks et al., 2020) to the order *Christensenellales* and to the family *CAG-138*.
**Description of *Candidatus* Aphodomorpha intestinavium sp. nov.**
*Candidatus* Aphodomorpha intestinavium (in.tes.tin.a’vi.um. L. neut. n. *intestinum* gut; L. fem. n. *avis* bird; N.L. gen. n. *intestinavium* of the gut of birds)
A bacterial species identified by metagenomic analyses. This species includes all bacteria with genomes that show ≥95% average nucleotide identity (ANI) to the type genome, which has been assigned the MAG ID ChiGjej2B2-16831 and which is available via NCBI BioSample SAMN15817204. This is a new name for the alphanumeric GTDB species sp900550525. The GC content of the type genome is 68.13% and the genome length is 1.6 Mbp.
**Description of *Candidatus* Aphodoplasma gen. nov.**
*Candidatus* Aphodoplasma (Aph.o.do.plas’ma. Gr. fem. n. *aphodos* dung; Gr. neut. n. *plasma* a form; N.L. neut. n. *Aphodoplasma* a microbe associated with faeces)
A bacterial genus identified by metagenomic analyses. The genus includes all bacteria with genomes that show ≥60% average amino acid identity (AAI) to the type genome from the type species *Candidatus* Aphodoplasma excrementigallinarum. This is a name for the alphanumeric GTDB genus UMGS1253. This genus has been assigned by GTDB-Tk v1.3.0 working on GTDB Release 05-RS95 (Chaumeil et al., 2019; Parks et al., 2020) to the order *Monoglobales_A* and to the family *UMGS1253*.

**Table:** 

**Description of *Candidatus* Aphodoplasma excrementigallinarum sp. nov.**
*Candidatus* Aphodoplasma excrementigallinarum (ex.cre.men.ti.gal.li.na’rum. L. neut. n. *excrementum* excrement; L. fem. n. *gallina* hen; N.L. gen. n. *excrementigallinarum* of hen excrement)
A bacterial species identified by metagenomic analyses. This species includes all bacteria with genomes that show ≥95% average nucleotide identity (ANI) to the type genome, which has been assigned the MAG ID 4920 and which is available via NCBI BioSample SAMN15817155. The GC content of the type genome is 54.59% and the genome length is 1.8 Mbp.
**Description of *Candidatus* Aphodosoma gen. nov.**
*Candidatus* Aphodosoma (Aph.o.do.so’ma. Gr. fem. n. *aphodos* dung; Gr. neut. n. *soma* a body; N.L. neut. n. *Aphodosoma* a microbe associated with faeces)
A bacterial genus identified by metagenomic analyses. The genus includes all bacteria with genomes that show ≥60% average amino acid identity (AAI) to the type genome from the type species *Candidatus* Aphodosoma intestinipullorum. This is a name for the alphanumeric GTDB genus SFVR01. This genus has been assigned by GTDB-Tk v1.3.0 working on GTDB Release 05-RS95 (Chaumeil et al., 2019; Parks et al., 2020) to the order *Bacteroidales* and to the family *Paludibacteraceae*.
**Description of *Candidatus* Aphodosoma intestinipullorum sp. nov.**
*Candidatus* Aphodosoma intestinipullorum (in.tes.ti.ni.pul.lo’rum. L. neut. n. *intestinum* gut; L. masc. n. *pullus* a young chicken; N.L. gen. n. *intestinipullorum* of the gut of young chickens)
A bacterial species identified by metagenomic analyses. This species includes all bacteria with genomes that show ≥95% average nucleotide identity (ANI) to the type genome, which has been assigned the MAG ID 3924 and which is available via NCBI BioSample SAMN15817132. The GC content of the type genome is 52.56% and the genome length is 2.4 Mbp.
**Description of *Candidatus* Aphodousia gen. nov.**
*Candidatus* Aphodousia (Aph.od.ou’si.a. Gr. fem. n. *aphodos* dung; Gr. fem. n. *ousia* an essence; N.L. fem. n. *Aphodousia* a microbe associated with faeces)
A bacterial genus identified by metagenomic analyses. The genus includes all bacteria with genomes that show ≥60% average amino acid identity (AAI) to the type genome from the type species *Candidatus* Aphodousia faecavium. This is a name for the alphanumeric GTDB genus CAG-521. This genus has been assigned by GTDB-Tk v1.3.0 working on GTDB Release 05-RS95 (Chaumeil et al., 2019; Parks et al., 2020) to the order *Burkholderiales* and to the family *Burkholderiaceae*.
**Description of *Candidatus* Aphodousia faecalis sp. nov.**
*Candidatus* Aphodousia faecalis (fae.ca’lis. L. fem. adj. *faecalis* of faeces)
A bacterial species identified by metagenomic analyses. This species includes all bacteria with genomes that show ≥95% average nucleotide identity (ANI) to the type genome, which has been assigned the MAG ID ChiW13-1064 and which is available via NCBI BioSample SAMN15817170. This is a new name for the alphanumeric GTDB species sp000437635. The GC content of the type genome is 47.35% and the genome length is 1.7 Mbp.
**Description of *Candidatus* Aphodousia faecavium sp. nov.**
*Candidatus* Aphodousia faecavium (faec.a’vi.um. L. fem. n. *faex, faecis* excrement; L. fem. n. *avis* bird; N.L. gen. n. *faecavium* of bird faeces)
A bacterial species identified by metagenomic analyses. This species includes all bacteria with genomes that show ≥95% average nucleotide identity (ANI) to the type genome, which has been assigned the MAG ID 10345 and which is available via NCBI BioSample SAMN15817126. The GC content of the type genome is 48.23% and the genome length is 1.7 Mbp.
**Description of *Candidatus* Aphodousia faecigallinarum sp. nov.**
*Candidatus* Aphodousia faecigallinarum (fae.ci.gal.li.na’rum. L. fem. n. *faex, faecis* excrement; L. fem. n. *gallina* hen; N.L. gen. n. *faecigallinarum* of hen faeces)
A bacterial species identified by metagenomic analyses. This species includes all bacteria with genomes that show ≥95% average nucleotide identity (ANI) to the type genome, which has been assigned the MAG ID 7463 and which is available via NCBI BioSample SAMN15817137. The GC content of the type genome is 48.37% and the genome length is 1.5 Mbp.
**Description of *Candidatus* Aphodousia faecipullorum sp. nov.**
*Candidatus* Aphodousia faecipullorum (fae.ci.pul.lo’rum. L. fem. n. *faex, faecis* excrement; L. masc. n. *pullus* a young chicken; N.L. gen. n. *faecipullorum* of young chicken faeces)
A bacterial species identified by metagenomic analyses. This species includes all bacteria with genomes that show ≥95% average nucleotide identity (ANI) to the type genome, which has been assigned the MAG ID CHK135-12538 and which is available via NCBI BioSample SAMN15817146. The GC content of the type genome is 48.08% and the genome length is 1.8 Mbp.

**Table:** 

**Description of *Candidatus* Aphodousia gallistercoris sp. nov.**
*Candidatus* Aphodousia gallistercoris (gal.li.ster’co.ris. L. masc. n *gallus* chicken; L. neut. n. *stercus* dung; N.L. gen. n. *gallistercoris* of chicken faeces)
A bacterial species identified by metagenomic analyses. This species includes all bacteria with genomes that show ≥95% average nucleotide identity (ANI) to the type genome, which has been assigned the MAG ID CHK121-301 and which is available via NCBI BioSample SAMN15817147. The GC content of the type genome is 52.58% and the genome length is 1.8 Mbp.
**Description of *Candidatus* Aphodovivens gen. nov.**
*Candidatus* Aphodovivens (Aph.o.do.vi’vens. Gr. fem. n. *aphodos* dung; N.L. pres. part. *vivens* living; N.L. fem. n. *Aphodovivens* a microbe associated with faeces)
A bacterial genus identified by metagenomic analyses. The genus includes all bacteria with genomes that show ≥60% average amino acid identity (AAI) to the type genome from the type species *Candidatus* Aphodovivens avicola. This is a name for the alphanumeric GTDB genus UMGS1293. This genus has been assigned by GTDB-Tk v1.3.0 working on GTDB Release 05-RS95 (Chaumeil et al., 2019; Parks et al., 2020) to the order *Coriobacteriales* and to the family *Eggerthellaceae*.
**Description of *Candidatus* Aphodovivens avicola sp. nov.**
*Candidatus* Aphodovivens avicola (a.vi’co.la. L. fem. n. *avis* bird; L. suff. *-cola* inhabitant of; N.L. n. *avicola* inhabitant of birds)
A bacterial species identified by metagenomic analyses. This species includes all bacteria with genomes that show ≥95% average nucleotide identity (ANI) to the type genome, which has been assigned the MAG ID ChiGjej6B6-21069 and which is available via NCBI BioSample SAMN15817067. The GC content of the type genome is 65.54% and the genome length is 2.2 Mbp.
**Description of *Candidatus* Aphodovivens avistercoris sp. nov.**
*Candidatus* Aphodovivens avistercoris (a.vi.ster’co.ris. L. fem. n. *avis* bird; L. neut. n. *stercus* dung; N.L. gen. n. *avistercoris* of bird faeces)
A bacterial species identified by metagenomic analyses. This species includes all bacteria with genomes that show ≥95% average nucleotide identity (ANI) to the type genome, which has been assigned the MAG ID ChiGjej5B5-3278 and which is available via NCBI BioSample SAMN15817093. The GC content of the type genome is 66.86% and the genome length is 2.4 Mbp.
**Description of *Candidatus* Aphodovivens excrementavium sp. nov.**
*Candidatus* Aphodovivens excrementavium (ex.cre.ment.a’vi.um. L. neut. n. *excrementum* excrement; L. fem. n. *avis* bird; N.L. gen. n. *excrementavium* of bird excrement)
A bacterial species identified by metagenomic analyses. This species includes all bacteria with genomes that show ≥95% average nucleotide identity (ANI) to the type genome, which has been assigned the MAG ID ChiGjej2B2-30709 and which is available via NCBI BioSample SAMN15817109. The GC content of the type genome is 58.74% and the genome length is 2.1 Mbp.
**Description of *Candidatus* Aquabacterium excrementipullorum sp. nov.**
*Candidatus* Aquabacterium excrementipullorum (ex.cre.men.ti.pul.lo’rum. L. neut. n. *excrementum* excrement; L. masc. n. *pullus* a young chicken; N.L. gen. n. *excrementipullorum* of young chicken excrement)
A bacterial species identified by metagenomic analyses. This species includes all bacteria with genomes that show ≥95% average nucleotide identity (ANI) to the type genome, which has been assigned the MAG ID ChiHile3-4534 and which is available via NCBI BioSample SAMN15816783. The GC content of the type genome is 67.11% and the genome length is 4.7 Mbp.
**Description of *Candidatus* Atopostipes pullistercoris sp. nov.**
*Candidatus* Atopostipes pullistercoris (pul.li.ster’co.ris. L. masc. n. *pullus* a young chicken; L. neut. n. *stercus* dung; N.L. gen. n. *pullistercoris* of young chicken faeces)
A bacterial species identified by metagenomic analyses. This species includes all bacteria with genomes that show ≥95% average nucleotide identity (ANI) to the type genome, which has been assigned the MAG ID CHK169-4300 and which is available via NCBI BioSample SAMN15816688. The GC content of the type genome is 34.84% and the genome length is 1.9 Mbp.
**Description of *Candidatus* Avacholeplasma gen. nov.**
*Candidatus* Avacholeplasma (Av.a.cho.le.plas’ma. L. fem. n. *avis* bird; N.L. neut. n. *Acholeplasma* a genus name; N.L. neut n. *Avacholeplasma* a genus related to the genus *Acholeplasma* but distinct from it and found in poultry)
A bacterial genus identified by metagenomic analyses. The genus includes all bacteria with genomes that show ≥60% average amino acid identity (AAI) to the type genome from the type species *Candidatus* Avacholeplasma faecigallinarum. This genus has been assigned by GTDB-Tk v1.3.0 working on GTDB Release 05-RS95 (Chaumeil et al., 2019; Parks et al., 2020) to the order *Acholeplasmatales* and to the family *Anaeroplasmataceae*.

**Table:** 

**Description of *Candidatus* Avacholeplasma faecigallinarum sp. nov.**
*Candidatus* Avacholeplasma faecigallinarum (fae.ci.gal.li.na’rum. L. fem. n. *faex, faecis* excrement; L. fem. n. *gallina* hen; N.L. gen. n. *faecigallinarum* of hen faeces)
A bacterial species identified by metagenomic analyses. This species includes all bacteria with genomes that show ≥95% average nucleotide identity (ANI) to the type genome, which has been assigned the MAG ID 3263 and which is available via NCBI BioSample SAMN15816972. The GC content of the type genome is 29.88% and the genome length is 1.3 Mbp.
**Description of *Candidatus* Avacidaminococcus gen. nov.**
*Candidatus* Avacidaminococcus (Av.a.cid.a.mi.no.coc’cus. L. fem. n. *avis* bird; N.L. masc. n. *Acidaminococcus* a genus name; N.L. masc. n. *Avacidaminococcus* a genus related to the genus *Acidaminococcus* but distinct from it and found in poultry)
A bacterial genus identified by metagenomic analyses. The genus includes all bacteria with genomes that show ≥60% average amino acid identity (AAI) to the type genome from the type species *Candidatus* Avacidaminococcus intestinavium. This genus has been assigned by GTDB-Tk v1.3.0 working on GTDB Release 05-RS95 (Chaumeil et al., 2019; Parks et al., 2020) to the order *Acidaminococcales* and to the family *Acidaminococcaceae*.
**Description of *Candidatus* Avacidaminococcus intestinavium sp. nov.**
*Candidatus* Avacidaminococcus intestinavium (in.tes.tin.a’vi.um. L. neut. n. *intestinum* gut; L. fem. n. *avis* bird; N.L. gen. n. *intestinavium* of the gut of birds)
A bacterial species identified by metagenomic analyses. This species includes all bacteria with genomes that show ≥95% average nucleotide identity (ANI) to the type genome, which has been assigned the MAG ID CHK160-1198 and which is available via NCBI BioSample SAMN15816987. The GC content of the type genome is 37.45% and the genome length is 1.6 Mbp.
**Description of *Candidatus* Avamphibacillus gen. nov.**
*Candidatus* Avamphibacillus (Av.am.phi.ba.cil’lus. L. fem. n. *avis* bird; N.L. masc. n. *Amphibacillus* a genus name; N.L. masc. n. *Avamphibacillus* a genus related to the genus *Amphibacillus* but distinct from it and found in poultry)
A bacterial genus identified by metagenomic analyses. The genus includes all bacteria with genomes that show ≥60% average amino acid identity (AAI) to the type genome from the type species *Candidatus* Avamphibacillus intestinigallinarum. This genus has been assigned by GTDB-Tk v1.3.0 working on GTDB Release 05-RS95 (Chaumeil et al., 2019; Parks et al., 2020) to the order *Bacillales* and to the family *Amphibacillaceae*.
**Description of *Candidatus* Avamphibacillus intestinigallinarum sp. nov.**
*Candidatus* Avamphibacillus intestinigallinarum (in.tes.ti.ni.gal.li.na’rum. L. neut. n. *intestinum* gut; L. fem. n. *gallina* hen; N.L. gen. n. *intestinigallinarum* of the gut of the hens)
A bacterial species identified by metagenomic analyses. This species includes all bacteria with genomes that show ≥95% average nucleotide identity (ANI) to the type genome, which has been assigned the MAG ID CHK125-3527 and which is available via NCBI BioSample SAMN15816959. The GC content of the type genome is 36.77% and the genome length is 2.0 Mbp.
**Description of *Candidatus* Avanaerovorax gen. nov.**
*Candidatus* Avanaerovorax (Av.an.a.e.ro.vo’rax. L. fem. n. *avis* bird; N.L. masc. n. *Anaerovorax* a genus name; N.L. masc. n. *Avanaerovorax* a genus related to the genus *Anaerovorax* but distinct from it and found in poultry)
A bacterial genus identified by metagenomic analyses. The genus includes all bacteria with genomes that show ≥60% average amino acid identity (AAI) to the type genome from the type species *Candidatus* Avanaerovorax faecigallinarum. This genus has been assigned by GTDB-Tk v1.3.0 working on GTDB Release 05-RS95 (Chaumeil et al., 2019; Parks et al., 2020) to the order *Peptostreptococcales* and to the family *Anaerovoracaceae*.
**Description of *Candidatus* Avanaerovorax faecigallinarum sp. nov.**
*Candidatus* Avanaerovorax faecigallinarum (fae.ci.gal.li.na’rum. L. fem. n. *faex, faecis* excrement; L. fem. n. *gallina* hen; N.L. gen. n. *faecigallinarum* of hen faeces)
A bacterial species identified by metagenomic analyses. This species includes all bacteria with genomes that show ≥95% average nucleotide identity (ANI) to the type genome, which has been assigned the MAG ID Gambia13-1450 and which is available via NCBI BioSample SAMN15816994. The GC content of the type genome is 48.68% and the genome length is 1.8 Mbp.
**Description of *Candidatus* Aveggerthella gen. nov.**
*Candidatus* Aveggerthella (Av.eg.ger.thel’la. L. fem. n. *avis* bird; N.L. fem. n. *Eggerthella* a genus name; N.L. fem. n. *Aveggerthella* a genus related to the genus *Eggerthella* but distinct from it and found in poultry)
A bacterial genus identified by metagenomic analyses. The genus includes all bacteria with genomes that show ≥60% average amino acid identity (AAI) to the type genome from the type species *Candidatus* Avieggerthella excrementigallinarum. This genus has been assigned by GTDB-Tk v1.3.0 working on GTDB Release 05-RS95 (Chaumeil et al., 2019; Parks et al., 2020) to the order *Coriobacteriales* and to the family *Eggerthellaceae*.

**Table:** 

**Description of *Candidatus* Aveggerthella excrementigallinarum sp. nov.**
*Candidatus* Aveggerthella excrementigallinarum (ex.cre.men.ti.gal.li.na’rum. L. neut. n. *excrementum* excrement; L. fem. n. *gallina* hen; N.L. gen. n. *excrementigallinarum* of hen excrement)
A bacterial species identified by metagenomic analyses. This species includes all bacteria with genomes that show ≥95% average nucleotide identity (ANI) to the type genome, which has been assigned the MAG ID ChiGjej4B4-3573 and which is available via NCBI BioSample SAMN15816976. The GC content of the type genome is 65.93% and the genome length is 2.0 Mbp.
**Description of *Candidatus* Aveggerthella stercoripullorum sp. nov.**
*Candidatus* Aveggerthella stercoripullorum (ster.co.ri.pul.lo’rum. L. neut. n. *stercus* dung; L. masc. n. *pullus* a young chicken; N.L. gen. n. *stercoripullorum* of the faceces of young chickens)
A bacterial species identified by metagenomic analyses. This species includes all bacteria with genomes that show ≥95% average nucleotide identity (ANI) to the type genome, which has been assigned the MAG ID ChiGjej1B1-2707 and which is available via NCBI BioSample SAMN15816950. The GC content of the type genome is 61.50% and the genome length is 2.1 Mbp.
**Description of *Candidatus* Avelusimicrobium gen. nov.**
*Candidatus* Avelusimicrobium (Av.e.lu.si.mi.cro’bi.um. L. fem. n. *avis* bird; N.L. neut. n. *Elusimicrobium* a genus name; N.L. neut. n. *Avelusimicrobium* a genus related to the genus *Elusimicrobium* but distinct from it and found in poultry)
A bacterial genus identified by metagenomic analyses. The genus includes all bacteria with genomes that show ≥60% average amino acid identity (AAI) to the type genome from the type species *Candidatus* Avielusimicrobium excrementipullorum. This genus has been assigned by GTDB-Tk v1.3.0 working on GTDB Release 05-RS95 (Chaumeil et al., 2019; Parks et al., 2020) to the order *Elusimicrobiales* and to the family *Elusimicrobiaceae*.
**Description of *Candidatus* Avelusimicrobium excrementipullorum sp. nov.**
*Candidatus* Avelusimicrobium excrementipullorum (ex.cre.men.ti.pul.lo’rum. L. neut. n. *excrementum* excrement; L. masc. n. *pullus* a young chicken; N.L. gen. n. *excrementipullorum* of young chicken excrement)
A bacterial species identified by metagenomic analyses. This species includes all bacteria with genomes that show ≥95% average nucleotide identity (ANI) to the type genome, which has been assigned the MAG ID CHK136-6324 and which is available via NCBI BioSample SAMN15817002. The GC content of the type genome is 53.46% and the genome length is 1.3 Mbp.
**Description of *Candidatus* Avibacteroides gen. nov.**
*Candidatus* Avibacteroides (A.vi.bac.te.ro’i.des. L. fem. n. *avis* bird; N.L. masc. n. *Bacteroides* a genus name; N.L. masc. n. *Avibacteroides* a genus related to the genus *Bacteroides* but distinct from it and found in poultry)
A bacterial genus identified by metagenomic analyses. The genus includes all bacteria with genomes that show ≥60% average amino acid identity (AAI) to the type genome from the type species *Candidatus* Avibacteroides excrementipullorum. This genus has been assigned by GTDB-Tk v1.3.0 working on GTDB Release 05-RS95 (Chaumeil et al., 2019; Parks et al., 2020) to the order *Bacteroidales* and to the family *Bacteroidaceae*.
**Description of *Candidatus* Avibacteroides avistercoris sp. nov.**
*Candidatus* Avibacteroides avistercoris (a.vi.ster’co.ris. L. fem. n. *avis* bird; L. neut. n. *stercus* dung; N.L. gen. n. *avistercoris* of bird faeces)
A bacterial species identified by metagenomic analyses. This species includes all bacteria with genomes that show ≥95% average nucleotide identity (ANI) to the type genome, which has been assigned the MAG ID MalCec1-1739 and which is available via NCBI BioSample SAMN15816974. The GC content of the type genome is 53.14% and the genome length is 2.2 Mbp.
**Description of *Candidatus* Avibacteroides excrementipullorum sp. nov.**
*Candidatus* Avibacteroides excrementipullorum (ex.cre.men.ti.pul.lo’rum. L. neut. n. *excrementum* excrement; L. masc. n. *pullus* a young chicken; N.L. gen. n. *excrementipullorum* of young chicken excrement)
A bacterial species identified by metagenomic analyses. This species includes all bacteria with genomes that show ≥95% average nucleotide identity (ANI) to the type genome, which has been assigned the MAG ID ChiHjej12B11-16860 and which is available via NCBI BioSample SAMN15816958. The GC content of the type genome is 47.79% and the genome length is 2.2 Mbp.
**Description of *Candidatus* Avibacteroides faecavium sp. nov.**
*Candidatus* Avibacteroides faecavium (faec.a’vi.um. L. fem. n. *faex, faecis* excrement; L. fem. n. *avis* bird; N.L. gen. n. *faecavium* of bird faeces)
A bacterial species identified by metagenomic analyses. This species includes all bacteria with genomes that show ≥95% average nucleotide identity (ANI) to the type genome, which has been assigned the MAG ID 3702 and which is available via NCBI BioSample SAMN15816980. The GC content of the type genome is 55.42% and the genome length is 2.1 Mbp.

**Table:** 

**Description of *Candidatus* Avichristensenella gen. nov.**
*Candidatus* Avichristensenella (A.vi.chris.ten.sen.el’la. L. fem. n. *avis* bird; N.L. fem. n. *Christensenella* a genus name; N.L. fem. n. *Avichristensenella* a genus related to the genus *Christensenella* but distinct from it and found in poultry)
A bacterial genus identified by metagenomic analyses. The genus includes all bacteria with genomes that show ≥60% average amino acid identity (AAI) to the type genome from the type species *Candidatus* Avichristensenella intestinipullorum. This genus has been assigned by GTDB-Tk v1.3.0 working on GTDB Release 05-RS95 (Chaumeil et al., 2019; Parks et al., 2020) to the order *Christensenellales* and to the family *CAG-74*.
**Description of *Candidatus* Avichristensenella intestinipullorum sp. nov.**
*Candidatus* Avichristensenella intestinipullorum (in.tes.ti.ni.pul.lo’rum. L. neut. n. *intestinum* gut; L. masc. n. *pullus* a young chicken; N.L. gen. n. *intestinipullorum* of the gut of young chickens)
A bacterial species identified by metagenomic analyses. This species includes all bacteria with genomes that show ≥95% average nucleotide identity (ANI) to the type genome, which has been assigned the MAG ID ChiHile30-977 and which is available via NCBI BioSample SAMN15816947. The GC content of the type genome is 63.80% and the genome length is 2.3 Mbp.
**Description of *Candidatus* Avidehalobacter gen. nov.**
*Candidatus* Avidehalobacter (A.vi.de.ha.lo.bac’ter. L. fem. n. *avis* bird; N.L. masc. n. *Dehalobacter* a genus name; N.L. masc. n. *Avidehalobacter* a genus related to the genus *Dehalobacter* but distinct from it and found in poultry)
A bacterial genus identified by metagenomic analyses. The genus includes all bacteria with genomes that show ≥60% average amino acid identity (AAI) to the type genome from the type species *Candidatus* Avidehalobacter gallistercoris. This genus has been assigned by GTDB-Tk v1.3.0 working on GTDB Release 05-RS95 (Chaumeil et al., 2019; Parks et al., 2020) to the order *UBA4068* and to the family *UBA5755*.
**Description of *Candidatus* Avidehalobacter gallistercoris sp. nov.**
*Candidatus* Avidehalobacter gallistercoris (gal.li.ster’co.ris. L. masc. n *gallus* chicken; L. neut. n. *stercus* dung; N.L. gen. n. *gallistercoris* of chicken faeces)
A bacterial species identified by metagenomic analyses. This species includes all bacteria with genomes that show ≥95% average nucleotide identity (ANI) to the type genome, which has been assigned the MAG ID 2830 and which is available via NCBI BioSample SAMN15816981. The GC content of the type genome is 52.20% and the genome length is 1.4 Mbp.
**Description of *Candidatus* Avidesulfovibrio gen. nov.**
*Candidatus* Avidesulfovibrio (A.vi.de.sul.fo.vi’bri.o. L. fem. n. *avis* bird; N.L. masc. n. *Desulfovibrio* a genus name; N.L. masc. n. *Avidesulfovibrio* a genus related to the genus *Desulfovibrio* but distinct from it and found in poultry)
A bacterial genus identified by metagenomic analyses. The genus includes all bacteria with genomes that show ≥60% average amino acid identity (AAI) to the type genome from the type species *Candidatus* Avidesulfovibrio excrementigallinarum. This genus has been assigned by GTDB-Tk v1.3.0 working on GTDB Release 05-RS95 (Chaumeil et al., 2019; Parks et al., 2020) to the order *Desulfovibrionales* and to the family *Desulfovibrionaceae*.
**Description of *Candidatus* Avidesulfovibrio excrementigallinarum sp. nov.**
*Candidatus* Avidesulfovibrio excrementigallinarum (ex.cre.men.ti.gal.li.na’rum. L. neut. n. *excrementum* excrement; L. fem. n. *gallina* hen; N.L. gen. n. *excrementigallinarum* of hen excrement)
A bacterial species identified by metagenomic analyses. This species includes all bacteria with genomes that show ≥95% average nucleotide identity (ANI) to the type genome, which has been assigned the MAG ID ChiHcec4-2777 and which is available via NCBI BioSample SAMN15816982. The GC content of the type genome is 60.70% and the genome length is 2.2 Mbp.
**Description of *Candidatus* Avigastranaerophilus gen. nov.**
*Candidatus* Avigastranaerophilus (A.vi.gastr.an.a.e.ro’phi.lus. L. fem. n. *avis* bird; N.L. masc. n. *Gastranaerophilus* a genus name; N.L. masc. n. *Avigastranaerophilus* a genus related to the genus *Gastranaerophilus* but distinct from it and found in poultry)
A bacterial genus identified by metagenomic analyses. The genus includes all bacteria with genomes that show ≥60% average amino acid identity (AAI) to the type genome from the type species *Candidatus* Avigastranaerophilus faecigallinarum. This genus has been assigned by GTDB-Tk v1.3.0 working on GTDB Release 05-RS95 (Chaumeil et al., 2019; Parks et al., 2020) to the order *Gastranaerophilales* and to the family *Gastranaerophilaceae*.
**Description of *Candidatus* Avigastranaerophilus faecigallinarum sp. nov.**
*Candidatus* Avigastranaerophilus faecigallinarum (fae.ci.gal.li.na’rum. L. fem. n. *faex, faecis* excrement; L. fem. n. *gallina* hen; N.L. gen. n. *faecigallinarum* of hen faeces)
A bacterial species identified by metagenomic analyses. This species includes all bacteria with genomes that show ≥95% average nucleotide identity (ANI) to the type genome, which has been assigned the MAG ID 5572 and which is available via NCBI BioSample SAMN15816968. The GC content of the type genome is 29.33% and the genome length is 2.2 Mbp.
**Description of *Candidatus* Avilachnospira gen. nov.**
*Candidatus* Avilachnospira (A.vi.lach.no.spi’ra. L. fem. n. *avis* bird; N.L. fem. n. *Lachnospira* a genus name; N.L. fem. n. *Avilachnospira* a genus related to the genus *Lachnospira* but distinct from it and found in poultry)
A bacterial genus identified by metagenomic analyses. The genus includes all bacteria with genomes that show ≥60% average amino acid identity (AAI) to the type genome from the type species *Candidatus* Avilachnospira avistercoris. This genus has been assigned by GTDB-Tk v1.3.0 working on GTDB Release 05-RS95 (Chaumeil et al., 2019; Parks et al., 2020) to the order *Lachnospirales* and to the family *Lachnospiraceae*.

**Table:** 

**Description of *Candidatus* Avilachnospira avicola sp. nov.**
*Candidatus* Avilachnospira avicola (a.vi’co.la. L. fem. n. *avis* bird; L. suff. *-cola* inhabitant of; N.L. n. *avicola* inhabitant of birds)
A bacterial species identified by metagenomic analyses. This species includes all bacteria with genomes that show ≥95% average nucleotide identity (ANI) to the type genome, which has been assigned the MAG ID ChiHecec3B27-5021 and which is available via NCBI BioSample SAMN15816990. The GC content of the type genome is 49.15% and the genome length is 1.6 Mbp.
**Description of *Candidatus* Avilachnospira avistercoris sp. nov.**
*Candidatus* Avilachnospira avistercoris (a.vi.ster’co.ris. L. fem. n. *avis* bird; L. neut. n. *stercus* dung; N.L. gen. n. *avistercoris* of bird faeces)
A bacterial species identified by metagenomic analyses. This species includes all bacteria with genomes that show ≥95% average nucleotide identity (ANI) to the type genome, which has been assigned the MAG ID ChiGjej5B5-15814 and which is available via NCBI BioSample SAMN15816991. The GC content of the type genome is 50.02% and the genome length is 1.6 Mbp.
**Description of *Candidatus* Avimonoglobus gen. nov.**
*Candidatus* Avimonoglobus (A.vi.mo.no.glo’bus. L. fem. n. *avis* bird; N.L. masc. n. *Monoglobus* a genus name; N.L. masc. n. *Avimonoglobus* a genus related to the genus *Monoglobus* but distinct from it and found in poultry)
A bacterial genus identified by metagenomic analyses. The genus includes all bacteria with genomes that show ≥60% average amino acid identity (AAI) to the type genome from the type species *Candidatus* Avimonoglobus intestinipullorum. This genus has been assigned by GTDB-Tk v1.3.0 working on GTDB Release 05-RS95 (Chaumeil et al., 2019; Parks et al., 2020) to the order *Monoglobales_A* and to the family *UBA1381*.
**Description of *Candidatus* Avimonoglobus intestinipullorum sp. nov.**
*Candidatus* Avimonoglobus intestinipullorum (in.tes.ti.ni.pul.lo’rum. L. neut. n. *intestinum* gut; L. masc. n. *pullus* a young chicken; N.L. gen. n. *intestinipullorum* of the gut of young chickens)
A bacterial species identified by metagenomic analyses. This species includes all bacteria with genomes that show ≥95% average nucleotide identity (ANI) to the type genome, which has been assigned the MAG ID ChiSjej4B22-9803 and which is available via NCBI BioSample SAMN15816985. The GC content of the type genome is 51.95% and the genome length is 1.8 Mbp.
**Description of *Candidatus* Avimuribaculum gen. nov.**
*Candidatus* Avimuribaculum (A.vi.mu.ri.ba’cu.lum. L. fem. n. *avis* bird; N.L. neut. n. *Muribaculum* a genus name; N.L. neut. n. *Avimuribaculum* a genus related to the genus *Muribaculum* but distinct from it and found in poultry)
A bacterial genus identified by metagenomic analyses. The genus includes all bacteria with genomes that show ≥60% average amino acid identity (AAI) to the type genome from the type species *Candidatus* Avimuribaculum pullicola. This genus has been assigned by GTDB-Tk v1.3.0 working on GTDB Release 05-RS95 (Chaumeil et al., 2019; Parks et al., 2020) to the order *Bacteroidales* and to the family *Muribaculaceae*.
**Description of *Candidatus* Avimuribaculum pullicola sp. nov.**
*Candidatus* Avimuribaculum pullicola (pul.li’co.la. L. masc. n. *pullus* a young chicken; L. suff. *-cola* inhabitant of; N.L. n. *pullicola* an inhabitant of young chickens)
A bacterial species identified by metagenomic analyses. This species includes all bacteria with genomes that show ≥95% average nucleotide identity (ANI) to the type genome, which has been assigned the MAG ID ChiHecec3B27-9160 and which is available via NCBI BioSample SAMN15816969. The GC content of the type genome is 47.58% and the genome length is 2.2 Mbp.
**Description of *Candidatus* Avipropionibacterium gen. nov.**
*Candidatus* Avipropionibacterium (A.vi.pro.pi.o.ni.bac.te’ri.um. L. fem. n. *avis* bird; N.L. neut. n. *Propionibacterium* a genus name; N.L. neut. n. *Avipropionibacterium* a genus related to the genus *Propionibacterium* but distinct from it and found in poultry)
A bacterial genus identified by metagenomic analyses. The genus includes all bacteria with genomes that show ≥60% average amino acid identity (AAI) to the type genome from the type species *Candidatus* Avipropionibacterium avicola. This genus has been assigned by GTDB-Tk v1.3.0 working on GTDB Release 05-RS95 (Chaumeil et al., 2019; Parks et al., 2020) to the order *Propionibacteriales* and to the family *Propionibacteriaceae*.
**Description of *Candidatus* Avipropionibacterium avicola sp. nov.**
*Candidatus* Avipropionibacterium avicola (a.vi’co.la. L. fem. n. *avis* bird; L. suff. *-cola* inhabitant of; N.L. n. *avicola* inhabitant of birds)
A bacterial species identified by metagenomic analyses. This species includes all bacteria with genomes that show ≥95% average nucleotide identity (ANI) to the type genome, which has been assigned the MAG ID ChiGjej1B1-24693 and which is available via NCBI BioSample SAMN15816979. The GC content of the type genome is 69.14% and the genome length is 3.2 Mbp.

**Table:** 

**Description of *Candidatus* Avirikenella gen. nov.**
*Candidatus* Avirikenella (A.vi.ri.ke.nel’la. L. fem. n. *avis* bird; N.L. fem. n. *Rikenella* a genus name; N.L. fem. n. *Avirikenella* a genus related to the genus *Rikenella* but distinct from it and found in poultry)
A bacterial genus identified by metagenomic analyses. The genus includes all bacteria with genomes that show ≥60% average amino acid identity (AAI) to the type genome from the type species *Candidatus* Avirikenella pullistercoris. This genus has been assigned by GTDB-Tk v1.3.0 working on GTDB Release 05-RS95 (Chaumeil et al., 2019; Parks et al., 2020) to the order *Bacteroidales* and to the family *Rikenellaceae*.
**Description of *Candidatus* Avirikenella pullistercoris sp. nov.**
*Candidatus* Avirikenella pullistercoris (pul.li.ster’co.ris. L. masc. n. *pullus* a young chicken; L. neut. n. *stercus* dung; N.L. gen. n. *pullistercoris* of young chicken faeces)
A bacterial species identified by metagenomic analyses. This species includes all bacteria with genomes that show ≥95% average nucleotide identity (ANI) to the type genome, which has been assigned the MAG ID 9321 and which is available via NCBI BioSample SAMN15816960. The GC content of the type genome is 41.91% and the genome length is 1.9 Mbp.
**Description of *Candidatus* Avisuccinivibrio gen. nov.**
*Candidatus* Avisuccinivibrio (A.vi.suc.ci.ni.vi’bri.o. L. fem. n. *avis* bird; N.L. masc. n. *Succinivibrio* a genus name; N.L. masc. n. *Avisuccinivibrio* a genus related to the genus *Succinivibrio* but distinct from it and found in poultry)
A bacterial genus identified by metagenomic analyses. The genus includes all bacteria with genomes that show ≥60% average amino acid identity (AAI) to the type genome from the type species *Candidatus* Avisuccinivibrio stercorigallinarum. This genus has been assigned by GTDB-Tk v1.3.0 working on GTDB Release 05-RS95 (Chaumeil et al., 2019; Parks et al., 2020) to the order *Enterobacterales* and to the family *Succinivibrionaceae*.
**Description of *Candidatus* Avisuccinivibrio pullicola sp. nov.**
*Candidatus* Avisuccinivibrio pullicola (pul.li’co.la. L. masc. n. *pullus* a young chicken; L. suff. *-cola* inhabitant of; N.L. n. *pullicola* an inhabitant of young chickens)
A bacterial species identified by metagenomic analyses. This species includes all bacteria with genomes that show ≥95% average nucleotide identity (ANI) to the type genome, which has been assigned the MAG ID 3820 and which is available via NCBI BioSample SAMN15816999. The GC content of the type genome is 55.94% and the genome length is 2.4 Mbp.
**Description of *Candidatus* Avisuccinivibrio stercorigallinarum sp. nov.**
*Candidatus* Avisuccinivibrio stercorigallinarum (ster.co.ri.gal.li.na’rum. L. neut. n. *stercus* dung; L. fem. n. *gallina* hen; N.L. gen. n. *stercorigallinarum* of hen faeces)
A bacterial species identified by metagenomic analyses. This species includes all bacteria with genomes that show ≥95% average nucleotide identity (ANI) to the type genome, which has been assigned the MAG ID 17213 and which is available via NCBI BioSample SAMN15817000. The GC content of the type genome is 54.49% and the genome length is 2.4 Mbp.
**Description of *Candidatus* Avitreponema gen. nov.**
*Candidatus* Avitreponema (A.vi.tre.po.ne’ma. L. fem. n. *avis* bird; N.L. neut. n. *Treponema* a genus name; N.L. neut. n. *Avitreponema* a genus related to the genus *Treponema* but distinct from it and found in poultry)
A bacterial genus identified by metagenomic analyses. The genus includes all bacteria with genomes that show ≥60% average amino acid identity (AAI) to the type genome from the type species *Candidatus* Avitreponema avistercoris. This genus has been assigned by GTDB-Tk v1.3.0 working on GTDB Release 05-RS95 (Chaumeil et al., 2019; Parks et al., 2020) to the order *Treponematales* and to the family *Treponemataceae*.
**Description of *Candidatus* Avitreponema avistercoris sp. nov.**
*Candidatus* Avitreponema avistercoris (a.vi.ster’co.ris. L. fem. n. *avis* bird; L. neut. n. *stercus* dung; N.L. gen. n. *avistercoris* of bird faeces)
A bacterial species identified by metagenomic analyses. This species includes all bacteria with genomes that show ≥95% average nucleotide identity (ANI) to the type genome, which has been assigned the MAG ID B3-4054 and which is available via NCBI BioSample SAMN15816977. The GC content of the type genome is 55.36% and the genome length is 1.9 Mbp.
**Description of *Candidatus* Avoscillospira gen. nov.**
*Candidatus* Avoscillospira (Av.os.cil.lo.spi’ra. L. fem. n. *avis* bird; N.L. fem. n. *Oscillospira* a genus name; N.L. fem. n. *Avoscillospira* a genus related to the genus *Oscillospira* but distinct from it and found in poultry)
A bacterial genus identified by metagenomic analyses. The genus includes all bacteria with genomes that show ≥60% average amino acid identity (AAI) to the type genome from the type species *Candidatus* Avioscillospira stercorigallinarum. This genus has been assigned by GTDB-Tk v1.3.0 working on GTDB Release 05-RS95 (Chaumeil et al., 2019; Parks et al., 2020) to the order *Oscillospirales* and to the family *Oscillospiraceae*.

**Table:** 

**Description of *Candidatus* Avoscillospira avicola sp. nov.**
*Candidatus* Avoscillospira avicola (a.vi’co.la. L. fem. n. *avis* bird; L. suff. *-cola* inhabitant of; N.L. n. *avicola* inhabitant of birds)
A bacterial species identified by metagenomic analyses. This species includes all bacteria with genomes that show ≥95% average nucleotide identity (ANI) to the type genome, which has been assigned the MAG ID ChiBcec15-4380 and which is available via NCBI BioSample SAMN15816934. The GC content of the type genome is 61.76% and the genome length is 2.5 Mbp.
**Description of *Candidatus* Avoscillospira avistercoris sp. nov.**
*Candidatus* Avoscillospira avistercoris (a.vi.ster’co.ris. L. fem. n. *avis* bird; L. neut. n. *stercus* dung; N.L. gen. n. *avistercoris* of bird faeces)
A bacterial species identified by metagenomic analyses. This species includes all bacteria with genomes that show ≥95% average nucleotide identity (ANI) to the type genome, which has been assigned the MAG ID ChiBcec16-1751 and which is available via NCBI BioSample SAMN15816964. The GC content of the type genome is 58.00% and the genome length is 2.4 Mbp.
**Description of *Candidatus* Avoscillospira stercorigallinarum sp. nov.**
*Candidatus* Avoscillospira stercorigallinarum (ster.co.ri.gal.li.na’rum. L. neut. n. *stercus* dung; L. fem. n. *gallina* hen; N.L. gen. n. *stercorigallinarum* of hen faeces)
A bacterial species identified by metagenomic analyses. This species includes all bacteria with genomes that show ≥95% average nucleotide identity (ANI) to the type genome, which has been assigned the MAG ID ChiSjej2B20-13462 and which is available via NCBI BioSample SAMN15816948. The GC content of the type genome is 63.01% and the genome length is 2.2 Mbp.
**Description of *Candidatus* Avoscillospira stercoripullorum sp. nov.**
*Candidatus* Avoscillospira stercoripullorum (ster.co.ri.pul.lo’rum. L. neut. n. *stercus* dung; L. masc. n. *pullus* a young chicken; N.L. gen. n. *stercoripullorum* of the faceces of young chickens)
A bacterial species identified by metagenomic analyses. This species includes all bacteria with genomes that show ≥95% average nucleotide identity (ANI) to the type genome, which has been assigned the MAG ID ChiHjej9B8-7071 and which is available via NCBI BioSample SAMN15816951. The GC content of the type genome is 60.69% and the genome length is 1.9 Mbp.
**Description of *Candidatus* Bacteroides avicola sp. nov.**
*Candidatus* Bacteroides avicola (a.vi’co.la. L. fem. n. *avis* bird; L. suff. *-cola* inhabitant of; N.L. n. *avicola* inhabitant of birds)
A bacterial species identified by metagenomic analyses. This species includes all bacteria with genomes that show ≥95% average nucleotide identity (ANI) to the type genome, which has been assigned the MAG ID ChiHjej12B11-9795 and which is available via NCBI BioSample SAMN15816830. This is a new name for the alphanumeric GTDB species sp002160055. The GC content of the type genome is 50.12% and the genome length is 3.0 Mbp.
**Description of *Candidatus* Bacteroides intestinavium sp. nov.**
*Candidatus* Bacteroides intestinavium (in.tes.tin.a’vi.um. L. neut. n. *intestinum* gut; L. fem. n. *avis* bird; N.L. gen. n. *intestinavium* of the gut of birds)
A bacterial species identified by metagenomic analyses. This species includes all bacteria with genomes that show ≥95% average nucleotide identity (ANI) to the type genome, which has been assigned the MAG ID ChiHecec1B25-7008 and which is available via NCBI BioSample SAMN15816665. The GC content of the type genome is 54.17% and the genome length is 2.6 Mbp.
**Description of *Candidatus* Bacteroides intestinigallinarum sp. nov.**
*Candidatus* Bacteroides intestinigallinarum (in.tes.ti.ni.gal.li.na’rum. L. neut. n. *intestinum* gut; L. fem. n. *gallina* hen; N.L. gen. n. *intestinigallinarum* of the gut of the hens)
A bacterial species identified by metagenomic analyses. This species includes all bacteria with genomes that show ≥95% average nucleotide identity (ANI) to the type genome, which has been assigned the MAG ID 2926 and which is available via NCBI BioSample SAMN15816831. This is a new name for the alphanumeric GTDB species sp003463205. The GC content of the type genome is 41.82% and the genome length is 5.8 Mbp.
**Description of *Candidatus* Bacteroides intestinipullorum sp. nov.**
*Candidatus* Bacteroides intestinipullorum (in.tes.ti.ni.pul.lo’rum. L. neut. n. *intestinum* gut; L. masc. n. *pullus* a young chicken; N.L. gen. n. *intestinipullorum* of the gut of young chickens)
A bacterial species identified by metagenomic analyses. This species includes all bacteria with genomes that show ≥95% average nucleotide identity (ANI) to the type genome, which has been assigned the MAG ID B3-3758 and which is available via NCBI BioSample SAMN15816671. The GC content of the type genome is 53.99% and the genome length is 2.7 Mbp.

**Table:** 

**Description of *Candidatus* Bacteroides merdavium sp. nov.**
*Candidatus* Bacteroides merdavium (merd.a’vi.um. L. fem. n. *merda* faeces; L. fem. n. *avis* bird; N.L. gen. n. *merdavium* of bird faeces)
A bacterial species identified by metagenomic analyses. This species includes all bacteria with genomes that show ≥95% average nucleotide identity (ANI) to the type genome, which has been assigned the MAG ID CHK118-2852 and which is available via NCBI BioSample SAMN15816687. The GC content of the type genome is 49.76% and the genome length is 2.9 Mbp.
**Description of *Candidatus* Bacteroides merdigallinarum sp. nov.**
*Candidatus* Bacteroides merdigallinarum (mer.di.gal.li.na’rum. L. fem. n. *merda* faeces; L. fem. n. *gallina* hen; N.L. gen. n. *merdigallinarum* of hen faeces)
A bacterial species identified by metagenomic analyses. This species includes all bacteria with genomes that show ≥95% average nucleotide identity (ANI) to the type genome, which has been assigned the MAG ID ChiHjej9B8-1298 and which is available via NCBI BioSample SAMN15816694. The GC content of the type genome is 54.52% and the genome length is 2.7 Mbp.
**Description of *Candidatus* Bacteroides merdipullorum sp. nov.**
*Candidatus* Bacteroides merdipullorum (mer.di.pul.lo’rum. L. fem. n. *merda* faeces; L. masc. n. *pullus* a young chicken; N.L. gen. n. *merdipullorum* of the faeces of young chickens)
A bacterial species identified by metagenomic analyses. This species includes all bacteria with genomes that show ≥95% average nucleotide identity (ANI) to the type genome, which has been assigned the MAG ID ChiHjej12B11-24981 and which is available via NCBI BioSample SAMN15816699. The GC content of the type genome is 53.73% and the genome length is 2.5 Mbp.
**Description of *Candidatus* Bacteroides pullicola sp. nov.**
*Candidatus* Bacteroides pullicola (pul.li’co.la. L. masc. n. *pullus* a young chicken; L. suff. *-cola* inhabitant of; N.L. n. *pullicola* an inhabitant of young chickens)
A bacterial species identified by metagenomic analyses. This species includes all bacteria with genomes that show ≥95% average nucleotide identity (ANI) to the type genome, which has been assigned the MAG ID Gambia2-208 and which is available via NCBI BioSample SAMN15816704. The GC content of the type genome is 55.18% and the genome length is 2.7 Mbp.
**Description of *Candidatus* Bariatricus faecipullorum sp. nov.**
*Candidatus* Bariatricus faecipullorum (fae.ci.pul.lo’rum. L. fem. n. *faex, faecis* excrement; L. masc. n. *pullus* a young chicken; N.L. gen. n. *faecipullorum* of young chicken faeces)
A bacterial species identified by metagenomic analyses. This species includes all bacteria with genomes that show ≥95% average nucleotide identity (ANI) to the type genome, which has been assigned the MAG ID 9095 and which is available via NCBI BioSample SAMN15816662. The GC content of the type genome is 51.54% and the genome length is 2.5 Mbp.
**Description of *Candidatus* Barnesiella excrementavium sp. nov.**
*Candidatus* Barnesiella excrementavium (ex.cre.ment.a’vi.um. L. neut. n. *excrementum* excrement; L. fem. n. *avis* bird; N.L. gen. n. *excrementavium* of bird excrement)
A bacterial species identified by metagenomic analyses. This species includes all bacteria with genomes that show ≥95% average nucleotide identity (ANI) to the type genome, which has been assigned the MAG ID 4398 and which is available via NCBI BioSample SAMN15816714. The GC content of the type genome is 52.73% and the genome length is 2.7 Mbp.
**Description of *Candidatus* Barnesiella excrementigallinarum sp. nov.**
*Candidatus* Barnesiella excrementigallinarum (ex.cre.men.ti.gal.li.na’rum. L. neut. n. *excrementum* excrement; L. fem. n. *gallina* hen; N.L. gen. n. *excrementigallinarum* of hen excrement)
A bacterial species identified by metagenomic analyses. This species includes all bacteria with genomes that show ≥95% average nucleotide identity (ANI) to the type genome, which has been assigned the MAG ID CHK169-14362 and which is available via NCBI BioSample SAMN15816739. The GC content of the type genome is 47.04% and the genome length is 2.6 Mbp.
**Description of *Candidatus* Barnesiella excrementipullorum sp. nov.**
*Candidatus* Barnesiella excrementipullorum (ex.cre.men.ti.pul.lo’rum. L. neut. n. *excrementum* excrement; L. masc. n. *pullus* a young chicken; N.L. gen. n. *excrementipullorum* of young chicken excrement)
A bacterial species identified by metagenomic analyses. This species includes all bacteria with genomes that show ≥95% average nucleotide identity (ANI) to the type genome, which has been assigned the MAG ID ChiHjej12B11-16260 and which is available via NCBI BioSample SAMN15816862. This is a new name for the alphanumeric GTDB species sp900542255. The GC content of the type genome is 50.69% and the genome length is 2.1 Mbp.

**Table:** 

**Description of *Candidatus* Barnesiella merdigallinarum sp. nov.**
*Candidatus* Barnesiella merdigallinarum (mer.di.gal.li.na’rum. L. fem. n. *merda* faeces; L. fem. n. *gallina* hen; N.L. gen. n. *merdigallinarum* of hen faeces)
A bacterial species identified by metagenomic analyses. This species includes all bacteria with genomes that show ≥95% average nucleotide identity (ANI) to the type genome, which has been assigned the MAG ID CHK136-6590 and which is available via NCBI BioSample SAMN15816819. This is a new name for the alphanumeric GTDB species sp002159975. The GC content of the type genome is 54.15% and the genome length is 2.7 Mbp.
**Description of *Candidatus* Barnesiella merdipullorum sp. nov.**
*Candidatus* Barnesiella merdipullorum (mer.di.pul.lo’rum. L. fem. n. *merda* faeces; L. masc. n. *pullus* a young chicken; N.L. gen. n. *merdipullorum* of the faeces of young chickens)
A bacterial species identified by metagenomic analyses. This species includes all bacteria with genomes that show ≥95% average nucleotide identity (ANI) to the type genome, which has been assigned the MAG ID 5648 and which is available via NCBI BioSample SAMN15816849. This is a new name for the alphanumeric GTDB species sp002161555. The GC content of the type genome is 52.62% and the genome length is 2.6 Mbp.
**Description of *Candidatus* Bilophila faecipullorum sp. nov.**
*Candidatus* Bilophila faecipullorum (fae.ci.pul.lo’rum. L. fem. n. *faex, faecis* excrement; L. masc. n. *pullus* a young chicken; N.L. gen. n. *faecipullorum* of young chicken faeces)
A bacterial species identified by metagenomic analyses. This species includes all bacteria with genomes that show ≥95% average nucleotide identity (ANI) to the type genome, which has been assigned the MAG ID ChiSxjej5B17-1746 and which is available via NCBI BioSample SAMN15816754. The GC content of the type genome is 63.22% and the genome length is 2.8 Mbp.
**Description of *Candidatus* Blautia avicola sp. nov.**
*Candidatus* Blautia avicola (a.vi’co.la. L. fem. n. *avis* bird; L. suff. *-cola* inhabitant of; N.L. n. *avicola* inhabitant of birds)
A bacterial species identified by metagenomic analyses. This species includes all bacteria with genomes that show ≥95% average nucleotide identity (ANI) to the type genome, which has been assigned the MAG ID ChiBcec6-4105 and which is available via NCBI BioSample SAMN15816794. The GC content of the type genome is 46.40% and the genome length is 3.1 Mbp.
**Description of *Candidatus* Blautia avistercoris sp. nov.**
*Candidatus* Blautia avistercoris (a.vi.ster’co.ris. L. fem. n. *avis* bird; L. neut. n. *stercus* dung; N.L. gen. n. *avistercoris* of bird faeces)
A bacterial species identified by metagenomic analyses. This species includes all bacteria with genomes that show ≥95% average nucleotide identity (ANI) to the type genome, which has been assigned the MAG ID 5548 and which is available via NCBI BioSample SAMN15816924. This is a new name for the alphanumeric GTDB species sp002159835. Although GTDB has assigned this species to the genus it calls Blautia_A, this genus designation cannot be incorporated into a well-formed binomial, so in naming this species, we have used the current validly published name for the genus. The GC content of the type genome is 45.38% and the genome length is 2.5 Mbp.
**Description of *Candidatus* Blautia excrementigallinarum sp. nov.**
*Candidatus* Blautia excrementigallinarum (ex.cre.men.ti.gal.li.na’rum. L. neut. n. *excrementum* excrement; L. fem. n. *gallina* hen; N.L. gen. n. *excrementigallinarum* of hen excrement)
A bacterial species identified by metagenomic analyses. This species includes all bacteria with genomes that show ≥95% average nucleotide identity (ANI) to the type genome, which has been assigned the MAG ID ChiSjej6B24-370 and which is available via NCBI BioSample SAMN15816894. Although GTDB has assigned this species to the genus it calls Blautia_A, this genus designation cannot be incorporated into a well-formed binomial, so in naming this species, we have used the current validly published name for the genus. The GC content of the type genome is 49.44% and the genome length is 2.3 Mbp.
**Description of *Candidatus* Blautia excrementipullorum sp. nov.**
*Candidatus* Blautia excrementipullorum (ex.cre.men.ti.pul.lo’rum. L. neut. n. *excrementum* excrement; L. masc. n. *pullus* a young chicken; N.L. gen. n. *excrementipullorum* of young chicken excrement)
A bacterial species identified by metagenomic analyses. This species includes all bacteria with genomes that show ≥95% average nucleotide identity (ANI) to the type genome, which has been assigned the MAG ID CHK197-7439 and which is available via NCBI BioSample SAMN15816885. Although GTDB has assigned this species to the genus it calls Blautia_A, this genus designation cannot be incorporated into a well-formed binomial, so in naming this species, we have used the current validly published name for the genus. The GC content of the type genome is 46.79% and the genome length is 3.3 Mbp.

**Table:** 

**Description of *Candidatus* Blautia faecavium sp. nov.**
*Candidatus* Blautia faecavium (faec.a’vi.um. L. fem. n. *faex, faecis* excrement; L. fem. n. *avis* bird; N.L. gen. n. *faecavium* of bird faeces)
A bacterial species identified by metagenomic analyses. This species includes all bacteria with genomes that show ≥95% average nucleotide identity (ANI) to the type genome, which has been assigned the MAG ID ChiSjej1B19-5720 and which is available via NCBI BioSample SAMN15816886. Although GTDB has assigned this species to the genus it calls Blautia_A, this genus designation cannot be incorporated into a well-formed binomial, so in naming this species, we have used the current validly published name for the genus. The GC content of the type genome is 45.35% and the genome length is 3.5 Mbp.
**Description of *Candidatus* Blautia faecigallinarum sp. nov.**
*Candidatus* Blautia faecigallinarum (fae.ci.gal.li.na’rum. L. fem. n. *faex, faecis* excrement; L. fem. n. *gallina* hen; N.L. gen. n. *faecigallinarum* of hen faeces)
A bacterial species identified by metagenomic analyses. This species includes all bacteria with genomes that show ≥95% average nucleotide identity (ANI) to the type genome, which has been assigned the MAG ID 14324 and which is available via NCBI BioSample SAMN15816901. Although GTDB has assigned this species to the genus it calls Blautia_A, this genus designation cannot be incorporated into a well-formed binomial, so in naming this species, we have used the current validly published name for the genus. The GC content of the type genome is 48.52% and the genome length is 2.9 Mbp.
**Description of *Candidatus* Blautia faecipullorum sp. nov.**
*Candidatus* Blautia faecipullorum (fae.ci.pul.lo’rum. L. fem. n. *faex, faecis* excrement; L. masc. n. *pullus* a young chicken; N.L. gen. n. *faecipullorum* of young chicken faeces)
A bacterial species identified by metagenomic analyses. This species includes all bacteria with genomes that show ≥95% average nucleotide identity (ANI) to the type genome, which has been assigned the MAG ID ChiSxjej6B18-2004 and which is available via NCBI BioSample SAMN15816906. Although GTDB has assigned this species to the genus it calls Blautia_A, this genus designation cannot be incorporated into a well-formed binomial, so in naming this species, we have used the current validly published name for the genus. The GC content of the type genome is 48.18% and the genome length is 3.5 Mbp.
**Description of *Candidatus* Blautia gallistercoris sp. nov.**
*Candidatus* Blautia gallistercoris (gal.li.ster’co.ris. L. masc. n *gallus* chicken; L. neut. n. *stercus* dung; N.L. gen. n. *gallistercoris* of chicken faeces)
A bacterial species identified by metagenomic analyses. This species includes all bacteria with genomes that show ≥95% average nucleotide identity (ANI) to the type genome, which has been assigned the MAG ID ChiSjej1B19-8411 and which is available via NCBI BioSample SAMN15816925. This is a new name for the alphanumeric GTDB species sp900542045. Although GTDB has assigned this species to the genus it calls Blautia_A, this genus designation cannot be incorporated into a well-formed binomial, so in naming this species, we have used the current validly published name for the genus. The GC content of the type genome is 48.96% and the genome length is 2.8 Mbp.
**Description of *Candidatus* Blautia intestinavium sp. nov.**
*Candidatus* Blautia intestinavium (in.tes.tin.a’vi.um. L. neut. n. *intestinum* gut; L. fem. n. *avis* bird; N.L. gen. n. *intestinavium* of the gut of birds)
A bacterial species identified by metagenomic analyses. This species includes all bacteria with genomes that show ≥95% average nucleotide identity (ANI) to the type genome, which has been assigned the MAG ID CHK186-553 and which is available via NCBI BioSample SAMN15816890. Although GTDB has assigned this species to the genus it calls Blautia_A, this genus designation cannot be incorporated into a well-formed binomial, so in naming this species, we have used the current validly published name for the genus. The GC content of the type genome is 47.27% and the genome length is 2.8 Mbp.
**Description of *Candidatus* Blautia intestinigallinarum sp. nov.**
*Candidatus* Blautia intestinigallinarum (in.tes.ti.ni.gal.li.na’rum. L. neut. n. *intestinum* gut; L. fem. n. *gallina* hen; N.L. gen. n. *intestinigallinarum* of the gut of the hens)
A bacterial species identified by metagenomic analyses. This species includes all bacteria with genomes that show ≥95% average nucleotide identity (ANI) to the type genome, which has been assigned the MAG ID CHK186-9876 and which is available via NCBI BioSample SAMN15816891. Although GTDB has assigned this species to the genus it calls Blautia_A, this genus designation cannot be incorporated into a well-formed binomial, so in naming this species, we have used the current validly published name for the genus. The GC content of the type genome is 46.77% and the genome length is 2.7 Mbp.
**Description of *Candidatus* Blautia intestinipullorum sp. nov.**
*Candidatus* Blautia intestinipullorum (in.tes.ti.ni.pul.lo’rum. L. neut. n. *intestinum* gut; L. masc. n. *pullus* a young chicken; N.L. gen. n. *intestinipullorum* of the gut of young chickens)
A bacterial species identified by metagenomic analyses. This species includes all bacteria with genomes that show ≥95% average nucleotide identity (ANI) to the type genome, which has been assigned the MAG ID ChiW16-4312 and which is available via NCBI BioSample SAMN15816892. Although GTDB has assigned this species to the genus it calls Blautia_A, this genus designation cannot be incorporated into a well-formed binomial, so in naming this species, we have used the current validly published name for the genus. The GC content of the type genome is 47.05% and the genome length is 2.4 Mbp.

**Table:** 

**Description of *Candidatus* Blautia merdavium sp. nov.**
*Candidatus* Blautia merdavium (merd.a’vi.um. L. fem. n. *merda* faeces; L. fem. n. *avis* bird; N.L. gen. n. *merdavium* of bird faeces)
A bacterial species identified by metagenomic analyses. This species includes all bacteria with genomes that show ≥95% average nucleotide identity (ANI) to the type genome, which has been assigned the MAG ID ChiBcec2-3848 and which is available via NCBI BioSample SAMN15816633. The GC content of the type genome is 48.60% and the genome length is 3.3 Mbp.
**Description of *Candidatus* Blautia merdigallinarum sp. nov.**
*Candidatus* Blautia merdigallinarum (mer.di.gal.li.na’rum. L. fem. n. *merda* faeces; L. fem. n. *gallina* hen; N.L. gen. n. *merdigallinarum* of hen faeces)
A bacterial species identified by metagenomic analyses. This species includes all bacteria with genomes that show ≥95% average nucleotide identity (ANI) to the type genome, which has been assigned the MAG ID ChiSxjej6B18-287 and which is available via NCBI BioSample SAMN15816815. This is a new name for the alphanumeric GTDB species sp900543715. The GC content of the type genome is 45.18% and the genome length is 3.3 Mbp.
**Description of *Candidatus* Blautia merdipullorum sp. nov.**
*Candidatus* Blautia merdipullorum (mer.di.pul.lo’rum. L. fem. n. *merda* faeces; L. masc. n. *pullus* a young chicken; N.L. gen. n. *merdipullorum* of the faeces of young chickens)
A bacterial species identified by metagenomic analyses. This species includes all bacteria with genomes that show ≥95% average nucleotide identity (ANI) to the type genome, which has been assigned the MAG ID 17058 and which is available via NCBI BioSample SAMN15816655. The GC content of the type genome is 45.05% and the genome length is 3.2 Mbp.
**Description of *Candidatus* Blautia ornithocaccae sp. nov.**
*Candidatus* Blautia ornithocaccae (or.ni.tho.cac’cae. Gr. masc. or fem. n. *ornis, ornithos* bird Gr. fem. n. *kakke* faeces; N.L. gen. n. *ornithocaccae* of bird faeces)
A bacterial species identified by metagenomic analyses. This species includes all bacteria with genomes that show ≥95% average nucleotide identity (ANI) to the type genome, which has been assigned the MAG ID ChiBcec1-3711 and which is available via NCBI BioSample SAMN15816880. This is a new name for the alphanumeric GTDB species sp002161285. The GC content of the type genome is 44.79% and the genome length is 3.1 Mbp.
**Description of *Candidatus* Blautia pullicola sp. nov.**
*Candidatus* Blautia pullicola (pul.li’co.la. L. masc. n. *pullus* a young chicken; L. suff. *-cola* inhabitant of; N.L. n. *pullicola* an inhabitant of young chickens)
A bacterial species identified by metagenomic analyses. This species includes all bacteria with genomes that show ≥95% average nucleotide identity (ANI) to the type genome, which has been assigned the MAG ID 1068 and which is available via NCBI BioSample SAMN15816689. The GC content of the type genome is 45.62% and the genome length is 3.0 Mbp.
**Description of *Candidatus* Blautia pullistercoris sp. nov.**
*Candidatus* Blautia pullistercoris (pul.li.ster’co.ris. L. masc. n. *pullus* a young chicken; L. neut. n. *stercus* dung; N.L. gen. n. *pullistercoris* of young chicken faeces)
A bacterial species identified by metagenomic analyses. This species includes all bacteria with genomes that show ≥95% average nucleotide identity (ANI) to the type genome, which has been assigned the MAG ID ChiHjej12B11-1927 and which is available via NCBI BioSample SAMN15816618. The GC content of the type genome is 45.73% and the genome length is 3.2 Mbp.
**Description of *Candidatus* Blautia stercoravium sp. nov.**
*Candidatus* Blautia stercoravium (ster.cor.a’vi.um. L. neut. n. *stercus* dung; L. fem. n. *avis* bird; N.L. gen. n. *stercoravium* of bird faeces)
A bacterial species identified by metagenomic analyses. This species includes all bacteria with genomes that show ≥95% average nucleotide identity (ANI) to the type genome, which has been assigned the MAG ID 3268 and which is available via NCBI BioSample SAMN15816738. The GC content of the type genome is 44.17% and the genome length is 2.7 Mbp.
**Description of *Candidatus* Blautia stercorigallinarum sp. nov.**
*Candidatus* Blautia stercorigallinarum (ster.co.ri.gal.li.na’rum. L. neut. n. *stercus* dung; L. fem. n. *gallina* hen; N.L. gen. n. *stercorigallinarum* of hen faeces)
A bacterial species identified by metagenomic analyses. This species includes all bacteria with genomes that show ≥95% average nucleotide identity (ANI) to the type genome, which has been assigned the MAG ID CHK195-9823 and which is available via NCBI BioSample SAMN15816627. The GC content of the type genome is 45.83% and the genome length is 3.1 Mbp.

**Table:** 

**Description of *Candidatus* Blautia stercoripullorum sp. nov.**
*Candidatus* Blautia stercoripullorum (ster.co.ri.pul.lo’rum. L. neut. n. *stercus* dung; L. masc. n. *pullus* a young chicken; N.L. gen. n. *stercoripullorum* of the faceces of young chickens)
A bacterial species identified by metagenomic analyses. This species includes all bacteria with genomes that show ≥95% average nucleotide identity (ANI) to the type genome, which has been assigned the MAG ID ChiW19-6364 and which is available via NCBI BioSample SAMN15816793. The GC content of the type genome is 44.78% and the genome length is 3.1 Mbp.
**Description of *Candidatus* Borkfalkia avicola sp. nov.**
*Candidatus* Borkfalkia avicola (a.vi’co.la. L. fem. n. *avis* bird; L. suff. *-cola* inhabitant of; N.L. n. *avicola* inhabitant of birds)
A bacterial species identified by metagenomic analyses. This species includes all bacteria with genomes that show ≥95% average nucleotide identity (ANI) to the type genome, which has been assigned the MAG ID CHK192-19661 and which is available via NCBI BioSample SAMN15816606. The GC content of the type genome is 58.92% and the genome length is 1.7 Mbp.
**Description of *Candidatus* Borkfalkia avistercoris sp. nov.**
*Candidatus* Borkfalkia avistercoris (a.vi.ster’co.ris. L. fem. n. *avis* bird; L. neut. n. *stercus* dung; N.L. gen. n. *avistercoris* of bird faeces)
A bacterial species identified by metagenomic analyses. This species includes all bacteria with genomes that show ≥95% average nucleotide identity (ANI) to the type genome, which has been assigned the MAG ID CHK187-5294 and which is available via NCBI BioSample SAMN15816607. The GC content of the type genome is 53.99% and the genome length is 1.7 Mbp.
**Description of *Candidatus* Borkfalkia excrementavium sp. nov.**
*Candidatus* Borkfalkia excrementavium (ex.cre.ment.a’vi.um. L. neut. n. *excrementum* excrement; L. fem. n. *avis* bird; N.L. gen. n. *excrementavium* of bird excrement)
A bacterial species identified by metagenomic analyses. This species includes all bacteria with genomes that show ≥95% average nucleotide identity (ANI) to the type genome, which has been assigned the MAG ID CHK199-9574 and which is available via NCBI BioSample SAMN15816608. The GC content of the type genome is 52.76% and the genome length is 1.6 Mbp.
**Description of *Candidatus* Borkfalkia excrementigallinarum sp. nov.**
*Candidatus* Borkfalkia excrementigallinarum (ex.cre.men.ti.gal.li.na’rum. L. neut. n. *excrementum* excrement; L. fem. n. *gallina* hen; N.L. gen. n. *excrementigallinarum* of hen excrement)
A bacterial species identified by metagenomic analyses. This species includes all bacteria with genomes that show ≥95% average nucleotide identity (ANI) to the type genome, which has been assigned the MAG ID 1345 and which is available via NCBI BioSample SAMN15816609. The GC content of the type genome is 53.42% and the genome length is 1.9 Mbp.
**Description of *Candidatus* Borkfalkia excrementipullorum sp. nov.**
*Candidatus* Borkfalkia excrementipullorum (ex.cre.men.ti.pul.lo’rum. L. neut. n. *excrementum* excrement; L. masc. n. *pullus* a young chicken; N.L. gen. n. *excrementipullorum* of young chicken excrement)
A bacterial species identified by metagenomic analyses. This species includes all bacteria with genomes that show ≥95% average nucleotide identity (ANI) to the type genome, which has been assigned the MAG ID CHK192-2667 and which is available via NCBI BioSample SAMN15816611. The GC content of the type genome is 55.63% and the genome length is 1.6 Mbp.
**Description of *Candidatus* Borkfalkia faecavium sp. nov.**
*Candidatus* Borkfalkia faecavium (faec.a’vi.um. L. fem. n. *faex, faecis* excrement; L. fem. n. *avis* bird; N.L. gen. n. *faecavium* of bird faeces)
A bacterial species identified by metagenomic analyses. This species includes all bacteria with genomes that show ≥95% average nucleotide identity (ANI) to the type genome, which has been assigned the MAG ID 2189 and which is available via NCBI BioSample SAMN15816731. The GC content of the type genome is 58.98% and the genome length is 1.7 Mbp.
**Description of *Candidatus* Borkfalkia faecigallinarum sp. nov.**
*Candidatus* Borkfalkia faecigallinarum (fae.ci.gal.li.na’rum. L. fem. n. *faex, faecis* excrement; L. fem. n. *gallina* hen; N.L. gen. n. *faecigallinarum* of hen faeces)
A bacterial species identified by metagenomic analyses. This species includes all bacteria with genomes that show ≥95% average nucleotide identity (ANI) to the type genome, which has been assigned the MAG ID 26628 and which is available via NCBI BioSample SAMN15816617. The GC content of the type genome is 62.49% and the genome length is 1.6 Mbp.

**Table:** 

**Description of *Candidatus* Borkfalkia faecipullorum sp. nov.**
*Candidatus* Borkfalkia faecipullorum (fae.ci.pul.lo’rum. L. fem. n. *faex, faecis* excrement; L. masc. n. *pullus* a young chicken; N.L. gen. n. *faecipullorum* of young chicken faeces)
A bacterial species identified by metagenomic analyses. This species includes all bacteria with genomes that show ≥95% average nucleotide identity (ANI) to the type genome, which has been assigned the MAG ID 811 and which is available via NCBI BioSample SAMN15816621. The GC content of the type genome is 54.29% and the genome length is 1.9 Mbp.
**Description of *Candidatus* Borkfalkia stercoripullorum sp. nov.**
*Candidatus* Borkfalkia stercoripullorum (ster.co.ri.pul.lo’rum. L. neut. n. *stercus* dung; L. masc. n. *pullus* a young chicken; N.L. gen. n. *stercoripullorum* of the faceces of young chickens)
A bacterial species identified by metagenomic analyses. This species includes all bacteria with genomes that show ≥95% average nucleotide identity (ANI) to the type genome, which has been assigned the MAG ID CHK196-13738 and which is available via NCBI BioSample SAMN15816588. The GC content of the type genome is 55.22% and the genome length is 1.8 Mbp.
**Description of *Candidatus* Brachybacterium intestinipullorum sp. nov.**
*Candidatus* Brachybacterium intestinipullorum (in.tes.ti.ni.pul.lo’rum. L. neut. n. *intestinum* gut; L. masc. n. *pullus* a young chicken; N.L. gen. n. *intestinipullorum* of the gut of young chickens)
A bacterial species identified by metagenomic analyses. This species includes all bacteria with genomes that show ≥95% average nucleotide identity (ANI) to the type genome, which has been assigned the MAG ID CHK130-7132 and which is available via NCBI BioSample SAMN15816812. This is a new name for the alphanumeric GTDB species sp003711805. The GC content of the type genome is 72.59% and the genome length is 3.5 Mbp.
**Description of *Candidatus* Brachybacterium merdavium sp. nov.**
*Candidatus* Brachybacterium merdavium (merd.a’vi.um. L. fem. n. *merda* faeces; L. fem. n. *avis* bird; N.L. gen. n. *merdavium* of bird faeces)
A bacterial species identified by metagenomic analyses. This species includes all bacteria with genomes that show ≥95% average nucleotide identity (ANI) to the type genome, which has been assigned the MAG ID ChiHjej13B12-24818 and which is available via NCBI BioSample SAMN15816666. The GC content of the type genome is 70.22% and the genome length is 3.7 Mbp.
**Description of *Candidatus* Brachybacterium merdigallinarum sp. nov.**
*Candidatus* Brachybacterium merdigallinarum (mer.di.gal.li.na’rum. L. fem. n. *merda* faeces; L. fem. n. *gallina* hen; N.L. gen. n. *merdigallinarum* of hen faeces)
A bacterial species identified by metagenomic analyses. This species includes all bacteria with genomes that show ≥95% average nucleotide identity (ANI) to the type genome, which has been assigned the MAG ID ChiHjej13B12-7362 and which is available via NCBI BioSample SAMN15816717. The GC content of the type genome is 71.16% and the genome length is 3.1 Mbp.
**Description of *Candidatus* Brevibacterium intestinavium sp. nov.**
*Candidatus* Brevibacterium intestinavium (in.tes.tin.a’vi.um. L. neut. n. *intestinum* gut; L. fem. n. *avis* bird; N.L. gen. n. *intestinavium* of the gut of birds)
A bacterial species identified by metagenomic analyses. This species includes all bacteria with genomes that show ≥95% average nucleotide identity (ANI) to the type genome, which has been assigned the MAG ID 5295 and which is available via NCBI BioSample SAMN15816668. The GC content of the type genome is 66.76% and the genome length is 3.2 Mbp.
**Description of *Candidatus* Brevibacterium intestinigallinarum sp. nov.**
*Candidatus* Brevibacterium intestinigallinarum (in.tes.ti.ni.gal.li.na’rum. L. neut. n. *intestinum* gut; L. fem. n. *gallina* hen; N.L. gen. n. *intestinigallinarum* of the gut of the hens)
A bacterial species identified by metagenomic analyses. This species includes all bacteria with genomes that show ≥95% average nucleotide identity (ANI) to the type genome, which has been assigned the MAG ID CHK132-2174 and which is available via NCBI BioSample SAMN15816673. The GC content of the type genome is 70.54% and the genome length is 2.7 Mbp.
**Description of *Candidatus* Butyricicoccus avicola sp. nov.**
*Candidatus* Butyricicoccus avicola (a.vi’co.la. L. fem. n. *avis* bird; L. suff. *-cola* inhabitant of; N.L. n. *avicola* inhabitant of birds)
A bacterial species identified by metagenomic analyses. This species includes all bacteria with genomes that show ≥95% average nucleotide identity (ANI) to the type genome, which has been assigned the MAG ID ChiSjej6B24-14740 and which is available via NCBI BioSample SAMN15816587. The GC content of the type genome is 59.67% and the genome length is 2.0 Mbp.

**Table:** 

**Description of *Candidatus* Borkfalkia faecipullorum sp. nov.**
*Candidatus* Borkfalkia faecipullorum (fae.ci.pul.lo’rum. L. fem. n. *faex, faecis* excrement; L. masc. n. *pullus* a young chicken; N.L. gen. n. *faecipullorum* of young chicken faeces)
A bacterial species identified by metagenomic analyses. This species includes all bacteria with genomes that show ≥95% average nucleotide identity (ANI) to the type genome, which has been assigned the MAG ID 811 and which is available via NCBI BioSample SAMN15816621. The GC content of the type genome is 54.29% and the genome length is 1.9 Mbp.
**Description of *Candidatus* Borkfalkia stercoripullorum sp. nov.**
*Candidatus* Borkfalkia stercoripullorum (ster.co.ri.pul.lo’rum. L. neut. n. *stercus* dung; L. masc. n. *pullus* a young chicken; N.L. gen. n. *stercoripullorum* of the faceces of young chickens)
A bacterial species identified by metagenomic analyses. This species includes all bacteria with genomes that show ≥95% average nucleotide identity (ANI) to the type genome, which has been assigned the MAG ID CHK196-13738 and which is available via NCBI BioSample SAMN15816588. The GC content of the type genome is 55.22% and the genome length is 1.8 Mbp.
**Description of *Candidatus* Brachybacterium intestinipullorum sp. nov.**
*Candidatus* Brachybacterium intestinipullorum (in.tes.ti.ni.pul.lo’rum. L. neut. n. *intestinum* gut; L. masc. n. *pullus* a young chicken; N.L. gen. n. *intestinipullorum* of the gut of young chickens)
A bacterial species identified by metagenomic analyses. This species includes all bacteria with genomes that show ≥95% average nucleotide identity (ANI) to the type genome, which has been assigned the MAG ID CHK130-7132 and which is available via NCBI BioSample SAMN15816812. This is a new name for the alphanumeric GTDB species sp003711805. The GC content of the type genome is 72.59% and the genome length is 3.5 Mbp.
**Description of *Candidatus* Brachybacterium merdavium sp. nov.**
*Candidatus* Brachybacterium merdavium (merd.a’vi.um. L. fem. n. *merda* faeces; L. fem. n. *avis* bird; N.L. gen. n. *merdavium* of bird faeces)
A bacterial species identified by metagenomic analyses. This species includes all bacteria with genomes that show ≥95% average nucleotide identity (ANI) to the type genome, which has been assigned the MAG ID ChiHjej13B12-24818 and which is available via NCBI BioSample SAMN15816666. The GC content of the type genome is 70.22% and the genome length is 3.7 Mbp.
**Description of *Candidatus* Brachybacterium merdigallinarum sp. nov.**
*Candidatus* Brachybacterium merdigallinarum (mer.di.gal.li.na’rum. L. fem. n. *merda* faeces; L. fem. n. *gallina* hen; N.L. gen. n. *merdigallinarum* of hen faeces)
A bacterial species identified by metagenomic analyses. This species includes all bacteria with genomes that show ≥95% average nucleotide identity (ANI) to the type genome, which has been assigned the MAG ID ChiHjej13B12-7362 and which is available via NCBI BioSample SAMN15816717. The GC content of the type genome is 71.16% and the genome length is 3.1 Mbp.
**Description of *Candidatus* Brevibacterium intestinavium sp. nov.**
*Candidatus* Brevibacterium intestinavium (in.tes.tin.a’vi.um. L. neut. n. *intestinum* gut; L. fem. n. *avis* bird; N.L. gen. n. *intestinavium* of the gut of birds)
A bacterial species identified by metagenomic analyses. This species includes all bacteria with genomes that show ≥95% average nucleotide identity (ANI) to the type genome, which has been assigned the MAG ID 5295 and which is available via NCBI BioSample SAMN15816668. The GC content of the type genome is 66.76% and the genome length is 3.2 Mbp.
**Description of *Candidatus* Brevibacterium intestinigallinarum sp. nov.**
*Candidatus* Brevibacterium intestinigallinarum (in.tes.ti.ni.gal.li.na’rum. L. neut. n. *intestinum* gut; L. fem. n. *gallina* hen; N.L. gen. n. *intestinigallinarum* of the gut of the hens)
A bacterial species identified by metagenomic analyses. This species includes all bacteria with genomes that show ≥95% average nucleotide identity (ANI) to the type genome, which has been assigned the MAG ID CHK132-2174 and which is available via NCBI BioSample SAMN15816673. The GC content of the type genome is 70.54% and the genome length is 2.7 Mbp.
**Description of *Candidatus* Butyricicoccus avicola sp. nov.**
*Candidatus* Butyricicoccus avicola (a.vi’co.la. L. fem. n. *avis* bird; L. suff. *-cola* inhabitant of; N.L. n. *avicola* inhabitant of birds)
A bacterial species identified by metagenomic analyses. This species includes all bacteria with genomes that show ≥95% average nucleotide identity (ANI) to the type genome, which has been assigned the MAG ID ChiSjej6B24-14740 and which is available via NCBI BioSample SAMN15816587. The GC content of the type genome is 59.67% and the genome length is 2.0 Mbp.

**Table:** 

**Description of *Candidatus* Caccocola faecigallinarum sp. nov.**
*Candidatus* Caccocola faecigallinarum (fae.ci.gal.li.na’rum. L. fem. n. *faex, faecis* excrement; L. fem. n. *gallina* hen; N.L. gen. n. *faecigallinarum* of hen faeces)
A bacterial species identified by metagenomic analyses. This species includes all bacteria with genomes that show ≥95% average nucleotide identity (ANI) to the type genome, which has been assigned the MAG ID ChiHjej11B10-3130 and which is available via NCBI BioSample SAMN15817215. This is a new name for the alphanumeric GTDB species sp900544635. The GC content of the type genome is 60.31% and the genome length is 2.3 Mbp.
**Description of *Candidatus* Caccocola faecipullorum sp. nov.**
*Candidatus* Caccocola faecipullorum (fae.ci.pul.lo’rum. L. fem. n. *faex, faecis* excrement; L. masc. n. *pullus* a young chicken; N.L. gen. n. *faecipullorum* of young chicken faeces)
A bacterial species identified by metagenomic analyses. This species includes all bacteria with genomes that show ≥95% average nucleotide identity (ANI) to the type genome, which has been assigned the MAG ID ChiHcec3-7374 and which is available via NCBI BioSample SAMN15817103. The GC content of the type genome is 59.08% and the genome length is 2.2 Mbp.
**Description of *Candidatus* Caccomonas gen. nov.**
*Candidatus* Caccomonas (Cac.co.mo’nas. Gr. fem. n. *kakke* dung; L. fem. n. *monas* a monad; N.L. fem. n. *Caccomonas* a microbe associated with faeces)
A bacterial genus identified by metagenomic analyses. The genus includes all bacteria with genomes that show ≥60% average amino acid identity (AAI) to the type genome from the type species *Candidatus* Caccomonas pullistercoris. This is a name for the alphanumeric GTDB genus CAG-617. This genus has been assigned by GTDB-Tk v1.3.0 working on GTDB Release 05-RS95 (Chaumeil et al., 2019; Parks et al., 2020) to the order *Bacteroidales* and to the family *Bacteroidaceae*.
**Description of *Candidatus* Caccomonas pullistercoris sp. nov.**
*Candidatus* Caccomonas pullistercoris (pul.li.ster’co.ris. L. masc. n. *pullus* a young chicken; L. neut. n. *stercus* dung; N.L. gen. n. *pullistercoris* of young chicken faeces)
A bacterial species identified by metagenomic analyses. This species includes all bacteria with genomes that show ≥95% average nucleotide identity (ANI) to the type genome, which has been assigned the MAG ID 5345 and which is available via NCBI BioSample SAMN15817152. The GC content of the type genome is 59.45% and the genome length is 2.2 Mbp.
**Description of *Candidatus* Caccomorpha gen. nov.**
*Candidatus* Caccomorpha (Cac.co.mor’pha. Gr. fem. n. *kakke* dung; Gr. fem. n. *morphe* a form, shape; N.L. fem. n. *Caccomorpha* a microbe associated with faeces)
A bacterial genus identified by metagenomic analyses. The genus includes all bacteria with genomes that show ≥60% average amino acid identity (AAI) to the type genome from the type species *Candidatus* Caccomorpha excrementavium. This is a name for the alphanumeric GTDB genus SZUA-448. This genus has been assigned by GTDB-Tk v1.3.0 working on GTDB Release 05-RS95 (Chaumeil et al., 2019; Parks et al., 2020) to the order *Lachnospirales* and to the family *Lachnospiraceae*.
**Description of *Candidatus* Caccomorpha excrementavium sp. nov.**
*Candidatus* Caccomorpha excrementavium (ex.cre.ment.a’vi.um. L. neut. n. *excrementum* excrement; L. fem. n. *avis* bird; N.L. gen. n. *excrementavium* of bird excrement)
A bacterial species identified by metagenomic analyses. This species includes all bacteria with genomes that show ≥95% average nucleotide identity (ANI) to the type genome, which has been assigned the MAG ID 1999 and which is available via NCBI BioSample SAMN15817145. The GC content of the type genome is 50.75% and the genome length is 2.7 Mbp.
**Description of *Candidatus* Caccoplasma gen. nov.**
*Candidatus* Caccoplasma (Cac.co.plas’ma. Gr. fem. n. *kakke* dung; Gr. neut. n. *plasma* a form; N.L. neut. n. *Caccoplasma* a microbe associated with faeces)
A bacterial genus identified by metagenomic analyses. The genus includes all bacteria with genomes that show ≥60% average amino acid identity (AAI) to the type genome from the type species *Candidatus* Caccoplasma merdavium. This is a name for the alphanumeric GTDB genus UBA11471. This genus has been assigned by GTDB-Tk v1.3.0 working on GTDB Release 05-RS95 (Chaumeil et al., 2019; Parks et al., 2020) to the order *Bacteroidales* and to the family UBA11471.
**Description of *Candidatus* Caccoplasma intestinavium sp. nov.**
*Candidatus* Caccoplasma intestinavium (in.tes.tin.a’vi.um. L. neut. n. *intestinum* gut; L. fem. n. *avis* bird; N.L. gen. n. *intestinavium* of the gut of birds)
A bacterial species identified by metagenomic analyses. This species includes all bacteria with genomes that show ≥95% average nucleotide identity (ANI) to the type genome, which has been assigned the MAG ID 21143 and which is available via NCBI BioSample SAMN15817187. This is a new name for the alphanumeric GTDB species sp000434215. The GC content of the type genome is 45.83% and the genome length is 2.4 Mbp.

**Table:** 

**Description of *Candidatus* Caccoplasma merdavium sp. nov.**
*Candidatus* Caccoplasma merdavium (merd.a’vi.um. L. fem. n. *merda* faeces; L. fem. n. *avis* bird; N.L. gen. n. *merdavium* of bird faeces)
A bacterial species identified by metagenomic analyses. This species includes all bacteria with genomes that show ≥95% average nucleotide identity (ANI) to the type genome, which has been assigned the MAG ID 6821 and which is available via NCBI BioSample SAMN15817190. This is a new name for the alphanumeric GTDB species sp900542765. The GC content of the type genome is 52.79% and the genome length is 2.4 Mbp.
**Description of *Candidatus* Caccoplasma merdipullorum sp. nov.**
*Candidatus* Caccoplasma merdipullorum (mer.di.pul.lo’rum. L. fem. n. *merda* faeces; L. masc. n. *pullus* a young chicken; N.L. gen. n. *merdipullorum* of the faeces of young chickens)
A bacterial species identified by metagenomic analyses. This species includes all bacteria with genomes that show ≥95% average nucleotide identity (ANI) to the type genome, which has been assigned the MAG ID G3-4614 and which is available via NCBI BioSample SAMN15817135. The GC content of the type genome is 46.70% and the genome length is 2.0 Mbp.
**Description of *Candidatus* Caccopulliclostridium gen. nov.**
*Candidatus* Caccopulliclostridium (Cac.co.pul.li.clos.tri’di.um. Gr. fem. n. *kakke* faeces; L. masc. n. *pullus* a young chicken; N.L. neut. n. *Clostridium* a genus name; N.L. neut. n. *Caccopulliclostridium* a genus related to the genus *Clostridium* but distinct from it and found in poultry faeces)
A bacterial genus identified by metagenomic analyses. The genus includes all bacteria with genomes that show ≥60% average amino acid identity (AAI) to the type genome from the type species *Candidatus* Caccopulliclostridium gallistercoris. This genus was identified but not named by Glendinning et al. (2020). This genus has been assigned by GTDB-Tk v1.3.0 working on GTDB Release 05-RS95 (Chaumeil et al., 2019; Parks et al., 2020) to the order *4C28d-15* and to the family *UBA1242*.
**Description of *Candidatus* Caccopulliclostridium gallistercoris sp. nov.**
*Candidatus* Caccopulliclostridium gallistercoris (gal.li.ster’co.ris. L. masc. n *gallus* chicken; L. neut. n. *stercus* dung; N.L. gen. n. *gallistercoris* of chicken faeces)
A bacterial species identified by metagenomic analyses. This species includes all bacteria with genomes that show ≥95% average nucleotide identity (ANI) to the type genome, which has been assigned the MAG ID CHK186-9395 and which is available via NCBI BioSample SAMN15816943. The GC content of the type genome is 35.40% and the genome length is 1.0 Mbp.
**Description of *Candidatus* Caccosoma gen. nov.**
*Candidatus* Caccosoma (Cac.co.so’ma. Gr. fem. n. *kakke* dung; Gr. neut. n. *soma* a body; N.L. neut. n. *Caccosoma* a microbe associated with faeces)
A bacterial genus identified by metagenomic analyses. The genus includes all bacteria with genomes that show ≥60% average amino acid identity (AAI) to the type genome from the type species *Candidatus* Caccosoma faecigallinarum. This is a name for the alphanumeric GTDB genus CAG-631. This genus has been assigned by GTDB-Tk v1.3.0 working on GTDB Release 05-RS95 (Chaumeil et al., 2019; Parks et al., 2020) to the order *RFN20* and to the family *CAG-631*.
**Description of *Candidatus* Caccosoma faecigallinarum sp. nov.**
*Candidatus* Caccosoma faecigallinarum (fae.ci.gal.li.na’rum. L. fem. n. *faex, faecis* excrement; L. fem. n. *gallina* hen; N.L. gen. n. *faecigallinarum* of hen faeces)
A bacterial species identified by metagenomic analyses. This species includes all bacteria with genomes that show ≥95% average nucleotide identity (ANI) to the type genome, which has been assigned the MAG ID 14508 and which is available via NCBI BioSample SAMN15817185. This is a new name for the alphanumeric GTDB species sp000433015. The GC content of the type genome is 30.38% and the genome length is 1.3 Mbp.
**Description of *Candidatus* Caccousia gen. nov.**
*Candidatus* Caccousia (Cacc.ou’si.a. Gr. fem. n. *kakke* dung; Gr. fem. n. *ousia* an essence; N.L. fem. n. *Caccousia* a microbe associated with faeces)
A bacterial genus identified by metagenomic analyses. The genus includes all bacteria with genomes that show ≥60% average amino acid identity (AAI) to the type genome from the type species *Candidatus* Caccousia avicola. This is a name for the alphanumeric GTDB genus An200. This genus has been assigned by GTDB-Tk v1.3.0 working on GTDB Release 05-RS95 (Chaumeil et al., 2019; Parks et al., 2020) to the order *Oscillospirales* and to the family *Acutalibacteraceae*.
**Description of *Candidatus* Caccousia avicola sp. nov.**
*Candidatus* Caccousia avicola (a.vi’co.la. L. fem. n. *avis* bird; L. suff. *-cola* inhabitant of; N.L. n. *avicola* inhabitant of birds)
A bacterial species identified by metagenomic analyses. This species includes all bacteria with genomes that show ≥95% average nucleotide identity (ANI) to the type genome, which has been assigned the MAG ID ChiSxjej1B13-7958 and which is available via NCBI BioSample SAMN15817070. The GC content of the type genome is 58.28% and the genome length is 2.2 Mbp.

**Table:** 

**Description of *Candidatus* Caccousia avistercoris sp. nov.**
*Candidatus* Caccousia avistercoris (a.vi.ster’co.ris. L. fem. n. *avis* bird; L. neut. n. *stercus* dung; N.L. gen. n. *avistercoris* of bird faeces)
A bacterial species identified by metagenomic analyses. This species includes all bacteria with genomes that show ≥95% average nucleotide identity (ANI) to the type genome, which has been assigned the MAG ID 3024 and which is available via NCBI BioSample SAMN15817047. The GC content of the type genome is 60.88% and the genome length is 2.6 Mbp.
**Description of *Candidatus* Caccousia stercoris sp. nov.**
*Candidatus* Caccousia stercoris (ster’co.ris. L. gen. n. *stercoris* of dung, excrement)
A bacterial species identified by metagenomic analyses. This species includes all bacteria with genomes that show ≥95% average nucleotide identity (ANI) to the type genome, which has been assigned the MAG ID 6086 and which is available via NCBI BioSample SAMN15817184. This is a new name for the alphanumeric GTDB species sp002160025. The GC content of the type genome is 56.84% and the genome length is 2.3 Mbp.
**Description of *Candidatus* Caccovicinus gen. nov.**
*Candidatus* Caccovicinus (Cac.co.vi.ci’nus. Gr. fem. n. *kakke* dung; L. masc. n. *vicinus* a neighbour; N.L. masc. n. *Caccovicinus* a microbe associated with faeces)
A bacterial genus identified by metagenomic analyses. The genus includes all bacteria with genomes that show ≥60% average amino acid identity (AAI) to the type genome from the type species *Candidatus* Caccovicinus merdipullorum. This is a name for the alphanumeric GTDB genus UMGS1370. This genus has been assigned by GTDB-Tk v1.3.0 working on GTDB Release 05-RS95 (Chaumeil et al., 2019; Parks et al., 2020) to the order *Lachnospirales* and to the family *Lachnospiraceae*.
**Description of *Candidatus* Caccovicinus merdipullorum sp. nov.**
*Candidatus* Caccovicinus merdipullorum (mer.di.pul.lo’rum. L. fem. n. *merda* faeces; L. masc. n. *pullus* a young chicken; N.L. gen. n. *merdipullorum* of the faeces of young chickens)
A bacterial species identified by metagenomic analyses. This species includes all bacteria with genomes that show ≥95% average nucleotide identity (ANI) to the type genome, which has been assigned the MAG ID CHK198_11255 and which is available via NCBI BioSample SAMN15817041. The GC content of the type genome is 50.44% and the genome length is 3.0 Mbp.
**Description of *Candidatus* Caccovivens gen. nov.**
*Candidatus* Caccovivens (Cac.co.vi’vens. Gr. fem. n. *kakke* dung; N.L. pres. part. *vivens* living; N.L. fem. n. *Caccovivens* a microbe associated with faeces)
A bacterial genus identified by metagenomic analyses. The genus includes all bacteria with genomes that show ≥60% average amino acid identity (AAI) to the type genome from the type species *Candidatus* Caccovivens faecavium. This is a name for the alphanumeric GTDB genus UBA11517. This genus has been assigned by GTDB-Tk v1.3.0 working on GTDB Release 05-RS95 (Chaumeil et al., 2019; Parks et al., 2020) to the order *Christensenellales* and to the family *UBA1242*.
**Description of *Candidatus* Caccovivens faecavium sp. nov.**
*Candidatus* Caccovivens faecavium (faec.a’vi.um. L. fem. n. *faex, faecis* excrement; L. fem. n. *avis* bird; N.L. gen. n. *faecavium* of bird faeces)
A bacterial species identified by metagenomic analyses. This species includes all bacteria with genomes that show ≥95% average nucleotide identity (ANI) to the type genome, which has been assigned the MAG ID ChiW6-1002 and which is available via NCBI BioSample SAMN15817119. The GC content of the type genome is 34.22% and the genome length is 1.1 Mbp.
**Description of *Candidatus* Cellulosilyticum pullistercoris sp. nov.**
*Candidatus* Cellulosilyticum pullistercoris (pul.li.ster’co.ris. L. masc. n. *pullus* a young chicken; L. neut. n. *stercus* dung; N.L. gen. n. *pullistercoris* of young chicken faeces)
A bacterial species identified by metagenomic analyses. This species includes all bacteria with genomes that show ≥95% average nucleotide identity (ANI) to the type genome, which has been assigned the MAG ID B5-657 and which is available via NCBI BioSample SAMN15816752. The GC content of the type genome is 33.78% and the genome length is 2.3 Mbp.
**Description of *Candidatus* Choladocola gen. nov.**
*Candidatus* Choladocola (Cho.la.do’co.la. Gr. fem. n. *cholas* guts; L. suff. *-cola* inhabitant of; N.L. fem. n. *Choladocola* a microbe associated with the intestines)
A bacterial genus identified by metagenomic analyses. The genus includes all bacteria with genomes that show ≥60% average amino acid identity (AAI) to the type genome from the type species *Candidatus* Choladocola avistercoris. This is a name for the alphanumeric GTDB genus UBA7182. This genus has been assigned by GTDB-Tk v1.3.0 working on GTDB Release 05-RS95 (Chaumeil et al., 2019; Parks et al., 2020) to the order *Lachnospirales* and to the family *Lachnospiraceae*.

**Table:** 

**Description of *Candidatus* Choladocola avistercoris sp. nov.**
*Candidatus* Choladocola avistercoris (a.vi.ster’co.ris. L. fem. n. *avis* bird; L. neut. n. *stercus* dung; N.L. gen. n. *avistercoris* of bird faeces)
A bacterial species identified by metagenomic analyses. This species includes all bacteria with genomes that show ≥95% average nucleotide identity (ANI) to the type genome, which has been assigned the MAG ID ChiBcec18-1958 and which is available via NCBI BioSample SAMN15817180. This is a new name for the alphanumeric GTDB species sp002160135. The GC content of the type genome is 50.79% and the genome length is 2.4 Mbp.
**Description of *Candidatus* Choladousia gen. nov.**
*Candidatus* Choladousia (Cho.lad.ou’si.a. Gr. fem. n. *cholas* guts; Gr. fem. n. *ousia* an essence; N.L. fem. n. *Choladousia* a microbe associated with the intestines)
A bacterial genus identified by metagenomic analyses. The genus includes all bacteria with genomes that show ≥60% average amino acid identity (AAI) to the type genome from the type species *Candidatus* Choladousia intestinavium. This is a name for the alphanumeric GTDB genus UBA7160. This genus has been assigned by GTDB-Tk v1.3.0 working on GTDB Release 05-RS95 (Chaumeil et al., 2019; Parks et al., 2020) to the order *Lachnospirales* and to the family *Lachnospiraceae*.
**Description of *Candidatus* Choladousia intestinavium sp. nov.**
*Candidatus* Choladousia intestinavium (in.tes.tin.a’vi.um. L. neut. n. *intestinum* gut; L. fem. n. *avis* bird; N.L. gen. n. *intestinavium* of the gut of birds)
A bacterial species identified by metagenomic analyses. This species includes all bacteria with genomes that show ≥95% average nucleotide identity (ANI) to the type genome, which has been assigned the MAG ID ChiSjej4B22-8148 and which is available via NCBI BioSample SAMN15817012. The GC content of the type genome is 49.57% and the genome length is 2.9 Mbp.
**Description of *Candidatus* Choladousia intestinigallinarum sp. nov.**
*Candidatus* Choladousia intestinigallinarum (in.tes.ti.ni.gal.li.na’rum. L. neut. n. *intestinum* gut; L. fem. n. *gallina* hen; N.L. gen. n. *intestinigallinarum* of the gut of the hens)
A bacterial species identified by metagenomic analyses. This species includes all bacteria with genomes that show ≥95% average nucleotide identity (ANI) to the type genome, which has been assigned the MAG ID ChiBcec11-13528 and which is available via NCBI BioSample SAMN15817065. The GC content of the type genome is 48.69% and the genome length is 3.1 Mbp.
**Description of *Candidatus* Choladousia intestinipullorum sp. nov.**
*Candidatus* Choladousia intestinipullorum (in.tes.ti.ni.pul.lo’rum. L. neut. n. *intestinum* gut; L. masc. n. *pullus* a young chicken; N.L. gen. n. *intestinipullorum* of the gut of young chickens)
A bacterial species identified by metagenomic analyses. This species includes all bacteria with genomes that show ≥95% average nucleotide identity (ANI) to the type genome, which has been assigned the MAG ID ChiSjej5B23-16397 and which is available via NCBI BioSample SAMN15817078. The GC content of the type genome is 50.54% and the genome length is 2.2 Mbp.
**Description of *Candidatus* Collinsella stercoripullorum sp. nov.**
*Candidatus* Collinsella stercoripullorum (ster.co.ri.pul.lo’rum. L. neut. n. *stercus* dung; L. masc. n. *pullus* a young chicken; N.L. gen. n. *stercoripullorum* of the faceces of young chickens)
A bacterial species identified by metagenomic analyses. This species includes all bacteria with genomes that show ≥95% average nucleotide identity (ANI) to the type genome, which has been assigned the MAG ID ChiGjej6B6-20822 and which is available via NCBI BioSample SAMN15816681. The GC content of the type genome is 68.61% and the genome length is 2.3 Mbp.
**Description of *Candidatus* Companilactobacillus pullicola sp. nov.**
*Candidatus* Companilactobacillus pullicola (pul.li’co.la. L. masc. n. *pullus* a young chicken; L. suff. *-cola* inhabitant of; N.L. n. *pullicola* an inhabitant of young chickens)
A bacterial species identified by metagenomic analyses. This species includes all bacteria with genomes that show ≥95% average nucleotide identity (ANI) to the type genome, which has been assigned the MAG ID 3204 and which is available via NCBI BioSample SAMN15816700. The GC content of the type genome is 35.87% and the genome length is 2.9 Mbp.
**Description of *Candidatus* Coprenecus gen. nov.**
*Candidatus* Coprenecus (Copr.en.e’cus. Gr. fem. n. *kopros* dung; Gr. masc. *enoikos* inhabitant; N.L. masc. n. *Coprenecus* a microbe associated with faeces)
A bacterial genus identified by metagenomic analyses. The genus includes all bacteria with genomes that show ≥60% average amino acid identity (AAI) to the type genome from the type species *Candidatus* Coprenecus pullicola. This is a name for the alphanumeric GTDB genus CAG-831. This genus has been assigned by GTDB-Tk v1.3.0 working on GTDB Release 05-RS95 (Chaumeil et al., 2019; Parks et al., 2020) to the order *Bacteroidales* and to the family *UBA932*.

**Table:** 

**Description of *Candidatus* Coprenecus avistercoris sp. nov.**
*Candidatus* Coprenecus avistercoris (a.vi.ster’co.ris. L. fem. n. *avis* bird; L. neut. n. *stercus* dung; N.L. gen. n. *avistercoris* of bird faeces)
A bacterial species identified by metagenomic analyses. This species includes all bacteria with genomes that show ≥95% average nucleotide identity (ANI) to the type genome, which has been assigned the MAG ID ChiHjej13B12-12457 and which is available via NCBI BioSample SAMN15817175. This is a new name for the alphanumeric GTDB species sp000432775. The GC content of the type genome is 56.44% and the genome length is 2.0 Mbp.
**Description of *Candidatus* Coprenecus merdigallinarum sp. nov.**
*Candidatus* Coprenecus merdigallinarum (mer.di.gal.li.na’rum. L. fem. n. *merda* faeces; L. fem. n. *gallina* hen; N.L. gen. n. *merdigallinarum* of hen faeces)
A bacterial species identified by metagenomic analyses. This species includes all bacteria with genomes that show ≥95% average nucleotide identity (ANI) to the type genome, which has been assigned the MAG ID ChiHecolR1B25-18470 and which is available via NCBI BioSample SAMN15817066. The GC content of the type genome is 55.94% and the genome length is 2.2 Mbp.
**Description of *Candidatus* Coprenecus merdipullorum sp. nov.**
*Candidatus* Coprenecus merdipullorum (mer.di.pul.lo’rum. L. fem. n. *merda* faeces; L. masc. n. *pullus* a young chicken; N.L. gen. n. *merdipullorum* of the faeces of young chickens)
A bacterial species identified by metagenomic analyses. This species includes all bacteria with genomes that show ≥95% average nucleotide identity (ANI) to the type genome, which has been assigned the MAG ID Gambia11-1358 and which is available via NCBI BioSample SAMN15817068. The GC content of the type genome is 54.29% and the genome length is 2.1 Mbp.
**Description of *Candidatus* Coprenecus pullicola sp. nov.**
*Candidatus* Coprenecus pullicola (pul.li’co.la. L. masc. n. *pullus* a young chicken; L. suff. *-cola* inhabitant of; N.L. n. *pullicola* an inhabitant of young chickens)
A bacterial species identified by metagenomic analyses. This species includes all bacteria with genomes that show ≥95% average nucleotide identity (ANI) to the type genome, which has been assigned the MAG ID ChiHjej9B8-15444 and which is available via NCBI BioSample SAMN15817077. The GC content of the type genome is 52.53% and the genome length is 2.1 Mbp.
**Description of *Candidatus* Coprenecus pullistercoris sp. nov.**
*Candidatus* Coprenecus pullistercoris (pul.li.ster’co.ris. L. masc. n. *pullus* a young chicken; L. neut. n. *stercus* dung; N.L. gen. n. *pullistercoris* of young chicken faeces)
A bacterial species identified by metagenomic analyses. This species includes all bacteria with genomes that show ≥95% average nucleotide identity (ANI) to the type genome, which has been assigned the MAG ID Gambia18-42 and which is available via NCBI BioSample SAMN15817084. The GC content of the type genome is 52.64% and the genome length is 2.0 Mbp.
**Description of *Candidatus* Coprenecus stercoravium sp. nov.**
*Candidatus* Coprenecus stercoravium (ster.cor.a’vi.um. L. neut. n. *stercus* dung; L. fem. n. *avis* bird; N.L. gen. n. *stercoravium* of bird faeces)
A bacterial species identified by metagenomic analyses. This species includes all bacteria with genomes that show ≥95% average nucleotide identity (ANI) to the type genome, which has been assigned the MAG ID Gambia16-554 and which is available via NCBI BioSample SAMN15817085. The GC content of the type genome is 51.84% and the genome length is 1.8 Mbp.
**Description of *Candidatus* Coprenecus stercorigallinarum sp. nov.**
*Candidatus* Coprenecus stercorigallinarum (ster.co.ri.gal.li.na’rum. L. neut. n. *stercus* dung; L. fem. n. *gallina* hen; N.L. gen. n. *stercorigallinarum* of hen faeces)
A bacterial species identified by metagenomic analyses. This species includes all bacteria with genomes that show ≥95% average nucleotide identity (ANI) to the type genome, which has been assigned the MAG ID 3382 and which is available via NCBI BioSample SAMN15817086. The GC content of the type genome is 53.66% and the genome length is 2.1 Mbp.
**Description of *Candidatus* Coprenecus stercoripullorum sp. nov.**
*Candidatus* Coprenecus stercoripullorum (ster.co.ri.pul.lo’rum. L. neut. n. *stercus* dung; L. masc. n. *pullus* a young chicken; N.L. gen. n. *stercoripullorum* of the faceces of young chickens)
A bacterial species identified by metagenomic analyses. This species includes all bacteria with genomes that show ≥95% average nucleotide identity (ANI) to the type genome, which has been assigned the MAG ID 7141 and which is available via NCBI BioSample SAMN15817115. The GC content of the type genome is 50.92% and the genome length is 1.7 Mbp.

**Table:** 

**Description of *Candidatus* Coprocola gen. nov.**
*Candidatus* Coprocola (Co.pro’co.la. Gr. fem. n. *kopros* dung; L. suff. *-cola* inhabitant of; N.L. fem. n. *Coprocola* a microbe associated with faeces)
A bacterial genus identified by metagenomic analyses. The genus includes all bacteria with genomes that show ≥60% average amino acid identity (AAI) to the type genome from the type species *Candidatus* Coprocola pullicola. This is a name for the alphanumeric GTDB genus ASF356. This genus has been assigned by GTDB-Tk v1.3.0 working on GTDB Release 05-RS95 (Chaumeil et al., 2019; Parks et al., 2020) to the order *Lachnospirales* and to the family *Anaerotignaceae*.
**Description of *Candidatus* Coprocola pullicola sp. nov.**
*Candidatus* Coprocola pullicola (pul.li’co.la. L. masc. n. *pullus* a young chicken; L. suff. *-cola* inhabitant of; N.L. n. *pullicola* an inhabitant of young chickens)
A bacterial species identified by metagenomic analyses. This species includes all bacteria with genomes that show ≥95% average nucleotide identity (ANI) to the type genome, which has been assigned the MAG ID CHK193-15662 and which is available via NCBI BioSample SAMN15817043. The GC content of the type genome is 33.91% and the genome length is 3.0 Mbp.
**Description of *Candidatus* Copromonas gen. nov.**
*Candidatus* Copromonas (Co.pro.mo’nas. Gr. fem. n. *kopros* dung; L. fem. n. *monas* a monad; N.L. fem. n. *Copromonas* a microbe associated with faeces)
A bacterial genus identified by metagenomic analyses. The genus includes all bacteria with genomes that show ≥60% average amino acid identity (AAI) to the type genome from the type species *Candidatus* Copromonas avistercoris. This is a name for the alphanumeric GTDB genus CAG-81. This genus has been assigned by GTDB-Tk v1.3.0 working on GTDB Release 05-RS95 (Chaumeil et al., 2019; Parks et al., 2020) to the order *Lachnospirales* and to the family *Lachnospiraceae*.
**Description of *Candidatus* Copromonas avistercoris sp. nov.**
*Candidatus* Copromonas avistercoris (a.vi.ster’co.ris. L. fem. n. *avis* bird; L. neut. n. *stercus* dung; N.L. gen. n. *avistercoris* of bird faeces)
A bacterial species identified by metagenomic analyses. This species includes all bacteria with genomes that show ≥95% average nucleotide identity (ANI) to the type genome, which has been assigned the MAG ID ChiSjej3B21-5768 and which is available via NCBI BioSample SAMN15817005. The GC content of the type genome is 52.88% and the genome length is 2.2 Mbp.
**Description of *Candidatus* Copromonas faecavium sp. nov.**
*Candidatus* Copromonas faecavium (faec.a’vi.um. L. fem. n. *faex, faecis* excrement; L. fem. n. *avis* bird; N.L. gen. n. *faecavium* of bird faeces)
A bacterial species identified by metagenomic analyses. This species includes all bacteria with genomes that show ≥95% average nucleotide identity (ANI) to the type genome, which has been assigned the MAG ID CHK180-2868 and which is available via NCBI BioSample SAMN15817009. The GC content of the type genome is 50.20% and the genome length is 2.7 Mbp.
**Description of *Candidatus* Copromorpha gen. nov.**
*Candidatus* Copromorpha (Co.pro.mor’pha. Gr. fem. n. *kopros* dung; Gr. fem. n. *morphe* a form, shape; N.L. fem. n. *Copromorpha* a microbe associated with faeces)
A bacterial genus identified by metagenomic analyses. The genus includes all bacteria with genomes that show ≥60% average amino acid identity (AAI) to the type genome from the type species *Candidatus* Copromorpha excrementavium. This is a name for the alphanumeric GTDB genus UBA1191. This genus has been assigned by GTDB-Tk v1.3.0 working on GTDB Release 05-RS95 (Chaumeil et al., 2019; Parks et al., 2020) to the order *Peptostreptococcales* and to the family *Anaerovoracaceae*.
**Description of *Candidatus* Copromorpha excrementavium sp. nov.**
*Candidatus* Copromorpha excrementavium (ex.cre.ment.a’vi.um. L. neut. n. *excrementum* excrement; L. fem. n. *avis* bird; N.L. gen. n. *excrementavium* of bird excrement)
A bacterial species identified by metagenomic analyses. This species includes all bacteria with genomes that show ≥95% average nucleotide identity (ANI) to the type genome, which has been assigned the MAG ID CHK176-22527 and which is available via NCBI BioSample SAMN15817193. This is a new name for the alphanumeric GTDB species sp900542385. The GC content of the type genome is 42.88% and the genome length is 1.8 Mbp.
**Description of *Candidatus* Copromorpha excrementigallinarum sp. nov.**
*Candidatus* Copromorpha excrementigallinarum (ex.cre.men.ti.gal.li.na’rum. L. neut. n. *excrementum* excrement; L. fem. n. *gallina* hen; N.L. gen. n. *excrementigallinarum* of hen excrement)
A bacterial species identified by metagenomic analyses. This species includes all bacteria with genomes that show ≥95% average nucleotide identity (ANI) to the type genome, which has been assigned the MAG ID ChiHcec3-6078 and which is available via NCBI BioSample SAMN15817131. The GC content of the type genome is 48.15% and the genome length is 2.0 Mbp.

**Table:** 

**Description of *Candidatus* Copromorpha excrementipullorum sp. nov.**
*Candidatus* Copromorpha excrementipullorum (ex.cre.men.ti.pul.lo’rum. L. neut. n. *excrementum* excrement; L. masc. n. *pullus* a young chicken; N.L. gen. n. *excrementipullorum* of young chicken excrement)
A bacterial species identified by metagenomic analyses. This species includes all bacteria with genomes that show ≥95% average nucleotide identity (ANI) to the type genome, which has been assigned the MAG ID ChiSjej4B22-8349 and which is available via NCBI BioSample SAMN15817205. This is a new name for the alphanumeric GTDB species sp900543485. The GC content of the type genome is 49.77% and the genome length is 1.9 Mbp.
**Description of *Candidatus* Coproplasma gen. nov.**
*Candidatus* Coproplasma (Co.pro.plas’ma. Gr. fem. n. *kopros* dung; Gr. neut. n. *plasma* a form; N.L. neut. n. *Coproplasma* a microbe associated with faeces)
A bacterial genus identified by metagenomic analyses. The genus includes all bacteria with genomes that show ≥60% average amino acid identity (AAI) to the type genome from the type species *Candidatus* Coproplasma stercoravium. This is a name for the alphanumeric GTDB genus UBA11940. This genus has been assigned by GTDB-Tk v1.3.0 working on GTDB Release 05-RS95 (Chaumeil et al., 2019; Parks et al., 2020) to the order *Christensenellales* and to the family *Borkfalkiaceae*.
**Description of *Candidatus* Coproplasma avicola sp. nov.**
*Candidatus* Coproplasma avicola (a.vi’co.la. L. fem. n. *avis* bird; L. suff. *-cola* inhabitant of; N.L. n. *avicola* inhabitant of birds)
A bacterial species identified by metagenomic analyses. This species includes all bacteria with genomes that show ≥95% average nucleotide identity (ANI) to the type genome, which has been assigned the MAG ID ChiW16-3235 and which is available via NCBI BioSample SAMN15817075. The GC content of the type genome is 51.11% and the genome length is 1.5 Mbp.
**Description of *Candidatus* Coproplasma avistercoris sp. nov.**
*Candidatus* Coproplasma avistercoris (a.vi.ster’co.ris. L. fem. n. *avis* bird; L. neut. n. *stercus* dung; N.L. gen. n. *avistercoris* of bird faeces)
A bacterial species identified by metagenomic analyses. This species includes all bacteria with genomes that show ≥95% average nucleotide identity (ANI) to the type genome, which has been assigned the MAG ID ChiW7-3743 and which is available via NCBI BioSample SAMN15817023. The GC content of the type genome is 56.32% and the genome length is 1.4 Mbp.
**Description of *Candidatus* Coproplasma excrementavium sp. nov.**
*Candidatus* Coproplasma excrementavium (ex.cre.ment.a’vi.um. L. neut. n. *excrementum* excrement; L. fem. n. *avis* bird; N.L. gen. n. *excrementavium* of bird excrement)
A bacterial species identified by metagenomic analyses. This species includes all bacteria with genomes that show ≥95% average nucleotide identity (ANI) to the type genome, which has been assigned the MAG ID CHK179-18245 and which is available via NCBI BioSample SAMN15817045. The GC content of the type genome is 50.26% and the genome length is 1.4 Mbp.
**Description of *Candidatus* Coproplasma excrementigallinarum sp. nov.**
*Candidatus* Coproplasma excrementigallinarum (ex.cre.men.ti.gal.li.na’rum. L. neut. n. *excrementum* excrement; L. fem. n. *gallina* hen; N.L. gen. n. *excrementigallinarum* of hen excrement)
A bacterial species identified by metagenomic analyses. This species includes all bacteria with genomes that show ≥95% average nucleotide identity (ANI) to the type genome, which has been assigned the MAG ID CHK195-12923 and which is available via NCBI BioSample SAMN15817050. The GC content of the type genome is 50.00% and the genome length is 1.4 Mbp.
**Description of *Candidatus* Coproplasma excrementipullorum sp. nov.**
*Candidatus* Coproplasma excrementipullorum (ex.cre.men.ti.pul.lo’rum. L. neut. n. *excrementum* excrement; L. masc. n. *pullus* a young chicken; N.L. gen. n. *excrementipullorum* of young chicken excrement)
A bacterial species identified by metagenomic analyses. This species includes all bacteria with genomes that show ≥95% average nucleotide identity (ANI) to the type genome, which has been assigned the MAG ID 10570 and which is available via NCBI BioSample SAMN15817148. The GC content of the type genome is 51.24% and the genome length is 1.7 Mbp.
**Description of *Candidatus* Coproplasma stercoravium sp. nov.**
*Candidatus* Coproplasma stercoravium (ster.cor.a’vi.um. L. neut. n. *stercus* dung; L. fem. n. *avis* bird; N.L. gen. n. *stercoravium* of bird faeces)
A bacterial species identified by metagenomic analyses. This species includes all bacteria with genomes that show ≥95% average nucleotide identity (ANI) to the type genome, which has been assigned the MAG ID CHK180-19203 and which is available via NCBI BioSample SAMN15817006. The GC content of the type genome is 51.86% and the genome length is 1.6 Mbp.

**Table:** 

**Description of *Candidatus* Coproplasma stercorigallinarum sp. nov.**
*Candidatus* Coproplasma stercorigallinarum (ster.co.ri.gal.li.na’rum. L. neut. n. *stercus* dung; L. fem. n. *gallina* hen; N.L. gen. n. *stercorigallinarum* of hen faeces)
A bacterial species identified by metagenomic analyses. This species includes all bacteria with genomes that show ≥95% average nucleotide identity (ANI) to the type genome, which has been assigned the MAG ID CHK191-24566 and which is available via NCBI BioSample SAMN15817171. This is a new name for the alphanumeric GTDB species sp900549005. The GC content of the type genome is 51.83% and the genome length is 1.6 Mbp.
**Description of *Candidatus* Coproplasma stercoripullorum sp. nov.**
*Candidatus* Coproplasma stercoripullorum (ster.co.ri.pul.lo’rum. L. neut. n. *stercus* dung; L. masc. n. *pullus* a young chicken; N.L. gen. n. *stercoripullorum* of the faceces of young chickens)
A bacterial species identified by metagenomic analyses. This species includes all bacteria with genomes that show ≥95% average nucleotide identity (ANI) to the type genome, which has been assigned the MAG ID ChiW25-3613 and which is available via NCBI BioSample SAMN15817071. The GC content of the type genome is 51.17% and the genome length is 1.7 Mbp.
**Description of *Candidatus* Coprosoma gen. nov.**
*Candidatus* Coprosoma (Co.pro.so’ma. Gr. fem. n. *kopros* dung; Gr. neut. n. *soma* a body; N.L. neut. n. *Coprosoma* a microbe associated with faeces)
A bacterial genus identified by metagenomic analyses. The genus includes all bacteria with genomes that show ≥60% average amino acid identity (AAI) to the type genome from the type species *Candidatus* Coprosoma intestinipullorum. This is a name for the alphanumeric GTDB genus CAG-822. This genus has been assigned by GTDB-Tk v1.3.0 working on GTDB Release 05-RS95 (Chaumeil et al., 2019; Parks et al., 2020) to the order *RF39* and to the family *CAG-822*.
**Description of *Candidatus* Coprosoma intestinipullorum sp. nov.**
*Candidatus* Coprosoma intestinipullorum (in.tes.ti.ni.pul.lo’rum. L. neut. n. *intestinum* gut; L. masc. n. *pullus* a young chicken; N.L. gen. n. *intestinipullorum* of the gut of young chickens)
A bacterial species identified by metagenomic analyses. This species includes all bacteria with genomes that show ≥95% average nucleotide identity (ANI) to the type genome, which has been assigned the MAG ID CHK147-3167 and which is available via NCBI BioSample SAMN15817008. The GC content of the type genome is 31.20% and the genome length is 1.3 Mbp.
**Description of *Candidatus* Coprousia gen. nov.**
*Candidatus* Coprousia (Copr.ou’si.a. Gr. fem. n. *kopros* dung; Gr. fem. n. *ousia* an essence; N.L. fem. n. *Coprousia* a microbe associated with faeces)
A bacterial genus identified by metagenomic analyses. The genus includes all bacteria with genomes that show ≥60% average amino acid identity (AAI) to the type genome from the type species *Candidatus* Coprousia avicola. This is a name for the alphanumeric GTDB genus An7. This genus has been assigned by GTDB-Tk v1.3.0 working on GTDB Release 05-RS95 (Chaumeil et al., 2019; Parks et al., 2020) to the order *Coriobacteriales* and to the family *Coriobacteriaceae*.
**Description of *Candidatus* Coprousia avicola sp. nov.**
*Candidatus* Coprousia avicola (a.vi’co.la. L. fem. n. *avis* bird; L. suff. *-cola* inhabitant of; N.L. n. *avicola* inhabitant of birds)
A bacterial species identified by metagenomic analyses. This species includes all bacteria with genomes that show ≥95% average nucleotide identity (ANI) to the type genome, which has been assigned the MAG ID ChiHjej11B10-5566 and which is available via NCBI BioSample SAMN15817195. This is a new name for the alphanumeric GTDB species sp002159765. The GC content of the type genome is 65.47% and the genome length is 2.4 Mbp.
**Description of *Candidatus* Coprovicinus gen. nov.**
*Candidatus* Coprovicinus (Co.pro.vi.ci’nus. Gr. fem. n. *kopros* dung; L. masc. n. *vicinus* a neighbour; N.L. masc. n. *Coprovicinus* a microbe associated with faeces)
A bacterial genus identified by metagenomic analyses. The genus includes all bacteria with genomes that show ≥60% average amino acid identity (AAI) to the type genome from the type species *Candidatus* Coprovicinus avistercoris. This is a name for the alphanumeric GTDB genus UMGS1418. This genus has been assigned by GTDB-Tk v1.3.0 working on GTDB Release 05-RS95 (Chaumeil et al., 2019; Parks et al., 2020) to the order *Coriobacteriales* and to the family *Atopobiaceae*.
**Description of *Candidatus* Coprovicinus avistercoris sp. nov.**
*Candidatus* Coprovicinus avistercoris (a.vi.ster’co.ris. L. fem. n. *avis* bird; L. neut. n. *stercus* dung; N.L. gen. n. *avistercoris* of bird faeces)
A bacterial species identified by metagenomic analyses. This species includes all bacteria with genomes that show ≥95% average nucleotide identity (ANI) to the type genome, which has been assigned the MAG ID ChiHjej12B11-29160 and which is available via NCBI BioSample SAMN15817198. This is a new name for the alphanumeric GTDB species sp900551595. The GC content of the type genome is 49.51% and the genome length is 1.7 Mbp.

**Table:** 

**Description of *Candidatus* Coprovivens gen. nov.**
*Candidatus* Coprovivens (Co.pro.vi’vens. Gr. fem. n. *kopros* dung; N.L. pres. part. *vivens* living; N.L. fem. n. *Coprovivens* a microbe associated with faeces)
A bacterial genus identified by metagenomic analyses. The genus includes all bacteria with genomes that show ≥60% average amino acid identity (AAI) to the type genome from the type species *Candidatus* Coprovivens excrementavium. This is a name for the alphanumeric GTDB genus UBA11963. This genus has been assigned by GTDB-Tk v1.3.0 working on GTDB Release 05-RS95 (Chaumeil et al., 2019; Parks et al., 2020) to the order *RF39* and to the family *CAG-1000*.
**Description of *Candidatus* Coprovivens excrementavium sp. nov.**
*Candidatus* Coprovivens excrementavium (ex.cre.ment.a’vi.um. L. neut. n. *excrementum* excrement; L. fem. n. *avis* bird; N.L. gen. n. *excrementavium* of bird excrement)
A bacterial species identified by metagenomic analyses. This species includes all bacteria with genomes that show ≥95% average nucleotide identity (ANI) to the type genome, which has been assigned the MAG ID 3297 and which is available via NCBI BioSample SAMN15817083. The GC content of the type genome is 28.25% and the genome length is 2.2 Mbp.
**Description of *Candidatus* Corynebacterium avicola sp. nov.**
*Candidatus* Corynebacterium avicola (a.vi’co.la. L. fem. n. *avis* bird; L. suff. *-cola* inhabitant of; N.L. n. *avicola* inhabitant of birds)
A bacterial species identified by metagenomic analyses. This species includes all bacteria with genomes that show ≥95% average nucleotide identity (ANI) to the type genome, which has been assigned the MAG ID CHK32-1732 and which is available via NCBI BioSample SAMN15816750. The GC content of the type genome is 66.95% and the genome length is 3.1 Mbp.
**Description of *Candidatus* Corynebacterium faecigallinarum sp. nov.**
*Candidatus* Corynebacterium faecigallinarum (fae.ci.gal.li.na’rum. L. fem. n. *faex, faecis* excrement; L. fem. n. *gallina* hen; N.L. gen. n. *faecigallinarum* of hen faeces)
A bacterial species identified by metagenomic analyses. This species includes all bacteria with genomes that show ≥95% average nucleotide identity (ANI) to the type genome, which has been assigned the MAG ID ChiHjej13B12-4958 and which is available via NCBI BioSample SAMN15816631. The GC content of the type genome is 66.91% and the genome length is 2.8 Mbp.
**Description of *Candidatus* Corynebacterium faecipullorum sp. nov.**
*Candidatus* Corynebacterium faecipullorum (fae.ci.pul.lo’rum. L. fem. n. *faex, faecis* excrement; L. masc. n. *pullus* a young chicken; N.L. gen. n. *faecipullorum* of young chicken faeces)
A bacterial species identified by metagenomic analyses. This species includes all bacteria with genomes that show ≥95% average nucleotide identity (ANI) to the type genome, which has been assigned the MAG ID 913 and which is available via NCBI BioSample SAMN15816858. This is a new name for the alphanumeric GTDB species sp001836165. The GC content of the type genome is 61.16% and the genome length is 2.1 Mbp.
**Description of *Candidatus* Corynebacterium gallistercoris sp. nov.**
*Candidatus* Corynebacterium gallistercoris (gal.li.ster’co.ris. L. masc. n. *gallus* chicken; L. neut. n. *stercus* dung; N.L. gen. n. *gallistercoris* of chicken faeces)
A bacterial species identified by metagenomic analyses. This species includes all bacteria with genomes that show ≥95% average nucleotide identity (ANI) to the type genome, which has been assigned the MAG ID 4376 and which is available via NCBI BioSample SAMN15816747. The GC content of the type genome is 62.96% and the genome length is 2.0 Mbp.
**Description of *Candidatus* Corynebacterium intestinavium sp. nov.**
*Candidatus* Corynebacterium intestinavium (in.tes.tin.a’vi.um. L. neut. n. *intestinum* gut; L. fem. n. *avis* bird; N.L. gen. n. *intestinavium* of the gut of birds)
A bacterial species identified by metagenomic analyses. This species includes all bacteria with genomes that show ≥95% average nucleotide identity (ANI) to the type genome, which has been assigned the MAG ID 5925 and which is available via NCBI BioSample SAMN15816787. The GC content of the type genome is 65.59% and the genome length is 1.9 Mbp.
**Description of *Candidatus* Cottocaccomicrobium gen. nov.**
*Candidatus* Cottocaccomicrobium (Cot.to.cac.co.mi.cro’bi.um. Gr. masc. n. *kottos* chicken; Gr. fem. n. *kakke* faeces; N.L. neut. n. *microbium* a microbe; N.L. neut. n. *Cottocaccomicrobium* a microbe asociated with chicken faeces)
A bacterial genus identified by metagenomic analyses. The genus includes all bacteria with genomes that show ≥60% average amino acid identity (AAI) to the type genome from the type species *Candidatus* Cottocaccamicrobium excrementipullorum. This genus was identified but not named by Glendinning et al. (2020). This genus has been assigned by GTDB-Tk v1.3.0 working on GTDB Release 05-RS95 (Chaumeil et al., 2019; Parks et al., 2020) to the order *Lachnospirales* and to the family *Lachnospiraceae*.

**Table:** 

**Description of *Candidatus* Cottocaccomicrobium excrementipullorum sp. nov.**
*Candidatus* Cottocaccomicrobium excrementipullorum (ex.cre.men.ti.pul.lo’rum. L. neut. n. *excrementum* excrement; L. masc. n. *pullus* a young chicken; N.L. gen. n. *excrementipullorum* of young chicken excrement)
A bacterial species identified by metagenomic analyses. This species includes all bacteria with genomes that show ≥95% average nucleotide identity (ANI) to the type genome, which has been assigned the MAG ID CHK179-5732 and which is available via NCBI BioSample SAMN15816932. The GC content of the type genome is 47.54% and the genome length is 3.4 Mbp.
**Description of *Candidatus* Cryptobacteroides gen. nov.**
*Candidatus* Cryptobacteroides (Cryp.to.bac.te.ro’i.des. Gr. masc. adj. *kryptos* hidden; N.L. masc. n. *Bacteroides* a genus name; N.L. masc. n. *Cryptobacteroides* a genus related to the genus *Bacteroides* but distinct from it)
A bacterial genus identified by metagenomic analyses. The genus includes all bacteria with genomes that show ≥60% average amino acid identity (AAI) to the type genome from the type species *Candidatus* Cryptobacteroides avicola. This is a name for the alphanumeric GTDB genus RC9. This genus has been assigned by GTDB-Tk v1.3.0 working on GTDB Release 05-RS95 (Chaumeil et al., 2019; Parks et al., 2020) to the order *Bacteroidales* and to the family *UBA932*.
**Description of *Candidatus* Cryptobacteroides avicola sp. nov.**
*Candidatus* Cryptobacteroides avicola (a.vi’co.la. L. fem. n. *avis* bird; L. suff. *-cola* inhabitant of; N.L. n. *avicola* inhabitant of birds)
A bacterial species identified by metagenomic analyses. This species includes all bacteria with genomes that show ≥95% average nucleotide identity (ANI) to the type genome, which has been assigned the MAG ID G3-8215 and which is available via NCBI BioSample SAMN15817056. The GC content of the type genome is 49.81% and the genome length is 2.6 Mbp.
**Description of *Candidatus* Cryptobacteroides avistercoris sp. nov.**
*Candidatus* Cryptobacteroides avistercoris (a.vi.ster’co.ris. L. fem. n. *avis* bird; L. neut. n. *stercus* dung; N.L. gen. n. *avistercoris* of bird faeces)
A bacterial species identified by metagenomic analyses. This species includes all bacteria with genomes that show ≥95% average nucleotide identity (ANI) to the type genome, which has been assigned the MAG ID B3-1481 and which is available via NCBI BioSample SAMN15817057. The GC content of the type genome is 59.28% and the genome length is 1.6 Mbp.
**Description of *Candidatus* Cryptobacteroides excrementavium sp. nov.**
*Candidatus* Cryptobacteroides excrementavium (ex.cre.ment.a’vi.um. L. neut. n. *excrementum* excrement; L. fem. n. *avis* bird; N.L. gen. n. *excrementavium* of bird excrement)
A bacterial species identified by metagenomic analyses. This species includes all bacteria with genomes that show ≥95% average nucleotide identity (ANI) to the type genome, which has been assigned the MAG ID B2-16538 and which is available via NCBI BioSample SAMN15817059. The GC content of the type genome is 50.29% and the genome length is 2.1 Mbp.
**Description of *Candidatus* Cryptobacteroides excrementigallinarum sp. nov.**
*Candidatus* Cryptobacteroides excrementigallinarum (ex.cre.men.ti.gal.li.na’rum. L. neut. n. *excrementum* excrement; L. fem. n. *gallina* hen; N.L. gen. n. *excrementigallinarum* of hen excrement)
A bacterial species identified by metagenomic analyses. This species includes all bacteria with genomes that show ≥95% average nucleotide identity (ANI) to the type genome, which has been assigned the MAG ID ChiHecolR1B25-7735 and which is available via NCBI BioSample SAMN15817167. This is a new name for the alphanumeric GTDB species sp900543205. The GC content of the type genome is 57.80% and the genome length is 1.8 Mbp.
**Description of *Candidatus* Cryptobacteroides excrementipullorum sp. nov.**
*Candidatus* Cryptobacteroides excrementipullorum (ex.cre.men.ti.pul.lo’rum. L. neut. n. *excrementum* excrement; L. masc. gen. pl. n. *pullorum* of young chickens; N.L. gen. n. *excrementipullorum* of young chicken excrement)
A bacterial species identified by metagenomic analyses. This species includes all bacteria with genomes that show ≥95% average nucleotide identity (ANI) to the type genome, which has been assigned the MAG ID 2478 and which is available via NCBI BioSample SAMN15817061. The GC content of the type genome is 52.35% and the genome length is 2.5 Mbp.
**Description of *Candidatus* Cryptobacteroides faecavium sp. nov.**
*Candidatus* Cryptobacteroides faecavium (faec.a’vi.um. L. fem. n. *faex, faecis* excrement; L. fem. n. *avis* bird; N.L. gen. n. *faecavium* of bird faeces)
A bacterial species identified by metagenomic analyses. This species includes all bacteria with genomes that show ≥95% average nucleotide identity (ANI) to the type genome, which has been assigned the MAG ID B2-22910 and which is available via NCBI BioSample SAMN15817063. The GC content of the type genome is 52.41% and the genome length is 2.4 Mbp.

**Table:** 

**Description of *Candidatus* Cryptobacteroides faecigallinarum sp. nov.**
*Candidatus* Cryptobacteroides faecigallinarum (fae.ci.gal.li.na’rum. L. fem. n. *faex, faecis* excrement; L. fem. n. *gallina* hen; N.L. gen. n. *faecigallinarum* of hen faeces)
A bacterial species identified by metagenomic analyses. This species includes all bacteria with genomes that show ≥95% average nucleotide identity (ANI) to the type genome, which has been assigned the MAG ID B1-13419 and which is available via NCBI BioSample SAMN15817072. The GC content of the type genome is 49.87% and the genome length is 2.0 Mbp.
**Description of *Candidatus* Cryptobacteroides faecipullorum sp. nov.**
*Candidatus* Cryptobacteroides faecipullorum (fae.ci.pul.lo’rum. L. fem. n. *faex, faecis* excrement; L. masc. n. *pullus* a young chicken; N.L. gen. n. *faecipullorum* of young chicken faeces)
A bacterial species identified by metagenomic analyses. This species includes all bacteria with genomes that show ≥95% average nucleotide identity (ANI) to the type genome, which has been assigned the MAG ID B1-15692 and which is available via NCBI BioSample SAMN15817080. The GC content of the type genome is 49.55% and the genome length is 2.3 Mbp.
**Description of *Candidatus* Cryptobacteroides gallistercoris sp. nov.**
*Candidatus* Cryptobacteroides gallistercoris (gal.li.ster’co.ris. L. masc. n *gallus* chicken; L. neut. n. *stercus* dung; N.L. gen. n. *gallistercoris* of chicken faeces)
A bacterial species identified by metagenomic analyses. This species includes all bacteria with genomes that show ≥95% average nucleotide identity (ANI) to the type genome, which has been assigned the MAG ID F1-3629 and which is available via NCBI BioSample SAMN15817088. The GC content of the type genome is 51.79% and the genome length is 2.0 Mbp.
**Description of *Candidatus* Cryptobacteroides intestinavium sp. nov.**
*Candidatus* Cryptobacteroides intestinavium (in.tes.tin.a’vi.um. L. neut. n. *intestinum* gut; L. fem. n. *avis* bird; N.L. gen. n. *intestinavium* of the gut of birds)
A bacterial species identified by metagenomic analyses. This species includes all bacteria with genomes that show ≥95% average nucleotide identity (ANI) to the type genome, which has been assigned the MAG ID B1-20833 and which is available via NCBI BioSample SAMN15817087. The GC content of the type genome is 51.51% and the genome length is 2.4 Mbp.
**Description of *Candidatus* Cryptobacteroides intestinigallinarum sp. nov.**
*Candidatus* Cryptobacteroides intestinigallinarum (in.tes.ti.ni.gal.li.na’rum. L. neut. n. *intestinum* gut; L. fem. n. *gallina* hen; N.L. gen. n. *intestinigallinarum* of the gut of the hens)
A bacterial species identified by metagenomic analyses. This species includes all bacteria with genomes that show ≥95% average nucleotide identity (ANI) to the type genome, which has been assigned the MAG ID B1-3475 and which is available via NCBI BioSample SAMN15817089. The GC content of the type genome is 49.55% and the genome length is 2.3 Mbp.
**Description of *Candidatus* Cryptobacteroides intestinipullorum sp. nov.**
*Candidatus* Cryptobacteroides intestinipullorum (in.tes.ti.ni.pul.lo’rum. L. neut. n. *intestinum* gut; L. masc. n. *pullus* a young chicken; N.L. gen. n. *intestinipullorum* of the gut of young chickens)
A bacterial species identified by metagenomic analyses. This species includes all bacteria with genomes that show ≥95% average nucleotide identity (ANI) to the type genome, which has been assigned the MAG ID 33258 and which is available via NCBI BioSample SAMN15817090. The GC content of the type genome is 50.60% and the genome length is 2.4 Mbp.
**Description of *Candidatus* Cryptobacteroides merdavium sp. nov.**
*Candidatus* Cryptobacteroides merdavium (merd.a’vi.um. L. fem. n. *merda* faeces; L. fem. n. *avis* bird; N.L. gen. n. *merdavium* of bird faeces)
A bacterial species identified by metagenomic analyses. This species includes all bacteria with genomes that show ≥95% average nucleotide identity (ANI) to the type genome, which has been assigned the MAG ID D5-748 and which is available via NCBI BioSample SAMN15817104. The GC content of the type genome is 50.98% and the genome length is 2.5 Mbp.
**Description of *Candidatus* Cryptobacteroides merdigallinarum sp. nov.**
*Candidatus* Cryptobacteroides merdigallinarum (mer.di.gal.li.na’rum. L. fem. n. *merda* faeces; L. fem. n. *gallina* hen; N.L. gen. n. *merdigallinarum* of hen faeces)
A bacterial species identified by metagenomic analyses. This species includes all bacteria with genomes that show ≥95% average nucleotide identity (ANI) to the type genome, which has been assigned the MAG ID 20514 and which is available via NCBI BioSample SAMN15817110. The GC content of the type genome is 54.69% and the genome length is 2.3 Mbp.

**Table:** 

**Description of *Candidatus* Cryptobacteroides merdipullorum sp. nov.**
*Candidatus* Cryptobacteroides merdipullorum (mer.di.pul.lo’rum. L. fem. n. *merda* faeces; L. masc. n. *pullus* a young chicken; N.L. gen. n. *merdipullorum* of the faeces of young chickens)
A bacterial species identified by metagenomic analyses. This species includes all bacteria with genomes that show ≥95% average nucleotide identity (ANI) to the type genome, which has been assigned the MAG ID ChiHecec2B26-709 and which is available via NCBI BioSample SAMN15817116. The GC content of the type genome is 57.16% and the genome length is 2.0 Mbp.
**Description of *Candidatus* Cryptobacteroides pullicola sp. nov.**
*Candidatus* Cryptobacteroides pullicola (pul.li’co.la. L. masc. n. *pullus* a young chicken; L. suff. *-cola* inhabitant of; N.L. n. *pullicola* an inhabitant of young chickens)
A bacterial species identified by metagenomic analyses. This species includes all bacteria with genomes that show ≥95% average nucleotide identity (ANI) to the type genome, which has been assigned the MAG ID ChiHecec2B26-3624 and which is available via NCBI BioSample SAMN15817179. This is a new name for the alphanumeric GTDB species sp001915575. The GC content of the type genome is 58.01% and the genome length is 1.7 Mbp.
**Description of *Candidatus* Desulfovibrio faecigallinarum sp. nov.**
*Candidatus* Desulfovibrio faecigallinarum (fae.ci.gal.li.na’rum. L. fem. n. *faex, faecis* excrement; L. fem. n. *gallina* hen; N.L. gen. n. *faecigallinarum* of hen faeces)
A bacterial species identified by metagenomic analyses. This species includes all bacteria with genomes that show ≥95% average nucleotide identity (ANI) to the type genome, which has been assigned the MAG ID 8923 and which is available via NCBI BioSample SAMN15816873. This is a new name for the alphanumeric GTDB species sp002159665. The GC content of the type genome is 57.29% and the genome length is 2.0 Mbp.
**Description of *Candidatus* Desulfovibrio gallistercoris sp. nov.**
*Candidatus* Desulfovibrio gallistercoris (gal.li.ster’co.ris. L. masc. n. *gallus* chicken; L. neut. n. *stercus* dung; N.L. gen. n. *gallistercoris* of chicken faeces)
A bacterial species identified by metagenomic analyses. This species includes all bacteria with genomes that show ≥95% average nucleotide identity (ANI) to the type genome, which has been assigned the MAG ID ChiGjej2B2-32749 and which is available via NCBI BioSample SAMN15816654. The GC content of the type genome is 64.54% and the genome length is 2.8 Mbp.
**Description of *Candidatus* Desulfovibrio intestinavium sp. nov.**
*Candidatus* Desulfovibrio intestinavium (in.tes.tin.a’vi.um. L. neut. n. *intestinum* gut; L. fem. n. *avis* bird; N.L. gen. n. *intestinavium* of the gut of birds)
A bacterial species identified by metagenomic analyses. This species includes all bacteria with genomes that show ≥95% average nucleotide identity (ANI) to the type genome, which has been assigned the MAG ID 5032 and which is available via NCBI BioSample SAMN15816664. The GC content of the type genome is 64.60% and the genome length is 2.5 Mbp.
**Description of *Candidatus* Desulfovibrio intestinigallinarum sp. nov.**
*Candidatus* Desulfovibrio intestinigallinarum (in.tes.ti.ni.gal.li.na’rum. L. neut. n. *intestinum* gut; L. fem. n. *gallina* hen; N.L. gen. n. *intestinigallinarum* of the gut of the hens)
A bacterial species identified by metagenomic analyses. This species includes all bacteria with genomes that show ≥95% average nucleotide identity (ANI) to the type genome, which has been assigned the MAG ID ChiHecec3B27-2601 and which is available via NCBI BioSample SAMN15816737. The GC content of the type genome is 61.03% and the genome length is 2.9 Mbp.
**Description of *Candidatus* Desulfovibrio intestinipullorum sp. nov.**
*Candidatus* Desulfovibrio intestinipullorum (in.tes.ti.ni.pul.lo’rum. L. neut. n. *intestinum* gut; L. masc. n. *pullus* a young chicken; N.L. gen. n. *intestinipullorum* of the gut of young chickens)
A bacterial species identified by metagenomic analyses. This species includes all bacteria with genomes that show ≥95% average nucleotide identity (ANI) to the type genome, which has been assigned the MAG ID ChiHecec2B26-446 and which is available via NCBI BioSample SAMN15816774. The GC content of the type genome is 60.23% and the genome length is 2.8 Mbp.
**Description of *Candidatus* Dietzia intestinigallinarum sp. nov.**
*Candidatus* Dietzia intestinigallinarum (in.tes.ti.ni.gal.li.na’rum. L. neut. n. *intestinum* gut; L. fem. n. *gallina* hen; N.L. gen. n. *intestinigallinarum* of the gut of the hens)
A bacterial species identified by metagenomic analyses. This species includes all bacteria with genomes that show ≥95% average nucleotide identity (ANI) to the type genome, which has been assigned the MAG ID ChiHjej12B11-1528 and which is available via NCBI BioSample SAMN15816635. The GC content of the type genome is 69.86% and the genome length is 3.9 Mbp.

**Table:** 

**Description of *Candidatus* Dietzia intestinipullorum sp. nov.**
*Candidatus* Dietzia intestinipullorum (in.tes.ti.ni.pul.lo’rum. L. neut. n. *intestinum* gut; L. masc. n. *pullus* a young chicken; N.L. gen. n. *intestinipullorum* of the gut of young chickens)
A bacterial species identified by metagenomic analyses. This species includes all bacteria with genomes that show ≥95% average nucleotide identity (ANI) to the type genome, which has been assigned the MAG ID ChiHjej13B12-8321 and which is available via NCBI BioSample SAMN15816639. The GC content of the type genome is 71.26% and the genome length is 3.0 Mbp.
**Description of *Candidatus* Dietzia merdigallinarum sp. nov.**
*Candidatus* Dietzia merdigallinarum (mer.di.gal.li.na’rum. L. fem. n. *merda* faeces; L. fem. n. *gallina* hen; N.L. gen. n. *merdigallinarum* of hen faeces)
A bacterial species identified by metagenomic analyses. This species includes all bacteria with genomes that show ≥95% average nucleotide identity (ANI) to the type genome, which has been assigned the MAG ID ChiHjej8B7-16427 and which is available via NCBI BioSample SAMN15816758. The GC content of the type genome is 68.76% and the genome length is 3.6 Mbp.
**Description of *Candidatus* Dorea faecigallinarum sp. nov.**
*Candidatus* Dorea faecigallinarum (fae.ci.gal.li.na’rum. L. fem. n. *faex, faecis* excrement; L. fem. n. *gallina* hen; N.L. gen. n. *faecigallinarum* of hen faeces)
A bacterial species identified by metagenomic analyses. This species includes all bacteria with genomes that show ≥95% average nucleotide identity (ANI) to the type genome, which has been assigned the MAG ID ChiHjej12B11-29902 and which is available via NCBI BioSample SAMN15816646. The GC content of the type genome is 50.13% and the genome length is 2.0 Mbp.
**Description of *Candidatus* Dorea faecipullorum sp. nov.**
*Candidatus* Dorea faecipullorum (fae.ci.pul.lo’rum. L. fem. n. *faex, faecis* excrement; L. masc. n. *pullus* a young chicken; N.L. gen. n. *faecipullorum* of young chicken faeces)
A bacterial species identified by metagenomic analyses. This species includes all bacteria with genomes that show ≥95% average nucleotide identity (ANI) to the type genome, which has been assigned the MAG ID ChiGjej2B2-10896 and which is available via NCBI BioSample SAMN15816847. This is a new name for the alphanumeric GTDB species sp900543315. The GC content of the type genome is 45.40% and the genome length is 2.3 Mbp.
**Description of *Candidatus* Dorea gallistercoris sp. nov.**
*Candidatus* Dorea gallistercoris (gal.li.ster’co.ris. L. masc. n. *gallus* chicken; L. neut. n. *stercus* dung; N.L. gen. n. *gallistercoris* of chicken faeces)
A bacterial species identified by metagenomic analyses. This species includes all bacteria with genomes that show ≥95% average nucleotide identity (ANI) to the type genome, which has been assigned the MAG ID ChiSxjej1B13-11762 and which is available via NCBI BioSample SAMN15816753. The GC content of the type genome is 51.71% and the genome length is 2.2 Mbp.
**Description of *Candidatus* Dorea intestinavium sp. nov.**
*Candidatus* Dorea intestinavium (in.tes.tin.a’vi.um. L. neut. n. *intestinum* gut; L. fem. n. *avis* bird; N.L. gen. n. *intestinavium* of the gut of birds)
A bacterial species identified by metagenomic analyses. This species includes all bacteria with genomes that show ≥95% average nucleotide identity (ANI) to the type genome, which has been assigned the MAG ID CHK160-14747 and which is available via NCBI BioSample SAMN15816767. The GC content of the type genome is 35.91% and the genome length is 1.9 Mbp.
**Description of *Candidatus* Dorea intestinigallinarum sp. nov.**
*Candidatus* Dorea intestinigallinarum (in.tes.ti.ni.gal.li.na’rum. L. neut. n. *intestinum* gut; L. fem. n. *gallina* hen; N.L. gen. n. *intestinigallinarum* of the gut of the hens)
A bacterial species identified by metagenomic analyses. This species includes all bacteria with genomes that show ≥95% average nucleotide identity (ANI) to the type genome, which has been assigned the MAG ID CHK188-17839 and which is available via NCBI BioSample SAMN15816854. This is a new name for the alphanumeric GTDB species sp000765215. The GC content of the type genome is 54.58% and the genome length is 2.5 Mbp.
**Description of *Candidatus* Dorea merdavium sp. nov.**
*Candidatus* Dorea merdavium (merd.a’vi.um. L. fem. n. *merda* faeces; L. fem. n. *avis* bird; N.L. gen. n. *merdavium* of bird faeces)
A bacterial species identified by metagenomic analyses. This species includes all bacteria with genomes that show ≥95% average nucleotide identity (ANI) to the type genome, which has been assigned the MAG ID ChiSxjej1B13-1060 and which is available via NCBI BioSample SAMN15816851. This is a new name for the alphanumeric GTDB species sp900312975. The GC content of the type genome is 53.23% and the genome length is 2.0 Mbp.

**Table:** 

**Description of *Candidatus* Dorea stercoravium sp. nov.**
*Candidatus* Dorea stercoravium (ster.cor.a’vi.um. L. neut. n. *stercus* dung; L. fem. n. *avis* bird; N.L. gen. n. *stercoravium* of bird faeces)
A bacterial species identified by metagenomic analyses. This species includes all bacteria with genomes that show ≥95% average nucleotide identity (ANI) to the type genome, which has been assigned the MAG ID ChiSjej1B19-6982 and which is available via NCBI BioSample SAMN15816837. This is a new name for the alphanumeric GTDB species sp002160985. The GC content of the type genome is 55.00% and the genome length is 2.5 Mbp.
**Description of *Candidatus* Duodenibacillus intestinavium sp. nov.**
*Candidatus* Duodenibacillus intestinavium (in.tes.tin.a’vi.um. L. neut. n. *intestinum* gut; L. fem. n. *avis* bird; N.L. gen. n. *intestinavium* of the gut of birds)
A bacterial species identified by metagenomic analyses. This species includes all bacteria with genomes that show ≥95% average nucleotide identity (ANI) to the type genome, which has been assigned the MAG ID 2430 and which is available via NCBI BioSample SAMN15816841. This is a new name for the alphanumeric GTDB species sp900538905. The GC content of the type genome is 55.01% and the genome length is 1.8 Mbp.
**Description of *Candidatus* Duodenibacillus intestinigallinarum sp. nov.**
*Candidatus* Duodenibacillus intestinigallinarum (in.tes.ti.ni.gal.li.na’rum. L. neut. n. *intestinum* gut; L. fem. n. *gallina* hen; N.L. gen. n. *intestinigallinarum* of the gut of the hens)
A bacterial species identified by metagenomic analyses. This species includes all bacteria with genomes that show ≥95% average nucleotide identity (ANI) to the type genome, which has been assigned the MAG ID CHK1-2119 and which is available via NCBI BioSample SAMN15816840. This is a new name for the alphanumeric GTDB species sp003472385. The GC content of the type genome is 56.00% and the genome length is 2.0 Mbp.
**Description of *Candidatus* Egerieenecus gen. nov.**
*Candidatus* Egerieenecus (E.ge.ri.e.en.e’cus. L. fem. n. *egeries* dung; Gr. masc. *enoikos* inhabitant; N.L. masc. n. *Egerieenecus* a microbe associated with faeces)
A bacterial genus identified by metagenomic analyses. The genus includes all bacteria with genomes that show ≥60% average amino acid identity (AAI) to the type genome from the type species *Candidatus* Egerieenecus merdigallinarum. This is a name for the alphanumeric GTDB genus UMGS1600. This genus has been assigned by GTDB-Tk v1.3.0 working on GTDB Release 05-RS95 (Chaumeil et al., 2019; Parks et al., 2020) to the order *Christensenellales* and to the family *CAG-74*.
**Description of *Candidatus* Egerieenecus merdigallinarum sp. nov.**
*Candidatus* Egerieenecus merdigallinarum (mer.di.gal.li.na’rum. L. fem. n. *merda* faeces; L. fem. n. *gallina* hen; N.L. gen. n. *merdigallinarum* of hen faeces)
A bacterial species identified by metagenomic analyses. This species includes all bacteria with genomes that show ≥95% average nucleotide identity (ANI) to the type genome, which has been assigned the MAG ID ChiSxjej2B14-4419 and which is available via NCBI BioSample SAMN15817218. This is a new name for the alphanumeric GTDB species sp900553295. The GC content of the type genome is 60.15% and the genome length is 2.5 Mbp.
**Description of *Candidatus* Egerieicola gen. nov.**
*Candidatus* Egerieicola (E.ge.ri.e.i’co.la. L. fem. n. *egeries* dung; L. suff. *-cola* inhabitant of; N.L. fem. n. *Egerieicola* a microbe associated with faeces)
A bacterial genus identified by metagenomic analyses. The genus includes all bacteria with genomes that show ≥60% average amino acid identity (AAI) to the type genome from the type species *Candidatus* Egerieicola faecale. This is a name for the alphanumeric GTDB genus UBA1375. This genus has been assigned by GTDB-Tk v1.3.0 working on GTDB Release 05-RS95 (Chaumeil et al., 2019; Parks et al., 2020) to the order *Oscillospirales* and to the family *Ruminococcaceae*.
**Description of *Candidatus* Egerieicola faecalis sp. nov.**
*Candidatus* Egerieicola faecale (fae.ca’lis. L. fem. adj. *faecalis* of faeces)
A bacterial species identified by metagenomic analyses. This species includes all bacteria with genomes that show ≥95% average nucleotide identity (ANI) to the type genome, which has been assigned the MAG ID 4509 and which is available via NCBI BioSample SAMN15817200. This is a new name for the alphanumeric GTDB species sp002305795. The GC content of the type genome is 55.66% and the genome length is 1.8 Mbp.
**Description of *Candidatus* Egerieicola pullicola sp. nov.**
*Candidatus* Egerieicola pullicola (pul.li’co.la. L. masc. n. *pullus* a young chicken; L. suff. *-cola* inhabitant of; N.L. n. *pullicola* an inhabitant of young chickens)
A bacterial species identified by metagenomic analyses. This species includes all bacteria with genomes that show ≥95% average nucleotide identity (ANI) to the type genome, which has been assigned the MAG ID CHK184-25365 and which is available via NCBI BioSample SAMN15817017. The GC content of the type genome is 52.81% and the genome length is 1.9 Mbp.

**Table:** 

**Description of *Candidatus* Egerieimonas gen. nov.**
*Candidatus* Egerieimonas (E.ge.ri.e.i.mo’nas. L. fem. n. *egeries* dung; L. fem. n. *monas* a monad; N.L. fem. n. *Egerieimonas* a microbe associated with faeces)
A bacterial genus identified by metagenomic analyses. The genus includes all bacteria with genomes that show ≥60% average amino acid identity (AAI) to the type genome from the type species *Candidatus* Egerieimonas intestinavium. This is a name for the alphanumeric GTDB genus UMGS1472. This genus has been assigned by GTDB-Tk v1.3.0 working on GTDB Release 05-RS95 (Chaumeil et al., 2019; Parks et al., 2020) to the order *Lachnospirales* and to the family *Lachnospiraceae*.
**Description of *Candidatus* Egerieimonas faecigallinarum sp. nov.**
*Candidatus* Egerieimonas faecigallinarum (fae.ci.gal.li.na’rum. L. fem. n. *faex, faecis* excrement; L. fem. n. *gallina* hen; N.L. gen. n. *faecigallinarum* of hen faeces)
A bacterial species identified by metagenomic analyses. This species includes all bacteria with genomes that show ≥95% average nucleotide identity (ANI) to the type genome, which has been assigned the MAG ID CHK180-10209 and which is available via NCBI BioSample SAMN15817015. The GC content of the type genome is 51.75% and the genome length is 2.9 Mbp.
**Description of *Candidatus* Egerieimonas intestinavium sp. nov.**
*Candidatus* Egerieimonas intestinavium (in.tes.tin.a’vi.um. L. neut. n. *intestinum* gut; L. fem. n. *avis* bird; N.L. gen. n. *intestinavium* of the gut of birds)
A bacterial species identified by metagenomic analyses. This species includes all bacteria with genomes that show ≥95% average nucleotide identity (ANI) to the type genome, which has been assigned the MAG ID ChiSxjej1B13-7041 and which is available via NCBI BioSample SAMN15817079. The GC content of the type genome is 55.23% and the genome length is 2.6 Mbp.
**Description of *Candidatus* Egerieisoma gen. nov.**
*Candidatus* Egerieisoma (E.ge.ri.e.so’ma. L. fem. n. *egeries* dung; Gr. neut. n. *soma* a body; N.L. neut. n. *Egerieisoma* a microbe associated with faeces)
A bacterial genus identified by metagenomic analyses. The genus includes all bacteria with genomes that show ≥60% average amino acid identity (AAI) to the type genome from the type species *Candidatus* Egerieisoma faecipullorum. This is a name for the alphanumeric GTDB genus UMGS1537. This genus has been assigned by GTDB-Tk v1.3.0 working on GTDB Release 05-RS95 (Chaumeil et al., 2019; Parks et al., 2020) to the order *UBA1212* and to the family *UBA1255*.
**Description of *Candidatus* Egerieisoma faecipullorum sp. nov.**
*Candidatus* Egerieisoma faecipullorum (fae.ci.pul.lo’rum. L. fem. n. *faex, faecis* excrement; L. masc. n. *pullus* a young chicken; N.L. gen. n. *faecipullorum* of young chicken faeces)
A bacterial species identified by metagenomic analyses. This species includes all bacteria with genomes that show ≥95% average nucleotide identity (ANI) to the type genome, which has been assigned the MAG ID CHK195-4489 and which is available via NCBI BioSample SAMN15817230. This is a new name for the alphanumeric GTDB species sp900543695. The GC content of the type genome is 50.69% and the genome length is 2.0 Mbp.
**Description of *Candidatus* Egerieousia gen. nov.**
*Candidatus* Egerieousia (E.ge.ri.e.ou’si.a. L. fem. n. *egeries* dung; Gr. fem. n. *ousia* an essence; N.L. fem. n. *Egerieousia* a microbe associated with faeces)
A bacterial genus identified by metagenomic analyses. The genus includes all bacteria with genomes that show ≥60% average amino acid identity (AAI) to the type genome from the type species *Candidatus* Egerieousia excrementavium. This is a name for the alphanumeric GTDB genus UBA1232. This genus has been assigned by GTDB-Tk v1.3.0 working on GTDB Release 05-RS95 (Chaumeil et al., 2019; Parks et al., 2020) to the order *Bacteroidales* and to the family *UBA932*.
**Description of *Candidatus* Egerieousia excrementavium sp. nov.**
*Candidatus* Egerieousia excrementavium (ex.cre.ment.a’vi.um. L. neut. n. *excrementum* excrement; L. fem. n. *avis* bird; N.L. gen. n. *excrementavium* of bird excrement)
A bacterial species identified by metagenomic analyses. This species includes all bacteria with genomes that show ≥95% average nucleotide identity (ANI) to the type genome, which has been assigned the MAG ID 15467 and which is available via NCBI BioSample SAMN15817149. The GC content of the type genome is 46.96% and the genome length is 1.5 Mbp.
**Description of *Candidatus* Eisenbergiella intestinigallinarum sp. nov.**
*Candidatus* Eisenbergiella intestinigallinarum (in.tes.ti.ni.gal.li.na’rum. L. neut. n. *intestinum* gut; L. fem. n. *gallina* hen; N.L. gen. n. *intestinigallinarum* of the gut of the hens)
A bacterial species identified by metagenomic analyses. This species includes all bacteria with genomes that show ≥95% average nucleotide identity (ANI) to the type genome, which has been assigned the MAG ID ChiBcec1-1630 and which is available via NCBI BioSample SAMN15816806. This is a new name for the alphanumeric GTDB species sp900544445. The GC content of the type genome is 53.15% and the genome length is 3.4 Mbp.

**Table:** 

**Description of *Candidatus* Eisenbergiella intestinipullorum sp. nov.**
*Candidatus* Eisenbergiella intestinipullorum (in.tes.ti.ni.pul.lo’rum. L. neut. n. *intestinum* gut; L. masc. n. *pullus* a young chicken; N.L. gen. n. *intestinipullorum* of the gut of young chickens)
A bacterial species identified by metagenomic analyses. This species includes all bacteria with genomes that show ≥95% average nucleotide identity (ANI) to the type genome, which has been assigned the MAG ID CHK177-9469 and which is available via NCBI BioSample SAMN15816580. The GC content of the type genome is 54.63% and the genome length is 3.5 Mbp.
**Description of *Candidatus* Eisenbergiella merdavium sp. nov.**
*Candidatus* Eisenbergiella merdavium (merd.a’vi.um. L. fem. n. *merda* faeces; L. fem. n. *avis* bird; N.L. gen. n. *merdavium* of bird faeces)
A bacterial species identified by metagenomic analyses. This species includes all bacteria with genomes that show ≥95% average nucleotide identity (ANI) to the type genome, which has been assigned the MAG ID USAMLcec2-132 and which is available via NCBI BioSample SAMN15816641. The GC content of the type genome is 54.07% and the genome length is 4.2 Mbp.
**Description of *Candidatus* Eisenbergiella merdigallinarum sp. nov.**
*Candidatus* Eisenbergiella merdigallinarum (mer.di.gal.li.na’rum. L. fem. n. *merda* faeces; L. fem. n. *gallina* hen; N.L. gen. n. *merdigallinarum* of hen faeces)
A bacterial species identified by metagenomic analyses. This species includes all bacteria with genomes that show ≥95% average nucleotide identity (ANI) to the type genome, which has been assigned the MAG ID USAMLcec3-2134 and which is available via NCBI BioSample SAMN15816643. The GC content of the type genome is 57.03% and the genome length is 3.2 Mbp.
**Description of *Candidatus* Eisenbergiella merdipullorum sp. nov.**
*Candidatus* Eisenbergiella merdipullorum (mer.di.pul.lo’rum. L. fem. n. *merda* faeces; L. masc. n. *pullus* a young chicken; N.L. gen. n. *merdipullorum* of the faeces of young chickens)
A bacterial species identified by metagenomic analyses. This species includes all bacteria with genomes that show ≥95% average nucleotide identity (ANI) to the type genome, which has been assigned the MAG ID CHK179-7159 and which is available via NCBI BioSample SAMN15816597. The GC content of the type genome is 51.92% and the genome length is 3.5 Mbp.
**Description of *Candidatus* Eisenbergiella pullicola sp. nov.**
*Candidatus* Eisenbergiella pullicola (pul.li’co.la. L. masc. n. *pullus* a young chicken; L. suff. *-cola* inhabitant of; N.L. n. *pullicola* an inhabitant of young chickens)
A bacterial species identified by metagenomic analyses. This species includes all bacteria with genomes that show ≥95% average nucleotide identity (ANI) to the type genome, which has been assigned the MAG ID CHK197-24098 and which is available via NCBI BioSample SAMN15816836. This is a new name for the alphanumeric GTDB species sp003343625. The GC content of the type genome is 54.54% and the genome length is 2.6 Mbp.
**Description of *Candidatus* Eisenbergiella pullistercoris sp. nov.**
*Candidatus* Eisenbergiella pullistercoris (pul.li.ster’co.ris. L. masc. n. *pullus* a young chicken; L. neut. n. *stercus* dung; N.L. gen. n. *pullistercoris* of young chicken faeces)
A bacterial species identified by metagenomic analyses. This species includes all bacteria with genomes that show ≥95% average nucleotide identity (ANI) to the type genome, which has been assigned the MAG ID ChiSxjej3B15-24422 and which is available via NCBI BioSample SAMN15816711. The GC content of the type genome is 56.23% and the genome length is 3.3 Mbp.
**Description of *Candidatus* Eisenbergiella stercoravium sp. nov.**
*Candidatus* Eisenbergiella stercoravium (ster.cor.a’vi.um. L. neut. n. *stercus* dung; L. fem. n. *avis* bird; N.L. gen. n. *stercoravium* of bird faeces)
A bacterial species identified by metagenomic analyses. This species includes all bacteria with genomes that show ≥95% average nucleotide identity (ANI) to the type genome, which has been assigned the MAG ID USAMLcec4-2206 and which is available via NCBI BioSample SAMN15816624. The GC content of the type genome is 51.92% and the genome length is 3.9 Mbp.
**Description of *Candidatus* Eisenbergiella stercorigallinarum sp. nov.**
*Candidatus* Eisenbergiella stercorigallinarum (ster.co.ri.gal.li.na’rum. L. neut. n. *stercus* dung; L. fem. n. *gallina* hen; N.L. gen. n. *stercorigallinarum* of hen faeces)
A bacterial species identified by metagenomic analyses. This species includes all bacteria with genomes that show ≥95% average nucleotide identity (ANI) to the type genome, which has been assigned the MAG ID ChiHjej8B7-25341 and which is available via NCBI BioSample SAMN15816792. The GC content of the type genome is 55.79% and the genome length is 2.8 Mbp.

**Table:** 

**Description of *Candidatus* Enterenecus gen. nov.**
*Candidatus* Enterenecus (En.ter.en.e’cus. Gr. neut. n. *enteron* the gut; Gr. masc. *enoikos* inhabitant; N.L. masc. n. *Enterenecus* a microbe associated with the intestines)
A bacterial genus identified by metagenomic analyses. The genus includes all bacteria with genomes that show ≥60% average amino acid identity (AAI) to the type genome from the type species *Candidatus* Enterenecus merdae. This is a name for the alphanumeric GTDB genus UBA9475. This genus has been assigned by GTDB-Tk v1.3.0 working on GTDB Release 05-RS95 (Chaumeil et al., 2019; Parks et al., 2020) to the order *Oscillospirales* and to the family *Oscillospiraceae*.
**Description of *Candidatus* Enterenecus avicola sp. nov.**
*Candidatus* Enterenecus avicola (a.vi’co.la. L. fem. n. *avis* bird; L. suff. *-cola* inhabitant of; N.L. n. *avicola* inhabitant of birds)
A bacterial species identified by metagenomic analyses. This species includes all bacteria with genomes that show ≥95% average nucleotide identity (ANI) to the type genome, which has been assigned the MAG ID 153 and which is available via NCBI BioSample SAMN15817108. The GC content of the type genome is 60.43% and the genome length is 1.9 Mbp.
**Description of *Candidatus* Enterenecus avistercoris sp. nov.**
*Candidatus* Enterenecus avistercoris (a.vi.ster’co.ris. L. fem. n. *avis* bird; L. neut. n. *stercus* dung; N.L. gen. n. *avistercoris* of bird faeces)
A bacterial species identified by metagenomic analyses. This species includes all bacteria with genomes that show ≥95% average nucleotide identity (ANI) to the type genome, which has been assigned the MAG ID ChiSxjej3B15-11837 and which is available via NCBI BioSample SAMN15817165. The GC content of the type genome is 64.30% and the genome length is 1.5 Mbp.
**Description of *Candidatus* Enterenecus faecium sp. nov.**
*Candidatus* Enterenecus faecium (fae’ci.um. L. fem. n. *faex, faecis* excrement; L. masc. gen. pl. n. *faecium* of faeces)
A bacterial species identified by metagenomic analyses. This species includes all bacteria with genomes that show ≥95% average nucleotide identity (ANI) to the type genome, which has been assigned the MAG ID ChiGjej2B2-12916 and which is available via NCBI BioSample SAMN15817211. This is a new name for the alphanumeric GTDB species sp002161675. The GC content of the type genome is 60.00% and the genome length is 2.0 Mbp.
**Description of *Candidatus* Enterenecus merdae sp. nov.**
*Candidatus* Enterenecus merdae (mer’dae. L. gen. n. *merdae* of faeces)
A bacterial species identified by metagenomic analyses. This species includes all bacteria with genomes that show ≥95% average nucleotide identity (ANI) to the type genome, which has been assigned the MAG ID ChiHcolR17-2730 and which is available via NCBI BioSample SAMN15817102. The GC content of the type genome is 63.48% and the genome length is 1.7 Mbp.
**Description of *Candidatus* Enterenecus stercoripullorum sp. nov.**
*Candidatus* Enterenecus stercoripullorum (ster.co.ri.pul.lo’rum. L. neut. n. *stercus* dung; L. masc. n. *pullus* a young chicken; N.L. gen. n. *stercoripullorum* of the faceces of young chickens)
A bacterial species identified by metagenomic analyses. This species includes all bacteria with genomes that show ≥95% average nucleotide identity (ANI) to the type genome, which has been assigned the MAG ID 3668 and which is available via NCBI BioSample SAMN15817106. The GC content of the type genome is 60.97% and the genome length is 1.8 Mbp.
**Description of *Candidatus* Enterocloster excrementigallinarum sp. nov.**
*Candidatus* Enterocloster excrementigallinarum (ex.cre.men.ti.gal.li.na’rum. L. neut. n. *excrementum* excrement; L. fem. n. *gallina* hen; N.L. gen. n. *excrementigallinarum* of hen excrement)
A bacterial species identified by metagenomic analyses. This species includes all bacteria with genomes that show ≥95% average nucleotide identity (ANI) to the type genome, which has been assigned the MAG ID CHK198-12963 and which is available via NCBI BioSample SAMN15816811. This is a new name for the alphanumeric GTDB species sp900547035. The GC content of the type genome is 51.32% and the genome length is 3.1 Mbp.
**Description of *Candidatus* Enterocloster excrementipullorum sp. nov.**
*Candidatus* Enterocloster excrementipullorum (ex.cre.men.ti.pul.lo’rum. L. neut. n. *excrementum* excrement; L. masc. n. *pullus* a young chicken; N.L. gen. n. *excrementipullorum* of young chicken excrement)
A bacterial species identified by metagenomic analyses. This species includes all bacteria with genomes that show ≥95% average nucleotide identity (ANI) to the type genome, which has been assigned the MAG ID CHK180-15479 and which is available via NCBI BioSample SAMN15816584. The GC content of the type genome is 53.85% and the genome length is 2.9 Mbp.

**Table:** 

**Description of *Candidatus* Enterocloster faecavium sp. nov.**
*Candidatus* Enterocloster faecavium (faec.a’vi.um. L. fem. n. *faex, faecis* excrement; L. fem. n. *avis* bird; N.L. gen. n. *faecavium* of bird faeces)
A bacterial species identified by metagenomic analyses. This species includes all bacteria with genomes that show ≥95% average nucleotide identity (ANI) to the type genome, which has been assigned the MAG ID CHK188-4685 and which is available via NCBI BioSample SAMN15816596. The GC content of the type genome is 52.23% and the genome length is 2.8 Mbp.
**Description of *Candidatus* Enterococcus avicola sp. nov.**
*Candidatus* Enterococcus avicola (a.vi’co.la. L. fem. n. *avis* bird; L. suff. *-cola* inhabitant of; N.L. n. *avicola* inhabitant of birds)
A bacterial species identified by metagenomic analyses. This species includes all bacteria with genomes that show ≥95% average nucleotide identity (ANI) to the type genome, which has been assigned the MAG ID CHK172-16539 and which is available via NCBI BioSample SAMN15816900. Although GTDB has assigned this species to the genus it calls Enterococcus_I, this genus designation cannot be incorporated into a well-formed binomial, so in naming this species, we have used the current validly published name for the genus. The GC content of the type genome is 36.87% and the genome length is 2.2 Mbp.
**Description of *Candidatus* Enterococcus stercoravium sp. nov.**
*Candidatus* Enterococcus stercoravium (ster.cor.a’vi.um. L. neut. n. *stercus* dung; L. fem. n. *avis* bird; N.L. gen. n. *stercoravium* of bird faeces)
A bacterial species identified by metagenomic analyses. This species includes all bacteria with genomes that show ≥95% average nucleotide identity (ANI) to the type genome, which has been assigned the MAG ID CHK172-14336 and which is available via NCBI BioSample SAMN15816907. Although GTDB has assigned this species to the genus it calls Enterococcus_C, this genus designation cannot be incorporated into a well-formed binomial, so in naming this species, we have used the current validly published name for the genus. The GC content of the type genome is 44.16% and the genome length is 2.3 Mbp.
**Description of *Candidatus* Enterococcus stercoripullorum sp. nov.**
*Candidatus* Enterococcus stercoripullorum (ster.co.ri.pul.lo’rum. L. neut. n. *stercus* dung; L. masc. n. *pullus* a young chicken; N.L. gen. n. *stercoripullorum* of the faceces of young chickens)
A bacterial species identified by metagenomic analyses. This species includes all bacteria with genomes that show ≥95% average nucleotide identity (ANI) to the type genome, which has been assigned the MAG ID ChiHjej12B11-924 and which is available via NCBI BioSample SAMN15816914. Although GTDB has assigned this species to the genus it calls Enterococcus_E, this genus designation cannot be incorporated into a well-formed binomial, so in naming this species, we have used the current validly published name for the genus. The GC content of the type genome is 36.20% and the genome length is 2.3 Mbp.
**Description of *Candidatus* Enterocola gen. nov.**
*Candidatus* Enterocola (En.te.ro’co.la. Gr. neut. n. *enteron* the gut; L. suff. *-cola* inhabitant of; N.L. fem. n. *Enterocola* a microbe associated with the gut)
A bacterial genus identified by metagenomic analyses. The genus includes all bacteria with genomes that show ≥60% average amino acid identity (AAI) to the type genome from the type species *Candidatus* Enterocola intestinipullorum. This is a name for the alphanumeric GTDB genus RUG163. This genus has been assigned by GTDB-Tk v1.3.0 working on GTDB Release 05-RS95 (Chaumeil et al., 2019; Parks et al., 2020) to the order *Bacteroidales* and to the family *Paludibacteraceae*.
**Description of *Candidatus* Enterocola intestinipullorum sp. nov.**
*Candidatus* Enterocola intestinipullorum (in.tes.ti.ni.pul.lo’rum. L. neut. n. *intestinum* gut; L. masc. n. *pullus* a young chicken; N.L. gen. n. *intestinipullorum* of the gut of young chickens)
A bacterial species identified by metagenomic analyses. This species includes all bacteria with genomes that show ≥95% average nucleotide identity (ANI) to the type genome, which has been assigned the MAG ID D3-1215 and which is available via NCBI BioSample SAMN15817113. The GC content of the type genome is 47.46% and the genome length is 1.8 Mbp.
**Description of *Candidatus* Enteromonas gen. nov.**
*Candidatus* Enteromonas (En.te.ro.mo’nas. Gr. neut. n. *enteron* the gut; L. fem. n. *monas* a monad; N.L. fem. n. *Enteromonas* a microbe associated with the intestines)
A bacterial genus identified by metagenomic analyses. The genus includes all bacteria with genomes that show ≥60% average amino acid identity (AAI) to the type genome from the type species *Candidatus* Enteromonas pullistercoris. This is a name for the alphanumeric GTDB genus UBA733. This genus has been assigned by GTDB-Tk v1.3.0 working on GTDB Release 05-RS95 (Chaumeil et al., 2019; Parks et al., 2020) to the order *RFN20* and to the family *CAG-826*.

**Table:** 

**Description of *Candidatus* Enteromonas pullicola sp. nov.**
*Candidatus* Enteromonas pullicola (pul.li’co.la. L. masc. n. *pullus* a young chicken; L. suff. *-cola* inhabitant of; N.L. n. *pullicola* an inhabitant of young chickens)
A bacterial species identified by metagenomic analyses. This species includes all bacteria with genomes that show ≥95% average nucleotide identity (ANI) to the type genome, which has been assigned the MAG ID ChiGjej1B1-22543 and which is available via NCBI BioSample SAMN15817133. The GC content of the type genome is 57.43% and the genome length is 1.2 Mbp.
**Description of *Candidatus* Enteromonas pullistercoris sp. nov.**
*Candidatus* Enteromonas pullistercoris (pul.li.ster’co.ris. L. masc. n. *pullus* a young chicken; L. neut. n. *stercus* dung; N.L. gen. n. *pullistercoris* of young chicken faeces)
A bacterial species identified by metagenomic analyses. This species includes all bacteria with genomes that show ≥95% average nucleotide identity (ANI) to the type genome, which has been assigned the MAG ID 17113 and which is available via NCBI BioSample SAMN15817142. The GC content of the type genome is 53.99% and the genome length is 1.4 Mbp.
**Description of *Candidatus* Enterosoma gen. nov.**
*Candidatus* Enterosoma (En.te.ro.so’ma. Gr. neut. n. *enteron* the gut; Gr. neut. n. *soma* a body; N.L. neut. n. *Enterosoma* a microbe associated with the intestines)
A bacterial genus identified by metagenomic analyses. The genus includes all bacteria with genomes that show ≥60% average amino acid identity (AAI) to the type genome from the type species *Candidatus* Enterosoma merdigallinarum. This is a name for the alphanumeric GTDB genus UBA7642. This genus has been assigned by GTDB-Tk v1.3.0 working on GTDB Release 05-RS95 (Chaumeil et al., 2019; Parks et al., 2020) to the order *RFN20* and to the family *CAG-288*.
**Description of *Candidatus* Enterosoma merdigallinarum sp. nov.**
*Candidatus* Enterosoma merdigallinarum (mer.di.gal.li.na’rum. L. fem. n. *merda* faeces; L. fem. n. *gallina* hen; N.L. gen. n. *merdigallinarum* of hen faeces)
A bacterial species identified by metagenomic analyses. This species includes all bacteria with genomes that show ≥95% average nucleotide identity (ANI) to the type genome, which has been assigned the MAG ID 33044 and which is available via NCBI BioSample SAMN15817141. The GC content of the type genome is 51.22% and the genome length is 1.4 Mbp.
**Description of *Candidatus* Enterousia gen. nov.**
*Candidatus* Enterousia (En.ter.ou’si.a. Gr. neut. n. *enteron* the gut; Gr. fem. n. *ousia* an essence; N.L. fem. n. *Enterousia* a microbe associated with the intestines)
A bacterial genus identified by metagenomic analyses. The genus includes all bacteria with genomes that show ≥60% average amino acid identity (AAI) to the type genome from the type species *Candidatus* Enterousia excrementavium. This is a name for the alphanumeric GTDB genus Rs-D84. This genus has been assigned by GTDB-Tk v1.3.0 working on GTDB Release 05-RS95 (Chaumeil et al., 2019; Parks et al., 2020) to the order *Rs-D84* and to the family *Rs-D84*.
**Description of *Candidatus* Enterousia avicola sp. nov.**
*Candidatus* Enterousia avicola (a.vi’co.la. L. fem. n. *avis* bird; L. suff. *-cola* inhabitant of; N.L. n. *avicola* inhabitant of birds)
A bacterial species identified by metagenomic analyses. This species includes all bacteria with genomes that show ≥95% average nucleotide identity (ANI) to the type genome, which has been assigned the MAG ID CHK136-897 and which is available via NCBI BioSample SAMN15817144. The GC content of the type genome is 39.01% and the genome length is 0.9 Mbp.
**Description of *Candidatus* Enterousia avistercoris sp. nov.**
*Candidatus* Enterousia avistercoris (a.vi.ster’co.ris. L. fem. n. *avis* bird; L. neut. n. *stercus* dung; N.L. gen. n. *avistercoris* of bird faeces)
A bacterial species identified by metagenomic analyses. This species includes all bacteria with genomes that show ≥95% average nucleotide identity (ANI) to the type genome, which has been assigned the MAG ID 8207 and which is available via NCBI BioSample SAMN15817150. The GC content of the type genome is 43.79% and the genome length is 0.8 Mbp.
**Description of *Candidatus* Enterousia excrementavium sp. nov.**
*Candidatus* Enterousia excrementavium (ex.cre.ment.a’vi.um. L. neut. n. *excrementum* excrement; L. fem. n. *avis* bird; N.L. gen. n. *excrementavium* of bird excrement)
A bacterial species identified by metagenomic analyses. This species includes all bacteria with genomes that show ≥95% average nucleotide identity (ANI) to the type genome, which has been assigned the MAG ID B1-16210 and which is available via NCBI BioSample SAMN15817158. The GC content of the type genome is 44.26% and the genome length is 0.9 Mbp.

**Table:** 

**Description of *Candidatus* Enterousia intestinigallinarum sp. nov.**
*Candidatus* Enterousia intestinigallinarum (in.tes.ti.ni.gal.li.na’rum. L. neut. n. *intestinum* gut; L. fem. n. *gallina* hen; N.L. gen. n. *intestinigallinarum* of the gut of the hens)
A bacterial species identified by metagenomic analyses. This species includes all bacteria with genomes that show ≥95% average nucleotide identity (ANI) to the type genome, which has been assigned the MAG ID ChiGjej3B3-5194 and which is available via NCBI BioSample SAMN15817183. This is a new name for the alphanumeric GTDB species sp900546185. The GC content of the type genome is 45.89% and the genome length is 0.9 Mbp.
**Description of *Candidatus* Erysipelatoclostridium merdavium sp. nov.**
*Candidatus* Erysipelatoclostridium merdavium (merd.a’vi.um. L. fem. n. *merda* faeces; L. fem. n. *avis* bird; N.L. gen. n. *merdavium* of bird faeces)
A bacterial species identified by metagenomic analyses. This species includes all bacteria with genomes that show ≥95% average nucleotide identity (ANI) to the type genome, which has been assigned the MAG ID ChiGjej1B1-14440 and which is available via NCBI BioSample SAMN15816860. This is a new name for the alphanumeric GTDB species sp002160495. The GC content of the type genome is 29.32% and the genome length is 2.6 Mbp.
**Description of *Candidatus* Eubacterium avistercoris sp. nov.**
*Candidatus* Eubacterium avistercoris (a.vi.ster’co.ris. L. fem. n. *avis* bird; L. neut. n. *stercus* dung; N.L. gen. n. *avistercoris* of bird faeces)
A bacterial species identified by metagenomic analyses. This species includes all bacteria with genomes that show ≥95% average nucleotide identity (ANI) to the type genome, which has been assigned the MAG ID CHK192-9172 and which is available via NCBI BioSample SAMN15816888. Although GTDB has assigned this species to the genus it calls Eubacterium_I, this genus designation cannot be incorporated into a well-formed binomial, so in naming this species, we have used the current validly published name for the genus. The GC content of the type genome is 45.90% and the genome length is 2.6 Mbp.
**Description of *Candidatus* Eubacterium faecale sp. nov.**
*Candidatus* Eubacterium faecale (fae.ca’le. L. neut. adj. *faecale* of faeces)
A bacterial species identified by metagenomic analyses. This species includes all bacteria with genomes that show ≥95% average nucleotide identity (ANI) to the type genome, which has been assigned the MAG ID CHK188-16595 and which is available via NCBI BioSample SAMN15816917. This is a new name for the alphanumeric GTDB species sp000431535. Although GTDB has assigned this species to the genus it calls Eubacterium_R, this genus designation cannot be incorporated into a well-formed binomial, so in naming this species, we have used the current validly published name for the genus. The GC content of the type genome is 46.56% and the genome length is 1.8 Mbp.
**Description of *Candidatus* Eubacterium faecavium sp. nov.**
*Candidatus* Eubacterium faecavium (faec.a’vi.um. L. fem. n. *faex, faecis* excrement; L. fem. n. *avis* bird; N.L. gen. n. *faecavium* of bird faeces)
A bacterial species identified by metagenomic analyses. This species includes all bacteria with genomes that show ≥95% average nucleotide identity (ANI) to the type genome, which has been assigned the MAG ID ChiHecec3B27-3607 and which is available via NCBI BioSample SAMN15816921. This is a new name for the alphanumeric GTDB species sp900539845. Although GTDB has assigned this species to the genus it calls Eubacterium_R, this genus designation cannot be incorporated into a well-formed binomial, so in naming this species, we have used the current validly published name for the genus. The GC content of the type genome is 45.56% and the genome length is 1.9 Mbp.
**Description of *Candidatus* Eubacterium faecigallinarum sp. nov.**
*Candidatus* Eubacterium faecigallinarum (fae.ci.gal.li.na’rum. L. fem. n. *faex, faecis* excrement; L. fem. n. *gallina* hen; N.L. gen. n. *faecigallinarum* of hen faeces)
A bacterial species identified by metagenomic analyses. This species includes all bacteria with genomes that show ≥95% average nucleotide identity (ANI) to the type genome, which has been assigned the MAG ID 8396 and which is available via NCBI BioSample SAMN15816904. Although GTDB has assigned this species to the genus it calls Eubacterium_R, this genus designation cannot be incorporated into a well-formed binomial, so in naming this species, we have used the current validly published name for the genus. The GC content of the type genome is 43.37% and the genome length is 1.6 Mbp.
**Description of *Candidatus* Eubacterium faecipullorum sp. nov.**
*Candidatus* Eubacterium faecipullorum (fae.ci.pul.lo’rum. L. fem. n. *faex, faecis* excrement; L. masc. n. *pullus* a young chicken; N.L. gen. n. *faecipullorum* of young chicken faeces)
A bacterial species identified by metagenomic analyses. This species includes all bacteria with genomes that show ≥95% average nucleotide identity (ANI) to the type genome, which has been assigned the MAG ID 421 and which is available via NCBI BioSample SAMN15816928. This is a new name for the alphanumeric GTDB species sp900546785. Although GTDB has assigned this species to the genus it calls Eubacterium_R, this genus designation cannot be incorporated into a well-formed binomial, so in naming this species, we have used the current validly published name for the genus. The GC content of the type genome is 47.26% and the genome length is 1.9 Mbp.

**Table:** 

**Description of *Candidatus* Eubacterium pullicola sp. nov.**
*Candidatus* Eubacterium pullicola (pul.li’co.la. L. masc. n. *pullus* a young chicken; L. suff. *-cola* inhabitant of; N.L. n. *pullicola* inhabitant of young chicken)
A bacterial species identified by metagenomic analyses. This species includes all bacteria with genomes that show ≥95% average nucleotide identity (ANI) to the type genome, which has been assigned the MAG ID ChiHjej12B11-11929 and which is available via NCBI BioSample SAMN15816916. This is a new name for the alphanumeric GTDB species sp900540015. Although GTDB has assigned this species to the genus it calls Eubacterium_M, this genus designation cannot be incorporated into a well-formed binomial, so in naming this species, we have used the current validly published name for the genus. The GC content of the type genome is 41.55% and the genome length is 1.2 Mbp.
**Description of *Candidatus* Evtepia excrementipullorum sp. nov.**
*Candidatus* Evtepia excrementipullorum (ex.cre.men.ti.pul.lo’rum. L. neut. n. *excrementum* excrement; L. masc. n. *pullus* a young chicken; N.L. gen. n. *excrementipullorum* of young chicken excrement)
A bacterial species identified by metagenomic analyses. This species includes all bacteria with genomes that show ≥95% average nucleotide identity (ANI) to the type genome, which has been assigned the MAG ID ChiSjej3B21-3892 and which is available via NCBI BioSample SAMN15816827. This is a new name for the alphanumeric GTDB species sp900546255. The GC content of the type genome is 63.06% and the genome length is 2.0 Mbp.
**Description of *Candidatus* Evtepia faecavium sp. nov.**
*Candidatus* Evtepia faecavium (faec.a’vi.um. L. fem. n. *faex, faecis* excrement; L. fem. n. *avis* bird; N.L. gen. n. *faecavium* of bird faeces)
A bacterial species identified by metagenomic analyses. This species includes all bacteria with genomes that show ≥95% average nucleotide identity (ANI) to the type genome, which has been assigned the MAG ID ChiHecec3B27-8621 and which is available via NCBI BioSample SAMN15816713. The GC content of the type genome is 65.40% and the genome length is 2.0 Mbp.
**Description of *Candidatus* Evtepia faecigallinarum sp. nov.**
*Candidatus* Evtepia faecigallinarum (fae.ci.gal.li.na’rum. L. fem. n. *faex, faecis* excrement; L. fem. n. *gallina* hen; N.L. gen. n. *faecigallinarum* of hen faeces)
A bacterial species identified by metagenomic analyses. This species includes all bacteria with genomes that show ≥95% average nucleotide identity (ANI) to the type genome, which has been assigned the MAG ID ChiHcec3-601 and which is available via NCBI BioSample SAMN15816724. The GC content of the type genome is 63.50% and the genome length is 2.4 Mbp.
**Description of *Candidatus* Excrementavichristensenella gen. nov.**
*Candidatus* Excrementavichristensenella (Ex.cre.ment.a.vi.chris.ten.sen.el’la. L. neut. n. *excrementum* excrement; L. fem. n. *avis* bird; N.L. fem. n. *Christensenella a genus* name; N.L. fem n. *Excrementavichristensenella* a genus related to the genus *Christensenella* but distinct from it and found in poultry faeces)
A bacterial genus identified by metagenomic analyses. The genus includes all bacteria with genomes that show ≥60% average amino acid identity (AAI) to the type genome from the type species *Candidatus* Excrementavichristensenella intestinipullorum. This genus has been assigned by GTDB-Tk v1.3.0 working on GTDB Release 05-RS95 (Chaumeil et al., 2019; Parks et al., 2020) to the order *Christensenellales* and to the family *CAG-74*.
**Description of *Candidatus* Excrementavichristensenella intestinipullorum sp. nov.**
*Candidatus* Excrementavichristensenella intestinipullorum (in.tes.ti.ni.pul.lo’rum. L. neut. n. *intestinum* gut; L. masc. n. *pullus* a young chicken; N.L. gen. n. *intestinipullorum* of the gut of young chickens)
A bacterial species identified by metagenomic analyses. This species includes all bacteria with genomes that show ≥95% average nucleotide identity (ANI) to the type genome, which has been assigned the MAG ID ChiGjej2B2-1688 and which is available via NCBI BioSample SAMN15816955. The GC content of the type genome is 62.99% and the genome length is 2.8 Mbp.
**Description of *Candidatus* Faecalibacterium avium sp. nov.**
*Candidatus* Faecalibacterium avium (a’vi.um. L. fem. pl. n. *avium* of birds)
A bacterial species identified by metagenomic analyses. This species includes all bacteria with genomes that show ≥95% average nucleotide identity (ANI) to the type genome, which has been assigned the MAG ID CHK182-10647 and which is available via NCBI BioSample SAMN15816876. This is a new name for the alphanumeric GTDB species sp002160915. The GC content of the type genome is 62.96% and the genome length is 2.2 Mbp.
**Description of *Candidatus* Faecalibacterium faecigallinarum sp. nov.**
*Candidatus* Faecalibacterium faecigallinarum (fae.ci.gal.li.na’rum. L. fem. n. *faex, faecis* excrement; L. fem. n. *gallina* hen; N.L. gen. n. *faecigallinarum* of hen faeces)
A bacterial species identified by metagenomic analyses. This species includes all bacteria with genomes that show ≥95% average nucleotide identity (ANI) to the type genome, which has been assigned the MAG ID ChiSjej5B23-2810 and which is available via NCBI BioSample SAMN15816583. The GC content of the type genome is 63.38% and the genome length is 2.1 Mbp.

**Table:** 

**Description of *Candidatus* Faecalibacterium faecipullorum sp. nov.**
*Candidatus* Faecalibacterium faecipullorum (fae.ci.pul.lo’rum. L. fem. n. *faex, faecis* excrement; L. masc. n. *pullus* a young chicken; N.L. gen. n. *faecipullorum* of young chicken faeces)
A bacterial species identified by metagenomic analyses. This species includes all bacteria with genomes that show ≥95% average nucleotide identity (ANI) to the type genome, which has been assigned the MAG ID ChiHjej9B8-13557 and which is available via NCBI BioSample SAMN15816651. The GC content of the type genome is 65.75% and the genome length is 2.1 Mbp.
**Description of *Candidatus* Faecalibacterium gallistercoris sp. nov.**
*Candidatus* Faecalibacterium gallistercoris (gal.li.ster’co.ris. L. masc. n. *gallus* chicken; L. neut. n. *stercus* dung; N.L. gen. n. *gallistercoris* of chicken faeces)
A bacterial species identified by metagenomic analyses. This species includes all bacteria with genomes that show ≥95% average nucleotide identity (ANI) to the type genome, which has been assigned the MAG ID ChiBcec16-3735 and which is available via NCBI BioSample SAMN15816605. The GC content of the type genome is 64.68% and the genome length is 2.1 Mbp.
**Description of *Candidatus* Faecalibacterium intestinavium sp. nov.**
*Candidatus* Faecalibacterium intestinavium (in.tes.tin.a’vi.um. L. neut. n. *intestinum* gut; L. fem. n. *avis* bird; N.L. gen. n. *intestinavium* of the gut of birds)
A bacterial species identified by metagenomic analyses. This species includes all bacteria with genomes that show ≥95% average nucleotide identity (ANI) to the type genome, which has been assigned the MAG ID 742 and which is available via NCBI BioSample SAMN15816744. The GC content of the type genome is 61.60% and the genome length is 1.8 Mbp.
**Description of *Candidatus* Faecalibacterium intestinigallinarum sp. nov.**
*Candidatus* Faecalibacterium intestinigallinarum (in.tes.ti.ni.gal.li.na’rum. L. neut. n. *intestinum* gut; L. fem. n. *gallina* hen; N.L. gen. n. *intestinigallinarum* of the gut of the hens)
A bacterial species identified by metagenomic analyses. This species includes all bacteria with genomes that show ≥95% average nucleotide identity (ANI) to the type genome, which has been assigned the MAG ID ChiHcolR34-3080 and which is available via NCBI BioSample SAMN15816770. The GC content of the type genome is 64.34% and the genome length is 2.1 Mbp.
**Description of *Candidatus* Faecalibacterium intestinipullorum sp. nov.**
*Candidatus* Faecalibacterium intestinipullorum (in.tes.ti.ni.pul.lo’rum. L. neut. n. *intestinum* gut; L. masc. n. *pullus* a young chicken; N.L. gen. n. *intestinipullorum* of the gut of young chickens)
A bacterial species identified by metagenomic analyses. This species includes all bacteria with genomes that show ≥95% average nucleotide identity (ANI) to the type genome, which has been assigned the MAG ID ChiHcolR21-11242 and which is available via NCBI BioSample SAMN15816785. The GC content of the type genome is 61.39% and the genome length is 2.1 Mbp.
**Description of *Candidatus* Faecalicoccus intestinipullorum sp. nov.**
*Candidatus* Faecalicoccus intestinipullorum (in.tes.ti.ni.pul.lo’rum. L. neut. n. *intestinum* gut; L. masc. n. *pullus* a young chicken; N.L. gen. n. *intestinipullorum* of the gut of young chickens)
A bacterial species identified by metagenomic analyses. This species includes all bacteria with genomes that show ≥95% average nucleotide identity (ANI) to the type genome, which has been assigned the MAG ID ChiHjej8B7-5959 and which is available via NCBI BioSample SAMN15816766. The GC content of the type genome is 40.89% and the genome length is 1.4 Mbp.
**Description of *Candidatus* Faecaligallichristensenella gen. nov.**
*Candidatus* Faecaligallichristensenella (Fae.ca.li.gal.li.chris.ten.sen.el’la. N.L. masc. adj. *faecalis* pertaining to faeces; L. masc. n. *gallus* chicken; N.L. fem. n. *Christensenella* a genus name; N.L. fem. n. *Faecaligallichristensenella* a genus related to the genus *Christensenella* but distinct from it and found in poultry faeces)
A bacterial genus identified by metagenomic analyses. The genus includes all bacteria with genomes that show ≥60% average amino acid identity (AAI) to the type genome from the type species *Candidatus* Faecaligallichristensenella faecipullorum. This genus was identified but not named by Glendinning et al. (2020). This genus has been assigned by GTDB-Tk v1.3.0 working on GTDB Release 05-RS95 (Chaumeil et al., 2019; Parks et al., 2020) to the order *Christensenellales* and to the family *CAG-74*.
**Description of *Candidatus* Faecaligallichristensenella faecipullorum sp. nov.**
*Candidatus* Faecaligallichristensenella faecipullorum (fae.ci.pul.lo’rum. L. fem. n. *faex, faecis* excrement; L. masc. n. *pullus* a young chicken; N.L. gen. n. *faecipullorum* of young chicken faeces)
A bacterial species identified by metagenomic analyses. This species includes all bacteria with genomes that show ≥95% average nucleotide identity (ANI) to the type genome, which has been assigned the MAG ID ChiSjej6B24-5839 and which is available via NCBI BioSample SAMN15816940. The GC content of the type genome is 58.49% and the genome length is 2.6 Mbp.

**Table:** 

**Description of *Candidatus* Faecenecus gen. nov.**
*Candidatus* Faecenecus (Faec.en.e’cus. L. fem. n. *faex* dregs; Gr. masc. *enoikos* inhabitant; N.L. masc. n. *Faecenecus* a microbe associated with faeces)
A bacterial genus identified by metagenomic analyses. The genus includes all bacteria with genomes that show ≥60% average amino acid identity (AAI) to the type genome from the type species *Candidatus* Faecenecus gallistercoris. This is a name for the alphanumeric GTDB genus CAG-988. This genus has been assigned by GTDB-Tk v1.3.0 working on GTDB Release 05-RS95 (Chaumeil et al., 2019; Parks et al., 2020) to the order *RF39* and to the family *CAG-611*.
**Description of *Candidatus* Faecenecus gallistercoris sp. nov.**
*Candidatus* Faecenecus gallistercoris (gal.li.ster’co.ris. L. masc. n *gallus* chicken; L. neut. n. *stercus* dung; N.L. gen. n. *gallistercoris* of chicken faeces)
A bacterial species identified by metagenomic analyses. This species includes all bacteria with genomes that show ≥95% average nucleotide identity (ANI) to the type genome, which has been assigned the MAG ID CHK165-10780 and which is available via NCBI BioSample SAMN15817166. This is a new name for the alphanumeric GTDB species sp003149915. The GC content of the type genome is 34.49% and the genome length is 1.2 Mbp.
**Description of *Candidatus* Faecicola gen. nov.**
*Candidatus* Faecicola (Fae.ci’co.la. L. fem. n. *faex* dregs; L. suff. *-cola* inhabitant of; N.L. fem. n. *Faecicola* a microbe associated with faeces)
A bacterial genus identified by metagenomic analyses. The genus includes all bacteria with genomes that show ≥60% average amino acid identity (AAI) to the type genome from the type species *Candidatus* Faecicola pullistercoris. This is a name for the alphanumeric GTDB genus CAG-1138. This genus has been assigned by GTDB-Tk v1.3.0 working on GTDB Release 05-RS95 (Chaumeil et al., 2019; Parks et al., 2020) to the order *4C28d-15* and to the family *CAG-917*.
**Description of *Candidatus* Faecicola pullistercoris sp. nov.**
*Candidatus* Faecicola pullistercoris (pul.li.ster’co.ris. L. masc. n. *pullus* a young chicken; L. fem. n. *avis* bird; N.L. gen. n. *pullistercoris* of young chicken faeces)
A bacterial species identified by metagenomic analyses. This species includes all bacteria with genomes that show ≥95% average nucleotide identity (ANI) to the type genome, which has been assigned the MAG ID 5944 and which is available via NCBI BioSample SAMN15817151. The GC content of the type genome is 48.54% and the genome length is 1.6 Mbp.
**Description of *Candidatus* Faecimonas gen. nov.**
*Candidatus* Faecimonas (Fae.ci.mo’nas. L. fem. n. *faex* dregs; L. fem. n. *monas* a monad; N.L. fem. n. *Faecimonas* a microbe associated with faeces)
A bacterial genus identified by metagenomic analyses. The genus includes all bacteria with genomes that show ≥60% average amino acid identity (AAI) to the type genome from the type species *Candidatus* Faecimonas intestinavium. This is a name for the alphanumeric GTDB genus CAG-877. This genus has been assigned by GTDB-Tk v1.3.0 working on GTDB Release 05-RS95 (Chaumeil et al., 2019; Parks et al., 2020) to the order *RF39* and to the family *CAG-611*.
**Description of *Candidatus* Faecimonas gallistercoris sp. nov.**
*Candidatus* Faecimonas gallistercoris (gal.li.ster’co.ris. L. masc. n *gallus* chicken; L. neut. n. *stercus* dung; N.L. gen. n. *gallistercoris* of chicken faeces)
A bacterial species identified by metagenomic analyses. This species includes all bacteria with genomes that show ≥95% average nucleotide identity (ANI) to the type genome, which has been assigned the MAG ID CHK189-3136 and which is available via NCBI BioSample SAMN15817016. The GC content of the type genome is 28.18% and the genome length is 1.4 Mbp.
**Description of *Candidatus* Faecimonas intestinavium sp. nov.**
*Candidatus* Faecimonas intestinavium (in.tes.tin.a’vi.um. L. neut. n. *intestinum* gut; L. fem. n. *avis* bird; N.L. gen. n. *intestinavium* of the gut of birds)
A bacterial species identified by metagenomic analyses. This species includes all bacteria with genomes that show ≥95% average nucleotide identity (ANI) to the type genome, which has been assigned the MAG ID USAMLcec2-12447 and which is available via NCBI BioSample SAMN15817225. This is a new name for the alphanumeric GTDB species sp900554305. The GC content of the type genome is 29.22% and the genome length is 1.8 Mbp.
**Description of *Candidatus* Faecimorpha gen. nov.**
*Candidatus* Faecimorpha (Fae.ci.mor’pha. L. fem. n. *faex* dregs; Gr. fem. n. *morphe* a form, shape; N.L. fem. n. *Faecimorpha* a microbe associated with faeces)
A bacterial genus identified by metagenomic analyses. The genus includes all bacteria with genomes that show ≥60% average amino acid identity (AAI) to the type genome from the type species *Candidatus* Faecimorpha stercoravium. This is a name for the alphanumeric GTDB genus UBA1390. This genus has been assigned by GTDB-Tk v1.3.0 working on GTDB Release 05-RS95 (Chaumeil et al., 2019; Parks et al., 2020) to the order *Lachnospirales* and to the family *UBA1390*.

**Table:** 

**Description of *Candidatus* Faecimorpha stercoravium sp. nov.**
*Candidatus* Faecimorpha stercoravium (ster.cor.a’vi.um. L. neut. n. *stercus* dung; L. fem. n. *avis* bird; N.L. gen. n. *stercoravium* of bird faeces)
A bacterial species identified by metagenomic analyses. This species includes all bacteria with genomes that show ≥95% average nucleotide identity (ANI) to the type genome, which has been assigned the MAG ID CHK195-9767 and which is available via NCBI BioSample SAMN15817172. This is a new name for the alphanumeric GTDB species sp002305315. The GC content of the type genome is 49.81% and the genome length is 2.4 Mbp.
**Description of *Candidatus* Faeciplasma gen. nov.**
*Candidatus* Faeciplasma (Fae.ci.plas’ma. L. fem. n. *faex* dregs; Gr. neut. n. *plasma* a form; N.L. neut. n. *Faeciplasma* a microbe associated with faeces)
A bacterial genus identified by metagenomic analyses. The genus includes all bacteria with genomes that show ≥60% average amino acid identity (AAI) to the type genome from the type species *Candidatus* Faeciplasma avium. This is a name for the alphanumeric GTDB genus UBA1409. This genus has been assigned by GTDB-Tk v1.3.0 working on GTDB Release 05-RS95 (Chaumeil et al., 2019; Parks et al., 2020) to the order *Oscillospirales* and to the family *Ruminococcaceae*.
**Description of *Candidatus* Faeciplasma avium sp. nov.**
*Candidatus* Faeciplasma avium (a’vi.um. L. fem. pl. n. *avium* of birds)
A bacterial species identified by metagenomic analyses. This species includes all bacteria with genomes that show ≥95% average nucleotide identity (ANI) to the type genome, which has been assigned the MAG ID 1370 and which is available via NCBI BioSample SAMN15817208. This is a new name for the alphanumeric GTDB species sp002305045. The GC content of the type genome is 51.56% and the genome length is 1.6 Mbp.
**Description of *Candidatus* Faeciplasma gallinarum sp. nov.**
*Candidatus* Faeciplasma gallinarum (gal.li.na’rum. L. fem. n. *gallina* a hen; L. gen. fem. pl. n. *gallinarum* of hens)
A bacterial species identified by metagenomic analyses. This species includes all bacteria with genomes that show ≥95% average nucleotide identity (ANI) to the type genome, which has been assigned the MAG ID CHK157-1446 and which is available via NCBI BioSample SAMN15817182. This is a new name for the alphanumeric GTDB species sp002338885. The GC content of the type genome is 49.59% and the genome length is 1.6 Mbp.
**Description of *Candidatus* Faeciplasma pullistercoris sp. nov.**
*Candidatus* Faeciplasma pullistercoris (pul.li.ster’co.ris. L. masc. n. *pullus* a young chicken; L. neut. n. *stercus* dung; N.L. gen. n. *pullistercoris* of young chicken faeces)
A bacterial species identified by metagenomic analyses. This species includes all bacteria with genomes that show ≥95% average nucleotide identity (ANI) to the type genome, which has been assigned the MAG ID CHK33-4379 and which is available via NCBI BioSample SAMN15817120. The GC content of the type genome is 49.45% and the genome length is 1.5 Mbp.
**Description of *Candidatus* Faecisoma gen. nov.**
*Candidatus* Faecisoma (Fae.ci.so’ma. L. fem. n. *faex* dregs; Gr. neut. n. *soma* a body; N.L. neut. n. *Faecisoma* a microbe associated with faeces)
A bacterial genus identified by metagenomic analyses. The genus includes all bacteria with genomes that show ≥60% average amino acid identity (AAI) to the type genome from the type species *Candidatus* Faecisoma merdavium. This is a name for the alphanumeric GTDB genus CAG-878. This genus has been assigned by GTDB-Tk v1.3.0 working on GTDB Release 05-RS95 (Chaumeil et al., 2019; Parks et al., 2020) to the order *RF39* and to the family *CAG-822*.
**Description of *Candidatus* Faecisoma merdavium sp. nov.**
*Candidatus* Faecisoma merdavium (merd.a’vi.um. L. fem. n. *merda* faeces; L. fem. n. *avis* bird; N.L. gen. n. *merdavium* of bird faeces)
A bacterial species identified by metagenomic analyses. This species includes all bacteria with genomes that show ≥95% average nucleotide identity (ANI) to the type genome, which has been assigned the MAG ID 6595 and which is available via NCBI BioSample SAMN15817101. The GC content of the type genome is 24.63% and the genome length is 1.3 Mbp.
**Description of *Candidatus* Faecivicinus gen. nov.**
*Candidatus* Faecivicinus (Fae.ci.vi.ci’nus. L. fem. n. *faex* dregs; L. masc. n. *vicinus* a neighbour; N.L. masc. n. *Faecivicinus* a microbe associated with faeces)
A bacterial genus identified by metagenomic analyses. The genus includes all bacteria with genomes that show ≥60% average amino acid identity (AAI) to the type genome from the type species *Candidatus* Faecivicinus avistercoris. This is a name for the alphanumeric GTDB genus UMGS1603. This genus has been assigned by GTDB-Tk v1.3.0 working on GTDB Release 05-RS95 (Chaumeil et al., 2019; Parks et al., 2020) to the order *Christensenellales* and to the family *CAG-74*.

**Table:** 

**Description of *Candidatus* Faecivicinus avistercoris sp. nov.**
*Candidatus* Faecivicinus avistercoris (a.vi.ster’co.ris. L. fem. n. *avis* bird; L. neut. n. *stercus* dung; N.L. gen. n. *avistercoris* of bird faeces)
A bacterial species identified by metagenomic analyses. This species includes all bacteria with genomes that show ≥95% average nucleotide identity (ANI) to the type genome, which has been assigned the MAG ID 905 and which is available via NCBI BioSample SAMN15817031. The GC content of the type genome is 63.46% and the genome length is 2.7 Mbp.
**Description of *Candidatus* Faecivivens gen. nov.**
*Candidatus* Faecivivens (Fae.ci.vi’vens. L. fem. n. *faex* dregs; N.L. pres. part. *vivens* living; N.L. fem. n. *Faecivivens* a microbe associated with faeces)
A bacterial genus identified by metagenomic analyses. The genus includes all bacteria with genomes that show ≥60% average amino acid identity (AAI) to the type genome from the type species *Candidatus* Faecivivens stercorigallinarum. This is a name for the alphanumeric GTDB genus UBA1448. This genus has been assigned by GTDB-Tk v1.3.0 working on GTDB Release 05-RS95 (Chaumeil et al., 2019; Parks et al., 2020) to the order *Oscillospirales* and to the family *Ruminococcaceae*.
**Description of *Candidatus* Faecivivens stercoravium sp. nov.**
*Candidatus* Faecivivens stercoravium (ster.cor.a’vi.um. L. neut. n. *stercus* dung; L. fem. n. *avis* bird; N.L. gen. n. *stercoravium* of bird faeces)
A bacterial species identified by metagenomic analyses. This species includes all bacteria with genomes that show ≥95% average nucleotide identity (ANI) to the type genome, which has been assigned the MAG ID CHK189-12415 and which is available via NCBI BioSample SAMN15817018. The GC content of the type genome is 59.75% and the genome length is 2.3 Mbp.
**Description of *Candidatus* Faecivivens stercorigallinarum sp. nov.**
*Candidatus* Faecivivens stercorigallinarum (ster.co.ri.gal.li.na’rum. L. neut. n. *stercus* dung; L. fem. n. *gallina* hen; N.L. gen. n. *stercorigallinarum* of hen faeces)
A bacterial species identified by metagenomic analyses. This species includes all bacteria with genomes that show ≥95% average nucleotide identity (ANI) to the type genome, which has been assigned the MAG ID 4960 and which is available via NCBI BioSample SAMN15817121. The GC content of the type genome is 52.99% and the genome length is 2.2 Mbp.
**Description of *Candidatus* Faecivivens stercoripullorum sp. nov.**
*Candidatus* Faecivivens stercoripullorum (ster.co.ri.pul.lo’rum. L. neut. n. *stercus* dung; L. masc. n. *pullus* a young chicken; N.L. gen. n. *stercoripullorum* of the faceces of young chickens)
A bacterial species identified by metagenomic analyses. This species includes all bacteria with genomes that show ≥95% average nucleotide identity (ANI) to the type genome, which has been assigned the MAG ID ChiBcec7-5410 and which is available via NCBI BioSample SAMN15817124. The GC content of the type genome is 50.38% and the genome length is 2.0 Mbp.
**Description of *Candidatus* Faecousia gen. nov.**
*Candidatus* Faecousia (Faec.ou’si.a. L. fem. n. *faex* dregs; Gr. fem. n. *ousia* an essence; N.L. fem. n. *Faecousia* a microbe associated with faeces)
A bacterial genus identified by metagenomic analyses. The genus includes all bacteria with genomes that show ≥60% average amino acid identity (AAI) to the type genome from the type species *Candidatus* Faecousia intestinigallinarum. This is a name for the alphanumeric GTDB genus CAG-110. This genus has been assigned by GTDB-Tk v1.3.0 working on GTDB Release 05-RS95 (Chaumeil et al., 2019; Parks et al., 2020) to the order *Oscillospirales* and to the family *Oscillospiraceae*.
**Description of *Candidatus* Faecousia excrementigallinarum sp. nov.**
*Candidatus* Faecousia excrementigallinarum (ex.cre.men.ti.gal.li.na’rum. L. neut. n. *excrementum* excrement; L. fem. n. *gallina* hen; N.L. gen. n. *excrementigallinarum* of hen excrement)
A bacterial species identified by metagenomic analyses. This species includes all bacteria with genomes that show ≥95% average nucleotide identity (ANI) to the type genome, which has been assigned the MAG ID 13361 and which is available via NCBI BioSample SAMN15817055. The GC content of the type genome is 56.40% and the genome length is 1.9 Mbp.
**Description of *Candidatus* Faecousia excrementipullorum sp. nov.**
*Candidatus* Faecousia excrementipullorum (ex.cre.men.ti.pul.lo’rum. L. neut. n. *excrementum* excrement; L. masc. n. *pullus* a young chicken; N.L. gen. n. *excrementipullorum* of young chicken excrement)
A bacterial species identified by metagenomic analyses. This species includes all bacteria with genomes that show ≥95% average nucleotide identity (ANI) to the type genome, which has been assigned the MAG ID ChiSxjej6B18-3616 and which is available via NCBI BioSample SAMN15817060. The GC content of the type genome is 56.19% and the genome length is 1.7 Mbp.

**Table:** 

**Description of *Candidatus* Faecousia faecavium sp. nov.**
*Candidatus* Faecousia faecavium (faec.a’vi.um. L. fem. n. *faex, faecis* excrement; L. fem. n. *avis* bird; N.L. gen. n. *faecavium* of bird faeces)
A bacterial species identified by metagenomic analyses. This species includes all bacteria with genomes that show ≥95% average nucleotide identity (ANI) to the type genome, which has been assigned the MAG ID ChiBcec21-2751 and which is available via NCBI BioSample SAMN15817064. The GC content of the type genome is 53.76% and the genome length is 2.4 Mbp.
**Description of *Candidatus* Faecousia faecigallinarum sp. nov.**
*Candidatus* Faecousia faecigallinarum (fae.ci.gal.li.na’rum. L. fem. n. *faex, faecis* excrement; L. fem. n. *gallina* hen; N.L. gen. n. *faecigallinarum* of hen faeces)
A bacterial species identified by metagenomic analyses. This species includes all bacteria with genomes that show ≥95% average nucleotide identity (ANI) to the type genome, which has been assigned the MAG ID ChiHcolR29-948 and which is available via NCBI BioSample SAMN15817073. The GC content of the type genome is 58.86% and the genome length is 1.9 Mbp.
**Description of *Candidatus* Faecousia faecipullorum sp. nov.**
*Candidatus* Faecousia faecipullorum (fae.ci.pul.lo’rum. L. fem. n. *faex, faecis* excrement; L. masc. n. *pullus* a young chicken; N.L. gen. n. *faecipullorum* of young chicken faeces)
A bacterial species identified by metagenomic analyses. This species includes all bacteria with genomes that show ≥95% average nucleotide identity (ANI) to the type genome, which has been assigned the MAG ID ChiHecec2B26-1122 and which is available via NCBI BioSample SAMN15817098. The GC content of the type genome is 55.50% and the genome length is 1.9 Mbp.
**Description of *Candidatus* Faecousia gallistercoris sp. nov.**
*Candidatus* Faecousia gallistercoris (gal.li.ster’co.ris. L. masc. n *gallus* chicken; L. neut. n. *stercus* dung; N.L. gen. n. *gallistercoris* of chicken faeces)
A bacterial species identified by metagenomic analyses. This species includes all bacteria with genomes that show ≥95% average nucleotide identity (ANI) to the type genome, which has been assigned the MAG ID 7739 and which is available via NCBI BioSample SAMN15817186. This is a new name for the alphanumeric GTDB species sp900546915. The GC content of the type genome is 58.30% and the genome length is 1.8 Mbp.
**Description of *Candidatus* Faecousia intestinavium sp. nov.**
*Candidatus* Faecousia intestinavium (in.tes.tin.a’vi.um. L. neut. n. *intestinum* gut; L. fem. n. *avis* bird; N.L. gen. n. *intestinavium* of the gut of birds)
A bacterial species identified by metagenomic analyses. This species includes all bacteria with genomes that show ≥95% average nucleotide identity (ANI) to the type genome, which has been assigned the MAG ID ChiHcec3-9842 and which is available via NCBI BioSample SAMN15817111. The GC content of the type genome is 57.16% and the genome length is 2.1 Mbp.
**Description of *Candidatus* Faecousia intestinigallinarum sp. nov.**
*Candidatus* Faecousia intestinigallinarum (in.tes.ti.ni.gal.li.na’rum. L. neut. n. *intestinum* gut; L. fem. n. *gallina* hen; N.L. gen. n. *intestinigallinarum* of the gut of the hens)
A bacterial species identified by metagenomic analyses. This species includes all bacteria with genomes that show ≥95% average nucleotide identity (ANI) to the type genome, which has been assigned the MAG ID ChiSxjej3B15-29383 and which is available via NCBI BioSample SAMN15817112. The GC content of the type genome is 55.74% and the genome length is 2.1 Mbp.
**Description of *Candidatus* Fimadaptatus gen. nov.**
*Candidatus* Fimadaptatus (Fim.a.dap.ta’tus. L. neut. n. *fimum* dung; L. past part. masc. *adaptatus* adapted to; N.L. masc. n. *Fimadaptatus* a microbe associated with faeces)
A bacterial genus identified by metagenomic analyses. The genus includes all bacteria with genomes that show ≥60% average amino acid identity (AAI) to the type genome from the type species *Candidatus* Fimadaptatus faecigallinarum. This is a name for the alphanumeric GTDB genus UMGS1633. This genus has been assigned by GTDB-Tk v1.3.0 working on GTDB Release 05-RS95 (Chaumeil et al., 2019; Parks et al., 2020) to the order *Christensenellales* and to the family *CAG-74*.
**Description of *Candidatus* Fimadaptatus faecigallinarum sp. nov.**
*Candidatus* Fimadaptatus faecigallinarum (fae.ci.gal.li.na’rum. L. fem. n. *faex, faecis* excrement; L. fem. n. *gallina* hen; N.L. gen. n. *faecigallinarum* of hen faeces)
A bacterial species identified by metagenomic analyses. This species includes all bacteria with genomes that show ≥95% average nucleotide identity (ANI) to the type genome, which has been assigned the MAG ID ChiSxjej2B14-8506 and which is available via NCBI BioSample SAMN15817140. The GC content of the type genome is 60.44% and the genome length is 2.8 Mbp.

**Table:** 

**Description of *Candidatus* Fimenecus gen. nov.**
*Candidatus* Fimenecus (Fim.en.e’cus. L. neut. n. *fimum* dung; Gr. masc. *enoikos* inhabitant; N.L. masc. n. *Fimenecus* a microbe associated with faeces)
A bacterial genus identified by metagenomic analyses. The genus includes all bacteria with genomes that show ≥60% average amino acid identity (AAI) to the type genome from the type species *Candidatus* Fimenecus excrementigallinarum. This is a name for the alphanumeric GTDB genus CAG-180. This genus has been assigned by GTDB-Tk v1.3.0 working on GTDB Release 05-RS95 (Chaumeil et al., 2019; Parks et al., 2020) to the order *Oscillospirales* and to the family *Acutalibacteraceae*.
**Description of *Candidatus* Fimenecus excrementavium sp. nov.**
*Candidatus* Fimenecus excrementavium (ex.cre.ment.a’vi.um. L. neut. n. *excrementum* excrement; L. fem. n. *avis* bird; N.L. gen. n. *excrementavium* of bird excrement)
A bacterial species identified by metagenomic analyses. This species includes all bacteria with genomes that show ≥95% average nucleotide identity (ANI) to the type genome, which has been assigned the MAG ID ChiSjej1B19-6168 and which is available via NCBI BioSample SAMN15817011. The GC content of the type genome is 50.61% and the genome length is 1.8 Mbp.
**Description of *Candidatus* Fimenecus excrementigallinarum sp. nov.**
*Candidatus* Fimenecus excrementigallinarum (ex.cre.men.ti.gal.li.na’rum. L. neut. n. *excrementum* excrement; L. fem. n. *gallina* hen; N.L. gen. n. *excrementigallinarum* of hen excrement)
A bacterial species identified by metagenomic analyses. This species includes all bacteria with genomes that show ≥95% average nucleotide identity (ANI) to the type genome, which has been assigned the MAG ID ChiGjej1B1-19959 and which is available via NCBI BioSample SAMN15817134. The GC content of the type genome is 60.29% and the genome length is 1.8 Mbp.
**Description of *Candidatus* Fimenecus stercoravium sp. nov.**
*Candidatus* Fimenecus stercoravium (ster.cor.a’vi.um. L. neut. n. *stercus* dung; L. fem. n. *avis* bird; N.L. gen. n. *stercoravium* of bird faeces)
A bacterial species identified by metagenomic analyses. This species includes all bacteria with genomes that show ≥95% average nucleotide identity (ANI) to the type genome, which has been assigned the MAG ID ChiHcolR13-3023 and which is available via NCBI BioSample SAMN15817181. This is a new name for the alphanumeric GTDB species sp002314305. The GC content of the type genome is 55.43% and the genome length is 1.9 Mbp.
**Description of *Candidatus* Fimicola gen. nov.**
*Candidatus* Fimicola (Fi.mi’co.la. L. neut. n. *fimum* dung; L. suff. *-cola* inhabitant of; N.L. fem. n. *Fimicola* a microbe associated with faeces)
A bacterial genus identified by metagenomic analyses. The genus includes all bacteria with genomes that show ≥60% average amino acid identity (AAI) to the type genome from the type species *Candidatus* Fimicola merdigallinarum. This is a name for the alphanumeric GTDB genus An114. This genus has been assigned by GTDB-Tk v1.3.0 working on GTDB Release 05-RS95 (Chaumeil et al., 2019; Parks et al., 2020) to the order *Lachnospirales* and to the family *Anaerotignaceae*.
**Description of *Candidatus* Fimicola cottocaccae sp. nov.**
*Candidatus* Fimicola cottocaccae (cot.to.cac’cae. Gr. masc. n. *kottos* chicken Gr. fem. n. *kakke* faeces; N.L. gen. n. *cottocaccae* of chicken faeces)
A bacterial species identified by metagenomic analyses. This species includes all bacteria with genomes that show ≥95% average nucleotide identity (ANI) to the type genome, which has been assigned the MAG ID ChiW9-1577 and which is available via NCBI BioSample SAMN15817191. This is a new name for the alphanumeric GTDB species sp002161055. The GC content of the type genome is 31.91% and the genome length is 1.8 Mbp.
**Description of *Candidatus* Fimicola merdigallinarum sp. nov.**
*Candidatus* Fimicola merdigallinarum (mer.di.gal.li.na’rum. L. fem. n. *merda* faeces; L. fem. n. *gallina* hen; N.L. gen. n. *merdigallinarum* of hen faeces)
A bacterial species identified by metagenomic analyses. This species includes all bacteria with genomes that show ≥95% average nucleotide identity (ANI) to the type genome, which has been assigned the MAG ID F6-4510 and which is available via NCBI BioSample SAMN15817136. The GC content of the type genome is 32.46% and the genome length is 1.8 Mbp.
**Description of *Candidatus* Fimihabitans gen. nov.**
*Candidatus* Fimihabitans (Fi.mi.ha’bi.tans. L. neut. n. *fimum* dung; L. pres. part. *habitans* an inhabitant; N.L. fem. n. *Fimihabitans* a microbe associated with faeces)
A bacterial genus identified by metagenomic analyses. The genus includes all bacteria with genomes that show ≥60% average amino acid identity (AAI) to the type genome from the type species *Candidatus* Fimihabitans intestinipullorum. This is a name for the alphanumeric GTDB genus UMGS1648. This genus has been assigned by GTDB-Tk v1.3.0 working on GTDB Release 05-RS95 (Chaumeil et al., 2019; Parks et al., 2020) to the order *RF39* and to the family *CAG-822*.

**Table:** 

**Description of *Candidatus* Fimihabitans intestinipullorum sp. nov.**
*Candidatus* Fimihabitans intestinipullorum (in.tes.ti.ni.pul.lo’rum. L. neut. n. *intestinum* gut; L. masc. n. *pullus* a young chicken; N.L. gen. n. *intestinipullorum* of the gut of young chickens)
A bacterial species identified by metagenomic analyses. This species includes all bacteria with genomes that show ≥95% average nucleotide identity (ANI) to the type genome, which has been assigned the MAG ID CHK197-8231 and which is available via NCBI BioSample SAMN15817229. This is a new name for the alphanumeric GTDB species sp900553765. The GC content of the type genome is 33.44% and the genome length is 1.3 Mbp.
**Description of *Candidatus* Fimimonas gen. nov.**
*Candidatus* Fimimonas (Fi.mi.mo’nas. L. neut. n. *fimum* dung; L. fem. n. *monas* a monad; N.L. fem. n. *Fimimonas* a microbe associated with faeces)
A bacterial genus identified by metagenomic analyses. The genus includes all bacteria with genomes that show ≥60% average amino acid identity (AAI) to the type genome from the type species *Candidatus* Fimimonas gallinarum. This is a name for the alphanumeric GTDB genus CAG-1435. This genus has been assigned by GTDB-Tk v1.3.0 working on GTDB Release 05-RS95 (Chaumeil et al., 2019; Parks et al., 2020) to the order *Christensenellales* and to the family *CAG-314*.
**Description of *Candidatus* Fimimonas gallinarum sp. nov.**
*Candidatus* Fimimonas gallinarum (gal.li.na’rum. L. fem. n. *gallina* a hen; L. gen. fem. pl. n. *gallinarum* of hens)
A bacterial species identified by metagenomic analyses. This species includes all bacteria with genomes that show ≥95% average nucleotide identity (ANI) to the type genome, which has been assigned the MAG ID CHK121-14286 and which is available via NCBI BioSample SAMN15817176. This is a new name for the alphanumeric GTDB species sp000433775. The GC content of the type genome is 45.96% and the genome length is 1.4 Mbp.
**Description of *Candidatus* Fimimonas merdipullorum sp. nov.**
*Candidatus* Fimimonas merdipullorum (mer.di.pul.lo’rum. L. fem. n. *merda* faeces; L. masc. n. *pullus* a young chicken; N.L. gen. n. *merdipullorum* of the faeces of young chickens)
A bacterial species identified by metagenomic analyses. This species includes all bacteria with genomes that show ≥95% average nucleotide identity (ANI) to the type genome, which has been assigned the MAG ID ChiHjej12B11-7776 and which is available via NCBI BioSample SAMN15817153. The GC content of the type genome is 53.17% and the genome length is 1.3 Mbp.
**Description of *Candidatus* Fimimorpha gen. nov.**
*Candidatus* Fimimorpha (Fi.mi.mor’pha. L. neut. n. *fimum* dung; Gr. fem. n. *morphe* a form, shape; N.L. fem. n. *Fimimorpha* a microbe associated with faeces)
A bacterial genus identified by metagenomic analyses. The genus includes all bacteria with genomes that show ≥60% average amino acid identity (AAI) to the type genome from the type species *Candidatus* Fimimorpha faecalis. This is a name for the alphanumeric GTDB genus CHKCI001. This genus has been assigned by GTDB-Tk v1.3.0 working on GTDB Release 05-RS95 (Chaumeil et al., 2019; Parks et al., 2020) to the order *Lachnospirales* and to the family *Lachnospiraceae*.
**Description of *Candidatus* Fimimorpha excrementavium sp. nov.**
*Candidatus* Fimimorpha excrementavium (ex.cre.ment.a’vi.um. L. neut. n. *excrementum* excrement; L. fem. n. *avis* bird; N.L. gen. n. *excrementavium* of bird excrement)
A bacterial species identified by metagenomic analyses. This species includes all bacteria with genomes that show ≥95% average nucleotide identity (ANI) to the type genome, which has been assigned the MAG ID CHK193-21555 and which is available via NCBI BioSample SAMN15817029. The GC content of the type genome is 48.70% and the genome length is 3.1 Mbp.
**Description of *Candidatus* Fimimorpha faecalis sp. nov.**
*Candidatus* Fimimorpha faecalis (fae.ca’lis. L. fem. adj. *faecalis* of faeces)
A bacterial species identified by metagenomic analyses. This species includes all bacteria with genomes that show ≥95% average nucleotide identity (ANI) to the type genome, which has been assigned the MAG ID ChiW13-3771 and which is available via NCBI BioSample SAMN15817177. This is a new name for the alphanumeric GTDB species sp900045905. The GC content of the type genome is 36.24% and the genome length is 2.9 Mbp.
**Description of *Candidatus* Fimiplasma gen. nov.**
*Candidatus* Fimiplasma (Fi.mi.plas’ma. L. neut. n. *fimum* dung; Gr. neut. n. *plasma* a form; N.L. neut. n. *Fimiplasma* a microbe associated with faeces)
A bacterial genus identified by metagenomic analyses. The genus includes all bacteria with genomes that show ≥60% average amino acid identity (AAI) to the type genome from the type species *Candidatus* Fimiplasma intestinipullorum. This is a name for the alphanumeric GTDB genus CHKCI006. This genus has been assigned by GTDB-Tk v1.3.0 working on GTDB Release 05-RS95 (Chaumeil et al., 2019; Parks et al., 2020) to the order *Erysipelotrichales* and to the family *Erysipelatoclostridiaceae*.

**Table:** 

**Description of *Candidatus* Fimiplasma intestinipullorum sp. nov.**
*Candidatus* Fimiplasma intestinipullorum (in.tes.ti.ni.pul.lo’rum. L. neut. n. *intestinum* gut; L. masc. n. *pullus* a young chicken; N.L. gen. n. *intestinipullorum* of the gut of young chickens)
A bacterial species identified by metagenomic analyses. This species includes all bacteria with genomes that show ≥95% average nucleotide identity (ANI) to the type genome, which has been assigned the MAG ID CHK195-11698 and which is available via NCBI BioSample SAMN15817196. This is a new name for the alphanumeric GTDB species sp900018345. The GC content of the type genome is 43.31% and the genome length is 2.5 Mbp.
**Description of *Candidatus* Fimisoma gen. nov.**
*Candidatus* Fimisoma (Fi.mi.so’ma. L. neut. n. *fimum* dung; Gr. neut. n. *soma* a body; N.L. neut. n. *Fimisoma* a microbe associated with faeces)
A bacterial genus identified by metagenomic analyses. The genus includes all bacteria with genomes that show ≥60% average amino acid identity (AAI) to the type genome from the type species *Candidatus* Fimisoma avicola. This is a name for the alphanumeric GTDB genus CAG-145. This genus has been assigned by GTDB-Tk v1.3.0 working on GTDB Release 05-RS95 (Chaumeil et al., 2019; Parks et al., 2020) to the order *Peptostreptococcales* and to the family *Anaerovoracaceae*.
**Description of *Candidatus* Fimisoma avicola sp. nov.**
*Candidatus* Fimisoma avicola (a.vi’co.la. L. fem. n. *avis* bird; L. suff. *-cola* inhabitant of; N.L. n. *avicola* inhabitant of birds)
A bacterial species identified by metagenomic analyses. This species includes all bacteria with genomes that show ≥95% average nucleotide identity (ANI) to the type genome, which has been assigned the MAG ID 11300 and which is available via NCBI BioSample SAMN15817197. This is a new name for the alphanumeric GTDB species sp900542565. The GC content of the type genome is 47.90% and the genome length is 2.0 Mbp.
**Description of *Candidatus* Fimivicinus gen. nov.**
*Candidatus* Fimivicinus (Fi.mi.vi.ci’nus. L. neut. n. *fimum* dung; L. masc. n. *vicinus* a neighbour; N.L. masc. n. *Fimivicinus* a microbe associated with faeces)
A bacterial genus identified by metagenomic analyses. The genus includes all bacteria with genomes that show ≥60% average amino acid identity (AAI) to the type genome from the type species *Candidatus* Fimivicinus intestinavium. This is a name for the alphanumeric GTDB genus UBA1691. This genus has been assigned by GTDB-Tk v1.3.0 working on GTDB Release 05-RS95 (Chaumeil et al., 2019; Parks et al., 2020) to the order *Oscillospirales* and to the family *Acutalibacteraceae*.
**Description of *Candidatus* Fimivicinus intestinavium sp. nov.**
*Candidatus* Fimivicinus intestinavium (in.tes.tin.a’vi.um. L. neut. n. *intestinum* gut; L. fem. n. *avis* bird; N.L. gen. n. *intestinavium* of the gut of birds)
A bacterial species identified by metagenomic analyses. This species includes all bacteria with genomes that show ≥95% average nucleotide identity (ANI) to the type genome, which has been assigned the MAG ID 2526 and which is available via NCBI BioSample SAMN15817188. This is a new name for the alphanumeric GTDB species sp900552985. The GC content of the type genome is 55.20% and the genome length is 2.5 Mbp.
**Description of *Candidatus* Fimivivens gen. nov.**
*Candidatus* Fimivivens (Fi.mi.vi’vens. L. neut. n. *fimum* dung; N.L. pres. part. *vivens* living; N.L. fem. n. *Fimivivens* a microbe associated with faeces)
A bacterial genus identified by metagenomic analyses. The genus includes all bacteria with genomes that show ≥60% average amino acid identity (AAI) to the type genome from the type species *Candidatus* Fimivivens faecavium. This is a name for the alphanumeric GTDB genus D5. This genus has been assigned by GTDB-Tk v1.3.0 working on GTDB Release 05-RS95 (Chaumeil et al., 2019; Parks et al., 2020) to the order *Oscillospirales* and to the family *Ruminococcaceae*.
**Description of *Candidatus* Fimivivens faecavium sp. nov.**
*Candidatus* Fimivivens faecavium (faec.a’vi.um. L. fem. n. *faex, faecis* excrement; L. fem. n. *avis* bird; N.L. gen. n. *faecavium* of bird faeces)
A bacterial species identified by metagenomic analyses. This species includes all bacteria with genomes that show ≥95% average nucleotide identity (ANI) to the type genome, which has been assigned the MAG ID CHK195-35099 and which is available via NCBI BioSample SAMN15817038. The GC content of the type genome is 58.86% and the genome length is 2.0 Mbp.
**Description of *Candidatus* Fimousia gen. nov.**
*Candidatus* Fimousia (Fim.ou’si.a. L. neut. n. *fimum* dung; Gr. fem. n. *ousia* an essence; N.L. fem. n. *Fimousia* a microbe associated with faeces)
A bacterial genus identified by metagenomic analyses. The genus includes all bacteria with genomes that show ≥60% average amino acid identity (AAI) to the type genome from the type species *Candidatus* Fimousia stercorigallinarum. This is a name for the alphanumeric GTDB genus 992a. This genus has been assigned by GTDB-Tk v1.3.0 working on GTDB Release 05-RS95 (Chaumeil et al., 2019; Parks et al., 2020) to the order *Lachnospirales* and to the family *Lachnospiraceae*.

**Table:** 

**Description of *Candidatus* Fimousia stercorigallinarum sp. nov.**
*Candidatus* Fimousia stercorigallinarum (ster.co.ri.gal.li.na’rum. L. neut. n. *stercus* dung; L. fem. n. *gallina* hen; N.L. gen. n. *stercorigallinarum* of hen faeces)
A bacterial species identified by metagenomic analyses. This species includes all bacteria with genomes that show ≥95% average nucleotide identity (ANI) to the type genome, which has been assigned the MAG ID ChiSxjej3B15-1827 and which is available via NCBI BioSample SAMN15817114. The GC content of the type genome is 41.52% and the genome length is 2.3 Mbp.
**Description of *Candidatus* Flavonifractor avicola sp. nov.**
*Candidatus* Flavonifractor avicola (a.vi’co.la. L. fem. n. *avis* bird; L. suff. *-cola* inhabitant of; N.L. n. *avicola* inhabitant of birds)
A bacterial species identified by metagenomic analyses. This species includes all bacteria with genomes that show ≥95% average nucleotide identity (ANI) to the type genome, which has been assigned the MAG ID CHK178-4001 and which is available via NCBI BioSample SAMN15816843. This is a new name for the alphanumeric GTDB species sp002161085. The GC content of the type genome is 60.44% and the genome length is 2.5 Mbp.
**Description of *Candidatus* Flavonifractor avistercoris sp. nov.**
*Candidatus* Flavonifractor avistercoris (a.vi.ster’co.ris. L. fem. n. *avis* bird; L. neut. n. *stercus* dung; N.L. gen. n. *avistercoris* of bird faeces)
A bacterial species identified by metagenomic analyses. This species includes all bacteria with genomes that show ≥95% average nucleotide identity (ANI) to the type genome, which has been assigned the MAG ID 6084 and which is available via NCBI BioSample SAMN15816821. This is a new name for the alphanumeric GTDB species sp002161215. The GC content of the type genome is 65.16% and the genome length is 2.4 Mbp.
**Description of *Candidatus* Flavonifractor intestinigallinarum sp. nov.**
*Candidatus* Flavonifractor intestinigallinarum (in.tes.ti.ni.gal.li.na’rum. L. neut. n. *intestinum* gut; L. fem. n. *gallina* hen; N.L. gen. n. *intestinigallinarum* of the gut of the hens)
A bacterial species identified by metagenomic analyses. This species includes all bacteria with genomes that show ≥95% average nucleotide identity (ANI) to the type genome, which has been assigned the MAG ID CHK192-8294 and which is available via NCBI BioSample SAMN15816592. The GC content of the type genome is 61.61% and the genome length is 2.5 Mbp.
**Description of *Candidatus* Flavonifractor intestinipullorum sp. nov.**
*Candidatus* Flavonifractor intestinipullorum (in.tes.ti.ni.pul.lo’rum. L. neut. n. *intestinum* gut; L. masc. n. *pullus* a young chicken; N.L. gen. n. *intestinipullorum* of the gut of young chickens)
A bacterial species identified by metagenomic analyses. This species includes all bacteria with genomes that show ≥95% average nucleotide identity (ANI) to the type genome, which has been assigned the MAG ID CHK189-11263 and which is available via NCBI BioSample SAMN15816594. The GC content of the type genome is 63.72% and the genome length is 2.2 Mbp.
**Description of *Candidatus* Flavonifractor merdavium sp. nov.**
*Candidatus* Flavonifractor merdavium (merd.a’vi.um. L. fem. n. *merda* faeces; L. fem. n. *avis* bird; N.L. gen. n. *merdavium* of bird faeces)
A bacterial species identified by metagenomic analyses. This species includes all bacteria with genomes that show ≥95% average nucleotide identity (ANI) to the type genome, which has been assigned the MAG ID 3313 and which is available via NCBI BioSample SAMN15816644. The GC content of the type genome is 62.46% and the genome length is 2.7 Mbp.
**Description of *Candidatus* Flavonifractor merdigallinarum sp. nov.**
*Candidatus* Flavonifractor merdigallinarum (mer.di.gal.li.na’rum. L. fem. n. *merda* faeces; L. fem. n. *gallina* hen; N.L. gen. n. *merdigallinarum* of hen faeces)
A bacterial species identified by metagenomic analyses. This species includes all bacteria with genomes that show ≥95% average nucleotide identity (ANI) to the type genome, which has been assigned the MAG ID ChiBcec16-6824 and which is available via NCBI BioSample SAMN15816721. The GC content of the type genome is 61.09% and the genome length is 2.6 Mbp.
**Description of *Candidatus* Flavonifractor merdipullorum sp. nov.**
*Candidatus* Flavonifractor merdipullorum (mer.di.pul.lo’rum. L. fem. n. *merda* faeces; L. masc. n. *pullus* a young chicken; N.L. gen. n. *merdipullorum* of the faeces of young chickens)
A bacterial species identified by metagenomic analyses. This species includes all bacteria with genomes that show ≥95% average nucleotide identity (ANI) to the type genome, which has been assigned the MAG ID ChiGjej6B6-1540 and which is available via NCBI BioSample SAMN15816748. The GC content of the type genome is 61.06% and the genome length is 2.1 Mbp.

**Table:** 

**Description of *Candidatus* Fournierella excrementavium sp. nov.**
*Candidatus* Fournierella excrementavium (ex.cre.ment.a’vi.um. L. neut. n. *excrementum* excrement; L. fem. n. *avis* bird; N.L. gen. n. *excrementavium* of bird excrement)
A bacterial species identified by metagenomic analyses. This species includes all bacteria with genomes that show ≥95% average nucleotide identity (ANI) to the type genome, which has been assigned the MAG ID ChiHcec27-1717 and which is available via NCBI BioSample SAMN15816881. This is a new name for the alphanumeric GTDB species sp004558145. The GC content of the type genome is 63.90% and the genome length is 2.4 Mbp.
**Description of *Candidatus* Fournierella excrementigallinarum sp. nov.**
*Candidatus* Fournierella excrementigallinarum (ex.cre.men.ti.gal.li.na’rum. L. neut. n. *excrementum* excrement; L. fem. n. *gallina* hen; N.L. gen. n. *excrementigallinarum* of hen excrement)
A bacterial species identified by metagenomic analyses. This species includes all bacteria with genomes that show ≥95% average nucleotide identity (ANI) to the type genome, which has been assigned the MAG ID 1136 and which is available via NCBI BioSample SAMN15816650. The GC content of the type genome is 64.27% and the genome length is 2.1 Mbp.
**Description of *Candidatus* Fournierella merdavium sp. nov.**
*Candidatus* Fournierella merdavium (merd.a’vi.um. L. fem. n. *merda* faeces; L. fem. n. *avis* bird; N.L. gen. n. *merdavium* of bird faeces)
A bacterial species identified by metagenomic analyses. This species includes all bacteria with genomes that show ≥95% average nucleotide identity (ANI) to the type genome, which has been assigned the MAG ID ChiBcec4-1730 and which is available via NCBI BioSample SAMN15816653. The GC content of the type genome is 64.33% and the genome length is 2.6 Mbp.
**Description of *Candidatus* Fournierella merdigallinarum sp. nov.**
*Candidatus* Fournierella merdigallinarum (mer.di.gal.li.na’rum. L. fem. n. *merda* faeces; L. fem. n. *gallina* hen; N.L. gen. n. *merdigallinarum* of hen faeces)
A bacterial species identified by metagenomic analyses. This species includes all bacteria with genomes that show ≥95% average nucleotide identity (ANI) to the type genome, which has been assigned the MAG ID 6296 and which is available via NCBI BioSample SAMN15816675. The GC content of the type genome is 65.05% and the genome length is 2.4 Mbp.
**Description of *Candidatus* Fournierella merdipullorum sp. nov.**
*Candidatus* Fournierella merdipullorum (mer.di.pul.lo’rum. L. fem. n. *merda* faeces; L. masc. n. *pullus* a young chicken; N.L. gen. n. *merdipullorum* of the faeces of young chickens)
A bacterial species identified by metagenomic analyses. This species includes all bacteria with genomes that show ≥95% average nucleotide identity (ANI) to the type genome, which has been assigned the MAG ID ChiGjej4B4-18154 and which is available via NCBI BioSample SAMN15816693. The GC content of the type genome is 62.57% and the genome length is 2.5 Mbp.
**Description of *Candidatus* Fournierella pullicola sp. nov.**
*Candidatus* Fournierella pullicola (pul.li’co.la. L. masc. n. *pullus* a young chicken; L. suff. *-cola* inhabitant of; N.L. n. *pullicola* an inhabitant of young chickens)
A bacterial species identified by metagenomic analyses. This species includes all bacteria with genomes that show ≥95% average nucleotide identity (ANI) to the type genome, which has been assigned the MAG ID 2239 and which is available via NCBI BioSample SAMN15816745. The GC content of the type genome is 62.57% and the genome length is 2.4 Mbp.
**Description of *Candidatus* Fournierella pullistercoris sp. nov.**
*Candidatus* Fournierella pullistercoris (pul.li.ster’co.ris. L. masc. n. *pullus* a young chicken; L. neut. n. *stercus* dung; N.L. gen. n. *pullistercoris* of young chicken faeces)
A bacterial species identified by metagenomic analyses. This species includes all bacteria with genomes that show ≥95% average nucleotide identity (ANI) to the type genome, which has been assigned the MAG ID B5-2728 and which is available via NCBI BioSample SAMN15816762. The GC content of the type genome is 52.45% and the genome length is 1.7 Mbp.
**Description of *Candidatus* Fusicatenibacter intestinigallinarum sp. nov.**
*Candidatus* Fusicatenibacter intestinigallinarum (in.tes.ti.ni.gal.li.na’rum. L. neut. n. *intestinum* gut; L. fem. n. *gallina* hen; N.L. gen. n. *intestinigallinarum* of the gut of the hens)
A bacterial species identified by metagenomic analyses. This species includes all bacteria with genomes that show ≥95% average nucleotide identity (ANI) to the type genome, which has been assigned the MAG ID CHK185-5351 and which is available via NCBI BioSample SAMN15816585. The GC content of the type genome is 51.22% and the genome length is 2.9 Mbp.

**Table:** 

**Description of *Candidatus* Fusicatenibacter intestinipullorum sp. nov.**
*Candidatus* Fusicatenibacter intestinipullorum (in.tes.ti.ni.pul.lo’rum. L. neut. n. *intestinum* gut; L. masc. n. *pullus* a young chicken; N.L. gen. n. *intestinipullorum* of the gut of young chickens)
A bacterial species identified by metagenomic analyses. This species includes all bacteria with genomes that show ≥95% average nucleotide identity (ANI) to the type genome, which has been assigned the MAG ID ChiBcec11-5794 and which is available via NCBI BioSample SAMN15816833. This is a new name for the alphanumeric GTDB species sp900543115. The GC content of the type genome is 49.70% and the genome length is 2.6 Mbp.
**Description of *Candidatus* Fusicatenibacter merdavium sp. nov.**
*Candidatus* Fusicatenibacter merdavium (merd.a’vi.um. L. fem. n. *merda* faeces; L. fem. n. *avis* bird; N.L. gen. n. *merdavium* of bird faeces)
A bacterial species identified by metagenomic analyses. This species includes all bacteria with genomes that show ≥95% average nucleotide identity (ANI) to the type genome, which has been assigned the MAG ID CHK183-1962 and which is available via NCBI BioSample SAMN15816614. The GC content of the type genome is 51.02% and the genome length is 2.7 Mbp.
**Description of *Candidatus* Fusobacterium pullicola sp. nov.**
*Candidatus* Fusobacterium pullicola (pul.li’co.la. L. masc. n. *pullus* a young chicken; L. suff. *-cola* inhabitant of; N.L. n. *pullicola* inhabitant of a young chicken)
A bacterial species identified by metagenomic analyses. This species includes all bacteria with genomes that show ≥95% average nucleotide identity (ANI) to the type genome, which has been assigned the MAG ID A6-441 and which is available via NCBI BioSample SAMN15816927. This is a new name for the alphanumeric GTDB species sp900549465. Although GTDB has assigned this species to the genus it calls Fusobacterium_A, this genus designation cannot be incorporated into a well-formed binomial, so in naming this species, we have used the current validly published name for the genus. The GC content of the type genome is 29.86% and the genome length is 1.8 Mbp.
**Description of *Candidatus* Gallacutalibacter gen. nov.**
*Candidatus* Gallacutalibacter (Gall.a.cu.ta.li.bac’ter. L. masc. n. *gallus* chicken; N.L. masc. n. *Acutalibacter* a genus name; N.L. masc. n. *Gallacutalibacter* a genus related to the genus *Acutalibacter* but distinct from it and found in poultry)
A bacterial genus identified by metagenomic analyses. The genus includes all bacteria with genomes that show ≥60% average amino acid identity (AAI) to the type genome from the type species *Candidatus* Gallacutalibacter pullicola. This genus was identified but not named by Glendinning et al. (2020). This genus has been assigned by GTDB-Tk v1.3.0 working on GTDB Release 05-RS95 (Chaumeil et al., 2019; Parks et al., 2020) to the order *Oscillospirales* and to the family *Acutalibacteraceae*.
**Description of *Candidatus* Gallacutalibacter pullicola sp. nov.**
*Candidatus* Gallacutalibacter pullicola (pul.li’co.la. L. masc. n. *pullus* a young chicken; L. suff. *-cola* inhabitant of; N.L. n. *pullicola* an inhabitant of young chickens)
A bacterial species identified by metagenomic analyses. This species includes all bacteria with genomes that show ≥95% average nucleotide identity (ANI) to the type genome, which has been assigned the MAG ID ChiSjej1B19-7085 and which is available via NCBI BioSample SAMN15816935. The GC content of the type genome is 56.02% and the genome length is 2.5 Mbp.
**Description of *Candidatus* Gallacutalibacter pullistercoris sp. nov.**
*Candidatus* Gallacutalibacter pullistercoris (pul.li.ster’co.ris. L. masc. n. *pullus* a young chicken; L. neut. n. *stercus* dung; N.L. gen. n. *pullistercoris* of young chicken faeces)
A bacterial species identified by metagenomic analyses. This species includes all bacteria with genomes that show ≥95% average nucleotide identity (ANI) to the type genome, which has been assigned the MAG ID 13869 and which is available via NCBI BioSample SAMN15816961. The GC content of the type genome is 51.21% and the genome length is 2.4 Mbp.
**Description of *Candidatus* Gallacutalibacter stercoravium sp. nov.**
*Candidatus* Gallacutalibacter stercoravium (ster.cor.a’vi.um. L. neut. n. *stercus* dung; L. fem. n. *avis* bird; N.L. gen. n. *stercoravium* of bird faeces)
A bacterial species identified by metagenomic analyses. This species includes all bacteria with genomes that show ≥95% average nucleotide identity (ANI) to the type genome, which has been assigned the MAG ID CHK176-13069 and which is available via NCBI BioSample SAMN15816939. The GC content of the type genome is 51.38% and the genome length is 2.7 Mbp.
**Description of *Candidatus* Gallibacteroides gen. nov.**
*Candidatus* Gallibacteroides (Gal.li.bac.te.ro’i.des. L. masc. n. *gallus* chicken; N.L. masc. n. *Bacteroides* a genus name; N.L. masc. n. *Gallibacteroides* a genus related to the genus *Bacteroides* but distinct from it and found in poultry)
A bacterial genus identified by metagenomic analyses. The genus includes all bacteria with genomes that show ≥60% average amino acid identity (AAI) to the type genome from the type species *Candidatus* Gallibacteroides avistercoris. This genus has been assigned by GTDB-Tk v1.3.0 working on GTDB Release 05-RS95 (Chaumeil et al., 2019; Parks et al., 2020) to the order *Bacteroidales* and to the family *Barnesiellaceae*.

**Table:** 

**Description of *Candidatus* Gallibacteroides avistercoris sp. nov.**
*Candidatus* Gallibacteroides avistercoris (a.vi.ster’co.ris. L. fem. n. *avis* bird; L. neut. n. *stercus* dung; N.L. gen. n. *avistercoris* of bird faeces)
A bacterial species identified by metagenomic analyses. This species includes all bacteria with genomes that show ≥95% average nucleotide identity (ANI) to the type genome, which has been assigned the MAG ID CHK158-818 and which is available via NCBI BioSample SAMN15816984. The GC content of the type genome is 46.12% and the genome length is 2.3 Mbp.
**Description of *Candidatus* Galligastranaerophilus gen. nov.**
*Candidatus* Galligastranaerophilus (Gal.li.gastr.an.a.e.ro’phi.lus. L. masc. n. *gallus* chicken; N.L. masc. n. *Gastranaerophilus* a genus name; N.L. masc. n. *Galligastranaerophilus* a genus related to the genus *Gastranaerophilus* but distinct from it and found in poultry)
A bacterial genus identified by metagenomic analyses. The genus includes all bacteria with genomes that show ≥60% average amino acid identity (AAI) to the type genome from the type species *Candidatus* Galligastranaerophilus faecipullorum. This genus has been assigned by GTDB-Tk v1.3.0 working on GTDB Release 05-RS95 (Chaumeil et al., 2019; Parks et al., 2020) to the order *Gastranaerophilales* and to the family *Gastranaerophilaceae*.
**Description of *Candidatus* Galligastranaerophilus faecipullorum sp. nov.**
*Candidatus* Galligastranaerophilus faecipullorum (fae.ci.pul.lo’rum. L. fem. n. *faex, faecis* excrement; L. masc. n. *pullus* a young chicken; N.L. gen. n. *faecipullorum* of young chicken faeces)
A bacterial species identified by metagenomic analyses. This species includes all bacteria with genomes that show ≥95% average nucleotide identity (ANI) to the type genome, which has been assigned the MAG ID ChiW23-1657 and which is available via NCBI BioSample SAMN15816949. The GC content of the type genome is 39.52% and the genome length is 1.7 Mbp.
**Description of *Candidatus* Galligastranaerophilus gallistercoris sp. nov.**
*Candidatus* Galligastranaerophilus gallistercoris (gal.li.ster’co.ris. L. masc. n *gallus* chicken; L. neut. n. *stercus* dung; N.L. gen. n. *gallistercoris* of chicken faeces)
A bacterial species identified by metagenomic analyses. This species includes all bacteria with genomes that show ≥95% average nucleotide identity (ANI) to the type genome, which has been assigned the MAG ID CHK123-4750 and which is available via NCBI BioSample SAMN15816963. The GC content of the type genome is 35.45% and the genome length is 1.8 Mbp.
**Description of *Candidatus* Galligastranaerophilus intestinavium sp. nov.**
*Candidatus* Galligastranaerophilus intestinavium (in.tes.tin.a’vi.um. L. neut. n. *intestinum* gut; L. fem. n. *avis* bird; N.L. gen. n. *intestinavium* of the gut of birds)
A bacterial species identified by metagenomic analyses. This species includes all bacteria with genomes that show ≥95% average nucleotide identity (ANI) to the type genome, which has been assigned the MAG ID CHK152-2871 and which is available via NCBI BioSample SAMN15816967. The GC content of the type genome is 35.80% and the genome length is 1.6 Mbp.
**Description of *Candidatus* Galligastranaerophilus intestinigallinarum sp. nov.**
*Candidatus* Galligastranaerophilus intestinigallinarum (in.tes.ti.ni.gal.li.na’rum. L. neut. n. *intestinum* gut; L. fem. n. *gallina* hen; N.L. gen. n. *intestinigallinarum* of the gut of the hens)
A bacterial species identified by metagenomic analyses. This species includes all bacteria with genomes that show ≥95% average nucleotide identity (ANI) to the type genome, which has been assigned the MAG ID CHK123-3438 and which is available via NCBI BioSample SAMN15816973. The GC content of the type genome is 32.50% and the genome length is 1.7 Mbp.
**Description of *Candidatus* Gallilactobacillus gen. nov.**
*Candidatus* Gallilactobacillus (Gal.li.lac.to.ba.cil’lus. L. masc. n. *gallus* chicken; N.L. masc. n. *Lactobacillus* a genus name; N.L. masc. n. *Gallilactobacillus* a genus related to the genus *Lactobacillus* but distinct from it and found in poultry)
A bacterial genus identified by metagenomic analyses. The genus includes all bacteria with genomes that show ≥60% average amino acid identity (AAI) to the type genome from the type species *Candidatus* Gallilactobacillus intestinavium. This genus has been assigned by GTDB-Tk v1.3.0 working on GTDB Release 05-RS95 (Chaumeil et al., 2019; Parks et al., 2020) to the order *Lactobacillales* and to the family *Lactobacillaceae*.
**Description of *Candidatus* Gallilactobacillus intestinavium sp. nov.**
*Candidatus* Gallilactobacillus intestinavium (in.tes.tin.a’vi.um. L. neut. n. *intestinum* gut; L. fem. n. *avis* bird; N.L. gen. n. *intestinavium* of the gut of birds)
A bacterial species identified by metagenomic analyses. This species includes all bacteria with genomes that show ≥95% average nucleotide identity (ANI) to the type genome, which has been assigned the MAG ID C6-149 and which is available via NCBI BioSample SAMN15816970. The GC content of the type genome is 29.69% and the genome length is 1.2 Mbp.

**Table:** 

**Description of *Candidatus* Gallimonas gallistercoris sp. nov.**
*Candidatus* Gallimonas gallistercoris (gal.li.ster’co.ris. L. masc. n *gallus* chicken; L. neut. n. *stercus* dung; N.L. gen. n. *gallistercoris* of chicken faeces)
A bacterial species identified by metagenomic analyses. This species includes all bacteria with genomes that show ≥95% average nucleotide identity (ANI) to the type genome, which has been assigned the MAG ID CHK156-179 and which is available via NCBI BioSample SAMN15816677. The GC content of the type genome is 58.55% and the genome length is 1.6 Mbp.
**Description of *Candidatus* Gallimonas intestinavium sp. nov.**
*Candidatus* Gallimonas intestinavium (in.tes.tin.a’vi.um. L. neut. n. *intestinum* gut; L. fem. n. *avis* bird; N.L. gen. n. *intestinavium* of the gut of birds)
A bacterial species identified by metagenomic analyses. This species includes all bacteria with genomes that show ≥95% average nucleotide identity (ANI) to the type genome, which has been assigned the MAG ID ChiW7-2402 and which is available via NCBI BioSample SAMN15816844. This is a new name for the alphanumeric GTDB species sp003343805. The GC content of the type genome is 58.63% and the genome length is 1.8 Mbp.
**Description of *Candidatus* Gallimonas intestinigallinarum sp. nov.**
*Candidatus* Gallimonas intestinigallinarum (in.tes.ti.ni.gal.li.na’rum. L. neut. n. *intestinum* gut; L. fem. n. *gallina* hen; N.L. gen. n. *intestinigallinarum* of the gut of the hens)
A bacterial species identified by metagenomic analyses. This species includes all bacteria with genomes that show ≥95% average nucleotide identity (ANI) to the type genome, which has been assigned the MAG ID CHK33-5263 and which is available via NCBI BioSample SAMN15816692. The GC content of the type genome is 57.82% and the genome length is 1.6 Mbp.
**Description of *Candidatus* Gallipaludibacter gen. nov.**
*Candidatus* Gallipaludibacter (Gal.li.pa.lu.di.bac’ter. L. masc. n. *gallus* chicken; N.L. masc. n. *Paludibacter* a genus name; N.L. masc. n. *Gallipaludibacter* a genus related to the genus *Paludibacter* but distinct from it and found in poultry)
A bacterial genus identified by metagenomic analyses. The genus includes all bacteria with genomes that show ≥60% average amino acid identity (AAI) to the type genome from the type species *Candidatus* Gallipaludibacter merdavium. This genus has been assigned by GTDB-Tk v1.3.0 working on GTDB Release 05-RS95 (Chaumeil et al., 2019; Parks et al., 2020) to the order *Bacteroidales* and to the family *Paludibacteraceae*.
**Description of *Candidatus* Gallipaludibacter merdavium sp. nov.**
*Candidatus* Gallipaludibacter merdavium (merd.a’vi.um. L. fem. n. *merda* faeces; L. fem. n. *avis* bird; N.L. gen. n. *merdavium* of bird faeces)
A bacterial species identified by metagenomic analyses. This species includes all bacteria with genomes that show ≥95% average nucleotide identity (ANI) to the type genome, which has been assigned the MAG ID G3-3990 and which is available via NCBI BioSample SAMN15816954. The GC content of the type genome is 41.91% and the genome length is 2.9 Mbp.
**Description of *Candidatus* Gallitreponema gen. nov.**
*Candidatus* Gallitreponema (Gal.li.tre.po.ne’ma. L. masc. n. *gallus* chicken; N.L. neut. n. *Treponema* a genus name; N.L. neut. n. *Gallitreponema* a genus related to the genus *Treponema* but distinct from it and found in poultry)
A bacterial genus identified by metagenomic analyses. The genus includes all bacteria with genomes that show ≥60% average amino acid identity (AAI) to the type genome from the type species *Candidatus* Gallitreponema excrementavium. This genus has been assigned by GTDB-Tk v1.3.0 working on GTDB Release 05-RS95 (Chaumeil et al., 2019; Parks et al., 2020) to the order *Treponematales* and to the family *Treponemataceae*.
**Description of *Candidatus* Gallitreponema excrementavium sp. nov.**
*Candidatus* Gallitreponema excrementavium (ex.cre.ment.a’vi.um. L. neut. n. *excrementum* excrement; L. fem. n. *avis* bird; N.L. gen. n. *excrementavium* of bird excrement)
A bacterial species identified by metagenomic analyses. This species includes all bacteria with genomes that show ≥95% average nucleotide identity (ANI) to the type genome, which has been assigned the MAG ID 10532 and which is available via NCBI BioSample SAMN15816962. The GC content of the type genome is 40.13% and the genome length is 2.4 Mbp.
**Description of *Candidatus* Galloscillospira gen. nov.**
*Candidatus* Galloscillospira (Gall.os.cil.lo.spi’ra. L. masc. n. *gallus* chicken; N.L. fem. n. *Oscillospira* a genus name; N.L. fem. n. *Galloscillospira*. a genus related to the genus *Oscillospira* but distinct from it and found in poultry)
A bacterial genus identified by metagenomic analyses. The genus includes all bacteria with genomes that show ≥60% average amino acid identity (AAI) to the type genome from the type species *Candidatus* Galloscillospira excrementipullorum. This genus belongs to the new family *Candidatus* Galloscillospiraceae.

**Table:** 

**Description of *Candidatus* Galloscillospira excrementavium sp. nov.**
*Candidatus* Galloscillospira excrementavium (ex.cre.ment.a’vi.um. L. neut. n. *excrementum* excrement; L. fem. n. *avis* bird; N.L. gen. n. *excrementavium* of bird excrement)
A bacterial species identified by metagenomic analyses. This species includes all bacteria with genomes that show ≥95% average nucleotide identity (ANI) to the type genome, which has been assigned the MAG ID ChiSjej1B19-13426 and which is available via NCBI BioSample SAMN15816937. The GC content of the type genome is 65.18% and the genome length is 2.1 Mbp.
**Description of *Candidatus* Galloscillospira excrementipullorum sp. nov.**
*Candidatus* Galloscillospira excrementipullorum (ex.cre.men.ti.pul.lo’rum. L. neut. n. *excrementum* excrement; L. masc. n. *pullus* a young chicken; N.L. gen. n. *excrementipullorum* of young chicken excrement)
A bacterial species identified by metagenomic analyses. This species includes all bacteria with genomes that show ≥95% average nucleotide identity (ANI) to the type genome, which has been assigned the MAG ID ChiHjej8B7-10251 and which is available via NCBI BioSample SAMN15816946. The GC content of the type genome is 60.78% and the genome length is 1.6 Mbp.
**Description of *Candidatus* Galloscillospira stercoripullorum sp. nov.**
*Candidatus* Galloscillospira stercoripullorum (ster.co.ri.pul.lo’rum. L. neut. n. *stercus* dung; L. masc. n. *pullus* a young chicken; N.L. gen. n. *stercoripullorum* of the faceces of young chickens)
A bacterial species identified by metagenomic analyses. This species includes all bacteria with genomes that show ≥95% average nucleotide identity (ANI) to the type genome, which has been assigned the MAG ID CHK33-6455 and which is available via NCBI BioSample SAMN15816975. The GC content of the type genome is 62.96% and the genome length is 1.9 Mbp.
**Description of *Candidatus* Galloscillospiraceae fam. nov.**
*Candidatus* Galloscillospiraceae (Gall.os.cil.lo.spi.ra.ce’ae. N.L. fem. n. *Galloscillospira*. type genus of the family genus; N.L. suff. *–ceae* to denote a family; N.L. fem. pl. n. *Galloscillospiraceae*, the family of the genus *Galloscillospira*)
A bacterial family identified by metagenomic analyses. This family has been defined by the absence of a family assignment for the type species when GTDB-Tk v1.3.0 is applied to GTDB Release 05-RS95 (Chaumeil et al., 2019; Parks et al., 2020). GTDB assigns the type species and thus the family to the order *Oscillospirales*.
**Description of *Candidatus* Gemmiger avicola sp. nov.**
*Candidatus* Gemmiger avicola (a.vi’co.la. L. fem. n. *avis* bird; L. suff. *-cola* inhabitant of; N.L. n. *avicola* inhabitant of birds)
A bacterial species identified by metagenomic analyses. This species includes all bacteria with genomes that show ≥95% average nucleotide identity (ANI) to the type genome, which has been assigned the MAG ID ChiBcec8-13705 and which is available via NCBI BioSample SAMN15816825. This is a new name for the alphanumeric GTDB species sp900548355. The GC content of the type genome is 61.69% and the genome length is 2.3 Mbp.
**Description of *Candidatus* Gemmiger avistercoris sp. nov.**
*Candidatus* Gemmiger avistercoris (a.vi.ster’co.ris. L. fem. n. *avis* bird; L. neut. n. *stercus* dung; N.L. gen. n. *avistercoris* of bird faeces)
A bacterial species identified by metagenomic analyses. This species includes all bacteria with genomes that show ≥95% average nucleotide identity (ANI) to the type genome, which has been assigned the MAG ID CHK188-11489 and which is available via NCBI BioSample SAMN15816604. The GC content of the type genome is 63.30% and the genome length is 2.2 Mbp.
**Description of *Candidatus* Gemmiger avium sp. nov.**
*Candidatus* Gemmiger avium (a’vi.um. L. fem. pl. n. *avium* of birds)
A bacterial species identified by metagenomic analyses. This species includes all bacteria with genomes that show ≥95% average nucleotide identity (ANI) to the type genome, which has been assigned the MAG ID ChiGjej4B4-15321 and which is available via NCBI BioSample SAMN15816926. This is a new name for the alphanumeric GTDB species sp002160955. Although GTDB has assigned this species to the genus it calls Gemmiger_A, this genus designation cannot be incorporated into a well-formed binomial, so in naming this species, we have used the current validly published name for the genus. The GC content of the type genome is 62.71% and the genome length is 2.6 Mbp.
**Description of *Candidatus* Gemmiger excrementavium sp. nov.**
*Candidatus* Gemmiger excrementavium (ex.cre.ment.a’vi.um. L. neut. n. *excrementum* excrement; L. fem. n. *avis* bird; N.L. gen. n. *excrementavium* of bird excrement)
A bacterial species identified by metagenomic analyses. This species includes all bacteria with genomes that show ≥95% average nucleotide identity (ANI) to the type genome, which has been assigned the MAG ID 3436 and which is available via NCBI BioSample SAMN15816690. The GC content of the type genome is 60.89% and the genome length is 2.5 Mbp.

**Table:** 

**Description of *Candidatus* Gemmiger excrementigallinarum sp. nov.**
*Candidatus* Gemmiger excrementigallinarum (ex.cre.men.ti.gal.li.na’rum. L. neut. n. *excrementum* excrement; L. fem. n. *avis* bird; N.L. gen. n. *excrementigallinarum* of hen excrement)
A bacterial species identified by metagenomic analyses. This species includes all bacteria with genomes that show ≥95% average nucleotide identity (ANI) to the type genome, which has been assigned the MAG ID ChiSxjej1B13-11774 and which is available via NCBI BioSample SAMN15816691. The GC content of the type genome is 58.73% and the genome length is 2.4 Mbp.
**Description of *Candidatus* Gemmiger excrementipullorum sp. nov.**
*Candidatus* Gemmiger excrementipullorum (ex.cre.men.ti.pul.lo’rum. L. neut. n. *excrementum* excrement; L. masc. n. *pullus* a young chicken; N.L. gen. n. *excrementipullorum* of young chicken excrement)
A bacterial species identified by metagenomic analyses. This species includes all bacteria with genomes that show ≥95% average nucleotide identity (ANI) to the type genome, which has been assigned the MAG ID ChiHecec2B26-7398 and which is available via NCBI BioSample SAMN15816726. The GC content of the type genome is 64.11% and the genome length is 2.1 Mbp.
**Description of *Candidatus* Gemmiger faecavium sp. nov.**
*Candidatus* Gemmiger faecavium (faec.a’vi.um. L. fem. n. *faex, faecis* excrement; L. fem. n. *avis* bird; N.L. gen. n. *faecavium* of bird faeces)
A bacterial species identified by metagenomic analyses. This species includes all bacteria with genomes that show ≥95% average nucleotide identity (ANI) to the type genome, which has been assigned the MAG ID ChiSxjej1B13-1558 and which is available via NCBI BioSample SAMN15816740. The GC content of the type genome is 60.04% and the genome length is 2.5 Mbp.
**Description of *Candidatus* Gemmiger faecigallinarum sp. nov.**
*Candidatus* Gemmiger faecigallinarum (fae.ci.gal.li.na’rum. L. fem. n. *faex, faecis* excrement; L. fem. n. *gallina* hen; N.L. gen. n. *faecigallinarum* of hen faeces)
A bacterial species identified by metagenomic analyses. This species includes all bacteria with genomes that show ≥95% average nucleotide identity (ANI) to the type genome, which has been assigned the MAG ID 14795 and which is available via NCBI BioSample SAMN15816795. The GC content of the type genome is 63.90% and the genome length is 2.6 Mbp.
**Description of *Candidatus* Gemmiger stercoravium sp. nov.**
*Candidatus* Gemmiger stercoravium (ster.cor.a’vi.um. L. neut. n. *stercus* dung; L. fem. n. *avis* bird; N.L. gen. n. *stercoravium* of bird faeces)
A bacterial species identified by metagenomic analyses. This species includes all bacteria with genomes that show ≥95% average nucleotide identity (ANI) to the type genome, which has been assigned the MAG ID ChiBcolR8-2160 and which is available via NCBI BioSample SAMN15816579. The GC content of the type genome is 65.46% and the genome length is 2.5 Mbp.
**Description of *Candidatus* Gemmiger stercorigallinarum sp. nov.**
*Candidatus* Gemmiger stercorigallinarum (ster.co.ri.gal.li.na’rum. L. neut. n. *stercus* dung; L. fem. n. *gallina* hen; N.L. gen. n. *stercorigallinarum* of hen faeces)
A bacterial species identified by metagenomic analyses. This species includes all bacteria with genomes that show ≥95% average nucleotide identity (ANI) to the type genome, which has been assigned the MAG ID CHK183-27628 and which is available via NCBI BioSample SAMN15816586. The GC content of the type genome is 64.08% and the genome length is 2.5 Mbp.
**Description of *Candidatus* Gemmiger stercoripullorum sp. nov.**
*Candidatus* Gemmiger stercoripullorum (ster.co.ri.pul.lo’rum. L. neut. n. *stercus* dung; L. masc. n. *pullus* a young chicken; N.L. gen. n. *stercoripullorum* of the faceces of young chickens)
A bacterial species identified by metagenomic analyses. This species includes all bacteria with genomes that show ≥95% average nucleotide identity (ANI) to the type genome, which has been assigned the MAG ID ChiSjej4B22-15101 and which is available via NCBI BioSample SAMN15816589. The GC content of the type genome is 64.37% and the genome length is 2.2 Mbp.
**Description of *Candidatus* Gordonibacter avicola sp. nov.**
*Candidatus* Gordonibacter avicola (a.vi’co.la. L. fem. n. *avis* bird; L. suff. *-cola* inhabitant of; N.L. n. *avicola* inhabitant of birds)
A bacterial species identified by metagenomic analyses. This species includes all bacteria with genomes that show ≥95% average nucleotide identity (ANI) to the type genome, which has been assigned the MAG ID ChiSxjej6B18-1472 and which is available via NCBI BioSample SAMN15816757. The GC content of the type genome is 59.91% and the genome length is 3.1 Mbp.

**Table:** 

**Description of *Candidatus* Halomonas stercoripullorum sp. nov.**
*Candidatus* Halomonas stercoripullorum (ster.co.ri.pul.lo’rum. L. neut. n. *stercus* dung; L. masc. n. *pullus* a young chicken; N.L. gen. n. *stercoripullorum* of the faceces of young chickens)
A bacterial species identified by metagenomic analyses. This species includes all bacteria with genomes that show ≥95% average nucleotide identity (ANI) to the type genome, which has been assigned the MAG ID 1193 and which is available via NCBI BioSample SAMN15816734. The GC content of the type genome is 59.74% and the genome length is 2.1 Mbp.
**Description of *Candidatus* Helicobacter avicola sp. nov.**
*Candidatus* Helicobacter avicola (a.vi’co.la. L. fem. n. *avis* bird; L. suff. *-cola* inhabitant of; N.L. n. *avicola* inhabitant of birds)
A bacterial species identified by metagenomic analyses. This species includes all bacteria with genomes that show ≥95% average nucleotide identity (ANI) to the type genome, which has been assigned the MAG ID 14449 and which is available via NCBI BioSample SAMN15816913. Although GTDB has assigned this species to the genus it calls Helicobacter_F, this genus designation cannot be incorporated into a well-formed binomial, so in naming this species, we have used the current validly published name for the genus. The GC content of the type genome is 42.81% and the genome length is 1.7 Mbp.
**Description of *Candidatus* Helicobacter avistercoris sp. nov.**
*Candidatus* Helicobacter avistercoris (a.vi.ster’co.ris. L. fem. n. *avis* bird; L. neut. n. *stercus* dung; N.L. gen. n. *avistercoris* of bird faeces)
A bacterial species identified by metagenomic analyses. This species includes all bacteria with genomes that show ≥95% average nucleotide identity (ANI) to the type genome, which has been assigned the MAG ID CHK158-8274 and which is available via NCBI BioSample SAMN15816903. Although GTDB has assigned this species to the genus it calls Helicobacter_G, this genus designation cannot be incorporated into a well-formed binomial, so in naming this species, we have used the current validly published name for the genus. The GC content of the type genome is 38.34% and the genome length is 1.4 Mbp.
**Description of *Candidatus* Hungatella pullicola sp. nov.**
*Candidatus* Hungatella pullicola (pul.li’co.la. L. masc. n. *pullus* a young chicken; L. suff. *-cola* inhabitant of; N.L. n. *pullicola* an inhabitant of young chickens)
A bacterial species identified by metagenomic analyses. This species includes all bacteria with genomes that show ≥95% average nucleotide identity (ANI) to the type genome, which has been assigned the MAG ID CHK186-17716 and which is available via NCBI BioSample SAMN15816620. The GC content of the type genome is 44.57% and the genome length is 2.9 Mbp.
**Description of *Candidatus* Ignatzschineria merdigallinarum sp. nov.**
*Candidatus* Ignatzschineria merdigallinarum (mer.di.gal.li.na’rum. L. fem. n. *merda* faeces; L. fem. n. *gallina* hen; N.L. gen. n. *merdigallinarum* of hen faeces)
A bacterial species identified by metagenomic analyses. This species includes all bacteria with genomes that show ≥95% average nucleotide identity (ANI) to the type genome, which has been assigned the MAG ID CHK160-9182 and which is available via NCBI BioSample SAMN15816769. The GC content of the type genome is 39.66% and the genome length is 2.3 Mbp.
**Description of *Candidatus* Intestinimonas merdavium sp. nov.**
*Candidatus* Intestinimonas merdavium (merd.a’vi.um. L. fem. n. *merda* faeces; L. fem. n. *avis* bird; N.L. gen. n. *merdavium* of bird faeces)
A bacterial species identified by metagenomic analyses. This species includes all bacteria with genomes that show ≥95% average nucleotide identity (ANI) to the type genome, which has been assigned the MAG ID CHK33-7979 and which is available via NCBI BioSample SAMN15816706. The GC content of the type genome is 61.44% and the genome length is 2.4 Mbp.
**Description of *Candidatus* Intestinimonas pullistercoris sp. nov.**
*Candidatus* Intestinimonas pullistercoris (pul.li.ster’co.ris. L. masc. n. *pullus* a young chicken; L. neut. n. *stercus* dung; N.L. gen. n. *pullistercoris* of young chicken faeces)
A bacterial species identified by metagenomic analyses. This species includes all bacteria with genomes that show ≥95% average nucleotide identity (ANI) to the type genome, which has been assigned the MAG ID CHK186-1790 and which is available via NCBI BioSample SAMN15816581. The GC content of the type genome is 64.76% and the genome length is 2.4 Mbp.
**Description of *Candidatus* Intestinimonas stercoravium sp. nov.**
*Candidatus* Intestinimonas stercoravium (ster.cor.a’vi.um. L. neut. n. *stercus* dung; L. fem. n. *avis* bird; N.L. gen. n. *stercoravium* of bird faeces)
A bacterial species identified by metagenomic analyses. This species includes all bacteria with genomes that show ≥95% average nucleotide identity (ANI) to the type genome, which has been assigned the MAG ID ChiBcolR2-622 and which is available via NCBI BioSample SAMN15816599. The GC content of the type genome is 65.33% and the genome length is 2.2 Mbp.

**Table:** 

**Description of *Candidatus* Intestinimonas stercorigallinarum sp. nov.**
*Candidatus* Intestinimonas stercorigallinarum (ster.co.ri.gal.li.na’rum. L. neut. n. *stercus* dung; L. fem. n. *gallina* hen; N.L. gen. n. *stercorigallinarum* of hen faeces)
A bacterial species identified by metagenomic analyses. This species includes all bacteria with genomes that show ≥95% average nucleotide identity (ANI) to the type genome, which has been assigned the MAG ID ChiSxjej2B14-1745 and which is available via NCBI BioSample SAMN15816719. The GC content of the type genome is 65.69% and the genome length is 2.1 Mbp.
**Description of *Candidatus* Janibacter merdipullorum sp. nov.**
*Candidatus* Janibacter merdipullorum (mer.di.pul.lo’rum. L. fem. n. *merda* faeces; L. masc. n. *pullus* a young chicken; N.L. gen. n. *merdipullorum* of the faeces of young chickens)
A bacterial species identified by metagenomic analyses. This species includes all bacteria with genomes that show ≥95% average nucleotide identity (ANI) to the type genome, which has been assigned the MAG ID ChiHjej13B12-21492 and which is available via NCBI BioSample SAMN15816685. The GC content of the type genome is 71.49% and the genome length is 2.8 Mbp.
**Description of *Candidatus* Jeotgalibaca merdavium sp. nov.**
*Candidatus* Jeotgalibaca merdavium (merd.a’vi.um. L. fem. n. *merda* faeces; L. fem. n. *avis* bird; N.L. gen. n. *merdavium* of bird faeces)
A bacterial species identified by metagenomic analyses. This species includes all bacteria with genomes that show ≥95% average nucleotide identity (ANI) to the type genome, which has been assigned the MAG ID CHK171-505 and which is available via NCBI BioSample SAMN15816828. This is a new name for the alphanumeric GTDB species sp001975685. The GC content of the type genome is 38.39% and the genome length is 2.0 Mbp.
**Description of *Candidatus* Jeotgalibaca pullicola sp. nov.**
*Candidatus* Jeotgalibaca pullicola (pul.li’co.la. L. masc. n. *pullus* a young chicken; L. suff. *-cola* inhabitant of; N.L. n. *pullicola* an inhabitant of young chickens)
A bacterial species identified by metagenomic analyses. This species includes all bacteria with genomes that show ≥95% average nucleotide identity (ANI) to the type genome, which has been assigned the MAG ID CHK172-9797 and which is available via NCBI BioSample SAMN15816826. This is a new name for the alphanumeric GTDB species sp003955755. The GC content of the type genome is 36.93% and the genome length is 2.6 Mbp.
**Description of *Candidatus* Jeotgalicoccus stercoravium sp. nov.**
*Candidatus* Jeotgalicoccus stercoravium (ster.cor.a’vi.um. L. neut. n. *stercus* dung; L. fem. n. *avis* bird; N.L. gen. n. *stercoravium* of bird faeces)
A bacterial species identified by metagenomic analyses. This species includes all bacteria with genomes that show ≥95% average nucleotide identity (ANI) to the type genome, which has been assigned the MAG ID CHK148-7025 and which is available via NCBI BioSample SAMN15816765. The GC content of the type genome is 36.17% and the genome length is 1.7 Mbp.
**Description of *Candidatus* Kurthia intestinigallinarum sp. nov.**
*Candidatus* Kurthia intestinigallinarum (in.tes.ti.ni.gal.li.na’rum. L. neut. n. *intestinum* gut; L. fem. n. *gallina* hen; N.L. gen. n. *intestinigallinarum* of the gut of the hens)
A bacterial species identified by metagenomic analyses. This species includes all bacteria with genomes that show ≥95% average nucleotide identity (ANI) to the type genome, which has been assigned the MAG ID CHK171-3164 and which is available via NCBI BioSample SAMN15816863. This is a new name for the alphanumeric GTDB species sp002418445. The GC content of the type genome is 39.62% and the genome length is 2.9 Mbp.
**Description of *Candidatus* Lachnoclostridium avicola sp. nov.**
*Candidatus* Lachnoclostridium avicola (a.vi’co.la. L. fem. n. *avis* bird; L. suff. *-cola* inhabitant of; N.L. n. *avicola* inhabitant of birds)
A bacterial species identified by metagenomic analyses. This species includes all bacteria with genomes that show ≥95% average nucleotide identity (ANI) to the type genome, which has been assigned the MAG ID CHK190-19777 and which is available via NCBI BioSample SAMN15816889. Although GTDB has assigned this species to the genus it calls Lachnoclostridium_A, this genus designation cannot be incorporated into a well-formed binomial, so in naming this species, we have used the current validly published name for the genus. The GC content of the type genome is 54.75% and the genome length is 2.9 Mbp.
**Description of *Candidatus* Lachnoclostridium pullistercoris sp. nov.**
*Candidatus* Lachnoclostridium pullistercoris (pul.li.ster’co.ris. L. masc. n. *pullus* a young chicken; L. neut. n. *stercus* dung; N.L. gen. n. *pullistercoris* of young chicken faeces)
A bacterial species identified by metagenomic analyses. This species includes all bacteria with genomes that show ≥95% average nucleotide identity (ANI) to the type genome, which has been assigned the MAG ID CHK183-5548 and which is available via NCBI BioSample SAMN15816884. Although GTDB has assigned this species to the genus it calls Lachnoclostridium_A, this genus designation cannot be incorporated into a well-formed binomial, so in naming this species, we have used the current validly published name for the genus. The GC content of the type genome is 54.35% and the genome length is 2.8 Mbp.

**Table:** 

**Description of *Candidatus* Lachnoclostridium stercoravium sp. nov.**
*Candidatus* Lachnoclostridium stercoravium (ster.cor.a’vi.um. L. neut. n. *stercus* dung; L. fem. n. *avis* bird; N.L. gen. n. *stercoravium* of bird faeces)
A bacterial species identified by metagenomic analyses. This species includes all bacteria with genomes that show ≥95% average nucleotide identity (ANI) to the type genome, which has been assigned the MAG ID CHK178-16964 and which is available via NCBI BioSample SAMN15816887. Although GTDB has assigned this species to the genus it calls Lachnoclostridium_A, this genus designation cannot be incorporated into a well-formed binomial, so in naming this species, we have used the current validly published name for the genus. The GC content of the type genome is 49.55% and the genome length is 3.0 Mbp.
**Description of *Candidatus* Lachnoclostridium stercorigallinarum sp. nov.**
*Candidatus* Lachnoclostridium stercorigallinarum (ster.co.ri.gal.li.na’rum. L. neut. n. *stercus* dung; L. fem. n. *gallina* hen; N.L. gen. n. *stercorigallinarum* of hen faeces)
A bacterial species identified by metagenomic analyses. This species includes all bacteria with genomes that show ≥95% average nucleotide identity (ANI) to the type genome, which has been assigned the MAG ID ChiBcec1-1093 and which is available via NCBI BioSample SAMN15816897. Although GTDB has assigned this species to the genus it calls Lachnoclostridium_A, this genus designation cannot be incorporated into a well-formed binomial, so in naming this species, we have used the current validly published name for the genus. The GC content of the type genome is 54.29% and the genome length is 2.4 Mbp.
**Description of *Candidatus* Lachnoclostridium stercoripullorum sp. nov.**
*Candidatus* Lachnoclostridium stercoripullorum (ster.co.ri.pul.lo’rum. L. neut. n. *stercus* dung; L. masc. n. *pullus* a young chicken; N.L. gen. n. *stercoripullorum* of the faceces of young chickens)
A bacterial species identified by metagenomic analyses. This species includes all bacteria with genomes that show ≥95% average nucleotide identity (ANI) to the type genome, which has been assigned the MAG ID ChiGjej4B4-12881 and which is available via NCBI BioSample SAMN15816908. Although GTDB has assigned this species to the genus it calls Lachnoclostridium_A, this genus designation cannot be incorporated into a well-formed binomial, so in naming this species, we have used the current validly published name for the genus. The GC content of the type genome is 59.38% and the genome length is 2.3 Mbp.
**Description of *Candidatus* Lactobacillus pullistercoris sp. nov.**
*Candidatus* Lactobacillus pullistercoris (pul.li.ster’co.ris. L. masc. n. *pullus* a young chicken; L. neut. n. *stercus* dung; N.L. gen. n. *pullistercoris* of young chicken faeces)
A bacterial species identified by metagenomic analyses. This species includes all bacteria with genomes that show ≥95% average nucleotide identity (ANI) to the type genome, which has been assigned the MAG ID F6-686 and which is available via NCBI BioSample SAMN15816686. The GC content of the type genome is 34.54% and the genome length is 1.7 Mbp.
**Description of *Candidatus* Lawsonibacter pullicola sp. nov.**
*Candidatus* Lawsonibacter pullicola (pul.li’co.la. L. masc. n. *pullus* a young chicken; L. suff. *-cola* inhabitant of; N.L. n. *pullicola* an inhabitant of young chickens)
A bacterial species identified by metagenomic analyses. This species includes all bacteria with genomes that show ≥95% average nucleotide identity (ANI) to the type genome, which has been assigned the MAG ID CHK178-3907 and which is available via NCBI BioSample SAMN15816869. This is a new name for the alphanumeric GTDB species sp002160305. The GC content of the type genome is 62.98% and the genome length is 2.3 Mbp.
**Description of *Candidatus* Levilactobacillus faecigallinarum sp. nov.**
*Candidatus* Levilactobacillus faecigallinarum (fae.ci.gal.li.na’rum. L. fem. n. *faex, faecis* excrement; L. fem. n. *gallina* hen; N.L. gen. n. *faecigallinarum* of hen faeces)
A bacterial species identified by metagenomic analyses. This species includes all bacteria with genomes that show ≥95% average nucleotide identity (ANI) to the type genome, which has been assigned the MAG ID CHK173-259 and which is available via NCBI BioSample SAMN15816755. The GC content of the type genome is 52.18% and the genome length is 1.8 Mbp.
**Description of *Candidatus* Ligilactobacillus avistercoris sp. nov.**
*Candidatus* Ligilactobacillus avistercoris (a.vi.ster’co.ris. L. fem. n. *avis* bird; L. neut. n. *stercus* dung; N.L. gen. n. *avistercoris* of bird faeces)
A bacterial species identified by metagenomic analyses. This species includes all bacteria with genomes that show ≥95% average nucleotide identity (ANI) to the type genome, which has been assigned the MAG ID ChiBile7-59 and which is available via NCBI BioSample SAMN15816642. The GC content of the type genome is 51.08% and the genome length is 1.2 Mbp.

**Table:** 

**Description of *Candidatus* Ligilactobacillus excrementavium sp. nov.**
*Candidatus* Ligilactobacillus excrementavium (ex.cre.ment.a’vi.um. L. neut. n. *excrementum* excrement; L. fem. n. *avis* bird; N.L. gen. n. *excrementavium* of bird excrement)
A bacterial species identified by metagenomic analyses. This species includes all bacteria with genomes that show ≥95% average nucleotide identity (ANI) to the type genome, which has been assigned the MAG ID 2259 and which is available via NCBI BioSample SAMN15816683. The GC content of the type genome is 38.00% and the genome length is 1.9 Mbp.
**Description of *Candidatus* Ligilactobacillus excrementigallinarum sp. nov.**
*Candidatus* Ligilactobacillus excrementigallinarum (ex.cre.men.ti.gal.li.na’rum. L. neut. n. *excrementum* excrement; L. fem. n. *gallina* hen; N.L. gen. n. *excrementigallinarum* of hen excrement)
A bacterial species identified by metagenomic analyses. This species includes all bacteria with genomes that show ≥95% average nucleotide identity (ANI) to the type genome, which has been assigned the MAG ID 6627 and which is available via NCBI BioSample SAMN15816741. The GC content of the type genome is 34.16% and the genome length is 1.2 Mbp.
**Description of *Candidatus* Ligilactobacillus excrementipullorum sp. nov.**
*Candidatus* Ligilactobacillus excrementipullorum (ex.cre.men.ti.pul.lo’rum. L. neut. n. *excrementum* excrement; L. masc. n. *pullus* a young chicken; N.L. gen. n. *excrementipullorum* of young chicken excrement)
A bacterial species identified by metagenomic analyses. This species includes all bacteria with genomes that show ≥95% average nucleotide identity (ANI) to the type genome, which has been assigned the MAG ID CHK171-2193 and which is available via NCBI BioSample SAMN15816749. The GC content of the type genome is 42.06% and the genome length is 2.0 Mbp.
**Description of *Candidatus* Ligilactobacillus faecavium sp. nov.**
*Candidatus* Ligilactobacillus faecavium (faec.a’vi.um. L. fem. n. *faex, faecis* excrement; L. fem. n. *avis* bird; N.L. gen. n. *faecavium* of bird faeces)
A bacterial species identified by metagenomic analyses. This species includes all bacteria with genomes that show ≥95% average nucleotide identity (ANI) to the type genome, which has been assigned the MAG ID 3439 and which is available via NCBI BioSample SAMN15816798. The GC content of the type genome is 40.05% and the genome length is 1.3 Mbp.
**Description of *Candidatus* Limadaptatus gen. nov.**
*Candidatus* Limadaptatus (Lim.a.dap.ta’tus. L. masc. n. *limus* dung; L. past part. masc. *adaptatus* adapted to; N.L. masc. n. *Limadaptatus* a microbe associated with faeces)
A bacterial genus identified by metagenomic analyses. The genus includes all bacteria with genomes that show ≥60% average amino acid identity (AAI) to the type genome from the type species *Candidatus* Limadaptatus stercoripullorum. This is a name for the alphanumeric GTDB genus UMGS1688. This genus has been assigned by GTDB-Tk v1.3.0 working on GTDB Release 05-RS95 (Chaumeil et al., 2019; Parks et al., 2020) to the order *Christensenellales* and to the family *CAG-917*.
**Description of *Candidatus* Limadaptatus stercoravium sp. nov.**
*Candidatus* Limadaptatus stercoravium (ster.cor.a’vi.um. L. neut. n. *stercus* dung; L. fem. n. *avis* bird; N.L. gen. n. *stercoravium* of bird faeces)
A bacterial species identified by metagenomic analyses. This species includes all bacteria with genomes that show ≥95% average nucleotide identity (ANI) to the type genome, which has been assigned the MAG ID CHK154-227 and which is available via NCBI BioSample SAMN15817097. The GC content of the type genome is 58.03% and the genome length is 1.4 Mbp.
**Description of *Candidatus* Limadaptatus stercorigallinarum sp. nov.**
*Candidatus* Limadaptatus stercorigallinarum (ster.co.ri.gal.li.na’rum. L. neut. n. *stercus* dung; L. fem. n. *gallina* hen; N.L. gen. n. *stercorigallinarum* of hen faeces)
A bacterial species identified by metagenomic analyses. This species includes all bacteria with genomes that show ≥95% average nucleotide identity (ANI) to the type genome, which has been assigned the MAG ID 1063 and which is available via NCBI BioSample SAMN15817231. This is a new name for the alphanumeric GTDB species sp900544575. The GC content of the type genome is 56.41% and the genome length is 1.5 Mbp.
**Description of *Candidatus* Limadaptatus stercoripullorum sp. nov.**
*Candidatus* Limadaptatus stercoripullorum (ster.co.ri.pul.lo’rum. L. neut. n. *stercus* dung; L. masc. n. *pullus* a young chicken; N.L. gen. n. *stercoripullorum* of the faceces of young chickens)
A bacterial species identified by metagenomic analyses. This species includes all bacteria with genomes that show ≥95% average nucleotide identity (ANI) to the type genome, which has been assigned the MAG ID 10406 and which is available via NCBI BioSample SAMN15817154. The GC content of the type genome is 61.19% and the genome length is 1.4 Mbp.

**Table:** 

**Description of *Candidatus* Limenecus gen. nov.**
*Candidatus* Limenecus (Lim.en.e’cus. L. masc. n. *limus* dung; Gr. masc. *enoikos* inhabitant; N.L. masc. n. *Limenecus* a microbe associated with faeces)
A bacterial genus identified by metagenomic analyses. The genus includes all bacteria with genomes that show ≥60% average amino acid identity (AAI) to the type genome from the type species *Candidatus* Limenecus avicola. This is a name for the alphanumeric GTDB genus CAG-306. This genus has been assigned by GTDB-Tk v1.3.0 working on GTDB Release 05-RS95 (Chaumeil et al., 2019; Parks et al., 2020) to the order *Gastranaerophilales* and to the family *Gastranaerophilaceae*.
**Description of *Candidatus* Limenecus avicola sp. nov.**
*Candidatus* Limenecus avicola (a.vi’co.la. L. fem. n. *avis* bird; L. suff. *-cola* inhabitant of; N.L. n. *avicola* inhabitant of birds)
A bacterial species identified by metagenomic analyses. This species includes all bacteria with genomes that show ≥95% average nucleotide identity (ANI) to the type genome, which has been assigned the MAG ID CHK154-7741 and which is available via NCBI BioSample SAMN15817206. This is a new name for the alphanumeric GTDB species sp000980375. The GC content of the type genome is 36.63% and the genome length is 2.1 Mbp.
**Description of *Candidatus* Limicola gen. nov.**
*Candidatus* Limicola (Li.mi’co.la. L. masc. n. *limus* dung; L. suff. *-cola* inhabitant of; N.L. fem. n. *Limicola* a microbe associated with faeces)
A bacterial genus identified by metagenomic analyses. The genus includes all bacteria with genomes that show ≥60% average amino acid identity (AAI) to the type genome from the type species *Candidatus* Limicola stercorigallinarum. This is a name for the alphanumeric GTDB genus An2-A. This genus has been assigned by GTDB-Tk v1.3.0 working on GTDB Release 05-RS95 (Chaumeil et al., 2019; Parks et al., 2020) to the order *Coriobacteriales* and to the family *Coriobacteriaceae*.
**Description of *Candidatus* Limicola stercorigallinarum sp. nov.**
*Candidatus* Limicola stercorigallinarum (ster.co.ri.gal.li.na’rum. L. neut. n. *stercus* dung; L. fem. n. *gallina* hen; N.L. gen. n. *stercorigallinarum* of hen faeces)
A bacterial species identified by metagenomic analyses. This species includes all bacteria with genomes that show ≥95% average nucleotide identity (ANI) to the type genome, which has been assigned the MAG ID ChiGjej1B1-16188 and which is available via NCBI BioSample SAMN15817082. The GC content of the type genome is 60.48% and the genome length is 2.0 Mbp.
**Description of *Candidatus* Limihabitans gen. nov.**
*Candidatus* Limihabitans (Li.mi.ha’bi.tans. L. masc. n. *limus* dung; L. pres. part. *habitans* an inhabitant; N.L. fem. n. *Limihabitans* a microbe associated with faeces)
A bacterial genus identified by metagenomic analyses. The genus includes all bacteria with genomes that show ≥60% average amino acid identity (AAI) to the type genome from the type species *Candidatus* Limihabitans stercoravium. This is a name for the alphanumeric GTDB genus UMGS1707. This genus has been assigned by GTDB-Tk v1.3.0 working on GTDB Release 05-RS95 (Chaumeil et al., 2019; Parks et al., 2020) to the order *4C28d-15* and to the family *CAG-314*.
**Description of *Candidatus* Limihabitans stercoravium sp. nov.**
*Candidatus* Limihabitans stercoravium (ster.cor.a’vi.um. L. neut. n. *stercus* dung; L. fem. n. *avis* bird; N.L. gen. n. *stercoravium* of bird faeces)
A bacterial species identified by metagenomic analyses. This species includes all bacteria with genomes that show ≥95% average nucleotide identity (ANI) to the type genome, which has been assigned the MAG ID 3394 and which is available via NCBI BioSample SAMN15817227. This is a new name for the alphanumeric GTDB species sp900547645. The GC content of the type genome is 43.23% and the genome length is 1.6 Mbp.
**Description of *Candidatus* Limimorpha gen. nov.**
*Candidatus* Limimorpha (Li.mi.mor’pha. L. masc. n. *limus* dung; Gr. fem. n. *morphe* a form, shape; N.L. fem. n. *Limimorpha* a microbe associated with faeces)
A bacterial genus identified by metagenomic analyses. The genus includes all bacteria with genomes that show ≥60% average amino acid identity (AAI) to the type genome from the type species *Candidatus* Limimorpha avicola. This is a name for the alphanumeric GTDB genus F082. This genus has been assigned by GTDB-Tk v1.3.0 working on GTDB Release 05-RS95 (Chaumeil et al., 2019; Parks et al., 2020) to the order *Bacteroidales* and to the family *F082*.
**Description of *Candidatus* Limimorpha avicola sp. nov.**
*Candidatus* Limimorpha avicola (a.vi’co.la. L. fem. n. *avis* bird; L. suff. *-cola* inhabitant of; N.L. n. *avicola* inhabitant of birds)
A bacterial species identified by metagenomic analyses. This species includes all bacteria with genomes that show ≥95% average nucleotide identity (ANI) to the type genome, which has been assigned the MAG ID Gambia15-481 and which is available via NCBI BioSample SAMN15817194. This is a new name for the alphanumeric GTDB species sp002633315. The GC content of the type genome is 38.09% and the genome length is 2.5 Mbp.

**Table:** 

**Description of *Candidatus* Limiplasma gen. nov.**
*Candidatus* Limiplasma (Li.mi.plas’ma. L. masc. n. *limus* dung; Gr. neut. n. *plasma* a form; N.L. neut. n. *Limiplasma* a microbe associated with faeces)
A bacterial genus identified by metagenomic analyses. The genus includes all bacteria with genomes that show ≥60% average amino acid identity (AAI) to the type genome from the type species *Candidatus* Limiplasma pullicola. This is a name for the alphanumeric GTDB genus Firm-11. This genus has been assigned by GTDB-Tk v1.3.0 working on GTDB Release 05-RS95 (Chaumeil et al., 2019; Parks et al., 2020) to the order *Christensenellales* and to the family *CAG-74*.
**Description of *Candidatus* Limiplasma merdipullorum sp. nov.**
*Candidatus* Limiplasma merdipullorum (mer.di.pul.lo’rum. L. fem. n. *merda* faeces; L. masc. n. *pullus* a young chicken; N.L. gen. n. *merdipullorum* of the faeces of young chickens)
A bacterial species identified by metagenomic analyses. This species includes all bacteria with genomes that show ≥95% average nucleotide identity (ANI) to the type genome, which has been assigned the MAG ID ChiBcec16-3123 and which is available via NCBI BioSample SAMN15817217. This is a new name for the alphanumeric GTDB species sp900540045. The GC content of the type genome is 61.78% and the genome length is 2.6 Mbp.
**Description of *Candidatus* Limiplasma pullicola sp. nov.**
*Candidatus* Limiplasma pullicola (pul.li’co.la. L. masc. n. *pullus* a young chicken; L. suff. *-cola* inhabitant of; N.L. n. *pullicola* an inhabitant of young chickens)
A bacterial species identified by metagenomic analyses. This species includes all bacteria with genomes that show ≥95% average nucleotide identity (ANI) to the type genome, which has been assigned the MAG ID 2223 and which is available via NCBI BioSample SAMN15817092. The GC content of the type genome is 62.99% and the genome length is 2.7 Mbp.
**Description of *Candidatus* Limiplasma pullistercoris sp. nov.**
*Candidatus* Limiplasma pullistercoris (pul.li.ster’co.ris. L. masc. n. *pullus* a young chicken; L. neut. n. *stercus* dung; N.L. gen. n. *pullistercoris* of young chicken faeces)
A bacterial species identified by metagenomic analyses. This species includes all bacteria with genomes that show ≥95% average nucleotide identity (ANI) to the type genome, which has been assigned the MAG ID ChiBcec15-2748 and which is available via NCBI BioSample SAMN15817228. This is a new name for the alphanumeric GTDB species sp900553905. The GC content of the type genome is 62.75% and the genome length is 2.8 Mbp.
**Description of *Candidatus* Limiplasma stercoravium sp. nov.**
*Candidatus* Limiplasma stercoravium (ster.cor.a’vi.um. L. neut. n. *stercus* dung; L. fem. n. *avis* bird; N.L. gen. n. *stercoravium* of bird faeces)
A bacterial species identified by metagenomic analyses. This species includes all bacteria with genomes that show ≥95% average nucleotide identity (ANI) to the type genome, which has been assigned the MAG ID CHK169-20388 and which is available via NCBI BioSample SAMN15817129. The GC content of the type genome is 62.11% and the genome length is 2.4 Mbp.
**Description of *Candidatus* Limisoma gen. nov.**
*Candidatus* Limisoma (Li.mi.so’ma. L. masc. n. *limus* dung; Gr. neut. n. *soma* a body; N.L. neut. n. *Limisoma* a microbe associated with faeces)
A bacterial genus identified by metagenomic analyses. The genus includes all bacteria with genomes that show ≥60% average amino acid identity (AAI) to the type genome from the type species *Candidatus* Limisoma faecipullorum. This is a name for the alphanumeric GTDB genus CAG-279. This genus has been assigned by GTDB-Tk v1.3.0 working on GTDB Release 05-RS95 (Chaumeil et al., 2019; Parks et al., 2020) to the order *Bacteroidales* and to the family *Muribaculaceae*.
**Description of *Candidatus* Limisoma faecipullorum sp. nov.**
*Candidatus* Limisoma faecipullorum (fae.ci.pul.lo’rum. L. fem. n. *faex, faecis* excrement; L. masc. n. *pullus* a young chicken; N.L. gen. n. *faecipullorum* of young chicken faeces)
A bacterial species identified by metagenomic analyses. This species includes all bacteria with genomes that show ≥95% average nucleotide identity (ANI) to the type genome, which has been assigned the MAG ID 6919 and which is available via NCBI BioSample SAMN15817069. The GC content of the type genome is 45.95% and the genome length is 2.2 Mbp.
**Description of *Candidatus* Limisoma gallistercoris sp. nov.**
*Candidatus* Limisoma gallistercoris (gal.li.ster’co.ris. L. masc. n *gallus* chicken; L. neut. n. *stercus* dung; N.L. gen. n. *gallistercoris* of chicken faeces)
A bacterial species identified by metagenomic analyses. This species includes all bacteria with genomes that show ≥95% average nucleotide identity (ANI) to the type genome, which has been assigned the MAG ID CHK136-1475 and which is available via NCBI BioSample SAMN15817174. This is a new name for the alphanumeric GTDB species sp900550025. The GC content of the type genome is 48.31% and the genome length is 2.2 Mbp.

**Table:** 

**Description of *Candidatus* Limisoma intestinavium sp. nov.**
*Candidatus* Limisoma intestinavium (in.tes.tin.a’vi.um. L. neut. n. *intestinum* gut; L. fem. n. *avis* bird; N.L. gen. n. *intestinavium* of the gut of birds)
A bacterial species identified by metagenomic analyses. This species includes all bacteria with genomes that show ≥95% average nucleotide identity (ANI) to the type genome, which has been assigned the MAG ID 17073 and which is available via NCBI BioSample SAMN15817199. This is a new name for the alphanumeric GTDB species sp900541555. The GC content of the type genome is 48.25% and the genome length is 1.8 Mbp.
**Description of *Candidatus* Limivicinus gen. nov.**
*Candidatus* Limivicinus (Li.mi.vi.ci’nus. L. masc. n. *limus* dung; L. masc. n. *vicinus* a neighbour; N.L. masc. n. *Limivicinus* a microbe associated with faeces)
A bacterial genus identified by metagenomic analyses. The genus includes all bacteria with genomes that show ≥60% average amino acid identity (AAI) to the type genome from the type species *Candidatus* Limivicinus faecipullorum. This is a name for the alphanumeric GTDB genus UBA1777. This genus has been assigned by GTDB-Tk v1.3.0 working on GTDB Release 05-RS95 (Chaumeil et al., 2019; Parks et al., 2020) to the order *Oscillospirales* and to the family *Oscillospiraceae*.
**Description of *Candidatus* Limivicinus faecipullorum sp. nov.**
*Candidatus* Limivicinus faecipullorum (fae.ci.pul.lo’rum. L. fem. n. *faex, faecis* excrement; L. masc. n. *pullus* a young chicken; N.L. gen. n. *faecipullorum* of young chicken faeces)
A bacterial species identified by metagenomic analyses. This species includes all bacteria with genomes that show ≥95% average nucleotide identity (ANI) to the type genome, which has been assigned the MAG ID ChiHcec3-8852 and which is available via NCBI BioSample SAMN15817081. The GC content of the type genome is 58.52% and the genome length is 1.9 Mbp.
**Description of *Candidatus* Limivivens gen. nov.**
*Candidatus* Limivivens (Li.mi.vi’vens. L. masc. n. *limus* dung; N.L. pres. part. *vivens* living; N.L. fem. n. *Limivivens* a microbe associated with faeces)
A bacterial genus identified by metagenomic analyses. The genus includes all bacteria with genomes that show ≥60% average amino acid identity (AAI) to the type genome from the type species *Candidatus* Limivivens intestinipullorum. This is a name for the alphanumeric GTDB genus GCA-900066135. This genus has been assigned by GTDB-Tk v1.3.0 working on GTDB Release 05-RS95 (Chaumeil et al., 2019; Parks et al., 2020) to the order *Lachnospirales* and to the family *Lachnospiraceae*.
**Description of *Candidatus* Limivivens intestinipullorum sp. nov.**
*Candidatus* Limivivens intestinipullorum (in.tes.ti.ni.pul.lo’rum. L. neut. n. *intestinum* gut; L. masc. n. *pullus* a young chicken; N.L. gen. n. *intestinipullorum* of the gut of young chickens)
A bacterial species identified by metagenomic analyses. This species includes all bacteria with genomes that show ≥95% average nucleotide identity (ANI) to the type genome, which has been assigned the MAG ID CHK190-19873 and which is available via NCBI BioSample SAMN15817025. The GC content of the type genome is 52.58% and the genome length is 3.4 Mbp.
**Description of *Candidatus* Limivivens merdigallinarum sp. nov.**
*Candidatus* Limivivens merdigallinarum (mer.di.gal.li.na’rum. L. fem. n. *merda* faeces; L. fem. n. *gallina* hen; N.L. gen. n. *merdigallinarum* of hen faeces)
A bacterial species identified by metagenomic analyses. This species includes all bacteria with genomes that show ≥95% average nucleotide identity (ANI) to the type genome, which has been assigned the MAG ID ChiSjej3B21-11622 and which is available via NCBI BioSample SAMN15817007. The GC content of the type genome is 50.09% and the genome length is 3.3 Mbp.
**Description of *Candidatus* Limosilactobacillus excrementigallinarum sp. nov.**
*Candidatus* Limosilactobacillus excrementigallinarum (ex.cre.men.ti.gal.li.na’rum. L. neut. n. *excrementum* excrement; L. fem. n. *gallina* hen; N.L. gen. n. *excrementigallinarum* of hen excrement)
A bacterial species identified by metagenomic analyses. This species includes all bacteria with genomes that show ≥95% average nucleotide identity (ANI) to the type genome, which has been assigned the MAG ID 2685 and which is available via NCBI BioSample SAMN15816672. The GC content of the type genome is 41.67% and the genome length is 1.4 Mbp.
**Description of *Candidatus* Limosilactobacillus faecipullorum sp. nov.**
*Candidatus* Limosilactobacillus faecipullorum (fae.ci.pul.lo’rum. L. fem. n. *faex, faecis* excrement; L. masc. n. *pullus* a young chicken; N.L. gen. n. *faecipullorum* of young chicken faeces)
A bacterial species identified by metagenomic analyses. This species includes all bacteria with genomes that show ≥95% average nucleotide identity (ANI) to the type genome, which has been assigned the MAG ID 7774 and which is available via NCBI BioSample SAMN15816663. The GC content of the type genome is 43.03% and the genome length is 1.5 Mbp.

**Table:** 

**Description of *Candidatus* Limosilactobacillus gallistercoris sp. nov.**
*Candidatus* Limosilactobacillus gallistercoris (gal.li.ster’co.ris. L. masc. n *gallus* chicken; L. neut. n. *stercus* dung; N.L. gen. n. *gallistercoris* of chicken faeces)
A bacterial species identified by metagenomic analyses. This species includes all bacteria with genomes that show ≥95% average nucleotide identity (ANI) to the type genome, which has been assigned the MAG ID CHK158-2993 and which is available via NCBI BioSample SAMN15816598. The GC content of the type genome is 52.74% and the genome length is 1.2 Mbp.
**Description of *Candidatus* Limosilactobacillus intestinavium sp. nov.**
*Candidatus* Limosilactobacillus intestinavium (in.tes.tin.a’vi.um. L. neut. n. *intestinum* gut; L. fem. n. *avis* bird; N.L. gen. n. *intestinavium* of the gut of birds)
A bacterial species identified by metagenomic analyses. This species includes all bacteria with genomes that show ≥95% average nucleotide identity (ANI) to the type genome, which has been assigned the MAG ID 2331 and which is available via NCBI BioSample SAMN15816838. This is a new name for the alphanumeric GTDB species sp900557215. The GC content of the type genome is 38.77% and the genome length is 1.5 Mbp.
**Description of *Candidatus* Limosilactobacillus intestinigallinarum sp. nov.**
*Candidatus* Limosilactobacillus intestinigallinarum (in.tes.ti.ni.gal.li.na’rum. L. neut. n. *intestinum* gut; L. fem. n. *gallina* hen; N.L. gen. n. *intestinigallinarum* of the gut of the hens)
A bacterial species identified by metagenomic analyses. This species includes all bacteria with genomes that show ≥95% average nucleotide identity (ANI) to the type genome, which has been assigned the MAG ID CHK176-5070 and which is available via NCBI BioSample SAMN15816600. The GC content of the type genome is 54.91% and the genome length is 1.5 Mbp.
**Description of *Candidatus* Limosilactobacillus intestinipullorum sp. nov.**
*Candidatus* Limosilactobacillus intestinipullorum (in.tes.ti.ni.pul.lo’rum. L. neut. n. *intestinum* gut; L. masc. n. *pullus* a young chicken; N.L. gen. n. *intestinipullorum* of the gut of young chickens)
A bacterial species identified by metagenomic analyses. This species includes all bacteria with genomes that show ≥95% average nucleotide identity (ANI) to the type genome, which has been assigned the MAG ID ChiHecolR2B26-165 and which is available via NCBI BioSample SAMN15816601. The GC content of the type genome is 49.12% and the genome length is 1.6 Mbp.
**Description of *Candidatus* Limosilactobacillus merdavium sp. nov.**
*Candidatus* Limosilactobacillus merdavium (merd.a’vi.um. L. fem. n. *merda* faeces; L. fem. n. *avis* bird; N.L. gen. n. *merdavium* of bird faeces)
A bacterial species identified by metagenomic analyses. This species includes all bacteria with genomes that show ≥95% average nucleotide identity (ANI) to the type genome, which has been assigned the MAG ID 876 and which is available via NCBI BioSample SAMN15816723. The GC content of the type genome is 39.60% and the genome length is 1.4 Mbp.
**Description of *Candidatus* Limosilactobacillus merdigallinarum sp. nov.**
*Candidatus* Limosilactobacillus merdigallinarum (mer.di.gal.li.na’rum. L. fem. n. *merda* faeces; L. fem. n. *gallina* hen; N.L. gen. n. *merdigallinarum* of hen faeces)
A bacterial species identified by metagenomic analyses. This species includes all bacteria with genomes that show ≥95% average nucleotide identity (ANI) to the type genome, which has been assigned the MAG ID ChiSxjej3B15-572 and which is available via NCBI BioSample SAMN15816736. The GC content of the type genome is 44.36% and the genome length is 1.4 Mbp.
**Description of *Candidatus* Limosilactobacillus merdipullorum sp. nov.**
*Candidatus* Limosilactobacillus merdipullorum (mer.di.pul.lo’rum. L. fem. n. *merda* faeces; L. masc. n. *pullus* a young chicken; N.L. gen. n. *merdipullorum* of the faeces of young chickens)
A bacterial species identified by metagenomic analyses. This species includes all bacteria with genomes that show ≥95% average nucleotide identity (ANI) to the type genome, which has been assigned the MAG ID ChiHejej3B27-2180 and which is available via NCBI BioSample SAMN15816756. The GC content of the type genome is 49.90% and the genome length is 1.3 Mbp.
**Description of *Candidatus* Limousia gen. nov.**
*Candidatus* Limousia (Lim.ou’si.a. L. masc. n. *limus* dung; Gr. fem. n. *ousia* an essence; N.L. fem. n. *Limousia* a microbe associated with faeces)
A bacterial genus identified by metagenomic analyses. The genus includes all bacteria with genomes that show ≥60% average amino acid identity (AAI) to the type genome from the type species *Candidatus* Limousia pullorum. This is a name for the alphanumeric GTDB genus An172. This genus has been assigned by GTDB-Tk v1.3.0 working on GTDB Release 05-RS95 (Chaumeil et al., 2019; Parks et al., 2020) to the order *Oscillospirales* and to the family *Acutalibacteraceae*.

**Table:** 

**Description of *Candidatus* Limousia pullorum sp. nov.**
*Candidatus* Limousia pullorum (pul.lo’rum. L. gen. pl. n. *pullorum* of young chickens)
A bacterial species identified by metagenomic analyses. This species includes all bacteria with genomes that show ≥95% average nucleotide identity (ANI) to the type genome, which has been assigned the MAG ID ChiGjej1B1-1684 and which is available via NCBI BioSample SAMN15817202. This is a new name for the alphanumeric GTDB species sp002160515. The GC content of the type genome is 40.91% and the genome length is 1.7 Mbp.
**Description of *Candidatus* Luteimonas excrementigallinarum sp. nov.**
*Candidatus* Luteimonas excrementigallinarum (ex.cre.men.ti.gal.li.na’rum. L. neut. n. *excrementum* excrement; L. fem. n. *gallina* hen; N.L. gen. n. *excrementigallinarum* of hen excrement)
A bacterial species identified by metagenomic analyses. This species includes all bacteria with genomes that show ≥95% average nucleotide identity (ANI) to the type genome, which has been assigned the MAG ID CHK165-14161 and which is available via NCBI BioSample SAMN15816707. The GC content of the type genome is 68.39% and the genome length is 2.5 Mbp.
**Description of *Candidatus* Luteococcus avicola sp. nov.**
*Candidatus* Luteococcus avicola (a.vi’co.la. L. fem. n. *avis* bird; L. suff. *-cola* inhabitant of; N.L. n. *avicola* inhabitant of birds)
A bacterial species identified by metagenomic analyses. This species includes all bacteria with genomes that show ≥95% average nucleotide identity (ANI) to the type genome, which has been assigned the MAG ID 4979 and which is available via NCBI BioSample SAMN15816867. This is a new name for the alphanumeric GTDB species sp002387005. The GC content of the type genome is 68.14% and the genome length is 2.9 Mbp.
**Description of *Candidatus* Mailhella excrementigallinarum sp. nov.**
*Candidatus* Mailhella excrementigallinarum (ex.cre.men.ti.gal.li.na’rum. L. neut. n. *excrementum* excrement; L. fem. n. *gallina* hen; N.L. gen. n. *excrementigallinarum* of hen excrement)
A bacterial species identified by metagenomic analyses. This species includes all bacteria with genomes that show ≥95% average nucleotide identity (ANI) to the type genome, which has been assigned the MAG ID 708 and which is available via NCBI BioSample SAMN15816871. This is a new name for the alphanumeric GTDB species sp003150275. The GC content of the type genome is 60.07% and the genome length is 3.0 Mbp.
**Description of *Candidatus* Mailhella merdavium sp. nov.**
*Candidatus* Mailhella merdavium (merd.a’vi.um. L. fem. n. *merda* faeces; L. fem. n. *avis* bird; N.L. gen. n. *merdavium* of bird faeces)
A bacterial species identified by metagenomic analyses. This species includes all bacteria with genomes that show ≥95% average nucleotide identity (ANI) to the type genome, which has been assigned the MAG ID ChiBcec6-11642 and which is available via NCBI BioSample SAMN15816648. The GC content of the type genome is 56.49% and the genome length is 2.7 Mbp.
**Description of *Candidatus* Mailhella merdigallinarum sp. nov.**
*Candidatus* Mailhella merdigallinarum (mer.di.gal.li.na’rum. L. fem. n. *merda* faeces; L. fem. n. *gallina* hen; N.L. gen. n. *merdigallinarum* of hen faeces)
A bacterial species identified by metagenomic analyses. This species includes all bacteria with genomes that show ≥95% average nucleotide identity (ANI) to the type genome, which has been assigned the MAG ID CHK186-16707 and which is available via NCBI BioSample SAMN15816842. This is a new name for the alphanumeric GTDB species sp900541395. The GC content of the type genome is 61.96% and the genome length is 2.4 Mbp.
**Description of *Candidatus* Massiliomicrobiota merdigallinarum sp. nov.**
*Candidatus* Massiliomicrobiota merdigallinarum (mer.di.gal.li.na’rum. L. fem. n. *merda* faeces; L. fem. n. *gallina* hen; N.L. gen. n. *merdigallinarum* of hen faeces)
A bacterial species identified by metagenomic analyses. This species includes all bacteria with genomes that show ≥95% average nucleotide identity (ANI) to the type genome, which has been assigned the MAG ID CHK183-8118 and which is available via NCBI BioSample SAMN15816832. This is a new name for the alphanumeric GTDB species sp002160815. The GC content of the type genome is 31.33% and the genome length is 2.4 Mbp.
**Description of *Candidatus* Mediterraneibacter avicola sp. nov.**
*Candidatus* Mediterraneibacter avicola (a.vi’co.la. L. fem. n. *avis* bird; L. suff. *-cola* inhabitant of; N.L. n. *avicola* inhabitant of birds)
A bacterial species identified by metagenomic analyses. This species includes all bacteria with genomes that show ≥95% average nucleotide identity (ANI) to the type genome, which has been assigned the MAG ID ChiGjej3B3-8055 and which is available via NCBI BioSample SAMN15816612. The GC content of the type genome is 48.65% and the genome length is 2.4 Mbp.

**Table:** 

**Description of *Candidatus* Mediterraneibacter caccavium sp. nov.**
*Candidatus* Mediterraneibacter caccavium (cacc.a’vi.um. Gr. fem. n. *kakke* faeces; L. fem. n. *avis* bird; N.L. gen. n. *caccavium* of bird faeces)
A bacterial species identified by metagenomic analyses. This species includes all bacteria with genomes that show ≥95% average nucleotide identity (ANI) to the type genome, which has been assigned the MAG ID ChiSjej5B23-15282 and which is available via NCBI BioSample SAMN15816861. This is a new name for the alphanumeric GTDB species sp002161355. The GC content of the type genome is 51.40% and the genome length is 2.6 Mbp.
**Description of *Candidatus* Mediterraneibacter caccogallinarum sp. nov.**
*Candidatus* Mediterraneibacter caccogallinarum (cac.co.gal.li.na’rum. Gr. fem. n. *kakke* faeces; L. fem. n. *gallina* hen; N.L. gen. n. *caccogallinarum* of hen faeces)
A bacterial species identified by metagenomic analyses. This species includes all bacteria with genomes that show ≥95% average nucleotide identity (ANI) to the type genome, which has been assigned the MAG ID ChiHcolR18-251 and which is available via NCBI BioSample SAMN15816801. This is a new name for the alphanumeric GTDB species sp002314255. The GC content of the type genome is 50.75% and the genome length is 2.6 Mbp.
**Description of *Candidatus* Mediterraneibacter colneyensis sp. nov.**
*Candidatus* Mediterraneibacter colneyensis (col.ney.en’sis. N.L. fem. adj. *colneyensis* pertaining to Colney, the Norfolk village which is home to the Quadram Institute where the species was first described)
A bacterial species identified by metagenomic analyses. This species includes all bacteria with genomes that show ≥95% average nucleotide identity (ANI) to the type genome, which has been assigned the MAG ID ChiGjej5B5-19924 and which is available via NCBI BioSample SAMN15816732. The GC content of the type genome is 50.96% and the genome length is 1.9 Mbp.
**Description of *Candidatus* Mediterraneibacter cottocaccae sp. nov.**
*Candidatus* Mediterraneibacter cottocaccae (cot.to.cac’cae. Gr. masc. n. *kottos* chicken Gr. fem. n. *kakke* faeces; N.L. gen. n. *cottocaccae* of chicken faeces)
A bacterial species identified by metagenomic analyses. This species includes all bacteria with genomes that show ≥95% average nucleotide identity (ANI) to the type genome, which has been assigned the MAG ID CHK192-87 and which is available via NCBI BioSample SAMN15816835. This is a new name for the alphanumeric GTDB species sp002160525. The GC content of the type genome is 50.07% and the genome length is 4.0 Mbp.
**Description of *Candidatus* Mediterraneibacter excrementavium sp. nov.**
*Candidatus* Mediterraneibacter excrementavium (ex.cre.ment.a’vi.um. L. neut. n. *excrementum* excrement; L. fem. n. *avis* bird; N.L. gen. n. *excrementavium* of bird excrement)
A bacterial species identified by metagenomic analyses. This species includes all bacteria with genomes that show ≥95% average nucleotide identity (ANI) to the type genome, which has been assigned the MAG ID ChiGjej2B2-38138 and which is available via NCBI BioSample SAMN15816630. The GC content of the type genome is 51.42% and the genome length is 2.1 Mbp.
**Description of *Candidatus* Mediterraneibacter excrementigallinarum sp. nov.**
*Candidatus* Mediterraneibacter excrementigallinarum (ex.cre.men.ti.gal.li.na’rum. L. neut. n. *excrementum* excrement; L. fem. n. *gallina* hen; N.L. gen. n. *excrementigallinarum* of hen excrement)
A bacterial species identified by metagenomic analyses. This species includes all bacteria with genomes that show ≥95% average nucleotide identity (ANI) to the type genome, which has been assigned the MAG ID CHK143-6153 and which is available via NCBI BioSample SAMN15816575. The GC content of the type genome is 48.97% and the genome length is 3.1 Mbp.
**Description of *Candidatus* Mediterraneibacter excrementipullorum sp. nov.**
*Candidatus* Mediterraneibacter excrementipullorum (ex.cre.men.ti.pul.lo’rum. L. neut. n. *excrementum* excrement; L. masc. n. *pullus* a young chicken; N.L. gen. n. *excrementipullorum* of young chicken excrement)
A bacterial species identified by metagenomic analyses. This species includes all bacteria with genomes that show ≥95% average nucleotide identity (ANI) to the type genome, which has been assigned the MAG ID ChiSjej6B24-3024 and which is available via NCBI BioSample SAMN15816810. This is a new name for the alphanumeric GTDB species sp9005552. The GC content of the type genome is 48.88% and the genome length is 2.4 Mbp.
**Description of *Candidatus* Mediterraneibacter faecavium sp. nov.**
*Candidatus* Mediterraneibacter faecavium (faec.a’vi.um. L. fem. n. *faex, faecis* excrement; L. fem. n. *avis* bird; N.L. gen. n. *faecavium* of bird faeces)
A bacterial species identified by metagenomic analyses. This species includes all bacteria with genomes that show ≥95% average nucleotide identity (ANI) to the type genome, which has been assigned the MAG ID CHK196-7946 and which is available via NCBI BioSample SAMN15816577. The GC content of the type genome is 49.45% and the genome length is 2.8 Mbp.

**Table:** 

**Description of *Candidatus* Mediterraneibacter faecigallinarum sp. nov.**
*Candidatus* Mediterraneibacter faecigallinarum (fae.ci.gal.li.na’rum. L. fem. n. *faex, faecis* excrement; L. fem. n. *gallina* hen; N.L. gen. n. *faecigallinarum* of hen faeces)
A bacterial species identified by metagenomic analyses. This species includes all bacteria with genomes that show ≥95% average nucleotide identity (ANI) to the type genome, which has been assigned the MAG ID ChiGjej1B1-1692 and which is available via NCBI BioSample SAMN15816637. The GC content of the type genome is 51.63% and the genome length is 2.6 Mbp.
**Description of *Candidatus* Mediterraneibacter faecipullorum sp. nov.**
*Candidatus* Mediterraneibacter faecipullorum (fae.ci.pul.lo’rum. L. fem. n. *faex, faecis* excrement; L. masc. n. *pullus* a young chicken; N.L. gen. n. *faecipullorum* of young chicken faeces)
A bacterial species identified by metagenomic analyses. This species includes all bacteria with genomes that show ≥95% average nucleotide identity (ANI) to the type genome, which has been assigned the MAG ID ChiW19-954 and which is available via NCBI BioSample SAMN15816638. The GC content of the type genome is 47.71% and the genome length is 2.7 Mbp.
**Description of *Candidatus* Mediterraneibacter gallistercoris sp. nov.**
*Candidatus* Mediterraneibacter gallistercoris (gal.li.ster’co.ris. L. masc. n. *gallus* chicken; L. neut. n. *stercus* dung; N.L. gen. n. *gallistercoris* of chicken faeces)
A bacterial species identified by metagenomic analyses. This species includes all bacteria with genomes that show ≥95% average nucleotide identity (ANI) to the type genome, which has been assigned the MAG ID CHK165-2605 and which is available via NCBI BioSample SAMN15816636. The GC content of the type genome is 47.20% and the genome length is 2.5 Mbp.
**Description of *Candidatus* Mediterraneibacter guildfordensis sp. nov.**
*Candidatus* Mediterraneibacter guildfordensis (guild.ford.en’sis. N.L. masc. adj. *guildfordensis* pertaining to Guildford, English town that is home to the University of Surrey)
A bacterial species identified by metagenomic analyses. This species includes all bacteria with genomes that show ≥95% average nucleotide identity (ANI) to the type genome, which has been assigned the MAG ID ChiHcec3-18395 and which is available via NCBI BioSample SAMN15816784. The GC content of the type genome is 52.47% and the genome length is 2.2 Mbp.
**Description of *Candidatus* Mediterraneibacter intestinavium sp. nov.**
*Candidatus* Mediterraneibacter intestinavium (in.tes.tin.a’vi.um. L. neut. n. *intestinum* gut; L. fem. n. *avis* bird; N.L. gen. n. *intestinavium* of the gut of birds)
A bacterial species identified by metagenomic analyses. This species includes all bacteria with genomes that show ≥95% average nucleotide identity (ANI) to the type genome, which has been assigned the MAG ID ChiBcec12-2655 and which is available via NCBI BioSample SAMN15816591. The GC content of the type genome is 50.53% and the genome length is 2.9 Mbp.
**Description of *Candidatus* Mediterraneibacter intestinigallinarum sp. nov.**
*Candidatus* Mediterraneibacter intestinigallinarum (in.tes.ti.ni.gal.li.na’rum. L. neut. n. *intestinum* gut; L. fem. n. *gallina* hen; N.L. gen. n. *intestinigallinarum* of the gut of the hens)
A bacterial species identified by metagenomic analyses. This species includes all bacteria with genomes that show ≥95% average nucleotide identity (ANI) to the type genome, which has been assigned the MAG ID ChiBcec15-2237 and which is available via NCBI BioSample SAMN15816645. The GC content of the type genome is 46.81% and the genome length is 3.1 Mbp.
**Description of *Candidatus* Mediterraneibacter intestinipullorum sp. nov.**
*Candidatus* Mediterraneibacter intestinipullorum (in.tes.ti.ni.pul.lo’rum. L. neut. n. *intestinum* gut; L. masc. n. *pullus* a young chicken; N.L. gen. n. *intestinipullorum* of the gut of young chickens)
A bacterial species identified by metagenomic analyses. This species includes all bacteria with genomes that show ≥95% average nucleotide identity (ANI) to the type genome, which has been assigned the MAG ID CHK161-4361 and which is available via NCBI BioSample SAMN15816656. The GC content of the type genome is 49.75% and the genome length is 2.5 Mbp.
**Description of *Candidatus* Mediterraneibacter merdavium sp. nov.**
*Candidatus* Mediterraneibacter merdavium (merd.a’vi.um. L. fem. n. *merda* faeces; L. fem. n. *avis* bird; N.L. gen. n. *merdavium* of bird faeces)
A bacterial species identified by metagenomic analyses. This species includes all bacteria with genomes that show ≥95% average nucleotide identity (ANI) to the type genome, which has been assigned the MAG ID ChiBcolR7-8672 and which is available via NCBI BioSample SAMN15816660. The GC content of the type genome is 49.88% and the genome length is 2.6 Mbp.

**Table:** 

**Description of *Candidatus* Mediterraneibacter merdigallinarum sp. nov.**
*Candidatus* Mediterraneibacter merdigallinarum (mer.di.gal.li.na’rum. L. fem. n. *merda* faeces; L. fem. n. *gallina* hen; N.L. gen. n. *merdigallinarum* of hen faeces)
A bacterial species identified by metagenomic analyses. This species includes all bacteria with genomes that show ≥95% average nucleotide identity (ANI) to the type genome, which has been assigned the MAG ID ChiW16-1363 and which is available via NCBI BioSample SAMN15816674. The GC content of the type genome is 46.95% and the genome length is 2.4 Mbp.
**Description of *Candidatus* Mediterraneibacter merdipullorum sp. nov.**
*Candidatus* Mediterraneibacter merdipullorum (mer.di.pul.lo’rum. L. fem. n. *merda* faeces; L. masc. n. *pullus* a young chicken; N.L. gen. n. *merdipullorum* of the faeces of young chickens)
A bacterial species identified by metagenomic analyses. This species includes all bacteria with genomes that show ≥95% average nucleotide identity (ANI) to the type genome, which has been assigned the MAG ID ChiSxjej4B16-6421 and which is available via NCBI BioSample SAMN15816679. The GC content of the type genome is 54.17% and the genome length is 2.2 Mbp.
**Description of *Candidatus* Mediterraneibacter norfolkensis sp. nov.**
*Candidatus* Mediterraneibacter norfolkensis (nor.folk.en’sis. N.L. masc. adj. *norfolkensis* pertaining to the English county of Norfolk)
A bacterial species identified by metagenomic analyses. This species includes all bacteria with genomes that show ≥95% average nucleotide identity (ANI) to the type genome, which has been assigned the MAG ID ChiW9-3490 and which is available via NCBI BioSample SAMN15816789. The GC content of the type genome is 48.85% and the genome length is 3.5 Mbp.
**Description of *Candidatus* Mediterraneibacter norwichensis sp. nov.**
*Candidatus* Mediterraneibacter norwichensis (nor.wich.en’sis. N.L. masc. adj. *norwichensis* pertaining to English city of Norwich)
A bacterial species identified by metagenomic analyses. This species includes all bacteria with genomes that show ≥95% average nucleotide identity (ANI) to the type genome, which has been assigned the MAG ID CHK180-4461 and which is available via NCBI BioSample SAMN15816628. The GC content of the type genome is 47.61% and the genome length is 2.6 Mbp.
**Description of *Candidatus* Mediterraneibacter ornithocaccae sp. nov.**
*Candidatus* Mediterraneibacter ornithocaccae (or.ni.tho.cac’cae. Gr. masc. or fem. n. *ornis, ornithos* bird Gr. fem. n. *kakke* faeces; N.L. gen. n. *ornithocaccae* of bird faeces)
A bacterial species identified by metagenomic analyses. This species includes all bacteria with genomes that show ≥95% average nucleotide identity (ANI) to the type genome, which has been assigned the MAG ID ChiGjej1B1-20579 and which is available via NCBI BioSample SAMN15816839. This is a new name for the alphanumeric GTDB species sp002159505. The GC content of the type genome is 47.31% and the genome length is 2.6 Mbp.
**Description of *Candidatus* Mediterraneibacter pullicola sp. nov.**
*Candidatus* Mediterraneibacter pullicola (pul.li’co.la. L. masc. n. *pullus* a young chicken; L. suff. *-cola* inhabitant of; N.L. n. *pullicola* an inhabitant of young chickens)
A bacterial species identified by metagenomic analyses. This species includes all bacteria with genomes that show ≥95% average nucleotide identity (ANI) to the type genome, which has been assigned the MAG ID ChiSjej2B20-11307 and which is available via NCBI BioSample SAMN15816678. The GC content of the type genome is 47.35% and the genome length is 2.1 Mbp.
**Description of *Candidatus* Mediterraneibacter pullistercoris sp. nov.**
*Candidatus* Mediterraneibacter pullistercoris (pul.li.ster’co.ris. L. masc. n. *pullus* a young chicken; L. neut. n. *stercus* dung; N.L. gen. n. *pullistercoris* of young chicken faeces)
A bacterial species identified by metagenomic analyses. This species includes all bacteria with genomes that show ≥95% average nucleotide identity (ANI) to the type genome, which has been assigned the MAG ID ChiHjej8B7-7219 and which is available via NCBI BioSample SAMN15816602. The GC content of the type genome is 48.73% and the genome length is 2.3 Mbp.
**Description of *Candidatus* Mediterraneibacter quadrami sp. nov.**
*Candidatus* Mediterraneibacter quadrami (quad.ra’mi. N.L. gen. n. *quadrami* of the Quadram Institute)
A bacterial species identified by metagenomic analyses. This species includes all bacteria with genomes that show ≥95% average nucleotide identity (ANI) to the type genome, which has been assigned the MAG ID ChiBcec15-3976 and which is available via NCBI BioSample SAMN15816790. The GC content of the type genome is 52.68% and the genome length is 2.0 Mbp.

**Table:** 

**Description of *Candidatus* Mediterraneibacter stercoravium sp. nov.**
*Candidatus* Mediterraneibacter stercoravium (ster.cor.a’vi.um. L. neut. n. *stercus* dung; L. fem. n. *avis* bird; N.L. gen. n. *stercoravium* of bird faeces)
A bacterial species identified by metagenomic analyses. This species includes all bacteria with genomes that show ≥95% average nucleotide identity (ANI) to the type genome, which has been assigned the MAG ID CHK196-3914 and which is available via NCBI BioSample SAMN15816603. The GC content of the type genome is 48.72% and the genome length is 2.5 Mbp.
**Description of *Candidatus* Mediterraneibacter stercorigallinarum sp. nov.**
*Candidatus* Mediterraneibacter stercorigallinarum (ster.co.ri.gal.li.na’rum. L. neut. n. *stercus* dung; L. fem. n. *gallina* hen; N.L. gen. n. *stercorigallinarum* of hen faeces)
A bacterial species identified by metagenomic analyses. This species includes all bacteria with genomes that show ≥95% average nucleotide identity (ANI) to the type genome, which has been assigned the MAG ID ChiGjej1B1-13045 and which is available via NCBI BioSample SAMN15816697. The GC content of the type genome is 50.04% and the genome length is 2.3 Mbp.
**Description of *Candidatus* Mediterraneibacter stercoripullorum sp. nov.**
*Candidatus* Mediterraneibacter stercoripullorum (ster.co.ri.pul.lo’rum. L. neut. n. *stercus* dung; L. masc. n. *pullus* a young chicken; N.L. gen. n. *stercoripullorum* of the faceces of young chickens)
A bacterial species identified by metagenomic analyses. This species includes all bacteria with genomes that show ≥95% average nucleotide identity (ANI) to the type genome, which has been assigned the MAG ID CHK195-1396 and which is available via NCBI BioSample SAMN15816610. The GC content of the type genome is 48.12% and the genome length is 3.2 Mbp.
**Description of *Candidatus* Mediterraneibacter surreyensis sp. nov.**
*Candidatus* Mediterraneibacter surreyensis (sur.rey.en’sis. N.L. masc. adj. *surreyensis* pertaining to the English county of Surrey where the samples in the study were collected)
A bacterial species identified by metagenomic analyses. This species includes all bacteria with genomes that show ≥95% average nucleotide identity (ANI) to the type genome, which has been assigned the MAG ID CHK177-12742 and which is available via NCBI BioSample SAMN15816593. The GC content of the type genome is 46.80% and the genome length is 2.9 Mbp.
**Description of *Candidatus* Mediterraneibacter tabaqchaliae sp. nov.**
*Candidatus* Mediterraneibacter tabaqchaliae (ta.baq.cha’li.ae. N.L. fem. gen. n. *tabaqchaliae* named in honour of British microbiologist Soad Tabaqchali)
A bacterial species identified by metagenomic analyses. This species includes all bacteria with genomes that show ≥95% average nucleotide identity (ANI) to the type genome, which has been assigned the MAG ID ChiGjej3B3-11674 and which is available via NCBI BioSample SAMN15816791. The GC content of the type genome is 51.91% and the genome length is 2.7 Mbp.
**Description of *Candidatus* Mediterraneibacter vanvlietii sp. nov.**
*Candidatus* Mediterraneibacter vanvlietii (van.vliet’i.i. N.L. gen. n. *vanvlietii* named in honour of Dutch microbiologist Arnoud van Vliet)
A bacterial species identified by metagenomic analyses. This species includes all bacteria with genomes that show ≥95% average nucleotide identity (ANI) to the type genome, which has been assigned the MAG ID ChiBcec1-362 and which is available via NCBI BioSample SAMN15816623. The GC content of the type genome is 48.47% and the genome length is 3.0 Mbp.
**Description of *Candidatus* Megamonas gallistercoris sp. nov.**
*Candidatus* Megamonas gallistercoris (gal.li.ster’co.ris. L. masc. n. *gallus* chicken; L. neut. n. *stercus* dung; N.L. gen. n. *gallistercoris* of chicken faeces)
A bacterial species identified by metagenomic analyses. This species includes all bacteria with genomes that show ≥95% average nucleotide identity (ANI) to the type genome, which has been assigned the MAG ID ChiGjej6B6-7947 and which is available via NCBI BioSample SAMN15816859. This is a new name for the alphanumeric GTDB species sp900554895. The GC content of the type genome is 40.34% and the genome length is 2.2 Mbp.
**Description of *Candidatus* Merdenecus gen. nov.**
*Candidatus* Merdenecus (Merd.en.e’cus. L. fem. n. *merda* dung; Gr. masc. *enoikos* inhabitant; N.L. masc. n. *Merdenecus* a microbe associated with faeces)
A bacterial genus identified by metagenomic analyses. The genus includes all bacteria with genomes that show ≥60% average amino acid identity (AAI) to the type genome from the type species *Candidatus* Merdenecus merdavium. This is a name for the alphanumeric GTDB genus MCWD5. This genus has been assigned by GTDB-Tk v1.3.0 working on GTDB Release 05-RS95 (Chaumeil et al., 2019; Parks et al., 2020) to the order *Lachnospirales* and to the family *Lachnospiraceae*.

**Table:** 

**Description of *Candidatus* Merdenecus pullicola sp. nov.**
*Candidatus* Merdenecus pullicola (pul.li’co.la. L. masc. n. *pullus* a young chicken; L. suff. *-cola* inhabitant of; N.L. n. *pullicola* an inhabitant of young chickens)
A bacterial species identified by metagenomic analyses. This species includes all bacteria with genomes that show ≥95% average nucleotide identity (ANI) to the type genome, which has been assigned the MAG ID CHK160-2840 and which is available via NCBI BioSample SAMN15817122. The GC content of the type genome is 35.46% and the genome length is 2.6 Mbp.
**Description of *Candidatus* Merdibacter merdavium sp. nov.**
*Candidatus* Merdibacter merdavium (merd.a’vi.um. L. fem. n. *merda* faeces; L. fem. n. *avis* bird; N.L. gen. n. *merdavium* of bird faeces)
A bacterial species identified by metagenomic analyses. This species includes all bacteria with genomes that show ≥95% average nucleotide identity (ANI) to the type genome, which has been assigned the MAG ID CHK187-11901 and which is available via NCBI BioSample SAMN15816582. The GC content of the type genome is 53.13% and the genome length is 2.1 Mbp.
**Description of *Candidatus* Merdibacter merdigallinarum sp. nov.**
*Candidatus* Merdibacter merdigallinarum (mer.di.gal.li.na’rum. L. fem. n. *merda* faeces; L. fem. n. *gallina* hen; N.L. gen. n. *merdigallinarum* of hen faeces)
A bacterial species identified by metagenomic analyses. This species includes all bacteria with genomes that show ≥95% average nucleotide identity (ANI) to the type genome, which has been assigned the MAG ID ChiGjej6B6-453 and which is available via NCBI BioSample SAMN15816595. The GC content of the type genome is 53.92% and the genome length is 1.8 Mbp.
**Description of *Candidatus* Merdibacter merdipullorum sp. nov.**
*Candidatus* Merdibacter merdipullorum (mer.di.pul.lo’rum. L. fem. n. *merda* faeces; L. masc. n. *pullus* a young chicken; N.L. gen. n. *merdipullorum* of the faeces of young chickens)
A bacterial species identified by metagenomic analyses. This species includes all bacteria with genomes that show ≥95% average nucleotide identity (ANI) to the type genome, which has been assigned the MAG ID ChiGjej1B1-19782 and which is available via NCBI BioSample SAMN15816850. This is a new name for the alphanumeric GTDB species sp900543035. The GC content of the type genome is 55.41% and the genome length is 1.9 Mbp.
**Description of *Candidatus* Merdicola gen. nov.**
*Candidatus* Merdicola (Mer.di’co.la. L. fem. n. *merda* dung; L. suff. *-cola* inhabitant of; N.L. fem. n. *Merdicola* a microbe associated with faeces)
A bacterial genus identified by metagenomic analyses. The genus includes all bacteria with genomes that show ≥60% average amino acid identity (AAI) to the type genome from the type species *Candidatus* Merdicola faecigallinarum. This is a name for the alphanumeric GTDB genus CAG-354. This genus has been assigned by GTDB-Tk v1.3.0 working on GTDB Release 05-RS95 (Chaumeil et al., 2019; Parks et al., 2020) to the order *TANB77* and to the family *CAG-508*.
**Description of *Candidatus* Merdicola faecigallinarum sp. nov.**
*Candidatus* Merdicola faecigallinarum (fae.ci.gal.li.na’rum. L. fem. n. *faex, faecis* excrement; L. fem. n. *gallina* hen; N.L. gen. n. *faecigallinarum* of hen faeces)
A bacterial species identified by metagenomic analyses. This species includes all bacteria with genomes that show ≥95% average nucleotide identity (ANI) to the type genome, which has been assigned the MAG ID CHK195-15760 and which is available via NCBI BioSample SAMN15817051. The GC content of the type genome is 28.83% and the genome length is 1.5 Mbp.
**Description of *Candidatus* Merdimorpha gen. nov.**
*Candidatus* Merdimorpha (Mer.di.mor’pha. L. fem. n. *merda* dung; Gr. fem. n. *morphe* a form, shape; N.L. fem. n. *Merdimorpha* a microbe associated with faeces)
A bacterial genus identified by metagenomic analyses. The genus includes all bacteria with genomes that show ≥60% average amino acid identity (AAI) to the type genome from the type species *Candidatus* Merdimorpha intestinavium. This is a name for the alphanumeric GTDB genus UBA1820. This genus has been assigned by GTDB-Tk v1.3.0 working on GTDB Release 05-RS95 (Chaumeil et al., 2019; Parks et al., 2020) to the order *Flavobacteriales* and to the family *UBA1820*.
**Description of *Candidatus* Merdimorpha intestinavium sp. nov.**
*Candidatus* Merdimorpha intestinavium (in.tes.tin.a’vi.um. L. neut. n. *intestinum* gut; L. fem. n. *avis* bird; N.L. gen. n. *intestinavium* of the gut of birds)
A bacterial species identified by metagenomic analyses. This species includes all bacteria with genomes that show ≥95% average nucleotide identity (ANI) to the type genome, which has been assigned the MAG ID CHK1-7158 and which is available via NCBI BioSample SAMN15817210. This is a new name for the alphanumeric GTDB species sp002314265. The GC content of the type genome is 56.50% and the genome length is 1.8 Mbp.

**Table:** 

**Description of *Candidatus* Merdimorpha stercoravium sp. nov.**
*Candidatus* Merdimorpha stercoravium (ster.cor.a’vi.um. L. neut. n. *stercus* dung; L. fem. n. *avis* bird; N.L. gen. n. *stercoravium* of bird faeces)
A bacterial species identified by metagenomic analyses. This species includes all bacteria with genomes that show ≥95% average nucleotide identity (ANI) to the type genome, which has been assigned the MAG ID 1383 and which is available via NCBI BioSample SAMN15817125. The GC content of the type genome is 57.82% and the genome length is 1.7 Mbp.
**Description of *Candidatus* Merdiplasma gen. nov.**
*Candidatus* Merdiplasma (Mer.di.plas’ma. L. fem. n. *merda* dung; Gr. neut. n. *plasma* a form; N.L. neut. n. *Merdiplasma* a microbe associated with faeces)
A bacterial genus identified by metagenomic analyses. The genus includes all bacteria with genomes that show ≥60% average amino acid identity (AAI) to the type genome from the type species *Candidatus* Merdiplasma excrementigallinarum. This is a name for the alphanumeric GTDB genus UBA2856. This genus has been assigned by GTDB-Tk v1.3.0 working on GTDB Release 05-RS95 (Chaumeil et al., 2019; Parks et al., 2020) to the order *Lachnospirales* and to the family *Lachnospiraceae*.
**Description of *Candidatus* Merdiplasma excrementigallinarum sp. nov.**
*Candidatus* Merdiplasma excrementigallinarum (ex.cre.men.ti.gal.li.na’rum. L. neut. n. *excrementum* excrement; L. fem. n. *gallina* hen; N.L. gen. n. *excrementigallinarum* of hen excrement)
A bacterial species identified by metagenomic analyses. This species includes all bacteria with genomes that show ≥95% average nucleotide identity (ANI) to the type genome, which has been assigned the MAG ID ChiBcec6-7307 and which is available via NCBI BioSample SAMN15817161. The GC content of the type genome is 52.97% and the genome length is 2.4 Mbp.
**Description of *Candidatus* Merdisoma gen. nov.**
*Candidatus* Merdisoma (Mer.di.so’ma. L. fem. n. *merda* dung; Gr. neut. n. *soma* a body; N.L. neut. n. *Merdisoma* a microbe associated with faeces)
A bacterial genus identified by metagenomic analyses. The genus includes all bacteria with genomes that show ≥60% average amino acid identity (AAI) to the type genome from the type species *Candidatus* Merdisoma merdipullorum. This is a name for the alphanumeric GTDB genus GCA-900066575. This genus has been assigned by GTDB-Tk v1.3.0 working on GTDB Release 05-RS95 (Chaumeil et al., 2019; Parks et al., 2020) to the order *Lachnospirales* and to the family *Lachnospiraceae*.
**Description of *Candidatus* Merdisoma faecale sp. nov.**
*Candidatus* Merdisoma faecalis (fae.ca’le. L. neut. adj. *faecale* of faeces)
A bacterial species identified by metagenomic analyses. This species includes all bacteria with genomes that show ≥95% average nucleotide identity (ANI) to the type genome, which has been assigned the MAG ID ChiBcolR2-1241 and which is available via NCBI BioSample SAMN15817219. This is a new name for the alphanumeric GTDB species sp002160765. The GC content of the type genome is 51.56% and the genome length is 2.7 Mbp.
**Description of *Candidatus* Merdisoma merdipullorum sp. nov.**
*Candidatus* Merdisoma merdipullorum (mer.di.pul.lo’rum. L. fem. n. *merda* faeces; L. masc. n. *pullus* a young chicken; N.L. gen. n. *merdipullorum* of the faeces of young chickens)
A bacterial species identified by metagenomic analyses. This species includes all bacteria with genomes that show ≥95% average nucleotide identity (ANI) to the type genome, which has been assigned the MAG ID CHK197-19677 and which is available via NCBI BioSample SAMN15817042. The GC content of the type genome is 50.06% and the genome length is 2.9 Mbp.
**Description of *Candidatus* Merdivicinus gen. nov.**
*Candidatus* Merdivicinus (Mer.di.vi.ci’nus. L. fem. n. *merda* dung; L. masc. n. *vicinus* a neighbour; N.L. masc. n. *Merdivicinus* a microbe associated with faeces)
A bacterial genus identified by metagenomic analyses. The genus includes all bacteria with genomes that show ≥60% average amino acid identity (AAI) to the type genome from the type species *Candidatus* Merdivicinus faecavium. This is a name for the alphanumeric GTDB genus UMGS1826. This genus has been assigned by GTDB-Tk v1.3.0 working on GTDB Release 05-RS95 (Chaumeil et al., 2019; Parks et al., 2020) to the order *Oscillospirales* and to the family *Ruminococcaceae*.
**Description of *Candidatus* Merdivicinus excrementipullorum sp. nov.**
*Candidatus* Merdivicinus excrementipullorum (ex.cre.men.ti.pul.lo’rum. L. neut. n. *excrementum* excrement; L. masc. n. *pullus* a young chicken; N.L. gen. n. *excrementipullorum* of young chicken excrement)
A bacterial species identified by metagenomic analyses. This species includes all bacteria with genomes that show ≥95% average nucleotide identity (ANI) to the type genome, which has been assigned the MAG ID CHK199-13235 and which is available via NCBI BioSample SAMN15817032. The GC content of the type genome is 54.67% and the genome length is 2.5 Mbp.

**Table:** 

**Description of *Candidatus* Merdivicinus faecavium sp. nov.**
*Candidatus* Merdivicinus faecavium (faec.a’vi.um. L. fem. n. *faex, faecis* excrement; L. fem. n. *avis* bird; N.L. gen. n. *faecavium* of bird faeces)
A bacterial species identified by metagenomic analyses. This species includes all bacteria with genomes that show ≥95% average nucleotide identity (ANI) to the type genome, which has been assigned the MAG ID CHK186-19003 and which is available via NCBI BioSample SAMN15817036. The GC content of the type genome is 61.26% and the genome length is 2.5 Mbp.
**Description of *Candidatus* Merdivicinus intestinavium sp. nov.**
*Candidatus* Merdivicinus intestinavium (in.tes.tin.a’vi.um. L. neut. n. *intestinum* gut; L. fem. n. *avis* bird; N.L. gen. n. *intestinavium* of the gut of birds)
A bacterial species identified by metagenomic analyses. This species includes all bacteria with genomes that show ≥95% average nucleotide identity (ANI) to the type genome, which has been assigned the MAG ID CHK188-1901 and which is available via NCBI BioSample SAMN15817003. The GC content of the type genome is 59.41% and the genome length is 2.3 Mbp.
**Description of *Candidatus* Merdivicinus intestinigallinarum sp. nov.**
*Candidatus* Merdivicinus intestinigallinarum (in.tes.ti.ni.gal.li.na’rum. L. neut. n. *intestinum* gut; L. fem. n. *gallina* hen; N.L. gen. n. *intestinigallinarum* of the gut of the hens)
A bacterial species identified by metagenomic analyses. This species includes all bacteria with genomes that show ≥95% average nucleotide identity (ANI) to the type genome, which has been assigned the MAG ID ChiBcec18-2170 and which is available via NCBI BioSample SAMN15817163. The GC content of the type genome is 56.27% and the genome length is 2.5 Mbp.
**Description of *Candidatus* Merdivivens gen. nov.**
*Candidatus* Merdivivens (Mer.di.vi’vens. L. fem. n. *merda* dung; N.L. pres. part. *vivens* living; N.L. fem. n. *Merdivivens* a microbe associated with faeces)
A bacterial genus identified by metagenomic analyses. The genus includes all bacteria with genomes that show ≥60% average amino acid identity (AAI) to the type genome from the type species *Candidatus* Merdivivens pullistercoris. This is a name for the alphanumeric GTDB genus UBA3382. This genus has been assigned by GTDB-Tk v1.3.0 working on GTDB Release 05-RS95 (Chaumeil et al., 2019; Parks et al., 2020) to the order *Bacteroidales* and to the family *UBA932*.
**Description of *Candidatus* Merdivivens faecigallinarum sp. nov.**
*Candidatus* Merdivivens faecigallinarum (fae.ci.gal.li.na’rum. L. fem. n. *faex, faecis* excrement; L. fem. n. *gallina* hen; N.L. gen. n. *faecigallinarum* of hen faeces)
A bacterial species identified by metagenomic analyses. This species includes all bacteria with genomes that show ≥95% average nucleotide identity (ANI) to the type genome, which has been assigned the MAG ID B3-2255 and which is available via NCBI BioSample SAMN15817168. This is a new name for the alphanumeric GTDB species sp002159555. The GC content of the type genome is 49.87% and the genome length is 1.9 Mbp.
**Description of *Candidatus* Merdivivens pullicola sp. nov.**
*Candidatus* Merdivivens pullicola (pul.li’co.la. L. masc. n. *pullus* a young chicken; L. suff. *-cola* inhabitant of; N.L. n. *pullicola* an inhabitant of young chickens)
A bacterial species identified by metagenomic analyses. This species includes all bacteria with genomes that show ≥95% average nucleotide identity (ANI) to the type genome, which has been assigned the MAG ID B1-8020 and which is available via NCBI BioSample SAMN15817062. The GC content of the type genome is 48.22% and the genome length is 2.0 Mbp.
**Description of *Candidatus* Merdivivens pullistercoris sp. nov.**
*Candidatus* Merdivivens pullistercoris (pul.li.ster’co.ris. L. masc. n. *pullus* a young chicken; L. neut. n. *stercus* dung; N.L. gen. n. *pullistercoris* of young chicken faeces)
A bacterial species identified by metagenomic analyses. This species includes all bacteria with genomes that show ≥95% average nucleotide identity (ANI) to the type genome, which has been assigned the MAG ID 10037 and which is available via NCBI BioSample SAMN15817074. The GC content of the type genome is 48.95% and the genome length is 2.1 Mbp.
**Description of *Candidatus* Merdousia gen. nov.**
*Candidatus* Merdousia (Merd.ou’si.a. L. fem. n. *merda* dung; Gr. fem. n. *ousia* an essence; N.L. fem. n. *Merdousia* a microbe associated with faeces)
A bacterial genus identified by metagenomic analyses. The genus includes all bacteria with genomes that show ≥60% average amino acid identity (AAI) to the type genome from the type species *Candidatus* Merdousia gallistercoris. This is a name for the alphanumeric GTDB genus CAG-312. This genus has been assigned by GTDB-Tk v1.3.0 working on GTDB Release 05-RS95 (Chaumeil et al., 2019; Parks et al., 2020) to the order *Opitutales* and to the family *CAG-312*.

**Table:** 

**Description of *Candidatus* Merdousia gallistercoris sp. nov.**
*Candidatus* Merdousia gallistercoris (gal.li.ster’co.ris. L. masc. n *gallus* chicken; L. neut. n. *stercus* dung; N.L. gen. n. *gallistercoris* of chicken faeces)
A bacterial species identified by metagenomic analyses. This species includes all bacteria with genomes that show ≥95% average nucleotide identity (ANI) to the type genome, which has been assigned the MAG ID CHK197-16368 and which is available via NCBI BioSample SAMN15817207. This is a new name for the alphanumeric GTDB species sp900545715. The GC content of the type genome is 49.37% and the genome length is 2.4 Mbp.
**Description of *Candidatus* Methanocorpusculum faecipullorum sp. nov.**
*Candidatus* Methanocorpusculum faecipullorum (fae.ci.pul.lo’rum. L. fem. n. *faex, faecis* excrement; L. masc. n. *pullus* a young chicken; N.L. gen. n. *faecipullorum* of young chicken faeces)
An archaeal species identified by metagenomic analyses. This species includes all archaea with genomes that show ≥95% average nucleotide identity (ANI) to the type genome, which has been assigned the MAG ID E1-3281 and which is available via NCBI BioSample SAMN15816796. The GC content of the type genome is 50.72% and the genome length is 1.2 Mbp.
**Description of *Candidatus* Methanospyradousia gen. nov.**
*Candidatus* Methanospyradousia (Meth.an.o.spy.rad.ou’si.a. N.L. neut. n. *methanum*, methane; N.L. pref. *methano*-, pertaining to methane; Gr. fem. n. *spyras* ball of dung; Gr. fem. n. *ousia* an essence; N.L. fem. n. *Methanospyradousia* a methanogenic microbe associated with the intestines)
An archaeal genus identified by metagenomic analyses. The genus includes all archaea with genomes that show ≥60% average amino acid identity (AAI) to the type genome from the type species *Candidatus* Methanospyradousia avicola. This is a name for the alphanumeric GTDB genus UBA71. This genus has been assigned by GTDB-Tk v1.3.0 working on GTDB Release 05-RS95 (Chaumeil et al., 2019; Parks et al., 2020) to the order *Methanomassiliicoccales* and to the family *Methanomethylophilaceae*.
**Description of *Candidatus* Methanospyradousia avicola sp. nov.**
*Candidatus* Methanospyradousia avicola (a.vi’co.la. L. fem. n. *avis* bird; L. suff. *-cola* inhabitant of; N.L. n. *avicola* inhabitant of birds)
An archaeal species identified by metagenomic analyses. This species includes all archaea with genomes that show ≥95% average nucleotide identity (ANI) to the type genome, which has been assigned the MAG ID 6227 and which is available via NCBI BioSample SAMN15817164. The GC content of the type genome is 60.22% and the genome length is 1.5 Mbp.
**Description of *Candidatus* Microbacterium pullistercoris sp. nov.**
*Candidatus* Microbacterium pullistercoris (pul.li.ster’co.ris. L. masc. n. *pullus* a young chicken; L. neut. n. *stercus* dung; N.L. gen. n. *pullistercoris* of young chicken faeces)
A bacterial species identified by metagenomic analyses. This species includes all bacteria with genomes that show ≥95% average nucleotide identity (ANI) to the type genome, which has been assigned the MAG ID ChiGjej1B1-5908 and which is available via NCBI BioSample SAMN15816649. The GC content of the type genome is 68.74% and the genome length is 2.5 Mbp.
**Description of *Candidatus* Microbacterium stercoravium sp. nov.**
*Candidatus* Microbacterium stercoravium (ster.cor.a’vi.um. L. neut. n. *stercus* dung; L. fem. n. *avis* bird; N.L. gen. n. *stercoravium* of bird faeces)
A bacterial species identified by metagenomic analyses. This species includes all bacteria with genomes that show ≥95% average nucleotide identity (ANI) to the type genome, which has been assigned the MAG ID ChiHjej8B7-3636 and which is available via NCBI BioSample SAMN15816680. The GC content of the type genome is 69.44% and the genome length is 2.5 Mbp.
**Description of *Candidatus* Monoglobus merdigallinarum sp. nov.**
*Candidatus* Monoglobus merdigallinarum (mer.di.gal.li.na’rum. L. fem. n. *merda* faeces; L. fem. n. *gallina* hen; N.L. gen. n. *merdigallinarum* of hen faeces)
A bacterial species identified by metagenomic analyses. This species includes all bacteria with genomes that show ≥95% average nucleotide identity (ANI) to the type genome, which has been assigned the MAG ID 5790 and which is available via NCBI BioSample SAMN15816780. The GC content of the type genome is 48.24% and the genome length is 1.5 Mbp.
**Description of *Candidatus* Mucispirillum faecigallinarum sp. nov.**
*Candidatus* Mucispirillum faecigallinarum (fae.ci.gal.li.na’rum. L. fem. n. *faex, faecis* excrement; L. fem. n. *gallina* hen; N.L. gen. n. *faecigallinarum* of hen faeces)
A bacterial species identified by metagenomic analyses. This species includes all bacteria with genomes that show ≥95% average nucleotide identity (ANI) to the type genome, which has been assigned the MAG ID ChiW4-1371 and which is available via NCBI BioSample SAMN15816684. The GC content of the type genome is 31.75% and the genome length is 2.2 Mbp.

**Table:** 

**Description of *Candidatus* Negativibacillus faecipullorum sp. nov.**
*Candidatus* Negativibacillus faecipullorum (fae.ci.pul.lo’rum. L. fem. n. *faex, faecis* excrement; L. masc. n. *pullus* a young chicken; N.L. gen. n. *faecipullorum* of young chicken faeces)
A bacterial species identified by metagenomic analyses. This species includes all bacteria with genomes that show ≥95% average nucleotide identity (ANI) to the type genome, which has been assigned the MAG ID ChiBcec6-1156 and which is available via NCBI BioSample SAMN15816879. This is a new name for the alphanumeric GTDB species sp900547455. The GC content of the type genome is 57.54% and the genome length is 2.0 Mbp.
**Description of *Candidatus* Nesterenkonia stercoripullorum sp. nov.**
*Candidatus* Nesterenkonia stercoripullorum (ster.co.ri.pul.lo’rum. L. neut. n. *stercus* dung; L. masc. n. *pullus* a young chicken; N.L. gen. n. *stercoripullorum* of the faceces of young chickens)
A bacterial species identified by metagenomic analyses. This species includes all bacteria with genomes that show ≥95% average nucleotide identity (ANI) to the type genome, which has been assigned the MAG ID ChiHejej3B27-3195 and which is available via NCBI BioSample SAMN15816751. The GC content of the type genome is 65.88% and the genome length is 2.6 Mbp.
**Description of *Candidatus* Niameybacter stercoravium sp. nov.**
*Candidatus* Niameybacter stercoravium (ster.cor.a’vi.um. L. neut. n. *stercus* dung; L. fem. n. *avis* bird; N.L. gen. n. *stercoravium* of bird faeces)
A bacterial species identified by metagenomic analyses. This species includes all bacteria with genomes that show ≥95% average nucleotide identity (ANI) to the type genome, which has been assigned the MAG ID 2467 and which is available via NCBI BioSample SAMN15816773. The GC content of the type genome is 35.14% and the genome length is 2.9 Mbp.
**Description of *Candidatus* Nocardiopsis merdipullorum sp. nov.**
*Candidatus* Nocardiopsis merdipullorum (mer.di.pul.lo’rum. L. fem. n. *merda* faeces; L. masc. n. *pullus* a young chicken; N.L. gen. n. *merdipullorum* of the faeces of young chickens)
A bacterial species identified by metagenomic analyses. This species includes all bacteria with genomes that show ≥95% average nucleotide identity (ANI) to the type genome, which has been assigned the MAG ID ChiHjej10B9-18110 and which is available via NCBI BioSample SAMN15816716. The GC content of the type genome is 65.81% and the genome length is 4.2 Mbp.
**Description of *Candidatus* Nosocomiicoccus stercorigallinarum sp. nov.**
*Candidatus* Nosocomiicoccus stercorigallinarum (ster.co.ri.gal.li.na’rum. L. neut. n. *stercus* dung; L. fem. n. *gallina* hen; N.L. gen. n. *stercorigallinarum* of hen faeces)
A bacterial species identified by metagenomic analyses. This species includes all bacteria with genomes that show ≥95% average nucleotide identity (ANI) to the type genome, which has been assigned the MAG ID CHK169-14505 and which is available via NCBI BioSample SAMN15816647. The GC content of the type genome is 34.64% and the genome length is 1.3 Mbp.
**Description of *Candidatus* Oceanisphaera merdipullorum sp. nov.**
*Candidatus* Oceanisphaera merdipullorum (mer.di.pul.lo’rum. L. fem. n. *merda* faeces; L. masc. n. *pullus* a young chicken; N.L. gen. n. *merdipullorum* of the faeces of young chickens)
A bacterial species identified by metagenomic analyses. This species includes all bacteria with genomes that show ≥95% average nucleotide identity (ANI) to the type genome, which has been assigned the MAG ID 819 and which is available via NCBI BioSample SAMN15816797. The GC content of the type genome is 50.19% and the genome length is 2.9 Mbp.
**Description of *Candidatus* Odoribacter faecigallinarum sp. nov.**
*Candidatus* Odoribacter faecigallinarum (fae.ci.gal.li.na’rum. L. fem. n. *faex, faecis* excrement; L. fem. n. *gallina* hen; N.L. gen. n. *faecigallinarum* of hen faeces)
A bacterial species identified by metagenomic analyses. This species includes all bacteria with genomes that show ≥95% average nucleotide identity (ANI) to the type genome, which has been assigned the MAG ID 23274 and which is available via NCBI BioSample SAMN15816743. The GC content of the type genome is 48.17% and the genome length is 2.2 Mbp.
**Description of *Candidatus* Olsenella avicola sp. nov.**
*Candidatus* Olsenella avicola (a.vi’co.la. L. fem. n. *avis* bird; L. suff. *-cola* inhabitant of; N.L. n. *avicola* inhabitant of birds)
A bacterial species identified by metagenomic analyses. This species includes all bacteria with genomes that show ≥95% average nucleotide identity (ANI) to the type genome, which has been assigned the MAG ID CHK1-7693 and which is available via NCBI BioSample SAMN15816923. This is a new name for the alphanumeric GTDB species sp002159625. Although GTDB has assigned this species to the genus it calls Olsenella_E, this genus designation cannot be incorporated into a well-formed binomial, so in naming this species, we have used the current validly published name for the genus. The GC content of the type genome is 67.59% and the genome length is 2.2 Mbp.

**Table:** 

**Description of *Candidatus* Olsenella avistercoris sp. nov.**
*Candidatus* Olsenella avistercoris (a.vi.ster’co.ris. L. fem. n. *avis* bird; L. neut. n. *stercus* dung; N.L. gen. n. *avistercoris* of bird faeces)
A bacterial species identified by metagenomic analyses. This species includes all bacteria with genomes that show ≥95% average nucleotide identity (ANI) to the type genome, which has been assigned the MAG ID CHK136-6238 and which is available via NCBI BioSample SAMN15816919. This is a new name for the alphanumeric GTDB species sp002160255. Although GTDB has assigned this species to the genus it calls Olsenella_E, this genus designation cannot be incorporated into a well-formed binomial, so in naming this species, we have used the current validly published name for the genus. The GC content of the type genome is 69.05% and the genome length is 2.0 Mbp.
**Description of *Candidatus* Olsenella excrementavium sp. nov.**
*Candidatus* Olsenella excrementavium (ex.cre.ment.a’vi.um. L. neut. n. *excrementum* excrement; L. fem. n. *avis* bird; N.L. gen. n. *excrementavium* of bird excrement)
A bacterial species identified by metagenomic analyses. This species includes all bacteria with genomes that show ≥95% average nucleotide identity (ANI) to the type genome, which has been assigned the MAG ID ChiHjej10B9-743 and which is available via NCBI BioSample SAMN15816922. This is a new name for the alphanumeric GTDB species sp002305805. Although GTDB has assigned this species to the genus it calls Olsenella_E, this genus designation cannot be incorporated into a well-formed binomial, so in naming this species, we have used the current validly published name for the genus. The GC content of the type genome is 66.50% and the genome length is 1.8 Mbp.
**Description of *Candidatus* Olsenella excrementigallinarum sp. nov.**
*Candidatus* Olsenella excrementigallinarum (ex.cre.men.ti.gal.li.na’rum. L. neut. n. *excrementum* excrement; L. fem. n. *gallina* hen; N.L. gen. n. *excrementigallinarum* of hen excrement)
A bacterial species identified by metagenomic analyses. This species includes all bacteria with genomes that show ≥95% average nucleotide identity (ANI) to the type genome, which has been assigned the MAG ID ChiHjej12B11-23512 and which is available via NCBI BioSample SAMN15816920. This is a new name for the alphanumeric GTDB species sp900119915. Although GTDB has assigned this species to the genus it calls Olsenella_E, this genus designation cannot be incorporated into a well-formed binomial, so in naming this species, we have used the current validly published name for the genus. The GC content of the type genome is 68.67% and the genome length is 1.8 Mbp.
**Description of *Candidatus* Olsenella pullicola sp. nov.**
*Candidatus* Olsenella pullicola (pul.li’co.la. L. masc. n. *pullus* a young chicken; L. suff. *-cola* inhabitant of; N.L. n. *pullicola* an inhabitant of young chickens)
A bacterial species identified by metagenomic analyses. This species includes all bacteria with genomes that show ≥95% average nucleotide identity (ANI) to the type genome, which has been assigned the MAG ID ChiHecec1B25-7792 and which is available via NCBI BioSample SAMN15816895. Although GTDB has assigned this species to the genus it calls Olsenella_E, this genus designation cannot be incorporated into a well-formed binomial, so in naming this species, we have used the current validly published name for the genus. The GC content of the type genome is 65.74% and the genome length is 2.3 Mbp.
**Description of *Candidatus* Olsenella pullistercoris sp. nov.**
*Candidatus* Olsenella pullistercoris (pul.li.ster’co.ris. L. masc. n. *pullus* a young chicken; L. neut. n. *stercus* dung; N.L. gen. n. *pullistercoris* of young chicken faeces)
A bacterial species identified by metagenomic analyses. This species includes all bacteria with genomes that show ≥95% average nucleotide identity (ANI) to the type genome, which has been assigned the MAG ID ChiHjej12B11-14209 and which is available via NCBI BioSample SAMN15816899. Although GTDB has assigned this species to the genus it calls Olsenella_E, this genus designation cannot be incorporated into a well-formed binomial, so in naming this species, we have used the current validly published name for the genus. The GC content of the type genome is 67.21% and the genome length is 1.9 Mbp.
**Description of *Candidatus* Olsenella stercoravium sp. nov.**
*Candidatus* Olsenella stercoravium (ster.cor.a’vi.um. L. neut. n. *stercus* dung; L. fem. n. *avis* bird; N.L. gen. n. *stercoravium* of bird faeces)
A bacterial species identified by metagenomic analyses. This species includes all bacteria with genomes that show ≥95% average nucleotide identity (ANI) to the type genome, which has been assigned the MAG ID ChiHecolR3B27-1887 and which is available via NCBI BioSample SAMN15816902. Although GTDB has assigned this species to the genus it calls Olsenella_E, this genus designation cannot be incorporated into a well-formed binomial, so in naming this species, we have used the current validly published name for the genus. The GC content of the type genome is 67.26% and the genome length is 1.8 Mbp.

**Table:** 

**Description of *Candidatus* Onthenecus gen. nov.**
*Candidatus* Onthenecus (Onth.en.e’cus. Gr. masc. n. *onthos* dung; Gr. masc. *enoikos* inhabitant; N.L. masc. n. *Onthenecus* a microbe associated with faeces)
A bacterial genus identified by metagenomic analyses. The genus includes all bacteria with genomes that show ≥60% average amino acid identity (AAI) to the type genome from the type species *Candidatus* Onthenecus intestinigallinarum. This is a name for the alphanumeric GTDB genus OEMS01. This genus has been assigned by GTDB-Tk v1.3.0 working on GTDB Release 05-RS95 (Chaumeil et al., 2019; Parks et al., 2020) to the order *Christensenellales* and to the family *CAG-74*.
**Description of *Candidatus* Onthenecus intestinigallinarum sp. nov.**
*Candidatus* Onthenecus intestinigallinarum (in.tes.ti.ni.gal.li.na’rum. L. neut. n. *intestinum* gut; L. fem. n. *gallina* hen; N.L. gen. n. *intestinigallinarum* of the gut of the hens)
A bacterial species identified by metagenomic analyses. This species includes all bacteria with genomes that show ≥95% average nucleotide identity (ANI) to the type genome, which has been assigned the MAG ID ChiSxjej2B14-6234 and which is available via NCBI BioSample SAMN15817054. The GC content of the type genome is 66.20% and the genome length is 2.4 Mbp.
**Description of *Candidatus* Onthocola gen. nov.**
*Candidatus* Onthocola (On.tho’co.la. Gr. masc. n. *onthos* dung; L. suff. *-cola* inhabitant of; N.L. fem. n. *Onthocola* a microbe associated with faeces)
A bacterial genus identified by metagenomic analyses. The genus includes all bacteria with genomes that show ≥60% average amino acid identity (AAI) to the type genome from the type species *Candidatus* Onthocola gallistercoris. This is a name for the alphanumeric GTDB genus. This genus has been assigned by GTDB-Tk v1.3.0 working on GTDB Release 05-RS95 (Chaumeil et al., 2019; Parks et al., 2020) to the order *Lachnospirales* and to the family *Lachnospiraceae*.
**Description of *Candidatus* Onthocola gallistercoris sp. nov.**
*Candidatus* Onthocola gallistercoris (gal.li.ster’co.ris. L. masc. n *gallus* chicken; L. neut. n. *stercus* dung; N.L. gen. n. *gallistercoris* of chicken faeces)
A bacterial species identified by metagenomic analyses. This species includes all bacteria with genomes that show ≥95% average nucleotide identity (ANI) to the type genome, which has been assigned the MAG ID CHK187-14744 and which is available via NCBI BioSample SAMN15817044. The GC content of the type genome is 48.32% and the genome length is 2.4 Mbp.
**Description of *Candidatus* Onthocola stercoravium sp. nov.**
*Candidatus* Onthocola stercoravium (ster.cor.a’vi.um. L. neut. n. *stercus* dung; L. fem. n. *avis* bird; N.L. gen. n. *stercoravium* of bird faeces)
A bacterial species identified by metagenomic analyses. This species includes all bacteria with genomes that show ≥95% average nucleotide identity (ANI) to the type genome, which has been assigned the MAG ID ChiW5-5982 and which is available via NCBI BioSample SAMN15817021. The GC content of the type genome is 28.08% and the genome length is 1.4 Mbp.
**Description of *Candidatus* Onthocola stercorigallinarum sp. nov.**
*Candidatus* Onthocola stercorigallinarum (ster.co.ri.gal.li.na’rum. L. neut. n. *stercus* dung; L. fem. n. *gallina* hen; N.L. gen. n. *stercorigallinarum* of hen faeces)
A bacterial species identified by metagenomic analyses. This species includes all bacteria with genomes that show ≥95% average nucleotide identity (ANI) to the type genome, which has been assigned the MAG ID CHK195-3072 and which is available via NCBI BioSample SAMN15817046. The GC content of the type genome is 27.21% and the genome length is 1.3 Mbp.
**Description of *Candidatus* Onthomonas gen. nov.**
*Candidatus* Onthomonas (On.tho.mo’nas. Gr. masc. n. *onthos* dung; L. fem. n. *monas* a monad; N.L. fem. n. *Onthomonas* a microbe associated with faeces)
A bacterial genus identified by metagenomic analyses. The genus includes all bacteria with genomes that show ≥60% average amino acid identity (AAI) to the type genome from the type species *Candidatus* Onthomonas avicola. This is a name for the alphanumeric GTDB genus NK3B98. This genus has been assigned by GTDB-Tk v1.3.0 working on GTDB Release 05-RS95 (Chaumeil et al., 2019; Parks et al., 2020) to the order *Oscillospirales* and to the family *Oscillospiraceae*.
**Description of *Candidatus* Onthomonas avicola sp. nov.**
*Candidatus* Onthomonas avicola (a.vi’co.la. L. fem. n. *avis* bird; L. suff. *-cola* inhabitant of; N.L. n. *avicola* inhabitant of birds)
A bacterial species identified by metagenomic analyses. This species includes all bacteria with genomes that show ≥95% average nucleotide identity (ANI) to the type genome, which has been assigned the MAG ID ChiGjej6B6-14002 and which is available via NCBI BioSample SAMN15817096. The GC content of the type genome is 63.01% and the genome length is 2.4 Mbp.

**Table:** 

**Description of *Candidatus* Onthomorpha gen. nov.**
*Candidatus* Onthomorpha (On.tho.mor’pha. Gr. masc. n. *onthos* dung; Gr. fem. n. *morphe* a form, shape; N.L. fem. n. *Onthomorpha* a microbe associated with faeces)
A bacterial genus identified by metagenomic analyses. The genus includes all bacteria with genomes that show ≥60% average amino acid identity (AAI) to the type genome from the type species *Candidatus* Onthomorpha intestinigallinarum. This is a name for the alphanumeric GTDB genus UBA3388. This genus has been assigned by GTDB-Tk v1.3.0 working on GTDB Release 05-RS95 (Chaumeil et al., 2019; Parks et al., 2020) to the order *Bacteroidales* and to the family *P3*.
**Description of *Candidatus* Onthomorpha intestinigallinarum sp. nov.**
*Candidatus* Onthomorpha intestinigallinarum (in.tes.ti.ni.gal.li.na’rum. L. neut. n. *intestinum* gut; L. fem. n. *gallina* hen; N.L. gen. n. *intestinigallinarum* of the gut of the hens)
A bacterial species identified by metagenomic analyses. This species includes all bacteria with genomes that show ≥95% average nucleotide identity (ANI) to the type genome, which has been assigned the MAG ID Gambia16-930 and which is available via NCBI BioSample SAMN15817128. The GC content of the type genome is 42.76% and the genome length is 1.7 Mbp.
**Description of *Candidatus* Onthoplasma gen. nov.**
*Candidatus* Onthoplasma (On.tho.plas’ma. Gr. masc. n. *onthos* dung; Gr. neut. n. *plasma* a form; N.L. neut. n. *Onthoplasma* a microbe associated with faeces)
A bacterial genus identified by metagenomic analyses. The genus includes all bacteria with genomes that show ≥60% average amino acid identity (AAI) to the type genome from the type species *Candidatus* Onthoplasma faecipullorum. This is a name for the alphanumeric GTDB genus UBA4626. This genus has been assigned by GTDB-Tk v1.3.0 working on GTDB Release 05-RS95 (Chaumeil et al., 2019; Parks et al., 2020) to the order *4C28d-15* and to the family *UBA1242*.
**Description of *Candidatus* Onthoplasma faecigallinarum sp. nov.**
*Candidatus* Onthoplasma faecigallinarum (fae.ci.gal.li.na’rum. L. fem. n. *faex, faecis* excrement; L. fem. n. *gallina* hen; N.L. gen. n. *faecigallinarum* of hen faeces)
A bacterial species identified by metagenomic analyses. This species includes all bacteria with genomes that show ≥95% average nucleotide identity (ANI) to the type genome, which has been assigned the MAG ID 5992 and which is available via NCBI BioSample SAMN15817127. The GC content of the type genome is 34.71% and the genome length is 1.0 Mbp.
**Description of *Candidatus* Onthoplasma faecipullorum sp. nov.**
*Candidatus* Onthoplasma faecipullorum (fae.ci.pul.lo’rum. L. fem. n. *faex, faecis* excrement; L. masc. n. *pullus* a young chicken; N.L. gen. n. *faecipullorum* of young chicken faeces)
A bacterial species identified by metagenomic analyses. This species includes all bacteria with genomes that show ≥95% average nucleotide identity (ANI) to the type genome, which has been assigned the MAG ID CHK191-42317 and which is available via NCBI BioSample SAMN15817053. The GC content of the type genome is 31.88% and the genome length is 1.1 Mbp.
**Description of *Candidatus* Onthosoma gen. nov.**
*Candidatus* Onthosoma (On.tho.so’ma. Gr. masc. n. *onthos* dung; Gr. neut. n. *soma* a body; N.L. neut. n. *Onthosoma* a microbe associated with faeces)
A bacterial genus identified by metagenomic analyses. The genus includes all bacteria with genomes that show ≥60% average amino acid identity (AAI) to the type genome from the type species *Candidatus* Onthosoma merdavium. This is a name for the alphanumeric GTDB genus OEMR01. This genus has been assigned by GTDB-Tk v1.3.0 working on GTDB Release 05-RS95 (Chaumeil et al., 2019; Parks et al., 2020) to the order *Erysipelotrichales* and to the family *Erysipelotrichaceae*.
**Description of *Candidatus* Onthosoma merdavium sp. nov.**
*Candidatus* Onthosoma merdavium (merd.a’vi.um. L. fem. n. *merda* faeces; L. fem. n. *avis* bird; N.L. gen. n. *merdavium* of bird faeces)
A bacterial species identified by metagenomic analyses. This species includes all bacteria with genomes that show ≥95% average nucleotide identity (ANI) to the type genome, which has been assigned the MAG ID ChiBcec15-4520 and which is available via NCBI BioSample SAMN15817169. This is a new name for the alphanumeric GTDB species sp900199515. The GC content of the type genome is 45.30% and the genome length is 1.6 Mbp.
**Description of *Candidatus* Onthousia gen. nov.**
*Candidatus* Onthousia (Onth.ou’si.a. Gr. masc. n. *onthos* dung; Gr. fem. n. *ousia* an essence; N.L. fem. n. *Onthousia* a microbe associated with faeces)
A bacterial genus identified by metagenomic analyses. The genus includes all bacteria with genomes that show ≥60% average amino acid identity (AAI) to the type genome from the type species *Candidatus* Onthousia faecavium. This is a name for the alphanumeric GTDB genus CAG-451. This genus has been assigned by GTDB-Tk v1.3.0 working on GTDB Release 05-RS95 (Chaumeil et al., 2019; Parks et al., 2020) to the order *RF39* and to the family *CAG-611*.

**Table:** 

**Description of *Candidatus* Onthousia excrementipullorum sp. nov.**
*Candidatus* Onthousia excrementipullorum (ex.cre.men.ti.pul.lo’rum. L. neut. n. *excrementum* excrement; L. masc. n. *pullus* a young chicken; N.L. gen. n. *excrementipullorum* of young chicken excrement)
A bacterial species identified by metagenomic analyses. This species includes all bacteria with genomes that show ≥95% average nucleotide identity (ANI) to the type genome, which has been assigned the MAG ID CHK184-20233 and which is available via NCBI BioSample SAMN15817019. The GC content of the type genome is 27.72% and the genome length is 1.3 Mbp.
**Description of *Candidatus* Onthousia faecavium sp. nov.**
*Candidatus* Onthousia faecavium (faec.a’vi.um. L. fem. n. *faex, faecis* excrement; L. fem. n. *avis* bird; N.L. gen. n. *faecavium* of bird faeces)
A bacterial species identified by metagenomic analyses. This species includes all bacteria with genomes that show ≥95% average nucleotide identity (ANI) to the type genome, which has been assigned the MAG ID CHK195-6217 and which is available via NCBI BioSample SAMN15817026. The GC content of the type genome is 28.40% and the genome length is 1.3 Mbp.
**Description of *Candidatus* Onthousia faecigallinarum sp. nov.**
*Candidatus* Onthousia faecigallinarum (fae.ci.gal.li.na’rum. L. fem. n. *faex, faecis* excrement; L. fem. n. *gallina* hen; N.L. gen. n. *faecigallinarum* of hen faeces)
A bacterial species identified by metagenomic analyses. This species includes all bacteria with genomes that show ≥95% average nucleotide identity (ANI) to the type genome, which has been assigned the MAG ID CHK135-1819 and which is available via NCBI BioSample SAMN15817105. The GC content of the type genome is 32.62% and the genome length is 1.1 Mbp.
**Description of *Candidatus* Onthousia faecipullorum sp. nov.**
*Candidatus* Onthousia faecipullorum (fae.ci.pul.lo’rum. L. fem. n. *faex, faecis* excrement; L. masc. n. *pullus* a young chicken; N.L. gen. n. *faecipullorum* of young chicken faeces)
A bacterial species identified by metagenomic analyses. This species includes all bacteria with genomes that show ≥95% average nucleotide identity (ANI) to the type genome, which has been assigned the MAG ID CHK195-26880 and which is available via NCBI BioSample SAMN15817040. The GC content of the type genome is 27.41% and the genome length is 1.4 Mbp.
**Description of *Candidatus* Onthovicinus gen. nov.**
*Candidatus* Onthovicinus (On.tho.vi.ci’nus. Gr. masc. n. *onthos* dung; L. masc. n. *vicinus* a neighbour; N.L. masc. n. *Onthovicinus* a microbe associated with faeces)
A bacterial genus identified by metagenomic analyses. The genus includes all bacteria with genomes that show ≥60% average amino acid identity (AAI) to the type genome from the type species *Candidatus* Onthovicinus excrementipullorum. This is a name for the alphanumeric GTDB genus UMGS1839. This genus has been assigned by GTDB-Tk v1.3.0 working on GTDB Release 05-RS95 (Chaumeil et al., 2019; Parks et al., 2020) to the order *Oscillospirales* and to the family *Acutalibacteraceae*.
**Description of *Candidatus* Onthovicinus excrementipullorum sp. nov.**
*Candidatus* Onthovicinus excrementipullorum (ex.cre.men.ti.pul.lo’rum. L. neut. n. *excrementum* excrement; L. masc. n. *pullus* a young chicken; N.L. gen. n. *excrementipullorum* of young chicken excrement)
A bacterial species identified by metagenomic analyses. This species includes all bacteria with genomes that show ≥95% average nucleotide identity (ANI) to the type genome, which has been assigned the MAG ID CHK185-12131 and which is available via NCBI BioSample SAMN15817020. The GC content of the type genome is 55.55% and the genome length is 2.4 Mbp.
**Description of *Candidatus* Onthovivens gen. nov.**
*Candidatus* Onthovivens (On.tho.vi’vens. Gr. masc. n. *onthos* dung; N.L. pres. part. *vivens* living; N.L. fem. n. *Onthovivens* a microbe associated with faeces)
A bacterial genus identified by metagenomic analyses. The genus includes all bacteria with genomes that show ≥60% average amino acid identity (AAI) to the type genome from the type species *Candidatus* Onthovivens merdipullorum. This is a name for the alphanumeric GTDB genus UBA4855. This genus has been assigned by GTDB-Tk v1.3.0 working on GTDB Release 05-RS95 (Chaumeil et al., 2019; Parks et al., 2020) to the order *RFN20* and to the family *CAG-826*.
**Description of *Candidatus* Onthovivens merdipullorum sp. nov.**
*Candidatus* Onthovivens merdipullorum (mer.di.pul.lo’rum. L. fem. n. *merda* faeces; L. masc. n. *pullus* a young chicken; N.L. gen. n. *merdipullorum* of the faeces of young chickens)
A bacterial species identified by metagenomic analyses. This species includes all bacteria with genomes that show ≥95% average nucleotide identity (ANI) to the type genome, which has been assigned the MAG ID 11159 and which is available via NCBI BioSample SAMN15817139. The GC content of the type genome is 27.05% and the genome length is 1.5 Mbp.

**Table:** 

**Description of *Candidatus* Ornithocaccomicrobium gen. nov.**
*Candidatus* Ornithocaccomicrobium (Or.ni.tho.cac.co.mi.cro’bi.um. Gr. masc. or fem. n. *ornis, ornithos* bird; Gr. fem. n. *kakke* faeces; N.L. neut. n. *microbium* a microbe; N.L. neut. n. *Ornithocaccomicrobium* A microbe found in chicken faceces)
A bacterial genus identified by metagenomic analyses. The genus includes all bacteria with genomes that show ≥60% average amino acid identity (AAI) to the type genome from the type species *Candidatus* Ornithocaccomicrobium faecavium. This genus was identified but not named by Glendinning et al. (2020). This genus has been assigned by GTDB-Tk v1.3.0 working on GTDB Release 05-RS95 (Chaumeil et al., 2019; Parks et al., 2020) to the order *Christensenellales* and to the family *CAG-74*.
**Description of *Candidatus* Ornithocaccomicrobium faecavium sp. nov.**
*Candidatus* Ornithocaccomicrobium faecavium (faec.a’vi.um. L. fem. n. *faex, faecis* excrement; L. fem. n. *avis* bird; N.L. gen. n. *faecavium* of bird faeces)
A bacterial species identified by metagenomic analyses. This species includes all bacteria with genomes that show ≥95% average nucleotide identity (ANI) to the type genome, which has been assigned the MAG ID CHK183-6373 and which is available via NCBI BioSample SAMN15816945. The GC content of the type genome is 59.48% and the genome length is 2.9 Mbp.
**Description of *Candidatus* Ornithoclostridium gen. nov.**
*Candidatus* Ornithoclostridium (Or.ni.tho.clos.tri’di.um. Gr. masc. or fem. n. *ornis, ornithos* bird; N.L. neut. n. *Clostridium* a genus name; N.L. neut. n. *Ornithoclostridium* a genus related to the genus *Clostridium* but distinct from it and found in poultry)
A bacterial genus identified by metagenomic analyses. The genus includes all bacteria with genomes that show ≥60% average amino acid identity (AAI) to the type genome from the type species *Candidatus* Ornithoclostridium excrementipullorum. This genus has been assigned by GTDB-Tk v1.3.0 working on GTDB Release 05-RS95 (Chaumeil et al., 2019; Parks et al., 2020) to the order *Christensenellales* and to the family *UBA3700*.
**Description of *Candidatus* Ornithoclostridium excrementipullorum sp. nov.**
*Candidatus* Ornithoclostridium excrementipullorum (ex.cre.men.ti.pul.lo’rum. L. neut. n. *excrementum* excrement; L. masc. n. *pullus* a young chicken; N.L. gen. n. *excrementipullorum* of young chicken excrement)
A bacterial species identified by metagenomic analyses. This species includes all bacteria with genomes that show ≥95% average nucleotide identity (ANI) to the type genome, which has been assigned the MAG ID ChiW5-1639 and which is available via NCBI BioSample SAMN15816971. The GC content of the type genome is 54.62% and the genome length is 1.6 Mbp.
**Description of *Candidatus* Ornithoclostridium faecavium sp. nov.**
*Candidatus* Ornithoclostridium faecavium (faec.a’vi.um. L. fem. n. *faex, faecis* excrement; L. fem. n. *avis* bird; N.L. gen. n. *faecavium* of bird faeces)
A bacterial species identified by metagenomic analyses. This species includes all bacteria with genomes that show ≥95% average nucleotide identity (ANI) to the type genome, which has been assigned the MAG ID 63 and which is available via NCBI BioSample SAMN15816992. The GC content of the type genome is 48.09% and the genome length is 1.8 Mbp.
**Description of *Candidatus* Ornithoclostridium faecigallinarum sp. nov.**
*Candidatus* Ornithoclostridium faecigallinarum (fae.ci.gal.li.na’rum. L. fem. n. *faex, faecis* excrement; L. fem. n. *gallina* hen; N.L. gen. n. *faecigallinarum* of hen faeces)
A bacterial species identified by metagenomic analyses. This species includes all bacteria with genomes that show ≥95% average nucleotide identity (ANI) to the type genome, which has been assigned the MAG ID ChiHcolR4-3946 and which is available via NCBI BioSample SAMN15816996. The GC content of the type genome is 57.12% and the genome length is 1.6 Mbp.
**Description of *Candidatus* Ornithomonoglobus gen. nov.**
*Candidatus* Ornithomonoglobus (Or.ni.tho.mo.no.glo’bus. Gr. masc. or fem. n. *ornis, ornithos* bird; N.L. masc. n. *Monoglobus* a genus name; N.L. masc. n. *Ornithomonoglobus* a genus related to the genus *Monoglobus* but distinct from it and found in poultry)
A bacterial genus identified by metagenomic analyses. The genus includes all bacteria with genomes that show ≥60% average amino acid identity (AAI) to the type genome from the type species *Candidatus* Ornithomonoglobus merdipullorum. This genus was identified but not named by Glendinning et al. (2020). This genus has been assigned by GTDB-Tk v1.3.0 working on GTDB Release 05-RS95 (Chaumeil et al., 2019; Parks et al., 2020) to the order *Monoglobales* and to the family *UBA1381*.
**Description of *Candidatus* Ornithomonoglobus intestinigallinarum sp. nov.**
*Candidatus* Ornithomonoglobus intestinigallinarum (in.tes.ti.ni.gal.li.na’rum. L. neut. n. *intestinum* gut; L. fem. n. *gallina* hen; N.L. gen. n. *intestinigallinarum* of the gut of the hens)
A bacterial species identified by metagenomic analyses. This species includes all bacteria with genomes that show ≥95% average nucleotide identity (ANI) to the type genome, which has been assigned the MAG ID CHK181-108 and which is available via NCBI BioSample SAMN15816941. The GC content of the type genome is 49.36% and the genome length is 2.2 Mbp.

**Table:** 

**Description of *Candidatus* Ornithomonoglobus merdipullorum sp. nov.**
*Candidatus* Ornithomonoglobus merdipullorum (mer.di.pul.lo’rum. L. fem. n. *merda* faeces; L. masc. n. *pullus* a young chicken; N.L. gen. n. *merdipullorum* of the faeces of young chickens)
A bacterial species identified by metagenomic analyses. This species includes all bacteria with genomes that show ≥95% average nucleotide identity (ANI) to the type genome, which has been assigned the MAG ID USAMLcec3-3695 and which is available via NCBI BioSample SAMN15816942. The GC content of the type genome is 48.52% and the genome length is 2.7 Mbp.
**Description of *Candidatus* Ornithospirochaeta gen. nov.**
*Candidatus* Ornithospirochaeta (Or.ni.tho.spi.ro.chae’ta. Gr. masc. or fem. n. *ornis, ornithos* bird; N.L. fem. n. *Spirochaeta* a genus name; N.L. fem. n. *Ornithospirochaeta* a genus related to the genus *Spirochaeta* but distinct from it and found in poultry)
A bacterial genus identified by metagenomic analyses. The genus includes all bacteria with genomes that show ≥60% average amino acid identity (AAI) to the type genome from the type species *Candidatus* Ornithospirochaeta stercoravium. This genus has been assigned by GTDB-Tk v1.3.0 working on GTDB Release 05-RS95 (Chaumeil et al., 2019; Parks et al., 2020) to the order *Sphaerochaetales* and to the family *Sphaerochaetaceae*.
**Description of *Candidatus* Ornithospirochaeta avicola sp. nov.**
*Candidatus* Ornithospirochaeta avicola (a.vi’co.la. L. fem. n. *avis* bird; L. suff. *-cola* inhabitant of; N.L. n. *avicola* inhabitant of birds)
A bacterial species identified by metagenomic analyses. This species includes all bacteria with genomes that show ≥95% average nucleotide identity (ANI) to the type genome, which has been assigned the MAG ID Gambia11-129 and which is available via NCBI BioSample SAMN15816993. The GC content of the type genome is 42.81% and the genome length is 1.5 Mbp.
**Description of *Candidatus* Ornithospirochaeta stercoravium sp. nov.**
*Candidatus* Ornithospirochaeta stercoravium (ster.cor.a’vi.um. L. neut. n. *stercus* dung; L. fem. n. *avis* bird; N.L. gen. n. *stercoravium* of bird faeces)
A bacterial species identified by metagenomic analyses. This species includes all bacteria with genomes that show ≥95% average nucleotide identity (ANI) to the type genome, which has been assigned the MAG ID 14700 and which is available via NCBI BioSample SAMN15816953. The GC content of the type genome is 46.26% and the genome length is 2.0 Mbp.
**Description of *Candidatus* Ornithospirochaeta stercorigallinarum sp. nov.**
*Candidatus* Ornithospirochaeta stercorigallinarum (ster.co.ri.gal.li.na’rum. L. neut. n. *stercus* dung; L. fem. n. *gallina* hen; N.L. gen. n. *stercorigallinarum* of hen faeces)
A bacterial species identified by metagenomic analyses. This species includes all bacteria with genomes that show ≥95% average nucleotide identity (ANI) to the type genome, which has been assigned the MAG ID ChiHecec3B27-9561 and which is available via NCBI BioSample SAMN15816957. The GC content of the type genome is 46.77% and the genome length is 1.9 Mbp.
**Description of *Candidatus* Ornithospirochaeta stercoripullorum sp. nov.**
*Candidatus* Ornithospirochaeta stercoripullorum (ster.co.ri.pul.lo’rum. L. neut. n. *stercus* dung; L. masc. n. *pullus* a young chicken; N.L. gen. n. *stercoripullorum* of the faceces of young chickens)
A bacterial species identified by metagenomic analyses. This species includes all bacteria with genomes that show ≥95% average nucleotide identity (ANI) to the type genome, which has been assigned the MAG ID 7293 and which is available via NCBI BioSample SAMN15816978. The GC content of the type genome is 45.57% and the genome length is 2.0 Mbp.
**Description of *Candidatus* Oscillibacter avistercoris sp. nov.**
*Candidatus* Oscillibacter avistercoris (a.vi.ster’co.ris. L. fem. n. *avis* bird; L. neut. n. *stercus* dung; N.L. gen. n. *avistercoris* of bird faeces)
A bacterial species identified by metagenomic analyses. This species includes all bacteria with genomes that show ≥95% average nucleotide identity (ANI) to the type genome, which has been assigned the MAG ID CHK176-14096 and which is available via NCBI BioSample SAMN15816820. This is a new name for the alphanumeric GTDB species sp900556925. The GC content of the type genome is 63.55% and the genome length is 2.3 Mbp.
**Description of *Candidatus* Oscillibacter excrementavium sp. nov.**
*Candidatus* Oscillibacter excrementavium (ex.cre.ment.a’vi.um. L. neut. n. *excrementum* excrement; L. fem. n. *avis* bird; N.L. gen. n. *excrementavium* of bird excrement)
A bacterial species identified by metagenomic analyses. This species includes all bacteria with genomes that show ≥95% average nucleotide identity (ANI) to the type genome, which has been assigned the MAG ID 5302 and which is available via NCBI BioSample SAMN15816661. The GC content of the type genome is 63.73% and the genome length is 2.5 Mbp.

**Table:** 

**Description of *Candidatus* Oscillibacter excrementigallinarum sp. nov.**
*Candidatus* Oscillibacter excrementigallinarum (ex.cre.men.ti.gal.li.na’rum. L. neut. n. *excrementum* excrement; L. fem. n. *gallina* hen; N.L. gen. n. *excrementigallinarum* of hen excrement)
A bacterial species identified by metagenomic analyses. This species includes all bacteria with genomes that show ≥95% average nucleotide identity (ANI) to the type genome, which has been assigned the MAG ID ChiBcec18-1249 and which is available via NCBI BioSample SAMN15816667. The GC content of the type genome is 64.01% and the genome length is 2.4 Mbp.
**Description of *Candidatus* Oscillibacter pullicola sp. nov.**
*Candidatus* Oscillibacter pullicola (pul.li’co.la. L. masc. n. *pullus* a young chicken; L. suff. *-cola* inhabitant of; N.L. n. *pullicola* an inhabitant of young chickens)
A bacterial species identified by metagenomic analyses. This species includes all bacteria with genomes that show ≥95% average nucleotide identity (ANI) to the type genome, which has been assigned the MAG ID ChiBcolR2-4535 and which is available via NCBI BioSample SAMN15816652. The GC content of the type genome is 63.62% and the genome length is 2.4 Mbp.
**Description of *Candidatus* Paenalcaligenes intestinipullorum sp. nov.**
*Candidatus* Paenalcaligenes intestinipullorum (in.tes.ti.ni.pul.lo’rum. L. neut. n. *intestinum* gut; L. masc. n. *pullus* a young chicken; N.L. gen. n. *intestinipullorum* of the gut of young chickens)
A bacterial species identified by metagenomic analyses. This species includes all bacteria with genomes that show ≥95% average nucleotide identity (ANI) to the type genome, which has been assigned the MAG ID 9264 and which is available via NCBI BioSample SAMN15816786. The GC content of the type genome is 51.92% and the genome length is 1.8 Mbp.
**Description of *Candidatus* Paenibacillus intestinavium sp. nov.**
*Candidatus* Paenibacillus intestinavium (in.tes.tin.a’vi.um. L. neut. n. *intestinum* gut; L. fem. n. *avis* bird; N.L. gen. n. *intestinavium* of the gut of birds)
A bacterial species identified by metagenomic analyses. This species includes all bacteria with genomes that show ≥95% average nucleotide identity (ANI) to the type genome, which has been assigned the MAG ID CHK172-12487 and which is available via NCBI BioSample SAMN15816909. Although GTDB has assigned this species to the genus it calls Paenibacillus_C, this genus designation cannot be incorporated into a well-formed binomial, so in naming this species, we have used the current validly published name for the genus. The GC content of the type genome is 39.31% and the genome length is 4.6 Mbp.
**Description of *Candidatus* Parabacteroides faecavium sp. nov.**
*Candidatus* Parabacteroides faecavium (faec.a’vi.um. L. fem. n. *faex, faecis* excrement; L. fem. n. *avis* bird; N.L. gen. n. *faecavium* of bird faeces)
A bacterial species identified by metagenomic analyses. This species includes all bacteria with genomes that show ≥95% average nucleotide identity (ANI) to the type genome, which has been assigned the MAG ID CHK152-2511 and which is available via NCBI BioSample SAMN15816864. This is a new name for the alphanumeric GTDB species sp000436495. The GC content of the type genome is 42.40% and the genome length is 3.4 Mbp.
**Description of *Candidatus* Parabacteroides intestinavium sp. nov.**
*Candidatus* Parabacteroides intestinavium (in.tes.tin.a’vi.um. L. neut. n. *intestinum* gut; L. fem. n. *avis* bird; N.L. gen. n. *intestinavium* of the gut of birds)
A bacterial species identified by metagenomic analyses. This species includes all bacteria with genomes that show ≥95% average nucleotide identity (ANI) to the type genome, which has been assigned the MAG ID ChiHjej11B10-3189 and which is available via NCBI BioSample SAMN15816658. The GC content of the type genome is 44.93% and the genome length is 2.8 Mbp.
**Description of *Candidatus* Parabacteroides intestinigallinarum sp. nov.**
*Candidatus* Parabacteroides intestinigallinarum (in.tes.ti.ni.gal.li.na’rum. L. neut. n. *intestinum* gut; L. fem. n. *gallina* hen; N.L. gen. n. *intestinigallinarum* of the gut of the hens)
A bacterial species identified by metagenomic analyses. This species includes all bacteria with genomes that show ≥95% average nucleotide identity (ANI) to the type genome, which has been assigned the MAG ID ChiHecec2B26-12326 and which is available via NCBI BioSample SAMN15816728. The GC content of the type genome is 52.90% and the genome length is 2.9 Mbp.
**Description of *Candidatus* Parabacteroides intestinipullorum sp. nov.**
*Candidatus* Parabacteroides intestinipullorum (in.tes.ti.ni.pul.lo’rum. L. neut. n. *intestinum* gut; L. masc. n. *pullus* a young chicken; N.L. gen. n. *intestinipullorum* of the gut of young chickens)
A bacterial species identified by metagenomic analyses. This species includes all bacteria with genomes that show ≥95% average nucleotide identity (ANI) to the type genome, which has been assigned the MAG ID ChiGjej6B6-14162 and which is available via NCBI BioSample SAMN15816857. This is a new name for the alphanumeric GTDB species sp900552415. The GC content of the type genome is 50.53% and the genome length is 3.2 Mbp.

**Table:** 

**Description of *Candidatus* Paralactobacillus gallistercoris sp. nov.**
*Candidatus* Paralactobacillus gallistercoris (gal.li.ster’co.ris. L. masc. n *gallus* chicken; L. neut. n. *stercus* dung; N.L. gen. n. *gallistercoris* of chicken faeces)
A bacterial species identified by metagenomic analyses. This species includes all bacteria with genomes that show ≥95% average nucleotide identity (ANI) to the type genome, which has been assigned the MAG ID F6-6636 and which is available via NCBI BioSample SAMN15816781. The GC content of the type genome is 35.69% and the genome length is 1.2 Mbp.
**Description of *Candidatus* Paraprevotella stercoravium sp. nov.**
*Candidatus* Paraprevotella stercoravium (ster.cor.a’vi.um. L. neut. n. *stercus* dung; L. fem. n. *avis* bird; N.L. gen. n. *stercoravium* of bird faeces)
A bacterial species identified by metagenomic analyses. This species includes all bacteria with genomes that show ≥95% average nucleotide identity (ANI) to the type genome, which has been assigned the MAG ID G3-2149 and which is available via NCBI BioSample SAMN15816669. The GC content of the type genome is 45.06% and the genome length is 3.2 Mbp.
**Description of *Candidatus* Paraprevotella stercorigallinarum sp. nov.**
*Candidatus* Paraprevotella stercorigallinarum (ster.co.ri.gal.li.na’rum. L. neut. n. *stercus* dung; L. fem. n. *gallina* hen; N.L. gen. n. *stercorigallinarum* of hen faeces)
A bacterial species identified by metagenomic analyses. This species includes all bacteria with genomes that show ≥95% average nucleotide identity (ANI) to the type genome, which has been assigned the MAG ID 11093 and which is available via NCBI BioSample 3SAMN15816852. This is a new name for the alphanumeric GTDB species sp900546665. The GC content of the type genome is 43.79% and the genome length is 2.9 Mbp.
**Description of *Candidatus* Parasutterella gallistercoris sp. nov.**
*Candidatus* Parasutterella gallistercoris (gal.li.ster’co.ris. L. masc. n *gallus* chicken; L. neut. n. *stercus* dung; N.L. gen. n. *gallistercoris* of chicken faeces)
A bacterial species identified by metagenomic analyses. This species includes all bacteria with genomes that show ≥95% average nucleotide identity (ANI) to the type genome, which has been assigned the MAG ID 21611 and which is available via NCBI BioSample SAMN15816870. This is a new name for the alphanumeric GTDB species sp000980495. The GC content of the type genome is 49.58% and the genome length is 1.9 Mbp.
**Description of *Candidatus* Pelethenecus gen. nov.**
*Candidatus* Pelethenecus (Pe.leth.en.e’cus. Gr. masc. n. *pelethos* dung; Gr. masc. *enoikos* inhabitant; N.L. masc. n. *Pelethenecus* a microbe associated with faeces)
A bacterial genus identified by metagenomic analyses. The genus includes all bacteria with genomes that show ≥60% average amino acid identity (AAI) to the type genome from the type species *Candidatus* Pelethenecus faecipullorum. This is a name for the alphanumeric GTDB genus UMGS268. This genus has been assigned by GTDB-Tk v1.3.0 working on GTDB Release 05-RS95 (Chaumeil et al., 2019; Parks et al., 2020) to the order *Acholeplasmatales* and to the family *Anaeroplasmataceae*.
**Description of *Candidatus* Pelethenecus faecipullorum sp. nov.**
*Candidatus* Pelethenecus faecipullorum (fae.ci.pul.lo’rum. L. fem. n. *faex, faecis* excrement; L. masc. n. *pullus* a young chicken; N.L. gen. n. *faecipullorum* of young chicken faeces)
A bacterial species identified by metagenomic analyses. This species includes all bacteria with genomes that show ≥95% average nucleotide identity (ANI) to the type genome, which has been assigned the MAG ID ChiW17-6978 and which is available via NCBI BioSample SAMN15817226. This is a new name for the alphanumeric GTDB species sp900540175. The GC content of the type genome is 39.85% and the genome length is 1.3 Mbp.
**Description of *Candidatus* Pelethocola gen. nov.**
*Candidatus* Pelethocola (Pe.le.tho’co.la. Gr. masc. n. *pelethos* dung; L. suff. *-cola* inhabitant of; N.L. fem. n. *Pelethocola* a microbe associated with faeces)
A bacterial genus identified by metagenomic analyses. The genus includes all bacteria with genomes that show ≥60% average amino acid identity (AAI) to the type genome from the type species *Candidatus* Pelethocola excrementipullorum. This is a name for the alphanumeric GTDB genus UBA5416. This genus has been assigned by GTDB-Tk v1.3.0 working on GTDB Release 05-RS95 (Chaumeil et al., 2019; Parks et al., 2020) to the order *Lachnospirales* and to the family *Lachnospiraceae*.
**Description of *Candidatus* Pelethocola excrementipullorum sp. nov.**
*Candidatus* Pelethocola excrementipullorum (ex.cre.men.ti.pul.lo’rum. L. neut. n. *excrementum* excrement; L. masc. n. *pullus* a young chicken; N.L. gen. n. *excrementipullorum* of young chicken excrement)
A bacterial species identified by metagenomic analyses. This species includes all bacteria with genomes that show ≥95% average nucleotide identity (ANI) to the type genome, which has been assigned the MAG ID CHK160-5124 and which is available via NCBI BioSample SAMN15817143. The GC content of the type genome is 43.72% and the genome length is 3.9 Mbp.

**Table:** 

**Description of *Candidatus* Pelethomonas gen. nov.**
*Candidatus* Pelethomonas (Pe.le.tho.mo’nas. Gr. masc. n. *pelethos* dung; L. fem. n. *monas* a monad; N.L. fem. n. *Pelethomonas* a microbe associated with faeces)
A bacterial genus identified by metagenomic analyses. The genus includes all bacteria with genomes that show ≥60% average amino acid identity (AAI) to the type genome from the type species *Candidatus* Pelethomonas intestinigallinarum. This is a name for the alphanumeric GTDB genus UMGS1872. This genus has been assigned by GTDB-Tk v1.3.0 working on GTDB Release 05-RS95 (Chaumeil et al., 2019; Parks et al., 2020) to the order *Oscillospirales* and to the family *Oscillospiraceae*.
**Description of *Candidatus* Pelethomonas intestinigallinarum sp. nov.**
*Candidatus* Pelethomonas intestinigallinarum (in.tes.ti.ni.gal.li.na’rum. L. neut. n. *intestinum* gut; L. fem. n. *gallina* hen; N.L. gen. n. *intestinigallinarum* of the gut of the hens)
A bacterial species identified by metagenomic analyses. This species includes all bacteria with genomes that show ≥95% average nucleotide identity (ANI) to the type genome, which has been assigned the MAG ID ChiSjej2B20-3600 and which is available via NCBI BioSample SAMN15817014. The GC content of the type genome is 63.89% and the genome length is 2.2 Mbp.
**Description of *Candidatus* Pelethosoma gen. nov.**
*Candidatus* Pelethosoma (Pe.le.tho.so’ma. Gr. masc. n. *pelethos* dung; Gr. neut. n. *soma* a body; N.L. neut. n. *Pelethosoma* a microbe associated with faeces)
A bacterial genus identified by metagenomic analyses. The genus includes all bacteria with genomes that show ≥60% average amino acid identity (AAI) to the type genome from the type species *Candidatus* Pelethosoma merdigallinarum. This is a name for the alphanumeric GTDB genus UMGS2016. This genus has been assigned by GTDB-Tk v1.3.0 working on GTDB Release 05-RS95 (Chaumeil et al., 2019; Parks et al., 2020) to the order *RF39* and to the family *CAG-822*.
**Description of *Candidatus* Pelethosoma merdigallinarum sp. nov.**
*Candidatus* Pelethosoma merdigallinarum (mer.di.gal.li.na’rum. L. fem. n. *merda* faeces; L. fem. n. *gallina* hen; N.L. gen. n. *merdigallinarum* of hen faeces)
A bacterial species identified by metagenomic analyses. This species includes all bacteria with genomes that show ≥95% average nucleotide identity (ANI) to the type genome, which has been assigned the MAG ID CHK195-5794 and which is available via NCBI BioSample SAMN15817039. The GC content of the type genome is 30.57% and the genome length is 1.3 Mbp.
**Description of *Candidatus* Pelethousia gen. nov.**
*Candidatus* Pelethousia (Pe.leth.ou’si.a. Gr. masc. n. *pelethos* dung; Gr. fem. n. *ousia* an essence; N.L. fem. n. *Pelethousia* a microbe associated with faeces)
A bacterial genus identified by metagenomic analyses. The genus includes all bacteria with genomes that show ≥60% average amino acid identity (AAI) to the type genome from the type species *Candidatus* Pelethousia gallinarum. This is a name for the alphanumeric GTDB genus UBA5394. This genus has been assigned by GTDB-Tk v1.3.0 working on GTDB Release 05-RS95 (Chaumeil et al., 2019; Parks et al., 2020) to the order *Christensenellales* and to the family *CAG-138*.
**Description of *Candidatus* Pelethousia gallinarum sp. nov.**
*Candidatus* Pelethousia gallinarum (gal.li.na’rum. L. fem. n. *gallina* a hen; L. fem. gen. pl. n. *gallinarum* of hens)
A bacterial species identified by metagenomic analyses. This species includes all bacteria with genomes that show ≥95% average nucleotide identity (ANI) to the type genome, which has been assigned the MAG ID ChiHcec27-1353 and which is available via NCBI BioSample SAMN15817178. This is a new name for the alphanumeric GTDB species sp003150565. The GC content of the type genome is 59.40% and the genome length is 2.0 Mbp.
**Description of *Candidatus* Phascolarctobacterium stercoravium sp. nov.**
*Candidatus* Phascolarctobacterium stercoravium (ster.cor.a’vi.um. L. neut. n. *stercus* dung; L. fem. n. *avis* bird; N.L. gen. n. *stercoravium* of bird faeces)
A bacterial species identified by metagenomic analyses. This species includes all bacteria with genomes that show ≥95% average nucleotide identity (ANI) to the type genome, which has been assigned the MAG ID ChiBcec14-732 and which is available via NCBI BioSample SAMN15816834. This is a new name for the alphanumeric GTDB species sp000436095. The GC content of the type genome is 46.50% and the genome length is 1.7 Mbp.

**Table:** 

**Description of *Candidatus* Phocaeicola caecigallinarum sp. nov.**
*Candidatus* Phocaeicola caecigallinarum (cae.ci.gal.li.na’rum. L. neut. n. *caecum* the caecum; L. fem. n. *gallina* a hen; N.L. gen. n. *caecigallinarum* of the caecum of hens)
A bacterial species identified by metagenomic analyses. This species includes all bacteria with genomes that show ≥95% average nucleotide identity (ANI) to the type genome, which has been assigned the MAG ID ChiHjej11B10-3694 and which is available via NCBI BioSample SAMN15816802. This is a new name for the alphanumeric GTDB species sp002161565. The GC content of the type genome is 46.18% and the genome length is 3.2 Mbp.
**Description of *Candidatus* Phocaeicola excrementigallinarum sp. nov.**
*Candidatus* Phocaeicola excrementigallinarum (ex.cre.men.ti.gal.li.na’rum. L. neut. n. *excrementum* excrement; L. fem. n. *gallina* hen; N.L. gen. n. *excrementigallinarum* of hen excrement)
A bacterial species identified by metagenomic analyses. This species includes all bacteria with genomes that show ≥95% average nucleotide identity (ANI) to the type genome, which has been assigned the MAG ID 12279 and which is available via NCBI BioSample SAMN15816632. The GC content of the type genome is 50.57% and the genome length is 2.5 Mbp.
**Description of *Candidatus* Phocaeicola excrementipullorum sp. nov.**
*Candidatus* Phocaeicola excrementipullorum (ex.cre.men.ti.pul.lo’rum. L. neut. n. *excrementum* excrement; L. masc. n. *pullus* a young chicken; N.L. gen. n. *excrementipullorum* of young chicken excrement)
A bacterial species identified by metagenomic analyses. This species includes all bacteria with genomes that show ≥95% average nucleotide identity (ANI) to the type genome, which has been assigned the MAG ID 8470 and which is available via NCBI BioSample SAMN15816808. This is a new name for the alphanumeric GTDB species sp900546095. The GC content of the type genome is 49.09% and the genome length is 3.1 Mbp.
**Description of *Candidatus* Phocaeicola faecigallinarum sp. nov.**
*Candidatus* Phocaeicola faecigallinarum (fae.ci.gal.li.na’rum. L. fem. n. *faex, faecis* excrement; L. fem. n. *gallina* hen; N.L. gen. n. *faecigallinarum* of hen faeces)
A bacterial species identified by metagenomic analyses. This species includes all bacteria with genomes that show ≥95% average nucleotide identity (ANI) to the type genome, which has been assigned the MAG ID G4-2901 and which is available via NCBI BioSample SAMN15816657. The GC content of the type genome is 40.33% and the genome length is 3.3 Mbp.
**Description of *Candidatus* Phocaeicola faecipullorum sp. nov.**
*Candidatus* Phocaeicola faecipullorum (fae.ci.pul.lo’rum. L. fem. n. *faex, faecis* excrement; L. masc. n. *pullus* a young chicken; N.L. gen. n. *faecipullorum* of young chicken faeces)
A bacterial species identified by metagenomic analyses. This species includes all bacteria with genomes that show ≥95% average nucleotide identity (ANI) to the type genome, which has been assigned the MAG ID 17637 and which is available via NCBI BioSample SAMN15816682. The GC content of the type genome is 39.94% and the genome length is 3.9 Mbp.
**Description of *Candidatus* Phocaeicola gallinarum sp. nov.**
*Candidatus* Phocaeicola gallinarum (gal.li.na’rum. L. fem. n. *gallina* a hen; L. gen. pl. n. gallinarum of hens)
A bacterial species identified by metagenomic analyses. This species includes all bacteria with genomes that show ≥95% average nucleotide identity (ANI) to the type genome, which has been assigned the MAG ID ChiGjej6B6-595 and which is available via NCBI BioSample SAMN15816805. This is a new name for the alphanumeric GTDB species sp900540105. The GC content of the type genome is 45.79% and the genome length is 2.8 Mbp.
**Description of *Candidatus* Phocaeicola gallistercoris sp. nov.**
*Candidatus* Phocaeicola gallistercoris (gal.li.ster’co.ris. L. masc. n *gallus* chicken; L. neut. n. *stercus* dung; N.L. gen. n. *gallistercoris* of chicken faeces)
A bacterial species identified by metagenomic analyses. This species includes all bacteria with genomes that show ≥95% average nucleotide identity (ANI) to the type genome, which has been assigned the MAG ID Gambia9-593 and which is available via NCBI BioSample SAMN15816698. The GC content of the type genome is 38.34% and the genome length is 2.3 Mbp.
**Description of *Candidatus* Phocaeicola merdavium sp. nov.**
*Candidatus* Phocaeicola merdavium (merd.a’vi.um. L. fem. n. *merda* faeces; L. fem. n. *avis* bird; N.L. gen. n. *merdavium* of bird faeces)
A bacterial species identified by metagenomic analyses. This species includes all bacteria with genomes that show ≥95% average nucleotide identity (ANI) to the type genome, which has been assigned the MAG ID CHK136-5299 and which is available via NCBI BioSample SAMN15816804. This is a new name for the alphanumeric GTDB species sp002161765. The GC content of the type genome is 44.54% and the genome length is 2.6 Mbp.

**Table:** 

**Description of *Candidatus* Phocaeicola merdigallinarum sp. nov.**
*Candidatus* Phocaeicola merdigallinarum (mer.di.gal.li.na’rum. L. fem. n. *merda* faeces; L. fem. n. *gallina* hen; N.L. gen. n. *merdigallinarum* of hen faeces)
A bacterial species identified by metagenomic analyses. This species includes all bacteria with genomes that show ≥95% average nucleotide identity (ANI) to the type genome, which has been assigned the MAG ID 17689 and which is available via NCBI BioSample SAMN15816829. This is a new name for the alphanumeric GTDB species sp900066455. The GC content of the type genome is 46.19% and the genome length is 3.3 Mbp.
**Description of *Candidatus* Prevotella avicola sp. nov.**
*Candidatus* Prevotella avicola (a.vi’co.la. L. fem. n. *avis* bird; L. suff. *-cola* inhabitant of; N.L. n. *avicola* inhabitant of birds)
A bacterial species identified by metagenomic analyses. This species includes all bacteria with genomes that show ≥95% average nucleotide identity (ANI) to the type genome, which has been assigned the MAG ID ChiHecec3B27-8219 and which is available via NCBI BioSample SAMN15816846. This is a new name for the alphanumeric GTDB species sp000435635. The GC content of the type genome is 51.22% and the genome length is 1.9 Mbp.
**Description of *Candidatus* Prevotella intestinigallinarum sp. nov.**
*Candidatus* Prevotella intestinigallinarum (in.tes.ti.ni.gal.li.na’rum. L. neut. n. *intestinum* gut; L. fem. n. *gallina* hen; N.L. gen. n. *intestinigallinarum* of the gut of the hens)
A bacterial species identified by metagenomic analyses. This species includes all bacteria with genomes that show ≥95% average nucleotide identity (ANI) to the type genome, which has been assigned the MAG ID 146 and which is available via NCBI BioSample SAMN15816872. This is a new name for the alphanumeric GTDB species sp900540415. The GC content of the type genome is 56.37% and the genome length is 2.9 Mbp.
**Description of *Candidatus* Prevotella stercoripullorum sp. nov.**
*Candidatus* Prevotella stercoripullorum (ster.co.ri.pul.lo’rum. L. neut. n. *stercus* dung; L. masc. n. *pullus* a young chicken; N.L. gen. n. *stercoripullorum* of the faceces of young chickens)
A bacterial species identified by metagenomic analyses. This species includes all bacteria with genomes that show ≥95% average nucleotide identity (ANI) to the type genome, which has been assigned the MAG ID USASDec6-549 and which is available via NCBI BioSample SAMN15816866. This is a new name for the alphanumeric GTDB species sp900554045. The GC content of the type genome is 53.35% and the genome length is 2.5 Mbp.
**Description of *Candidatus* Protoclostridium stercorigallinarum sp. nov.**
*Candidatus* Protoclostridium stercorigallinarum (ster.co.ri.gal.li.na’rum. L. neut. n. *stercus* dung; L. fem. n. *gallina* hen; N.L. gen. n. *stercorigallinarum* of hen faeces)
A bacterial species identified by metagenomic analyses. This species includes all bacteria with genomes that show ≥95% average nucleotide identity (ANI) to the type genome, which has been assigned the MAG ID 12435 and which is available via NCBI BioSample SAMN15816772. The GC content of the type genome is 56.70% and the genome length is 1.7 Mbp.
**Description of *Candidatus* Pseudogracilibacillus intestinigallinarum sp. nov.**
*Candidatus* Pseudogracilibacillus intestinigallinarum (in.tes.ti.ni.gal.li.na’rum. L. neut. n. *intestinum* gut; L. fem. n. *gallina* hen; N.L. gen. n. *intestinigallinarum* of the gut of the hens)
A bacterial species identified by metagenomic analyses. This species includes all bacteria with genomes that show ≥95% average nucleotide identity (ANI) to the type genome, which has been assigned the MAG ID CHK169-2315 and which is available via NCBI BioSample SAMN15816775. The GC content of the type genome is 35.03% and the genome length is 2.5 Mbp.
**Description of *Candidatus* Pseudomonas excrementavium sp. nov.**
*Candidatus* Pseudomonas excrementavium (ex.cre.ment.a’vi.um. L. neut. n. *excrementum* excrement; L. fem. n. *avis* bird; N.L. gen. n. *excrementavium* of bird excrement)
A bacterial species identified by metagenomic analyses. This species includes all bacteria with genomes that show ≥95% average nucleotide identity (ANI) to the type genome, which has been assigned the MAG ID CHK174-787 and which is available via NCBI BioSample SAMN15816898. Although GTDB has assigned this species to the genus it calls Pseudomonas_D, this genus designation cannot be incorporated into a well-formed binomial, so in naming this species, we have used the current validly published name for the genus. The GC content of the type genome is 61.84% and the genome length is 3.0 Mbp.

**Table:** 

**Description of *Candidatus* Pullibacteroides gen. nov.**
*Candidatus* Pullibacteroides (Pul.li.bac.te.ro’i.des. L. masc. n. *pullus* a young chicken; N.L. masc. n. *Bacteroides* a genus name; N.L. masc. n. *Pullibacteroides* a genus related to the genus *Bacteroides* but distinct from it and found in poultry)
A bacterial genus identified by metagenomic analyses. The genus includes all bacteria with genomes that show ≥60% average amino acid identity (AAI) to the type genome from the type species *Candidatus* Pullibacteroides excrementavium. This genus has been assigned by GTDB-Tk v1.3.0 working on GTDB Release 05-RS95 (Chaumeil et al., 2019; Parks et al., 2020) to the order *Bacteroidales* and to the family *P3*.
**Description of *Candidatus* Pullibacteroides excrementavium sp. nov.**
*Candidatus* Pullibacteroides excrementavium (ex.cre.ment.a’vi.um. L. neut. n. *excrementum* excrement; L. fem. n. *avis* bird; N.L. gen. n. *excrementavium* of bird excrement)
A bacterial species identified by metagenomic analyses. This species includes all bacteria with genomes that show ≥95% average nucleotide identity (ANI) to the type genome, which has been assigned the MAG ID 2889 and which is available via NCBI BioSample SAMN15816989. The GC content of the type genome is 51.34% and the genome length is 2.4 Mbp.
**Description of *Candidatus* Pullichristensenella gen. nov.**
*Candidatus* Pullichristensenella (Pul.li.chris.ten.sen.el’la. L. masc. n. *pullus* a young chicken; N.L. fem. n. *Christensenella* a genus name; N.L. fem. n. *Pullichristensenella* a genus related to the genus *Christensenella* but distinct from it and found in poultry)
A bacterial genus identified by metagenomic analyses. The genus includes all bacteria with genomes that show ≥60% average amino acid identity (AAI) to the type genome from the type species *Candidatus* Pullichristensenella avicola. This genus has been assigned by GTDB-Tk v1.3.0 working on GTDB Release 05-RS95 (Chaumeil et al., 2019; Parks et al., 2020) to the order *Christensenellales* and to the family *CAG-74*.
**Description of *Candidatus* Pullichristensenella avicola sp. nov.**
*Candidatus* Pullichristensenella avicola (a.vi’co.la. L. fem. n. *avis* bird; L. suff. *-cola* inhabitant of; N.L. n. *avicola* inhabitant of birds)
A bacterial species identified by metagenomic analyses. This species includes all bacteria with genomes that show ≥95% average nucleotide identity (ANI) to the type genome, which has been assigned the MAG ID 10205 and which is available via NCBI BioSample SAMN15816956. The GC content of the type genome is 63.02% and the genome length is 2.3 Mbp.
**Description of *Candidatus* Pullichristensenella excrementigallinarum sp. nov.**
*Candidatus* Pullichristensenella excrementigallinarum (ex.cre.men.ti.gal.li.na’rum. L. neut. n. *excrementum* excrement; L. fem. n. *gallina* hen; N.L. gen. n. *excrementigallinarum* of hen excrement)
A bacterial species identified by metagenomic analyses. This species includes all bacteria with genomes that show ≥95% average nucleotide identity (ANI) to the type genome, which has been assigned the MAG ID ChiHcec3-11533 and which is available via NCBI BioSample SAMN15816983. The GC content of the type genome is 57.19% and the genome length is 2.2 Mbp.
**Description of *Candidatus* Pullichristensenella excrementipullorum sp. nov.**
*Candidatus* Pullichristensenella excrementipullorum (ex.cre.men.ti.pul.lo’rum. L. neut. n. *excrementum* excrement; L. masc. n. *pullus* a young chicken; N.L. gen. n. *excrementipullorum* of young chicken excrement)
A bacterial species identified by metagenomic analyses. This species includes all bacteria with genomes that show ≥95% average nucleotide identity (ANI) to the type genome, which has been assigned the MAG ID 1279 and which is available via NCBI BioSample SAMN15817001. The GC content of the type genome is 63.33% and the genome length is 2.7 Mbp.
**Description of *Candidatus* Pullichristensenella stercorigallinarum sp. nov.**
*Candidatus* Pullichristensenella stercorigallinarum (ster.co.ri.gal.li.na’rum. L. neut. n. *stercus* dung; L. fem. n. *gallina* hen; N.L. gen. n. *stercorigallinarum* of hen faeces)
A bacterial species identified by metagenomic analyses. This species includes all bacteria with genomes that show ≥95% average nucleotide identity (ANI) to the type genome, which has been assigned the MAG ID ChiSjej6B24-2974 and which is available via NCBI BioSample SAMN15816933. The GC content of the type genome is 60.06% and the genome length is 2.7 Mbp.
**Description of *Candidatus* Pullichristensenella stercoripullorum sp. nov.**
*Candidatus* Pullichristensenella stercoripullorum (ster.co.ri.pul.lo’rum. L. neut. n. *stercus* dung; L. masc. n. *pullus* a young chicken; N.L. gen. n. *stercoripullorum* of the faceces of young chickens)
A bacterial species identified by metagenomic analyses. This species includes all bacteria with genomes that show ≥95% average nucleotide identity (ANI) to the type genome, which has been assigned the MAG ID 5266 and which is available via NCBI BioSample SAMN15816952. The GC content of the type genome is 64.02% and the genome length is 2.3 Mbp.

**Table:** 

**Description of *Candidatus* Pullilachnospira gen. nov.**
*Candidatus* Pullilachnospira (Pul.li.lach.no.spi’ra. L. masc. n. *pullus* a young chicken; N.L. fem. n. *Lachnospira* a genus name; N.L. fem. n. *Pullilachnospira* a genus related to the genus *Lachnospira* but distinct from it and found in poultry)
A bacterial genus identified by metagenomic analyses. The genus includes all bacteria with genomes that show ≥60% average amino acid identity (AAI) to the type genome from the type species *Candidatus* Pullilachnospira stercoravium. This genus was identified but not named by Glendinning et al. (2020). This genus has been assigned by GTDB-Tk v1.3.0 working on GTDB Release 05-RS95 (Chaumeil et al., 2019; Parks et al., 2020) to the order *Lachnospirales* and to the family *Lachnospiraceae*.
**Description of *Candidatus* Pullilachnospira gallistercoris sp. nov.**
*Candidatus* Pullilachnospira gallistercoris (gal.li.ster’co.ris. L. masc. n *gallus* chicken; L. neut. n. *stercus* dung; N.L. gen. n. *gallistercoris* of chicken faeces)
A bacterial species identified by metagenomic analyses. This species includes all bacteria with genomes that show ≥95% average nucleotide identity (ANI) to the type genome, which has been assigned the MAG ID ChiSjej5B23-6657 and which is available via NCBI BioSample SAMN15816936. The GC content of the type genome is 53.36% and the genome length is 2.5 Mbp.
**Description of *Candidatus* Pullilachnospira intestinigallinarum sp. nov.**
*Candidatus* Pullilachnospira intestinigallinarum (in.tes.ti.ni.gal.li.na’rum. L. neut. n. *intestinum* gut; L. fem. n. *gallina* hen; N.L. gen. n. *intestinigallinarum* of the gut of the hens)
A bacterial species identified by metagenomic analyses. This species includes all bacteria with genomes that show ≥95% average nucleotide identity (ANI) to the type genome, which has been assigned the MAG ID CHK192-16996 and which is available via NCBI BioSample SAMN15816938. The GC content of the type genome is 51.71% and the genome length is 2.8 Mbp.
**Description of *Candidatus* Pullilachnospira stercoravium sp. nov.**
*Candidatus* Pullilachnospira stercoravium (ster.cor.a’vi.um. L. neut. n. *stercus* dung; L. fem. n. *avis* bird; N.L. gen. n. *stercoravium* of bird faeces)
A bacterial species identified by metagenomic analyses. This species includes all bacteria with genomes that show ≥95% average nucleotide identity (ANI) to the type genome, which has been assigned the MAG ID ChiBcec2-4451 and which is available via NCBI BioSample SAMN15816944. The GC content of the type genome is 53.14% and the genome length is 2.8 Mbp.
**Description of *Candidatus* Pygmaiobacter gallistercoris sp. nov.**
*Candidatus* Pygmaiobacter gallistercoris (gal.li.ster’co.ris. L. masc. n. *gallus* chicken; L. neut. n. *stercus* dung; N.L. gen. n. *gallistercoris* of chicken faeces)
A bacterial species identified by metagenomic analyses. This species includes all bacteria with genomes that show ≥95% average nucleotide identity (ANI) to the type genome, which has been assigned the MAG ID ChiGjej6B6-17065 and which is available via NCBI BioSample SAMN15816776. The GC content of the type genome is 61.74% and the genome length is 1.6 Mbp.
**Description of *Candidatus* Rikenella faecigallinarum sp. nov.**
*Candidatus* Rikenella faecigallinarum (fae.ci.gal.li.na’rum. L. fem. n. *faex, faecis* excrement; L. fem. n. *gallina* hen; N.L. gen. n. *faecigallinarum* of hen faeces)
A bacterial species identified by metagenomic analyses. This species includes all bacteria with genomes that show ≥95% average nucleotide identity (ANI) to the type genome, which has been assigned the MAG ID ChiBcec15-1070 and which is available via NCBI BioSample SAMN15816768. The GC content of the type genome is 56.15% and the genome length is 1.8 Mbp.
**Description of *Candidatus* Rothia avicola sp. nov.**
*Candidatus* Rothia avicola (a.vi’co.la. L. fem. n. *avis* bird; L. suff. *-cola* inhabitant of; N.L. n. *avicola* inhabitant of birds)
A bacterial species identified by metagenomic analyses. This species includes all bacteria with genomes that show ≥95% average nucleotide identity (ANI) to the type genome, which has been assigned the MAG ID ChiHjej12B11-9195 and which is available via NCBI BioSample SAMN15816701. The GC content of the type genome is 60.05% and the genome length is 2.1 Mbp.
**Description of *Candidatus* Rothia avistercoris sp. nov.**
*Candidatus* Rothia avistercoris (a.vi.ster’co.ris. L. fem. n. *avis* bird; L. neut. n. *stercus* dung; N.L. gen. n. *avistercoris* of bird faeces)
A bacterial species identified by metagenomic analyses. This species includes all bacteria with genomes that show ≥95% average nucleotide identity (ANI) to the type genome, which has been assigned the MAG ID ChiHjej10B9-4811 and which is available via NCBI BioSample SAMN15816788. The GC content of the type genome is 59.79% and the genome length is 2.0 Mbp.

**Table:** 

**Description of *Candidatus* Ruania gallistercoris sp. nov.**
*Candidatus* Ruania gallistercoris (gal.li.ster’co.ris. L. masc. n. *gallus* chicken; L. neut. n. *stercus* dung; N.L. gen. n. *gallistercoris* of chicken faeces)
A bacterial species identified by metagenomic analyses. This species includes all bacteria with genomes that show ≥95% average nucleotide identity (ANI) to the type genome, which has been assigned the MAG ID ChiGjej4B4-7305 and which is available via NCBI BioSample SAMN15816695. The GC content of the type genome is 69.62% and the genome length is 4.4 Mbp.
**Description of *Candidatus* Rubneribacter avistercoris sp. nov.**
*Candidatus* Rubneribacter avistercoris (a.vi.ster’co.ris. L. fem. n. *avis* bird; L. neut. n. *stercus* dung; N.L. gen. n. *avistercoris* of bird faeces)
A bacterial species identified by metagenomic analyses. This species includes all bacteria with genomes that show ≥95% average nucleotide identity (ANI) to the type genome, which has been assigned the MAG ID ChiGjej6B6-20359 and which is available via NCBI BioSample SAMN15816703. The GC content of the type genome is 65.17% and the genome length is 3.2 Mbp.
**Description of *Candidatus* Ruminococcus avistercoris sp. nov.**
*Candidatus* Ruminococcus avistercoris (a.vi.ster’co.ris. L. fem. n. *avis* bird; L. neut. n. *stercus* dung; N.L. gen. n. *avistercoris* of bird faeces)
A bacterial species identified by metagenomic analyses. This species includes all bacteria with genomes that show ≥95% average nucleotide identity (ANI) to the type genome, which has been assigned the MAG ID CHK186-6582 and which is available via NCBI BioSample SAMN15816883. Although GTDB has assigned this species to the genus it calls Ruminococcus_G, this genus designation cannot be incorporated into a well-formed binomial, so in naming this species, we have used the current validly published name for the genus. The GC content of the type genome is 50.07% and the genome length is 2.3 Mbp.
**Description of *Candidatus* Ruminococcus gallistercoris sp. nov.**
*Candidatus* Ruminococcus gallistercoris (gal.li.ster’co.ris. L. masc. n *gallus* chicken; L. neut. n. *stercus* dung; N.L. gen. n. *gallistercoris* of chicken faeces)
A bacterial species identified by metagenomic analyses. This species includes all bacteria with genomes that show ≥95% average nucleotide identity (ANI) to the type genome, which has been assigned the MAG ID ChiBcec12-341 and which is available via NCBI BioSample SAMN15816918. This is a new name for the alphanumeric GTDB species sp900552925. Although GTDB has assigned this species to the genus it calls Ruminococcus_H, this genus designation cannot be incorporated into a well-formed binomial, so in naming this species, we have used the current validly published name for the genus. The GC content of the type genome is 62.03% and the genome length is 2.1 Mbp.
**Description of *Candidatus* Ruminococcus intestinipullorum sp. nov.**
*Candidatus* Ruminococcus intestinipullorum (in.tes.ti.ni.pul.lo’rum. L. neut. n. *intestinum* gut; L. masc. n. *pullus* a young chicken; N.L. gen. n. *intestinipullorum* of the gut of young chickens)
A bacterial species identified by metagenomic analyses. This species includes all bacteria with genomes that show ≥95% average nucleotide identity (ANI) to the type genome, which has been assigned the MAG ID 1485 and which is available via NCBI BioSample SAMN15816905. Although GTDB has assigned this species to the genus it calls Ruminococcus_B, this genus designation cannot be incorporated into a well-formed binomial, so in naming this species, we have used the current validly published name for the genus. The GC content of the type genome is 35.77% and the genome length is 2.1 Mbp.
**Description of *Candidatus* Ruthenibacterium avium sp. nov.**
*Candidatus* Ruthenibacterium avium (a’vi.um. L. fem. pl. n. *avium* of birds)
A bacterial species identified by metagenomic analyses. This species includes all bacteria with genomes that show ≥95% average nucleotide identity (ANI) to the type genome, which has been assigned the MAG ID ChiBcec8-14828 and which is available via NCBI BioSample SAMN15816823. This is a new name for the alphanumeric GTDB species sp002315015. The GC content of the type genome is 51.27% and the genome length is 2.2 Mbp.
**Description of *Candidatus* Ruthenibacterium merdavium sp. nov.**
*Candidatus* Ruthenibacterium merdavium (merd.a’vi.um. L. fem. n. *merda* faeces; L. fem. n. *avis* bird; N.L. gen. n. *merdavium* of bird faeces)
A bacterial species identified by metagenomic analyses. This species includes all bacteria with genomes that show ≥95% average nucleotide identity (ANI) to the type genome, which has been assigned the MAG ID 5933 and which is available via NCBI BioSample SAMN15816578. The GC content of the type genome is 51.15% and the genome length is 2.0 Mbp.
**Description of *Candidatus* Ruthenibacterium merdigallinarum sp. nov.**
*Candidatus* Ruthenibacterium merdigallinarum (mer.di.gal.li.na’rum. L. fem. n. *merda* faeces; L. fem. n. *gallina* hen; N.L. gen. n. *merdigallinarum* of hen faeces)
A bacterial species identified by metagenomic analyses. This species includes all bacteria with genomes that show ≥95% average nucleotide identity (ANI) to the type genome, which has been assigned the MAG ID ChiSjej6B24-7098 and which is available via NCBI BioSample SAMN15816763. The GC content of the type genome is 65.28% and the genome length is 2.2 Mbp.

**Table:** 

**Description of *Candidatus* Ruthenibacterium merdipullorum sp. nov.**
*Candidatus* Ruthenibacterium merdipullorum (mer.di.pul.lo’rum. L. fem. n. *merda* faeces; L. masc. n. *pullus* a young chicken; N.L. gen. n. *merdipullorum* of the faeces of young chickens)
A bacterial species identified by metagenomic analyses. This species includes all bacteria with genomes that show ≥95% average nucleotide identity (ANI) to the type genome, which has been assigned the MAG ID ChiSxjej5B17-15602 and which is available via NCBI BioSample SAMN15816878. This is a new name for the alphanumeric GTDB species sp900546885. The GC content of the type genome is 59.69% and the genome length is 2.1 Mbp.
**Description of *Candidatus* Salinicoccus merdavium sp. nov.**
*Candidatus* Salinicoccus merdavium (merd.a’vi.um. L. fem. n. *merda* faeces; L. fem. n. *avis* bird; N.L. gen. n. *merdavium* of bird faeces)
A bacterial species identified by metagenomic analyses. This species includes all bacteria with genomes that show ≥95% average nucleotide identity (ANI) to the type genome, which has been assigned the MAG ID ChiHjej12B11-20095 and which is available via NCBI BioSample SAMN15816874. This is a new name for the alphanumeric GTDB species sp002360325. The GC content of the type genome is 44.20% and the genome length is 1.8 Mbp.
**Description of *Candidatus* Salinicoccus stercoripullorum sp. nov.**
*Candidatus* Salinicoccus stercoripullorum (ster.co.ri.pul.lo’rum. L. neut. n. *stercus* dung; L. masc. n. *pullus* a young chicken; N.L. gen. n. *stercoripullorum* of the faceces of young chickens)
A bacterial species identified by metagenomic analyses. This species includes all bacteria with genomes that show ≥95% average nucleotide identity (ANI) to the type genome, which has been assigned the MAG ID ChiHjej13B12-752 and which is available via NCBI BioSample SAMN15816771. The GC content of the type genome is 48.14% and the genome length is 2.3 Mbp.
**Description of *Candidatus* Savagella gallinarum sp. nov.**
*Candidatus* Savagella gallinarum (gal.li.na’rum. L. fem. n. *gallina* a hen; L. gen. pl. n. *gallinarum* of hens)
A bacterial species identified by metagenomic analyses. This species includes all bacteria with genomes that show ≥95% average nucleotide identity (ANI) to the type genome, which has been assigned the MAG ID CHK166-5537 and which is available via NCBI BioSample SAMN15816803. This is a new name for the alphanumeric GTDB species sp001655775. The GC content of the type genome is 26.42% and the genome length is 1.8 Mbp.
**Description of *Candidatus* Scatavimonas gen. nov.**
*Candidatus* Scatavimonas (Scat.a.vi.mon’as Gr. neut. n. *skor, skatos* dung; L. fem. n. avis bird; L. fem. n. monas a monad; N.L. fem. n. N.L. neut. n. *Scatavimonas* a microbe associated with bird faeces)
A bacterial genus identified by metagenomic analyses. The genus includes all bacteria with genomes that show ≥60% average amino acid identity (AAI) to the type genome from the type species *Candidatus* Scatavimonas merdigallinarum. This is a name for the alphanumeric GTDB genus UMGS403. This genus has been assigned by GTDB-Tk v1.3.0 working on GTDB Release 05-RS95 (Chaumeil et al., 2019; Parks et al., 2020) to the order *Oscillospirales* and to the family *Acutalibacteraceae*.
**Description of *Candidatus* Scatavimonas merdigallinarum sp. nov.**
*Candidatus* Scatavimonas merdigallinarum (mer.di.gal.li.na’rum. L. fem. n. *merda* faeces; L. fem. n. *gallina* hen; N.L. gen. n. *merdigallinarum* of hen faeces)
A bacterial species identified by metagenomic analyses. This species includes all bacteria with genomes that show ≥95% average nucleotide identity (ANI) to the type genome, which has been assigned the MAG ID ChiSjej1B19-3389 and which is available via NCBI BioSample SAMN15817212. This is a new name for the alphanumeric GTDB species sp900541975. The GC content of the type genome is 46.67% and the genome length is 1.7 Mbp.
**Description of *Candidatus* Scatenecus gen. nov.**
*Candidatus* Scatenecus (Scat.en.e’cus. Gr. neut. n. *skor, skatos* dung; Gr. masc. *enoikos* inhabitant; N.L. masc. n. *Scatenecus* a microbe associated with the intestines)
A bacterial genus identified by metagenomic analyses. The genus includes all bacteria with genomes that show ≥60% average amino acid identity (AAI) to the type genome from the type species *Candidatus* Scatenecus faecavium. This is a name for the alphanumeric GTDB genus QAMI01. This genus has been assigned by GTDB-Tk v1.3.0 working on GTDB Release 05-RS95 (Chaumeil et al., 2019; Parks et al., 2020) to the order *Gastranaerophilales* and to the family *Gastranaerophilaceae*.

**Table:** 

**Description of *Candidatus* Scatenecus faecavium sp. nov.**
*Candidatus* Scatenecus faecavium (faec.a’vi.um. L. fem. n. *faex, faecis* excrement; L. fem. n. *avis* bird; N.L. gen. n. *faecavium* of bird faeces)
A bacterial species identified by metagenomic analyses. This species includes all bacteria with genomes that show ≥95% average nucleotide identity (ANI) to the type genome, which has been assigned the MAG ID CHK152-2994 and which is available via NCBI BioSample SAMN15817221. This is a new name for the alphanumeric GTDB species sp900551915. The GC content of the type genome is 37.15% and the genome length is 1.9 Mbp.
**Description of *Candidatus* Scatocola gen. nov.**
*Candidatus* Scatocola (Sca.to’co.la. Gr. neut. n. *skor, skatos* dung; L. suff. *-cola* inhabitant of; N.L. fem. n. *Scatocola* a microbe associated with faeces)
A bacterial genus identified by metagenomic analyses. The genus includes all bacteria with genomes that show ≥60% average amino acid identity (AAI) to the type genome from the type species *Candidatus* Scatocola faecipullorum. This is a name for the alphanumeric GTDB genus CAG-495. This genus has been assigned by GTDB-Tk v1.3.0 working on GTDB Release 05-RS95 (Chaumeil et al., 2019; Parks et al., 2020) to the order *RF32* and to the family *CAG-239*.
**Description of *Candidatus* Scatocola faecigallinarum sp. nov.**
*Candidatus* Scatocola faecigallinarum (fae.ci.gal.li.na’rum. L. fem. n. *faex, faecis* excrement; L. fem. n. *gallina* hen; N.L. gen. n. *faecigallinarum* of hen faeces)
A bacterial species identified by metagenomic analyses. This species includes all bacteria with genomes that show ≥95% average nucleotide identity (ANI) to the type genome, which has been assigned the MAG ID 2846 and which is available via NCBI BioSample SAMN15817209. This is a new name for the alphanumeric GTDB species sp000436375. The GC content of the type genome is 49.31% and the genome length is 1.7 Mbp.
**Description of *Candidatus* Scatocola faecipullorum sp. nov.**
*Candidatus* Scatocola faecipullorum (fae.ci.pul.lo’rum. L. fem. n. *faex, faecis* excrement; L. masc. n. *pullus* a young chicken; N.L. gen. n. *faecipullorum* of young chicken faeces)
A bacterial species identified by metagenomic analyses. This species includes all bacteria with genomes that show ≥95% average nucleotide identity (ANI) to the type genome, which has been assigned the MAG ID ChiW3-316 and which is available via NCBI BioSample SAMN15817201. This is a new name for the alphanumeric GTDB species sp001917125. The GC content of the type genome is 47.24% and the genome length is 1.7 Mbp.
**Description of *Candidatus* Scatomonas gen. nov.**
*Candidatus* Scatomonas (Sca.to.mo’nas. Gr. neut. n. *skor, skatos* dung; L. fem. n. *monas* a monad; N.L. fem. n. *Scatomonas* a microbe associated with the intestines)
A bacterial genus identified by metagenomic analyses. The genus includes all bacteria with genomes that show ≥60% average amino acid identity (AAI) to the type genome from the type species *Candidatus* Scatomonas merdigallinarum. This is a name for the alphanumeric GTDB genus OF09-33XD. This genus has been assigned by GTDB-Tk v1.3.0 working on GTDB Release 05-RS95 (Chaumeil et al., 2019; Parks et al., 2020) to the order *Lachnospirales* and to the family *Lachnospiraceae*.
**Description of *Candidatus* Scatomonas merdavium sp. nov.**
*Candidatus* Scatomonas merdavium (merd.a’vi.um. L. fem. n. *merda* faeces; L. fem. n. *avis* bird; N.L. gen. n. *merdavium* of bird faeces)
A bacterial species identified by metagenomic analyses. This species includes all bacteria with genomes that show ≥95% average nucleotide identity (ANI) to the type genome, which has been assigned the MAG ID ChiSjej5B23-9500 and which is available via NCBI BioSample SAMN15817033. The GC content of the type genome is 53.14% and the genome length is 2.3 Mbp.
**Description of *Candidatus* Scatomonas merdigallinarum sp. nov.**
*Candidatus* Scatomonas merdigallinarum (mer.di.gal.li.na’rum. L. fem. n. *merda* faeces; L. fem. n. *gallina* hen; N.L. gen. n. *merdigallinarum* of hen faeces)
A bacterial species identified by metagenomic analyses. This species includes all bacteria with genomes that show ≥95% average nucleotide identity (ANI) to the type genome, which has been assigned the MAG ID CHK191-20366 and which is available via NCBI BioSample SAMN15817035. The GC content of the type genome is 53.29% and the genome length is 2.3 Mbp.
**Description of *Candidatus* Scatomonas pullistercoris sp. nov.**
*Candidatus* Scatomonas pullistercoris (pul.li.ster’co.ris. L. masc. n. *pullus* a young chicken; L. neut. n. *stercus* dung; N.L. gen. n. *pullistercoris* of young chicken faeces)
A bacterial species identified by metagenomic analyses. This species includes all bacteria with genomes that show ≥95% average nucleotide identity (ANI) to the type genome, which has been assigned the MAG ID CHK188-20938 and which is available via NCBI BioSample SAMN15817052. The GC content of the type genome is 53.16% and the genome length is 2.3 Mbp.

**Table:** 

**Description of *Candidatus* Scatomorpha gen. nov.**
*Candidatus* Scatomorpha (Sca.to.mor’pha. Gr. neut. n. *skor, skatos* dung; Gr. fem. n. *morphe* a form, shape; N.L. fem. n. *Scatomorpha* a microbe associated with faeces)
A bacterial genus identified by metagenomic analyses. The genus includes all bacteria with genomes that show ≥60% average amino acid identity (AAI) to the type genome from the type species *Candidatus* Scatomorpha merdavium. This is a name for the alphanumeric GTDB genus UBA5446. This genus has been assigned by GTDB-Tk v1.3.0 working on GTDB Release 05-RS95 (Chaumeil et al., 2019; Parks et al., 2020) to the order *Oscillospirales* and to the family *Oscillospiraceae*.
**Description of *Candidatus* Scatomorpha gallistercoris sp. nov.**
*Candidatus* Scatomorpha gallistercoris (gal.li.ster’co.ris. L. masc. n *gallus* chicken; L. neut. n. *stercus* dung; N.L. gen. n. *gallistercoris* of chicken faeces)
A bacterial species identified by metagenomic analyses. This species includes all bacteria with genomes that show ≥95% average nucleotide identity (ANI) to the type genome, which has been assigned the MAG ID ChiHjej12B11-5383 and which is available via NCBI BioSample SAMN15817213. This is a new name for the alphanumeric GTDB species sp900544765. The GC content of the type genome is 60.13% and the genome length is 2.4 Mbp.
**Description of *Candidatus* Scatomorpha intestinavium sp. nov.**
*Candidatus* Scatomorpha intestinavium (in.tes.tin.a’vi.um. L. neut. n. *intestinum* gut; L. fem. n. *avis* bird; N.L. gen. n. *intestinavium* of the gut of birds)
A bacterial species identified by metagenomic analyses. This species includes all bacteria with genomes that show ≥95% average nucleotide identity (ANI) to the type genome, which has been assigned the MAG ID ChiBcolR7-354 and which is available via NCBI BioSample SAMN15817058. The GC content of the type genome is 62.47% and the genome length is 2.0 Mbp.
**Description of *Candidatus* Scatomorpha intestinigallinarum sp. nov.**
*Candidatus* Scatomorpha intestinigallinarum (in.tes.ti.ni.gal.li.na’rum. L. neut. n. *intestinum* gut; L. fem. n. *gallina* hen; N.L. gen. n. *intestinigallinarum* of the gut of the hens)
A bacterial species identified by metagenomic analyses. This species includes all bacteria with genomes that show ≥95% average nucleotide identity (ANI) to the type genome, which has been assigned the MAG ID ChiGjej3B3-7149 and which is available via NCBI BioSample SAMN15817216. This is a new name for the alphanumeric GTDB species sp900544295. The GC content of the type genome is 63.01% and the genome length is 2.3 Mbp.
**Description of *Candidatus* Scatomorpha intestinipullorum sp. nov.**
*Candidatus* Scatomorpha intestinipullorum (in.tes.ti.ni.pul.lo’rum. L. neut. n. *intestinum* gut; L. masc. n. *pullus* a young chicken; N.L. gen. n. *intestinipullorum* of the gut of young chickens)
A bacterial species identified by metagenomic analyses. This species includes all bacteria with genomes that show ≥95% average nucleotide identity (ANI) to the type genome, which has been assigned the MAG ID CHK1-1240 and which is available via NCBI BioSample SAMN15817022. The GC content of the type genome is 61.56% and the genome length is 2.5 Mbp.
**Description of *Candidatus* Scatomorpha merdavium sp. nov.**
*Candidatus* Scatomorpha merdavium (merd.a’vi.um. L. fem. n. *merda* faeces; L. fem. n. *avis* bird; N.L. gen. n. *merdavium* of bird faeces)
A bacterial species identified by metagenomic analyses. This species includes all bacteria with genomes that show ≥95% average nucleotide identity (ANI) to the type genome, which has been assigned the MAG ID ChiSxjej3B15-13231 and which is available via NCBI BioSample SAMN15817220. This is a new name for the alphanumeric GTDB species sp004553625. The GC content of the type genome is 61.38% and the genome length is 2.3 Mbp.
**Description of *Candidatus* Scatomorpha merdigallinarum sp. nov.**
*Candidatus* Scatomorpha merdigallinarum (mer.di.gal.li.na’rum. L. fem. n. *merda* faeces; L. fem. n. *gallina* hen; N.L. gen. n. *merdigallinarum* of hen faeces)
A bacterial species identified by metagenomic analyses. This species includes all bacteria with genomes that show ≥95% average nucleotide identity (ANI) to the type genome, which has been assigned the MAG ID CHK187-5235 and which is available via NCBI BioSample SAMN15817028. The GC content of the type genome is 58.23% and the genome length is 2.3 Mbp.
**Description of *Candidatus* Scatomorpha merdipullorum sp. nov.**
*Candidatus* Scatomorpha merdipullorum (mer.di.pul.lo’rum. L. fem. n. *merda* faeces; L. masc. n. *pullus* a young chicken; N.L. gen. n. *merdipullorum* of the faeces of young chickens)
A bacterial species identified by metagenomic analyses. This species includes all bacteria with genomes that show ≥95% average nucleotide identity (ANI) to the type genome, which has been assigned the MAG ID ChiHjej10B9-9673 and which is available via NCBI BioSample SAMN15817099. The GC content of the type genome is 64.45% and the genome length is 1.8 Mbp.

**Table:** 

**Description of *Candidatus* Scatomorpha pullicola sp. nov.**
*Candidatus* Scatomorpha pullicola (pul.li’co.la. L. masc. n. *pullus* a young chicken; L. suff. *-cola* inhabitant of; N.L. n. *pullicola* an inhabitant of young chickens)
A bacterial species identified by metagenomic analyses. This species includes all bacteria with genomes that show ≥95% average nucleotide identity (ANI) to the type genome, which has been assigned the MAG ID ChiSjej5B23-7677 and which is available via NCBI BioSample SAMN15817223. This is a new name for the alphanumeric GTDB species sp900543085. The GC content of the type genome is 62.30% and the genome length is 2.1 Mbp.
**Description of *Candidatus* Scatomorpha pullistercoris sp. nov.**
*Candidatus* Scatomorpha pullistercoris (pul.li.ster’co.ris. L. masc. n. *pullus* a young chicken; L. neut. n. *stercus* dung; N.L. gen. n. *pullistercoris* of young chicken faeces)
A bacterial species identified by metagenomic analyses. This species includes all bacteria with genomes that show ≥95% average nucleotide identity (ANI) to the type genome, which has been assigned the MAG ID ChiHecec3B27-6122 and which is available via NCBI BioSample SAMN15817222. This is a new name for the alphanumeric GTDB species sp900546615. The GC content of the type genome is 61.20% and the genome length is 2.2 Mbp.
**Description of *Candidatus* Scatomorpha stercoravium sp. nov.**
*Candidatus* Scatomorpha stercoravium (ster.cor.a’vi.um. L. neut. n. *stercus* dung; L. fem. n. *avis* bird; N.L. gen. n. *stercoravium* of bird faeces)
A bacterial species identified by metagenomic analyses. This species includes all bacteria with genomes that show ≥95% average nucleotide identity (ANI) to the type genome, which has been assigned the MAG ID ChiHecec3B27-8609 and which is available via NCBI BioSample SAMN15817107. The GC content of the type genome is 64.50% and the genome length is 2.0 Mbp.
**Description of *Candidatus* Scatomorpha stercorigallinarum sp. nov.**
*Candidatus* Scatomorpha stercorigallinarum (ster.co.ri.gal.li.na’rum. L. neut. n. *stercus* dung; L. fem. n. *gallina* hen; N.L. gen. n. *stercorigallinarum* of hen faeces)
A bacterial species identified by metagenomic analyses. This species includes all bacteria with genomes that show ≥95% average nucleotide identity (ANI) to the type genome, which has been assigned the MAG ID ChiHjej9B8-2268 and which is available via NCBI BioSample SAMN15817162. The GC content of the type genome is 64.82% and the genome length is 2.0 Mbp.
**Description of *Candidatus* Scatoplasma gen. nov.**
*Candidatus* Scatoplasma (Sca.to.plas’ma. Gr. neut. n. *skor, skatos* dung; Gr. neut. n. *plasma* a form; N.L. neut. n. *Scatoplasma* a microbe associated with faeces)
A bacterial genus identified by metagenomic analyses. The genus includes all bacteria with genomes that show ≥60% average amino acid identity (AAI) to the type genome from the type species *Candidatus* Scatoplasma merdavium. This is a name for the alphanumeric GTDB genus UBA6879. This genus has been assigned by GTDB-Tk v1.3.0 working on GTDB Release 05-RS95 (Chaumeil et al., 2019; Parks et al., 2020) to the order *RFN20* and to the family *CAG-288*.
**Description of *Candidatus* Scatoplasma merdavium sp. nov.**
*Candidatus* Scatoplasma merdavium (merd.a’vi.um. L. fem. n. *merda* faeces; L. fem. n. *avis* bird; N.L. gen. n. *merdavium* of bird faeces)
A bacterial species identified by metagenomic analyses. This species includes all bacteria with genomes that show ≥95% average nucleotide identity (ANI) to the type genome, which has been assigned the MAG ID 1748 and which is available via NCBI BioSample SAMN15817159. The GC content of the type genome is 36.99% and the genome length is 1.0 Mbp.
**Description of *Candidatus* Scatosoma gen. nov.**
*Candidatus* Scatosoma (Sca.to.so’ma. Gr. neut. n. *skor, skatos* dung; Gr. neut. n. *soma* a body; N.L. neut. n. *Scatosoma* a microbe associated with the intestines)
A bacterial genus identified by metagenomic analyses. The genus includes all bacteria with genomes that show ≥60% average amino acid identity (AAI) to the type genome from the type species *Candidatus* Scatosoma pullicola. This is a name for the alphanumeric GTDB genus QALS01. This genus has been assigned by GTDB-Tk v1.3.0 working on GTDB Release 05-RS95 (Chaumeil et al., 2019; Parks et al., 2020) to the order *Christensenellales* and to the family *Borkfalkiaceae*.
**Description of *Candidatus* Scatosoma pullicola sp. nov.**
*Candidatus* Scatosoma pullicola (pul.li’co.la. L. masc. n. *pullus* a young chicken; L. suff. *-cola* inhabitant of; N.L. n. *pullicola* an inhabitant of young chickens)
A bacterial species identified by metagenomic analyses. This species includes all bacteria with genomes that show ≥95% average nucleotide identity (ANI) to the type genome, which has been assigned the MAG ID CHK183-20193 and which is available via NCBI BioSample SAMN15817013. The GC content of the type genome is 57.19% and the genome length is 2.0 Mbp.

**Table:** 

**Description of *Candidatus* Scatosoma pullistercoris sp. nov.**
*Candidatus* Scatosoma pullistercoris (pul.li.ster’co.ris. L. masc. n. *pullus* a young chicken; L. neut. n. *stercus* dung; N.L. gen. n. *pullistercoris* of young chicken faeces)
A bacterial species identified by metagenomic analyses. This species includes all bacteria with genomes that show ≥95% average nucleotide identity (ANI) to the type genome, which has been assigned the MAG ID 11687 and which is available via NCBI BioSample SAMN15817138. The GC content of the type genome is 55.35% and the genome length is 1.5 Mbp.
**Description of *Candidatus* Scatousia gen. nov.**
*Candidatus* Scatousia (Scat.ou’si.a. Gr. neut. n. *skor, skatos* dung; Gr. fem. n. *ousia* an essence; N.L. fem. n. *Scatousia* a microbe associated with faeces)
A bacterial genus identified by metagenomic analyses. The genus includes all bacteria with genomes that show ≥60% average amino acid identity (AAI) to the type genome from the type species *Candidatus* Scatousia excrementipullorum. This is a name for the alphanumeric GTDB genus CAG-484. This genus has been assigned by GTDB-Tk v1.3.0 working on GTDB Release 05-RS95 (Chaumeil et al., 2019; Parks et al., 2020) to the order *Gastranaerophilales* and to the family *Gastranaerophilaceae*.
**Description of *Candidatus* Scatousia excrementigallinarum sp. nov.**
*Candidatus* Scatousia excrementigallinarum (ex.cre.men.ti.gal.li.na’rum. L. neut. n. *excrementum* excrement; L. fem. n. *gallina* hen; N.L. gen. n. *excrementigallinarum* of hen excrement)
A bacterial species identified by metagenomic analyses. This species includes all bacteria with genomes that show ≥95% average nucleotide identity (ANI) to the type genome, which has been assigned the MAG ID 6276 and which is available via NCBI BioSample SAMN15817091. The GC content of the type genome is 36.46% and the genome length is 2.8 Mbp.
**Description of *Candidatus* Scatousia excrementipullorum sp. nov.**
*Candidatus* Scatousia excrementipullorum (ex.cre.men.ti.pul.lo’rum. L. neut. n. *excrementum* excrement; L. masc. n. *pullus* a young chicken; N.L. gen. n. *excrementipullorum* of young chicken excrement)
A bacterial species identified by metagenomic analyses. This species includes all bacteria with genomes that show ≥95% average nucleotide identity (ANI) to the type genome, which has been assigned the MAG ID 10192 and which is available via NCBI BioSample SAMN15817157. The GC content of the type genome is 36.06% and the genome length is 1.8 Mbp.
**Description of *Candidatus* Scatovicinus gen. nov.**
*Candidatus* Scatovicinus (Sca.to.vi.ci’nus. Gr. neut. n. *skor, skatos* dung; L. masc. n. *vicinus* a neighbour; N.L. masc. n. *Scatovicinus* a microbe associated with faeces)
A bacterial genus identified by metagenomic analyses. The genus includes all bacteria with genomes that show ≥60% average amino acid identity (AAI) to the type genome from the type species *Candidatus* Scatovicinus merdipullorum. This is a name for the alphanumeric GTDB genus UMGS403. This genus has been assigned by GTDB-Tk v1.3.0 working on GTDB Release 05-RS95 (Chaumeil et al., 2019; Parks et al., 2020) to the order *Oscillospirales* and to the family *Acutalibacteraceae*.
**Description of *Candidatus* Scatovicinus merdipullorum sp. nov.**
*Candidatus* Scatovicinus merdipullorum (mer.di.pul.lo’rum. L. fem. n. *merda* faeces; L. masc. n. *pullus* a young chicken; N.L. gen. n. *merdipullorum* of the faeces of young chickens)
A bacterial species identified by metagenomic analyses. This species includes all bacteria with genomes that show ≥95% average nucleotide identity (ANI) to the type genome, which has been assigned the MAG ID CHK181-9830 and which is available via NCBI BioSample SAMN15817214. This is a new name for the alphanumeric GTDB species sp900541565. The GC content of the type genome is 46.80% and the genome length is 1.9 Mbp.
**Description of *Candidatus* Scatovivens gen. nov.**
*Candidatus* Scatovivens (Sca.to.vi’vens. Gr. neut. n. *skor, skatos* dung; N.L. pres. part. *vivens* living; N.L. fem. n. *Scatovivens* a microbe associated with faeces)
A bacterial genus identified by metagenomic analyses. The genus includes all bacteria with genomes that show ≥60% average amino acid identity (AAI) to the type genome from the type species *Candidatus* Scatovivens faecipullorum. This is a name for the alphanumeric GTDB genus UBA7001. This genus has been assigned by GTDB-Tk v1.3.0 working on GTDB Release 05-RS95 (Chaumeil et al., 2019; Parks et al., 2020) to the order *TANB77* and to the family *CAG-508*.

**Table:** 

**Description of *Candidatus* Scatovivens faecipullorum sp. nov.**
*Candidatus* Scatovivens faecipullorum (fae.ci.pul.lo’rum. L. fem. n. *faex, faecis* excrement; L. masc. n. *pullus* a young chicken; N.L. gen. n. *faecipullorum* of young chicken faeces)
A bacterial species identified by metagenomic analyses. This species includes all bacteria with genomes that show ≥95% average nucleotide identity (ANI) to the type genome, which has been assigned the MAG ID ChiSxjej5B17-16517 and which is available via NCBI BioSample SAMN15817189. This is a new name for the alphanumeric GTDB species sp900553685. The GC content of the type genome is 25.63% and the genome length is 1.9 Mbp.
**Description of *Candidatus* Scybalenecus merdavium sp. nov.**
*Candidatus* Scybalenecus merdavium (merd.a’vi.um. L. fem. n. *merda* faeces; L. fem. n. *avis* bird; N.L. gen. n. *merdavium* of bird faeces)
A bacterial species identified by metagenomic analyses. This species includes all bacteria with genomes that show ≥95% average nucleotide identity (ANI) to the type genome, which has been assigned the MAG ID CHK176-6737 and which is available via NCBI BioSample SAMN15817203. This is a new name for the alphanumeric GTDB species sp900546735. The GC content of the type genome is 52.55% and the genome length is 1.8 Mbp.
**Description of *Candidatus* Scybalocola faecavium sp. nov.**
*Candidatus* Scybalocola faecavium (faec.a’vi.um. L. fem. n. *faex, faecis* excrement; L. fem. n. *avis* bird; N.L. gen. n. *faecavium* of bird faeces)
A bacterial species identified by metagenomic analyses. This species includes all bacteria with genomes that show ≥95% average nucleotide identity (ANI) to the type genome, which has been assigned the MAG ID CHK196-3395 and which is available via NCBI BioSample SAMN15817004. The GC content of the type genome is 45.55% and the genome length is 3.6 Mbp.
**Description of *Candidatus* Scybalocola faecigallinarum sp. nov.**
*Candidatus* Scybalocola faecigallinarum (fae.ci.gal.li.na’rum. L. fem. n. *faex, faecis* excrement; L. fem. n. *gallina* hen; N.L. gen. n. *faecigallinarum* of hen faeces)
A bacterial species identified by metagenomic analyses. This species includes all bacteria with genomes that show ≥95% average nucleotide identity (ANI) to the type genome, which has been assigned the MAG ID CHK178-757 and which is available via NCBI BioSample SAMN15817027. The GC content of the type genome is 46.78% and the genome length is 3.5 Mbp.
**Description of *Candidatus* Scybalocola faecipullorum sp. nov.**
*Candidatus* Scybalocola faecipullorum (fae.ci.pul.lo’rum. L. fem. n. *faex, faecis* excrement; L. masc. n. *pullus* a young chicken; N.L. gen. n. *faecipullorum* of young chicken faeces)
A bacterial species identified by metagenomic analyses. This species includes all bacteria with genomes that show ≥95% average nucleotide identity (ANI) to the type genome, which has been assigned the MAG ID CHK194-7924 and which is available via NCBI BioSample SAMN15817034. The GC content of the type genome is 44.36% and the genome length is 2.5 Mbp.
**Description of *Candidatus* Scybalomonas excrementavium sp. nov.**
*Candidatus* Scybalomonas excrementavium (ex.cre.ment.a’vi.um. L. neut. n. *excrementum* excrement; L. fem. n. *avis* bird; N.L. gen. n. *excrementavium* of bird excrement)
A bacterial species identified by metagenomic analyses. This species includes all bacteria with genomes that show ≥95% average nucleotide identity (ANI) to the type genome, which has been assigned the MAG ID E3-2379 and which is available via NCBI BioSample SAMN15817094. The GC content of the type genome is 32.78% and the genome length is 2.5 Mbp.
**Description of *Candidatus* Scybalomonas excrementigallinarum sp. nov.**
*Candidatus* Scybalomonas excrementigallinarum (ex.cre.men.ti.gal.li.na’rum. L. neut. n. *excrementum* excrement; L. fem. n. *gallina* hen; N.L. gen. n. *excrementigallinarum* of hen excrement)
A bacterial species identified by metagenomic analyses. This species includes all bacteria with genomes that show ≥95% average nucleotide identity (ANI) to the type genome, which has been assigned the MAG ID 3201 and which is available via NCBI BioSample SAMN15817095. The GC content of the type genome is 34.32% and the genome length is 3.2 Mbp.
**Description of *Candidatus* Scybalosoma faecavium sp. nov.**
*Candidatus* Scybalosoma faecavium (faec.a’vi.um. L. fem. n. *faex, faecis* excrement; L. fem. n. *avis* bird; N.L. gen. n. *faecavium* of bird faeces)
A bacterial species identified by metagenomic analyses. This species includes all bacteria with genomes that show ≥95% average nucleotide identity (ANI) to the type genome, which has been assigned the MAG ID ChiSxjej1B13-2233 and which is available via NCBI BioSample SAMN15817224. This is a new name for the alphanumeric GTDB species sp900545085. The GC content of the type genome is 47.85% and the genome length is 1.6 Mbp.

**Table:** 

**Description of *Candidatus* Scybalousia intestinigallinarum sp. nov.**
*Candidatus* Scybalousia intestinigallinarum (in.tes.ti.ni.gal.li.na’rum. L. neut. n. *intestinum* gut; L. fem. n. *gallina* hen; N.L. gen. n. *intestinigallinarum* of the gut of the hens)
A bacterial species identified by metagenomic analyses. This species includes all bacteria with genomes that show ≥95% average nucleotide identity (ANI) to the type genome, which has been assigned the MAG ID CHK193-12526 and which is available via NCBI BioSample SAMN15817037. The GC content of the type genome is 31.54% and the genome length is 1.5 Mbp.
**Description of *Candidatus* Scybalenecus gen. nov.**
*Candidatus* Scybalenecus (Scy.bal.en.e’cus. Gr. neut. n. *skybalon* dung; Gr. masc. *enoikos* inhabitant; N.L. masc. n. *Scybalenecus* a microbe associated with faeces)
A bacterial genus identified by metagenomic analyses. The genus includes all bacteria with genomes that show ≥60% average amino acid identity (AAI) to the type genome from the type species *Candidatus* Scybalenecus merdavium. This is a name for the alphanumeric GTDB genus UMGS905. This genus has been assigned by GTDB-Tk v1.3.0 working on GTDB Release 05-RS95 (Chaumeil et al., 2019; Parks et al., 2020) to the order *Oscillospirales* and to the family *Acutalibacteraceae*.
**Description of *Candidatus* Scybalocola gen. nov.**
*Candidatus* Scybalocola (Scy.ba.lo’co.la. Gr. neut. n. *skybalon* dung; L. suff. *-cola* inhabitant of; N.L. fem. n. *Scybalocola* a microbe associated with faeces)
A bacterial genus identified by metagenomic analyses. The genus includes all bacteria with genomes that show ≥60% average amino acid identity (AAI) to the type genome from the type species *Candidatus* Scybalocola faecigallinarum. This is a name for the alphanumeric GTDB genus UBA7096. This genus has been assigned by GTDB-Tk v1.3.0 working on GTDB Release 05-RS95 (Chaumeil et al., 2019; Parks et al., 2020) to the order *Lachnospirales* and to the family *Lachnospiraceae*.
**Description of *Candidatus* Scybalomonas gen. nov.**
*Candidatus* Scybalomonas (Scy.ba.lo.mo’nas. Gr. neut. n. *skybalon* dung; L. fem. n. *monas* a monad; N.L. fem. n. *Scybalomonas* a microbe associated with faeces)
A bacterial genus identified by metagenomic analyses. The genus includes all bacteria with genomes that show ≥60% average amino acid identity (AAI) to the type genome from the type species *Candidatus* Scybalomonas excrementigallinarum. This is a name for the alphanumeric GTDB genus UMGS680. This genus has been assigned by GTDB-Tk v1.3.0 working on GTDB Release 05-RS95 (Chaumeil et al., 2019; Parks et al., 2020) to the order *Lachnospirales* and to the family *Lachnospiraceae*.
**Description of *Candidatus* Scybalosoma gen. nov.**
*Candidatus* Scybalosoma (Scy.ba.lo.so’ma. Gr. neut. n. *skybalon* dung; Gr. neut. n. *soma* a body; N.L. neut. n. *Scybalosoma* a microbe associated with faeces)
A bacterial genus identified by metagenomic analyses. The genus includes all bacteria with genomes that show ≥60% average amino acid identity (AAI) to the type genome from the type species *Candidatus* Scybalosoma faecavium. This is a name for the alphanumeric GTDB genus UMGS743. This genus has been assigned by GTDB-Tk v1.3.0 working on GTDB Release 05-RS95 (Chaumeil et al., 2019; Parks et al., 2020) to the order *Christensenellales* and to the family *Christensenellaceae*.
**Description of *Candidatus* Scybalousia gen. nov.**
*Candidatus* Scybalousia (Scy.bal.ou’si.a. Gr. neut. n. *skybalon* dung; Gr. fem. n. *ousia* an essence; N.L. fem. n. *Scybalousia* a microbe associated with faeces)
A bacterial genus identified by metagenomic analyses. The genus includes all bacteria with genomes that show ≥60% average amino acid identity (AAI) to the type genome from the type species *Candidatus* Scybalousia intestinigallinarum. This is a name for the alphanumeric GTDB genus UBA7057. This genus has been assigned by GTDB-Tk v1.3.0 working on GTDB Release 05-RS95 (Chaumeil et al., 2019; Parks et al., 2020) to the order *RF39* and to the family *CAG-611*.
**Description of *Candidatus* Sellimonas avistercoris sp. nov.**
*Candidatus* Sellimonas avistercoris (a.vi.ster’co.ris. L. fem. n. *avis* bird; L. neut. n. *stercus* dung; N.L. gen. n. *avistercoris* of bird faeces)
A bacterial species identified by metagenomic analyses. This species includes all bacteria with genomes that show ≥95% average nucleotide identity (ANI) to the type genome, which has been assigned the MAG ID ChiBcec13-3606 and which is available via NCBI BioSample SAMN15816877. This is a new name for the alphanumeric GTDB species sp002161525. The GC content of the type genome is 46.79% and the genome length is 2.6 Mbp.
**Description of *Candidatus* Sphingobacterium stercorigallinarum sp. nov.**
*Candidatus* Sphingobacterium stercorigallinarum (ster.co.ri.gal.li.na’rum. L. neut. n. *stercus* dung; L. fem. n. *gallina* hen; N.L. gen. n. *stercorigallinarum* of hen faeces)
A bacterial species identified by metagenomic analyses. This species includes all bacteria with genomes that show ≥95% average nucleotide identity (ANI) to the type genome, which has been assigned the MAG ID CHK174-1108 and which is available via NCBI BioSample SAMN15816705. The GC content of the type genome is 44.57% and the genome length is 2.9 Mbp.

**Table:** 

**Description of *Candidatus* Sphingobacterium stercoripullorum sp. nov.**
*Candidatus* Sphingobacterium stercoripullorum (ster.co.ri.pul.lo’rum. L. neut. n. *stercus* dung; L. masc. n. *pullus* a young chicken; N.L. gen. n. *stercoripullorum* of the faceces of young chickens)
A bacterial species identified by metagenomic analyses. This species includes all bacteria with genomes that show ≥95% average nucleotide identity (ANI) to the type genome, which has been assigned the MAG ID 1719 and which is available via NCBI BioSample SAMN15816733. The GC content of the type genome is 39.36% and the genome length is 2.7 Mbp.
**Description of *Candidatus* Sphingomonas excrementigallinarum sp. nov.**
*Candidatus* Sphingomonas excrementigallinarum (ex.cre.men.ti.gal.li.na’rum. L. neut. n. *excrementum* excrement; L. fem. n. *gallina* hen; N.L. gen. n. *excrementigallinarum* of hen excrement)
A bacterial species identified by metagenomic analyses. This species includes all bacteria with genomes that show ≥95% average nucleotide identity (ANI) to the type genome, which has been assigned the MAG ID 1562 and which is available via NCBI BioSample SAMN15816779. The GC content of the type genome is 66.49% and the genome length is 3.7 Mbp.
**Description of *Candidatus* Spyradenecus gen. nov.**
*Candidatus* Spyradenecus (Spy.rad.en.e’cus. Gr. fem. n. *spyras* ball of dung; Gr. masc. *enoikos* inhabitant; N.L. masc. n. *Spyradenecus* a microbe associated with the intestines)
A bacterial genus identified by metagenomic analyses. The genus includes all bacteria with genomes that show ≥60% average amino acid identity (AAI) to the type genome from the type species *Candidatus* Spyradenecus faecavium. This is a name for the alphanumeric GTDB genus W1P29-020. This genus has been assigned by GTDB-Tk v1.3.0 working on GTDB Release 05-RS95 (Chaumeil et al., 2019; Parks et al., 2020) to the order *RFP12* and to the family *W1P29-020*.
**Description of *Candidatus* Spyradenecus faecavium sp. nov.**
*Candidatus* Spyradenecus faecavium (faec.a’vi.um. L. fem. n. *faex, faecis* excrement; L. fem. n. *avis* bird; N.L. gen. n. *faecavium* of bird faeces)
A bacterial species identified by metagenomic analyses. This species includes all bacteria with genomes that show ≥95% average nucleotide identity (ANI) to the type genome, which has been assigned the MAG ID 35461 and which is available via NCBI BioSample SAMN15817160. The GC content of the type genome is 68.44% and the genome length is 1.8 Mbp.
**Description of *Candidatus* Spyradocola gen. nov.**
*Candidatus* Spyradocola (Spy.ra.do’co.la. Gr. fem. n. *spyras* ball of dung; L. suff. *-cola* inhabitant of; N.L. fem. n. *Spyradocola* a microbe associated with the intestines)
A bacterial genus identified by metagenomic analyses. The genus includes all bacteria with genomes that show ≥60% average amino acid identity (AAI) to the type genome from the type species *Candidatus* Spyradocola merdavium. This is a name for the alphanumeric GTDB genus UBA7102. This genus has been assigned by GTDB-Tk v1.3.0 working on GTDB Release 05-RS95 (Chaumeil et al., 2019; Parks et al., 2020) to the order *Christensenellales* and to the family *UBA1750*.
**Description of *Candidatus* Spyradocola merdavium sp. nov.**
*Candidatus* Spyradocola merdavium (merd.a’vi.um. L. fem. n. *merda* faeces; L. fem. n. *avis* bird; N.L. gen. n. *merdavium* of bird faeces)
A bacterial species identified by metagenomic analyses. This species includes all bacteria with genomes that show ≥95% average nucleotide identity (ANI) to the type genome, which has been assigned the MAG ID CHK191-18038 and which is available via NCBI BioSample SAMN15817024. The GC content of the type genome is 66.20% and the genome length is 2.6 Mbp.
**Description of *Candidatus* Spyradomonas gen. nov.**
*Candidatus* Spyradomonas (Spy.ra.do.mo’nas. Gr. fem. n. *spyras* ball of dung; L. fem. n. *monas* a monad; N.L. fem. n. *Spyradomonas* a microbe associated with faeces)
A bacterial genus identified by metagenomic analyses. The genus includes all bacteria with genomes that show ≥60% average amino acid identity (AAI) to the type genome from the type species *Candidatus* Spyradomonas excrementavium. This is a name for the alphanumeric GTDB genus UMGS951. This genus has been assigned by GTDB-Tk v1.3.0 working on GTDB Release 05-RS95 (Chaumeil et al., 2019; Parks et al., 2020) to the order *Gastranaerophilales* and to the family *Gastranaerophilaceae*.
**Description of *Candidatus* Spyradomonas excrementavium sp. nov.**
*Candidatus* Spyradomonas excrementavium (ex.cre.ment.a’vi.um. L. neut. n. *excrementum* excrement; L. fem. n. *avis* bird; N.L. gen. n. *excrementavium* of bird excrement)
A bacterial species identified by metagenomic analyses. This species includes all bacteria with genomes that show ≥95% average nucleotide identity (ANI) to the type genome, which has been assigned the MAG ID CHK149-2741 and which is available via NCBI BioSample SAMN15817232. This is a new name for the alphanumeric GTDB species sp900547155. The GC content of the type genome is 41.54% and the genome length is 2.0 Mbp.

**Table:** 

**Description of *Candidatus* Spyradosoma gen. nov.**
*Candidatus* Spyradosoma (Spy.ra.do.so’ma. Gr. fem. n. *spyras* ball of dung; Gr. neut. n. *soma* a body; N.L. neut. n. *Spyradosoma* a microbe associated with faeces)
A bacterial genus identified by metagenomic analyses. The genus includes all bacteria with genomes that show ≥60% average amino acid identity (AAI) to the type genome from the type species *Candidatus* Spyradosoma merdigallinarum. This is a name for the alphanumeric GTDB genus W0P29-029. This genus has been assigned by GTDB-Tk v1.3.0 working on GTDB Release 05-RS95 (Chaumeil et al., 2019; Parks et al., 2020) to the order *Opitutales* and to the family *UBA953*.
**Description of *Candidatus* Spyradosoma merdigallinarum sp. nov.**
*Candidatus* Spyradosoma merdigallinarum (mer.di.gal.li.na’rum. L. fem. n. *merda* faeces; L. fem. n. *gallina* hen; N.L. gen. n. *merdigallinarum* of hen faeces)
A bacterial species identified by metagenomic analyses. This species includes all bacteria with genomes that show ≥95% average nucleotide identity (ANI) to the type genome, which has been assigned the MAG ID 10669 and which is available via NCBI BioSample SAMN15817156. The GC content of the type genome is 62.43% and the genome length is 1.6 Mbp.
**Description of *Candidatus* Stackebrandtia excrementipullorum sp. nov.**
*Candidatus* Stackebrandtia excrementipullorum (ex.cre.men.ti.pul.lo’rum. L. neut. n. *excrementum* excrement; L. masc. n. *pullus* a young chicken; N.L. gen. n. *excrementipullorum* of young chicken excrement)
A bacterial species identified by metagenomic analyses. This species includes all bacteria with genomes that show ≥95% average nucleotide identity (ANI) to the type genome, which has been assigned the MAG ID ChiHjej8B7-33794 and which is available via NCBI BioSample SAMN15816761. The GC content of the type genome is 64.17% and the genome length is 4.1 Mbp.
**Description of *Candidatus* Stackebrandtia faecavium sp. nov.**
*Candidatus* Stackebrandtia faecavium (faec.a’vi.um. L. fem. n. *faex, faecis* excrement; L. fem. n. *avis* bird; N.L. gen. n. *faecavium* of bird faeces)
A bacterial species identified by metagenomic analyses. This species includes all bacteria with genomes that show ≥95% average nucleotide identity (ANI) to the type genome, which has been assigned the MAG ID ChiGjej4B4-770 and which is available via NCBI BioSample SAMN15816782. The GC content of the type genome is 62.84% and the genome length is 3.4 Mbp.
**Description of *Candidatus* Stercoripulliclostridium gen. nov.**
*Candidatus* Stercoripulliclostridium (Ster.co.ri.pul.li.clos.tri’di.um. L. neut. n. *stercus* dung; N.L. masc. n. *pullus* a young chicken; N.L. neut. n. *Clostridium* a genus name; N.L. neut. n. *Stercoripulliclostridium* a genus related to the genus *Clostridium* but distinct from it and found in poultry faeces)
A bacterial genus identified by metagenomic analyses. The genus includes all bacteria with genomes that show ≥60% average amino acid identity (AAI) to the type genome from the type species *Candidatus* Stercoripulliclostridium merdipullorum. This genus has been assigned by GTDB-Tk v1.3.0 working on GTDB Release 05-RS95 (Chaumeil et al., 2019; Parks et al., 2020) to the order *Christensenellales* and to the family *DTU072*.
**Description of *Candidatus* Stercoripulliclostridium merdigallinarum sp. nov.**
*Candidatus* Stercoripulliclostridium merdigallinarum (mer.di.gal.li.na’rum. L. fem. n. *merda* faeces; L. fem. n. *gallina* hen; N.L. gen. n. *merdigallinarum* of hen faeces)
A bacterial species identified by metagenomic analyses. This species includes all bacteria with genomes that show ≥95% average nucleotide identity (ANI) to the type genome, which has been assigned the MAG ID 18911 and which is available via NCBI BioSample SAMN15816986. The GC content of the type genome is 48.67% and the genome length is 1.5 Mbp.
**Description of *Candidatus* Stercoripulliclostridium merdipullorum sp. nov.**
*Candidatus* Stercoripulliclostridium merdipullorum (mer.di.pul.lo’rum. L. fem. n. *merda* faeces; L. masc. n. *pullus* a young chicken; N.L. gen. n. *merdipullorum* of the faeces of young chickens)
A bacterial species identified by metagenomic analyses. This species includes all bacteria with genomes that show ≥95% average nucleotide identity (ANI) to the type genome, which has been assigned the MAG ID 23406 and which is available via NCBI BioSample SAMN15816995. The GC content of the type genome is 53.33% and the genome length is 1.7 Mbp.
**Description of *Candidatus* Stercoripulliclostridium pullicola sp. nov.**
*Candidatus* Stercoripulliclostridium pullicola (pul.li’co.la. L. masc. n. *pullus* a young chicken; L. suff. *-cola* inhabitant of; N.L. n. *pullicola* an inhabitant of young chickens)
A bacterial species identified by metagenomic analyses. This species includes all bacteria with genomes that show ≥95% average nucleotide identity (ANI) to the type genome, which has been assigned the MAG ID 517 and which is available via NCBI BioSample SAMN15816997. The GC content of the type genome is 52.41% and the genome length is 1.6 Mbp.

**Table:** 

**Description of *Candidatus* Stercorousia gen. nov.**
*Candidatus* Stercorousia (Ster.cor.ou’si.a. L. neut. n. *stercus* dung; Gr. fem. n. *ousia* an essence; N.L. fem. n. *Stercorousia* a microbe associated with the intestines)
A bacterial genus identified by metagenomic analyses. The genus includes all bacteria with genomes that show ≥60% average amino acid identity (AAI) to the type genome from the type species *Candidatus* Stercorousia faecigallinarum. This is a name for the alphanumeric GTDB genus Zag1. This genus has been assigned by GTDB-Tk v1.3.0 working on GTDB Release 05-RS95 (Chaumeil et al., 2019; Parks et al., 2020) to the order *Gastranaerophilales* and to the family *Gastranaerophilaceae*.
**Description of *Candidatus* Stercorousia faecigallinarum sp. nov.**
*Candidatus* Stercorousia faecigallinarum (fae.ci.gal.li.na’rum. L. fem. n. *faex, faecis* excrement; L. fem. n. *gallina* hen; N.L. gen. n. *faecigallinarum* of hen faeces)
A bacterial species identified by metagenomic analyses. This species includes all bacteria with genomes that show ≥95% average nucleotide identity (ANI) to the type genome, which has been assigned the MAG ID CHK154-323 and which is available via NCBI BioSample SAMN15817192. This is a new name for the alphanumeric GTDB species sp000438175. The GC content of the type genome is 34.85% and the genome length is 2.1 Mbp.
**Description of *Candidatus* Streptococcus faecavium sp. nov.**
*Candidatus* Streptococcus faecavium (faec.a’vi.um. L. fem. n. *faex, faecis* excrement; L. fem. n. *avis* bird; N.L. gen. n. *faecavium* of bird faeces)
A bacterial species identified by metagenomic analyses. This species includes all bacteria with genomes that show ≥95% average nucleotide identity (ANI) to the type genome, which has been assigned the MAG ID ChiBcolR9-63 and which is available via NCBI BioSample SAMN15816845. This is a new name for the alphanumeric GTDB species sp002300045. The GC content of the type genome is 40.90% and the genome length is 1.4 Mbp.
**Description of *Candidatus* Sutterella merdavium sp. nov.**
*Candidatus* Sutterella merdavium (merd.a’vi.um. L. fem. n. *merda* faeces; L. fem. n. *avis* bird; N.L. gen. n. *merdavium* of bird faeces)
A bacterial species identified by metagenomic analyses. This species includes all bacteria with genomes that show ≥95% average nucleotide identity (ANI) to the type genome, which has been assigned the MAG ID ChiGjej6B6-11950 and which is available via NCBI BioSample SAMN15816882. This is a new name for the alphanumeric GTDB species sp900543805. The GC content of the type genome is 62.35% and the genome length is 2.1 Mbp.
**Description of *Candidatus* Tetragenococcus pullicola sp. nov.**
*Candidatus* Tetragenococcus pullicola (pul.li’co.la. L. masc. n. *pullus* a young chicken; L. suff. *-cola* inhabitant of; N.L. n. *pullicola* an inhabitant of young chickens)
A bacterial species identified by metagenomic analyses. This species includes all bacteria with genomes that show ≥95% average nucleotide identity (ANI) to the type genome, which has been assigned the MAG ID CHK175-10598 and which is available via NCBI BioSample SAMN15816709. The GC content of the type genome is 36.35% and the genome length is 2.6 Mbp.
**Description of *Candidatus* Tidjanibacter faecipullorum sp. nov.**
*Candidatus* Tidjanibacter faecipullorum (fae.ci.pul.lo’rum. L. fem. n. *faex, faecis* excrement; L. masc. n. *pullus* a young chicken; N.L. gen. n. *faecipullorum* of young chicken faeces)
A bacterial species identified by metagenomic analyses. This species includes all bacteria with genomes that show ≥95% average nucleotide identity (ANI) to the type genome, which has been assigned the MAG ID ChiHjej11B10-19426 and which is available via NCBI BioSample SAMN15816696. The GC content of the type genome is 60.46% and the genome length is 1.8 Mbp.
**Description of *Candidatus* Tidjanibacter gallistercoris sp. nov.**
*Candidatus* Tidjanibacter gallistercoris (gal.li.ster’co.ris. L. masc. n. *gallus* chicken; L. neut. n. *stercus* dung; N.L. gen. n. *gallistercoris* of chicken faeces)
A bacterial species identified by metagenomic analyses. This species includes all bacteria with genomes that show ≥95% average nucleotide identity (ANI) to the type genome, which has been assigned the MAG ID ChiSxjej4B16-7142 and which is available via NCBI BioSample SAMN15816746. The GC content of the type genome is 58.50% and the genome length is 2.0 Mbp.
**Description of *Candidatus* Treponema excrementipullorum sp. nov.**
*Candidatus* Treponema excrementipullorum (ex.cre.men.ti.pul.lo’rum. L. neut. n. *excrementum* excrement; L. masc. n. *pullus* a young chicken; N.L. gen. n. *excrementipullorum* of young chicken excrement)
A bacterial species identified by metagenomic analyses. This species includes all bacteria with genomes that show ≥95% average nucleotide identity (ANI) to the type genome, which has been assigned the MAG ID Gambia15-2214 and which is available via NCBI BioSample SAMN15816896. Although GTDB has assigned this species to the genus it calls Treponema_F, this genus designation cannot be incorporated into a well-formed binomial, so in naming this species, we have used the current validly published name for the genus. The GC content of the type genome is 39.91% and the genome length is 2.3 Mbp.

**Table:** 

**Description of *Candidatus* Treponema faecavium sp. nov.**
*Candidatus* Treponema faecavium (faec.a’vi.um. L. fem. n. *faex, faecis* excrement; L. fem. n. *avis* bird; N.L. gen. n. *faecavium* of bird faeces)
A bacterial species identified by metagenomic analyses. This species includes all bacteria with genomes that show ≥95% average nucleotide identity (ANI) to the type genome, which has been assigned the MAG ID USASDec8-330 and which is available via NCBI BioSample SAMN15816910. Although GTDB has assigned this species to the genus it calls Treponema_F, this genus designation cannot be incorporated into a well-formed binomial, so in naming this species, we have used the current validly published name for the genus. The GC content of the type genome is 53.41% and the genome length is 2.2 Mbp.
**Description of *Candidatus* Ureaplasma intestinipullorum sp. nov.**
*Candidatus* Ureaplasma intestinipullorum (in.tes.ti.ni.pul.lo’rum. L. neut. n. *intestinum* gut; L. masc. n. *pullus* a young chicken; N.L. gen. n. *intestinipullorum* of the gut of young chickens)
A bacterial species identified by metagenomic analyses. This species includes all bacteria with genomes that show ≥95% average nucleotide identity (ANI) to the type genome, which has been assigned the MAG ID A5-1222 and which is available via NCBI BioSample SAMN15816777. The GC content of the type genome is 24.43% and the genome length is 0.6 Mbp.
**Description of *Candidatus* Ventrenecus gen. nov.**
*Candidatus* Ventrenecus (Ventr.en.e’cus. L. masc. n. *venter* the belly; Gr. masc. *enoikos* inhabitant; N.L. masc. n. *Ventrenecus* a microbe associated with faeces)
A bacterial genus identified by metagenomic analyses. The genus includes all bacteria with genomes that show ≥60% average amino acid identity (AAI) to the type genome from the type species *Candidatus* Ventrenecus avicola. This is a name for the alphanumeric GTDB genus UMGS1217. This genus has been assigned by GTDB-Tk v1.3.0 working on GTDB Release 05-RS95 (Chaumeil et al., 2019; Parks et al., 2020) to the order *RF39* and to the family *CAG-1000*.
**Description of *Candidatus* Ventrenecus avicola sp. nov.**
*Candidatus* Ventrenecus avicola (a.vi’co.la. L. fem. n. *avis* bird; L. suff. *-cola* inhabitant of; N.L. n. *avicola* inhabitant of birds)
A bacterial species identified by metagenomic analyses. This species includes all bacteria with genomes that show ≥95% average nucleotide identity (ANI) to the type genome, which has been assigned the MAG ID ChiW22-487 and which is available via NCBI BioSample SAMN15817076. The GC content of the type genome is 31.09% and the genome length is 1.3 Mbp.
**Description of *Candidatus* Ventrenecus stercoripullorum sp. nov.**
*Candidatus* Ventrenecus stercoripullorum (ster.co.ri.pul.lo’rum. L. neut. n. *stercus* dung; L. masc. n. *pullus* a young chicken; N.L. gen. n. *stercoripullorum* of the faceces of young chickens)
A bacterial species identified by metagenomic analyses. This species includes all bacteria with genomes that show ≥95% average nucleotide identity (ANI) to the type genome, which has been assigned the MAG ID CHK197-17881 and which is available via NCBI BioSample SAMN15817010. The GC content of the type genome is 30.90% and the genome length is 1.4 Mbp.
**Description of *Candidatus* Ventricola gen. nov.**
*Candidatus* Ventricola (Ven.tri’co.la. L. masc. n. *venter* the belly; L. suff. *-cola* inhabitant of; N.L. fem. n. *Ventricola* a microbe associated with faeces)
A bacterial genus identified by metagenomic analyses. The genus includes all bacteria with genomes that show ≥60% average amino acid identity (AAI) to the type genome from the type species *Candidatus* Ventricola intestinavium. This is a name for the alphanumeric GTDB genus SFFH01. This genus has been assigned by GTDB-Tk v1.3.0 working on GTDB Release 05-RS95 (Chaumeil et al., 2019; Parks et al., 2020) to the order *Christensenellales* and to the family *CAG-74*.
**Description of *Candidatus* Ventricola gallistercoris sp. nov.**
*Candidatus* Ventricola gallistercoris (gal.li.ster’co.ris. L. masc. n *gallus* chicken; L. neut. n. *stercus* dung; N.L. gen. n. *gallistercoris* of chicken faeces)
A bacterial species identified by metagenomic analyses. This species includes all bacteria with genomes that show ≥95% average nucleotide identity (ANI) to the type genome, which has been assigned the MAG ID ChiHcec16-310 and which is available via NCBI BioSample SAMN15817100. The GC content of the type genome is 60.63% and the genome length is 2.4 Mbp.
**Description of *Candidatus* Ventricola intestinavium sp. nov.**
*Candidatus* Ventricola intestinavium (in.tes.tin.a’vi.um. L. neut. n. *intestinum* gut; L. fem. n. *avis* bird; N.L. gen. n. *intestinavium* of the gut of birds)
A bacterial species identified by metagenomic analyses. This species includes all bacteria with genomes that show ≥95% average nucleotide identity (ANI) to the type genome, which has been assigned the MAG ID 8987 and which is available via NCBI BioSample SAMN15817130. The GC content of the type genome is 59.95% and the genome length is 2.2 Mbp.
**Description of *Candidatus* Ventrimonas gen. nov.**
*Candidatus* Ventrimonas (Ven.tri.mo’nas. L. masc. n. *venter* the belly; L. fem. n. *monas* a monad; N.L. fem. n. *Ventrimonas* a microbe associated with faeces)
A bacterial genus identified by metagenomic analyses. The genus includes all bacteria with genomes that show ≥60% average amino acid identity (AAI) to the type genome from the type species *Candidatus* Ventrimonas merdavium. This is a name for the alphanumeric GTDB genus UBA9502. This genus has been assigned by GTDB-Tk v1.3.0 working on GTDB Release 05-RS95 (Chaumeil et al., 2019; Parks et al., 2020) to the order *Lachnospirales* and to the family *Lachnospiraceae*.
**Description of *Candidatus* Ventrimonas merdavium sp. nov.**
*Candidatus* Ventrimonas merdavium (merd.a’vi.um. L. fem. n. *merda* faeces; L. fem. n. *avis* bird; N.L. gen. n. *merdavium* of bird faeces)
A bacterial species identified by metagenomic analyses. This species includes all bacteria with genomes that show ≥95% average nucleotide identity (ANI) to the type genome, which has been assigned the MAG ID USAMLcec2-739 and which is available via NCBI BioSample SAMN15817118. The GC content of the type genome is 57.43% and the genome length is 3.0 Mbp.
**Description of *Candidatus* Ventrisoma gen. nov.**
*Candidatus* Ventrisoma (Ven.tri.so’ma. L. masc. n. *venter* the belly; Gr. neut. n. *soma* a body; N.L. neut. n. *Ventrisoma* a microbe associated with faeces)
A bacterial genus identified by metagenomic analyses. The genus includes all bacteria with genomes that show ≥60% average amino acid identity (AAI) to the type genome from the type species *Candidatus* Ventrisoma faecale. This is a name for the alphanumeric GTDB genus UC5-1-2E3. This genus has been assigned by GTDB-Tk v1.3.0 working on GTDB Release 05-RS95 (Chaumeil et al., 2019; Parks et al., 2020) to the order *Lachnospirales* and to the family *Lachnospiraceae*.
**Description of *Candidatus* Ventrisoma faecale sp. nov.**
*Candidatus* Ventrisoma faecale (fae.ca’le. L. neut. adj. *faecale* of faeces)
A bacterial species identified by metagenomic analyses. This species includes all bacteria with genomes that show ≥95% average nucleotide identity (ANI) to the type genome, which has been assigned the MAG ID ChiHcolR19-5415 and which is available via NCBI BioSample SAMN15817173. This is a new name for the alphanumeric GTDB species sp001304875. The GC content of the type genome is 55.91% and the genome length is 2.8 Mbp.
**Description of *Candidatus* Ventrousia gen. nov.**
*Candidatus* Ventrousia (Ventr.ou’si.a. L. masc. n. *venter* the belly; Gr. fem. n. *ousia* an essence; N.L. fem. n. *Ventrousia* a microbe associated with faeces)
A bacterial genus identified by metagenomic analyses. The genus includes all bacteria with genomes that show ≥60% average amino acid identity (AAI) to the type genome from the type species *Candidatus* Ventrousia excrementavium. This is a name for the alphanumeric GTDB genus SCN-57-10. This genus has been assigned by GTDB-Tk v1.3.0 working on GTDB Release 05-RS95 (Chaumeil et al., 2019; Parks et al., 2020) to the order *Oscillospirales* and to the family *Butyricicoccaceae*.
**Description of *Candidatus* Ventrousia excrementavium sp. nov.**
*Candidatus* Ventrousia excrementavium (ex.cre.ment.a’vi.um. L. neut. n. *excrementum* excrement; L. fem. n. *avis* bird; N.L. gen. n. *excrementavium* of bird excrement)
A bacterial species identified by metagenomic analyses. This species includes all bacteria with genomes that show ≥95% average nucleotide identity (ANI) to the type genome, which has been assigned the MAG ID CHK191-8634 and which is available via NCBI BioSample SAMN15817048. The GC content of the type genome is 57.61% and the genome length is 2.1 Mbp.
**Description of *Candidatus* Yaniella excrementavium sp. nov.**
*Candidatus* Yaniella excrementavium (ex.cre.ment.a’vi.um. L. neut. n. *excrementum* excrement; L. fem. n. *avis* bird; N.L. gen. n. *excrementavium* of bird excrement)
A bacterial species identified by metagenomic analyses. This species includes all bacteria with genomes that show ≥95% average nucleotide identity (ANI) to the type genome, which has been assigned the MAG ID ChiHjej13B12-778 and which is available via NCBI BioSample SAMN15816702. The GC content of the type genome is 55.30% and the genome length is 2.5 Mbp.
**Description of *Candidatus* Yaniella excrementigallinarum sp. nov.**
*Candidatus* Yaniella excrementigallinarum (ex.cre.men.ti.gal.li.na’rum. L. neut. n. *excrementum* excrement; L. fem. n. *gallina* hen; N.L. gen. n. *excrementigallinarum* of hen excrement)
A bacterial species identified by metagenomic analyses. This species includes all bacteria with genomes that show ≥95% average nucleotide identity (ANI) to the type genome, which has been assigned the MAG ID 4905 and which is available via NCBI BioSample SAMN15816764. The GC content of the type genome is 53.17% and the genome length is 2.6 Mbp.

### Taxonomic diversity of cultured bacterial isolates

To extend our metagenomics analyses, we applied culture-based methods to six faecal samples that appeared species-rich in Kraken 2 analyses and in so doing obtained 282 isolates from aerobic culture (~80% of isolates) and anaerobic culture (~20% of isolates) (Table S16). All isolates underwent genome sequencing on the Illumina platform and phylogenetic analysis to enable taxonomic assignment. The resulting chicken gut culture collection was found to contain 56 genera, 93 species and 162 strains drawn from five phyla. These included thirty novel species, with all novel species confirmed to originate from a monophyletic group through phylogenetic analysis against all available reference genomes of their respective genus (Fig. S2). Curiously, there was no overlap between the species that we obtained and those reported by Medvecky et al. (2018), suggesting that we are far from exhausting the set of species that can be cultured from this habitat. As with the metagenomic species, all novel or previously unnamed genera and species from cultured isolates were assigned Linnaean binomials (Table 2; Table S17). Species-level ANI clustering of all MAGs and all cultured isolates according to phylum is provided in Fig. S3.

**Table 2:** Protologues for new taxa cultured from chicken faeces.

**Description of *Acinetobacter pecorum* sp. nov.**
(pe.co’rum. L. gen. pl. n. *pecorum* of flocks of sheep, birds etc., as this species has been isolated from chickens and sheep)
The type strain for this bacterial species has been cultured from chicken faeces after overnight incubation on brain-heart infusion agar at 37 °C under aerobic conditions. The species has been assigned to this genus by the software tool GTDB-Tk v1.3.0 applied to the Genome Taxonomy Database (GTDB) Release 05-RS95 (Chaumeil et al., 2019; Parks et al., 2020). Its status as a species within this genus has been confirmed by phylogenetic analysis of all available reference genomes from the genus (Fig. S3).
The species includes all bacteria with genomes that show ≥95% average nucleotide identity (ANI) to the genome of the type strain, which is available via GenBank accession GCA_014837015.1. The GC content of the type strain is 42.9% and the genome size is 3.2 Mbp. GTDB has given this species the alphanumerical designation sp001647535 and the two other genomes assigned to this species are derived from sheep isolates (RefSeq assembly accessions GCF_001647535.1, GCF_001647575.1) (Gupta et al., 2016). The type strain has been deposited in NCTC and DSMZ, where it can be identified via the original strain reference Sa1BUA6.
**Description of *Arthrobacter gallicola* sp. nov.**
(gal.li’co.la. L. masc. n. *gallus* a cock; N.L. suff. *-cola* an inhabitant of; N.L. masc. or fem. n. *gallicola* an inhabitant of the chicken)
The type strain for this bacterial species has been cultured from chicken faeces after overnight incubation on Columbia blood agar at 37 °C under aerobic conditions. The species has been assigned to this genus by the software tool GTDB-Tk v1.3.0 applied to the Genome Taxonomy Database (GTDB) Release 05-RS95 (Chaumeil et al., 2019; Parks et al., 2020). Its status as a species within this genus has been confirmed by phylogenetic analysis of all available reference genomes from the genus (Fig. S3).
The species includes all bacteria with genomes that show ≥95% average nucleotide identity (ANI) to the genome of the type strain, which is available via GenBank accession GCA_014836775.1. The GC content of the type strain is 65.5% and the genome size is 3.7 Mbp. Although GTDB has assigned this species to the genus it calls *Arthrobacter_B*, this genus designation cannot be incorporated into a well-formed binomial, so in naming this species, we have used the basonym for the genus. The type strain has been deposited in NCTC and DSMZ, where it can be identified via the original strain reference Sa2CUA1.
**Description of *Arthrobacter pullicola* sp. nov.**
(pul.li’co.la. L. masc. n. *pullus* a young chicken; N.L. suff. *-cola* an inhabitant of; N.L. masc. or fem. n. *pullicola* an inhabitant of young chickens)
The type strain for this bacterial species has been cultured from chicken faeces after overnight incubation on brain-heart infusion agar at 37 °C under aerobic conditions. The species has been assigned to this genus by the software tool GTDB-Tk v1.3.0 applied to the Genome Taxonomy Database (GTDB) Release 05-RS95 (Chaumeil et al., 2019; Parks et al., 2020). Its status as a species within this genus has been confirmed by phylogenetic analysis of all available reference genomes from the genus (Fig. S3).
The species includes all bacteria with genomes that show ≥95% average nucleotide identity (ANI) to the genome of the type strain, which is available via GenBank accession GCA_014836875.1. The GC content of the type strain is 65.7% and the genome size is 3.7 Mbp. Although GTDB has assigned this species to the genus it calls *Arthrobacter_B*, this genus designation cannot be incorporated into a well-formed binomial, so in naming this species, we have used the basonym for the genus. The type strain has been deposited in NCTC and DSMZ, where it can be identified via the original strain reference Sa2BUA2.
**Description of *Bacillus norwichensis* sp. nov.**
(nor.wich.en’sis. N.L. masc. adj. *norwichensis* pertaining to English city of Norwich, where the organism was isolated)
The type strain for this bacterial species has been cultured from chicken faeces after overnight incubation on Columbia blood agar at 37 °C under aerobic conditions. The species has been assigned to this genus by the software tool GTDB-Tk v1.3.0 applied to the Genome Taxonomy Database (GTDB) Release 05-RS95 (Chaumeil et al., 2019; Parks et al., 2020). Its status as a species within this genus has been confirmed by phylogenetic analysis of all available reference genomes from the genus (Fig. S3).
The species includes all bacteria with genomes that show ≥95% average nucleotide identity (ANI) to the genome of the type strain, which is available via GenBank accession GCA_014836955.1. The GC content of the type strain is 40.2% and the genome size is 4.7 Mbp. Although GTDB has assigned this species to the genus it calls *Bacillus_AM*, this genus designation cannot be incorporated into a well-formed binomial, so in naming this species, we have used the basonym for the genus. The type strain has been deposited in NCTC and DSMZ, where it can be identified via the original strain reference Sa1BUA2
**Description of *Brevibacterium gallinarum* sp. nov.**
(gal.li.na’rum. L. pl. gen. n. *gallinarum* of hens)
The type strain for this bacterial species has been cultured from chicken faeces after overnight incubation on brain-heart infusion agar at 37 °C under aerobic conditions. The species has been assigned to this genus by the software tool GTDB-Tk v1.3.0 applied to the Genome Taxonomy Database (GTDB) Release 05-RS95 (Chaumeil et al., 2019; Parks et al., 2020). Its status as a species within this genus has been confirmed by phylogenetic analysis of all available reference genomes from the genus (Fig. S3).
The species includes all bacteria with genomes that show ≥95% average nucleotide identity (ANI) to the genome of the type strain, which is available via GenBank accession GCA_014836885.1. The GC content of the type strain is 67.0% and the genome size is 3.2 Mbp. The type strain has been deposited in NCTC and DSMZ, where it can be identified via the original strain reference Re57
**Description of *Brevundimonas guildfordensis* sp. nov.**
(guild.ford.en’sis. N.L. fem. adj. *guildfordensis* pertaining to English town Guildford, home to the University of Surrey, where the samples were processed)
The type strain for this bacterial species has been cultured from chicken faeces after overnight incubation on Columbia blood agar at 37 °C under aerobic conditions. The species has been assigned to this genus by the software tool GTDB-Tk v1.3.0 applied to the Genome Taxonomy Database (GTDB) Release 05-RS95 (Chaumeil et al., 2019; Parks et al., 2020). Its status as a species within this genus has been confirmed by phylogenetic analysis of all available reference genomes from the genus (Fig. S3).
The species includes all bacteria with genomes that show ≥95% average nucleotide identity (ANI) to the genome of the type strain, which is available via GenBank accession GCA_014836405.1. The GC content of the type strain is 67.3% and the genome size is 2.9 Mbp. The type strain has been deposited in NCTC and DSMZ, where it can be identified via the original strain reference Sa3CVA3
**Description of *Cellulomonas avistercoris*** sp. nov.
(a.vi.ster’co.ris. L. fem. n. *avis* bird; L. neut. n. *stercus* dung; N.L. gen. n. *avistercoris* of bird faeces)
The type strain for this bacterial species has been cultured from chicken faeces after overnight incubation on Columbia blood agar at 37 °C under aerobic conditions. The species has been assigned to this genus by the software tool GTDB-Tk v1.3.0 applied to the Genome Taxonomy Database (GTDB) Release 05-RS95 (Chaumeil et al., 2019; Parks et al., 2020). Its status as a species within this genus has been confirmed by phylogenetic analysis of all available reference genomes from the genus (Fig. S3).
The species includes all bacteria with genomes that show ≥95% average nucleotide identity (ANI) to the genome of the type strain, which is available via GenBank accession GCA_014836445.1. The GC content of the type strain is 74.5% and the genome size is 4.2 Mbp. The type strain has been deposited in NCTC and DSMZ, where it can be identified via the original strain reference Sa3CUA2
**Description of *Clostridium cibarium* sp. nov.**
(ci.ba’ri.um. L. neut. adj. *cibarium* pertaining to food, as this species has been isolated from chickens and zha-chili)
The type strain for this bacterial species has been cultured from chicken faeces after overnight incubation on Columbia blood agar at 37 °C under anaerobic conditions. The species has been assigned to this genus by the software tool GTDB-Tk v1.3.0 applied to the Genome Taxonomy Database (GTDB) Release 05-RS95 (Chaumeil et al., 2019; Parks et al., 2020). Its status as a species within this genus has been confirmed by phylogenetic analysis of all available reference genomes from the genus (Fig. S3).
The species includes all bacteria with genomes that show ≥95% average nucleotide identity (ANI) to the genome of the type strain, which is available via GenBank accession GCA_014836335.1. The GC content of the type strain is 29.8% and the genome size is 4.3 Mbp. GTDB has given this species the alphanumerical designation sp007115085. One other isolate from this species (RefSeq accession GCA_007115085.1) has been cultured from zha-chili, a Chinese fermented food. The type strain has been deposited in NCTC and DSMZ, where it can be identified via the original strain reference Sa3CVN1.
**Description of *Clostridium gallinarum* sp. nov.**
(gal.li.na’rum. L. pl. gen. n. *gallinarum* of hens)
The type strain for this bacterial species has been cultured from chicken faeces after overnight incubation on Columbia blood agar at 37 °C under anaerobic conditions. The species has been assigned to this genus by the software tool GTDB-Tk v1.3.0 applied to the Genome Taxonomy Database (GTDB) Release 05-RS95 (Chaumeil et al., 2019; Parks et al., 2020). Its status as a species within this genus has been confirmed by phylogenetic analysis of all available reference genomes from the genus (Fig. S3).
The species includes all bacteria with genomes that show ≥95% average nucleotide identity (ANI) to the genome of the type strain, which is available via GenBank accession GCA_014836325.1. The GC content of the type strain is 27.2% and the genome size is 3.4 Mbp. The type strain has been deposited in NCTC and DSMZ, where it can be identified via the original strain reference Sa3CUN1.
**Description of *Clostridium faecium* sp. nov.**
(fae’ci.um. N.L. gen. pl. n. *faecium*, of faeces, as this species has been isolated from chicken and human faeces)
The type strain for this bacterial species has been cultured from chicken faeces after overnight incubation on brain-heart infusion agar at 37 °C under anaerobic conditions. The species has been assigned to this genus by the software tool GTDB-Tk v1.3.0 applied to the Genome Taxonomy Database (GTDB) Release 05-RS95 (Chaumeil et al., 2019; Parks et al., 2020). Its status as a species within this genus has been confirmed by phylogenetic analysis of all available reference genomes from the genus (Fig. S3).
The species includes all bacteria with genomes that show ≥95% average nucleotide identity (ANI) to the genome of the type strain, which is available via GenBank accession GCA_014836835.1. The GC content of the type strain is 28.7% and the genome size is 3.9 Mbp. Although GTDB has assigned this species to the genus it calls *Clostridium_J*, this genus designation cannot be incorporated into a well-formed binomial, so in naming this species, we have used the basonym for the genus. GTDB has given this species the alphanumerical designation sp900547625, which includes a gut isolate from a preterm human infant (GenBank accession GCA_900547625.1). The type strain has been deposited in NCTC and DSMZ, where it can be identified via the original strain reference N37
**Description of *Comamonas avium* sp. nov.**
(a’vi.um. L. gen. pl. n. *avium* of birds)
The type strain for this bacterial species has been cultured from chicken faeces after overnight incubation on brain-heart infusion agar at 37 °C under aerobic conditions. The species has been assigned to this genus by the software tool GTDB-Tk v1.3.0 applied to the Genome Taxonomy Database (GTDB) Release 05-RS95 (Chaumeil et al., 2019; Parks et al., 2020). Its status as a species within this genus has been confirmed by phylogenetic analysis of all available reference genomes from the genus (Fig. S3).
The species includes all bacteria with genomes that show ≥95% average nucleotide identity (ANI) to the genome of the type strain, which is available via GenBank accession GCA_014836675.1. The GC content of the type strain is 57.5% and the genome size is 3.9 Mbp. The type strain has been deposited in NCTC and DSMZ, where it can be identified via the original strain reference Sa2CVA6
**Description of *Corynebacterium gallinarum* sp. nov.**
(gal.li.na’rum. L. pl. gen. n. *gallinarum* of hens)
The type strain for this bacterial species has been cultured from chicken faeces after overnight incubation on YCFA (yeast extract, casitone and fatty acid) agar at 37 °C under aerobic conditions. The species has been assigned to this genus by the software tool GTDB-Tk v1.3.0 applied to the Genome Taxonomy Database (GTDB) Release 05-RS95 (Chaumeil et al., 2019; Parks et al., 2020). Its status as a species within this genus has been confirmed by phylogenetic analysis of all available reference genomes from the genus (Fig. S3).
The species includes all bacteria with genomes that show ≥95% average nucleotide identity (ANI) to the genome of the type strain, which is available via GenBank accession GCA_014837045.1. The GC content of the type strain is 63.1% and the genome size is 3.1 Mbp. The type strain has been deposited in NCTC and DSMZ, where it can be identified via the original strain reference Sa1YVA5
**Description of *Cytobacillus stercorigallinarum* sp. nov.**
(ster.co.ri.gal.li.na’rum. L. neut. n. *stercus* faeces; L. fem. n. *gallina* a hen; N.L. gen. n. *stercorigallinarum* of hen faeces)
The type strain for this bacterial species has been cultured from chicken faeces after overnight incubation on brain-heart infusion agar at 37 °C under aerobic conditions. The species has been assigned to this genus by the software tool GTDB-Tk v1.3.0 applied to the Genome Taxonomy Database (GTDB) Release 05-RS95 (Chaumeil et al., 2019; Parks et al., 2020). Its status as a species within this genus has been confirmed by phylogenetic analysis of all available reference genomes from the genus (Fig. S3).
The species includes all bacteria with genomes that show ≥95% average nucleotide identity (ANI) to the genome of the type strain, which is available via GenBank accession GCA_014836495.1. The GC content of the type strain is 36.8% and the genome size is 4.4 Mbp. GTDB has assigned this species to the genus it calls *Bacillus_AA*, which cannot be incorporated into a well-formed binomial. However, according to (Patel & Gupta, 2020), the newly named genus *Cytobacillus* encompasses other species classified by GTDB within the genus designation *Bacillus_AA* and therefore *Cytobacillus* is treated as a synonym of GTDB *Bacillus_AA*. The type strain has been deposited in NCTC and DSMZ, where it can be identified via the original strain reference Sa5YUA1.
**Description of *Escherichia whittamii* sp. nov.**
(whit.tam’i.i. N.L. gen. n. *whittamii*, named in honour of American microbiologist Thomas S. Whittam)
The type strain for this bacterial species has been cultured from chicken faeces after overnight incubation on Columbia blood agar at 37 °C under anaerobic conditions. The species has been assigned to this genus by the software tool GTDB-Tk v1.3.0 applied to the Genome Taxonomy Database (GTDB) Release 05-RS95 (Chaumeil et al., 2019; Parks et al., 2020). Its status as a species within this genus has been confirmed by phylogenetic analysis of reference genomes from the genus (Fig. 4).
The species includes all bacteria with genomes that show ≥95% average nucleotide identity (ANI) to the genome of the type strain, which is available via GenBank accession GCA_014836715.1. The GC content of the type strain is 50.6% and the genome size is 4.6 Mbp. GTDB has given this species the alphanumerical designation sp001660175 and has assigned two other cultured isolates to this species, both of which come from birds (RefSeq assembly accessions GCF_001660175.1, GCF_002965485.1) (Clermont et al., 2011; Gangiredla et al., 2018). The type strain has been deposited in NCTC and DSMZ, where it can be identified via the original strain reference Sa2BVA5.
**Description of *Fictibacillus norfolkensis* sp. nov.**
(nor.folk.en’sis. N.L. masc. adj. *norfolkensis* pertaining to the English county of Norfolk)
The type strain for this bacterial species has been cultured from chicken faeces after overnight incubation on YCFA (yeast extract, casitone and fatty acid) agar at 37 °C under aerobic conditions. The species has been assigned to this genus by the software tool GTDB-Tk v1.3.0 applied to the Genome Taxonomy Database (GTDB) Release 05-RS95 (Chaumeil et al., 2019; Parks et al., 2020). Its status as a species within this genus has been confirmed by phylogenetic analysis of all available reference genomes from the genus (Fig. S3).
The species includes all bacteria with genomes that show ≥95% average nucleotide identity (ANI) to the genome of the type strain, which is available via GenBank accession GCA_014836645.1. The GC content of the type strain is 39.5% and the genome size is 4.0 Mbp. Although GTDB has assigned this species to the genus it calls *Fictibacillus_B*, this genus designation cannot be incorporated into a well-formed binomial, so in naming this species, we have used the basonym for the genus. The type strain has been deposited in NCTC and DSMZ, where it can be identified via the original strain reference Sa2CUA10
**Description of *Kaistella pullorum* sp. nov.**
(pul.lo’rum. L. gen. pl. n. *pullorum* of chickens)
The type strain for this bacterial species has been cultured from chicken faeces after overnight incubation on Columbia blood agar at 37 °C under aerobic conditions. The species has been assigned to this genus by the software tool GTDB-Tk v1.3.0 applied to the Genome Taxonomy Database (GTDB) Release 05-RS95 (Chaumeil et al., 2019; Parks et al., 2020). Its status as a species within this genus has been confirmed by phylogenetic analysis of all available reference genomes from the genus (Fig. S3).
The species includes all bacteria with genomes that show ≥95% average nucleotide identity (ANI) to the genome of the type strain, which is available via GenBank accession GCA_014837035.1. The GC content of the type strain is 42.9% and the genome size is 2.6 Mbp. The type strain has been deposited in NCTC and DSMZ, where it can be identified via the original strain reference Sa1CVA4
**Description of *Limosilactobacillus avistercoris* sp. nov.**
(a.vi.ster’co.ris. L. fem. n. *avis* bird; L. neut. n. *stercus* dung; N.L. gen. n. *avistercoris* of bird faeces)
The type strain for this bacterial species has been cultured from chicken faeces after overnight incubation on brain-heart infusion agar at 37 °C under anaerobic conditions. The species has been assigned to this genus by the software tool GTDB-Tk v1.3.0 applied to the Genome Taxonomy Database (GTDB) Release 05-RS95 (Chaumeil et al., 2019; Parks et al., 2020). Its status as a species within this genus has been confirmed by phylogenetic analysis of all available reference genomes from the genus (Fig. S3).
The species includes all bacteria with genomes that show ≥95% average nucleotide identity (ANI) to the genome of the type strain, which is available via GenBank accession GCA_014836425.1. The GC content of the type strain is 39.9% and the genome size is 1.8 Mbp. GTDB has assigned this species to the genus it calls *Lactobacillus_H*, which cannot be incorporated into a well-formed binomial. However, according to (Zheng et al., 2020) *Limosilactobacillus* encompasses other species classified by GTDB within the genus designation *Lactobacillus_H* and therefore *Limosilactobacillus* is treated as a synonym for GDTB designation *Lactobacillus_H*. The type strain has been deposited in NCTC and DSMZ, where it can be identified via the original strain reference Sa3CUN2
**Description of *Luteimonas colneyensis* sp. nov.**
(col.ney.en’sis. N.L. fem. adj. *colneyensis* pertaining to the English village of Colney, home to the Quadram Institute, where the species was first described)
The type strain for this bacterial species has been cultured from chicken faeces after overnight incubation on Columbia blood agar at 37 °C under aerobic conditions. The species has been assigned to this genus by the software tool GTDB-Tk v1.3.0 applied to the Genome Taxonomy Database (GTDB) Release 05-RS95 (Chaumeil et al., 2019; Parks et al., 2020). Its status as a species within this genus has been confirmed by phylogenetic analysis of all available reference genomes from the genus Fig. S3).
The species includes all bacteria with genomes that show ≥95% average nucleotide identity (ANI) to the genome of the type strain, which is available via GenBank accession GCA_014836665.1. The GC content of the type strain is 71.0% and the genome size is 3.0 Mbp. The type strain has been deposited in NCTC and DSMZ, where it can be identified via the original strain reference Sa2BVA3
**Description of *Microbacterium commune* sp. nov.**
(com.mu’ne. L. neut. adj. *commune* common, referring to diverse habitats, as this species has been isolated from mosquitos and chicken)
The type strain for this bacterial species has been cultured from chicken faeces after overnight incubation on Columbia blood agar at 37 °C under aerobic conditions. The species has been assigned to this genus by the software tool GTDB-Tk v1.3.0 applied to the Genome Taxonomy Database (GTDB) Release 05-RS95 (Chaumeil et al., 2019; Parks et al., 2020). Its status as a species within this genus has been confirmed by phylogenetic analysis of all available reference genomes from the genus (Fig. S3).
The species includes all bacteria with genomes that show ≥95% average nucleotide identity (ANI) to the genome of the type strain, which is available via GenBank accession GCA_014836945.1. The GC content of the type strain is 70.3% and the genome size is 3.3 Mbp. GTDB has given this species the alphanumerical designation sp001878835, which also contains a mosquito isolate (RefSeq accession GCF_001878835.1). The type strain has been deposited in NCTC and DSMZ, where it can be identified via the original strain reference Re1.
**Description of *Microbacterium gallinarum* sp. nov.**
(gal.li.na’rum. L. pl. gen. n. *gallinarum* of hens)
The type strain for this bacterial species has been cultured from chicken faeces after overnight incubation on brain-heart infusion agar at 37 °C under aerobic conditions. The species has been assigned to this genus by the software tool GTDB-Tk v1.3.0 applied to the Genome Taxonomy Database (GTDB) Release 05-RS95 (Chaumeil et al., 2019; Parks et al., 2020). Its status as a species within this genus has been confirmed by phylogenetic analysis of all available reference genomes from the genus (Fig. S3).
The species includes all bacteria with genomes that show ≥95% average nucleotide identity (ANI) to the genome of the type strain, which is available via GenBank accession GCA_014837165.1. The GC content of the type strain is 69.4% and the genome size is 2.8 Mbp. The type strain has been deposited in NCTC and DSMZ, where it can be identified via the original strain reference Sa1CUA4
**Description of *Microbacterium pullorum* sp. nov.**
(pul.lo’rum. L. gen. pl. n. *pullorum* of chickens)
The type strain for this bacterial species has been cultured from chicken faeces after overnight incubation on Columbia blood agar at 37 °C under aerobic conditions. The species has been assigned to this genus by the software tool GTDB-Tk v1.3.0 applied to the Genome Taxonomy Database (GTDB) Release 05-RS95 (Chaumeil et al., 2019; Parks et al., 2020). Its status as a species within this genus has been confirmed by phylogenetic analysis of all available reference genomes from the genus (Fig. S3).
The species includes all bacteria with genomes that show ≥95% average nucleotide identity (ANI) to the genome of the type strain, which is available via GenBank accession GCA_014836535.1. The GC content of the type strain is 70.1% and the genome size is 3.1 Mbp. The type strain has been deposited in NCTC and DSMZ, where it can be identified via the original strain reference Sa4CUA7
**Description of *Oceanitalea stevensii* sp. nov.**
(ste.ven’si.i. N.L. gen. n. *stevensii*, named in honour of British microbiologist Mark Stevens)
The type strain for this bacterial species has been cultured from chicken faeces after overnight incubation on brain-heart infusion agar at 37 °C under aerobic conditions. The species has been assigned to this genus by the software tool GTDB-Tk v1.3.0 applied to the Genome Taxonomy Database (GTDB) Release 05-RS95 (Chaumeil et al., 2019; Parks et al., 2020). Its status as a species within this genus has been confirmed by phylogenetic analysis of all available reference genomes from the genus (Fig. S3).
The species includes all bacteria with genomes that show ≥95% average nucleotide identity (ANI) to the genome of the type strain, which is available via GenBank accession GCA_014837105.1. The GC content of the type strain is 73.4% and the genome size is 3.5 Mbp. The type strain has been deposited in NCTC and DSMZ, where it can be identified via the original strain reference Sa1BUA1.
**Description of *Ochrobactrum gallinarum* sp. nov.**
(gal.li.na’rum. L. pl. gen. n. *gallinarum* of hens)
The type strain for this bacterial species has been cultured from chicken faeces after overnight incubation on brain-heart infusion at 37 °C under aerobic conditions. The species has been assigned to this genus by the software tool GTDB-Tk v1.3.0 applied to the Genome Taxonomy Database (GTDB) Release 05-RS95 (Chaumeil et al., 2019; Parks et al., 2020). Its status as a species within this genus has been confirmed by phylogenetic analysis of all available reference genomes from the genus (Fig. S3).
The species includes all bacteria with genomes that show ≥95% average nucleotide identity (ANI) to the genome of the type strain, which is available via GenBank accession GCA_014836735.1. The GC content of the type strain is 53.5% and the genome size is 5.0 Mbp. The type strain has been deposited in NCTC and DSMZ, where it can be identified via the original strain reference Sa2BUA5
**Description of *Oerskovia douganii* sp. nov.**
(dou.ga’ni.i. N.L. gen. n. *douganii* named in honour of British microbiologist Gordon Dougan)
The type strain for this bacterial species has been cultured from chicken faeces after overnight incubation on brain-heart infusion at 37 °C under aerobic conditions. The species has been assigned to this genus by the software tool GTDB-Tk v1.3.0 applied to the Genome Taxonomy Database (GTDB) Release 05-RS95 (Chaumeil et al., 2019; Parks et al., 2020). Its status as a species within this genus has been confirmed by phylogenetic analysis of all available reference genomes from the genus (Fig. S3).
The species includes all bacteria with genomes that show ≥95% average nucleotide identity (ANI) to the genome of the type strain, which is available via GenBank accession GCA_015142735.1. The GC content of the type strain is 72.5% and the genome size is 4.3 Mbp. The type strain has been deposited in NCTC and DSMZ, where it can be identified via the original strain reference Sa1BUA8
**Description of *Oerskovia gallyi* sp. nov.**
(gal’ly.i. N.L. gen. n. *gallyi* named in honour of British microbiologist David Gally)
The type strain for this bacterial species has been cultured from chicken faeces after overnight incubation on brain-heart infusion agar at 37 °C under aerobic conditions. The species has been assigned to this genus by the software tool GTDB-Tk v1.3.0 applied to the Genome Taxonomy Database (GTDB) Release 05-RS95 (Chaumeil et al., 2019; Parks et al., 2020). Its status as a species within this genus has been confirmed by phylogenetic analysis of all available reference genomes from the genus (Fig. S3).
The species includes all bacteria with genomes that show ≥95% average nucleotide identity (ANI) to the genome of the type strain, which is available via GenBank accession GCA_014836745.1. The GC content of the type strain is 72.5% and the genome size is 4.3 Mbp. The type strain has been deposited in NCTC and DSMZ, where it can be identified via the original strain reference Sa2CUA8
**Description of *Oerskovia merdavium* sp. nov.**
(merd.a’vi.um. L. fem. n. *merda* faeces; L. fem. n. *avis* bird; N.L. gen. n. *merdavium* of bird faeces)
The type strain for this bacterial species has been cultured from chicken faeces after overnight incubation on Columbia blood agar at 37 °C under aerobic conditions. The species has been assigned to this genus by the software tool GTDB-Tk v1.3.0 applied to the Genome Taxonomy Database (GTDB) Release 05-RS95 (Chaumeil et al., 2019; Parks et al., 2020). Its status as a species within this genus has been confirmed by phylogenetic analysis of all available reference genomes from the genus (Fig. S3).
The species includes all bacteria with genomes that show ≥95% average nucleotide identity (ANI) to the genome of the type strain, which is available via GenBank accession GCA_014836755.1. The GC content of the type strain is 72.1% and the genome size is 4.5 Mbp. The type strain has been deposited in NCTC and DSMZ, where it can be identified via the original strain reference Sa2CUA9.
**Description of *Oerskovia rustica* sp. nov.**
(rus’ti.ca. L fem adj. *rustica* of the countryside, as isolates have been obtained from soil and chickens)
The type strain for this bacterial species has been cultured from chicken faeces after overnight incubation on Columbia blood agar at 37 °C under aerobic conditions. The species has been assigned to this genus by the software tool GTDB-Tk v1.3.0 applied to the Genome Taxonomy Database (GTDB) Release 05-RS95 (Chaumeil et al., 2019; Parks et al., 2020). Its status as a species within this genus has been confirmed by phylogenetic analysis of all available reference genomes from the genus (Fig. S3).
The species includes all bacteria with genomes that show ≥95% average nucleotide identity (ANI) to the genome of the type strain, which is available via GenBank accession GCA_014836555.1. The GC content of the type strain is 72.5% and the genome size is 4.4 Mbp. GTDB has given this species the alphanumerical designation sp005937995, which includes a soil isolate (RefSeq accession GCF_005937995.2). The type strain has been deposited in NCTC and DSMZ, where it can be identified via the original strain reference Sa4CUA1.
**Description of *Paenibacillus gallinarum* sp. nov.**
(gal.li.na’rum. L. pl. gen. n. *gallinarum* of hens)
The type strain for this bacterial species has been cultured from chicken faeces after overnight incubation on Columbia blood agar at 37 °C under aerobic conditions. The species has been assigned to this genus by the software tool GTDB-Tk v1.3.0 applied to the Genome Taxonomy Database (GTDB) Release 05-RS95 (Chaumeil et al., 2019; Parks et al., 2020). Its status as a species within this genus has been confirmed by phylogenetic analysis of all available reference genomes from the genus (Fig. S3).
The species includes all bacteria with genomes that show ≥95% average nucleotide identity (ANI) to the genome of the type strain, which is available via GenBank accession GCA_014836635.1. The GC content of the type strain is 41.2% and the genome size is 5.4 Mbp. The type strain has been deposited in NCTC and DSMZ, where it can be identified via the original strain reference Sa2BVA9
**Description of *Phocaeicola faecium* sp. nov.**
(fae’ci.um L. gen. pl. n. *faecium*, of faeces)
The type strain for this bacterial species has been cultured from chicken faeces after overnight incubation on YCFA (yeast extract, casitone and fatty acid) agar at 37 °C under anaerobic conditions. The species has been assigned to this genus by the software tool GTDB-Tk v1.3.0 applied to the Genome Taxonomy Database (GTDB) Release 05-RS95 (Chaumeil et al., 2019; Parks et al., 2020). Its status as a species within this genus has been confirmed by phylogenetic analysis of all available reference genomes from the genus (Fig. S3).
The species includes all bacteria with genomes that show ≥95% average nucleotide identity (ANI) to the genome of the type strain, which is available via GenBank accession GCA_014837055.1. The GC content of the type strain is 45.6% and the genome size is 3.5 Mbp. GTDB has given this species the alphanumerical designation sp900540105, which includes a gut isolate from an infant human (GenBank accession GCA_900540105.1). The type strain has been deposited in NCTC and DSMZ, where it can be identified via the original strain reference Sa1YUN3
**Description of *Phocaeicola intestinalis* sp. nov.**
(in.tes.ti.na’lis. N.L. masc./fem. adj. *intestinalis*, pertaining to the intestines)
The type strain for this bacterial species has been cultured from chicken faeces after overnight incubation on Columbia blood agar at 37 °C under anaerobic conditions. The species has been assigned to this genus by the software tool GTDB-Tk v1.3.0 applied to the Genome Taxonomy Database (GTDB) Release 05-RS95 (Chaumeil et al., 2019; Parks et al., 2020). Its status as a species within this genus has been confirmed by phylogenetic analysis of all available reference genomes from the genus (Fig. S3).
The species includes all bacteria with genomes that show ≥95% average nucleotide identity (ANI) to the genome of the type strain, which is available via GenBank accession GCA_014837065.1. The GC content of the type strain is 45.7% and the genome size is 4.1 Mbp. GTDB has given this species the alphanumerical designation sp002161565, which includes isolates from the human and chicken guts (GenBank accession numbers GCA_000432695.1, GCA_900540165.1, GCF_002159615.1, GCF_002159755.1, GCF_002160215.1, GCF_002161565.1). The type strain has been deposited in NCTC and DSMZ, where it can be identified via the original strain reference Sa1CVN1.
**Description of *Planococcus wigleyi* sp. nov.**
(wig’ley.i. N.L. masc. gen. n. *wigleyi* named in honour of British microbiologist Paul Wigley)
The type strain for this bacterial species has been cultured from chicken faeces after overnight incubation on brain-heart infusion agar at 37 °C under aerobic conditions. The species has been assigned to this genus by the software tool GTDB-Tk v1.3.0 applied to the Genome Taxonomy Database (GTDB) Release 05-RS95 (Chaumeil et al., 2019; Parks et al., 2020). Its status as a species within this genus has been confirmed by phylogenetic analysis of all available reference genomes from the genus (Fig. S3).
The species includes all bacteria with genomes that show ≥95% average nucleotide identity (ANI) to the genome of the type strain, which is available via GenBank accession GCA_014836985.1. The GC content of the type strain is 45.0% and the genome size is 3.8 Mbp. Although GTDB has assigned a genus name with an alphabetic suffix *Planococcus_A*, this genus designation cannot be incorporated into a well-formed binomial, so in naming this species, we have used the basonym for the genus. The type strain has been deposited in NCTC and DSMZ, where it can be identified via the original strain reference Sa1BUA13
**Description of *Psychrobacillus faecigallinarum* sp. nov.**
(fae.ci.gal.li.na’rum. L. fem. n. *faex, faecis* faeces; L. fem. n. *gallina* a hen; N.L. gen. n. *faecigallinarum* of hen faeces)
The type strain for this bacterial species has been cultured from chicken faeces after overnight incubation on brain-heart infusion agar at 37 °C under aerobic conditions. The species has been assigned to this genus by the software tool GTDB-Tk v1.3.0 applied to the Genome Taxonomy Database (GTDB) Release 05-RS95 (Chaumeil et al., 2019; Parks et al., 2020). Its status as a species within this genus has been confirmed by phylogenetic analysis of all available reference genomes from the genus (Fig. S3).
The species includes all bacteria with genomes that show ≥95% average nucleotide identity (ANI) to the genome of the type strain, which is available via GenBank accession GCA_014836595.1. The GC content of the type strain is 36.5% and the genome size is 4.0 Mbp. The type strain has been deposited in NCTC and DSMZ, where it can be identified via the original strain reference Sa2BUA9
**Description of *Psychrobacter communis* sp. nov.**
(com.mu’nis. L. masc. adj. *communis* common, referring to diverse habitats from which this species has been isolated, including chickens and soil)
The type strain for this bacterial species has been cultured from chicken faeces after overnight incubation on xyz medium at 37 °C under aerobic conditions. The species has been assigned to this genus by the software tool GTDB-Tk v1.3.0 applied to the Genome Taxonomy Database (GTDB) Release 05-RS95 (Chaumeil et al., 2019; Parks et al., 2020). Its status as a species within this genus has been confirmed by phylogenetic analysis of all available reference genomes from the genus (Fig. S3).
The species includes all bacteria with genomes that show ≥95% average nucleotide identity (ANI) to the genome of the type strain, which is available via GenBank accession GCA_014836505.1. The GC content of the type strain is 43.7% and the genome size is 3.0 Mbp. GTDB has given this species the alphanumerical designation sp001652315, which contains six environmental isolates from a variety of sources including soil (GCA_002332465.1, GCA_002439405.1, GCA_003524605.1, GCA_007280595.1, GCF_001652315.1, GCF_002836335.1) The type strain has been deposited in NCTC and DSMZ, where it can be identified via the original strain reference Sa4CVA2
**Description of *Serpens gallinarum* sp. nov.**
(gal.li.na’rum. L. pl. gen. n. *gallinarum* of hens)
The type strain for this bacterial species has been cultured from chicken faeces after overnight incubation on Columbia blood agar at 37 °C under aerobic conditions. The species has been assigned to this genus by the software tool GTDB-Tk v1.3.0 applied to the Genome Taxonomy Database (GTDB) Release 05-RS95 (Chaumeil et al., 2019; Parks et al., 2020). Its status as a species within this genus has been confirmed by phylogenetic analysis of all available reference genomes from the genus (Fig. S3).
The species includes all bacteria with genomes that show ≥95% average nucleotide identity (ANI) to the genome of the type strain, which is available via GenBank accession GCA_014836765.1. The GC content of the type strain is 61.0% and the genome size is 3.9 Mbp. Although GTDB has assigned a genus name with an alphabetic suffix *Pseudomonas_H*, which cannot be incorporated into a well-formed binomial. However, GDTB genus *Pseudomonas_H* includes *Pseudomonas flexibilis*, where the basonym is *Serpens* (Hespell, 1977), so we have used this genus name. The type strain has been deposited in NCTC and DSMZ, where it can be identified via the original strain reference Sa2CUA2
**Description of *Solibacillus faecavium* sp. nov.**
(faec.a’vi.um. L. fem. n. *faex, faecis* faeces; L. fem. n. *avis* bird; N.L. gen. n. *faecavium* of bird faeces)
The type strain for this bacterial species has been cultured from chicken faeces after overnight incubation on brain-heart infusion agar at 37 °C under aerobic conditions. The species has been assigned to this genus by the software tool GTDB-Tk v1.3.0 applied to the Genome Taxonomy Database (GTDB) Release 05-RS95 (Chaumeil et al., 2019; Parks et al., 2020). Its status as a species within this genus has been confirmed by phylogenetic analysis of all available reference genomes from the genus (Fig. S3).
The species includes all bacteria with genomes that show ≥95% average nucleotide identity (ANI) to the genome of the type strain, which is available via GenBank accession GCA_014836905.1. The GC content of the type strain is 37.1% and the genome size is 3.8 Mbp. The type strain has been deposited in NCTC and DSMZ, where it can be identified via the original strain reference A46
**Description of *Solibacillus merdavium* sp. nov.**
(merd.a’vi.um. L. fem. n. *merda* faeces; L. fem. n. *avis* bird; N.L. gen. n. *merdavium* of bird faeces)
The type strain for this bacterial species has been cultured from chicken faeces after overnight incubation on YCFA (yeast extract, casitone and fatty acid) agar at 37 °C under aerobic conditions. The species has been assigned to this genus by the software tool GTDB-Tk v1.3.0 applied to the Genome Taxonomy Database (GTDB) Release 05-RS95 (Chaumeil et al., 2019; Parks et al., 2020). Its status as a species within this genus has been confirmed by phylogenetic analysis of all available reference genomes from the genus (Fig. S3).
The species includes all bacteria with genomes that show ≥95% average nucleotide identity (ANI) to the genome of the type strain, which is available via GenBank accession GCA_014836935.1. The GC content of the type strain is 37.0% and the genome size is 3.8 Mbp. The type strain has been deposited in NCTC and DSMZ, where it can be identified via the original strain reference Sa1YVA6
**Description of *Sporosarcina gallistercoris* sp. nov.**
(gal.li.ster’co.ris. L. masc. n. *gallus* a cock; L. neut. n. *stercus* dung; N.L. gen. n. *gallistercoris* of faeces of a cock)
The type strain for this bacterial species has been cultured from chicken faeces after overnight incubation on YCFA (yeast extract, casitone and fatty acid) agar at 37 °C under aerobic conditions. The species has been assigned to this genus by the software tool GTDB-Tk v1.3.0 applied to the Genome Taxonomy Database (GTDB) Release 05-RS95 (Chaumeil et al., 2019; Parks et al., 2020). Its status as a species within this genus has been confirmed by phylogenetic analysis of all available reference genomes from the genus (Fig. S3).
The species includes all bacteria with genomes that show ≥95% average nucleotide identity (ANI) to the genome of the type strain, which is available via GenBank accession GCA_014836415.1. The GC content of the type strain is 44.1% and the genome size is 3.1 Mbp. Although GTDB has assigned a genus name with an alphabetic suffix *Sporosarcina_A*, this genus designation cannot be incorporated into a well-formed binomial, so in naming this species, we have used the basonym for the genus. The type strain has been deposited in NCTC and DSMZ, where it can be identified via the original strain reference Sa3CUA8
**Description of *Sporosarcina quadrami* sp. nov.**
(qua.dra’mi. N.L. gen. n. *quadrami*, of the Quadram Institute, where the species was first cultured.)
The type strain for this bacterial species has been cultured from chicken faeces after overnight incubation on brain-heart infusion agar at 37 °C under aerobic conditions. The species has been assigned to this genus by the software tool GTDB-Tk v1.3.0 applied to the Genome Taxonomy Database (GTDB) Release 05-RS95 (Chaumeil et al., 2019; Parks et al., 2020). Its status as a species within this genus has been confirmed by phylogenetic analysis of all available reference genomes from the genus (Fig. S3).
The species includes all bacteria with genomes that show ≥95% average nucleotide identity (ANI) to the genome of the type strain, which is available via GenBank accession GCA_014836615.1. The GC content of the type strain is 41.4% and the genome size is 3.6 Mbp. Although GTDB has assigned a genus name with an alphabetic suffix *Sporosarcina_B*, this genus designation cannot be incorporated into a well-formed binomial, so in naming this species, we have used the basonym for the genus. The type strain has been deposited in NCTC and DSMZ, where it can be identified via the original strain reference Sa2YVA2
**Description of *Stenotrophomonas pennii* sp. nov.**
(pen’ni.i. N.L. gen. n. *pennii*, named in honour of British microbiologist Charles W. Penn)
The type strain for this bacterial species has been cultured from chicken faeces after overnight incubation on brain-heart infusion agar at 37 °C under aerobic conditions. The species has been assigned to this genus by the software tool GTDB-Tk v1.3.0 applied to the Genome Taxonomy Database (GTDB) Release 05-RS95 (Chaumeil et al., 2019; Parks et al., 2020). Its status as a species within this genus has been confirmed by phylogenetic analysis of all available reference genomes from the genus (Fig. S3).
The species includes all bacteria with genomes that show ≥95% average nucleotide identity (ANI) to the genome of the type strain, which is available via GenBank accession GCA_014836545.1. The GC content of the type strain is 66.4% and the genome size is 3.9 Mbp. GTDB has given this species the alphanumerical designation sp002836635, which includes four environmental isolates (RefSeq accessions GCF_000834105.1, GCF_002836635.1, GCF_002836645.1, GCF_002836675.1). The type strain has been deposited in NCTC and DSMZ, where it can be identified via the original strain reference Sa5BUN4
**Description of *Ureibacillus galli* sp. nov.**
(gal’li. L. masc. gen. n. *galli* of a chicken)
The type strain for this bacterial species has been cultured from chicken faeces after overnight incubation on brain-heart infusion agar at 37 °C under aerobic conditions. The species has been assigned to this genus by the software tool GTDB-Tk v1.3.0 applied to the Genome Taxonomy Database (GTDB) Release 05-RS95 (Chaumeil et al., 2019; Parks et al., 2020). Its status as a species within this genus has been confirmed by phylogenetic analysis of all available reference genomes from the genus (Fig. S3).
The species includes all bacteria with genomes that show ≥95% average nucleotide identity (ANI) to the genome of the type strain, which is available via GenBank accession GCA_014836845.1. The GC content of the type strain is 35.2% and the genome size is 3.7 Mbp. The type strain has been deposited in NCTC and DSMZ, where it can be identified via the original strain reference Re31.
**Description of *Xanthomonas surreyensis* sp. nov.**
(sur.rey.en’sis. N.L. fem. adj. surreyensis pertaining to the English county of Surrey, where the samples were obtained)
The type strain for this bacterial species has been cultured from chicken faeces after overnight incubation on brain-heart infusion agar at 37 °C under aerobic conditions. The species has been assigned to this genus by the software tool GTDB-Tk v1.3.0 applied to the Genome Taxonomy Database (GTDB) Release 05-RS95 (Chaumeil et al., 2019; Parks et al., 2020). Its status as a species within this genus has been confirmed by phylogenetic analysis of all available reference genomes from the genus (Fig. S3).
The species includes all bacteria with genomes that show ≥95% average nucleotide identity (ANI) to the genome of the type strain, which is available via GenBank accession GCA_014836395.1. The GC content of the type strain is 68.8% and the genome size is 5.4 Mbp. Although GTDB has assigned a genus name with an alphabetic suffix *Xanthomonas_A*, this genus designation cannot be incorporated into a well-formed binomial, so in naming this species, we have used the basonym for the genus. The type strain has been deposited in NCTC and DSMZ, where it can be identified via the original strain reference Sa3BUA13.

**Figure 5:**
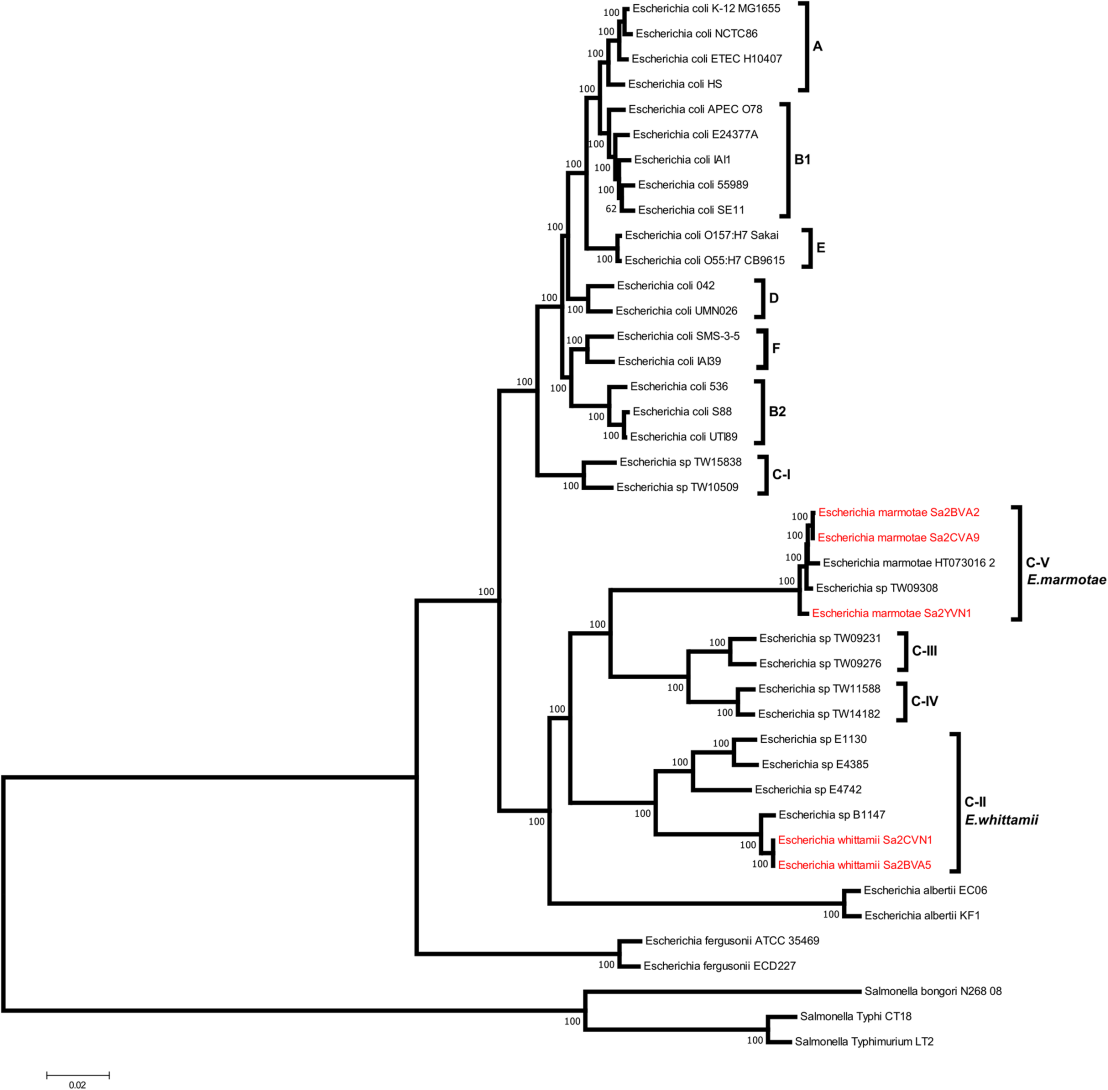
Phylogenetic tree showing the relationships between *Escherichia marmotae*, *Escherichia whittamii* and the other *Escherichia* species and cryptic clades. The tree was constructed by RAxML maximum likelihood analysis of a core genome alignment generated using Mugsy. The scale bar indicates the number of substitutions per site represented by the branch length shown. Numbers on branches indicate the percentage bootstrap support out of 100 replicates. Strains sequenced as part of this study are highlighted in red.

Interestingly, alongside ten cultured isolates of the well-characterised species *Escherichia coli*, we recovered three isolates from *Escherichia marmotae* (a species recently described in Himalayan marmots (Liu et al., 2015)). As previously reported, the *E. marmotae* strains cluster closely with the *Escherichia* Clade V (Liu et al., 2019; Walk, 2015), so all members of this clade should be considered members of this species (Fig. 5; Table S18). Further analysis of the GTDB species designated *Escherichia* sp001660175 (https://gtdb.ecogenomic.org/searches?s=al&q=sp001660175) confirmed that this species forms a monophyletic lineage that corresponds to Clade II, among the cryptic environmental clades described by Walk et al. (2009), which has subsequently been documented in birds (Clermont et al., 2011). As Clade II is comparable in divergence to the other *Escherichia* spp. and cryptic clades, we have therefore assigned the Linnaean binomial *Escherichia whittamii* to designate a new species (Table 2), honouring the outstanding contribution of Thomas S. Whittam to the study of *Escherichia* spp. (Walk & Feng, 2011).

**Figure 6:**
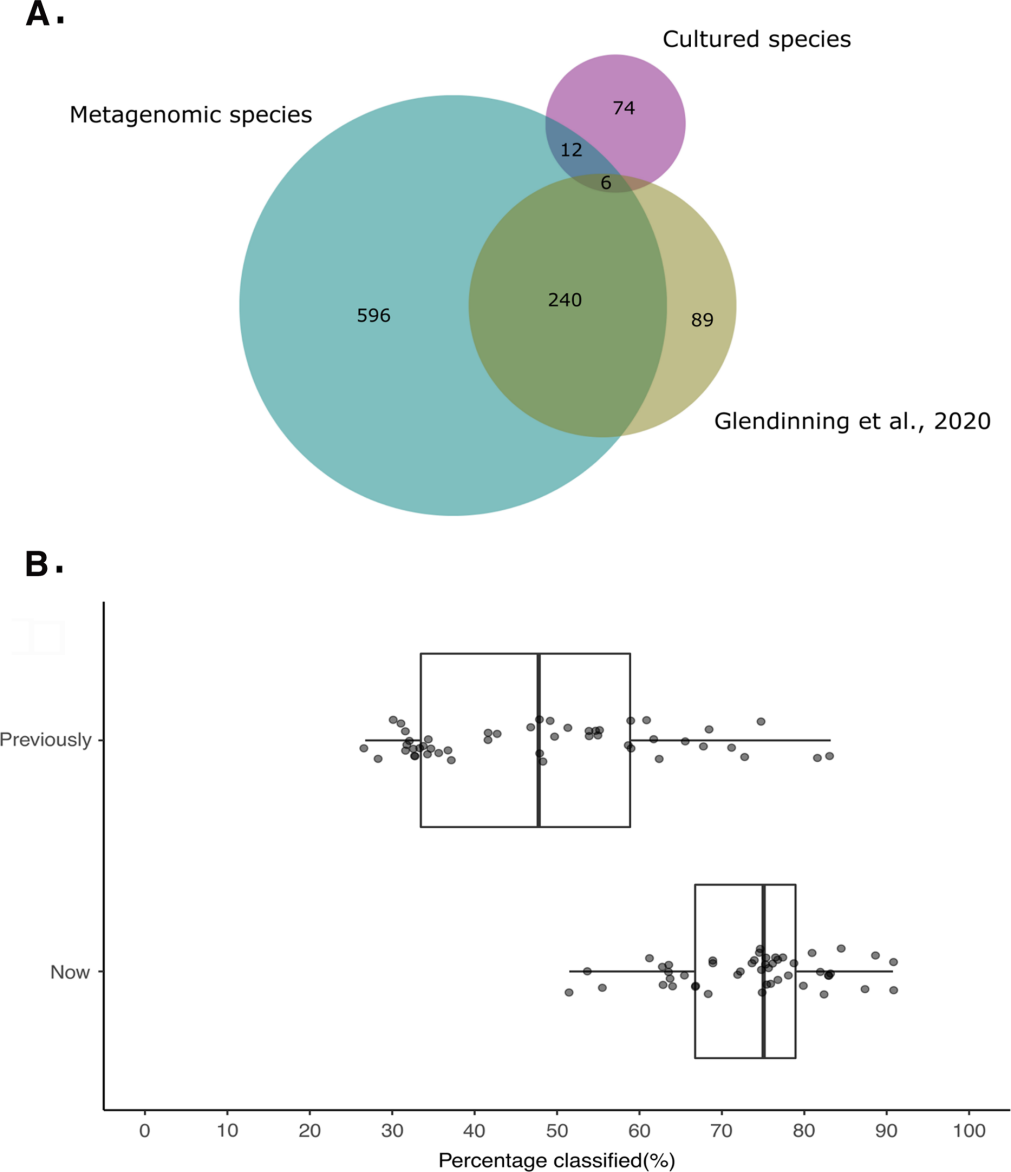
Sequence novelty. (A) Venn diagram showing shared and unique taxonomic species among three data sources; cultured isolates derived from six chicken faecal samples (Cultured species), metagenomic species identified from a combined dataset of >630 chicken gastrointestinal metagenome samples (Metagenomic species); MAGs also found by Glendinning et al. (2020). (B) Percentage of classified metagenomic reads derived from 50 chicken faecal samples according to a standard Kraken 2 database (Previously) and to a standard Kraken 2 database with the addition of the 2,344 genomic and metagenomic sequences derived from this study (Now).

We found that only 16 species were common to our cultured isolates and our MGS. Subsequent sequence mapping allowed us to detect a further two cultured species at ≥1× coverage in at least one metagenomic sample (Fig. 6A; Table S19). The genomes from cultured isolates were on average 20% larger than the corresponding MAG sequences retrieved from the same source sample (Table S20), which is in line with the completeness threshold of 80% we adopted in quality assurance of the MAGs. However, when we performed detailed gene content analyses on three abundant species in both cultured and metagenomic datasets—*Lactobacillus reuteri* (with the synonym *Limosilactobacillus reuteri), Escherichia coli* (including the synonym *Escherichia flexneri*) and *Enterococcus faecium*—we found that >99% of the genes from the core genomes and nearly half of the genes in the accessory genomes of cultured species were represented in at least one MAG. These observations suggest that our high-quality MAGs are sufficiently complete to warrant *Candidatus* names.

We analysed our chicken faecal metagenomes with a Kraken 2 database derived from genomes representing our candidate metagenomic and cultured species; this yielded a considerable improvement in the number of reads that can be classified through rapid phylogenetic profiling (Fig. 6B).

### Distribution of microbial species

**Figure 7:**
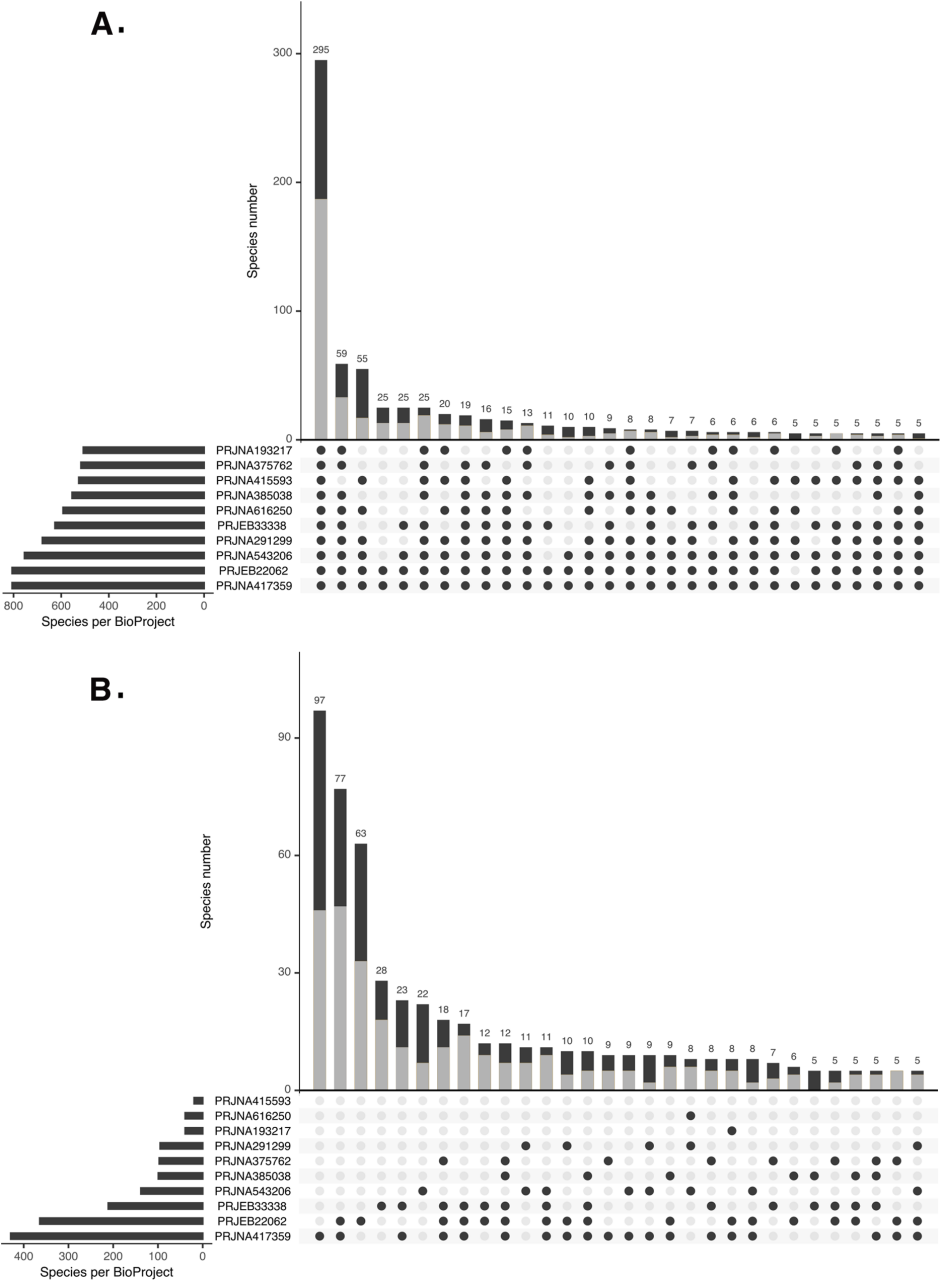
UpSet plots depicting presence of 820 metagenomic species across all BioProjects included within this study. (A) 1× coverage b. (B) 10× coverage. Bars are stacked according to taxonomic species novelty, with black-stacked bars depicting novel species and grey depicting species previously described in public databases or published studies. Only intersections with five or more species are shown.

An analysis of the distribution of 820 MGSs across the entire metagenomic dataset revealed marked variation between samples, with not a single species present at ≥1× coverage in all samples and only 39 species present in >90% of samples—although 441 species were present in >50% of samples at ≥1× coverage (Table S21). At ≥1× coverage, co-occurrence of nearly 300 species (*n* = 295) was identified across all 10 BioProjects (Fig. 7A), with no species identified in all BioProjects at ≥10× coverage (Fig. 7B). Focusing on samples from distinct anatomical or physiological sites (faeces, caecum etc.), we found no species present in all faecal samples at >1× coverage and only two species were found in all caecal samples at >1× coverage: both of them newly named in this study: *Candidatus* Paraprevotella stercoravium and Candidatus *Blautia pullistercoris* (the latter identified but not named by Glendinning et al. (2020)). These findings rule out the concept of a core chicken gut microbiome. Studies on the human gut microbiome provide a useful comparison in that, in a recent study, only 14 genera were found to be shared across 95% of samples from the human gut (Falony et al., 2016).

Among the species with high coverage, frequency is clearly linked to Bioproject. Although species quantification curves showed that the number of species identified increased rapidly with the number of samples, species discovery appeared to plateau at approximately 230 species after including only 50 metagenomes (Fig. S2A). Only two species appeared to be restricted (at ≥1× coverage) to just a single sample: *Aliarcobacter thereius* and *Candidatus* Avibacteroides faecavium. Correlation clustering confirmed structure in the data linked to BioProject (Fig. S2B)—for example, the BioProject from the study by Glendinning et al. (2020) clearly shows enhancement of clostridial species compared to other BioProjects, which reflects the fact that samples in that study were sourced from chicks with no post-hatching contact with an adult bird. However, the BioSamples do not appear to cluster by country (Fig. S2C) and show only limited clustering by anatomical/physiological sample site (Fig. S2D). Unfortunately, there is insufficient metadata for other potentially important factors, such as breed, age or diet to draw conclusions on how these might influence clustering.

## Discussion

Given the dominance of chickens in the planetary biomass, the chicken gut microbiome ranks as one of the most abundant microbial communities on the planet. Here, we have exploited two complementary approaches—metagenomics and culture—to create an extensive catalogue of genes, genomes and isolates from this important ecosystem. Our work illustrates the value of combining culture-dependent and culture-independent approaches in analysing microbiomes.

We have clearly demonstrated the advantages of shotgun metagenomic sequencing when applied to the chicken gut microbiome, providing catalogues of genes and genome sequences that takes us well beyond what can be achieved using 16S ribosomal RNA gene sequences. Similarly, the current study is much wider in scope than the previous study by Glendinning et al. (2020), not just including analyses of viral genomes and cultured isolates, while also incorporating MAGs built from data not just from that study but from all publicly available metagenomic datasets. Furthermore, the limited overlap between bacterial species represented among our cultured isolates and in our MGS reinforces the utility of the combined approach. Nonetheless, the substantial co-linearity between genomes obtained by the two approaches—and with those from another similar metagenomic study (Glendinning et al., 2020)—confirms the reliability of our binning approaches.

We were surprised to find such a remarkable phylogenetic diversity within this commonplace livestock ecosystem—diversity that rivals that associated with the human gut. Our work has more than doubled the number of bacterial species known to reside in the chicken gut and has resulted in the creation of an unprecedented number of new *Candidatus* species. By including well-formed Latin binomials with the genomes we have uploaded into public repositories, we have ensured that the new proposed names and associated sequences will be integrated into commonly used online taxonomies and databases and will provide a stable taxonomic nomenclature for future studies. In addition, we have provided proof-of-principle for a scalable approach to Linnaean nomenclature that could be applied to species recovered from other metagenomic assembly projects.

Given that we did not recover by culture some of the organisms that appear most abundant by metagenomics, there is clearly scope for additional culture-based investigations, using a wider range of cultural conditions—perhaps drawing on the precedent of the Human Microbiome Project to create and target a list of the ‘most-wanted-for-culture’ organisms documented by metagenomics (Fodor et al., 2012). The fact that novel metagenomic species are still being recovered from human gut datasets that include tens of thousands of metagenomes (Almeida et al., 2019)—twinned with the promise of novel long-read and proximity-capture approaches to metagenome analyses (Stewart et al., 2018)—make it clear that our attempts here to analyse all currently available chicken gut metagenomes provide far from the last word on microbial diversity in this abundant and important ecosystem. Nonetheless, the availability of so many novel genes, genome and species represents a substantial step forward.

## Conclusions

The extensive catalogue of genes, genomes and isolates we have created here substantially improves the coverage of the chicken gut microbiome in the public databases and will make it possible to profile sequences from the chicken gut much more rapidly, easily and comprehensively, providing a valuable resource that lays the ground-work for future comparative and intervention studies. This study also sets a provocative precedent—relevant not just to animal microbiomes, but to studies on all microbiomes—assigning well-formed Latin binomials to hundreds of metagenomic species in a scalable alternative to the automated use of bland, unstable, user-unfriendly alphanumerical designations. Drawing on the precedent set by the current study, we have recently extended this approach to encompass creation of more than a million new names for Bacteria and Archaea (Pallen, Telatin & Oren, 2020). Thus, the time is now ripe to bring Linnaeus right into the heart of microbiome studies.

## Supplemental Information

10.7717/peerj.10941/supp-1 Supplemental Information 1 Supplemental Data. ***Table S1***. Culture media used for culture of isolates from six chicken faecal samples.
***Table S2*.** Summary of sequencing data from 50 chicken faecal samples (BioProject ID PRJNA543206) using Illumina NextSeq sequencing platform
***Table S3*.** Bracken read based and relative abundance values for 50 chicken faecal samples from BioProject ID PRJNA543206.
***Table S4***. Summary of reads classified by Kraken 2 for 50 chicken faecal samples from BioProject ID PRJNA543206.
***Table S5*.** 1,455 dereplicated Scaffold sequences ≥10kb identified as Category 1 or Category 2 by VirSorter found in 50 metagenomic samples form chicken faeces.
***Table S6*.** BLASTN analysis for 10 bacteriophage genomes showing similarity to known reference bacteriophage sequences at nucleotide level (percentage identity >70%; query covering >50%). For each query sequence, the three best HITs (when ordered according to E-value) have been described.
***Table S7*.** Coverage values for four identified coliphages showing similarity to known bacteriophage genomes of *Escherichia coli* across the 50 metagenomic samples from which they were derived.
***Table S8*.** Family-level taxonomic classification for nearly 600 de-replicated, bacteriophage genomes of near-full completion.
***Table S9*.** Metadata and sequencing statistics associated with 582 publicly available metagenomic samples derived from the domesticated chicken.
***Table S10*.** Genome statistics for MAGs of >80% completion and <5% contamination constructed from 632 metagenomic samples and binned using MetaBAT2.
***Table S11***. Genome statistics for Metagenomic Species of >80% completion and <5% contamination derived from gene-based clustering from 632 metagenomic samples.
***Table S12*.** Taxonomic analysis for Metagenomic Species according to GTDB (release 95), Kraken 2 and SpecI classification.
***Table S13*.** Final genus and species level taxonomic classification for all de-replicated metagenomically-derived genome sequences. Newly assigned Latin binomials, alongside their respective etymologies have been provided where appropriate.
***Table S14***. PubMed hits associated with identified species and the chicken.
***Table S15*.** Latin and Greek roots used combinatorically to create new genus and species names.
***Table S16*.** Genome statistics, clustering statistics at 95% and 99% ANI and final taxonomic classification for all cultured isolates.
**Table S17.** Final genus and species level taxonomic classification for all de-replicated genome sequences from cultured isolates. Newly assigned Latin binomials, alongside their respective etymologies have been provided where appropriate.
***Table S18*.** Delineation of *E. whittamii* by ANI using genomes representing the full diversity of the genus Escherichia
***Table S19*.** Redundancy of MGS, cultured species and a comparable dataset of published MAGs (Glendinning et al., 2020).
***Table S20***. Comparison of genome statistics associated with species found in abundance in both metagenomic and cultured datasets
***Table S21***. Coverage statistics for 820 MGS sequences across 632 metagenomic samples

10.7717/peerj.10941/supp-2 Supplemental Information 2 Supplemental Figures. ***Figure S1*.** Comparisons between metagenomic samples according to BioProject from which they were sourced with regard to **a.** Number of paired reads input and **b.** Assembled scaffold N50. **c**. Completeness and contamination of MAGs and MGS derived from single assembly or co-assembly of all metagenomic samples. Quality estimates were estimated by CheckM and are reported for all Low Quality (designated Reconstructed) (≥50% completion, <5% contamination) Medium Quality (≥80% - ≤90% completion, <5% contamination) and High Quality (>90% completion, <5% contamination) MAGs, with MAGs clustered to form MGS being shown in blue.
***Figure S2a*.** Species accumulation curve for 632 samples over 10 publicly available BioProjects accounting for 820 MGS. Only studies with > 1 sample are included in this plot. A further curve has been plotted in black to depict species accumulation for all metagenomic samples from all BioProjects. Inset plot shows magnification of original plot for species 0-250 (.pdf format). ***b***. A heatmap depicting the abundance of the top 300 MGS, ordered by maximum sample abundance, across 632 samples derived from 10 BioProjects. Rows (MGS) were ordered by correlation clustering while columns (metagenomic sample) were ordered by source BioProject. Metagenomic species have been annotated with their taxonomic class. All data were Log_10_ transformed with Blue colour depicting species of low abundance and Red showing high abundance. ***c*. **Principle component analysis (PCA) based on Bray Curtis dissimilarity matrices between country of origin for MGS relative abundance. ***d*. **PCA based on Bray Curtis dissimilarity matrices based on GIT sample site for MGS relative abundance.
***Figure S3*. **Phylogenetic trees reconstructed for genomes of each novel cultured isolate against all available reference genomes of the respective genus obtained from NCBI. Trees were reconstructed using PhyloPhlAn 3.0.58 (Asnicar et al., 2020) against 400 marker genes before reconstruction using FastTree and RAxML of a MAFFT sequence alignment. Genomes of isolates cultured in this study have been highlighted in red and detailed with their respective strain identifier.
***Figure S4*. **Primary ANI clustering of all MAGs and cultured isolates separated according to phylum as determined by dREP. Each plot clusters at species-level according to 95% ANI, as shown by a continual dotted line. Sequence clusters are assigned colours according to designated species-level cluster.

## Additional Information and Declarations

### Data Availability

The following information was supplied regarding data availability: Data are available at BioProject ID PRJNA543206 and Figshare: Gilroy, Rachel (2020): Gene catalogue and MAGs. figshare. Dataset. DOI 10.6084/m9.figshare.13116809.v4. Gilroy, Rachel (2020): Cultured isolates. figshare. Dataset. DOI 10.6084/m9.figshare.13234556.v3. Gilroy, Rachel (2020): Gene catalogue and MAGs. figshare. Dataset. DOI 10.6084/m9.figshare.13116809.v4. Gilroy, Rachel (2020): Cultured isolates. figshare. Dataset. DOI 10.6084/m9.figshare.13234556.v3. Gilroy, Rachel (2020): MAGs. figshare. Dataset. DOI 10.6084/m9.figshare.13187396.v1. Gilroy, Rachel (2020): MAGs10. figshare. Dataset. DOI 10.6084/m9.figshare.13234478.v1.

### New Species Registration

The following information was supplied regarding the registration of a newly described species: Hundreads of new candidatus taxa and dozens of new cultured bacterial taxa are detailed in the article and Supplemental Material.
