# Supplementary material for: Extensive microbial diversity within the chicken gut microbiome revealed by metagenomics and culture
Source: PeerJ. 2021 Apr 6;9:e10941. doi: 10.7717/peerj.10941 (PMC8035907; doi:10.7717/peerj.10941)
Supplement: Supplemental Information 2 — Figure S1. Comparisons between metagenomic samples according to BioProject from which they were sourced with regard to a. Number of paired reads input and b. Assembled scaffold N50. c. Completeness and contamination of MAGs and MGS derived from single assembly or co-assembly of all metagenomic samples. Quality estimates were estimated by CheckM and are reported for all Low Quality (designated Reconstructed) (≥50% completion, <5% contamination) Medium Quality (≥80% - ≤90% completion, <5% contamination) and High Quality (>90% completion, <5% contamination) MAGs, with MAGs clustered to form MGS being shown in blue. Figure S2a. Species accumulation curve for 632 samples over 10 publicly available BioProjects accounting for 820 MGS. Only studies with > 1 sample are included in this plot. A further curve has been plotted in black to depict species accumulation for all metagenomic samples from all BioProjects. Inset plot shows magnification of original plot for species 0-250 (.pdf format). b. A heatmap depicting the abundance of the top 300 MGS, ordered by maximum sample abundance, across 632 samples derived from 10 BioProjects. Rows (MGS) were ordered by correlation clustering while columns (metagenomic sample) were ordered by source BioProject. Metagenomic species have been annotated with their taxonomic class. All data were Log10 transformed with Blue colour depicting species of low abundance and Red showing high abundance. c. Principle component analysis (PCA) based on Bray Curtis dissimilarity matrices between country of origin for MGS relative abundance. d. PCA based on Bray Curtis dissimilarity matrices based on GIT sample site for MGS relative abundance. Figure S3. Phylogenetic trees reconstructed for genomes of each novel cultured isolate against all available reference genomes of the respective genus obtained from NCBI. Trees were reconstructed using PhyloPhlAn 3.0.58 (Asnicar et al., 2020) against 400 marker genes before reconstruction using FastTree and RAxML o [file peerj-09-10941-s002.pdf]

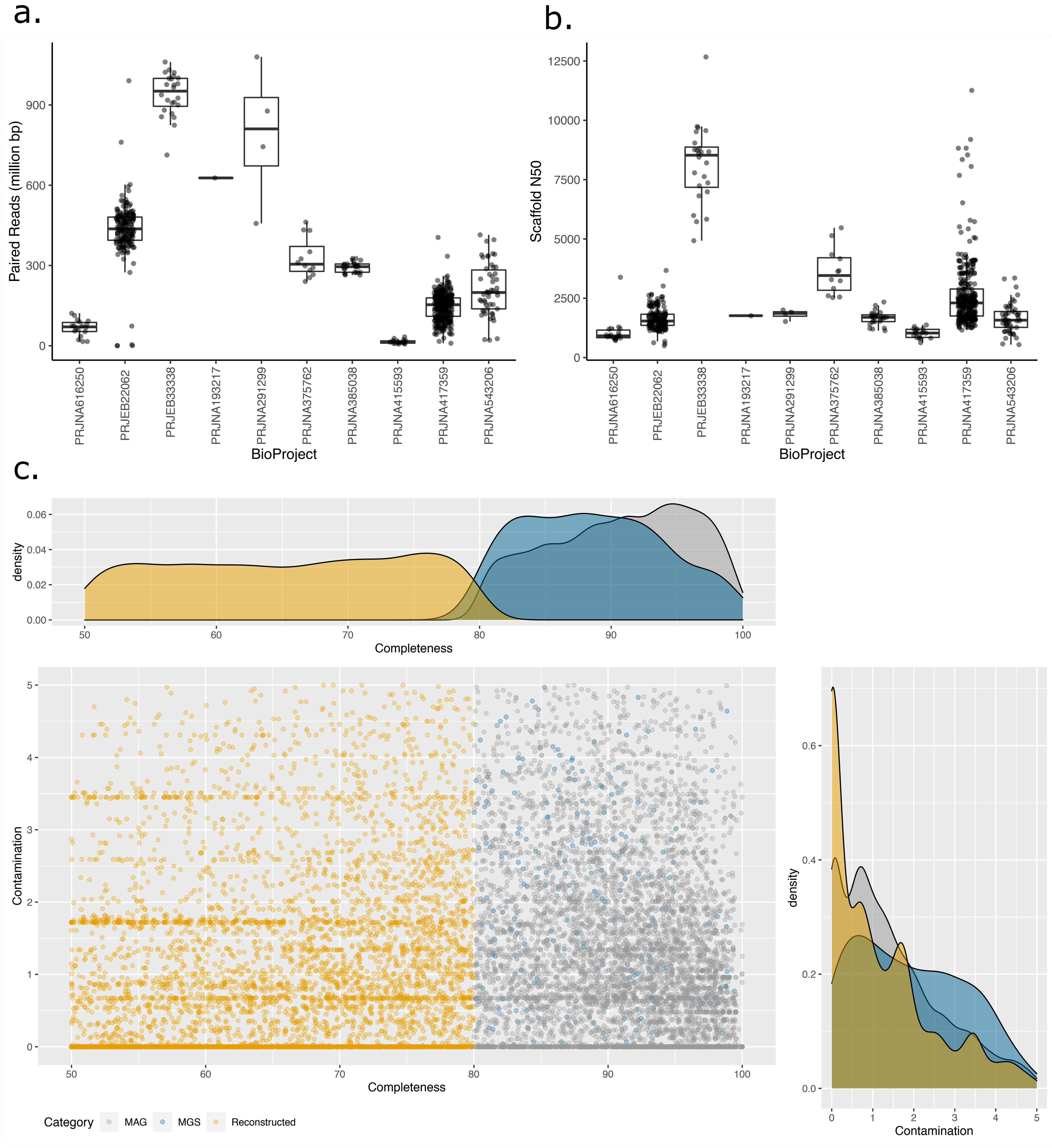

Figure S1. Comparisons between metagenomic samples according to BioProject from which they were sourced with regard to **a.** Number of paired reads input and **b.** Assembled scaffold N50. **c.** Completeness and contamination of MAGs and MGS derived from single assembly or co-assembly of all metagenomic samples. Quality estimates were estimated by CheckM and are reported for all Low Quality (designated Reconstructed) (≥50% completion, <5% contamination) Medium Quality (≥80% - ≤90% completion, <5% contamination) and High Quality (>90% completion, <5% contamination) MAGs, with MAGs clustered to form MGS being shown in blue.

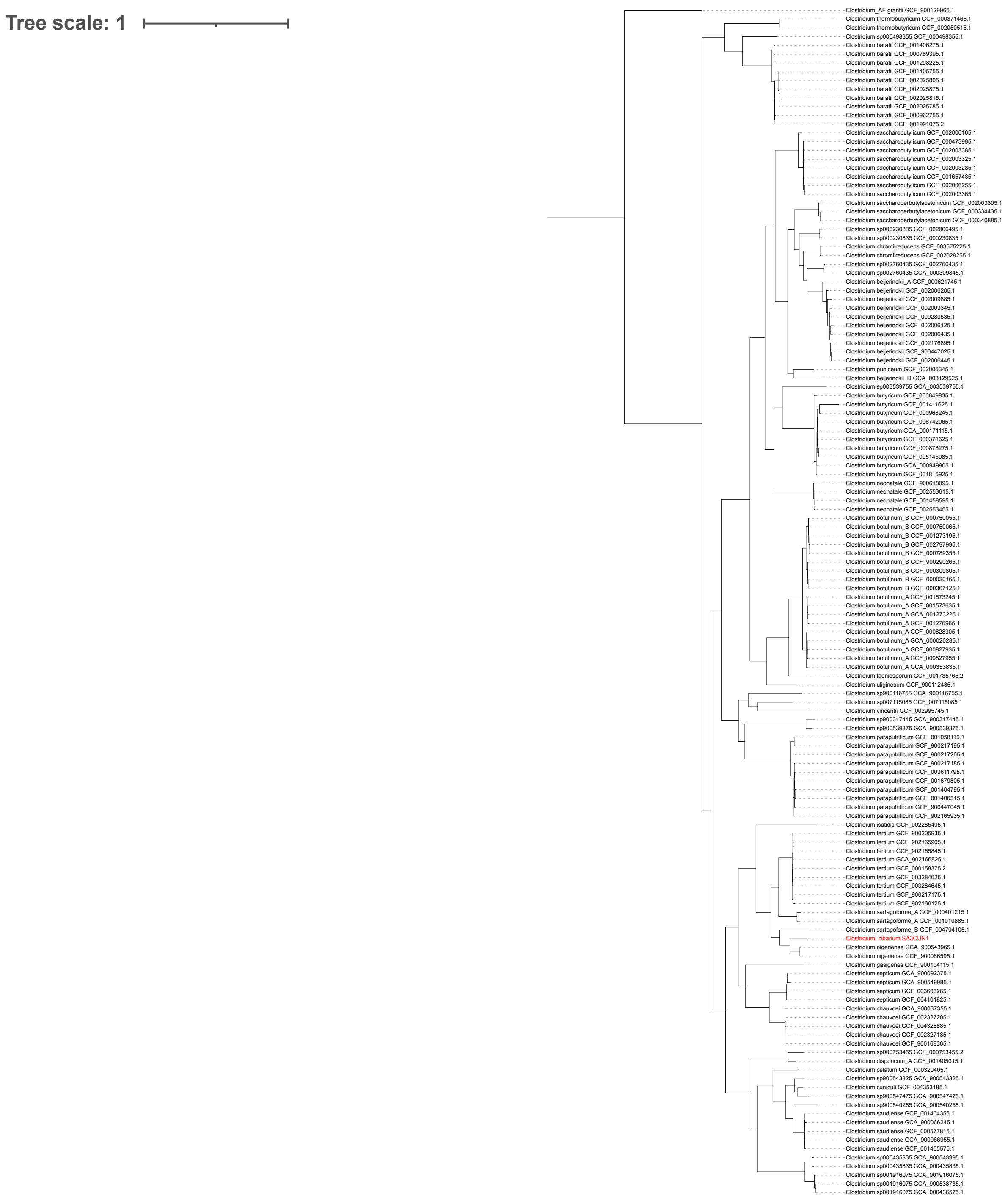
Tree scale: 1
Clostridium_AF grantii GCF_900129965.1
Clostridium thermobutyricum GCF_000371465.1
Clostridium thermobutyricum GCF_002050515.1
Clostridium sp000498355 GCF_000498355.1
Clostridium baratii GCF_001406275.1
Clostridium baratii GCF_000789395.1
Clostridium baratii GCF_001298225.1
Clostridium baratii GCF_001405755.1
Clostridium baratii GCF_002025805.1
Clostridium baratii GCF_002025875.1
Clostridium baratii GCF_002025815.1
Clostridium baratii GCF_002025785.1
Clostridium baratii GCF_000962755.1
Clostridium baratii GCF_001991075.2
Clostridium saccharobutylicum GCF_002006165.1
Clostridium saccharobutylicum GCF_000473995.1
Clostridium saccharobutylicum GCF_002003385.1
Clostridium saccharobutylicum GCF_002003325.1
Clostridium saccharobutylicum GCF_002003285.1
Clostridium saccharobutylicum GCF_001657435.1
Clostridium saccharobutylicum GCF_002006255.1
Clostridium saccharobutylicum GCF_002003365.1
Clostridium saccharoperbutylacetonicum GCF_002003305.1
Clostridium saccharoperbutylacetonicum GCF_000334435.1
Clostridium saccharoperbutylacetonicum GCF_000340885.1
Clostridium sp000230835 GCF_002006495.1
Clostridium sp000230835 GCF_000230835.1
Clostridium chromiireducens GCF_003575225.1
Clostridium chromiireducens GCF_002029255.1
Clostridium sp002760435 GCF_002760435.1
Clostridium sp002760435 GCA_000309845.1
Clostridium beijerinckii_A GCF_000621745.1
Clostridium beijerinckii GCF_002006205.1
Clostridium beijerinckii GCF_002009885.1
Clostridium beijerinckii GCF_002003345.1
Clostridium beijerinckii GCF_000280535.1
Clostridium beijerinckii GCF_002006125.1
Clostridium beijerinckii GCF_002006435.1
Clostridium beijerinckii GCF_002176895.1
Clostridium beijerinckii GCF_900447025.1
Clostridium beijerinckii GCF_002006445.1
Clostridium puniceum GCF_002006345.1
Clostridium beijerinckii_D GCA_003129525.1
Clostridium sp003539755 GCA_003539755.1
Clostridium butyricum GCF_003849835.1
Clostridium butyricum GCF_001411625.1
Clostridium butyricum GCF_000968245.1
Clostridium butyricum GCF_006742065.1
Clostridium butyricum GCA_000171115.1
Clostridium butyricum GCF_000371625.1
Clostridium butyricum GCF_000878275.1
Clostridium butyricum GCF_005145085.1
Clostridium butyricum GCA_000949905.1
Clostridium butyricum GCF_001815925.1
Clostridium neonatale GCF_900618095.1
Clostridium neonatale GCF_002553615.1
Clostridium neonatale GCF_001458595.1
Clostridium neonatale GCF_002553455.1
Clostridium botulinum_B GCF_000750055.1
Clostridium botulinum_B GCF_000750065.1
Clostridium botulinum_B GCF_001273195.1
Clostridium botulinum_B GCF_002797995.1
Clostridium botulinum_B GCF_000789355.1
Clostridium botulinum_B GCF_900290265.1
Clostridium botulinum_B GCF_000309805.1
Clostridium botulinum_B GCF_000020165.1
Clostridium botulinum_B GCF_000307125.1
Clostridium botulinum_A GCF_001573245.1
Clostridium botulinum_A GCF_001573635.1
Clostridium botulinum_A GCA_001273225.1
Clostridium botulinum_A GCF_001276965.1
Clostridium botulinum_A GCF_000828305.1
Clostridium botulinum_A GCA_000020285.1
Clostridium botulinum_A GCF_000827935.1
Clostridium botulinum_A GCF_000827955.1
Clostridium botulinum_A GCA_000353835.1
Clostridium taeniosporum GCF_001735765.2
Clostridium uliginosum GCF_900112485.1
Clostridium sp900116755 GCA_900116755.1
Clostridium sp007115085 GCF_007115085.1
Clostridium vincentii GCF_002995745.1
Clostridium sp900317445 GCA_900317445.1
Clostridium sp900539375 GCA_900539375.1
Clostridium paraputrificum GCF_001058115.1
Clostridium paraputrificum GCF_900217195.1
Clostridium paraputrificum GCF_900217205.1
Clostridium paraputrificum GCF_900217185.1
Clostridium paraputrificum GCF_003611795.1
Clostridium paraputrificum GCF_001679805.1
Clostridium paraputrificum GCF_001404795.1
Clostridium paraputrificum GCF_001406515.1
Clostridium paraputrificum GCF_900447045.1
Clostridium paraputrificum GCF_902165935.1
Clostridium isatidis GCF_002285495.1
Clostridium tertium GCF_900205935.1
Clostridium tertium GCF_902165905.1
Clostridium tertium GCF_902165845.1
Clostridium tertium GCA_902166825.1
Clostridium tertium GCF_000158375.2
Clostridium tertium GCF_003284625.1
Clostridium tertium GCF_003284645.1
Clostridium tertium GCF_900217175.1
Clostridium tertium GCF_902166125.1
Clostridium sartagoforme_A GCF_000401215.1
Clostridium sartagoforme_A GCF_001010885.1
Clostridium sartagoforme_B GCF_004794105.1
Clostridium cibarium SA3CUN1
Clostridium nigeriense GCA_900543965.1
Clostridium nigeriense GCF_900086595.1
Clostridium gasigenes GCF_900104115.1
Clostridium septicum GCA_900092375.1
Clostridium septicum GCA_900549985.1
Clostridium septicum GCF_003606265.1
Clostridium septicum GCF_004101825.1
Clostridium chauvoei GCA_900037355.1
Clostridium chauvoei GCF_002327205.1
Clostridium chauvoei GCF_004328885.1
Clostridium chauvoei GCF_002327185.1
Clostridium chauvoei GCF_900168365.1
Clostridium sp000753455 GCF_000753455.2
Clostridium disporicum_A GCF_001405015.1
Clostridium celatum GCF_000320405.1
Clostridium sp900543325 GCA_900543325.1
Clostridium cuniculi GCF_004353185.1
Clostridium sp900547475 GCA_900547475.1
Clostridium sp900540255 GCA_900540255.1
Clostridium saudiense GCF_001404355.1
Clostridium saudiense GCA_900066245.1
Clostridium saudiense GCF_000577815.1
Clostridium saudiense GCA_900066955.1
Clostridium saudiense GCF_001405575.1
Clostridium sp000435835 GCA_900543995.1
Clostridium sp000435835 GCA_000435835.1
Clostridium sp001916075 GCA_001916075.1
Clostridium sp001916075 GCA_900538735.1
Clostridium sp001916075 GCA_000436575.1

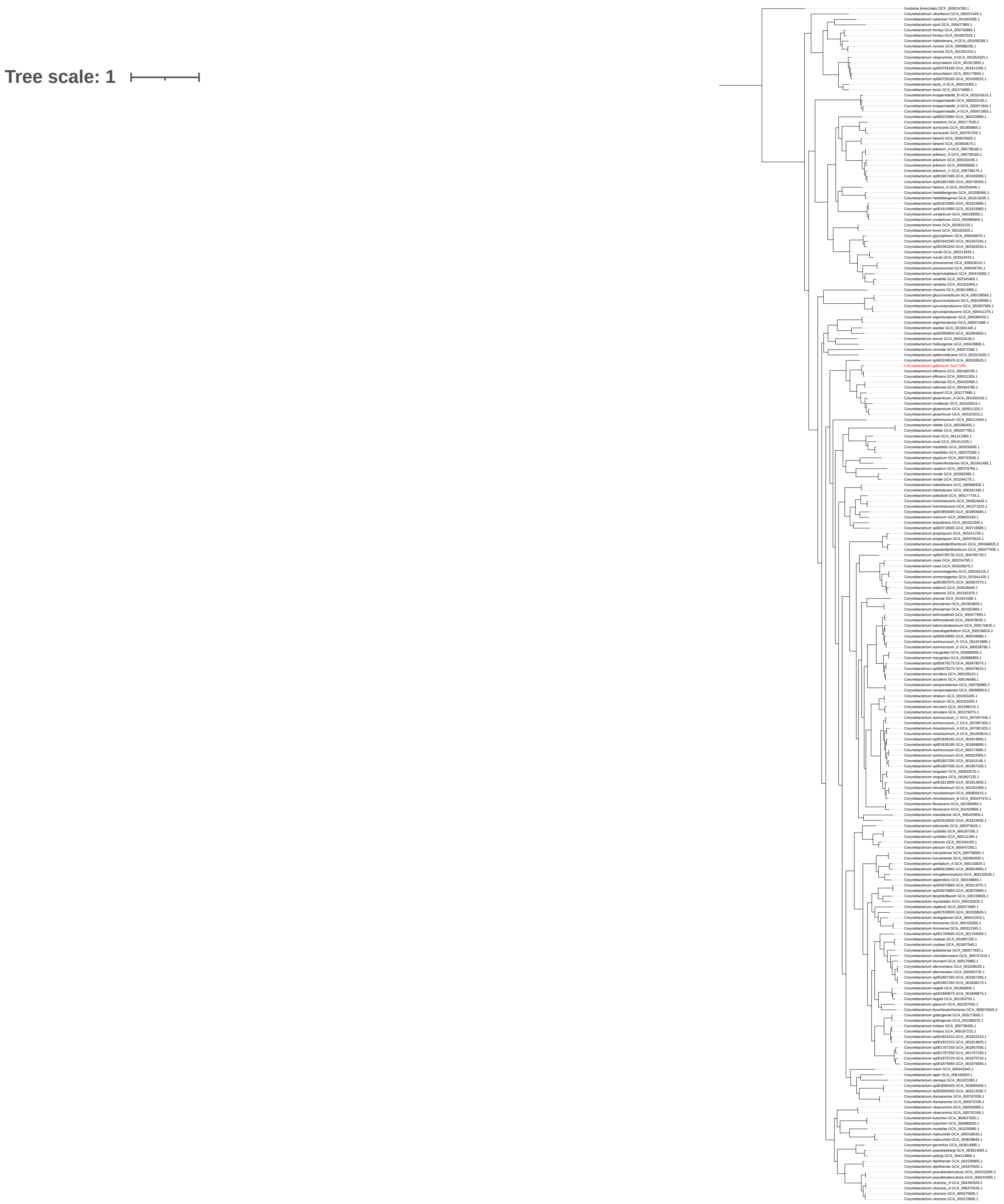
Tree scale: 1

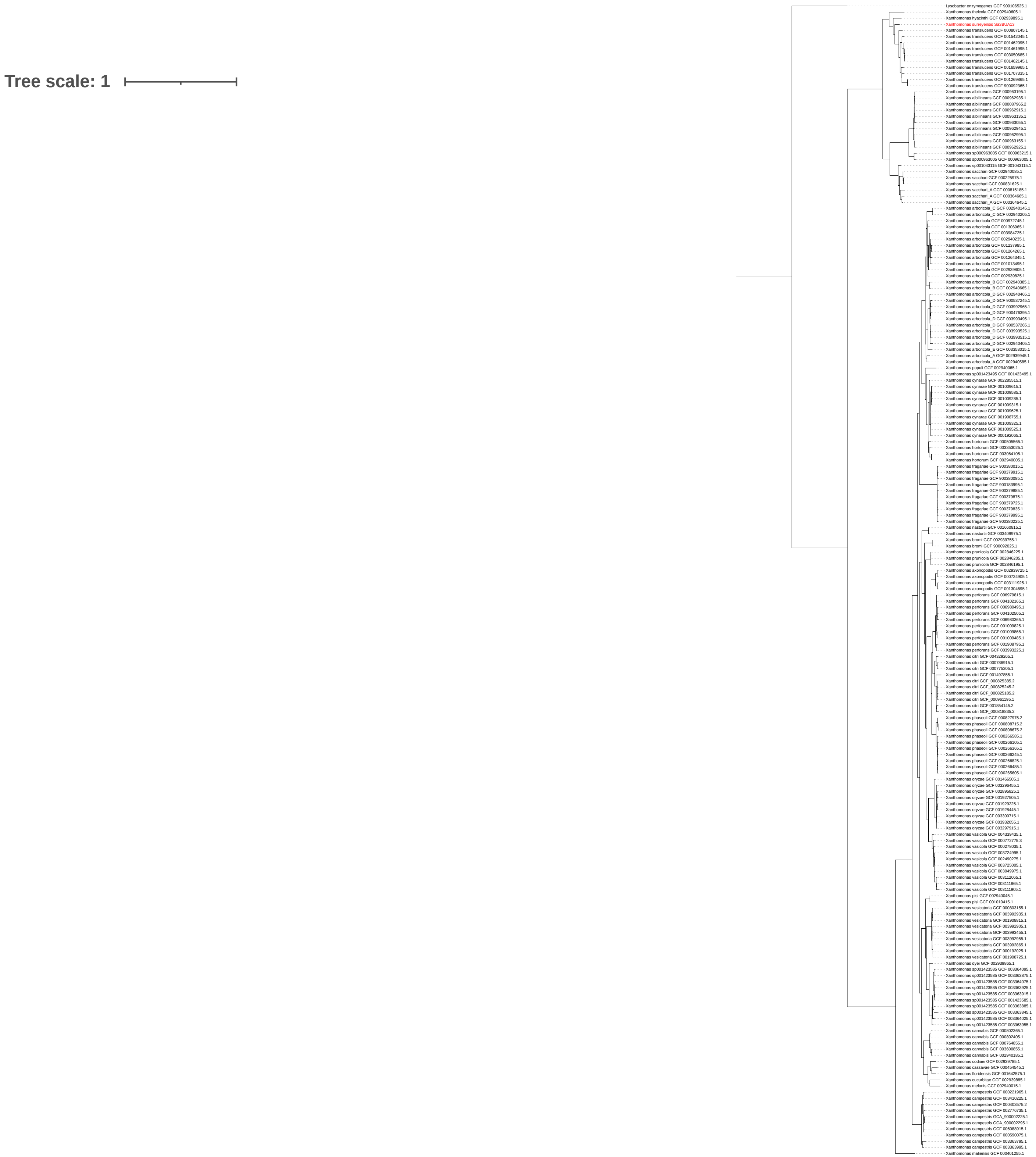
Tree scale: 1
Lysobacter enzymogenes GCF 900106525.1
Xanthomonas theicola GCF 002940605.1
Xanthomonas hyacinthi GCF 002939895.1
Xanthomonas surreyensis Sa3BUA13
Xanthomonas translucens GCF 000807145.1
Xanthomonas translucens GCF 001542045.1
Xanthomonas translucens GCF 001462095.1
Xanthomonas translucens GCF 001461995.1
Xanthomonas translucens GCF 003050685.1
Xanthomonas translucens GCF 001462145.1
Xanthomonas translucens GCF 001659965.1
Xanthomonas translucens GCF 001707335.1
Xanthomonas translucens GCF 001269865.1
Xanthomonas translucens GCF 900092365.1
Xanthomonas albilineans GCF 000963195.1
Xanthomonas albilineans GCF 000962935.1
Xanthomonas albilineans GCF 000087965.2
Xanthomonas albilineans GCF 000962915.1
Xanthomonas albilineans GCF 000963135.1
Xanthomonas albilineans GCF 000963055.1
Xanthomonas albilineans GCF 000962945.1
Xanthomonas albilineans GCF 000962995.1
Xanthomonas albilineans GCF 000963155.1
Xanthomonas albilineans GCF 000962925.1
Xanthomonas sp000963005 GCF 000963215.1
Xanthomonas sp000963005 GCF 000963005.1
Xanthomonas sp001043115 GCF 001043115.1
Xanthomonas sacchari GCF 002940085.1
Xanthomonas sacchari GCF 000225975.1
Xanthomonas sacchari GCF 000831625.1
Xanthomonas sacchari_A GCF 000815185.1
Xanthomonas sacchari_A GCF 000364665.1
Xanthomonas sacchari_A GCF 000364645.1
Xanthomonas arboricola_C GCF 002940145.1
Xanthomonas arboricola_C GCF 002940205.1
Xanthomonas arboricola GCF 000972745.1
Xanthomonas arboricola GCF 001306965.1
Xanthomonas arboricola GCF 003984725.1
Xanthomonas arboricola GCF 002940235.1
Xanthomonas arboricola GCF 001237985.1
Xanthomonas arboricola GCF 001264265.1
Xanthomonas arboricola GCF 001264345.1
Xanthomonas arboricola GCF 001013495.1
Xanthomonas arboricola GCF 002939805.1
Xanthomonas arboricola GCF 002939825.1
Xanthomonas arboricola_B GCF 002940385.1
Xanthomonas arboricola_B GCF 002940665.1
Xanthomonas arboricola_D GCF 002940465.1
Xanthomonas arboricola_D GCF 900537245.1
Xanthomonas arboricola_D GCF 003992965.1
Xanthomonas arboricola_D GCF 900476395.1
Xanthomonas arboricola_D GCF 003993495.1
Xanthomonas arboricola_D GCF 900537265.1
Xanthomonas arboricola_D GCF 003993525.1
Xanthomonas arboricola_D GCF 003993515.1
Xanthomonas arboricola_D GCF 002940405.1
Xanthomonas arboricola_E GCF 003353015.1
Xanthomonas arboricola_A GCF 002939945.1
Xanthomonas arboricola_A GCF 002940585.1
Xanthomonas populi GCF 002940065.1
Xanthomonas sp001423495 GCF 001423495.1
Xanthomonas cynarae GCF 002285515.1
Xanthomonas cynarae GCF 001009615.1
Xanthomonas cynarae GCF 001009585.1
Xanthomonas cynarae GCF 001009285.1
Xanthomonas cynarae GCF 001009315.1
Xanthomonas cynarae GCF 001009625.1
Xanthomonas cynarae GCF 001908755.1
Xanthomonas cynarae GCF 001009325.1
Xanthomonas cynarae GCF 001009525.1
Xanthomonas cynarae GCF 000192065.1
Xanthomonas hortorum GCF 000505565.1
Xanthomonas hortorum GCF 003353025.1
Xanthomonas hortorum GCF 003064105.1
Xanthomonas hortorum GCF 002940005.1
Xanthomonas fragariae GCF 900380015.1
Xanthomonas fragariae GCF 900379915.1
Xanthomonas fragariae GCF 900380085.1
Xanthomonas fragariae GCF 900183995.1
Xanthomonas fragariae GCF 900379885.1
Xanthomonas fragariae GCF 900379875.1
Xanthomonas fragariae GCF 900379725.1
Xanthomonas fragariae GCF 900379835.1
Xanthomonas fragariae GCF 900379995.1
Xanthomonas fragariae GCF 900380225.1
Xanthomonas nasturtii GCF 001660815.1
Xanthomonas nasturtii GCF 003409975.1
Xanthomonas bromi GCF 002939755.1
Xanthomonas bromi GCF 900092025.1
Xanthomonas prunicola GCF 002846225.1
Xanthomonas prunicola GCF 002846205.1
Xanthomonas prunicola GCF 002846195.1
Xanthomonas axonopodis GCF 002939725.1
Xanthomonas axonopodis GCF 000724905.1
Xanthomonas axonopodis GCF 003111925.1
Xanthomonas axonopodis GCF 001304695.1
Xanthomonas perforans GCF 006979815.1
Xanthomonas perforans GCF 004102165.1
Xanthomonas perforans GCF 006980495.1
Xanthomonas perforans GCF 004102505.1
Xanthomonas perforans GCF 006980365.1
Xanthomonas perforans GCF 001009825.1
Xanthomonas perforans GCF 001009865.1
Xanthomonas perforans GCF 001009485.1
Xanthomonas perforans GCF 001908795.1
Xanthomonas perforans GCF 003993225.1
Xanthomonas citri GCF 004329265.1
Xanthomonas citri GCF 000786915.1
Xanthomonas citri GCF 000775205.1
Xanthomonas citri GCF 001497855.1
Xanthomonas citri GCF_000825385.2
Xanthomonas citri GCF_000825245.2
Xanthomonas citri GCF_000825185.2
Xanthomonas citri GCF_000961195.1
Xanthomonas citri GCF 001854145.2
Xanthomonas citri GCF_000818835.2
Xanthomonas phaseoli GCF 000827975.2
Xanthomonas phaseoli GCF 000808715.2
Xanthomonas phaseoli GCF 000808675.2
Xanthomonas phaseoli GCF 000266585.1
Xanthomonas phaseoli GCF 000266105.1
Xanthomonas phaseoli GCF 000266365.1
Xanthomonas phaseoli GCF 000266245.1
Xanthomonas phaseoli GCF 000266825.1
Xanthomonas phaseoli GCF 000266485.1
Xanthomonas phaseoli GCF 000265605.1
Xanthomonas oryzae GCF 001466505.1
Xanthomonas oryzae GCF 003296455.1
Xanthomonas oryzae GCF 002895825.1
Xanthomonas oryzae GCF 001927505.1
Xanthomonas oryzae GCF 001929225.1
Xanthomonas oryzae GCF 001928445.1
Xanthomonas oryzae GCF 003300715.1
Xanthomonas oryzae GCF 003932055.1
Xanthomonas oryzae GCF 003297915.1
Xanthomonas vasicola GCF 004339435.1
Xanthomonas vasicola GCF 000772775.3
Xanthomonas vasicola GCF 000278035.1
Xanthomonas vasicola GCF 003724995.1
Xanthomonas vasicola GCF 002490275.1
Xanthomonas vasicola GCF 003725005.1
Xanthomonas vasicola GCF 003949975.1
Xanthomonas vasicola GCF 003112065.1
Xanthomonas vasicola GCF 003111865.1
Xanthomonas vasicola GCF 003111905.1
Xanthomonas pisi GCF 002940045.1
Xanthomonas pisi GCF 001010415.1
Xanthomonas vesicatoria GCF 000803155.1
Xanthomonas vesicatoria GCF 003992935.1
Xanthomonas vesicatoria GCF 001908815.1
Xanthomonas vesicatoria GCF 003992905.1
Xanthomonas vesicatoria GCF 003993455.1
Xanthomonas vesicatoria GCF 003992955.1
Xanthomonas vesicatoria GCF 003992865.1
Xanthomonas vesicatoria GCF 000192025.1
Xanthomonas vesicatoria GCF 001908725.1
Xanthomonas dyei GCF 002939865.1
Xanthomonas sp001423585 GCF 003364095.1
Xanthomonas sp001423585 GCF 003363875.1
Xanthomonas sp001423585 GCF 003364075.1
Xanthomonas sp001423585 GCF 003363925.1
Xanthomonas sp001423585 GCF 003363915.1
Xanthomonas sp001423585 GCF 001423585.1
Xanthomonas sp001423585 GCF 003363885.1
Xanthomonas sp001423585 GCF 003363845.1
Xanthomonas sp001423585 GCF 003364025.1
Xanthomonas sp001423585 GCF 003363955.1
Xanthomonas cannabis GCF 000802365.1
Xanthomonas cannabis GCF 000802405.1
Xanthomonas cannabis GCF 000764855.1
Xanthomonas cannabis GCF 003600855.1
Xanthomonas cannabis GCF 002940185.1
Xanthomonas codiaei GCF 002939785.1
Xanthomonas cassavae GCF 000454545.1
Xanthomonas floridensis GCF 001642575.1
Xanthomonas cucurbitae GCF 002939885.1
Xanthomonas melonis GCF 002940015.1
Xanthomonas campestris GCF 000221965.1
Xanthomonas campestris GCF 003410225.1
Xanthomonas campestris GCF 000403575.2
Xanthomonas campestris GCF 002776735.1
Xanthomonas campestris GCA_900002225.1
Xanthomonas campestris GCA_900002295.1
Xanthomonas campestris GCF 006088915.1
Xanthomonas campestris GCF 000590075.1
Xanthomonas campestris GCF 003363795.1
Xanthomonas campestris GCF 003363995.1
Xanthomonas maliensis GCF 000401255.1

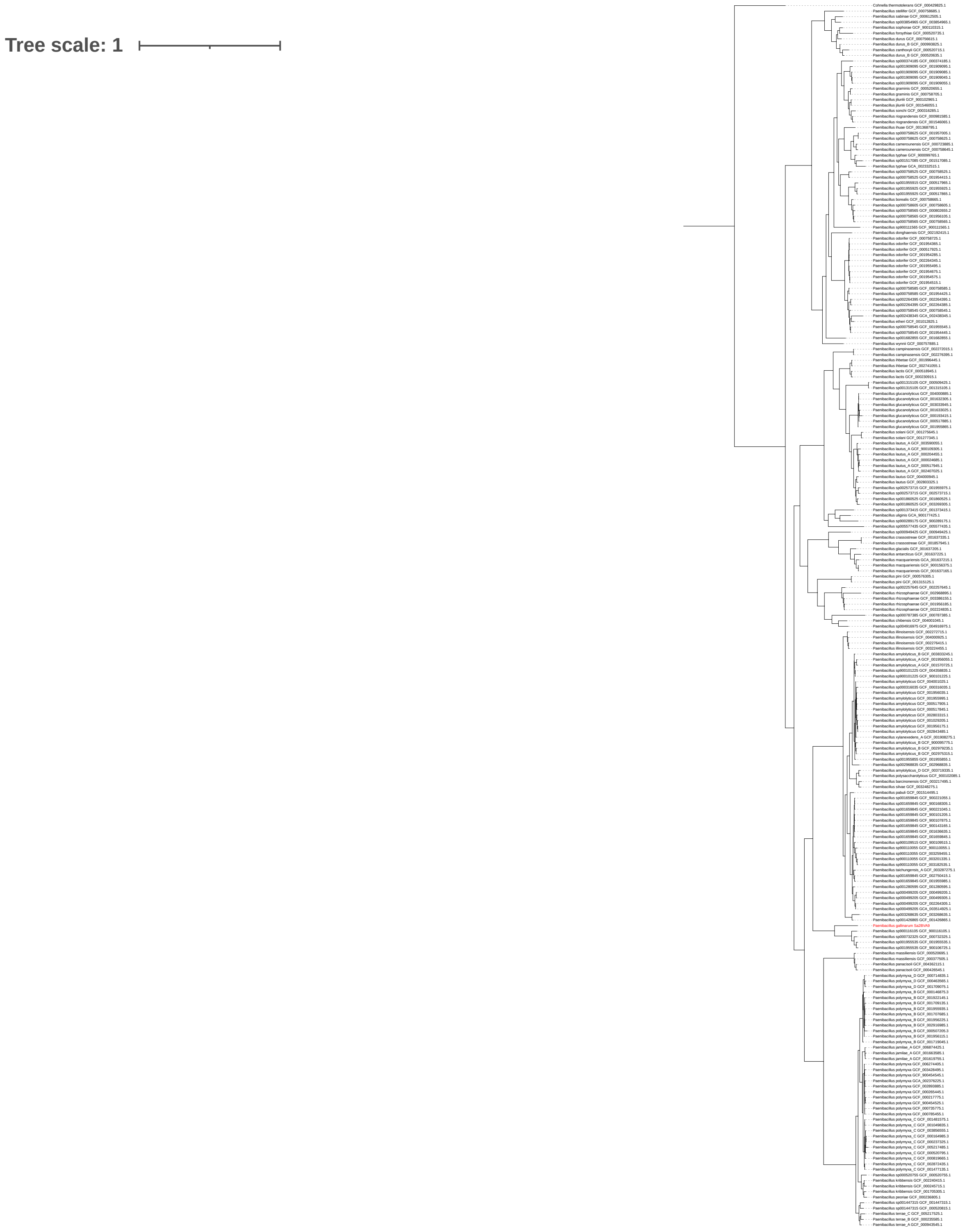
Tree scale: 1

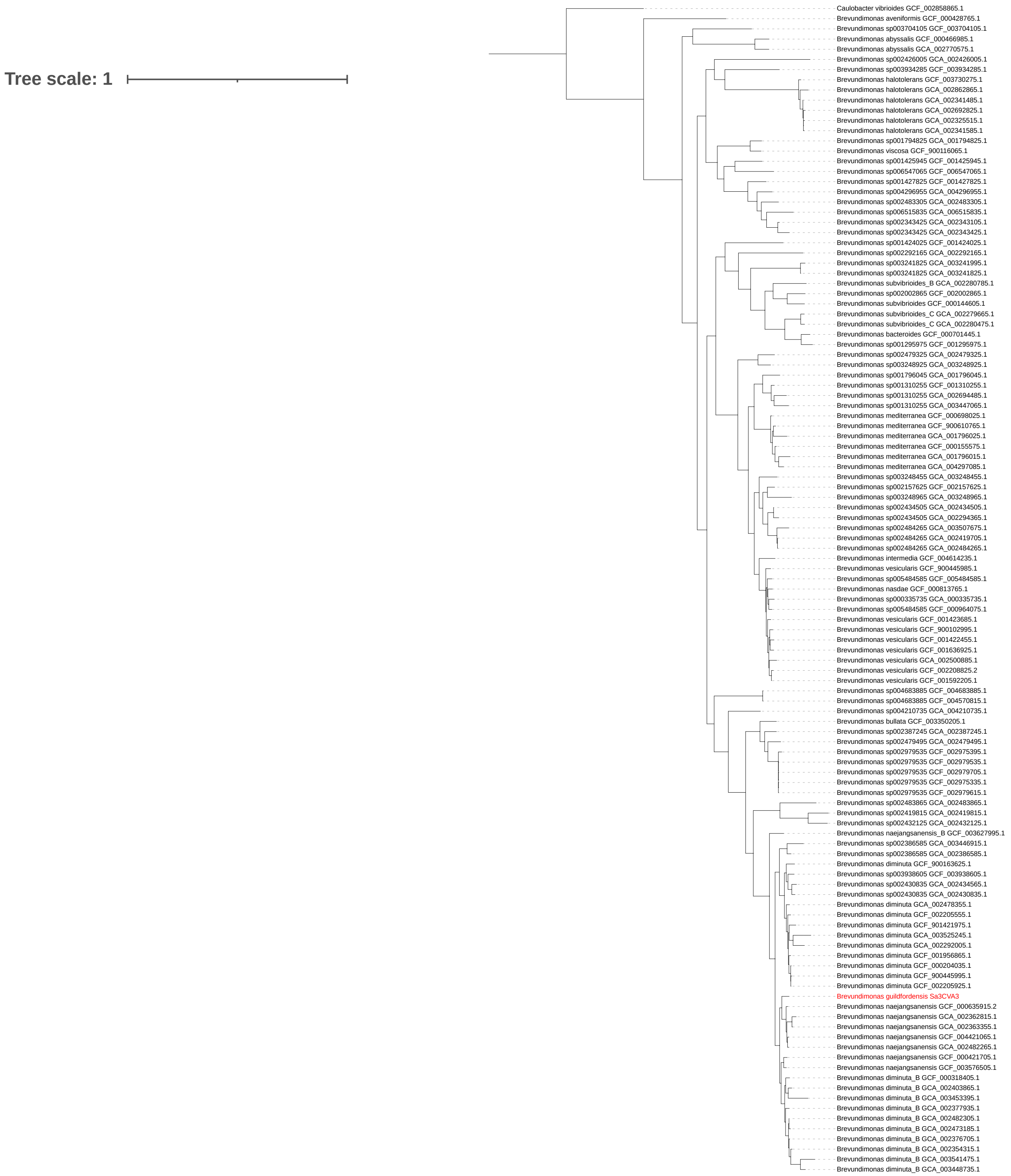

Tree scale: 1
Caulobacter vibrioides GCF_002858865.1
Brevundimonas aveniformis GCF_000428765.1
Brevundimonas sp003704105 GCF_003704105.1
Brevundimonas abyssalis GCF_000466985.1
Brevundimonas abyssalis GCA_002770575.1
Brevundimonas sp002426005 GCA_002426005.1
Brevundimonas sp003934285 GCF_003934285.1
Brevundimonas halotolerans GCF_003730275.1
Brevundimonas halotolerans GCA_002862865.1
Brevundimonas halotolerans GCA_002341485.1
Brevundimonas halotolerans GCA_002692825.1
Brevundimonas halotolerans GCA_002325515.1
Brevundimonas halotolerans GCA_002341585.1
Brevundimonas sp001794825 GCA_001794825.1
Brevundimonas viscosa GCF_900116065.1
Brevundimonas sp001425945 GCF_001425945.1
Brevundimonas sp006547065 GCF_006547065.1
Brevundimonas sp001427825 GCF_001427825.1
Brevundimonas sp004296955 GCA_004296955.1
Brevundimonas sp002483305 GCA_002483305.1
Brevundimonas sp006515835 GCA_006515835.1
Brevundimonas sp002343425 GCA_002343105.1
Brevundimonas sp002343425 GCA_002343425.1
Brevundimonas sp001424025 GCF_001424025.1
Brevundimonas sp002292165 GCA_002292165.1
Brevundimonas sp003241825 GCA_003241995.1
Brevundimonas sp003241825 GCA_003241825.1
Brevundimonas subvibrioides_B GCA_002280785.1
Brevundimonas sp002002865 GCF_002002865.1
Brevundimonas subvibrioides GCF_000144605.1
Brevundimonas subvibrioides_C GCA_002279665.1
Brevundimonas subvibrioides_C GCA_002280475.1
Brevundimonas bacteroides GCF_000701445.1
Brevundimonas sp001295975 GCF_001295975.1
Brevundimonas sp002479325 GCA_002479325.1
Brevundimonas sp003248925 GCA_003248925.1
Brevundimonas sp001796045 GCA_001796045.1
Brevundimonas sp001310255 GCF_001310255.1
Brevundimonas sp001310255 GCA_002694485.1
Brevundimonas sp001310255 GCA_003447065.1
Brevundimonas mediterranea GCF_000698025.1
Brevundimonas mediterranea GCF_900610765.1
Brevundimonas mediterranea GCA_001796025.1
Brevundimonas mediterranea GCF_000155575.1
Brevundimonas mediterranea GCA_001796015.1
Brevundimonas mediterranea GCA_004297085.1
Brevundimonas sp003248455 GCA_003248455.1
Brevundimonas sp002157625 GCF_002157625.1
Brevundimonas sp003248965 GCA_003248965.1
Brevundimonas sp002434505 GCA_002434505.1
Brevundimonas sp002434505 GCA_002294365.1
Brevundimonas sp002484265 GCA_003507675.1
Brevundimonas sp002484265 GCA_002419705.1
Brevundimonas sp002484265 GCA_002484265.1
Brevundimonas intermedia GCF_004614235.1
Brevundimonas vesicularis GCF_900445985.1
Brevundimonas sp005484585 GCF_005484585.1
Brevundimonas nasdae GCF_000813765.1
Brevundimonas sp000335735 GCA_000335735.1
Brevundimonas sp005484585 GCF_000964075.1
Brevundimonas vesicularis GCF_001423685.1
Brevundimonas vesicularis GCF_900102995.1
Brevundimonas vesicularis GCF_001422455.1
Brevundimonas vesicularis GCF_001636925.1
Brevundimonas vesicularis GCA_002500885.1
Brevundimonas vesicularis GCF_002208825.2
Brevundimonas vesicularis GCF_001592205.1
Brevundimonas sp004683885 GCF_004683885.1
Brevundimonas sp004683885 GCF_004570815.1
Brevundimonas sp004210735 GCA_004210735.1
Brevundimonas bullata GCF_003350205.1
Brevundimonas sp002387245 GCA_002387245.1
Brevundimonas sp002479495 GCA_002479495.1
Brevundimonas sp002979535 GCF_002975395.1
Brevundimonas sp002979535 GCF_002979535.1
Brevundimonas sp002979535 GCF_002979705.1
Brevundimonas sp002979535 GCF_002975335.1
Brevundimonas sp002979535 GCF_002979615.1
Brevundimonas sp002483865 GCA_002483865.1
Brevundimonas sp002419815 GCA_002419815.1
Brevundimonas sp002432125 GCA_002432125.1
Brevundimonas naejangsanensis_B GCF_003627995.1
Brevundimonas sp002386585 GCA_003446915.1
Brevundimonas sp002386585 GCA_002386585.1
Brevundimonas diminuta GCF_900163625.1
Brevundimonas sp003938605 GCF_003938605.1
Brevundimonas sp002430835 GCA_002434565.1
Brevundimonas sp002430835 GCA_002430835.1
Brevundimonas diminuta GCA_002478355.1
Brevundimonas diminuta GCF_002205555.1
Brevundimonas diminuta GCF_901421975.1
Brevundimonas diminuta GCA_003525245.1
Brevundimonas diminuta GCA_002292005.1
Brevundimonas diminuta GCF_001956865.1
Brevundimonas diminuta GCF_000204035.1
Brevundimonas diminuta GCF_900445995.1
Brevundimonas diminuta GCF_002205925.1
Brevundimonas guildfordensis Sa3CVA3
Brevundimonas naejangsanensis GCF_000635915.2
Brevundimonas naejangsanensis GCA_002362815.1
Brevundimonas naejangsanensis GCA_002363355.1
Brevundimonas naejangsanensis GCF_004421065.1
Brevundimonas naejangsanensis GCA_002482265.1
Brevundimonas naejangsanensis GCF_000421705.1
Brevundimonas naejangsanensis GCF_003576505.1
Brevundimonas diminuta_B GCF_000318405.1
Brevundimonas diminuta_B GCA_002403865.1
Brevundimonas diminuta_B GCA_003453395.1
Brevundimonas diminuta_B GCA_002377935.1
Brevundimonas diminuta_B GCA_002482305.1
Brevundimonas diminuta_B GCA_002473185.1
Brevundimonas diminuta_B GCA_002376705.1
Brevundimonas diminuta_B GCA_002354315.1
Brevundimonas diminuta_B GCA_003541475.1
Brevundimonas diminuta_B GCA_003448735.1

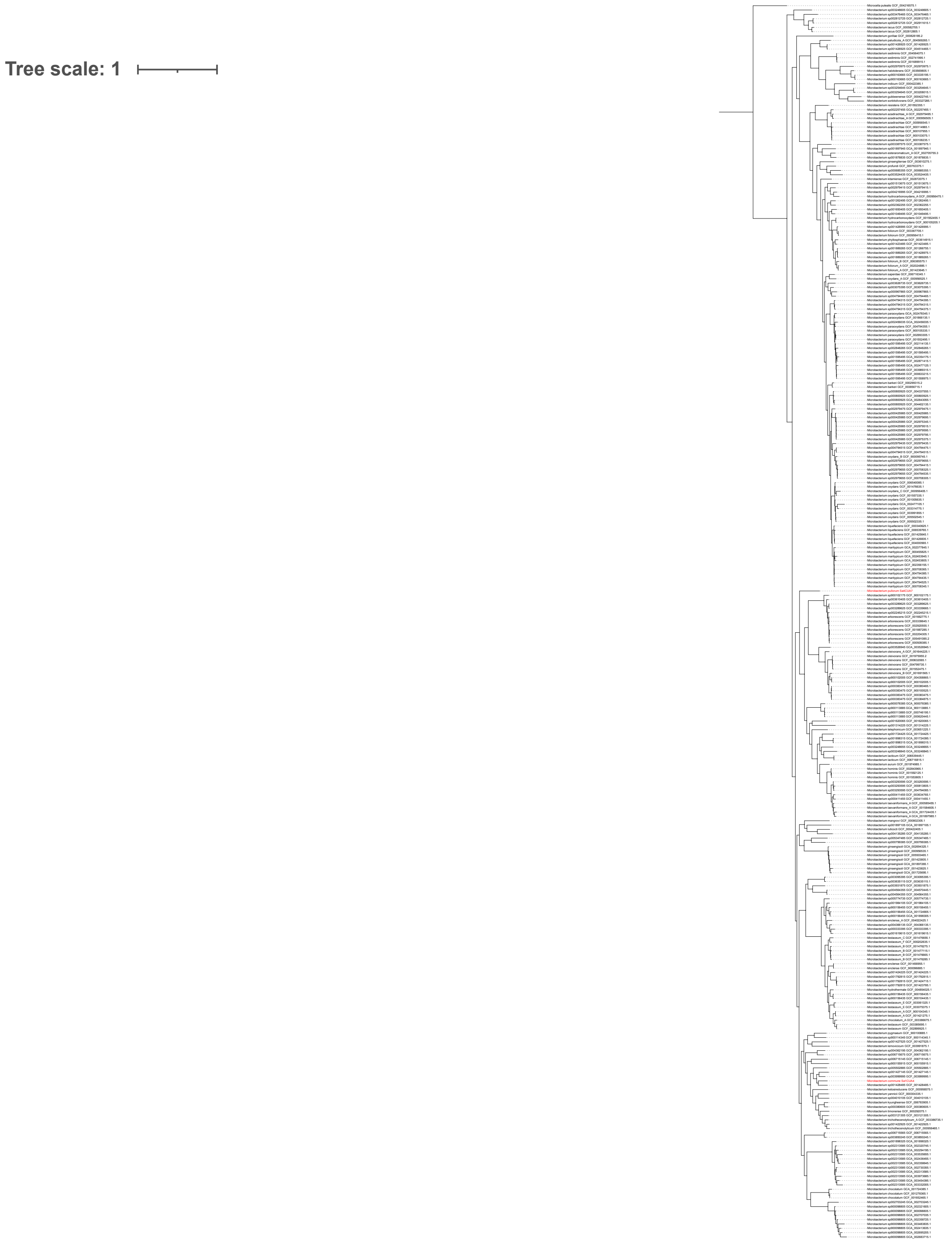
Tree scale: 1

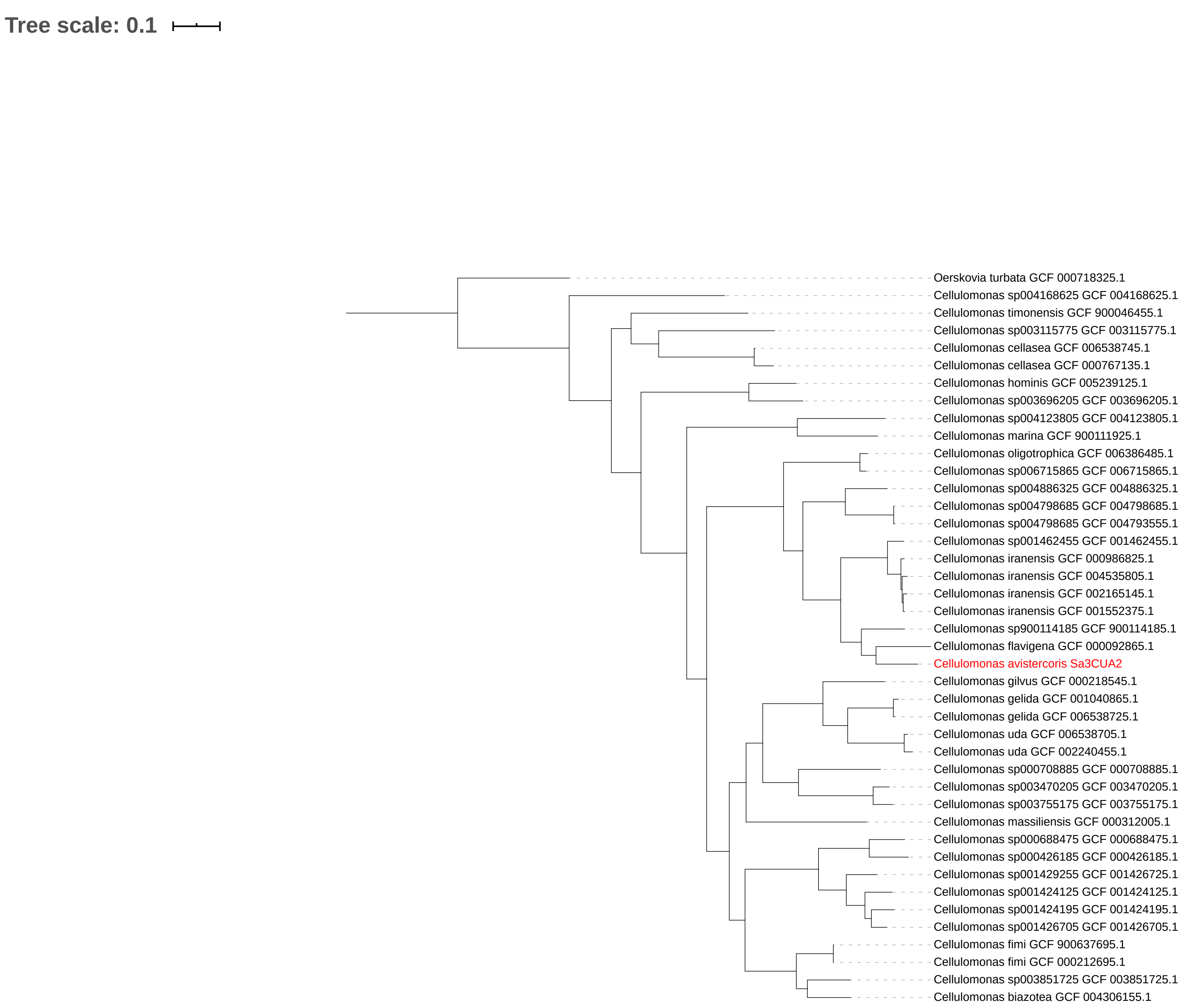
Tree scale: 0.1
Oerskovia turbata GCF 000718325.1
Cellulomonas sp004168625 GCF 004168625.1
Cellulomonas timonensis GCF 900046455.1
Cellulomonas sp003115775 GCF 003115775.1
Cellulomonas cellasea GCF 006538745.1
Cellulomonas cellasea GCF 000767135.1
Cellulomonas hominis GCF 005239125.1
Cellulomonas sp003696205 GCF 003696205.1
Cellulomonas sp004123805 GCF 004123805.1
Cellulomonas marina GCF 900111925.1
Cellulomonas oligotrophica GCF 006386485.1
Cellulomonas sp006715865 GCF 006715865.1
Cellulomonas sp004886325 GCF 004886325.1
Cellulomonas sp004798685 GCF 004798685.1
Cellulomonas sp004798685 GCF 004793555.1
Cellulomonas sp001462455 GCF 001462455.1
Cellulomonas iranensis GCF 000986825.1
Cellulomonas iranensis GCF 004535805.1
Cellulomonas iranensis GCF 002165145.1
Cellulomonas iranensis GCF 001552375.1
Cellulomonas sp900114185 GCF 900114185.1
Cellulomonas flavigena GCF 000092865.1
Cellulomonas avistercoris Sa3CUA2
Cellulomonas gilvus GCF 000218545.1
Cellulomonas gelida GCF 001040865.1
Cellulomonas gelida GCF 006538725.1
Cellulomonas uda GCF 006538705.1
Cellulomonas uda GCF 002240455.1
Cellulomonas sp000708885 GCF 000708885.1
Cellulomonas sp003470205 GCF 003470205.1
Cellulomonas sp003755175 GCF 003755175.1
Cellulomonas massiliensis GCF 000312005.1
Cellulomonas sp000688475 GCF 000688475.1
Cellulomonas sp000426185 GCF 000426185.1
Cellulomonas sp001429255 GCF 001426725.1
Cellulomonas sp001424125 GCF 001424125.1
Cellulomonas sp001424195 GCF 001424195.1
Cellulomonas sp001426705 GCF 001426705.1
Cellulomonas fimi GCF 900637695.1
Cellulomonas fimi GCF 000212695.1
Cellulomonas sp003851725 GCF 003851725.1
Cellulomonas biazotea GCF 004306155.1

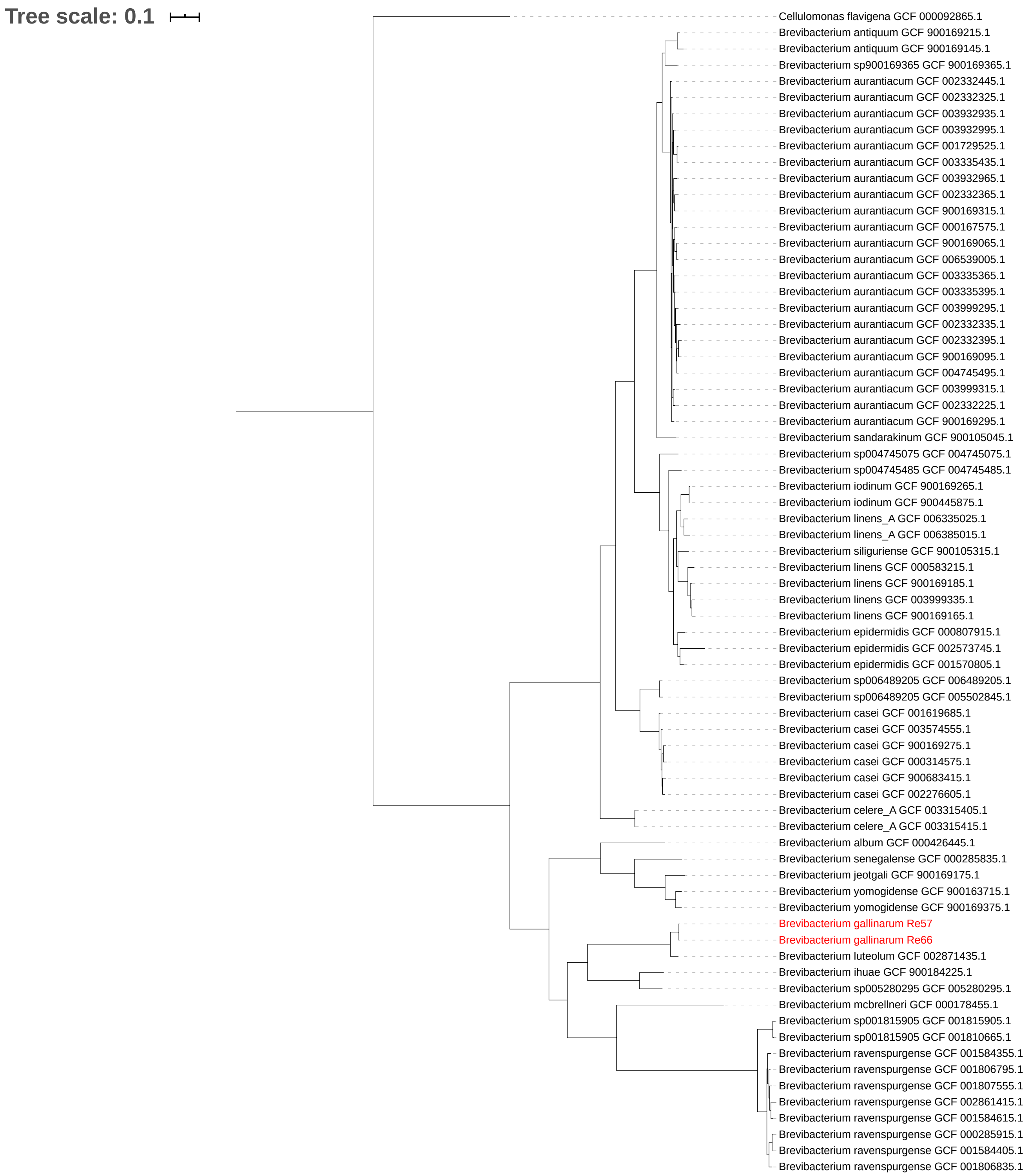
Tree scale: 0.1
Cellulomonas flavigena GCF 000092865.1
Brevibacterium antiquum GCF 900169215.1
Brevibacterium antiquum GCF 900169145.1
Brevibacterium sp900169365 GCF 900169365.1
Brevibacterium aurantiacum GCF 002332445.1
Brevibacterium aurantiacum GCF 002332325.1
Brevibacterium aurantiacum GCF 003932935.1
Brevibacterium aurantiacum GCF 003932995.1
Brevibacterium aurantiacum GCF 001729525.1
Brevibacterium aurantiacum GCF 003335435.1
Brevibacterium aurantiacum GCF 003932965.1
Brevibacterium aurantiacum GCF 002332365.1
Brevibacterium aurantiacum GCF 900169315.1
Brevibacterium aurantiacum GCF 000167575.1
Brevibacterium aurantiacum GCF 900169065.1
Brevibacterium aurantiacum GCF 006539005.1
Brevibacterium aurantiacum GCF 003335365.1
Brevibacterium aurantiacum GCF 003335395.1
Brevibacterium aurantiacum GCF 003999295.1
Brevibacterium aurantiacum GCF 002332335.1
Brevibacterium aurantiacum GCF 002332395.1
Brevibacterium aurantiacum GCF 900169095.1
Brevibacterium aurantiacum GCF 004745495.1
Brevibacterium aurantiacum GCF 003999315.1
Brevibacterium aurantiacum GCF 002332225.1
Brevibacterium aurantiacum GCF 900169295.1
Brevibacterium sandarakinum GCF 900105045.1
Brevibacterium sp004745075 GCF 004745075.1
Brevibacterium sp004745485 GCF 004745485.1
Brevibacterium iodinum GCF 900169265.1
Brevibacterium iodinum GCF 900445875.1
Brevibacterium linens_A GCF 006335025.1
Brevibacterium linens_A GCF 006385015.1
Brevibacterium siliguriense GCF 900105315.1
Brevibacterium linens GCF 000583215.1
Brevibacterium linens GCF 900169185.1
Brevibacterium linens GCF 003999335.1
Brevibacterium linens GCF 900169165.1
Brevibacterium epidermidis GCF 000807915.1
Brevibacterium epidermidis GCF 002573745.1
Brevibacterium epidermidis GCF 001570805.1
Brevibacterium sp006489205 GCF 006489205.1
Brevibacterium sp006489205 GCF 005502845.1
Brevibacterium casei GCF 001619685.1
Brevibacterium casei GCF 003574555.1
Brevibacterium casei GCF 900169275.1
Brevibacterium casei GCF 000314575.1
Brevibacterium casei GCF 900683415.1
Brevibacterium casei GCF 002276605.1
Brevibacterium celere_A GCF 003315405.1
Brevibacterium celere_A GCF 003315415.1
Brevibacterium album GCF 000426445.1
Brevibacterium senegalense GCF 000285835.1
Brevibacterium jeotgali GCF 900169175.1
Brevibacterium yomogidense GCF 900163715.1
Brevibacterium yomogidense GCF 900169375.1
Brevibacterium gallinarum Re57
Brevibacterium gallinarum Re66
Brevibacterium luteolum GCF 002871435.1
Brevibacterium ihuae GCF 900184225.1
Brevibacterium sp005280295 GCF 005280295.1
Brevibacterium mcbrellneri GCF 000178455.1
Brevibacterium sp001815905 GCF 001815905.1
Brevibacterium sp001815905 GCF 001810665.1
Brevibacterium ravenspurgense GCF 001584355.1
Brevibacterium ravenspurgense GCF 001806795.1
Brevibacterium ravenspurgense GCF 001807555.1
Brevibacterium ravenspurgense GCF 002861415.1
Brevibacterium ravenspurgense GCF 001584615.1
Brevibacterium ravenspurgense GCF 000285915.1
Brevibacterium ravenspurgense GCF 001584405.1
Brevibacterium ravenspurgense GCF 001806835.1

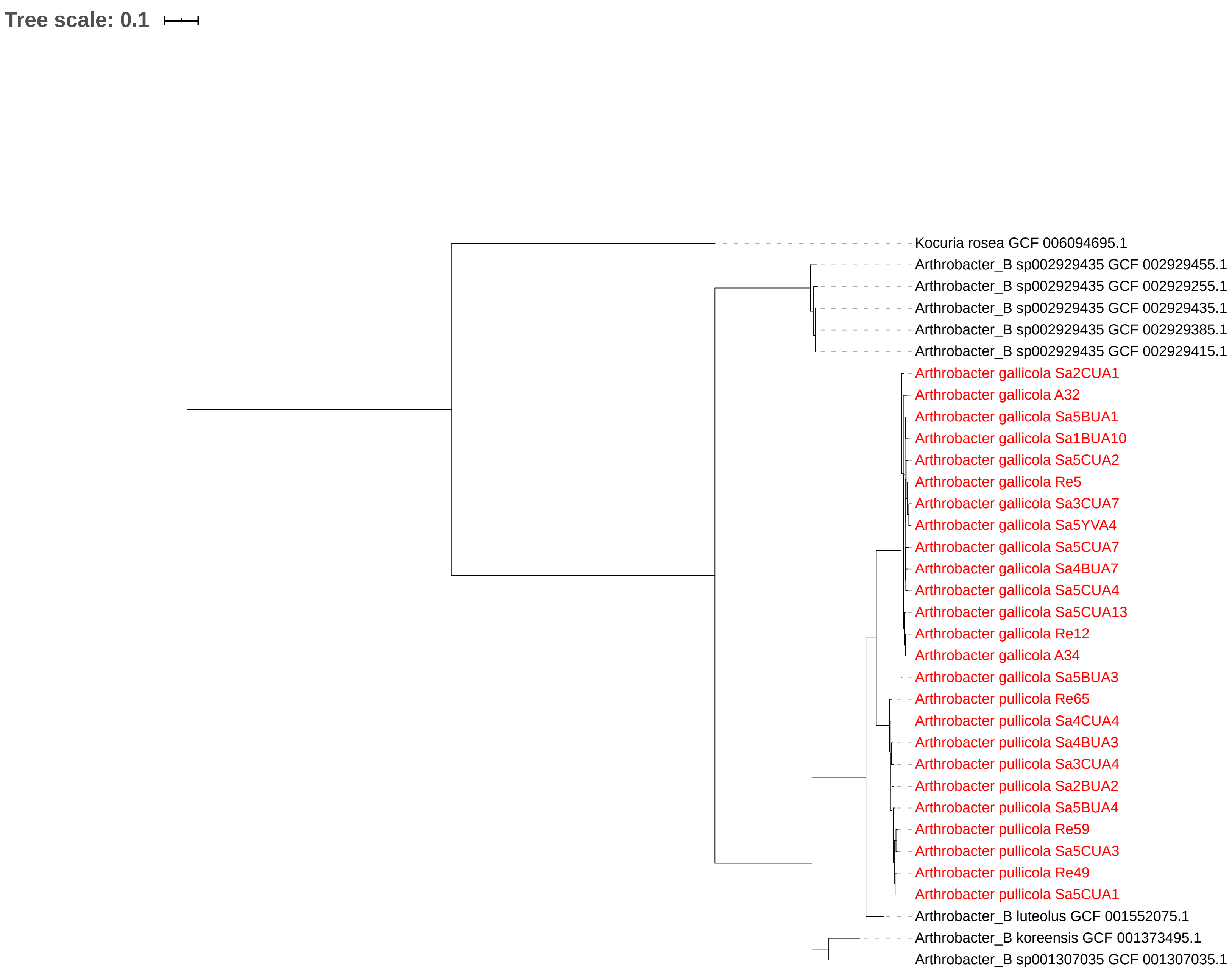

Tree scale: 0.1
Kocuria rosea GCF 006094695.1
Arthrobacter_B sp002929435 GCF 002929455.1
Arthrobacter_B sp002929435 GCF 002929255.1
Arthrobacter_B sp002929435 GCF 002929435.1
Arthrobacter_B sp002929435 GCF 002929385.1
Arthrobacter_B sp002929435 GCF 002929415.1
Arthrobacter gallicola Sa2CUA1
Arthrobacter gallicola A32
Arthrobacter gallicola Sa5BUA1
Arthrobacter gallicola Sa1BUA10
Arthrobacter gallicola Sa5CUA2
Arthrobacter gallicola Re5
Arthrobacter gallicola Sa3CUA7
Arthrobacter gallicola Sa5YVA4
Arthrobacter gallicola Sa5CUA7
Arthrobacter gallicola Sa4BUA7
Arthrobacter gallicola Sa5CUA4
Arthrobacter gallicola Sa5CUA13
Arthrobacter gallicola Re12
Arthrobacter gallicola A34
Arthrobacter gallicola Sa5BUA3
Arthrobacter pullicola Re65
Arthrobacter pullicola Sa4CUA4
Arthrobacter pullicola Sa4BUA3
Arthrobacter pullicola Sa3CUA4
Arthrobacter pullicola Sa2BUA2
Arthrobacter pullicola Sa5BUA4
Arthrobacter pullicola Re59
Arthrobacter pullicola Sa5CUA3
Arthrobacter pullicola Re49
Arthrobacter pullicola Sa5CUA1
Arthrobacter_B luteolus GCF 001552075.1
Arthrobacter_B koreensis GCF 001373495.1
Arthrobacter_B sp001307035 GCF 001307035.1

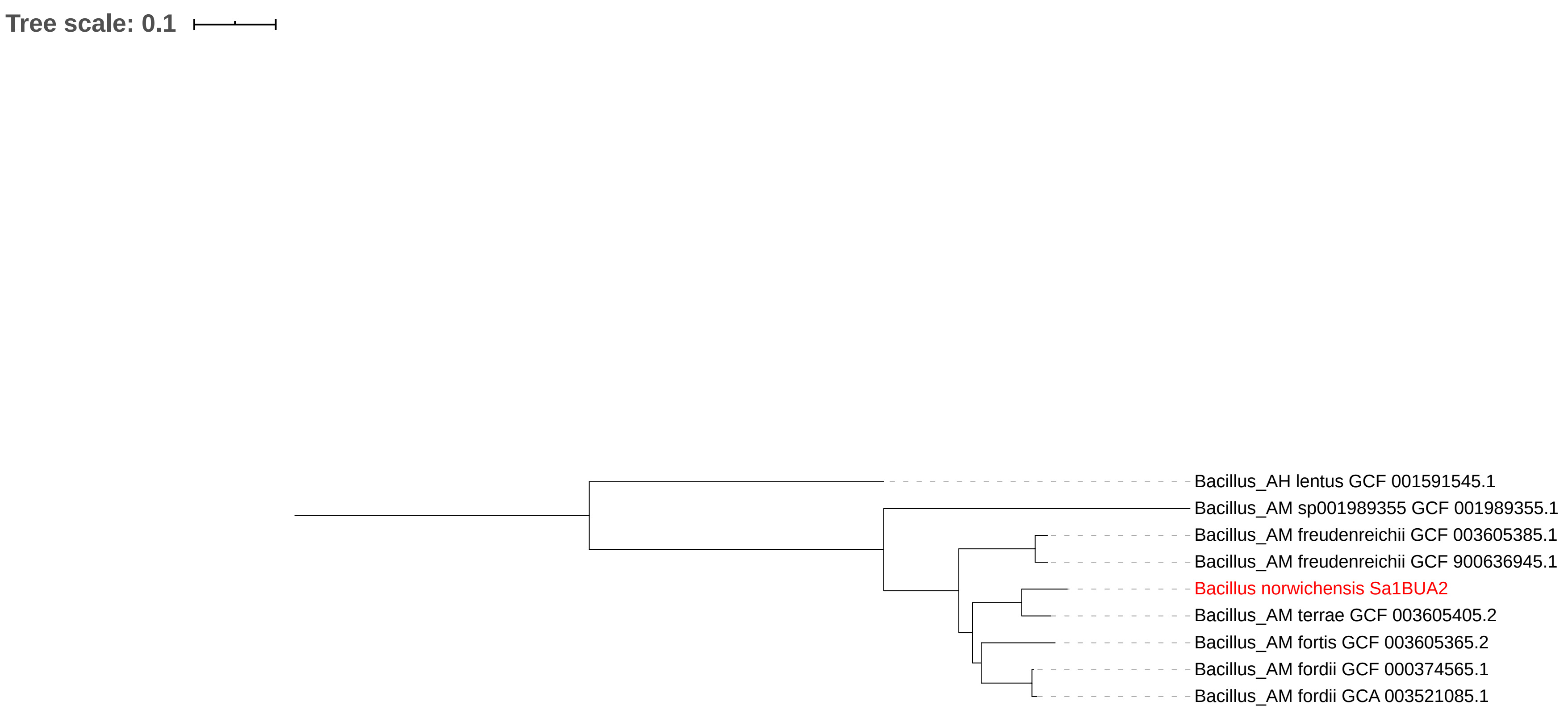
Tree scale: 0.1
Bacillus_AH lentus GCF 001591545.1
Bacillus_AM sp001989355 GCF 001989355.1
Bacillus_AM freudenreichii GCF 003605385.1
Bacillus_AM freudenreichii GCF 900636945.1
Bacillus norwichensis Sa1BUA2
Bacillus_AM terrae GCF 003605405.2
Bacillus_AM fortis GCF 003605365.2
Bacillus_AM fordii GCF 000374565.1
Bacillus_AM fordii GCA 003521085.1

Tree scale:

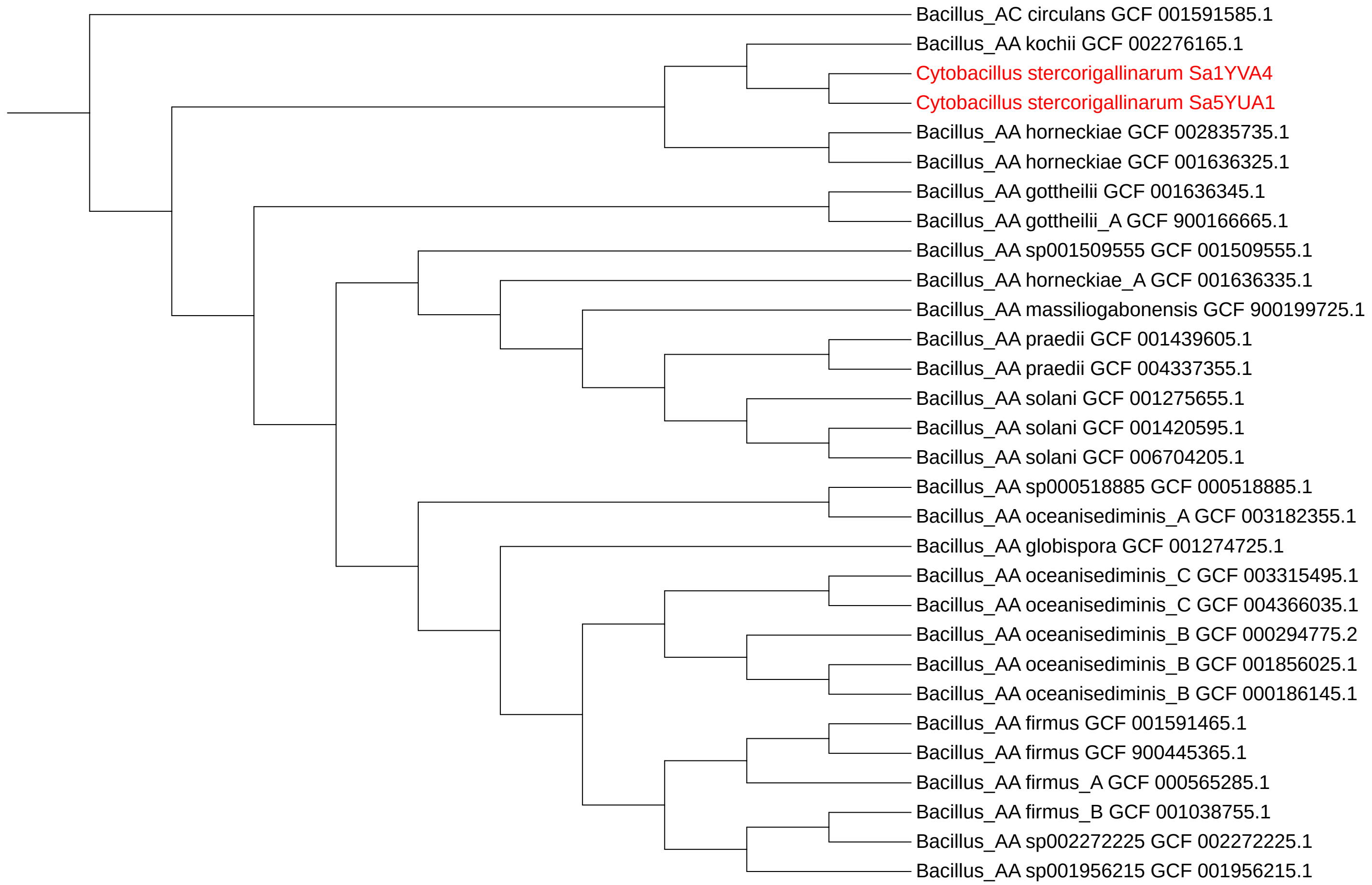
Bacillus_AC circulans GCF 001591585.1
Bacillus_AA kochii GCF 002276165.1
Cytobacillus stercorigallinarum Sa1YVA4
Cytobacillus stercorigallinarum Sa5YUA1
Bacillus_AA horneckiae GCF 002835735.1
Bacillus_AA horneckiae GCF 001636325.1
Bacillus_AA gottheilii GCF 001636345.1
Bacillus_AA gottheilii_A GCF 900166665.1
Bacillus_AA sp001509555 GCF 001509555.1
Bacillus_AA horneckiae_A GCF 001636335.1
Bacillus_AA massiliogabonensis GCF 900199725.1
Bacillus_AA praedii GCF 001439605.1
Bacillus_AA praedii GCF 004337355.1
Bacillus_AA solani GCF 001275655.1
Bacillus_AA solani GCF 001420595.1
Bacillus_AA solani GCF 006704205.1
Bacillus_AA sp000518885 GCF 000518885.1
Bacillus_AA oceanisediminis_A GCF 003182355.1
Bacillus_AA globispora GCF 001274725.1
Bacillus_AA oceanisediminis_C GCF 003315495.1
Bacillus_AA oceanisediminis_C GCF 004366035.1
Bacillus_AA oceanisediminis_B GCF 000294775.2
Bacillus_AA oceanisediminis_B GCF 001856025.1
Bacillus_AA oceanisediminis_B GCF 000186145.1
Bacillus_AA firmus GCF 001591465.1
Bacillus_AA firmus GCF 900445365.1
Bacillus_AA firmus_A GCF 000565285.1
Bacillus_AA firmus_B GCF 001038755.1
Bacillus_AA sp002272225 GCF 002272225.1
Bacillus_AA sp001956215 GCF 001956215.1

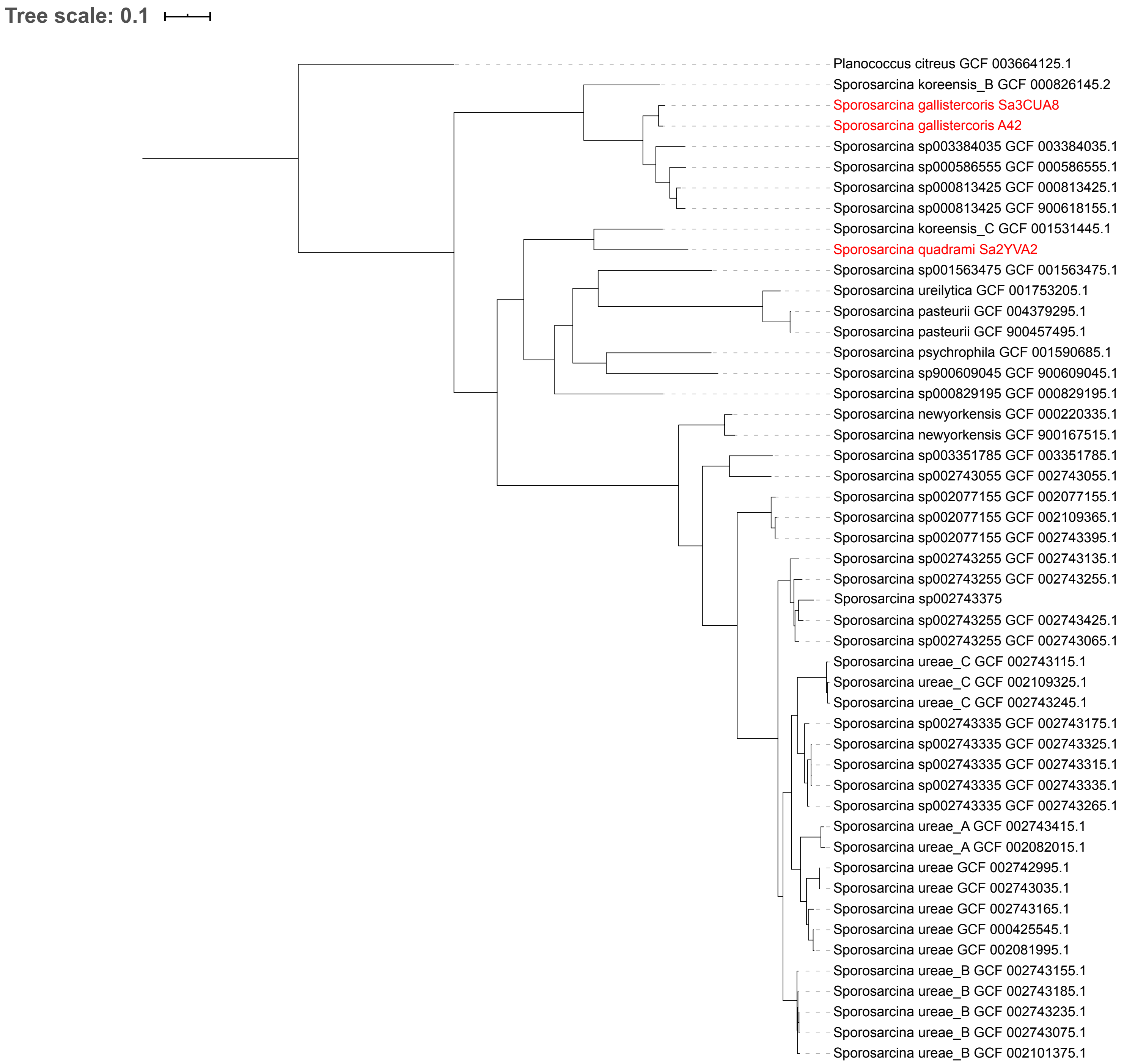
Tree scale: 0.1
Planococcus citreus GCF 003664125.1
Sporosarcina koreensis_B GCF 000826145.2
Sporosarcina gallistercoris Sa3CUA8
Sporosarcina gallistercoris A42
Sporosarcina sp003384035 GCF 003384035.1
Sporosarcina sp000586555 GCF 000586555.1
Sporosarcina sp000813425 GCF 000813425.1
Sporosarcina sp000813425 GCF 900618155.1
Sporosarcina koreensis_C GCF 001531445.1
Sporosarcina quadrami Sa2YVA2
Sporosarcina sp001563475 GCF 001563475.1
Sporosarcina ureilytica GCF 001753205.1
Sporosarcina pasteurii GCF 004379295.1
Sporosarcina pasteurii GCF 900457495.1
Sporosarcina psychrophila GCF 001590685.1
Sporosarcina sp900609045 GCF 900609045.1
Sporosarcina sp000829195 GCF 000829195.1
Sporosarcina newyorkensis GCF 000220335.1
Sporosarcina newyorkensis GCF 900167515.1
Sporosarcina sp003351785 GCF 003351785.1
Sporosarcina sp002743055 GCF 002743055.1
Sporosarcina sp002077155 GCF 002077155.1
Sporosarcina sp002077155 GCF 002109365.1
Sporosarcina sp002077155 GCF 002743395.1
Sporosarcina sp002743255 GCF 002743135.1
Sporosarcina sp002743255 GCF 002743255.1
Sporosarcina sp002743375
Sporosarcina sp002743255 GCF 002743425.1
Sporosarcina sp002743255 GCF 002743065.1
Sporosarcina ureae_C GCF 002743115.1
Sporosarcina ureae_C GCF 002109325.1
Sporosarcina ureae_C GCF 002743245.1
Sporosarcina sp002743335 GCF 002743175.1
Sporosarcina sp002743335 GCF 002743325.1
Sporosarcina sp002743335 GCF 002743315.1
Sporosarcina sp002743335 GCF 002743335.1
Sporosarcina sp002743335 GCF 002743265.1
Sporosarcina ureae_A GCF 002743415.1
Sporosarcina ureae_A GCF 002082015.1
Sporosarcina ureae GCF 002742995.1
Sporosarcina ureae GCF 002743035.1
Sporosarcina ureae GCF 002743165.1
Sporosarcina ureae GCF 000425545.1
Sporosarcina ureae GCF 002081995.1
Sporosarcina ureae_B GCF 002743155.1
Sporosarcina ureae_B GCF 002743185.1
Sporosarcina ureae_B GCF 002743235.1
Sporosarcina ureae_B GCF 002743075.1
Sporosarcina ureae_B GCF 002101375.1

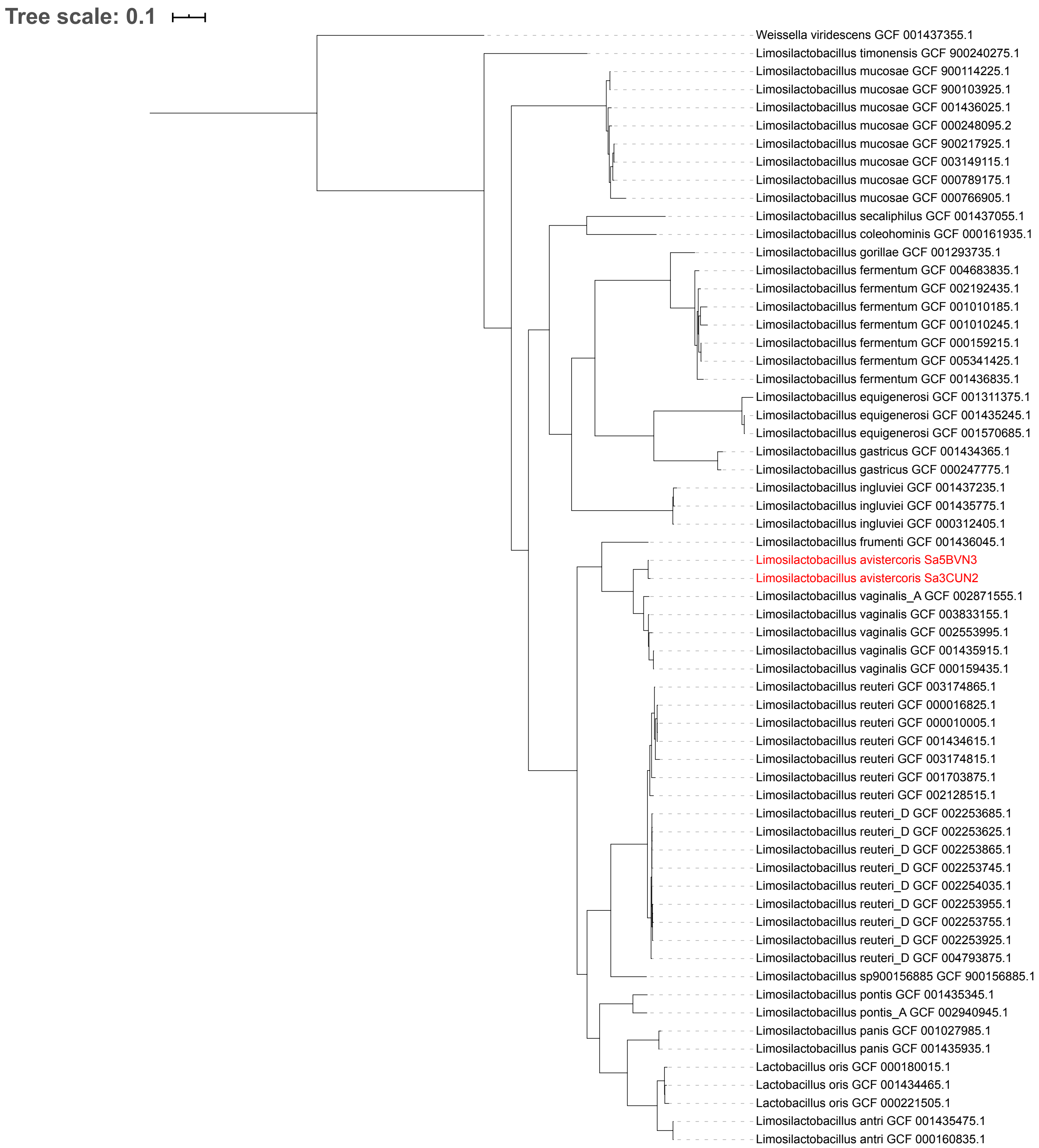
Tree scale: 0.1
Weissella viridescens GCF 001437355.1
Limosilactobacillus timonensis GCF 900240275.1
Limosilactobacillus mucosae GCF 900114225.1
Limosilactobacillus mucosae GCF 900103925.1
Limosilactobacillus mucosae GCF 001436025.1
Limosilactobacillus mucosae GCF 000248095.2
Limosilactobacillus mucosae GCF 900217925.1
Limosilactobacillus mucosae GCF 003149115.1
Limosilactobacillus mucosae GCF 000789175.1
Limosilactobacillus mucosae GCF 000766905.1
Limosilactobacillus secaliphilus GCF 001437055.1
Limosilactobacillus coleohominis GCF 000161935.1
Limosilactobacillus gorillae GCF 001293735.1
Limosilactobacillus fermentum GCF 004683835.1
Limosilactobacillus fermentum GCF 002192435.1
Limosilactobacillus fermentum GCF 001010185.1
Limosilactobacillus fermentum GCF 001010245.1
Limosilactobacillus fermentum GCF 000159215.1
Limosilactobacillus fermentum GCF 005341425.1
Limosilactobacillus fermentum GCF 001436835.1
Limosilactobacillus equigenerosi GCF 001311375.1
Limosilactobacillus equigenerosi GCF 001435245.1
Limosilactobacillus equigenerosi GCF 001570685.1
Limosilactobacillus gastricus GCF 001434365.1
Limosilactobacillus gastricus GCF 000247775.1
Limosilactobacillus ingluviei GCF 001437235.1
Limosilactobacillus ingluviei GCF 001435775.1
Limosilactobacillus ingluviei GCF 000312405.1
Limosilactobacillus frumenti GCF 001436045.1
Limosilactobacillus avistercoris Sa5BVN3
Limosilactobacillus avistercoris Sa3CUN2
Limosilactobacillus vaginalis_A GCF 002871555.1
Limosilactobacillus vaginalis GCF 003833155.1
Limosilactobacillus vaginalis GCF 002553995.1
Limosilactobacillus vaginalis GCF 001435915.1
Limosilactobacillus vaginalis GCF 000159435.1
Limosilactobacillus reuteri GCF 003174865.1
Limosilactobacillus reuteri GCF 000016825.1
Limosilactobacillus reuteri GCF 000010005.1
Limosilactobacillus reuteri GCF 001434615.1
Limosilactobacillus reuteri GCF 003174815.1
Limosilactobacillus reuteri GCF 001703875.1
Limosilactobacillus reuteri GCF 002128515.1
Limosilactobacillus reuteri_D GCF 002253685.1
Limosilactobacillus reuteri_D GCF 002253625.1
Limosilactobacillus reuteri_D GCF 002253865.1
Limosilactobacillus reuteri_D GCF 002253745.1
Limosilactobacillus reuteri_D GCF 002254035.1
Limosilactobacillus reuteri_D GCF 002253955.1
Limosilactobacillus reuteri_D GCF 002253755.1
Limosilactobacillus reuteri_D GCF 002253925.1
Limosilactobacillus reuteri_D GCF 004793875.1
Limosilactobacillus sp900156885 GCF 900156885.1
Limosilactobacillus pontis GCF 001435345.1
Limosilactobacillus pontis_A GCF 002940945.1
Limosilactobacillus panis GCF 001027985.1
Limosilactobacillus panis GCF 001435935.1
Lactobacillus oris GCF 000180015.1
Lactobacillus oris GCF 001434465.1
Lactobacillus oris GCF 000221505.1
Limosilactobacillus antri GCF 001435475.1
Limosilactobacillus antri GCF 000160835.1

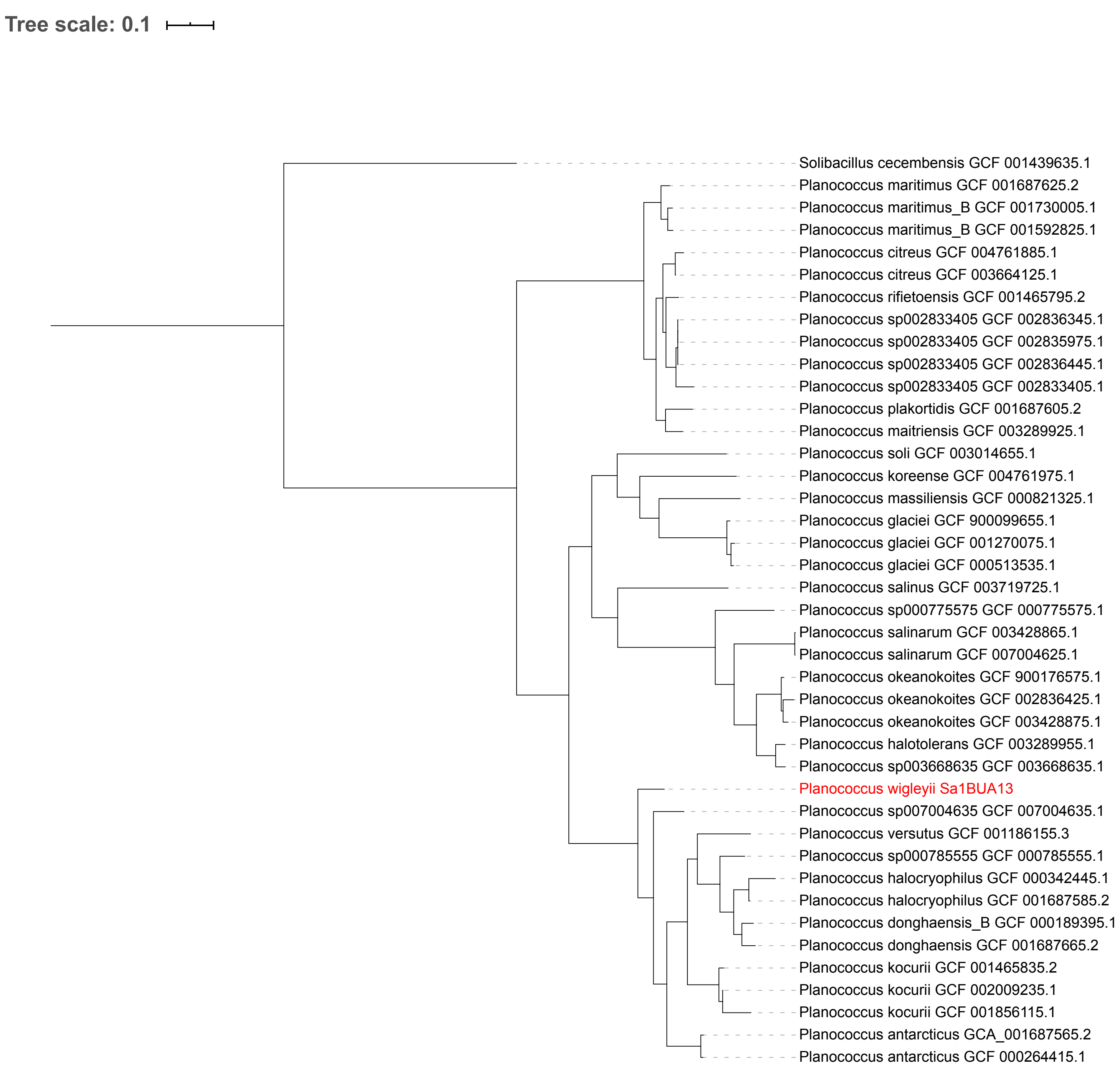

Tree scale: 0.1
Solibacillus cecembensis GCF 001439635.1
Planococcus maritimus GCF 001687625.2
Planococcus maritimus_B GCF 001730005.1
Planococcus maritimus_B GCF 001592825.1
Planococcus citreus GCF 004761885.1
Planococcus citreus GCF 003664125.1
Planococcus rifietoensis GCF 001465795.2
Planococcus sp002833405 GCF 002836345.1
Planococcus sp002833405 GCF 002835975.1
Planococcus sp002833405 GCF 002836445.1
Planococcus sp002833405 GCF 002833405.1
Planococcus plakortidis GCF 001687605.2
Planococcus maitriensis GCF 003289925.1
Planococcus soli GCF 003014655.1
Planococcus koreense GCF 004761975.1
Planococcus massiliensis GCF 000821325.1
Planococcus glaciei GCF 900099655.1
Planococcus glaciei GCF 001270075.1
Planococcus glaciei GCF 000513535.1
Planococcus salinus GCF 003719725.1
Planococcus sp000775575 GCF 000775575.1
Planococcus salinarum GCF 003428865.1
Planococcus salinarum GCF 007004625.1
Planococcus okeanokoites GCF 900176575.1
Planococcus okeanokoites GCF 002836425.1
Planococcus okeanokoites GCF 003428875.1
Planococcus halotolerans GCF 003289955.1
Planococcus sp003668635 GCF 003668635.1
Planococcus wigleyii Sa1BUA13
Planococcus sp007004635 GCF 007004635.1
Planococcus versutus GCF 001186155.3
Planococcus sp000785555 GCF 000785555.1
Planococcus halocryophilus GCF 000342445.1
Planococcus halocryophilus GCF 001687585.2
Planococcus donghaensis_B GCF 000189395.1
Planococcus donghaensis GCF 001687665.2
Planococcus kocurii GCF 001465835.2
Planococcus kocurii GCF 002009235.1
Planococcus kocurii GCF 001856115.1
Planococcus antarcticus GCA_001687565.2
Planococcus antarcticus GCF 000264415.1

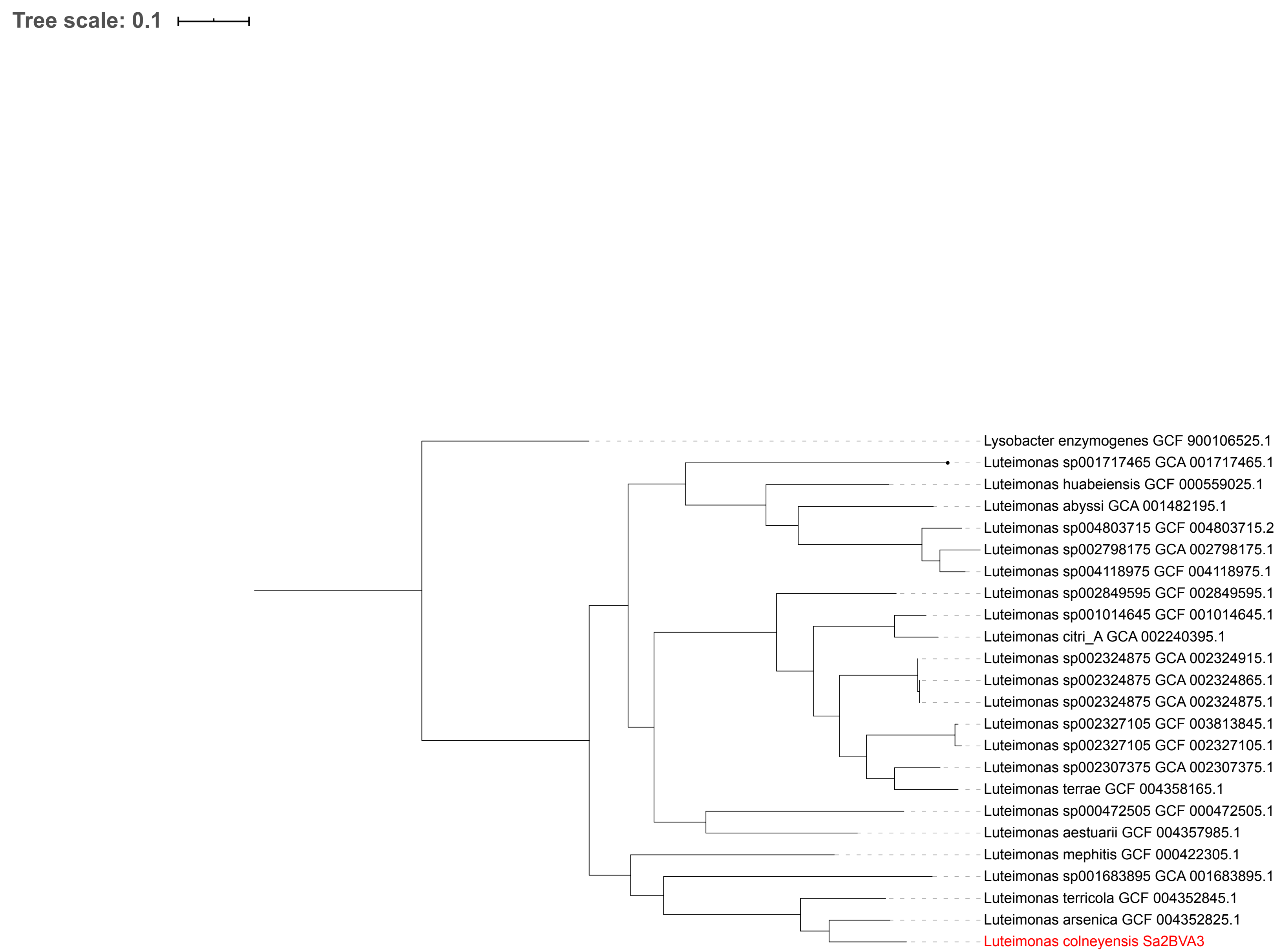
Tree scale: 0.1
Lysobacter enzymogenes GCF 900106525.1
Luteimonas sp001717465 GCA 001717465.1
Luteimonas huabeiensis GCF 000559025.1
Luteimonas abyssi GCA 001482195.1
Luteimonas sp004803715 GCF 004803715.2
Luteimonas sp002798175 GCA 002798175.1
Luteimonas sp004118975 GCF 004118975.1
Luteimonas sp002849595 GCF 002849595.1
Luteimonas sp001014645 GCF 001014645.1
Luteimonas citri_A GCA 002240395.1
Luteimonas sp002324875 GCA 002324915.1
Luteimonas sp002324875 GCA 002324865.1
Luteimonas sp002324875 GCA 002324875.1
Luteimonas sp002327105 GCF 003813845.1
Luteimonas sp002327105 GCF 002327105.1
Luteimonas sp002307375 GCA 002307375.1
Luteimonas terrae GCF 004358165.1
Luteimonas sp000472505 GCF 000472505.1
Luteimonas aestuarii GCF 004357985.1
Luteimonas mephitis GCF 000422305.1
Luteimonas sp001683895 GCA 001683895.1
Luteimonas terricola GCF 004352845.1
Luteimonas arsenica GCF 004352825.1
Luteimonas colneyensis Sa2BVA3

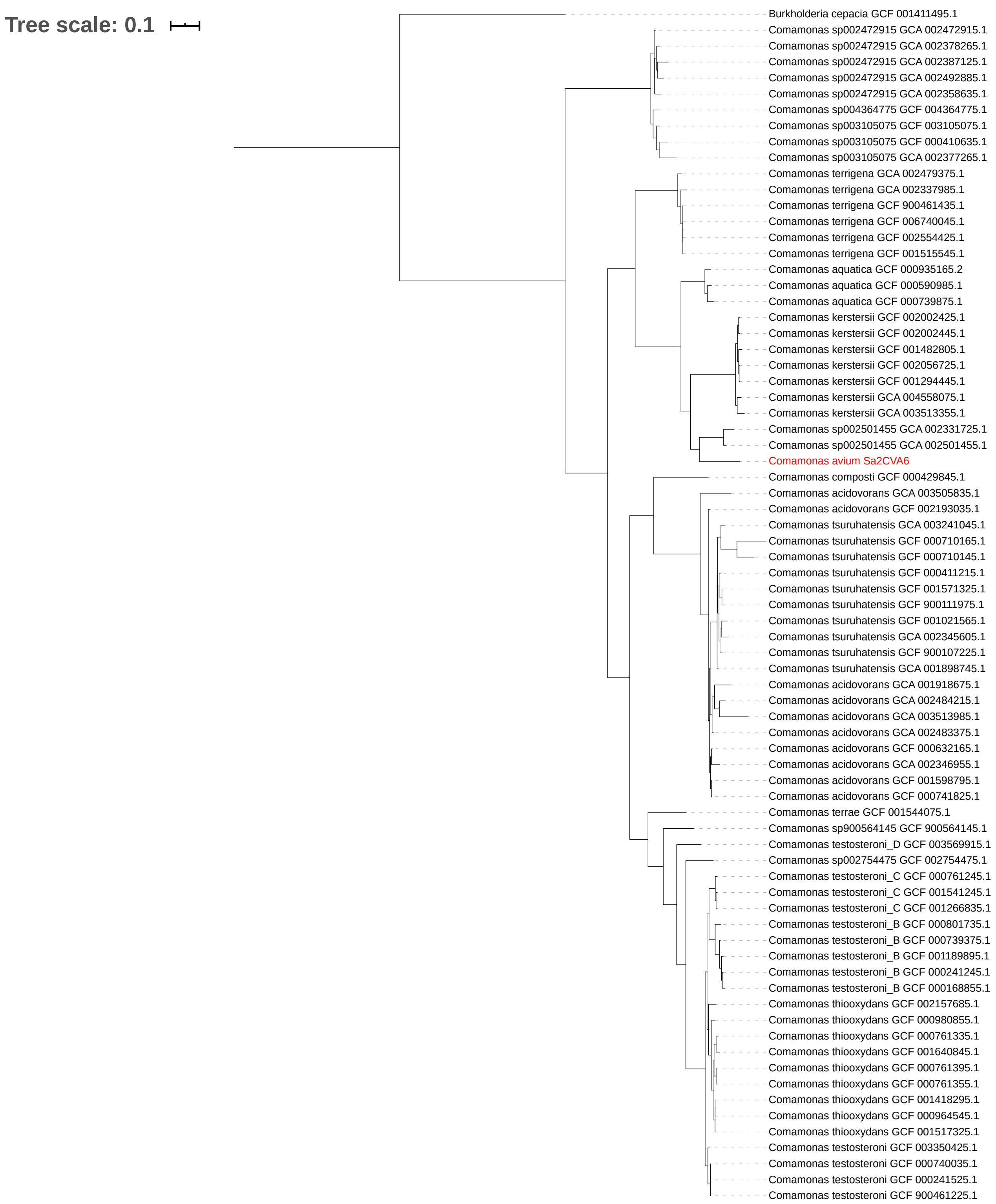
Tree scale: 0.1
Burkholderia cepacia GCF 001411495.1
Comamonas sp002472915 GCA 002472915.1
Comamonas sp002472915 GCA 002378265.1
Comamonas sp002472915 GCA 002387125.1
Comamonas sp002472915 GCA 002492885.1
Comamonas sp002472915 GCA 002358635.1
Comamonas sp004364775 GCF 004364775.1
Comamonas sp003105075 GCF 003105075.1
Comamonas sp003105075 GCF 000410635.1
Comamonas sp003105075 GCA 002377265.1
Comamonas terrigena GCA 002479375.1
Comamonas terrigena GCA 002337985.1
Comamonas terrigena GCF 900461435.1
Comamonas terrigena GCF 006740045.1
Comamonas terrigena GCF 002554425.1
Comamonas terrigena GCF 001515545.1
Comamonas aquatica GCF 000935165.2
Comamonas aquatica GCF 000590985.1
Comamonas aquatica GCF 000739875.1
Comamonas kerstersii GCF 002002425.1
Comamonas kerstersii GCF 002002445.1
Comamonas kerstersii GCF 001482805.1
Comamonas kerstersii GCF 002056725.1
Comamonas kerstersii GCF 001294445.1
Comamonas kerstersii GCA 004558075.1
Comamonas kerstersii GCA 003513355.1
Comamonas sp002501455 GCA 002331725.1
Comamonas sp002501455 GCA 002501455.1
Comamonas avium Sa2CVA6
Comamonas composti GCF 000429845.1
Comamonas acidovorans GCA 003505835.1
Comamonas acidovorans GCF 002193035.1
Comamonas tsuruhatensis GCA 003241045.1
Comamonas tsuruhatensis GCF 000710165.1
Comamonas tsuruhatensis GCF 000710145.1
Comamonas tsuruhatensis GCF 000411215.1
Comamonas tsuruhatensis GCF 001571325.1
Comamonas tsuruhatensis GCF 900111975.1
Comamonas tsuruhatensis GCF 001021565.1
Comamonas tsuruhatensis GCA 002345605.1
Comamonas tsuruhatensis GCF 900107225.1
Comamonas tsuruhatensis GCA 001898745.1
Comamonas acidovorans GCA 001918675.1
Comamonas acidovorans GCA 002484215.1
Comamonas acidovorans GCA 003513985.1
Comamonas acidovorans GCA 002483375.1
Comamonas acidovorans GCF 000632165.1
Comamonas acidovorans GCA 002346955.1
Comamonas acidovorans GCF 001598795.1
Comamonas acidovorans GCF 000741825.1
Comamonas terrae GCF 001544075.1
Comamonas sp900564145 GCF 900564145.1
Comamonas testosteroni_D GCF 003569915.1
Comamonas sp002754475 GCF 002754475.1
Comamonas testosteroni_C GCF 000761245.1
Comamonas testosteroni_C GCF 001541245.1
Comamonas testosteroni_C GCF 001266835.1
Comamonas testosteroni_B GCF 000801735.1
Comamonas testosteroni_B GCF 000739375.1
Comamonas testosteroni_B GCF 001189895.1
Comamonas testosteroni_B GCF 000241245.1
Comamonas testosteroni_B GCF 000168855.1
Comamonas thiooxydans GCF 002157685.1
Comamonas thiooxydans GCF 000980855.1
Comamonas thiooxydans GCF 000761335.1
Comamonas thiooxydans GCF 001640845.1
Comamonas thiooxydans GCF 000761395.1
Comamonas thiooxydans GCF 000761355.1
Comamonas thiooxydans GCF 001418295.1
Comamonas thiooxydans GCF 000964545.1
Comamonas thiooxydans GCF 001517325.1
Comamonas testosteroni GCF 003350425.1
Comamonas testosteroni GCF 000740035.1
Comamonas testosteroni GCF 000241525.1
Comamonas testosteroni GCF 900461225.1

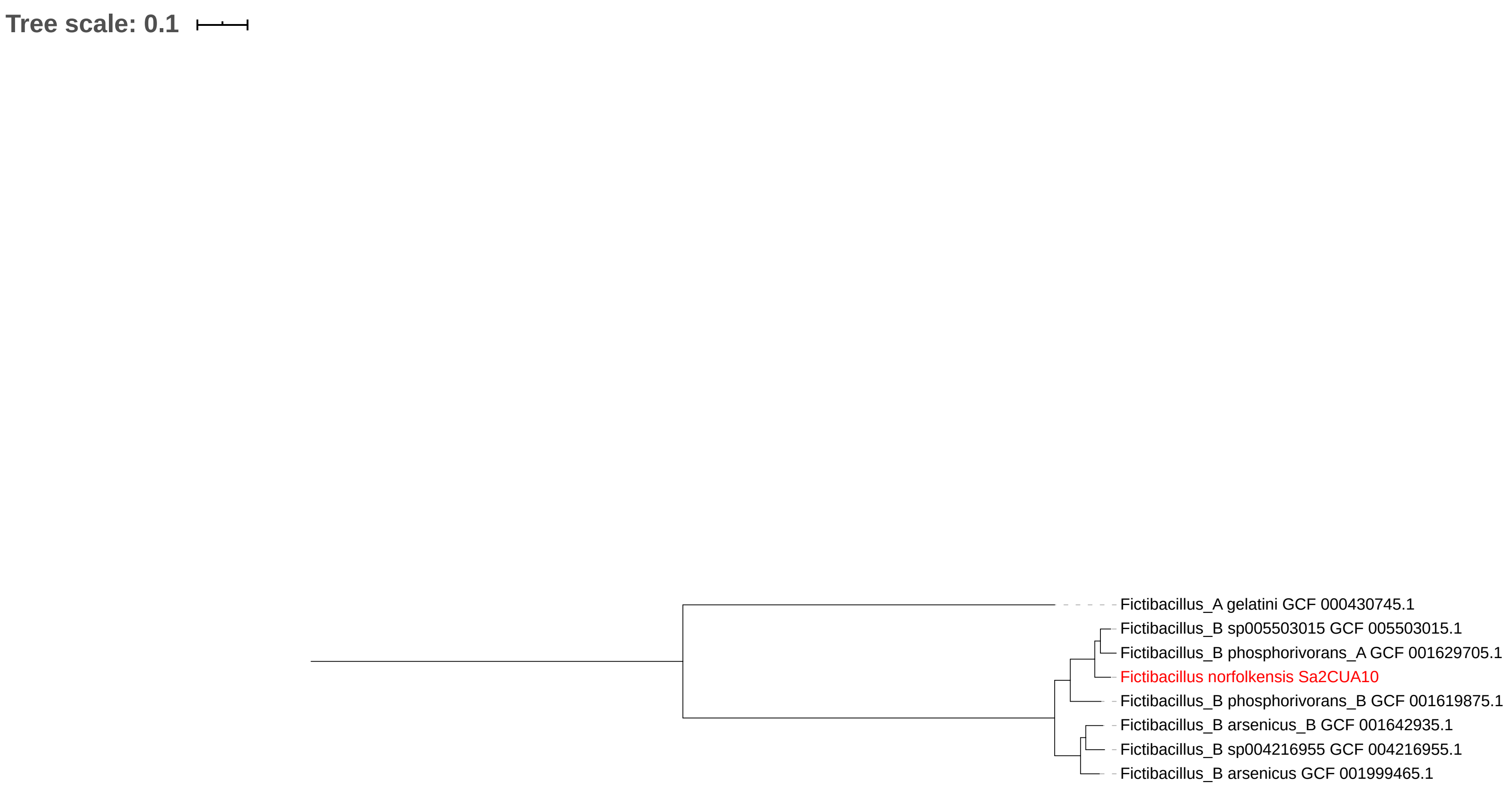
Tree scale: 0.1
Fictibacillus_A gelatini GCF 000430745.1
Fictibacillus_B sp005503015 GCF 005503015.1
Fictibacillus_B phosphorivorans_A GCF 001629705.1
Fictibacillus norfolkensis Sa2CUA10
Fictibacillus_B phosphorivorans_B GCF 001619875.1
Fictibacillus_B arsenicus_B GCF 001642935.1
Fictibacillus_B sp004216955 GCF 004216955.1
Fictibacillus_B arsenicus GCF 001999465.1

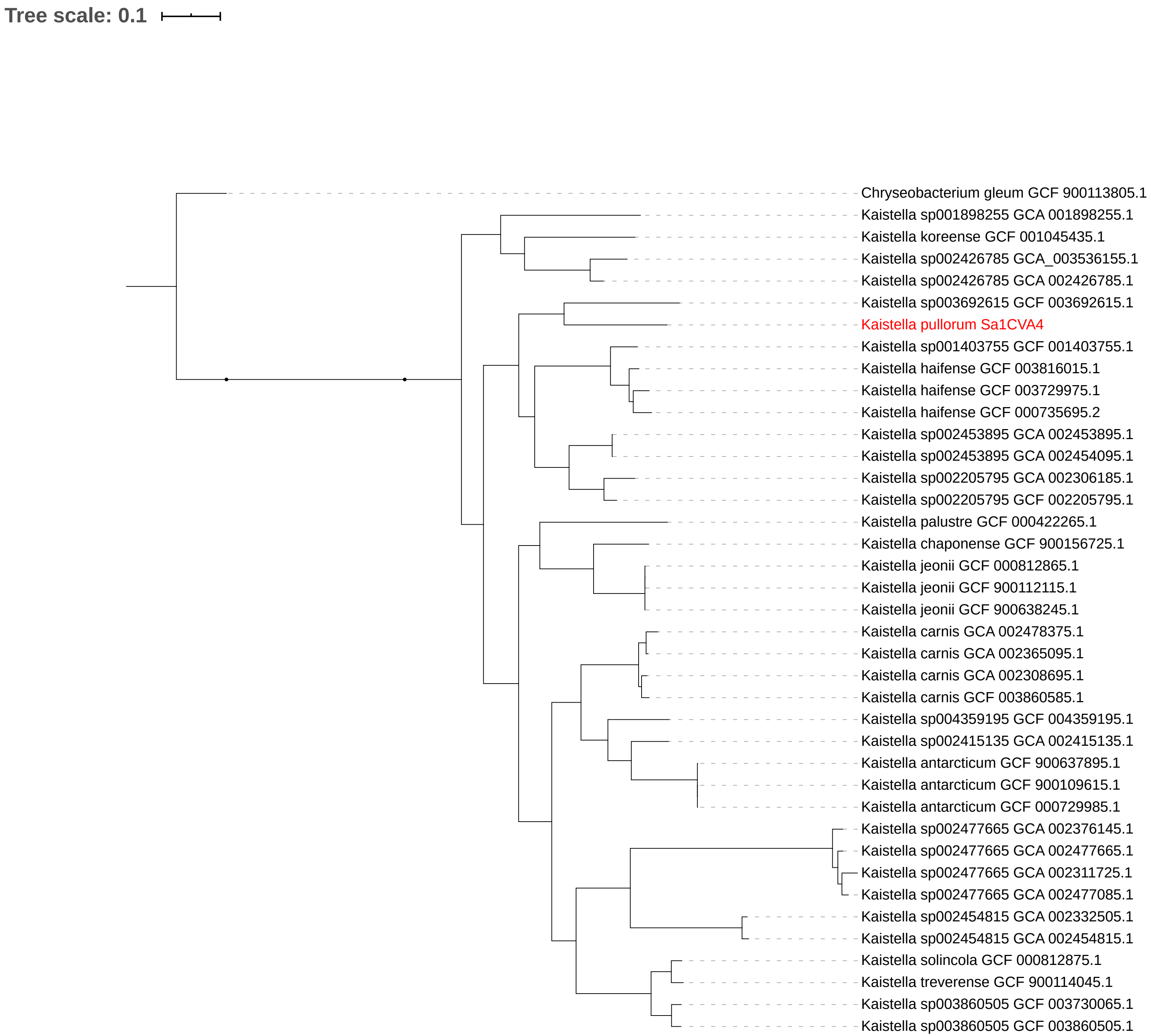
Tree scale: 0.1
Chryseobacterium gleum GCF 900113805.1
Kaistella sp001898255 GCA 001898255.1
Kaistella koreense GCF 001045435.1
Kaistella sp002426785 GCA_003536155.1
Kaistella sp002426785 GCA 002426785.1
Kaistella sp003692615 GCF 003692615.1
Kaistella pullorum Sa1CVA4
Kaistella sp001403755 GCF 001403755.1
Kaistella haifense GCF 003816015.1
Kaistella haifense GCF 003729975.1
Kaistella haifense GCF 000735695.2
Kaistella sp002453895 GCA 002453895.1
Kaistella sp002453895 GCA 002454095.1
Kaistella sp002205795 GCA 002306185.1
Kaistella sp002205795 GCF 002205795.1
Kaistella palustre GCF 000422265.1
Kaistella chaponense GCF 900156725.1
Kaistella jeonii GCF 000812865.1
Kaistella jeonii GCF 900112115.1
Kaistella jeonii GCF 900638245.1
Kaistella carnis GCA 002478375.1
Kaistella carnis GCA 002365095.1
Kaistella carnis GCA 002308695.1
Kaistella carnis GCF 003860585.1
Kaistella sp004359195 GCF 004359195.1
Kaistella sp002415135 GCA 002415135.1
Kaistella antarcticum GCF 900637895.1
Kaistella antarcticum GCF 900109615.1
Kaistella antarcticum GCF 000729985.1
Kaistella sp002477665 GCA 002376145.1
Kaistella sp002477665 GCA 002477665.1
Kaistella sp002477665 GCA 002311725.1
Kaistella sp002477665 GCA 002477085.1
Kaistella sp002454815 GCA 002332505.1
Kaistella sp002454815 GCA 002454815.1
Kaistella solincola GCF 000812875.1
Kaistella treverense GCF 900114045.1
Kaistella sp003860505 GCF 003730065.1
Kaistella sp003860505 GCF 003860505.1

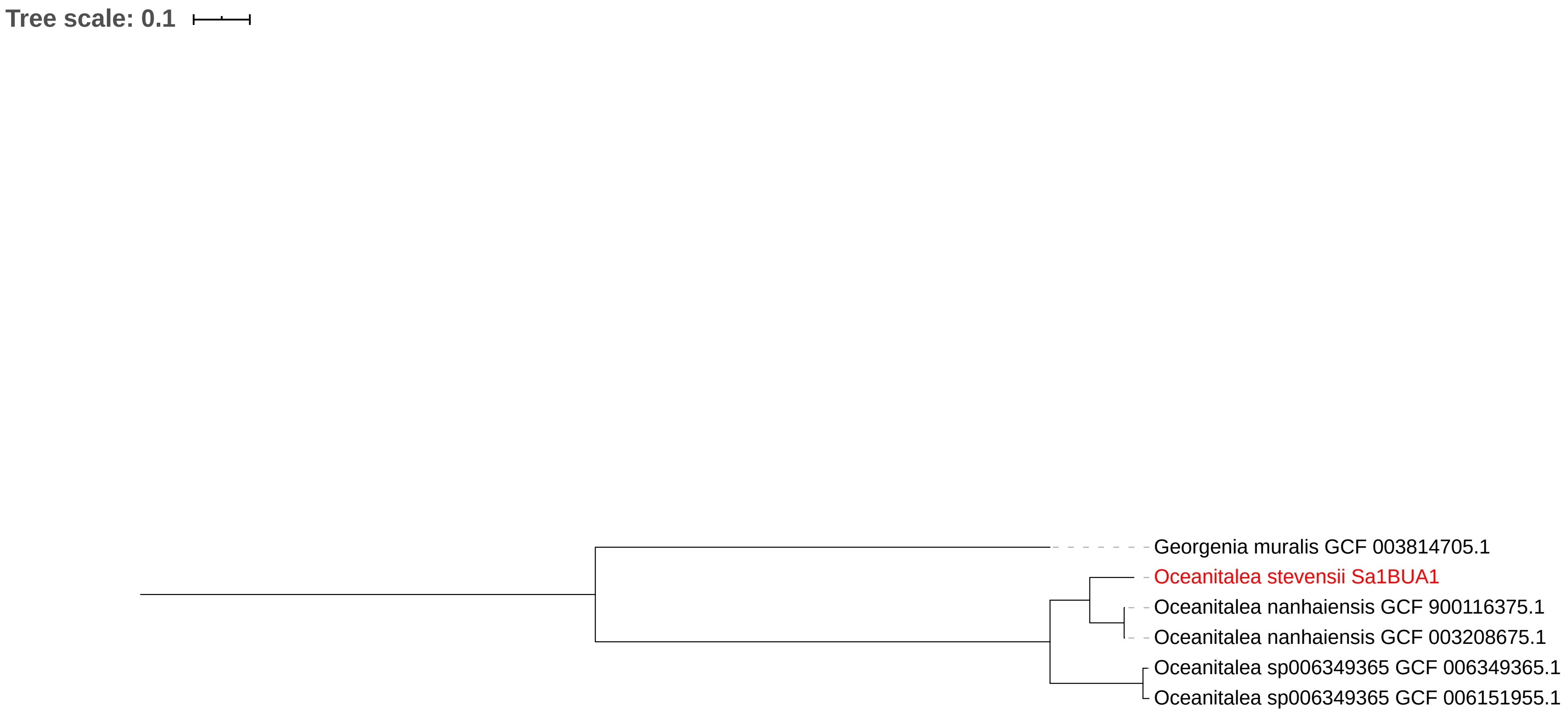
Tree scale: 0.1
Georgenia muralis GCF 003814705.1
Oceanitalea stevensii Sa1BUA1
Oceanitalea nanhaiensis GCF 900116375.1
Oceanitalea nanhaiensis GCF 003208675.1
Oceanitalea sp006349365 GCF 006349365.1
Oceanitalea sp006349365 GCF 006151955.1

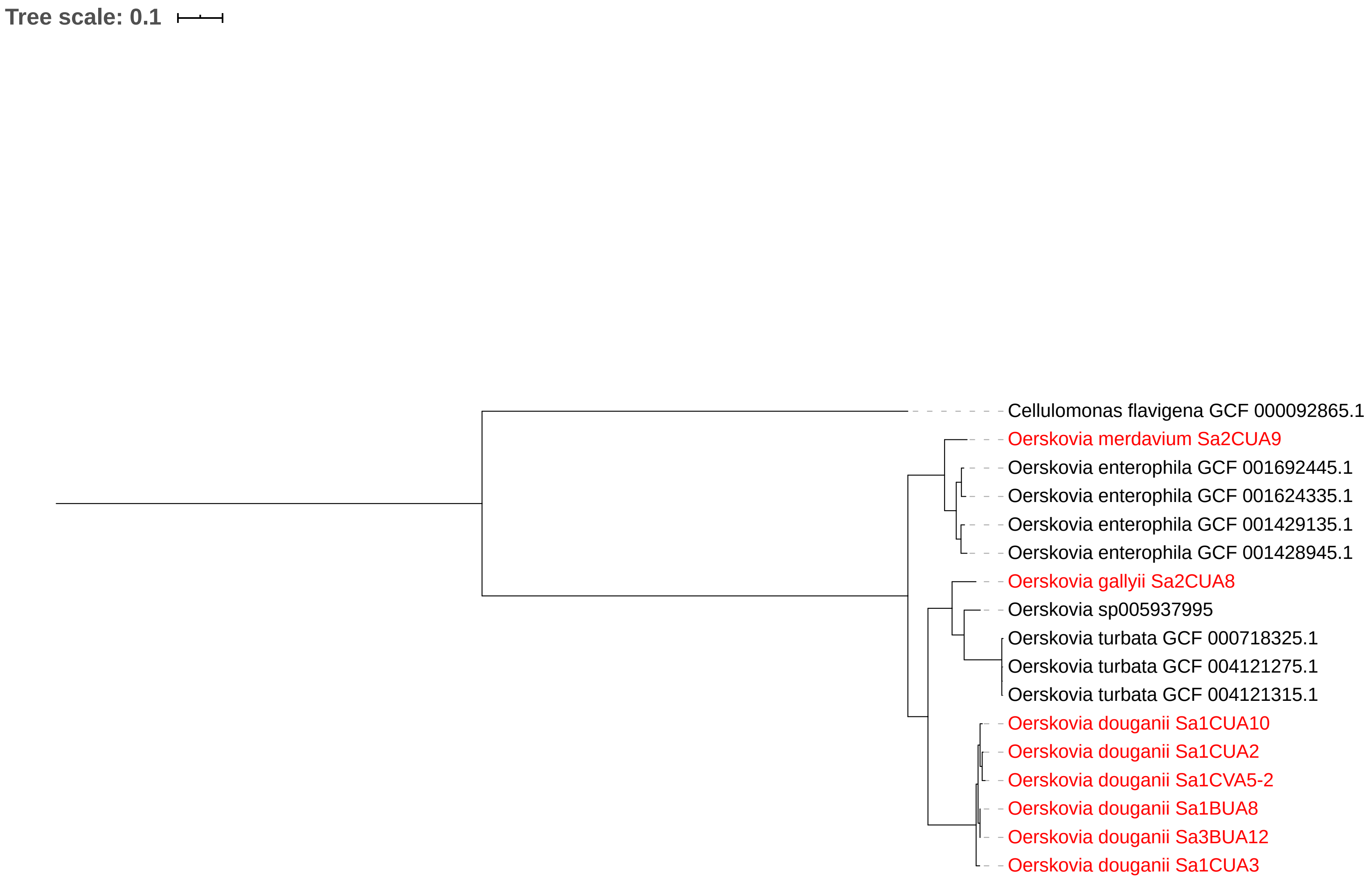
Tree scale: 0.1
Cellulomonas flavigena GCF 000092865.1
Oerskovia merdavium Sa2CUA9
Oerskovia enterophila GCF 001692445.1
Oerskovia enterophila GCF 001624335.1
Oerskovia enterophila GCF 001429135.1
Oerskovia enterophila GCF 001428945.1
Oerskovia gallyii Sa2CUA8
Oerskovia sp005937995
Oerskovia turbata GCF 000718325.1
Oerskovia turbata GCF 004121275.1
Oerskovia turbata GCF 004121315.1
Oerskovia douganii Sa1CUA10
Oerskovia douganii Sa1CUA2
Oerskovia douganii Sa1CVA5-2
Oerskovia douganii Sa1BUA8
Oerskovia douganii Sa3BUA12
Oerskovia douganii Sa1CUA3

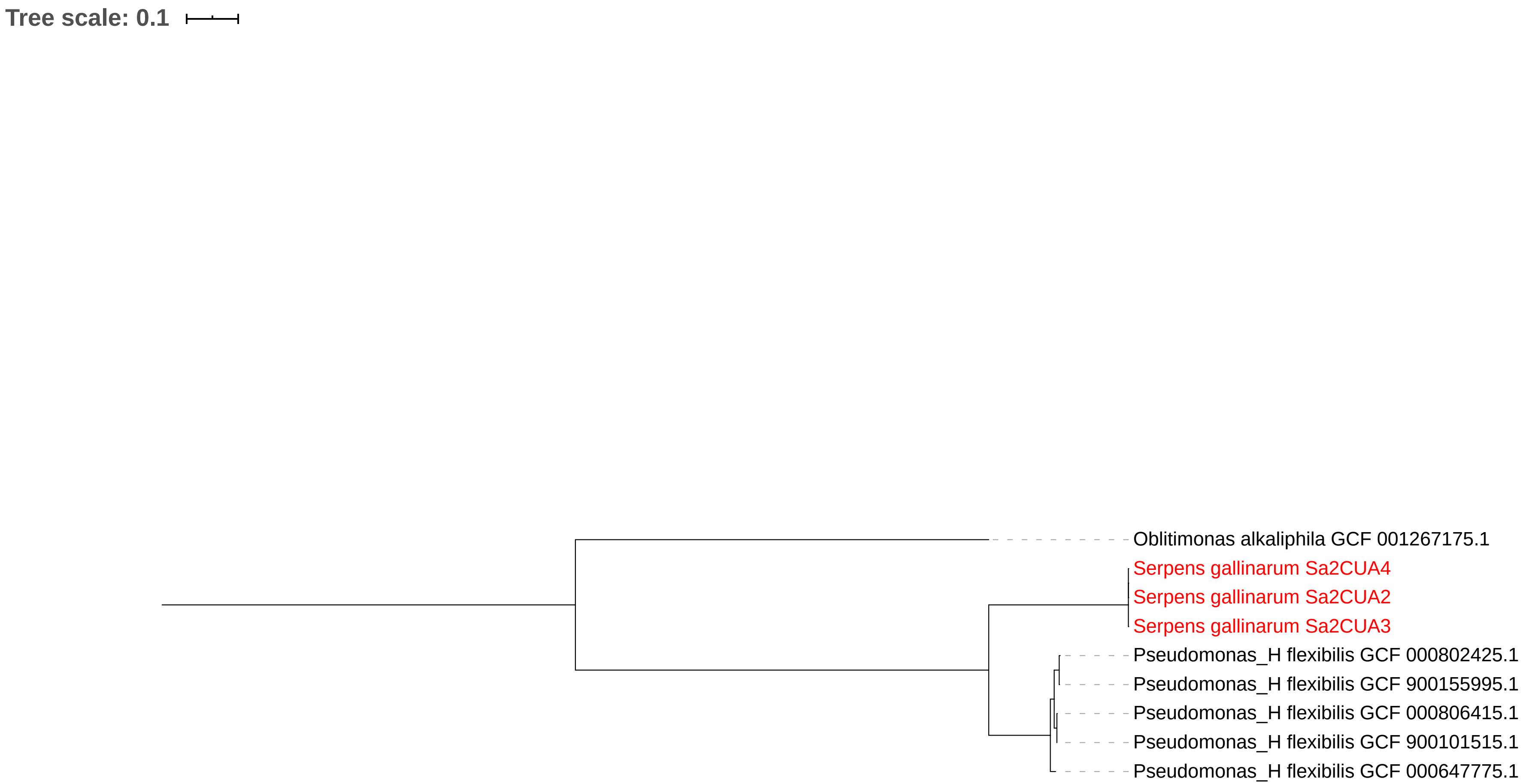

Tree scale: 0.1
Oblitimonas alkaliphila GCF 001267175.1
Serpens gallinarum Sa2CUA4
Serpens gallinarum Sa2CUA2
Serpens gallinarum Sa2CUA3
Pseudomonas_H flexibilis GCF 000802425.1
Pseudomonas_H flexibilis GCF 900155995.1
Pseudomonas_H flexibilis GCF 000806415.1
Pseudomonas_H flexibilis GCF 900101515.1
Pseudomonas_H flexibilis GCF 000647775.1

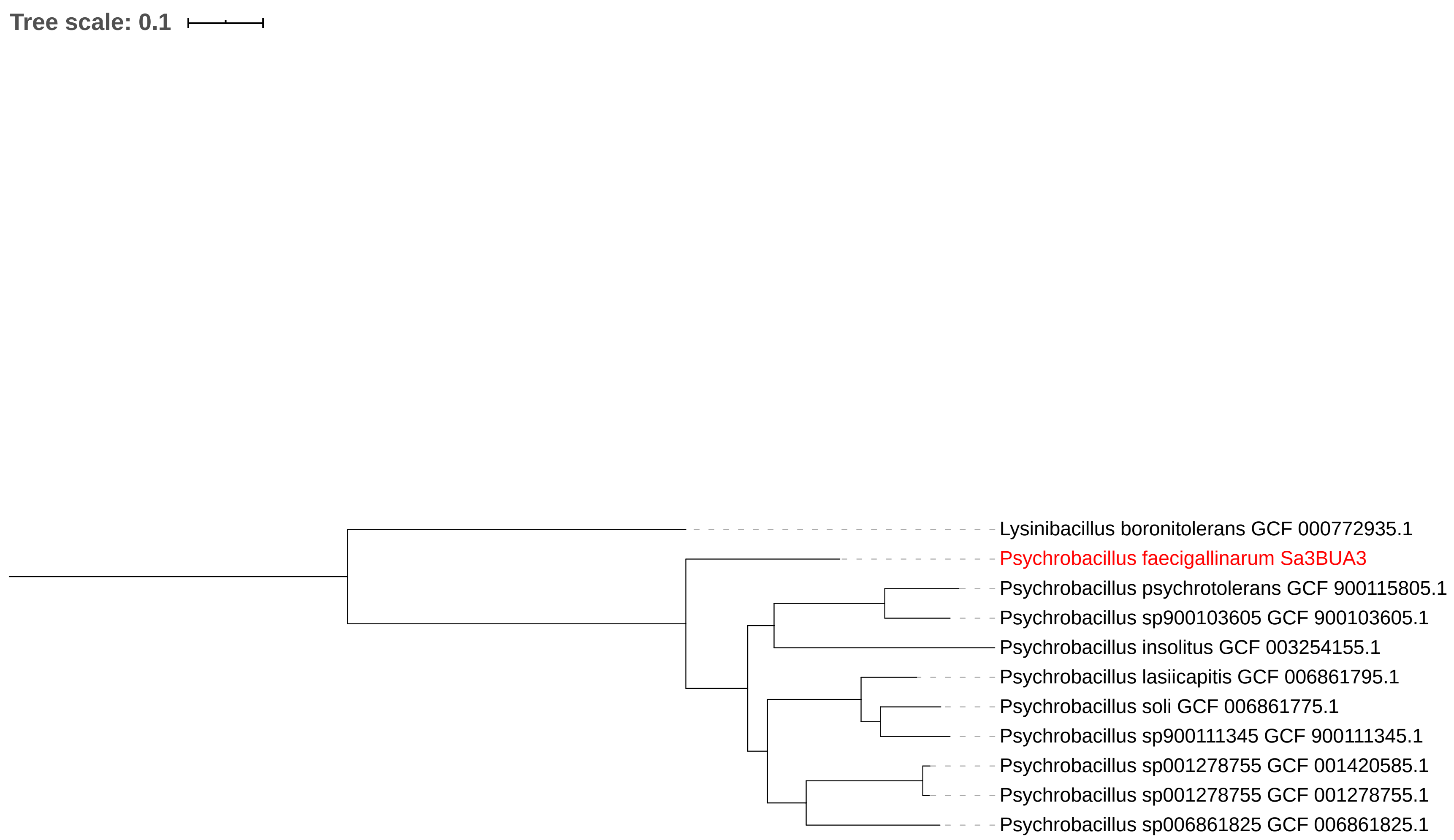
Tree scale: 0.1
Lysinibacillus boronitolerans GCF 000772935.1
Psychrobacillus faecigallinarum Sa3BUA3
Psychrobacillus psychrotolerans GCF 900115805.1
Psychrobacillus sp900103605 GCF 900103605.1
Psychrobacillus insolitus GCF 003254155.1
Psychrobacillus lasiicapitis GCF 006861795.1
Psychrobacillus soli GCF 006861775.1
Psychrobacillus sp900111345 GCF 900111345.1
Psychrobacillus sp001278755 GCF 001420585.1
Psychrobacillus sp001278755 GCF 001278755.1
Psychrobacillus sp006861825 GCF 006861825.1

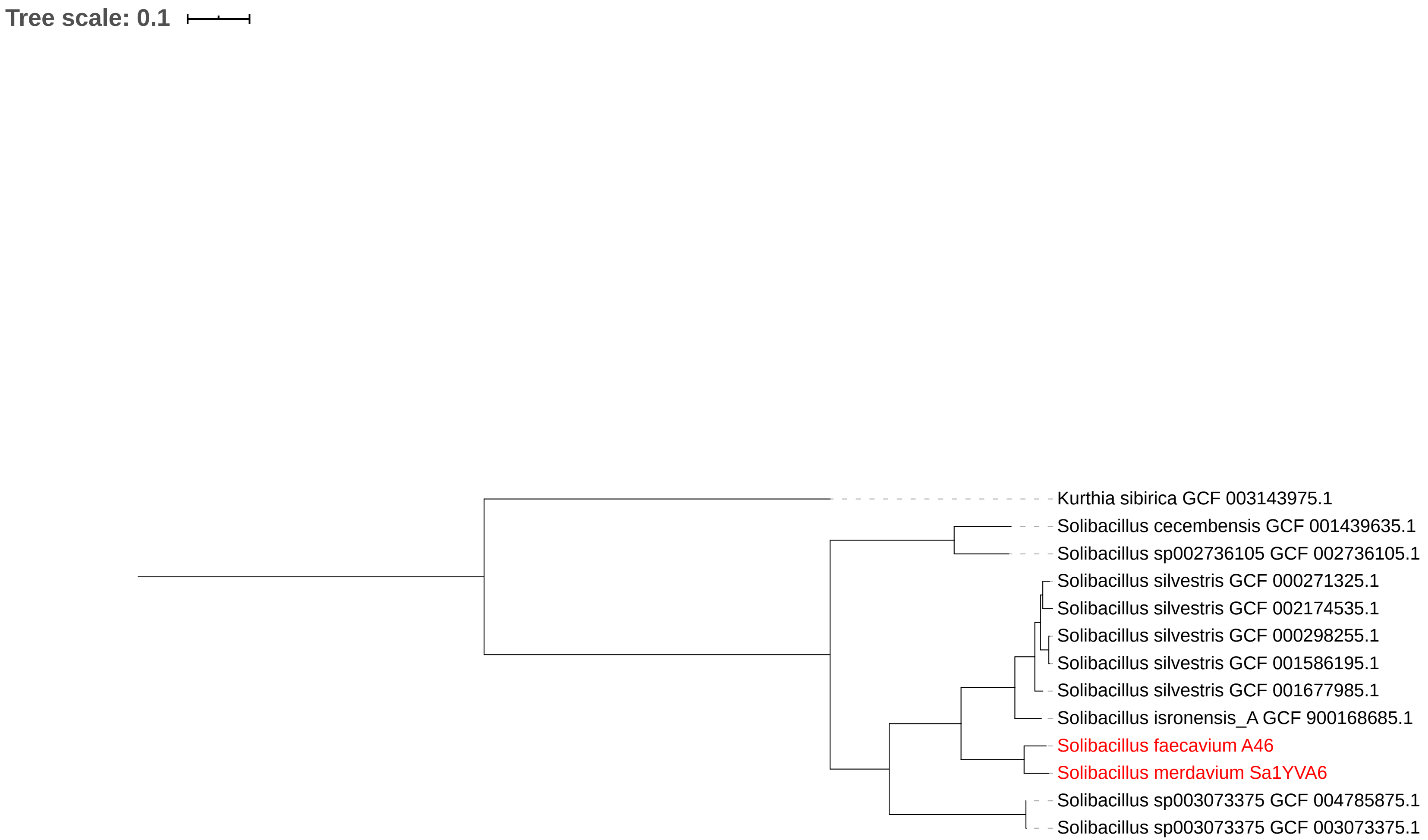

Tree scale: 0.1
Kurthia sibirica GCF 003143975.1
Solibacillus cecembensis GCF 001439635.1
Solibacillus sp002736105 GCF 002736105.1
Solibacillus silvestris GCF 000271325.1
Solibacillus silvestris GCF 002174535.1
Solibacillus silvestris GCF 000298255.1
Solibacillus silvestris GCF 001586195.1
Solibacillus silvestris GCF 001677985.1
Solibacillus isronensis_A GCF 900168685.1
Solibacillus faecavium A46
Solibacillus merdavium Sa1YVA6
Solibacillus sp003073375 GCF 004785875.1
Solibacillus sp003073375 GCF 003073375.1

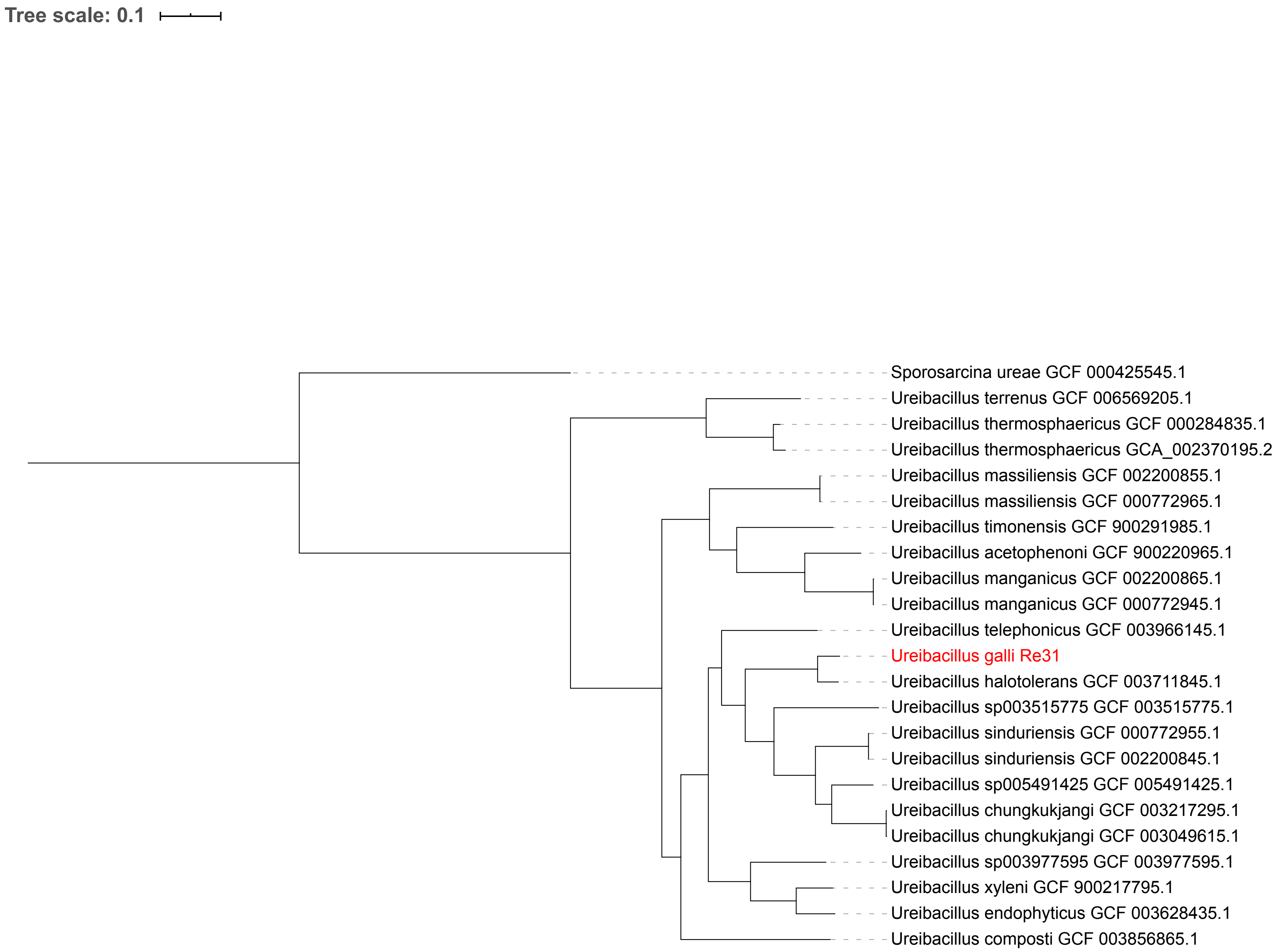

Figure S2. Phylogenetic trees reconstructed for genomes of each novel cultured isolate against all available reference genomes of the respective genus obtained from NCBI. Trees were reconstructed using PhyloPhlAn 3.0.58 against 400 marker genes before reconstruction using FastTree and RAxML of a MAFFT sequence alignment. Genomes of isolates cultured in this study have been highlighted in red and detailed with their respective strain identifier.

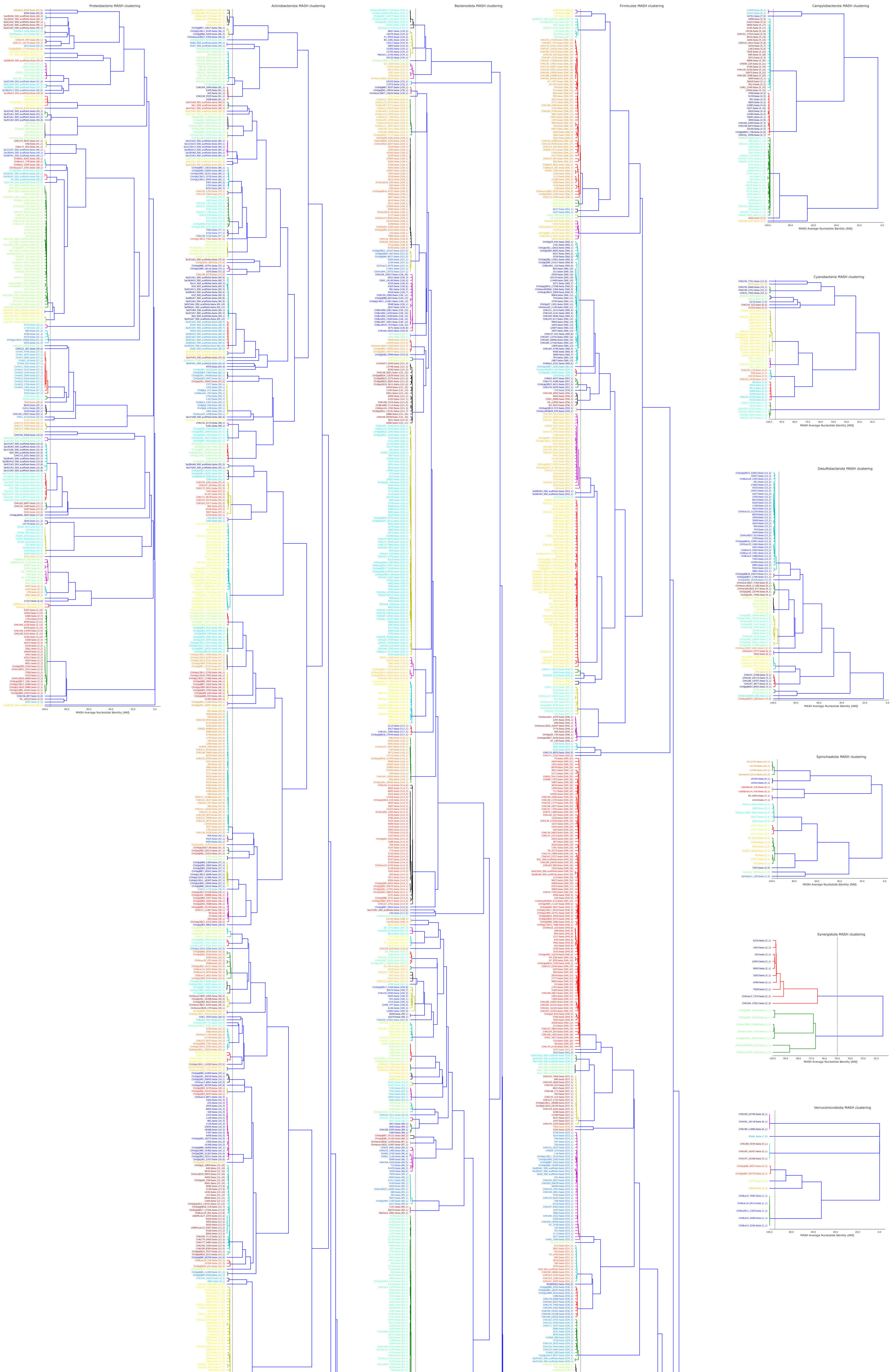
Proteobacteria MASH clustering
MASH Average Nucleotide Identity (ANI)
Actinobacteriota MASH clustering
Bacteroidota MASH clustering
Firmicutes MASH clustering
Campylobacterota MASH clustering
MASH Average Nucleotide Identity (ANI)
Cyanobacteria MASH clustering
MASH Average Nucleotide Identity (ANI)
Desulfobacterota MASH clustering
MASH Average Nucleotide Identity (ANI)
Spirochaetota MASH clustering
MASH Average Nucleotide Identity (ANI)
Synergistota MASH clustering
MASH Average Nucleotide Identity (ANI)
Verrucomicrobiota MASH clustering
MASH Average Nucleotide Identity (ANI)

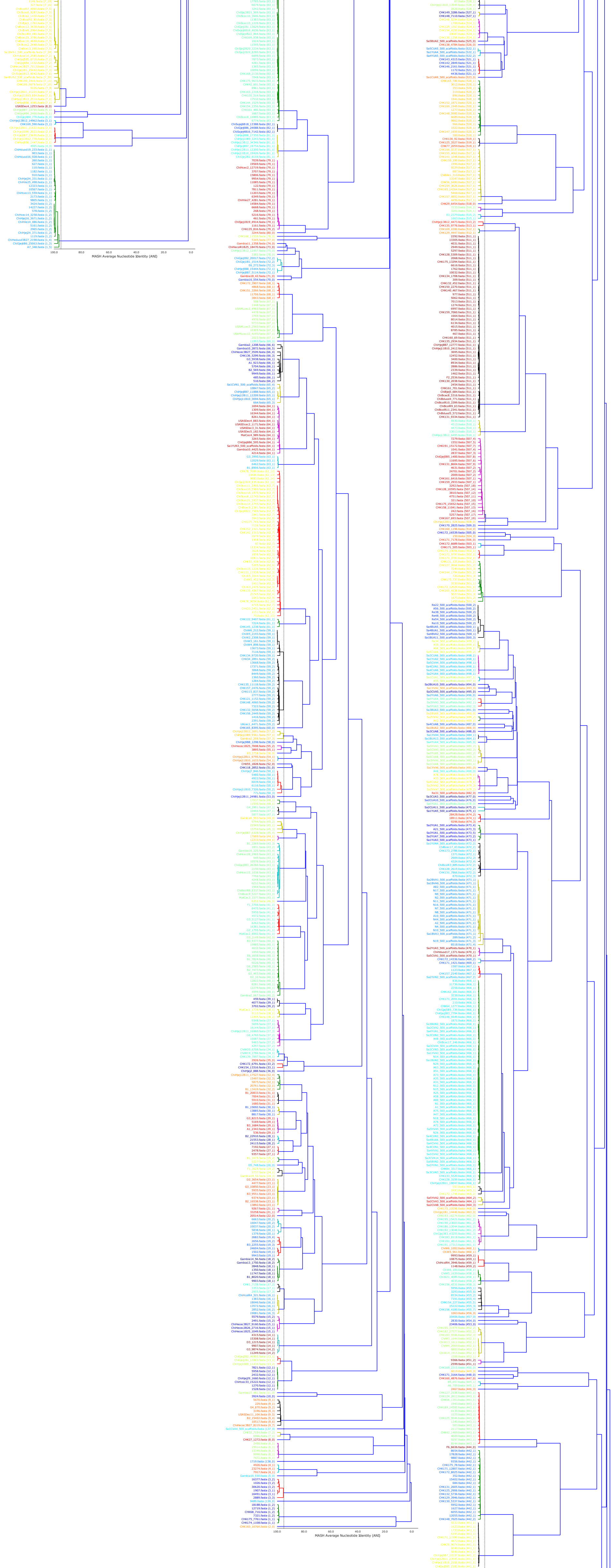
100.0
80.0
60.0
40.0
20.0
0.0
MASH Average Nucleotide Identity (ANI)
100.0
80.0
60.0
40.0
20.0
0.0
MASH Average Nucleotide Identity (ANI)

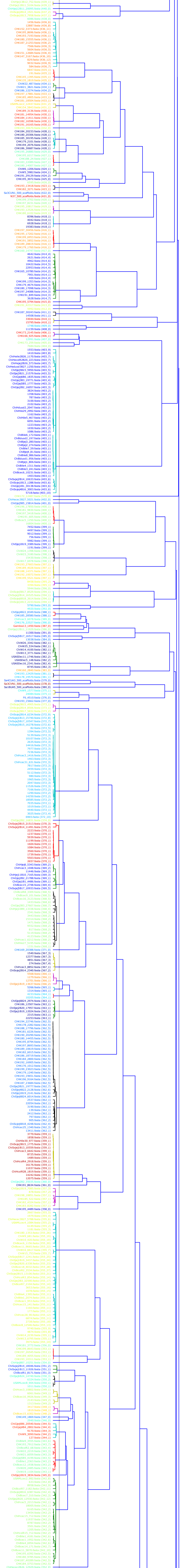

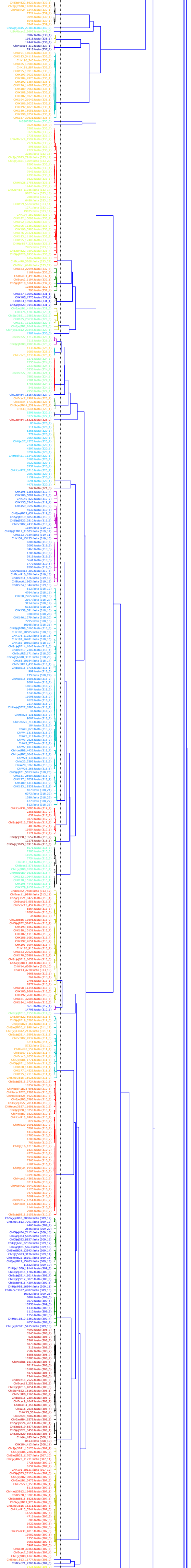

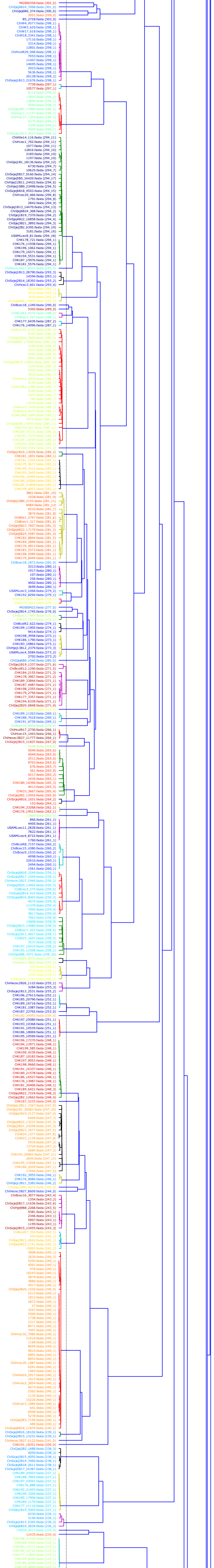

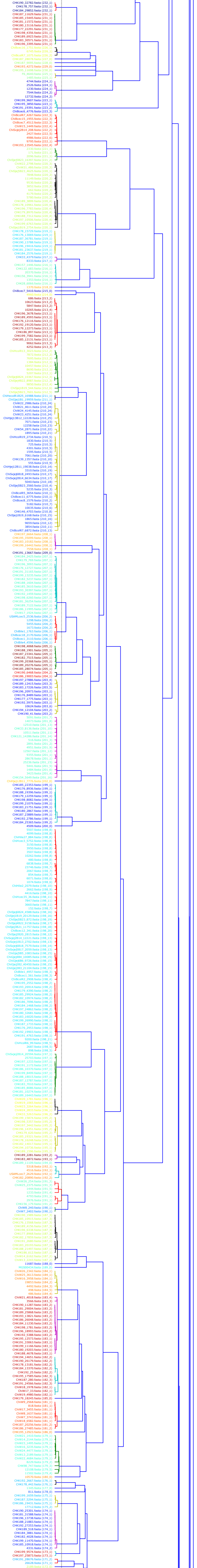

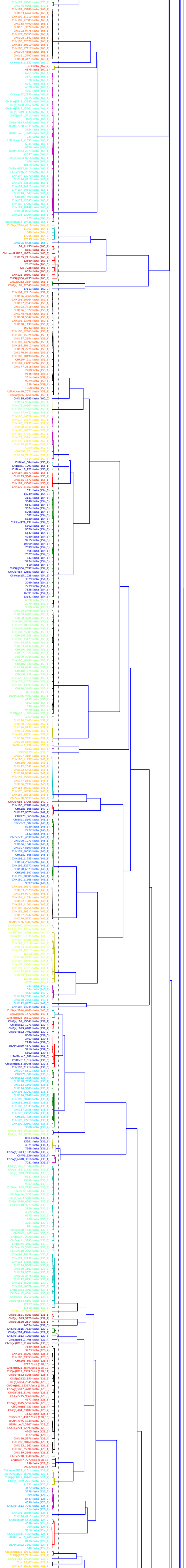

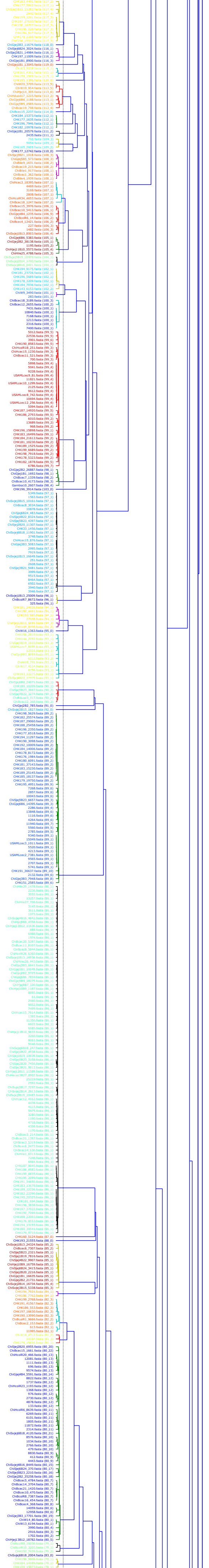

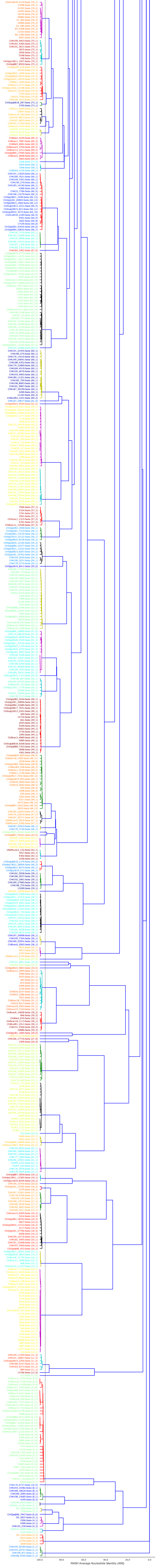

Figure S3 Primary ANI clustering of all MAGs and cultured isolates separated according to phylum as determined by dREP. Each plot clusters at species-level according to 95% ANI, as shown by a continual dotted line. Sequence clusters are assigned colours according to designated species-level cluster.

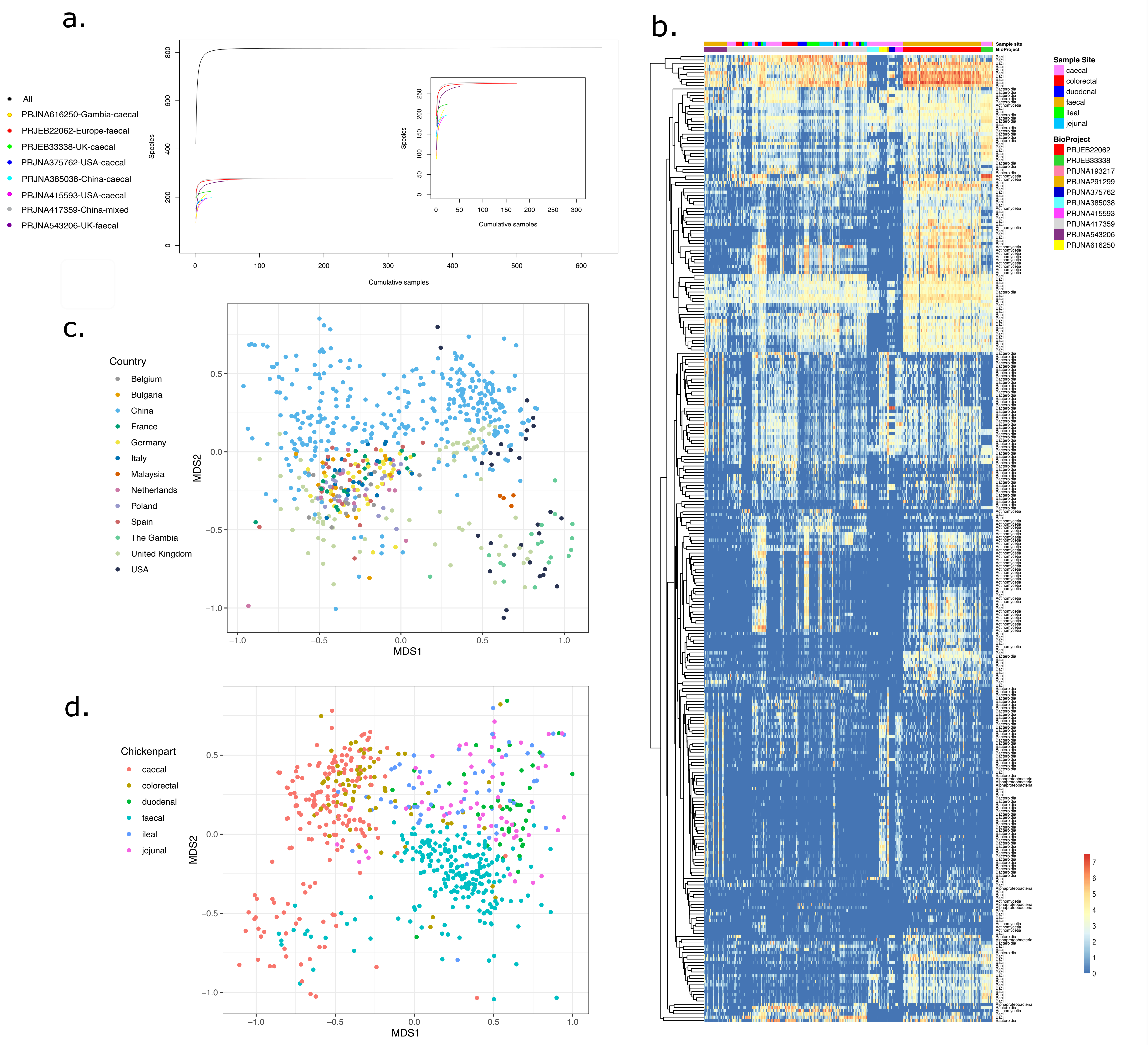

Figure S4a Species accumulation curve for 632 samples over 10 publicly available BioProjects accounting for 820 MGS. Only studies with > 1 sample are included in this plot. A further curve has been plotted to depict species accumulation for all metagenomic samples from all BioProjects (.pdf format).**b**. A heatmap depicting the abundance of the top 300 MGS, ordered by maximum sample abundance, across 632 samples derived from 10 BioProjects. Rows (MGS) were ordered by correlation clustering while columns (metagenomic sample) were ordered by source BioProject. Metagenomic species have been annotated with their taxonomic class. All data were Log10 transformed with Blue colour depicting species of low abundance and Red showing high abundance.**c**. Principle component analysis (PCA) based on Bray Curtis dissimilarity matrices between country of origin for MGS relative abundance. d. and for GIT sample site
